# Supplementary material for: The range and reach of qualitative research in neurosurgery: A scoping review
Source: PLoS One. 2025 Aug 21;20(8):e0330770. doi: 10.1371/journal.pone.0330770 (PMC12370046; doi:10.1371/journal.pone.0330770)
Supplement: S2 Table — Full electronic search strategy deployed in Medline. (DOCX) [file pone.0330770.s002.docx]

**Full electronic Search Strategy for Medline**

| **#** | **Query** |
| --- | --- |
| S10 | S1 AND S2 AND S9 |
| S9 | S3 OR S4 OR S5 |
| S8 | S6 OR S7 |
| S7 | (MH "Cervical Vertebrae+") OR (MH "Spine+") OR (MH "Brachial Plexus+") OR (MH "Cauda Equina Syndrome") OR (MH "Intervertebral Disc Displacement") OR (MH "Spinal Stenosis") OR (MH "Neck Pain") OR (MH "Scoliosis") OR (MH "Spinal Neoplasms") OR (MH "Tarlov Cysts") OR (MH "Fractures, Compression") OR (MH "Brain Concussion+") OR (MH "Astrocytoma+") OR (MH "Brain Mapping+") OR (MH "Brain Neoplasms+") OR (MH "Craniopharyngioma") OR (MH "Pituitary ACTH Hypersecretion") OR (MH "Glioblastoma") OR (MH "Meningioma") OR (MH "Neurofibromatoses+") OR (MH "Pituitary Neoplasms+") OR (MH "Neurilemmoma+") OR (MH "Neuralgia+") OR (MH "Muscle Spasticity") OR (MH "Trigeminal Neuralgia") OR (MH "Craniosynostoses+") OR (MH "Craniofacial Abnormalities+") OR (MH "Hydrocephalus+") OR (MH "Medulloblastoma") OR (MH "Infratentorial Neoplasms+") OR (MH "Spinal Dysraphism+") OR (MH "Neural Tube Defects+") OR (MH "Ventriculoperitoneal Shunt") OR (MH "Dystonia+") OR (MH "Epilepsy+") OR (MH "Movement Disorders+") OR (MH "Parkinson Disease") OR (MH "Vagus Nerve Stimulation") OR (MH "Seizures+") OR (MH "Arteriovenous Malformations+") OR (MH "Carotid Stenosis") OR (MH "Endarterectomy, Carotid") OR (MH "Hemangioma, Cavernous, Central Nervous System") OR (MH "Intracranial Aneurysm") OR (MH "Cerebrovascular Disorders+") OR (MH "Intracranial Hemorrhages+") |
| S6 | (MH "Surgical Procedures, Operative+") |
| S5 | TI( ( (Spine n3 (cervical OR thoracic OR lumbar OR lumbosacral)) or “Spinal Cord” or nerve or “brachial plexus” or “Cauda Equina Syndrome” or (“Herniated Disc” n3 (cervical OR thoracic OR lumbar OR lumbosacral)) or “Spinal Stenosis” or “Neck Pain” or Scoliosis or “Spin* Infection*” or “Tarlov Cyst” or “Vertebral Compression Fracture*” or concussion or Astrocytoma or “Brain mapping” or “Brain Metastas*” or ((Brain or head* or pituitary or cerebral or “Posterior fossa” or Infratentorial or spine or spinal) n2 (Tumo* or cancer* or neoplasm*)) or Craniopharyngioma* or “Cushing* Disease” or ("pituitary ACTH hypersecretion") or “Glioblastoma” or "Meningioma*" or "Neurofibromatos*" or “Brain Biopsy” or Schwannoma or Neurilemmoma or Spasticity or “Trigeminal Neuralgia” or “Chiari Malformation*” or Craniosynostos* or “Craniofacial Disorder*” or "Hydrocephalus" or "Medulloblastoma*" or “Spina Bifida” or “Tethered Spinal Cord Syndrome” or “Neural Tube Defect*” or “Ventriculoperitoneal Shunt” or (Shunt n3 (Infection* or Malfunction*)) or “Dystonia” or “Movement Disorder*” or “Parkinson* Disease” or “Vagus Nerve Stimulation” or “Epilepsy” or “seizure*” or “Arteriovenous Malformation*” or “Carotid Endarterectomy” or “carotid Stenosis” or “Cavernous Malformation*” or ((Intracerebral or intracranial or cerebral) n1 (aneurysm or Hemorrhag* or Haemorrhag*))) n5 (surger* or surgical* or operate* or operation* or (brain surgeon*) or neurosurg*) )) |
| S4 | AB(( ( (Spine n3 (cervical OR thoracic OR lumbar OR lumbosacral)) or “Spinal Cord” or nerve or “brachial plexus” or “Cauda Equina Syndrome” or (“Herniated Disc” n3 (cervical OR thoracic OR lumbar OR lumbosacral)) or “Spinal Stenosis” or “Neck Pain” or Scoliosis or “Spin* Infection*” or “Tarlov Cyst” or “Vertebral Compression Fracture*” or concussion or Astrocytoma or “Brain mapping” or “Brain Metastas*” or ((Brain or head* or pituitary or cerebral or “Posterior fossa” or Infratentorial or spine or spinal) n2 (Tumo* or cancer* or neoplasm*)) or Craniopharyngioma* or “Cushing* Disease” or ("pituitary ACTH hypersecretion") or “Glioblastoma” or "Meningioma*" or "Neurofibromatos*" or “Brain Biopsy” or Schwannoma or Neurilemmoma or Spasticity or “Trigeminal Neuralgia” or “Chiari Malformation*” or Craniosynostos* or “Craniofacial Disorder*” or "Hydrocephalus" or "Medulloblastoma*" or “Spina Bifida” or “Tethered Spinal Cord Syndrome” or “Neural Tube Defect*” or “Ventriculoperitoneal Shunt” or (Shunt n3 (Infection* or Malfunction*)) or “Dystonia” or “Movement Disorder*” or “Parkinson* Disease” or “Vagus Nerve Stimulation” or “Epilepsy” or “seizure*” or “Arteriovenous Malformation*” or “Carotid Endarterectomy” or “carotid Stenosis” or “Cavernous Malformation*” or ((Intracerebral or intracranial or cerebral) n1 (aneurysm or Hemorrhag* or Haemorrhag*))) n5 (surger* or surgical* or operate* or operation* or (brain surgeon*) or neurosurg*) )) |
| S3 | ( (MH "Brain Injuries+") OR (MH "Intracranial Aneurysm") OR (MH "Intracranial Arterial Diseases+") OR (MH "Cerebrovascular Disorders+") OR (MH "Cerebrovascular Trauma+") ) OR ( (MH "Neurosurgery") OR (MH "Neurosurgical Procedures+") ) OR ( AB (“Deep Brain Stimulation” or “Stereotactic Radiosurgery” ) OR TI (“Deep Brain Stimulation” or “Stereotactic Radiosurgery” ) ) OR (MH "Deep Brain Stimulation") OR ( AB (Craniotomy or intracranial) OR TI (Craniotomy or intracranial) ) OR (MH "Craniotomy+") OR ( TI(((Brain or Cerebro* or head*) n3 (disord* or trauma* or damage or injur*))) or AB((((Brain or Cerebro* or head*) n3 (disord* or trauma* or damage or injur*))) ) OR ( TI(((operat* or surg* or procedur*) n3 (brain* or neuro* or head*))) or AB(((operat* or surg* or procedur*) n3 (brain* or neuro* or head*))) ) OR ( AB Neurosurg* OR TI Neurosurg* )OR ( AB tbi OR TI tbi ) or TI (Craniotom* or craniectom* or hemicraniectom* or hemi-craniectom* or “burr hole*” or laminectom* or discectom* or diskectom*) or AB (Craniotom* or craniectom* or hemicraniectom* or hemi-craniectom* or “burr hole*” or laminectom* or discectom* or diskectom*) or (MH "Craniotomy+") OR (MH "Laminectomy") OR (MH "Diskectomy+") |
| S2 | ( ( TI(clinician* or physician* or doctor* or trainee* or consultant* or attending* or surgeon* or neurosurgeon*) or AB (clinician* or physician* or doctor* or trainee* or consultant* or attending* or surgeon* or neurosurgeon*) ) OR ( (MH "Physicians") OR (MH "Consultants") OR (MH "Medical Staff, Hospital") OR (MH "Neurosurgeons") OR (MH "Surgeons") ) ) OR ( ( TI (patient* or “service user*” or famil* or parent* or spous* or sibling or mother* or father* or parent* or carer* or caregiver* or “care giver*”) or AB (patient* or “service user*” or famil* or parent* or spous* or sibling or mother* or father* or parent* or carer* or caregiver* or “care giver*”) ) OR ( (MH "Family") OR (MH "Adult Children") OR (MH "Family Relations") OR (MH "Parents") OR (MH "Siblings") OR (MH "Spouses") OR (MH "Fathers") OR (MH "Mothers") or (MM "Caregivers") or (MH Patients) ) ) |
| S1 | ( TI ("case stud*" OR Case-stud* or “constant compar*” or “content analysis” or ethnograph* or "discourse* analys*" or “discurs* analysi*” or (field n0 (study or studies or research)) or fieldwork* or “focus group*” or (grounded n0 (theor* or study or studies or research or analys*)) or hermeneutic* or heuristic* or interpretive or “life experience*” or “lived experience*” or narrative or phenomenolog* or qualitative or “thematic analys*” ) or AB ("case stud*" OR Case-stud* or “constant compar*” or “content analysis” or ethnograph* or "discourse* analys*" or “discurs* analysi*” or (field n0 (study or studies or research)) or fieldwork* or “focus group*” or (grounded n0 (theor* or study or studies or research or analys*)) or hermeneutic* or heuristic* or interpretive or “life experience*” or “lived experience*” or narrative or phenomenolog* or qualitative or “thematic analys*” ) OR ( TI (("semi-structured" or semistructured or unstructured or "in-depth" or indepth) n2 (interview* or discussion* or questionnaire*)) or AB (("semi-structured" or semistructured or unstructured or "in-depth" or indepth) n2 (interview* or discussion* or questionnaire*)) ) OR ( (MH "Qualitative Research") OR (MH "Hermeneutics") OR (MH "Grounded Theory") |
