# Supplementary material for: The range and reach of qualitative research in neurosurgery: A scoping review
Source: PLoS One. 2025 Aug 21;20(8):e0330770. doi: 10.1371/journal.pone.0330770 (PMC12370046; doi:10.1371/journal.pone.0330770)
Supplement: S3 Table — Data extraction for papers included in this scoping review. (PDF) [file pone.0330770.s003.pdf]

Author(s)	Specialty (RCS)	Year	Lead author country	Lead author profession	Lead author affiliation	Multi-authorship origin	Journal	Title	Aim(s) or question (s)	Patient demographic: Adult/ Pediatrics/ mixed	Pathology	Country of data collection	Methodology or Research design	Paradigm / theoretical framework	Participants	Total qualitative sample size	Data collection methods	Qualitative data analysis	Analytical theory	Use of reporting guidelines	Results
Bosanac, Hamilton, Lucak, et al.	Functional	2018	Australia	Not reported	Department of Psychiatry	All HIC	BMC Psychiatry	***Identity challenges and 'burden of normality' after DBS for severe OCD: A narrative case study***	to explore the subjective experience of preparing for and undergoing DBS as a treatment for severe and treatment-refractory OCD and the experience of the impact of the treatment.	Adult	Deep Brain Stimulation; OCD; idiopathic adolescent scoliosis.	Australia	Case study	Not reported	Family members, carers, signifcant others	2	Interviews (open, in-depth, semi-structured, open ended)	Narrative analysis	Labov and Waletzky (1967)	COREQ	The parallel stories show how OCD posed severe challenges to identity and social milestones, with profound positive and negative impact on the person and family. Yet symptom remission was accompanied by expanded horizons, but also by uncertainty and intense distress associated with the changed identity.
Coleman, McIntosh and Wilson	Functional	2019	Australia	Not reported	Melbourne School of Psychological Sciences	All HIC	Epilepsia Open	***Identifying the trajectory of social milestones 15-20 years after epilepsy surgery: Realistic timelines for postsurgical expectations***	Short- to medium-term research consistently points toward improvements in social outcomes after epilepsy surgery; however, no study has mapped out postsurgical social timelines, particularly for longer-term (>15Â years) outcomes.	Adult	Epilepsy	Australia	Mixed methods	Not reported	Patients	39	Mixed: Interviews (open, unstructured, in-depth, semi-structured, open ended) AND questionnaires	Thematic: Content analysis	Braun & Clarke (2006)	Not reported	There was a common sequence of social milestone achievement, spanning 20Â years post surgery. Typically, patients first (re)gained their license, then attempted educational and vocational gains, followed by establishing long-term relationships and finally a family unit. Rare, intermittent seizures post surgery did not appear to have detrimental effects on social trajectories. Those who experienced a reduction in seizures showed increased likelihood of attaining social milestones compared to those with ongoing seizures.
Coleman, McIntosh and Wilson	Functional	2020	Australia	Not reported	Melbourne School of Psychological Sciences, The University of Melbourne	All HIC	Epilepsy and Behavior	***A patient-centered approach to understanding long-term psychosocial adjustment and meaning-making, 15 to 20"¯years after epilepsy surgery***	To explore how patients reflected on and made sense of their adjustment trajectories, 15 to 20 years after seizure surgery. This included the impact of surgery on their sense of self and broader psychosocial functioning.	Adult	Epilepsy	Australia	Grounded theory	Not reported	Patients	40	Interviews (open, in-depth, semi-structured, open ended)	Grounded theory analysis/constant comparison/open, axial and/or selective coding	Glaser & Strauss (1967)	Not reported	Patient narratives revealed a common process of psychosocial change and meaning-making triggered by surgery, which was often perceived as a major turning point in life. Patients reflected on moving through an early postsurgical period (< 5 years) of upheaval and psychological disequilibrium. While this period was often remembered as stressful, difficulties were softened and/or reframed in hindsight. Through this process of reframing and meaning-making, patients were able to reestablish equilibrium and a sense of normality. Differences were evident in how patients navigated the process of meaning-making, and the extent to which they felt surgery had changed their self-identity.
Liddle, Phillips, Gustafsson, et al.	Functional	2018	Australia	Occupational therapist	Asia Pacific Center for Neuromodulation	All HIC	Australian Occupational Therapy Journal	***Understanding the lived experiences of Parkinson's disease and deep brain stimulation (DBS) through occupational changes***	To explore the lived experiences of people undergoing DBS for Parkinson's disease	Adult	Parkinsons, DBS	Australia	Phenomenology (Descriptive)	Not reported	Patients AND Family members, carers, signifcant other AND HCPs	35	Interviews (open, unstructured, in-depth, semi-structured, open ended)	Not clearly stated	Patton (2002)	Not reported	Perspectives and experiences of 14 people with PD undergoing DBS, 10 family members and 11 health professionals were Analyzed. Occupations emerged as a key aspect throughout the DBS experience. Two major themes captured the role of occupation in relation to DBS: Occupations as a barometer, where occupational experiences and performances shaped people's understanding of their condition, the impact of treatments and their overall adjustment; and Shifting occupational identity where the life transition of DBS altered the occupational experiences of relationships, volition, roles and responsibilities of people with PD and their family members
Mosley, Robinson, Coyne, et al.	Functional	2021	Australia	Psychiatrist	Systems Neuroscience Group; Neurosciences Queensland, Queensland Brain Institute, Faculty of Medicine	All HIC	Neuroethics	***"Woe Betides Anybody Who Tries to Turn me Down" A Qualitative Analysis of Neuropsychiatric Symptoms Following Subthalamic Deep Brain Stimulation for Parkinson's Disease***	To explore the meaning and significance of stimulation related neuropsychiatric symptoms amongst a purposive sample of persons with PD and their spousal caregivers.	Adult	Parkinsons; Deep brain stimulation (Subthalamic)	Australia	Qualitative	Not reported	Patients AND Family members, carers, signifcant others	20	Interviews (open, unstructured, in-depth, semi-structured, open ended)	Thematic analysis	Braun & Clarke (2006)	COREQ	Caregivers were highly burdened by these symptoms and both patients and caregivers felt unprepared for their consequences, despite having received information prior to DBS, desiring greater family and peer engagement prior to neurosurgery. Participants held conflicting opinions as to whether emergent symptoms were attributable to neurostimulation. Many felt that they reflected aspects of the person's "real" or "younger" personality. Those participants who perceived a close relationship between stimulation changes and changes in mental state were more likely to view these symptoms as inauthentic and uncontrollable. Unexpected and troublesome neuropsychiatric symptoms occurred despite a pre-operative education program that was delivered to all participants. This suggests that such symptoms are difficult to predict and manage even if best practice guidelines are followed by experienced Centers.
Raffaele	Functional	2018	Australia	Not reported	Discipline of Rehabilitation Counselling, Faculty of Health Sciences	N/A	Australian Journal of Rehabilitation Counselling	***A Qualitative Study Exploring Family Life in Men Following Neurosurgery for Adult Onset Epileptic Seizures (AOES).***	What characterizes family relations for men with AOES for which they received surgery?	Adult	Epilepsy	Australia	Case study: Multiple design	Not reported	Patients	5	Interviews (ethnographic)	IPA AND data visualization techniques	Biggerstaff & Thompson, (2008)	Not reported	Themes from IPA: Role marginalization, role dependency and role enmeshment or blurring of social boundaries. Changes in family relationships post elective neurosurgery affected the participants wellbeing., despite self-reported improvements in seizure activity.
Raffaele and Tinofirei	Functional	2020	Australia	Not reported	Rehabilitation counselling, faculty of medicine and health	All HIC	Australian Journal of Rehabilitation Counselling	***Men with adult-onset epilepsy: Their experiences of health care provider relationship qualities following neurosurgery***	What qualities define the health care provider and person with AOES relationships that would be important for sustained recovery and SWB?	Adult	Epilepsy	Australia	IPA AND Multiple case study	Not reported	Patients	5	Interviews (open, in-depth, semi-structured, open ended)	IPA	Colaizzi, (1978); Smith & Osborn, 2015	Not reported	3 clustered themes: timeliness, support and responsibility based on whether participants felt education received from HCWs influenced their SWB. Men reported routine exclusion and impersonal treatment.
Raffaele, Mpofu, Smith-Merry, et al.	Functional	2017	Australia	Not reported	Faculty of health sciences	Mixed HIC/LMIC	Australian Journal of Rehabilitation Counselling	***Men with adult onset epileptic seizures: Their coping strategies and sense of subjective wellbeing following elective neurosurgery***	To understand the coping strategies used by men with AOES following elective neurosurgery, and how these adaptive skills relate to their subjective wellbeing	Adult	Epilepsy	Australia	IPA AND Multiple case study	Not reported	Patients	5	Interviews (open, in-depth, semi-structured, open ended)	Thematic analysis (Line by line)	Luborsky (1994)	Not reported	5 themes: threat minimization, cognitive reconstruction for meanings with AOES survivorship, emotional acceptance and wish fulfilling fantasy (all felt to have largely positive effects on SWB) and self-blame (generated negative sense of wellbeing). men appeared to experience social stigma post elective surgery intended to minimize ongoing seizures. Men experienced significant role marginalization by family and co-workers and poor communication provided by HCW.

Shih, Francis-Auton, Nikpour et al.	Functional	2020	Australia	Social scientist	Australian Center for Health Engagement, Evidence and Values, School of Health & Society; Australian Institute of Health Innovation, Faculty of Medicine and Health Sciences	All HIC	Epilepsy and Behavior	***Enhancing quality of life among epilepsy surgery patients: Interlinking multiple social and relational determinants***	This study focuses on how patients preparing for or have undergone resective brain surgery conceptualize QOL in the context of their condition and treatment, and how clinical and social aspects of their care are harnessed to enhance QOL holistically.	Adult	Epilepsy	Australia	Qualitative	Not reported	Patients AND HCP (consulting clinician)	21	Mixed: Interviews (open, in-depth, semi-structured, open ended) AND Observation of consultations	Thematic analysis	Charmaz & Mitchell (2001); Creswell (2013); Braun & Clarke (2012)	Not reported	(1) A myriad of QOL dimensions are highly interrelated and interdependent with mutual 'spin-off' effects: Uncontrolled seizures impacted beyond physical and cognitive health, disrupting important social identities such as being successful parents, spouses, and career professionals. The desire for good clinical outcomes from surgery was justified against the need to mitigate these social and personal concerns. (2) In postsurgery care, there were complementary effects of clinical interventions and social factors on patients' QOL. Psychosocial well-being was supported by a combination of improved physical health, self-confidence, psychological interventions, and social support from employers and educators who were sensitive to patients' specialized needs. (3) Engaging in education, employment, and government services influenced not only socioeconomic well-being, but also a sense of social inclusion. Advocacy made on behalf of patients by clinicians and family members has helped to better manage patients' eligibility for social services provision.
Shih, Nikpour, Bleasel, et al.	Functional	2018	Australia	Not reported	Australian Institute of Health Innovation, Faculty of Medicine and Health Sciences	All HIC	Epilepsy and Behavior	***Leading up to saying "yes": A qualitative study on the experience of patients with refractory epilepsy regarding presurgical investigation for resective surgery***	To examine the experiences of adult patients with refractory epilepsy leading up to and during presurgical investigation and how their perceptions of resective surgery are shaped.	Adult	Epilepsy	Australia	Qualitative (Intramethod qualitative approach)	Not reported	Patients AND HCPs (epilepsy specialists)	36	Mixed: Interviews (open, unstructured, in-depth, semi-structured, open ended) AND observations	Thematic analysis	Braun & Clarke (2013)	Not reported	Patients reflected on prior experiences of poor seizure control and inadequate antiepileptic drug management and a lack of clarity about their condition before referral to tertiary care. Poor continuity of care and disrupted care transitions affected patients from regional locations. Tertiary referral increased engagement with personalized information about refractory epilepsy, which intensified during presurgical assessments with additional hospital visits and consultations. Experiential information, such as testimonials of other patients, influenced perceptions of surgery and fostered more trust and confidence towards healthcare professionals.
Shirbin, McIntosh and Wilson	Functional	2009	Australia	Not reported	School of behavioral Science	All HIC	Epilepsy and Behavior	***The experience of seizures after epilepsy surgery***	To identify key themes derived from content analysis of 15 in-depth patient interviews about the experience of seizure recurrence.	Adult	Epilepsy	Australia	Phenomenology	Not reported	Patients	15	Interviews (open, unstructured, in-depth, semi-structured, open ended)	Content analysis	Not reported	Not reported	The results showed a prominence of psychological issues over medical concerns. The four most frequently expressed themes were perceived success of surgery, medication, acceptance of seizure recurrence, and personal independence. Despite seizure recurrence, patient sentiments were not universally negative; rather there was heterogeneity of views, with some reporting ambivalence and others a sense of satisfaction with outcome. The findings provide evidence for the importance of cognitive reframing and benefit finding in the context of seizure recurrence.
Thomson, Segrave, Racine, et al.	Functional	2020	Australia	Psychologist	Monash University, Melbourne	All HIC	Qualitative Health Research	***"He's Back so I'm Not Alone": The Impact of Deep Brain Stimulation on Personality, Self, and Relationships in Parkinson's Disease***	To examine the significance and meaning of DBS-related changes in personality and self for patients and caregivers.	Adult	Parkinsons; DBS	Australia	Qualitative	Realist paradigm	Patients AND Family members, carers, signifcant others	22	Interviews (open, unstructured, in-depth, semi-structured, open ended)	Thematic analysis: Realist	Braun & Clarke (2006)	Not reported	We identified three themes present prior to DBS that reflected a time of anticipation, while three themes present after DBS reflected a process of adjustment. Participants noted both positive and negative personality changes, with some, but not all, attributing them to the stimulation. The risk of stimulation-related personality change should be weighed against the procedure's motor benefits and considered in the context of disease- and medication-related personality changes.
Gilbert	Functional	2018	USA	Not reported	Center for Sensorimotor Neural Engineering, Department of Philosophy	N/A	Neuroethics	***Deep Brain Stimulation: Inducing Self-Estrangement***	To explore first-personal accounts of perceived self-change by patients implanted with DBS	Adult	Parkinsons; DBS	Australia	Phenomenology	Not reported	Patients	17	Interviews (open, unstructured, in-depth, semi-structured, open ended)	Phenomenological analysis	Speziale & Carpenter (2007)	Not reported	Patients appear to experience postoperative DBS-induced changes in the form of self-estrangement. Using the insights from patients' subjective perceptions of postoperative self-change provides a potent explanation of potential DBS-induced self-estrangement.
Gilbert, Goddard, Viaña, et al.	Functional	2017	USA	Not reported	University of Tasmania and University of Washington	All HIC	AJOB Neuroscience	***I Miss Being Me: Phenomenological Effects of Deep Brain Stimulation***	To explore perceptions of self-change by patients implanted with DBS	Adult	Parkinsons; DBS	Australia	Phenomenology	Not reported	Patients	17	Interviews (open, unstructured, in-depth, semi-structured, open ended)	Other: grouping patients' self-experience into four main phenomenological clusters of experience	Not reported	Not reported	The study concluded that (1) the more patients preoperatively felt alienated by their illness, the more they experienced postoperative self-estrangement, and (2) the notion of self-estrangement seems to exist in association with certain common qualitative characters, namely, loss of control, which reflects a deteriorative estrangement, and distorted perception of capacities, which reveals a restorative estrangement.
Elliott, Lach and Smith	Functional	2000	Canada	Not reported	Departments of Nursing/Division of Neurology	All HIC	Epilepsy and Behavior	***Adolescent and Maternal Perspectives of Quality of Life and Neuropsychological Status Following Epilepsy Surgery***	To identify the nature and direction of postsurgical changes in each of the quality-of-life domains from the perspective of both adolescents and their mothers.	Adolescents	Epilepsy	Canada	Mixed methods	Not reported	Patients AND Family members, carers, signifcant others	26	Mixed: Interviews (open, unstructured, in-depth, semi-structured, open ended) AND questionnaires	Thematic analysis	Morse & Fields (1995)	Not reported	The results demonstrate that change after epilepsy surgery is multifaceted, and that adolescent and maternal perceptions are notably different. Whereas most adolescents experienced positive changes, mothers simultaneously reported many more negative changes along with positive changes. Furthermore, mothers frequently described aspects of their adolescent's life that continued to be of concern, whereas adolescents did not.
Lunney, Wahby, Sauro, et al.	Functional	2018	Canada	Not reported	Department of Clinical Neurosciences	All HIC	Epileptic disorders: international epilepsy journal with videotape	***Patient satisfaction with epilepsy surgery: what is important to patients?***	To systematically obtain patient-identified factors related to satisfaction with epilepsy surgery as a means of informing clinicians about the ways that patients evaluate outcomes of their treatment and as a conceptual basis for the future development of epilepsy surgery patient satisfaction scales.	Adult	Epilepsy	Canada	Delphi/consensus	Not reported	Patients AND HCPs (inc. NSx)	12	Mixed: Focus groups AND Delphi	Thematic: content analysis	Braun & Clarke (2006)	Not reported	A list of 31 items embodied 12 themes related to patient-reported satisfaction with epilepsy surgery. These included adverse effects, medical care or rehabilitation, seizure control, post-operative recovery, anti-seizure medication, independence, seizure worry, ability to drive, social relationships, self-confidence, improved cognitive function, and improved physical health.
Mendelsohn, Lipsman and Bernstein	Functional	2010	Canada	MD/Physician	Division of Neurosurgery	All HIC	Journal of Neurosurgery	***Neurosurgeons' perspectives on psychosurgery and neuroenhancement: A qualitative study at one center***	To gauge the opinions of neurosurgical staff and trainees toward various uses of neuromodulation technology including psychosurgery and neuroenhancement.	N/A	Deep brain stimulation	Canada	Qualitative	Not reported	NSx	47	Interviews (open, unstructured, in-depth, semi-structured, open ended)	Grounded theory analysis/constant comparison/open, axial and/or selective coding (Thematic)	Strauss and Corbin (1998)	Not reported	Several general themes emerged from the interviews. These included universal support for psychosurgery given adequate informed consent and rigorous scientific methodology, as well as a relative consensus regarding the priority given to patient autonomy and the preservation of personal identity. Participants' attitudes toward the future use of DBS and other means of neuromodulation for cognitive enhancement and personality alteration revealed less agreement, although most participants felt that alteration of nonpathological traits is objectionable

Bell, Maxwell, McAndrews, et al.	Functional	2011	Canada	Not reported	Neuroethics Research Unit	All HIC	World Neurosurgery	***Deep brain stimulation and ethics: Perspectives from a multisite qualitative study of Canadian neurosurgical centers***	To examine health care provider perspectives on ethical and social challenges encountered in DBS.	Unclear	Parkinsons; DBS	Canada	Qualitative	Not reported	HCPs (inc. NSx)	20	Interviews (open, unstructured, in-depth, semi-structured, open ended)	Content analysis	Hsieh & Shannon (2005)	Not reported	Several key ethical issues, such as patient screening and resource allocation, were identified by members of neurosurgical teams. Providers described challenges in selecting patients for DBS on the basis of unclear evidence-based guidance regarding behavioral issues or cognitive criteria. Varied contexts of resource allocation, including some very challenging schemas, were also reported. In addition, the management of patients in the community was highlighted as a source of ethical and clinical complexity, given the need for coordinated long-term care.
Apantaku, Aguiar, Kaal, et al.	Functional	2021	Canada	Researcher	Faculty of Pharmaceutical Sciences & Division of Neurology	All HIC	The Patient: Patient-Centered Outcomes Research	***Understanding attributes that influence physician and caregiver decisions about neurotechnology for pediatric drug-resistant epilepsy: A formative qualitative study to support the development of a discrete choice experiment***	to analyze decision making regarding neurotechnological interventions for pediatric drug-resistant epilepsy from the perspective of physicians and caregivers and the derivation of attributes for a discrete choice experiment.	Pediatric	Epilepsy	Canada and USA	Qualitative (Focus group methodology)	Random utility theory/Lancaster's theory of demand	Family members, carers, signifcant others AND HCPs	55	Focus groups	Thematic analysis (inductive)	Braun & Clarke (2006)	Hollins 2020	The results highlight the presence of central attributes that are considered by both groups in decision making, such as "chances of seizure freedom", "risk" "availability of evidence", and "cost to families", as well as attributes that reflect important differences between groups. Physicians were focused on the specifics of treatment options, while caregivers thought more holistically, considering the overall well-being of their children.
McDonald, Hrincu, Connolly, et al.	Functional	2021	Canada	Not reported	Division of Neurology, Department of Medicine,	All HIC	Journal of Child Neurology	***Novel Neurotechnological Interventions for Pediatric Drug-Resistant Epilepsy: Physician Perspectives***	To investigate factors that guide clinician choices for the adoption of novel neurotechnologies to treat pediatric drug resistant epilepsy.	Pediatric	Epilepsy	Canada and USA	Qualitative	Not reported	HCPs (inc. NSx)	33	Focus groups	Content analysis	Elo & Kyngas (2008), Glasser & Strauss (2017)	SRQR	Discussions revealed two major thematic branches: 1) clinical decision-making; 2) ethical considerations. Under clinical decision-making, physicians emphasized scientific evidence and patient candidacy when assessing neurotechnologies for patients. Ongoing seizures without intervention was important for safety and neurodevelopment. Under ethical considerations, resource allocation, among other financial considerations for technology adoption, were considerable sources of pressure on decision- making. Access to neurotechnology was a salient theme differentiating Canadian and American contexts.
Cabrera, Courchesne, Bittlinger, et al.	Functional	2021	USA	Not reported	Center for Ethics & Humanities in the Life Sciences	All HIC	Culture, Medicine and Psychiatry	***Authentic Self and Last Resort: International Perceptions of Psychiatric Neurosurgery***	(a) To explore awareness of different psychiatric neurosurgery procedures; (b) characterize the understanding of and values related to the different procedures; and, (c) identify key ethical concerns.	Adult	Psychiatric neurosurgery; Deep brain stimulation	Canada, Berlin, Spain	Qualitative	Pragmatic neuroethics	Public/lay	48	Focus groups	Content analysis (Directed)	Hsieh and Shannon (2005), Krippendorff (2004)	Not reported	Participants across all cities hold concerns about the last resort nature of psychiatric neuro-surgery and the potential impact on the authentic self of patients who undergo these procedures. The views captured serve to advance discussion on the appropriate timing for psychiatric neurosurgery, promote sound health policy for the allocation of this resource, and foster scientific literacy about advances for mental health internationally.
Paget, Campbell, Blaxland, et al.	Functional	2021	Australia	Not reported	The Children's Hospital at Westmead; Faculty of Medicine and Health	All HIC	Child: Care, Health and Development	***Life-changing surgery': English-language news media representation of selective dorsal rhizotomy***	To determine how selective dorsal rhizotomy is represented in the English-language news media.	Pediatric	Cerebral palsy & selective dorsal rhizotomy	Canada, New Zealand, Australia, United States, United Kingdom	Qualitative	Not reported	Other	Other: 186 articles describing 91 children	Documentary sources: News media articles from July 2015 - July 2018	Content analysis	Caulfield et al. (2014), Kamenova et al. (2014)	Not reported	One hundred and eighty-six articles were identified describing 91 different children (45 male), almost all with cerebral palsy, median age 4 years old. One hundred and twenty-six articles were written prior to surgery; in many articles, SDR surgery involved travel overseas and/or fundraising. SDR was described universally in positive terms with little discussion of risks. Content of articles variably included the specialized nature of SDR, parental frustration with their local health system and their hope for positive outcomes. There was geographical variation in both numbers of articles and content.
Lewis, Maier, Horstkötter, et al.	Functional	2015	Germany	Not reported	Department of Neurology	All HIC	Journal of Neurology	***The impact of subthalamic deep brain stimulation on caregivers of Parkinson's disease patients: an exploratory study***	To study the caregivers' perception of their own well-being 1 year after subthalamic deep brain stimulation (STN-DBS) surgery in Parkinson's disease (PD) patients, using a qualitative and quantitative approach.	Adult	Parkinsons, subthalamic deep brain stimulation	Germany	Mixed methods (Questionnaires ; Qualitative)	Not reported	Family members, carers, signifcant others	25	Mixed: Interviews (open, unstructured, in-depth, semi-structured, open ended) AND questionnaires	Content analysis	Mayring (2008)	Not reported	At 3-month FU, caregivers were more indecisive concerning their own well-being than at 1-year FU. At 1-year FU, caregivers from the negative group had greater depression, anxiety and lower QoL ratings. They were significantly older compared to the positive group. Patients' depression showed significantly stronger improvement in the positive outcome group. Patients' apathy and depression ratings were significant covariates of caregivers' QoL. Our results show that at 1-year FU over 50 % of the caregivers rated their subjective well-being as negative. Especially older and more depressed caregivers are at risk.
Voigt	Functional	2021	Germany	Not reported	Albert Ludwig University of Freiburg,	All HIC	Neuroethics	***Bodily Felt Freedom: an Ethical Perspective on Positive Aspects of Deep Brain Stimulation***	To emphasize the hitherto neglected positive aspects of deep brain stimulation	Adult	Parkinsons; DBS	Germany	Phenomenology	Anthropology of Plasticity,	Patients	8	Mixed: Interviews (narrative or partly-structured) AND focus groups	Phenomenological analysis	Merleau-Ponty (1965)	Not reported	In the presented analysis, we have seen that patients with PD, who are very restricted in their capability to move and feel unfree because of that, can regain their feeling of freedom through DBS (although not completely). Immediately after the surgery, the feeling of freedom is expressed in euphoria. This feeling is comparable (even it is "artificial") to the feeling that is caused by extreme sporting activity and leads to the feeling of being detached. However, euphoria has a downside: it can lead to compulsive behavior that cause some patients to suffer. Besides the feeling of freedom that is expressed in euphoria and bears the risk of limiting rational autonomy, we have seen that DBS allows a feeling of freedom that is more latent. This moderate version has its roots in movements, which accompany life all along. This effect of DBS is experienced by the patients as positive, because they being with oneself again. DBS rehabilitates the feeling of freedom on a very fundamental level; feelings rooted in the freedom of movement. DBS is a means to re-create the felt body next to a series of feelings of delight.

Gardner, Warren, Addison, et al.	Functional	2019	Australia	Not reported	School of Social Sciences	All HIC	Social Science and Medicine	***Persuasive bodies: Testimonies of deep brain stimulation and Parkinson's on YouTube***	To examine how DBS, Parkinson's disease, and DBS recipients themselves, are delineated within these YouTube videos.	Unclear	Parkinsons; DBS	International	Grounded theory	Not reported	Other	60	Social media content: YouTube videos	Grounded theory analysis/constant comparison/open, axial and/or selective coding	Charmaz (2014)	Not reported	The videos, we demonstrate, contain common compositional and stylistic elements that collectively represent DBS as a technological fix, and which accentuate the autonomy of the DBS recipient. The relational, interpersonal dimensions of chronic illness, and the complex impact of DBS on family dynamics, are elided. We therefore shed light on the means by which high expectations regarding DBS are sustained and circulated, and more generally, we illustrate how potentially powerful representations of medical technologies can emerge from the intersection of social media platforms, afflicted bodies and patient narratives.
Scaratti, Zorzi, Guastafierro, et al.	Functional	2020	Italy	Psychologist (incl. clinical, neuro-)	Neurology, Public Health, Disability Unit, Fondazione IRCCS Istituto Neurologico Carlo Besta	All HIC	European Journal of Pediatric Neurology	***Long term perceptions of illness and self after Deep Brain Stimulation in pediatric dystonia: A narrative research***	The present study aims to explore the experience of illness and the relation with the device in adult patients suffering from dystonia who underwent DBS surgery in pediatric age.	Adult (Pediatric experience)	Deep brain stimulation	Italy	Narrative inquiry	Not reported	Patients	8	Other: Written interview form	Thematic analysis	Guest, MacQueen, & Namey (2012) Lieblich, Tuval-Mashiach, & Ziber (1998)	Not reported	Five main themes emerged: "relationship with the disease", "experience related to DBS procedure", "relationship with one's own body", "fears", "thoughts about future". Despite a general satisfaction in relation to DBS intervention, some patients expressed difficulties, such as the acceptance of changes in one's own body, concerns and fears regarding the device and the future, also considering the critical phase of transition from childhood to adulthood.
de Haan, Rietveld, Stokhof, et al.	Functional	2013	The Netherlands	Not reported	Department of Psychiatry, Academic Medical Center	All HIC	Frontiers in Human Neuroscience	***The phenomenology of deep brain stimulation-induced changes in OCD: An enactive affordance-based model***	To capture the changes in the patients' phenomenology and make sense of the broad range of changes they report after DBS.	Adult	Obsessive Compulsive Disorder; DBS	Netherlands	Grounded theory (loose variant)	Phenomenology and ecological psychology,	Patients	14	Interviews (open, unstructured, in-depth, semi-structured, open ended)	Grounded theory analysis/constant comparison/open, axial and/or selective coding (loose variant)	Strauss and Corbin, (1994)	Not reported	The first aspect is the patients' experience of the world. We propose to specify the patients' world in terms of a field of affordances, with the three dimensions of broadness of scope ("width" of the field), temporal horizon ("depth"), and relevance of the perceived affordances ("height"). The second aspect is the person-side of the interaction, that is, the patients' self-experience, notably their moods and feelings. Thirdly, we point to the different characteristics of the way in which patients relate to the world. And lastly, the existential stance refers to the stance that patients take toward the changes they experience: the second-order evaluative relation to their interactions and themselves.
de Haan, Rietveld, Stokhof, et al.	Functional	2015	The Netherlands	Philosopher	Department of Psychiatry, Academic Medical Center	All HIC	PLoS ONE	***Effects of deep brain stimulation on the lived experience of obsessive-compulsive disorder patients: In-depth interviews with 18 Patients***	To explore and describe the changes in the experiences and life-world of OCD patients following DBS treatment.	Adult	Obsessive Compulsive Disorder; DBS	Netherlands	Grounded theory (roughly follows)	Phenomenology and ecological psychology,	Patients	18	Interviews (open, unstructured, in-depth, semi-structured, open ended)	Grounded theory analysis/constant comparison/open, axial and/or selective coding (roughly follows)	Strauss A, Corbin J (1994)	Not reported	We list the changes grouped in four domains: with regard to (a) person, (b) (social) world, (c) characteristics of person-world interactions, and (d) existential stance.
Nijhuis, Van Heek, Bloem, et al.	Functional	2016	The Netherlands	Not reported	Department of Neurology	All HIC	Journal of Parkinson's Disease	***Choosing an advanced therapy in Parkinson's disease; Is it an evidence-based decision in current practice?***	To explore current decision-making in advanced PD	Adult	Parkinsons; Deep brain stimulation	Netherlands	Qualitative	Not reported	Patients AND Family members, carers, signifcant others AND HCPs	46	Mixed: Focus groups AND semi-structured interviews	Thematic analysis	Braun & Clarke (2006)	COREQ	Four themes representing current experiences with the decision-making process were identified: 1) information and information needs, 2) factors influencing treatment choice and individual decision strategies, 3) decision-making roles, and 4) barriers and facilitators to shared decision-making (SDM). Patient preferences were taken into account, however patients were not always provided with adequate information. The professional's expertise influenced the decision-making process in both positive and negative ways. Although professionals and patients considered SDM essential for the decision of an advanced treatment, they mentioned several barriers for the implementation in current practice.
Ahlberg, Laakso and Hartelius	Functional	2011	Sweden	Not reported	Division of Speech and Language Pathology	All HIC	Parkinson's Disease	***Perceived changes in communication as an effect of STN surgery in Parkinson's disease: A qualitative interview study***	To explore four individuals' perspective of the way their speech and communication changed as a result of subthalamic nucleus deep brain stimulation treatment for Parkinson's disease.	Adult	Parkinsons; DBS (subthalamic nucleus)	Sweden	Qualitative	Not reported	Patients	4	Interviews (open, unstructured, in-depth, semi-structured, open ended)	Content analysis	Graneheim and Lundman (2004); Burnard (1991)	Not reported	The first theme included sub-themes describing both increased and unexpected communication difficulties such as a more vulnerable speech function, re-emerging stuttering and cognitive difficulties affecting communication. The second theme comprised strategies to improve communication, using different speech techniques and communicative support, as well as trying to achieve changes in medical and stimulation parameters. The third theme included descriptions of mixed feelings surrounding the surgery. Participants described the surgery as an unavoidable dramatic change, associated both with improved quality of life but also uncertainty and lack of information, particularly regarding speech and communication changes.
Hariz and Hamberg	Functional	2014	Sweden	Not reported	Occupational Therapy	All HIC	Neuromodulation	***Perceptions of living with a device-based treatment: An account of patients treated with deep brain stimulation for Parkinson's disease***	To explore patients' own perceptions of living with an implanted device.	Adult	Parkinsons; DBS	Sweden	Qualitative	Not reported	Patients	42	Interviews (open, unstructured, in-depth, semi-structured, open ended)	Grounded theory analysis/constant comparison/open, axial and/or selective coding	Corbin & Strauss (2008)	Not reported	The device's existence was immaterial to most patients, however mattered to some where the hardware was more obvious. Less than a third of the patients expressed various concerns and worries about carefulness surrounding the device in daily life. Many patients relied heavily on the DBS nurses and expressed an almost total confidence in them regarding every aspect of the DBS device. Patients more or less expressed difficulties in finding the optimal match between decrease of symptoms and stimulation-induced side-effects.
Hariz, Limousin and Hamberg	Functional	2016	Sweden	Not reported	Occupational Therapy	All HIC	Journal of Parkinson's Disease	***"DBS means everything - For some time" Patients' perspectives on daily life with deep brain stimulation for Parkinson's disease***	To collect and Analyze patients' narratives about their everyday experiences of being on chronic DBS.	Adult	Deep brain stimulation	Sweden	Qualitative	Not reported	Patients	42	Interviews (open, unstructured, in-depth, semi-structured, open ended)	Grounded theory analysis/constant comparison/open and axial/selective coding	Corbin & Strauss (2008)	Not reported	Participants were on chronic DBS at time of interview and had experienced the significance of DBS in daily life. Core categories were revealed, namely DBS means everything for some time, relief from invasive tremor, a rescue from cramps and pain, easier movement swings and more predictable living space, Parkinson's progressing despite DBS. Overall participants found relief in their chronic use of DBS for relief of parkinsonian symptoms and functional restoration. An overarching theme that was consistent was that participants understood the ability to regain previous functioning was isolated in time as Parkinson's progressed.

Ljunggren, Winblad, Hällgren Graneheim, et al.	Functional	2021	Sweden	Neuropsycholo gist	Department of Clinical Neuroscience, Institute of Neuroscience and Physiology	All HIC	Epilepsy and Behavior	***Experiences of emotional and psychosocial functioning after frontal lobe resection for epilepsy***	To explore patients' and relatives' experiences of cognitive, emotional and social cognitive functioning after frontal lobe epilepsy surgery.	Adult	Parkinsons	Sweden	Qualitative	Not reported	Patients AND Family members, carers, signifcant others	26	Interviews (open, unstructured, in-depth, semi-structured, open ended)	Content analysis	Krippendorff (2013) Graneheim & Lundman (2004	Not reported	Positive as well as negative consequences were described both by patients and relatives. Feelings of relief and an increased capacity to experience emotions of well-being were mainly experienced as related to seizure freedom. A newfound autonomy and a more grown-up identity as opposed to a self- image based on epilepsy was also highlighted. However, results also showed that even for seizure free patients, FLR could give rise to negative experiences, the most prominent of which were mental fatigue, lowered mood and social withdrawal. Coping strategies included planning ahead to avoid mental exhaustion. Over all, respondents considered that the epilepsy surgery had been a risk well worth taking and that positive consequences outweighed the negative ones.
Ozanne, Verdinelli, Olsson, et al.	Functional	2016	Sweden	Not reported	Institute of Health and Care Sciences; Department of Clinical Neuroscience and Rehabilitation, Institute of Neuroscience and Physiology	All HIC	Epilepsy and Behavior	***Parental experiences before and long-term after their children's hemispherotomy - A population-based qualitative study***	To explore parents' experiences before and after hemispherotomy as reported at a long-term follow-up and their view on received information and support	Pediatric	Epilepsy	Sweden	Qualitative (descriptive)	Not reported	Family members, carers, signifcant others	24	Interviews (open, unstructured, in-depth, semi-structured, open ended)	Content analysis	Graneheim & Lundman (2004), Krippendorff (2013)	Not reported	Before surgery, the theme 'Living in a chaotic bubble' illuminates how parents felt: the family lived in isolation, they felt both dissatisfaction and satisfaction about support and information, and they experienced that surgery was a question about life or death. After surgery, the theme 'Hovering between success and disaster' illuminates how parents hovered between happiness if the surgery was successful and sadness about e.g., complications and behavior problems. They experienced both excellent and poor support, in hospital and at rehabilitation. Regardless of all concerns, parents were satisfied that the child had received an operation. The hemispherotomies were successful and generated a better life situation. However, in order to cope, families need support and information throughout the whole process, from the onset of epilepsy and for a long time after surgery. If the child has behavior problems, an assessment should be made before surgery in order for the families to get adequate support. The specialist team needs to be involved as early as possible and follow the families for several years after surgery. Focus should be on the whole family, if needed including grandparents; family-centered care might be relevant for this patient group.
Ozanne, Verdinelli, Olsson, et al.	Functional	2018	Sweden	Not reported	Institute of Health and Care Sciences,	All HIC	Epilepsy & behavior : E&B	***Callosotomy in children - Parental experiences reported at long-term follow-up***	To explore parental experiences of the family's life situation before and long after their child had undergone callosotomy	Pediatric	Epilepsy; callosotomy	Sweden	Qualitative	Not reported	Family members, carers, signifcant others	13	Interviews (open, unstructured, in-depth, semi-structured, open ended)	Content analysis	Graneheim & Lundman (2004, 2017)	Not reported	Before surgery, parents felt that they lived in a chaotic bubble with an unbearable situation; their child had severe and frequent seizures and had to be looked after constantly. Most parents were both satisfied and dissatisfied with the given support and information. However, if the child did not improve after surgery, parents often felt that the information before surgery had not been adequate. After surgery, they found a glimpse of hope. They felt that the family got a new life; the reduced seizure severity led to a better life situation for the family. The support was described as both good and poor. The family life situation was complex, and even if they were partly satisfied with the support, it was still not enough. However, the life situation was also very stressful because of remaining seizures, behavioral problems, and sometimes, adverse effects of surgery. The families lived in disappointment and difficulty and had to fight for their rights. This indicates that these families need more information and social service coordination both before and long after surgery. They need not only tools to manage the child's disabilities but also substantial help to care for the child and to receive the social support they need.
Sperens, Hamberg and Hariz	Functional	2017	Sweden	Not reported	Department of Community Medicine and Rehabilitation, Occupational Therapy	All HIC	Parkinson's Disease	***Are Patients Ready for "eARLYSTIM"? Attitudes towards Deep Brain Stimulation among Female and Male Patients with Moderately Advanced Parkinson's Disease***	To explore, in female and male patients with medically treated, moderately advanced Parkinson's disease (PD), their knowledge and reasoning about Deep Brain Stimulation (DBS).	Adult	Parkinsons; DBS	Sweden	Grounded theory	Not reported	Patients	23	Interviews (open, unstructured, in-depth, semi-structured, open ended)	Grounded theory analysis/consta nt comparison/op en and axial/selective coding	Cobin & Strauss (2008); Charmaz (2014)	Not reported	From the patients' narratives, the core category "Processing DBS: balancing symptoms, fears and hopes" was established. The patients were knowledgeable about DBS and expressed cautious and well considered attitudes towards its outcome but did not consider themselves ill enough to undergo DBS. They were aware of its potential side-effects. They considered DBS as the last option when oral medication is no longer sufficient. There was no difference between men and women in their reasoning and attitudes towards DBS. Conclusion. This study suggests that knowledge about the pros and cons of DBS exists among PD patients and that they have a cautious attitude towards DBS. Our patients did not seem to endorse an earlier implementation of DBS, and they considered that it should be the last resort when really needed.
Sylvén, Olsson, Hallböök, et al.	Functional	2020	Sweden	Not reported	Dept. of Pediatrics, Institute of Clinical Sciences; Queen Silvia Children's Hospital	All HIC	Epilepsy and Behavior	***In the best case seizure-free' - Parental hopes and worries before and satisfaction after their child's epilepsy surgery***	The objective of this study was to gain a better understanding of parental hopes and worries before and subsequent experiences two years after their child had undergone epilepsy surgery.	Pediatric	Epilepsy	Sweden	Survey	Not reported	Family members, carers, signifcant others	107	Open ended questions	Thematic analysis (inductive)	Braun & Clarke (2006)	Not reported	Before surgery, parental hopes focus on not only seizure freedom or reduction but also potential improvements in child development and emotional-behavioral functioning. Worries before surgery include not only potential injury or loss of skills but also a concern that the surgical procedure would not lead to an improvement in the child's seizures. The vast majority of parents experienced positive aspects at the two-year follow-up including seizure freedom or reduction but also perceived improvements in behavior, development, and sleep. This suggests that for many, expectations for the surgery were met. A small number of parents reported negative effects of surgery including loss of skills, worsening/lack of improvement in seizure frequency, or negative impact on development.
Hamberg and Hariz	Functional	2014	Sweden	Not reported	Department of Public Health and Clinical Medicine, Family Medicine	All HIC	BMC Neurology	***The decision-making process leading to deep brain stimulation in men and women with Parkinson's disease - an interview study***	To investigate the decision making process to undergo DBS from the patient's perspective, and explored any gender patterns in the participants' decision-making.	Adult	Parkinsons	Sweden and UK	Mixed methods (Grounded theory; Descriptive statistics)	Not reported	Patients	50	Interviews (open, unstructured, in-depth, semi-structured, open ended)	Grounded theory analysis/consta nt comparison/op en, axial and/or selective coding	Corbin & Strauss (2008)	Not reported	Three different approaches to DBS were identified among the patients. 'Taking own initiative', included 48% of the patients and implied that the patients' own initiatives and arguments had been crucial for having surgery. 'Agreeing when offered', and accepting DBS when suggested by doctors embraced 43%. The third approach, 'Hesitating and waiting' included < 10% of the patients. Most of the men were either 'taking own initiative' or 'agreeing when offered'. The 11 women were evenly distributed in all three approaches. Among the interviewed, more women than men expressed strong fear of complications and more women consulted friends and relatives prior to deciding about DBS. Half of the patients had held a leadership position at work or in another Organization, and among patients 'taking own initiative' the proportion with leadership experiences was 80%. At time for surgery ten men but no woman were professionally active.

Hariz, Limousin, Tisch, et al.	Functional	2011	Sweden	Not reported	Occupational Therapy	All HIC	Movement disorders	***Patients' perceptions of life shift after deep brain stimulation for primary dystonia--A qualitative study***	To explore perceptions of changes in life in patients with primary dystonia after deep brain stimulation.	Adult	Primary dystonia; DBS	Sweden and UK	Qualitative	Not reported	Patients	13	Interviews (open, unstructured, in-depth, semi-structured, open ended)	Grounded theory analysis/constant comparison/open and axial/selective coding	Bryant & Charmaz (2007)	Not reported	Four categories were revealed after coding: (1) struggling with a disabling and disfiguring disorder, (2) searching for a turning point, (3) a taste of one's true potential, and (4) transition with guidance. Emphasis is placed by the participants, on the "life changing" nature of the condition and intervention. Focus was placed on the physical changes undergone post surgery, posture changes, new movements their bodies never could perform.
Gámez, Brugger and Biller-Andorno	Functional	2021	Switzerland	PhD student (specialty not reported)	Institute of Medical Bioethics and History of Medicine	All HIC	International Journal of Environmental Research and Public Health	***Parkinson's disease and deep brain stimulation have an impact on my life: A multimodal study on the experiences of patients and family caregivers***	To better understand how patients and family caregivers experience life with PD and DBS, the impact of both on their personal and social lives, and their perception of the changes that have occurred as a result of the disease and the treatment	Adult	Parkinsons; DBS	Switzerland	Qualitative	Not reported	Patients AND Family members, carers, signifcant others	36	Mixed: Interviews (open, unstructured, in-depth, semi-structured, open ended) and drawings	Thematic analysis	Fereday and Muir-Cochrane (2006)	Not reported	Seven principal themes have been identified: "everyone's Parkinson's is different", "changing as a person during the disease", "going through Parkinson's together", "DBS improved my life", "I am treated with DBS but I have Parkinson's still", "DBS is not perfect", and "being different after DBS". PD is perceived as an unpredictable and heterogeneous disease that changes from person to person, as does the effect of DBS. While DBS side-effects may have an impact on patients' personality, behavior, and self-perception, PD symptoms and drug side-effects also have a great impact on these aspects.
İbrahimoğlu, Mersin and Akyol	Functional	2020	Turkey	Not reported	Nursing Department, Faculty of Health Sciences	All LMIC	Acta medica academica	***The Experiences of Patients with Deep Brain Stimulation in Parkinson's Disease: Challenges, Expectations, and Accomplishments***	To determine the challenges, expectations and accomplishments of patients with DBS in Parkinson's disease.	Adult	Parkinsons; DBS	Turkey	Qualitative	Not reported	Patients	7	Focus groups	Content analysis	Not reported	Not reported	Among the participants, six patients were male, and one patient was female. The mean age of the patients was 56.85±16.48. Three main themes were revealed in the study. These were (1) Reborn; decrease in dependence, sense of accomplishment, enjoyment of life, (2) Prejudice; perceived as severely ill by others and (3) Fear; not being accustomed to the device, loss of device function.
Allsop, Twiddy, Grant, et al.	Functional	2015	UK	Not reported	Leeds Institute of Health Sciences	All HIC	Acta Neurochirurgica	***Diagnosis, medication, and surgical management for patients with trigeminal neuralgia: a qualitative study***	Capturing subjective experiences can be used to inform the impact of the condition on quality of life and may contribute to a better understanding of current clinical practice with the aim of improving patient care.	Adult	Nerve	UK	Qualitative	Essentialist framework	Patients	16	Focus groups	Thematic analysis	Braun & Clarke (2006)	Not reported	The impact of TN and treatment on the lives of participants emerged as four predominant themes: (1) diagnosis and support with TN, (2) living in fear of TN pain, (3) isolation and social withdrawal, and (4) medication burden and looking for a cure.
Austin, Lin, Selway, et al.	Functional	2017	UK	Not reported	Department of Clinical Psychology	All HIC	European Journal of Pediatric Neurology	***What parents think and feel about deep brain stimulation in Pediatric secondary dystonia including cerebral palsy: A qualitative study of parental decision-making***	To explore parental decision-making for deep brain stimulation in Pediatric secondary dystonia.	Pediatric	Deep brain stimulation in secondary dystonia	UK	Qualitative	Not reported	Family members, carers, signifcant others	8	Interviews (open, unstructured, in-depth, semi-structured, open ended)	IPA	Smith, Flowers & Larkin, (2009)	Not reported	For all parents the decision was viewed as significant, with life altering consequences for the child. These results suggested that parents were motivated by a hope for a better life and parental duty. This was weighed against consideration of risks, what the child had to lose, and uncertainty of DBS outcome. Decisions were also influenced by the perspectives of their child and professionals.
Heath, Abdin, Begum, et al.	Functional	2016	UK	Not reported	Department of Psychology	All HIC	Epilepsy and Behavior	***Putting children forward for epilepsy surgery: A qualitative study of UK parents' and health professionals' decision-making experiences***	To explore how parents and health professionals make decisions regarding putting children forward for pediatric epilepsy surgery.	Pediatric	Epilepsy	UK	Qualitative	Not reported	Family members, carers, signifcant others AND HCPs (inc. NSx)	19	Interviews (open, unstructured, in-depth, semi-structured, open ended)	Thematic analysis (inductive)	Braun & Clarke (2006)	Not reported	PARENTS: Presentation of surgery as a treatment option was seen as novel and unexpected however providing hope. Decision-making was difficult in most scenarios. Balancing potential costs and benefits concerning the child's future life was a significant factor. In search of information and family decisions parents felt they were given inadequate information on treatment options. Parents typically did not make decisions in isolation but rather involved the wider family unit. Many parents considered surgery as a last report option. HEALTH PROFESSIONALS: Triangulating information involved use of slideshows at MDT meetings, sound communication by this method facilitated better continuity of treatment for the MDT. Cohesive team work proved to be an important theme. Patient and family perspectives were thoroughly considered when a =n MDT decision was taken.
Nelson, Gray and Woolfall	Functional	2021	UK	Psychologist (incl. clinical, neuro-)	Department of Clinical Psychology	All HIC	Seizure	***Parents' experiences of their child's assessment on an epilepsy surgery pathway: A qualitative study***	To explore parents' experiences of their child consideration for epilepsy surgery to inform future service development and delivery.	Pediatric	Epilepsy	UK	Phenomenology	Not reported	Family members, carers, signifcant others	15	Interviews (open, unstructured, in-depth, semi-structured, open ended)	Reflexive thematic analysis	Braun & Clarke (2006, 2019)	COREQ	Initial discussions of epilepsy surgery were described as 'shocking' but also as a source of hope. However, unclear communication between staff and parents, including lack of information about the steps, assessments/investigations and timeframes involved in the process of assessment for surgery led to some feeling 'out of control,' uncertain and in some cases distressed. Parents described examples of positive support from staff, yet many felt they needed additional general and emotional support throughout the epilepsy surgery pathway.
O'Brien, Gray and Woolfall	Functional	2020	UK	Psychologist (incl. clinical, neuro-)	Department of Clinical Psychology	All HIC	Seizure	***Child and parent experiences of childhood epilepsy surgery and adjustment to life following surgery: A qualitative study***	To explore children's and parents' experiences of epilepsy surgery and associated psychosocial outcomes.	Pediatric	Epilepsy	UK	Qualitative	Not reported	Patients AND Family members, carers, signifcant others	16	Interviews (open, unstructured, in-depth, semi-structured, open ended)	Grounded theory analysis/constant comparison/open, axial and/or selective coding	Glaser (1965), Braun and Clarke (2006, 2013)	Not reported	Epilepsy had significant impact on quality of life for children and parents. Concerns about long term wellbeing, risks to safety and hope for a 'normal' life contributed to the decision to pursue surgery. All participants described improvements in seizure control, alongside improved psychological wellbeing, quality of life, social relationships and family functioning. However, children and their parents experienced difficulties adjusting to a new post-surgical identity and 'missing' aspects of their pre-surgical life with epilepsy.
Poole, Mercadante, Singhota, et al.	Functional	2022	UK	Not reported	Eastman Dental Hospital	All HIC	British Journal of Pain	***Exploring patient satisfaction of a joint-consultation clinic for trigeminal neuralgia: Enabling improved decision-making***	To understand patients' level of satisfaction with the joint-consultation clinic and evaluate utilization of a clinical decision-making tool.	Adult	Trigeminal neuralgia (TGN)	UK	Mixed methods (survey)	Not reported	Patients	41	Open ended questions	Thematic analysis	Creswell (2012), Braun & Clarke (2006)	Not reported	Forty-one patients (77% response rate) participated in the survey, and the results were overwhelmingly positive for the joint-consultation clinic regarding satisfaction. The benefits were broad ranging including increased understanding, collaboration and confidence in decision-making.
Shahmoon, Smith and Jahanshahi	Functional	2019	UK	Not reported	UCL Institute of Neurology; Department of Motor Neuroscience and Movement Disorders	Mixed HIC/LMIC	Parkinson's Disease	***The Lived Experiences of Deep Brain Stimulation in Parkinson's Disease: An Interpretative Phenomenological Analysis***	To explore how 10 male people with PD experienced life after STN-DBS surgery	Adult	Parkinsons; Deep brain stimulation (subthalmic nucleus)	UK	IPA	Not reported	Patients	10	Interviews (open, unstructured, in-depth, semi-structured, open ended)	IPA	Smith, Flowers & Larkin (2009)	Not reported	Two themes emerged. The first, "Healed and relieved: all that glitters is not gold," highlights the benefits and the personal "costs" of surgery. The second, "The change within: new interpretations of the present and future unfold," explores how patients reinterpreted their lives as individuals and members of society in the present and as they face their future. Relief, gratitude, disappointment, and the need for social support are expressed as well as a new appraisal of values and the future. STN-DBS alters the life course of people with PD, and this study provides new insight into psychological and social issues that surgery raises for the patient and their family system. These psychosocial issues should be taken into account when preparing the patient and their family for surgery or supporting them postoperatively.

Swift	Functional	2012	UK	Not reported	Center for Ethics in Medicine	N/A	Journal of Empirical Research on Human Research Ethics	***Sham surgery trial controls: Perspectives of patients and their relatives***	To explore attitudes toward sham surgery and reasoning about hypothetical participation in a sham-controlled trial	Adult	Parkinsons	UK	Qualitative	Naturalistic paradigm	Patients AND Family members, carers, signifcant others		20	Interviews (open, unstructured, in-depth, semi-structured, open ended)	Framework analysis	Ritchie & Spencer, (1994)	Not reported	Results showed that attitudes toward sham surgery may not necessarily predict trial participation behavior. A small majority of interviewees deemed sham surgery ethically acceptable with certain provisos, but hypothetical participation was driven primarily by disease severity and a lack of standard treatment options, with a preference for receiving the real surgery over sham.
Haahr, Kirkevold, Hall, et al.	Functional	2010	Denmark	Not reported	Department of Nursing Science, School of Public Health	All HIC	International Journal of Nursing Studies	***From miracle to reconciliation: A hermeneutic phenomenological study exploring the experience of living with Parkinson's disease following Deep Brain Stimulation***	To explore and describe the experience of living with Parkinson's disease when treated with Deep Brain Stimulation.	Adult	Parkinsons; DBS	Unclear	Phenomenology (Hermeneutic)	Hermeneutical phenomenological approach	Patients		9	Interviews (open, unstructured, in-depth, semi-structured, open ended)	Phenomenological analysis	van Manen (1990, 2006)	Not reported	The treatment had a major impact on the body. Participants experienced great bodily changes and went through a process of adjustment in three phases during the first year of treatment with Deep Brain Stimulation. These stages were; being liberated: a kind of miracle, changes as a challenge: decline or opportunity and reconciliation: re-defining life with Parkinson's disease. The course of the process was unique for each participant, but dominant was that difficulties during the adjustment of stimulation and medication did affect the re-defining process
Haahr, Kirkevold, Hall, et al.	Functional	2013	Denmark	RN/Nurse	Department of Nursing Science, School of Public Health	All HIC	Journal of Advanced Nursing	***'Being in it together': Living with a partner receiving deep brain stimulation for advanced Parkinson's disease - a hermeneutic phenomenological study***	To explore the lived experience of being a spouse to a person living with advanced Parkinson's disease, before and during the first year of deep brain stimulation.	Adult	Parkinsons; DBS	Unclear	Phenomenology (Hermeneutic)	Hermeneutical phenomenological approach	Family members, carers, signifcant others		10	Interviews (open, unstructured, in-depth, semi-structured, open ended)	Thematic analysis	van Manen (1990, 2006)	Not reported	The uniting theme 'Solidarity - the base for joined responsibility and concern' was the foundation for the relationship between spouses and their partners. Before treatment, the theme 'Living in partnership' was dominant. After treatment two dichotomous courses were described 'A sense of freedom embracing life' and 'The challenge of changes and constraint'.
Ziesen, Kilian and Schlaepfer	Functional	2020	Germany	Not reported	Chair of Sociology of Technology and Organization (STO) at RWTH Aachen University	All HIC	Personalized Medicine in Psychiatry	***Beyond antidepressant effects of deep brain stimulation - A systematic qualitative approach***	The decisive goal is the patients' view of their own body and the implanted technique, which is addressed by means of a qualitative approach.	Unclear	DBS; psychiatric disorders	Unclear	Grounded theory	Not reported	Patients AND Researchers		24	Mixed: Interviews (open, unstructured, in-depth, semi-structured, open ended) AND observations	Grounded theory analysis/constant comparison/open, axial and/or selective coding	Strauss & Corbin (1990)	Not reported	Two central concerns of the patients became visible: On the one hand, the striving for normality with the focus on the stimulation settings. On the other hand, the desire for self-regulation by means of technology in the event of a deterioration in mood. In addition, almost all patients report that they do not feel the technique and do not feel externally determined by the technique.
Afifi, Schwarze, Stilp et. al.	Functional	2019	USA / Egypt	MD/Physician	Division of Plastic Surgery	Mixed HIC/LMIC	Plastic and Reconstructive Surgery	***"Like a Normal Person Again": A Qualitative Analysis of the Impact of Headache Surgery***	(1) explore patient perceptions of migraines and the changes brought on by surgery, and (2) assess how these changes affected patients' ability to function in daily life.	Adult	Headache	Unclear	Grounded theory	The study was designed using a theoretical framework but not clearly stated which.	Patients		15	Interviews (open, unstructured, in-depth, semi-structured, open ended)	Grounded theory analysis/constant comparison/open and axial/selective coding	Miles and Huberman (1994); Charmaz (2006);	COREQ	Participants reported improvements in one or more domains of pain following surgery, and changes in medication use and effectiveness. Even in individuals with persistent pain postoperatively, surgery appeared to facilitate an improvement in headache self-efficacy, including an ability to participate in daily activities. Migraineurs frequently described a new degree of control over at least one aspect of their pain.
Ahrens, Ostendorf, Lado, et al.	Functional	2022	USA	MD/Physician	Nationwide Children's Hospital, Columbus, OH	All HIC	Neurology	***Impact of the COVID-19 Pandemic on Epilepsy Center Practice in the United States***	to describe data trends in 2020 relative to 2019 in the setting of practice changes due, in large part, to the COVID-19 pandemic.	Unclear	Epilepsy	USA	Survey	Not reported	Other	Other: 187 annual report data		Documentray sources: Annual report data free text comments	Thematic analysis	Not reported	Not reported	Three overarching themes emerged: patient-, system-, and staff-related challenges
Baca, Pieters, Iwaki, et al.	Functional	2015	USA	Associate Professor of Neurology	Department of Neurology	All HIC	Epilepsia	***"a journey around the world": Parent narratives of the journey to pediatric resective epilepsy surgery and beyond***	To identify the nature and range of parent-perceived barriers to timely receipt of pediatric epilepsy surgery.	Pediatric	Epilepsy	USA	Qualitative	Not reported	Family members, carers, signifcant others		37	Interviews (open, unstructured, in-depth, semi-structured, open ended)	Thematic analysis AND diagramming	Braun & Clarke (2006)	Not reported	Thematic analysis revealed four themes (with subthemes) along the journey to surgery and beyond: (1) recognition—"something is wrong" (unfamiliarity with epilepsy, identification of medical emergency); (2) searching and finding—"a circuitous journey" (information seeking, finding the right doctors, multiple medications, insurance obstacles, parental stress); (3) surgery is a viable option—"the right spot" (surgery as last resort, surgery as best option, hoping for candidacy); and (4) life now—"we took the steps we needed to" (a new life, giving back).
Barrios-Anderson, McLaughlin, Patrick, et al.	Functional	2022	USA	Not reported	Warren Alpert Medical School/Department of Neurosurgery/Psychiatric Neurosurgery Program	All HIC	Frontiers in Integrative Neuroscience	***The Patient Lived-Experience of Ventral Capsulotomy for Obsessive-Compulsive Disorder: An Interpretive Phenomenological Analysis of Neuroablative Psychiatric Neurosurgery***	To examine the lived experiences of patients who have undergone Ventral Capsulotomy for severe, treatment-resistant Obsessive Compulsive Disorder.	Adult	OCD; ventral capsulotomy	USA	IPA	Phenomenological, hermeneutic, and idiographic theory	Patients		6	Interviews (open, unstructured, in-depth, semi-structured, open ended)	IPA	Smith et al (2009)	Not reported	Transcript analysis revealed four superordinate themes: (1) the lived experience of treatment-resistant OCD, (2) the pre-surgical conceptualization of the operation, (3) the experience of the surgery, and (4) the post-surgical experience.
Cabrera, Kelly-Blake and Sidiropoulos	Functional	2020	USA	Not reported	Center of Ethics and Humanities in the Life Sciences	All HIC	Brain Sciences	***Perspectives on deep brain stimulation and its earlier use for Parkinson's disease: A qualitative study of US patients***	To explore preferences about the timing of DBS, we asked PD patients with DBS whether they would have preferred the implantation procedure to have occurred earlier after diagnosis.	Adult	Parkinsons; DBS	USA	Qualitative	Not reported	Patients		20	Other: Structured interview with closed and open questions	Content analysis	Braun & Clarke (2006), Vaismoradi, Turunen, & Bondas (2013), Elo & Kyngäs, (2008)	Not reported	We found that the majority of our participants (72%) had high overall satisfaction with DBS in addressing motor symptoms (mean of 7.5/10) and quality of life (mean of 8.25/10). Participants were mixed about whether they would have undergone DBS earlier than they did, with five participants being unsure and the remaining nearly equally divided between yes and no.
Cabrera, Sarva and Sidiropoulos	Functional	2019	USA	Not reported	Center of Ethics and Humanities in the Life Sciences	All HIC	World Neurosurgery	***Perspectives on the Earlier Use of Deep Brain Stimulation for Parkinson Disease from a Qualitative Study of U.S. Clinicians***	To explore clinicians' attitudes toward earlier use of DBS.	Adult	Parkinsons; DBS	USA	Qualitative	Not reported	HCPs (inc. NSx)		12	Other: Structured interview with closed and open questions	Content analysis	Braun & Clarke (2006), Mack et al, (2005); Vaismoradi, Turunen, & Bondas (2013)	Not reported	We found that most clinicians considered earlier use not solely to be time dependent but instead determined by patient symptoms. Only 16.8% were aware of the FDA's recent indication of early use of DBS, with 25% of our respondents being unsure as to whether it should be seen as an early treatment modality. On average, neurologists suggested DBS as the next treatment option, after medications have been exhausted, typically 6 years after diagnosis.

Choi, Pargeon, Bausell, et al.	Functional	2011	USA	Epileptologist	Department of neurology	All HIC	Epilepsy and Behavior	***Temporal lobe epilepsy surgery: What do patients want to know?***	To describe the information patients need to make informed decisions about TLE surgery.	Adult	Epilepsy	USA	Qualitative	Not reported	Patients	20	Focus groups	Thematic analysis	Vaughn et al. (1996)	Not reported	Twenty patients who had undergone TLE surgery described the information used in their decision-making process, and evaluated the potential for a patient decision aid to assist other patients who are considering surgery. Thematic analysis revealed information needs that were both experiential (i.e., learning about other patients' experiences through testimonials) and factual (i.e., individualized statistical information). Patients also made suggestions on how this information should be delivered to patients.
Dewar, Pieters and Fried	Functional	2021	USA	RN/Nurse	Department of Neurology, David Geffen School of Medicine	All HIC	Frontiers in Neurology	***Surgical Decision-Making for Temporal Lobe Epilepsy: Patient Experiences of the Informed Consent Process***	To explore how subjective perceptions of illness severity were described by a sample of participants with drug-resistant epilepsy (DRE) who were considering surgery.	Adult	Epilepsy	USA	Grounded theory	Constructivist	Patients	35	Interviews (open, unstructured, in-depth, semi-structured, open ended)	Grounded theory analysis/constant comparison/open, axial and/or selective coding	Charmaz (2023)	Not reported	A grounded theory with four interrelated categories was developed to reflect the process by which participants arrived at an explanation of illness severity. Illness severity for participants evolved as participants reflected upon the burdensome impact of uncontrolled seizures on self and others. Epilepsy, when compared with other chronic conditions, was described as less serious, and participants imagined that other peoples' seizures were comparatively worse than their own. Illness severity was not uppermost in participants' minds but emerged as a concept that was both relative and linked to social burden. Perceptions of overall disease severity expanded upon determinants of seizure severity to offer a more complete explanation of what patients themselves did about longstanding, uncontrolled epilepsy.
Israel, Gutierrez-Meza, Albano, et al.	Functional	2021	USA	MD/Physician	School of Medicine and Public Health	Mixed HIC/LMIC	Annals of Plastic Surgery	***Qualitative Analysis of Single-Site Headache Surgery: Is It Different from Multiple-Site Surgery?***	To investigate patient perceptions following single-site surgery and compare themes in patients undergoing single-site surgery with those from a previously published conceptual framework generated with patients undergoing multisite surgery.	Adult	Nerve; Migraine surgery	USA	Qualitative	Not reported	Patients	14	Interviews (open, unstructured, in-depth, semi-structured, open ended)	Content analysis	Hsieh & Shannon (2005)	Not reported	Similar recurring themes emerged from the single-site cohort, and the conceptual framework was applicable to all participants. Two new themes emerged from the single-site analysis. First, 5 of 14 participants described being "migraine- free" postoperatively, a finding not observed in the multisite group. Second, several individuals described financial benefits after surgery, via decreased prescription medication requirements, raises at work, and improved productivity.
Klein, Goering, Gagne, et al.	Functional	2016	USA	Not reported	Center for Sensorimotor Neural Engineering and Department of Philosophy	All HIC	Brain-Computer Interfaces	***Brain-computer interface-based control of closed-loop brain stimulation: attitudes and ethical considerations***	We interviewed individuals for their perspectives about closed-loop or next-generation DBS devices, given their experience with open-loop DBS.	Adult	Deep brain stimulation for MDD or OCD	USA	Qualitative	Not reported	Patients	15	Mixed: Focus groups AND semi-structured interviews	Content analysis	Hsieh & Shannon (2005), Kondracki et al. (2002), Miles & Huberman (1984), Morse & Field (1995)	Not reported	We identified four major themes: control over device function, authentic self, relationship effects, and meaningful consent. Each has implications for the design of closed-loop systems for non-motor disorders.
Mancuso, Lee, Dy et al.	Functional	2015	USA	Not reported	Department of Medicine	All HIC	Hand	***Expectations and limitations due to brachial plexus injury: a qualitative study***	To ascertain expectations of surgery for patients with BPI (Brachial plexus injury) and their experiences with BPI, particularly the impact of BPI on quality of life and functional status.	Adult	Nerve, brachial plexus injury	USA	Grounded theory	Not reported	Patients	23	Interviews (open, unstructured, in-depth, semi-structured, open ended)	Grounded theory analysis/constant comparison/open, axial and/or selective coding	Strauss and Corbin (1998), Berkowitz and Inui (1998)	Not reported	Ten preoperative and 13 postoperative patients were interviewed; mean age was 37 years, 19 were men, all were employed/students, and most injuries were due to trauma. Preoperative patients cited several main expectations, including pain-related issues, and improvement in arm movement, self-care, family interactions, and global life function. Work-related expectations were tailored to employment type. Preoperative and postoperative patients reported that pain, altered sensation, difficulty managing self-care, becoming physically and financially dependent, and disability in work/school were major issues. All patients reported making major compensations, particularly using the uninjured arm. Most reported multiple mental health effects, were distressed with long recovery times, were self-conscious about appearance, and avoided public situations. Additional stresses were finding and paying for BPI surgery. Some reported BPI impacted overall physical health, life priorities, and decision-making processes. Four postoperative patients reported hardly any improvement, four reported some/a good deal, and five reported a great deal of improvement.
Mergenthaler, Chiong, Dohan, et al.	Functional	2021	USA	Not reported	Department of Neurology	All HIC	AJOB Neuroscience	***A Qualitative Analysis of Ethical Perspectives on Recruitment and Consent for Human Intracranial Electrophysiology Studies***	To collect the qualitative lived experiences of researchers that expand beyond study protocols and procedures as described in an institutional review board application.	Unclear	Intracranial electrophysiology research	USA	Qualitative	Not reported	Other: Researchers (inc. NSx)	26	Interviews (open, unstructured, in-depth, semi-structured, open ended)	Grounded theory analysis/constant comparison/open, axial and/or selective coding (modified)	Creswell (2007); Lingard, Albert & Levenson (2008)	Not reported	Respondents described significant heterogeneity in recruitment and consent procedures, even among studies employing similar techniques. In some studies, clinician-investigators were specifically barred from obtaining consent, while in other studies clinician-investigators were specifically required to obtain consent; regulatory guidance was inconsistent. Respondents also described various models for subject selection, the timing of consent, and continuing consent for temporally extended studies. Respondents expressed ethical concerns about participants' vulnerability and the communication of research-related risks. We found a lack of consensus among investigators regarding recruitment and consent methods in human intracranial electrophysiology. This likely reflects the novelty and complexity of such studies and indicates a need for further discussion and development of best practices in this research domain.
Merner, Frazier, Ford, et al.	Functional	2021	USA	Not reported	Department of Psychological Sciences; Department of Neurology	All HIC	Frontiers in Human Neuroscience	***Changes in Patients' Desired Control of Their Deep Brain Stimulation and Subjective Global Control Over the Course of Deep Brain Stimulation***	To examine changes in patients' desired control of the deep brain stimulator (DBS) and perception of global life control throughout DBS	Adult	Parkinsons; deep brain stimulation	USA	Mixed methods	Not reported	Patients	52	Mixed: Interviews (open, unstructured, in-depth, semi-structured, open ended) AND questionnaires	Content analysis	Elo & Kyngäs (2008)	Not reported	Participants reported significant increases in their perception of global control over time and significant declines in their desired control of the stimulation. These changes were unrelated to improvements in motor symptoms. Improvements in global control were negatively correlated with a decline in desired stimulation control. Qualitative data indicate that participants have changed, nuanced levels of desired control over their stimulators. Increased global life control following DBS may be attributed to increased control over PD symptoms, increased ability to engage in valued activities, and increased overall self-regulation, while other domains related to global control remained unaffected by DBS.
Pieters, Dewar, Ranit, et al.	Functional	2020	USA	Not reported	School of Nursing	All HIC	Chronic Illness	***Surgical decision-making among patients with uncontrolled epilepsy: "Making important decisions about my brain, which I happen to love"***	To explore decision-making from patients' perceptions of risks and benefits of epilepsy surgery for refractory focal seizures.	Adult	Epilepsy	USA	Grounded theory	Constructivism / symbolic interactionism	Patients	35	Interviews (open, unstructured, in-depth, semi-structured, open ended)	Grounded theory analysis/constant comparison/open, axial and/or selective coding	Charmaz (2014)	Not reported	For this sample of participants decision-making about surgery was complex, centering on the meaning of illness for the self and the impact of epilepsy and its treatment for significant others. Two interrelated categories crystalized from our data: the unique context of brain surgery and how the decisional counterweights of risks and benefits were considered.

Pieters, Iwaki, Vickrey, et al.	Functional	2016	USA	Not reported	School of Nursing	All HIC	Epilepsy and Behavior	***"It was five years of hell": Parental experiences of navigating and processing the slow and arduous time to pediatric resective epilepsy surgery***	To describe experiences during the arduous time before the referral and the parent-reported facilitators that helped them move forward through this slow time.	Pediatric	Epilepsy	USA	Qualitative	Not reported	Family members, carers, signifcant others	37	Interviews (open, unstructured, in-depth, semi-structured, open ended)	Thematic analysis	Hsiesh and Shannon (2005), Vaismoradi, Turunen and Bondas (2013)	Not reported	Parents, 41.3years of age on average, were mostly Caucasian, English-speaking, mothers, married, and employed. The mean age at surgery for children was 8.2years with a mean time from epilepsy onset to surgery of 5.4years. Parental decision-making was facilitated when parents eventually received a presurgical referral and navigated to a multidisciplinary team that they trusted to care for their child with medically refractory epilepsy. Four themes described the experiences that parents used to feel a sense of moving forward. The first theme, processing, involved working through feelings and was mostly done alone. The second theme, navigating the complex unknowns of the health-care system, was more active and purposeful. Processing co-occurred with navigating in a fluid intersection, the third theme, which was evidenced by deliberate actions. The fourth theme, facilitators, explained helpful ways of processing and navigating; parents utilized these mechanisms to turn vulnerable times following the distress of their child's diagnosis into an experience of productivity.
Samanta, Ostendorf, Singh, et al.	Functional	2022	USA	Not reported	Neurology Division, Department of Pediatrics	All HIC	Journal of Child Neurology	***Physicians' Perspectives on Presurgical Discussion and Shared Decision-Making in Pediatric Epilepsy Surgery***	To qualitatively explore the approach of pediatric epilepsy providers when counseling regarding surgical options for epilepsy, presenting risks and benefits of surgery, overcoming resistance to surgery, and fostering shared decision making with patients and families.	Pediatric	Epilepsy	USA	Qualitative	Not reported	HCPs (inc. NSx)	11	Interviews (open, unstructured, in-depth, semi-structured, open ended)	Content analysis: Inductive, thematic	Azungah (2018)	Not reported	A blended inductive-deductive analysis revealed three key themes (with subthemes) of presurgical discussions: (1) Candidate selection and initial discussion about epilepsy surgery (neurologists compared to epileptologists, the timing of the discussion, reluctant families) (2) Detailed individualized counseling about epilepsy surgery (shared decision-making [enablers and barriers] and risk-benefit analysis [balancing risks and benefits, statistical benefit estimation, discussion about SUDEP, prognostication about cognitive and behavioral outcomes, risks of surgery]) (3) Tools to improve decision-making (educational interventions for patients and families and provider- and organization-specific interventions).
Seaburn and Erba	Functional	2003	USA	Not reported	School of Medicine and Dentistry	All HIC	Family Process	***The family experience of "sudden health": The case of intractable epilepsy***	To report the experience of "sudden health" among six families who participated in an exploratory qualitative study of families with a member who elects to have corrective surgery for intractable epilepsy.	Adult	Epilepsy	USA	Case study: Phenomenology AND Grounded Theory	Not reported	Patients AND Family members, carers, signifcant others	29	Mixed: Interviews (open, unstructured, in-depth, semi-structured, open ended) AND meetings with neurologists/ neuropsychiatrist AND medical file review	Grounded theory analysis/constant comparison/open and axial/selective coding	Lincoln and Guba (1985)	Not reported	Findings indicated that (1) families were organized in two primary ways (nesting and crisis) to deal with epilepsy and the aftermath of surgery and (2) "sudden health" had differing effects on these families depending on their organizational style, emotional communication process, and developmental dynamics.
Watson, Afra, Bartolini, et al.	Functional	2021	USA	Not reported	LivaNova, Neuromodulation Unit, Houston, Texas	All HIC	Epilepsy and Behavior	***A journey into the unknown: An ethnographic examination of drug-resistant epilepsy treatment and management in the United States***	To further understand challenges living with and managing DRE, a team of medical anthropologists conducted ethnographic field assessments with patients to qualitatively understand their experience with DRE across the United States.	Mixed	Epilepsy	USA	Ethnography	Not reported	Patients AND Family members, carers, signifcant other AND HCPs (inc. NSx)	30	Mixed: Ethnography field assessments AND semi-structured interviews AND general observations	Other: inductive and deductive Analyzes, journey mapping, and needs clustering to illustrate an aggregate of the patient journey.	Not reported	Not reported	Ethnographic assessments of difficult-to-treat disease states have successfully identified patient–provider gaps not accessible by quantitative means [57]. By employing this observational science, we discovered the profound impact of untimely disease management leading up to and after receiving a DRE diagnosis. This delay manifests itself after patients are formally given the diagnosis of being drug-resistant, when missed disease education and non-pharmacological treatment opportunities are finally recognized. By identifying unique stages of the journey of a patient with epilepsy, we present treatment and management optimization strategies that could improve patients' sense of control over their epilepsy and empower them to become more active participants in their care. These strategies include incorporating resources to streamline patient–provider communication and introducing surgical treatment options earlier in patients' treatment journey (Fig. 3). Importantly, non-pharmacological treatments should be recognized as the gold-standard upon a patient's DRE diagnosis.
Acevedo, N. and Castle, D. and Bosanac, P. and Rossell, S.	Functional	2023	Australia	Other: PhD student	Centre for Mental Health,	All HIC	Brain Sciences	***Phenomenological Changes Associated with Deep Brain Stimulation for Obsessive Compulsive Disorder: A Cognitive Appraisal Model of Recovery***	to provide a comprehensive evaluation of DBS effects in individuals with OCD, with a view to providing scientific evidence on the spectrum of DBS induced effects, addressing neuroethical concerns and elucidating the patient perspective	Adult	OCD; DBS	Australia	Qualitative	implicit and latent approach	Patients AND Families/carers/ Significant other	12	Interviews (semi-structured, open, in depth)	Other: thematic, content, and interpretative phenomenological analysis (IPA)	Braun and Clark (2006); Alase (2017); Bengtsson (2016)	COREQ	Profound psychopathological changes were expressed; individuals felt more alive,<br>had improved cognitive affective control, greater engagement in the world, and were able to manage their OCD. Through suppression of the condition, self-constructs were able to re-emerge and develop. A framework describing the progression of phenomenological changes, and a theoretical model describing changes in the cognitive appraisal of intrusions influencing recovery are proposed
Thomson, C. J. and Segrave, R. A. and Fitzgerald, P. B. and Richardson, K. E. and Racine, E. and Carter, A.	Functional	2023	Australia	Psychologist	School of Psychological Sciences, Turner Institute for Brain and Mental Health; Wicking Dementia Research and Education Centre	All HIC	Plos One	***Personal and relational changes following deep brain stimulation for treatment-resistant depression: A prospective qualitative study with patients and caregivers***	to qualitatively examine how DBS for treatment-resistant depression impacts patient personality, self-concept, and relationships from the perspectives of both patients and caregivers.	Adult	DBS; depression	Australia	Qualitative	Not explicitly stated	Patients AND Families/carers/ Significant other	21	Interviews (semi-structured, open, in depth)	Thematic analysis	Braun and Clark (2006)	COREQ	Three primary themes were identified: (a) impact of mental illness and treatment on self-concept; (b) device acceptability and usability, and (c) relationships and connection. Severe refractory depression had profoundly impacted who patients were, how they viewed themselves, and the quality and functioning of their relationships. Patients who benefited from DBS felt reconnected with their premorbid self, yet still far from their ideal self. While reductions in depression were broadly beneficial for relationships, the process of adjusting relationship dynamics created new challenges. All patients reported recharging difficulties and challenges adapting to the device.
Bouchal, D. S. R. and Ferguson, A. L. and Green, T. and McAusland, L. and Kiss, Z. and Ramasubbu, R.	Functional	2023	Canada	Unclear/not stated	Faculty of Nursing	All HIC	Journal of Psychiatric and Mental Health Nursing	***Personal recovery associated with deep brain stimulation for treatment-resistant depression: A constructivist grounded theory study***	to explore processes of personal recovery in patients with treatment-resistant depression following subcallosal cingulate-deep brain stimulation.	Adult	DBS	CAN	Grounded theory	Constructivist	Patients AND Families/carers/ Significant other	29	Interviews (semi-structured, open, in depth)	Constant comparison	Charmaz, 2014	Not reported	While every participant and their families' journey were unique following the deep brain stimulation intervention, a theoretical model of Balancing to Establish a Reconstructed Self emerged from the data. The themes underlying the model were (1) Balancing to Establish a Reconstructed Self: A Whole-Body Experience, (2) The Liminal Space in-between: Balancing with Cautious Optimism, (3) Hope: Transitioning from Emotion-Focussed Living to Goal-Oriented Planning and (4) Support: Negotiating Relationships.

Harding, L. and McFarlane, J. and Honey, C. R. and McDonald, P. J. and Illes, J.	Functional	2023	Canada	Unclear/not stated	Neuroethics Canada, Division of Neurology, Department of Medicine,	All HIC	Canadian Journal of Neurological Sciences	***Mapping the Landscape of Equitable Access to Advanced Neurotechnologies in Canada***	To explore the barriers, enablers, and areas of opportunity for equitable and meaningful access to advanced neurotechnologies for diverse patient communities across Canada.	Mixed		CAN	Qualitative	pragmatic neuroethics interpretive framework	HCPs (Inc NSx)	24	Mixed/multiple: Semi-structured interviews AND rating scale questionnaires	Other: qualitative content analysis and thematic analysis	Braun and Clark (2006)	Not reported	Seven major themes emerged from the qualitative analysis: Health care system , Neurotechnology features , Patient demographics , Target condition features , Ethics , Upstream barriers and enablers , and Areas of opportunity . Descriptive statistics of the Likert-scale responses suggest that interviewees perceive a disparity between the imperative of access to advanced neurotechnologies for people living in rural and remote areas and the likelihood of achieving such access.
Moens, M. and Van Doorslaer, L. and Billot, M. and Eeckman, E. and Roulaud, M. and Rigoard, P. and Fobelets, M. and Goudman, L.	Functional	2024	Belgium	Unclear/not stated	Department of Neurosurgery; STIMULUS Research Group; Center for Neurosciences; Department of Radiology; Pain in Motion Research Group, Department of Physiotherapy, Human Physiology and Anatomy	All HIC	Journal of Medical Internet Research	***Examining the Type, Quality, and Content of Web-Based Information for People With Chronic Pain Interested in Spinal Cord Stimulation: Social Listening Study***	to explore the type, quality, and content of web-based information regarding spinal cord stimulation (SCS) for chronic pain that is freely available and targeted at health care consumers.	Unclear	Spinal cord stimulation	International	Qualitative	Not explicitly stated	Mixed (please state in comments)	630	Online social media content	Thematic analysis	Not stated	Not reported	The initial search identified 2174 entries, of which 630 (28.98%) entries were eventually withheld, which could be categorized as web pages, including news and blogs (114/630, 18.1%); Reddit (Reddit, Inc) posts (32/630, 5.1%); Vimeo (Vimeo, Inc) hits (38/630, 6%); or YouTube (Google LLC) hits (446/630, 70.8%). Most posts originated in the United States (519/630, 82.4%). Regarding the content of information, 66.2% (383/579) of the entries discussed (fully discussed or partially discussed) how SCS works. In total, 55.6% (322/579) of the entries did not elaborate on the fact that there may be >1 potential treatment choice and 47.7% (276/579) did not discuss the influence of SCS on the overall quality of life. The inductive coding revealed 4 main themes. The first theme of pain and the burden of pain (1274/8886, 14.34% coding references) explained about pain, pain management, individual impact of pain, and patient experiences. The second theme included neuromodulation as a treatment approach (3258/8886, 36.66% coding references), incorporating the background on neuromodulation, patient-centered care, SCS therapy, and risks. Third, several device-related aspects (1722/8886, 19.38% coding references) were presented. As a final theme, patient benefits and testimonials of treatment with SCS (2632/8886, 29.62% coding references) were revealed with subthemes regarding patient benefits, eligibility, and testimonials and expectations.
Elkaim, L. M. and Niazi, F. and Levett, J. J. and Bokhari, R. and Gorodetsky, C. and Breitbart, S. and Alotaibi, F. and Alluhaybi, A. A. and Weil, A. G. and Fallah, A. and Alotaibi, N. M. and Ibrahim, G. M.	Functional	2022	Canada	Physician/MD	Department of Neurology and Neurosurgery	All HIC	Neurosurgical Focus	***Deep brain stimulation in children and youth: perspectives of patients and caregivers gleaned through Twitter***	to descrbe the landscape of social media use surrounding DBS in children and youth.	Paediatric	DBS	International	Qualitative	Non stated	Mixed (pateints, caregivers, researchers, news media outlets)	877 Tweets from 816 accounts	Online Tweets	Modified thematic analysis; Open and axial coding	Non stated	Not reported	Most tweets were from patients or caregivers, researchers, or news media outlets. The most common themes among analyzed tweets were research discussing novel findings (45.2%) or personal experiences of patients or caregivers (27.4%). Sentiment analysis showed that 54.5% of tweets were positive, 35.1% were neutral, and 10.4% were negative. The presence of pictures or videos increased the tweet engagement count by an average of 10.5 (95% CI 7.3–13.6). Tweets about personal patient experiences (β = 6, 95% CI 0.95–12) and tweets tagging other accounts (β =3.2, 95% CI 0.63–5.8) were also significantly associated with higher engagement metrics
Kyte, E. B. and Skogan, A. H. and Baklid, A. B. and Malmgren, K. and Ozanne, A. and Alfstad, K. A.	Functional	2023	Norway	Neuropsychologist	The National Centre for Epilepsy,	All HIC	Epilepsy & Behavior	***Patients' long-term perspectives on gains and losses after temporal lobe resection for epilepsy***	To explore temporal lobe resection patients' experiences more than 10 years after surgery, their perceptions of change, and what influences long-term satisfaction with surgery	Adult	Epilepsy surgery	Norway	Qualitative	Non stated	Patients	50	Interviews (semi-structured, open, in depth)	Reflexive thematic analysis	Braun and Clarke (2006; 2019)	Not reported	Participants' answers were divided into two main themes: "looking back on surgery" and"considering gains and losses from surgery". Most participants expressed satisfaction with having undergone surgery. Nevertheless, postsurgical problems had been encountered, and presurgical hopes had only partly been fulfilled. They described memory and naming problems with a major impact on daily life. Further, they had thoughts about effects on employment, independence, and feelings of loneliness, and expressed a need for more and better preoperative information.
Shawahna, R. and Jaber, M.	Functional	2022	Palestine	Unclear/not stated	Department of Physiology, Pharmacology and Toxicology, Faculty of Medicine and Health Sciences	All LMIC	World Neurosurgery	***Development of Consensus-Based Recommendations to Prevent/Minimize Medication Errors in the Perioperative Care of Patients with Epilepsy: A Mixed-Method***	To explore medication errors in the perioperative care of patients with epilepsy and developed consensus-based recommendations to prevent/minimize these errors.	Unclear	epilepsy	Palestine	Mixed methods,	Interpretive	Patients AND clinicians	40	Interviews (semi-structured, open, in depth)	Other: Interpretive description	Thorne (1997)	COREQ	A total of 1400 minutes of interview time was analyzed in this study. Of the panelists, 39 (78.0%) agreed that patients with epilepsy present unique challenges to providers of perioperative care that make them prone to medication errors. The interviewees in this study described 32 different medication error situations that occurred while providing perioperative care services to patients with epilepsy. In this study, 35 consensus-based recommendations to prevent/minimize medication errors in the perioperative care of patients with epilepsy were developed.
van Westen, M. and Rietveld, E. and van Hout, A. and Denys, D.	Functional	2023	The Netherlands	Psychiatrist and philosopher	Department of Psychiatry	All HIC	Phenomenology and the Cognitive Sciences	***Deep brain stimulation is no ON/OFF-switch': an ethnography of clinical expertise in psychiatric practice***	To explore the role of clinical expertise through an ethnography of the case of DBS optimization in OCD.	Unclear	DBS for OCD	the Netherlands	Ethnography	Interpretive	HCPs (Inc NSx)	18 months observation + 9 interviews	Mixed: In-depth interviews and participant observation	Open and axial coding	Boeije and Bleijenbergh (2019)	Not reported	By repeatedly observing particular situations for an extended period of time, we found that there are recurrent patterns in the ways clinicians interact with patients. These patterns of clinical practice shape the possibilities clinicians have for making sense of DBS-induced changes in patients' lived experience and behavior. Collective established patterns of clinical practice are dynamic and change under the influence of individual learning experiences in particular situations, opening up new possibilities and challenges. We conclude that patterns of clinical practice and particular situations are mutually constitutive. Ethnography is ideally suited to bring this relation into view thanks to its broad temporal scope and focus on the life-world. Based on our findings, we argue that clinical expertise not only implies skillful engagement with a concrete situation but also with the patterns of clinical practice that shape what is possible in this specific situation. Given this constraining and enabling role of practices, it is important to investigate them in order to find ways to improve diagnostic and therapeutic possibilities.

Shahmoon, S. and Limousin, P. and Jahanshahi, M.	Functional	2023	UK	Other: Therapist	Unit of Functional Neurosurgery, Department of Clinical and Movement Neurosciences	All HIC	Parkinsons Disease	***Exploring the Caregiver Role after Deep Brain Stimulation Surgery for Parkinson's Disease: A Qualitative Analysis***	to explore how caregiver spouses make sense of themselves one and five years after their partner's deep brain stimulation (DBS) surgery for Parkinson's disease.	Adult	DBS / Parkinsons	UK	Qualitative	Not explicitly stated	Family/Carer/Significant other	16	Interviews (semi-structured, open, in depth)	Content analysis; IPA	Kondracki et al. (2002); Smith (2009)	Not reported	A content analysis showed (1) how these 8 caregivers shared less than half as many self-reflections than the other caregivers, (2) that there was a bias to reflect on their partner's experience answering the opening question, (3) the bias continued when answering subsequent questions, and (4) there was a lack of awareness of this bias. No other patterns of behaviour or themes were able to be extracted. The remaining 8 interviews were transcribed and analysed using IPA. This analysis discovered 3 inter-related themes: (1) DBS allows carers to question and shift the caregiver role, (2) Parkinson's unites and DBS divides, and (3) seeing myself and my needs, DBS enhances visibility. How these caregivers interacted with these themes depended on when their partners were operated. The results suggested that spouses maintained the role of caregiver one year post DBS because they struggle to identify themselves in any other way but were more comfortable reassociating into the role of spouse 5 years post surgery. Further inquiry into caregiver and patient identity roles post DBS is recommended as a means of supporting their psychosocial adjustment after surgery.
Waite, G. and Chugh, D. and Cawker, S. and Oulton, K. and Wray, J. and Harniess, P.	Functional	2023	UK	Paediatric physiotherapist	Physiotherapy Department	All HIC	Child: Care, Health and Development	***Wanting no regrets': Parental decision making around selective dorsal rhizotomy***	to explore parental experiences and expectation in the decision-making process around the SDR operation, physiotherapy treatment and outcomes.	Paediatric	Ceerebral palsy (selective dorsal rhizotomy)	UK	Qualitative	Constructivist	Family/Carer/Significant other	18	Interviews (semi-structured, open, in depth)	Thematic analysis	Braun and Clarke (2006)	Not reported	Decision making involved an interacting process between the 'parental information seeking experience', 'influence of professional encounters and relationships' and 'emotional and social aspects'. Despite underlying uncertainties about outcomes, parental drivers of expectations and aspirations for their child preceded and sustained this decision-making process. A foundational narrative of 'wanting no regrets' resolved their decision to undertake SDR. Outcomes did not always match expectations, and parents moved away from a position of idealism, which was driven in part by parental information seeking including overly optimistic media representation.
Boulicault, M. and Goering, S. and Klein, E. and Dougherty, D. and Widge, A. S.	Functional	2023	UK	Other: trained in empirical bioethics	Department of Philosophy	All HIC	Neuroethics	***The Role of Family Members in Psychiatric Deep Brain Stimulation Trials: More Than Psychosocial Support***	Analyzes the complex ways in which family relationships can afect DBS trial participation, and how DBS trial participation in turn infuences family relationships	Adult	DBS	USA	Qualitative	Constructionist	Patients AND Families/carers/ Significant other	14	Interviews (semi-structured, open, in depth)	Thematic analysis (Dyadic thematic analysis, inductive and theoretical)	Braun and Clark (2006); Manning, and Adrianne (2015)	Not reported	The frst theme encompasses how DBS recipients and their family members conceptualize the purpose of DBS. Themes 2 and 3 relate to diferent stages of participation in a DBS trial: the decision to enroll in a trial, and the process of understanding and navigating personal identity following implantation, respectively. Theme 4 relates to how the experience of undergoing DBS infuences relationships, and in turn, theme 5 relates to how relationships infuence experiences. Theme 6 concerns the values and priorities of family members.
Balzekas, I. and Richardson, J. P. and Lorence, I. and Lundstrom, B. N. and Worrell, G. A. and Sharp, R. R.	Functional	2024	USA	Other: MD-PhD candidate	Bioelectronics Neurophysiology and Engineering Laboratory	All HIC	Neurology-Clinical Practice	***Qualitative Analysis of Decision to Pursue Electrical Brain Stimulation by Patients With Drug-Resistant Epilepsy and Their Caregivers***	To better understand patients' decisions to purse invasive Electrical Brain Stimulation for Drug-Resistant Epilepsy	Adult	DBS; Epilepsy	USA	Qualitative	Non stated	Patients AND Families/carers/ Significant other	45	Interviews (semi-structured, open, in depth)	Other: Modified inductive	McQueen et al. (1998)	Not reported	Patients' motivations included (1) improved quality of life (2) intolerability of antiseizure medications, (3) desperation, and (4) patient-family dynamics. Both patients and caregivers described a desire to alleviate burdens of the other. Patient apprehensions about EBS focused on invasiveness and the presence of electrodes in the brain. Previous experiences with invasive monitoring and the ability to see hardware in person during clinical visits influenced patients' comfort in proceeding with EBS. Despite realistic expectations for modest and delayed benefits, patients held out hope for an exceptionally positive outcome.
Dorfman, N. and Snellman, L. and Kerley, Y. and Kostick-Quenet, K. and Lazaro-Munoz, G. and Storch, E. A. and Blumenthal-Barby, J.	Functional	2023	USA	Unclear/not stated	Center for Medical Ethics and Health Policy	All HIC	Neuroethics	***Hope and Optimism in Pediatric Deep Brain Stimulation: Key Stakeholder Perspectives***	To examine stakeholder experiences and perspectives on hope and unrealistic optimism in the context of decision-making about DBS for childhood dystonia and provides insights for clinicians seeking to implement efective communication strategies.	Paediatric	DBS; Dystonia	USA	Qualitative	Non stated	Families/carers/ Significant other AND clinicians	73	Interviews (semi-structured, open, in depth)	Content analysis	Boyatzis (1998)	Not reported	Clinicians expressed concerns about caregiver false hopes (86%, 25/29) and desperation (68.9%, 20/29) in light of DBS being a last resort. As a result, 68.9% of clinicians (20/29) expressed that they intentionally tried to lower caregiver expectations about DBS outcomes. Clinicians also expressed concern that, on the fip side, unrealistic pessimism drives away some patients who might otherwise beneft from DBS (34.5%, 10/29). Caregivers viewed DBS as the last option that they had to try (61.3%, 27/44), and 73% of caregivers (32/44) viewed themselves as having high hopes but reasonable expectations. Fewer than half (43%, 19/44) expressed that they struggled setting outcome expectations due to the uncertainty of DBS, and 50% of post-DBS caregivers (14/28) expressed some negative feelings post treatment due to unmet expectations. 43% of caregivers (19/44) had experiences with clinicians who tried to set low expectations about the potential benefts of DBS.
Haeusermann, T. and Liu, E. Y. and Fong, K. C. and Dohan, D. and Chiong, W.	Functional	2024	USA	Unclear/not stated	UCSF Weill Institute for Neurosciences	All HIC	Epilepsy & Behavior	***Patient experiences of resection versus responsive neurostimulation for drug-resistant epilepsy***	to explore the perspectives and experiences of both Patients undergoing traditional resection as well as those implanted with Responsive Neurostimulation System to capture the spectrum of considerations in surgical epilepsy more fully.	Adult	epilepsy	USA	Ethnography	Non stated	Patients AND Families/carers/ Significant other	32	Mixed/multiple: Clinic observations AND Semi-structured interviews	Other: Unclear	non stated	Not reported	Using an applied ethnographic approach, we identified three major themes in the experiences of resection versus RNS patients. First, for patients in both cohorts, the therapeutic journey was circuitous in ways that defied standardized first-, second-, and third- line of care models. Second, in conceptualizing risk, resection patients emphasized the permanent loss of "taking out" brain tissue whereas RNS patients highlighted the reversibility of "putting in" a device. Lastly, in considering benefit, resection patients perceived their surgery as potentially curative while RNS patients understood implantation as primarily palliative with possible additional diagnostic benefit from chronic electrocorticography.
Haeusermann, Tobias and Lechner, Cailin R. and Fong, Kristina Celeste and Bernstein Sideman, Alissa and Jaworska, Agnieszka and Chiong, Winston and Dohan, Daniel	Functional	2023	USA	Unclear/not stated	Decision Lab, UCSF Memory and Aging Center	All HIC	AJOB neuroscience	***Closed-Loop Neuromodulation and Self-Perception in Clinical Treatment of Refractory Epilepsy***	to observe how conceptual neuroethical concerns manifest in clinical treatment with responsive neurostimulation (RNS) as a treatment for refractory epilepsy	Adult	epilepsy	USA	Ethnography	Non stated	Patients AND Families/carers/ Significant other	24	Mixed/multiple: Clinic observations AND interviews	Other: Deductive and inductive analysis	non stated	Not reported	Participants generally did not attribute changes in patients' personalities or self-perception to implantation of or stimulation using RNS. They did report that RNS affected patients' experiences and conceptions of illness. In particular, the capacity to store and display electrophysiological data produced a common frame of reference and a shared vocabulary among patients and clinicians.

Makoshi, Z. and Raskin, J. and Bollo, R. and Rocque, B. and Zickmund, S. and Galyean, P. and Perry, G. and Browd, S. and Gross, P. and Bjornson, K. and Leonard, J.	Functional	2023	USA	Unclear/not stated	Department of Neurosciences	All HIC	Pediatric Neurology	***Research Paper A Mixed Methods Study of Practice Variation in Selective Dorsal Rhizotomy: A Study by the Cerebral Palsy Research Network***	to investigate provider perception about SDR candidates against the characteristics of those undergoing SDR in the CP Research Network (CPRN) registry.	Paediatric	Cerebral palsy; selective dorsal rhizotomy	USA	Mixed methods,	Not explicitly stated	Other (please state in comments)	41	Interviews (semi-structured, open, in depth)	Thematic analysis	Not stated	SRQR	SDR was performed in 238 individuals, majority aged eight to 12 years (n = 105), GMFCS level II (n = 46), and white (n = 183). Most neurosurgeons perform a single-level SDR. Providers believe the majority of individuals undergoing SDR are between five and six years and GMFCS level II with variable agreement. There was no significant agreement about the youngest age ($P = 0.451$) or ideal GMFCS level ($P = 0.451$) for SDR. Providers had agreement on the oldest age for SDR ($P = 0.041$), how to screen for dystonia ($P < 0.001$), and dystonia as a contraindication for SDR ($P < 0.0005$).
Merner, A. R. and Kostick-Quenet, K. and Campbell, T. A. and Pham, M. T. and Sanchez, C. E. and Torgerson, L. and Robinson, J. and Pereira, S. and Outram, S. and Koenig, B. A. and Starr, P. A. and Gunduz, A. and Foote, K. D. and Okun, M. S. and Goodman, W. and McGuire, A. L. and Zuk, P. and LÃ¡zaro-MuÃ±oz, G.	Functional	2023	USA	Psychologist	Center for Bioethics	All HIC	Brain Stimulation	***Participant perceptions of changes in psychosocial domains following participation in an adaptive deep brain stimulation trial***	to examine the perspectives of patients who underwent DBS regarding changes to their personality, authenticity, autonomy, risk-taking, and overall quality of life.	Adult	DBS	USA	Mixed methods,	Not explicitly stated	Patients	21	Interviews (semi-structured, open, in depth)	Content analysis	Braun and Clarke (2006) and Boyatzis (1998)	Not reported	Patients (n = 21) who were enrolled in adaptive DBS trials for Parkinson's disease, essential tremor, obsessive-compulsive disorder, Tourette's syndrome, or dystonia participated. Qualitative data revealed that participants, in general, reported positive experiences with alterations in what was described as 'personality, mood, and behavior changes.' The majority of participants reported increases in quality of life. No participants reported 'regretting the decision to undergo DBS.'
Merner, Amanda R. and Frazier, Thomas W. and Ford, Paul J. and Lapin, Brittany and Wilt, Joshua and Racine, Eric and Gase, Natalie and Leslie, Essence and Machado, Andre and Vitek, Jerrold L. and Kubu, Cynthia S.	Functional	2024	USA	Unclear/not stated	Center for Neurological Restoration; Center for Bioethics	All HIC	JAMA network open	***A Patient-Centered Perspective on Changes in Personal Characteristics After Deep Brain Stimulation***	To determine whether DBS is associated with changes in characteristics that patients with PD identify as personally meaningful.	Adult	DBS	USA	Mixed methods,	Not explicitly stated	Patients AND Families/carers/ Significant other	104	Interviews (semi-structured, open, in depth)	Content analysis	Not stated	Not reported	Fifty-two of 54 dyads of patients with PD and their care partners (96.3%) were recruited from a consecutive series approved for DBS (36 patients [69.2%] were male and 45 care partners [86.5%] were female; mean [SD] age of patients, 61.98 [8.55] years). Two patients and 1 care partner were lost to follow-up. Increases in the mean VAS score (indicative of greater manifestation of [ie, positive changes in] specific characteristics) were apparent following DBS for ratings of both the patients (Wald $\chi2 = 16.104$; $P < .001$) and care partners (Wald $\chi2 = 6.746$; $P < .001$) over time. The slopes of the changes for both the patient and care partners were correlated, indicating agreement in observed changes over time. The individual level analyses indicated that scores for most patients and care partners remained the same or increased.
Peabody Smith, A. and Taiclet, L. and Ebadi, H. and Levy, L. and Weber, M. and Caruso, E. M. and Pouratian, N. and Feinsinger, A.	Functional	2023	USA	Unclear/not stated	Department of Medicine	All HIC	AJOB Empirical Bioethics	***"They were already inside my head to begin with": Trust, Translational Misconception, and Intraoperative Brain Research***	To explore interpretations of risks and benefits, enrollment motivations, and experiences of participating in awake brain research	Adult	DBS	USA	Qualitative	Not explicitly stated	Patients	14	Interviews (semi-structured, open, in depth)	Reflexive thematic analysis	Braun and Clarke (2006), Braun and Clarke (2012), Braun and Clarke (2019)	Not reported	Seven themes were identified from participant narratives, including robust attitudes of trust, high valuations of basic science research, impacts of the surgical context, and mixed experiences of participation
Smith, J. N. and Dorfman, N. and Hurley, M. and Cenolli, I. and Kostick-Quenet, K. and Lazaro-Munoz, G. and Storch, E. A. and Blumenthal-Barby, J.	Functional	2023	USA	Unclear/not stated	Center for Medical Ethics and Health Policy	All HIC	Clinical Ethics	***Perspectives on informed assent and bodily integrity in prospective deep brain stimulation for youth with refractory obsessive-compulsive disorder***	to solicit and assess the views of stakeholders (children, parents, clinicians) on pediatric assent, autonomy, and bodily integrity in the context of potential pediatric deep brain stimulation for obsessive-compulsive disorder.	Paediatric	DBS for OCD	USA	Qualitative	Not explicitly stated	Patients AND Families/carers/ Significant other AND HCPs	65	Interviews (semi-structured, open, in depth)	Thematic content analysis	Not stated	Not reported	A majority of respondents (74%, 48/65) across all three stakeholder groups voiced that the decision-making process should be collaborative and involve everyone (clinicians: 84% or 21/25, caregivers 71% or 15/21, and patients 63% or 12/19). We identified a split between respondents' views on who should have the final say in the event of disagreement (38% or 25/65 favored the patient versus 35% or 23/65 favoring caregivers). A split between respondents also emerged concerning the maturity relevant for deep brain stimulation decision-making, with 45% (29/65) favoring developmental maturity (age/physiological development) and 45% (29/65) favoring decisional maturity (capacity to understand and weigh information). A majority of clinicians indicated that they would not move forward with deep brain stimulation without securing patient assent (80% or 20/25), with some stating the only exception is if patient quality of life was very poor and/or they lacked insight. Both caregivers and patients expressed a significant respect for the patient's right to bodily integrity, with 67% of caregivers (14/21) and 68% of patients (13/19) justifying patient involvement in decision-making specifically with reference to infringements of bodily integrity.

Smith, J. N. and Dorfman, N. and Hurley, M. and Cenolli, I. and Kostick-Quenet, K. and Storch, E. A. and LÃ¡zaro-MuÃ±oz, G. and Blumenthal-Barby, J.	Functional	2024	USA	Unclear/not stated	Center for Medical Ethics and Health Policy	All HIC	Cambridge Quarterly of Healthcare Ethics	***Adolescent OCD Patient and Caregiver Perspectives on Identity, Authenticity, and Normalcy in Potential Deep Brain Stimulation Treatment***	To understand patients' views about identity changes due to DBS in obsessive-compulsive disorder (OCD)	Adolescents	DBS for OCD	USA	Qualitative	Not explicitly stated	Patients AND Families/carers/ Significant other	40	Interviews (semi-structured, open, in depth)	Thematic content analysis	Not stated	Not reported	All patient respondents and half of caregivers reported that DBS would impact patient self-identity in significant ways. For example, many patients expressed how DBS could positively impact identity by allowing them to explore their identities free from OCD. Others voiced concerns that DBS-related resolution of OCD might negatively impact patient agency and authenticity. Half of patients expressed that DBS may positively facilitate social access through relieving symptoms, while half indicated that DBS could increase social stigma. These views give insights into how to approach decision-making and informed consent if DBS for OCD becomes available for adolescents. They also offer insights into adolescent experiences of disability identity and "normalcy" in the context of OCD.
Jenkin, T. and D'Cruz, K. and Anderson, V. and Scheinberg, A. and Knight, S.	Mixed	2023	Australia	Other: Master of Psychology and PhD student	Murdoch Children's Research Institute; Melbourne School of Psychological Sciences	All HIC	Disability and Rehabilitation	***Family-centred service in paediatric acquired brain injury rehabilitation: perspectives of children and adolescents and their families***	to develop a better understanding of the experiences of children/adolescents with moderate to severe ABI and their families regarding family-centred service and what it means to them. It also aimed to explore how family members participate in rehabilitation and whether children/adolescents with ABI and their families would change anything about family participation.	Mixed	Mixed ABI	Australia	Grounded theory	Constructivist	Patients AND Families/carers/ Significant other	19	Interviews (semi-structured, open, in depth)	Grounded theory analysis	Charmaz, 2014	Not reported	wo themes and five sub-themes were developed: (1) Working together as a team: Valuing clinicians' expert knowledge; Doing rehabilitation together; and Sharing family knowledge; and (2) Navigating rehabilitation as a family: Recognising family needs; Juggling family life; and Making rehabilitation work for the family. Participants reflected on the centrality of the child/adolescent with ABI during rehabilitation, and the ways that family needs, life, and involvement in rehabilitation change over time.
Backhouse and Rodger	Mixed	1999	Australia	Occupational therapist	The Department of Occupational Therapy	All HIC	Australian Occupational Therapy Journal	***The transition from school to employment for young people with acquired brain injury: Parent and student perceptions***	to examine the perceptions and experiences of adolescents with ABI and their parents in relation to their school to work transition.	Adolescents	Mixed ABI	Australia	Qualitative	Not reported	Patients AND Family members, carers, signifcant others	14	Focus groups	Inductive	Not reported	Not reported	Results of the study were categorized according to the three questions posed during the focus groups. These related to school integration, future goals, and assistance and impediments in reaching these goals.
Brakenridge, Leow, Kendall, et al.	Mixed	2021	Australia	Post-doctoral research fellow	RECOVER Injury Research Center	All HIC	Disability and Rehabilitation	***Exploring the lived return-to-work experience of individuals with acquired brain injury: use of vocational services and environmental, personal and injury-related influences***	To explore work outcomes, vocational services, barriers and facilitators for returning to work in individuals with acquired brain injury (ABI) in Queensland, Australia and to identify areas for improvement.	Adult	Mixed ABI	Australia	Qualitative	Realist approach	Patients	10	Interviews (open, unstructured, in-depth, semi-structured, open ended)	Thematic analysis: Realist	Braun & Clarke (2006)	COREQ	Participants either returned to the same work, different work, did not maintain work or did not have any work since their injury. Use of vocational services depended on participants' needs and insurance. Facilitators for return to work (RTW) were a supportive workplace and family, vocational rehabilitation that met the individual's needs, insurance coverage and self-motivation. Workplaces that were not understanding of brain injury, employment service providers who were unable to find work for participants, and physical and cognitive deficits were barriers to RTW. Workplaces, employment service providers and individuals require more information about the deficits associated with brain injury.
Butera-Prinzi and Perlesz	Mixed	2004	Australia	Not reported	The Bouverie Center, La Trobe University	All HIC	Brain Injury	***Through children's eyes: Children's experience of living with a parent with an acquired brain injury***	What has the experience been like post-trauma for a small sample of children whose father has an ABI?	Adult	Mixed ABI	Australia	Qualitative	Phenomenological framework	Family members, carers, signifcant others	4	Mixed: In-depth semi-structured interviews AND questionnaire	Other: Inductive analysis	Minichiello et al (1995)	Not reported	Significant themes to emerge from the interviews included the children's experience of their fathers becoming ill and being hospitalized; the 'loss' of their fathers and of their mothers to their fathers' injuries; facing, coping with and reconciling the changes in their fathers, who had transformed from loving competent parents to difficult and childlike fathers; the impact of the ABI on other aspects of their lives; the availability of support networks; the complexity of emotions expressed by the children; their resilience and the positive outcomes gained from their experiences despite their sense of adversity.
Dawes, Carlino, Van den Berg, et al.	Mixed	2022	Australia	Social Worker	South Australian Brain Injury Rehabilitation Service	All HIC	Disability and Rehabilitation	***Life altering effects on children when a family member has an acquired brain injury; a qualitative exploration of child and family perceptions***	To investigate the impact of familial acquired brain injury on children and adult family members, including their views of the support provided, gaps and recommendations for future interventions.	Unclear	Mixed ABI	Australia	Phenomenology	Not reported	Family members, carers, signifcant others	26	Interviews (open, unstructured, in-depth, semi-structured, open ended)	Grounded theory analysis/constant comparison/open, axial and/or selective coding (Thematic, inductive, deductive)	Not reported	Not reported	Analyzes revealed four main themes: (1) help parents help their children, (2) improve family functioning by giving children meaningful roles, (3) staff: don't leave children "in the dark," and (4) support for children is not one size fits all.
Gould, Carminati and Ponsford	Mixed	2021	Australia	Not reported	Turner Institute for Brain and Mental Health, School of Psychological Sciences, Monash University & Monash Epworth Rehabilitation Research Center, Epworth Healthcare	All HIC	Neuropsychological rehabilitation	***"They just say how stupid I was for being conned". Cyberscams and acquired brain injury: A qualitative exploration of the lived experience of survivors and close others***	To qualitatively explore the lived experiences of cyberscams and the aftermath from the perspectives of survivors with ABI and their COs	Adult	Mixed ABI	Australia	Qualitative	Constructivist	Patients AND Family members, carers, signifcant others	13	Interviews (open, unstructured, in-depth, semi-structured, open ended)	Reflexive thematic analysis	Braun & Clarke (2006)	COREQ	Reflexive thematic analysis of interview transcripts identified seven themes: "who is at the helm?: vulnerabilities," "the lure: scammer tactics," "scammers aboard: scam experience," "the discovery," "sinking in: impacts," "responding to the mayday: responses from others," and "lifesavers: suggestions for intervention." The journey towards scam victimization was complex, and complicated by the ABI. Cyberscams contributed to substantial financial disadvantage, loss of trust and shame. ABI related impairments and social isolation reportedly increased scam vulnerability and interfered with intervention attempts by family and professionals. Confusion, denial and disbelief created further barriers to discovery. The practical and emotional impacts on both cyberscam survivors with ABI and their family members, and a lack of effective intervention, highlight the need for increased education and awareness in order to improve online safety for those with ABI.
Hall, Grohn, Nalder, et al.	Mixed	2012	Australia	Not reported	The University of Queensland	All HIC	Brain Impairment	***A mixed methods study of the experience of transition to the community of working-aged people with non-traumatic brain injury***	To explore the transition experiences of individuals with nontraumatic brain injury using mixed methods approach.	Adult	Mixed ABI	Australia	Mixed methods (covergent design)	Not reported	Patients	6	Mixed: Interviews (open, unstructured, in-depth, semi-structured, open ended) AND questionnaires	Content analysis	Graneheim & Lundman (2004)	Not reported	Qualitative content analysis of interviews identified three themes: (1) changes in role performance, (2) support and services and (3) coping with life after brain injury. The transition experience was characterized by loss of valued roles including driving and work, identified as major barriers to regaining independence post discharge. Informal support provided by family and friends were relied on, while formal supports were accessed infrequently. Life post-injury presented a number of challenges including adjusting to changes in physical and cognitive abilities and a fear of reinjury. Qualitative data were supported by an overall trend of improved functioning on the quantitative measures over the 6 months.

Killington, Pearson, Campbell, et al.	Mixed	2021	Australia	Not reported	South Australia Department of Health, South Australia Brain Injury Rehabilitation Services, Central	All HIC	International Journal of Therapy and Rehabilitation	***Managing fatigue after an acquired brain injury: A pilot randomized controlled trial and qualitative investigation***	To determine whether therapy supporting fatigue management can be provided economically in groups to inpatients undertaking rehabilitation.	Adult	Mixed ABI	Australia	Mixed methods (a pilot randomized control trial and a parallel qualitative investigation)	Not reported	Patients	10	Interviews (open, unstructured, in-depth, semi-structured, open ended)	Thematic analysis	Krefting, (1991)	Not reported	A repeated measures analysis of variance with time as a within-subject factor and group as a between-subjects factor showed no interaction effect of group × time for fatigue, quality of life or acquisition of knowledge; however, knowledge improved over time, irrespective of group allocation (P<0.01). Although most participants found the group work satisfactory in terms of overall knowledge development, a number would have preferred to address their specific fatigue issues in an individual session. Participants described a confusing journey understanding fatigue symptoms before therapy, but reported an improved understanding and acquiring management strategies after receiving the educational therapy sessions.
Kitter and Sharman	Mixed	2015	Australia	Not reported	School of Social Sciences	All HIC	Brain Injury	***Caregivers support needs and factors promoting resiliency after brain injury***	To explore the challenges, support needs and coping strategies of caregivers of people with an acquired brain injury (ABI).	Mixed	Mixed ABI	Australia	Qualitative	Not reported	Family members, carers, signifcant others	20	Interviews (open, unstructured, in-depth, semi-structured, open ended)	Thematic analysis	Braun & Clarke (2006), Attride-Stirling (2001)	Not reported	Through thematic data analysis, three central themes were revealed: (a) barriers impeding quality-of-life, (b) support needed to improve quality-of-life and (c) factors enabling quality-of-life. All perspectives from the participants involved are synthesized to provide a rich depiction of caregivers' support needs and coping strategies.
Liang, Fleming, Gustafsson, et al.	Mixed	2016	Australia	Not reported	Division of Occupational Therapy, School of Health and Rehabilitation Sciences,	All HIC	Brain Injury	***Family members' experiences of driving disruption after acquired brain injury***	1) To explore family members' lived experiences of driving disruption at early and later stages of the recovery continuum following acquired brain injury (ABI). 2) To describe health-related quality of life of family members of individuals with ABI who are experiencing driving disruption.	Adult	Mixed ABI	Australia	Mixed methods (Descriptive phenomenologi cal research approach)	Phenomenologi cal	Family members, carers, signifcant others	15	Mixed: Interviews (open, unstructured, in-depth, semi-structured, open ended) AND questionnaires	Thematic analysis	Patton (2015)	Not reported	Two main themes were identified: Different for everyone: how driving disruption affects families, and Making it harder: context of driving disruption. The challenges of driving disruption were reported more frequently and with a more intense focus by family members who were caring for their relative for more than 1 year post-injury. This group also reported higher caregiver strain and poorer health-related quality of life. Reduced satisfaction with life, poor mental health and affected family functioning were reported by both groups.
Liang, Liddle, Fleming, et al.	Mixed	2016	Australia	Not reported	Division of Occupational Therapy, School of Health and Rehabilitation Sciences	All HIC	Australian occupational therapy journal	***Family members' narratives of lifespace: Mapping changes before and after a brain injury causing driving disruption***	To describe the quantitative changes in family members' lifespace after brain injury and understand their subjective experiences through interacting with maps during narratives.	Adult	Mixed ABI	Australia	Mixed methods (Mapping; Narrative analysis)	Not reported	Family members, carers, signifcant others	15	Mixed: Interviews (open, unstructured, in-depth, semi-structured, open ended) AND Maps to capture quantitative and qualitative aspects of lifespace.	Narrative analysis	Howie (2013)	Not reported	Quantitative data from the mapping revealed an increase in travel locations for nine participants, a decrease for five, and no change for one participant. Data analysis revealed four typologies which complemented and enriched the quantitative data: (i) I will do everything for him or her; (ii) Trying to fit all in; (iii) We spend all our time together now; (iv) I need to also care for myself.
Liang, Fleming, Gustafsson, et al.	Mixed	2016	Australia	Occupational therapist	School of Health and Rehabilitation Sciences, Division of Occupational Therapy,	All HIC	British Journal of Occupational Therapy	***Occupational experience of caregiving during driving disruption following an acquired brain injury***	To explore, using a phenomenological approach, family members' lived experiences of the occupations they take on during driving disruption following acquired brain injury (ABI).	Adult	Mixed ABI	Australia	Phenomenology (Descriptive)	Phenomenology	Family members, carers, signifcant others	15	Interviews (open, unstructured, in-depth, semi-structured, open ended)	Thematic analysis	Patton (2015)	Not reported	The occupational experiences related to caregiving during driving disruption emerged as a key finding. The meaning and activities comprising the caregiving occupation during driving disruption are captured in three themes: (1) More than just driving; (2) The invisible and undervalued care and (3) Being a therapist at home. Family members highlighted the challenges of managing broader and multiple responsibilities.
Manchella, Khurana, Duke, et al.	Mixed	2011	Australia	Not reported	Department of Neurosurgery	All HIC	British Journal of Neurosurgery	***The experience of patients undergoing awake craniotomy for intracranial masses: Expectations, recall, satisfaction and functional outcome***	To assess the expectations, recall, satisfaction and functional outcome of 26 consecutive patients undergoing awake craniotomy using the 'asleep–awake asleep' protocol.	Adult	Mixed ABI; awake craniotomy	Australia	Qualitative	Not reported	Patients	26	Interviews (open, unstructured, in-depth, semi-structured, open ended)	Thematic analysis (Modified)	Pope & Mays (2006)	Not reported	The following themes emerged from this study: (1) most patients demonstrated a good understanding of the rationale behind awake craniotomy; (2) patients felt the asleep–awake–asleep Anesthetic protocol used in this series was appropriate; (3) patients' confidence and preparedness for surgery was high, attributed to preparation by the surgical team. Seven of 26 (27%) patients had no recollection of being awake. Most patients had a positive Anesthetic and surgical experience, while a minority of patients reported experiencing more than slight pain (2/26; 8%) and discomfort (3/26; 12%), fear (4/26; 15%) or claustrophobia (1/26; 4%) intra-operatively. At follow-up (6 weeks post-operatively), most patients were functionally unimpaired; there was only one permanent neurological complication of surgery. We found that 24/26 (92%) patients were satisfied with their experience; one patient had no opinion and another one was unsatisfied. Five of 26 (19%) patients still reported more than slight discomfort, and 3/26 (12%) reported more than slight pain attributable to the surgery. A summary of the English peer-reviewed literature on the patient experience of awake craniotomy is also incorporated.
Sharp, Bye, Llewellyn, et al.	Mixed	2006	Australia	Not reported	Occupational Therapy Program, College of Health and Science	All HIC	Disability and Rehabilitation	***Fitting back in: Adolescents returning to school after severe acquired brain injury***	To explore the experiences of Australian adolescents with severe acquired brain injury (ABI) and their families as the adolescent returned to school. In particular, to understand the influence of services and support on the school return.	Adolescents	Mixed ABI	Australia	Grounded theory	Not reported	Family members, carers, signifcant others	8	Interviews (open, unstructured, in-depth, semi-structured, open ended)	Grounded theory analysis/consta nt comparison/op en, axial and/or selective coding	Strauss & Corbin (1998)	Not reported	wo critical phases for adolescents returning to school post ABI were identified: organizing the school return and being back at school. Experience in these phases was influenced by length of school absence, extent of noticeable and hidden problems and school response to the students' return post injury. The central concept characterizing experience was the challenge of fitting back in. Some adolescents fitted back in adequately and continued school participation. Those that did not fit in tried again by revisiting the organizing phase, re-evaluated their school experience and either continued or left school.
Turner, Fleming, Cornwell, et al.	Mixed	2007	Australia	Not reported	School of Health and Rehabilitation Sciences	All HIC	Brain Injury	***A qualitative study of the transition from hospital to home for individuals with acquired brain injury and their family caregivers***	To explore the transition experiences from hospital to home of a purposive sample of individuals with acquired brain injury (ABI).	Adult	Mixed ABI	Australia	Phenomenology	Phenomenologi cal	Patients AND Family members, carers, signifcant others	24	Interviews (open, unstructured, in-depth, semi-structured, open ended)	Inductive	Patton (2002)	Not reported	Through the inductive analysis process, a summary coding framework was developed that included that following eight main categories: the hospital experience; the transition process; the role of family caregivers; post-discharge services; friendship networks and community involvement; meaningful activities and time management; physical and psychological wellbeing; and barriers and facilitators.

Turner, Fleming, Ownsworth, et al.	Mixed	2011	Australia	Not reported	School of Health and Rehabilitation Sciences	All HIC	Disability and Rehabilitation	***Perceived service and support needs during transition from hospital to home following acquired brain injury***	To explore the service and support needs of individuals with acquired brain injury (ABI) and their family caregivers during the transition phase from hospital to home.	Adult	Mixed ABI	Australia	Other: Phenomenological & grounded theory approaches	Not reported	Patients AND Family members, carers, signifcant others	38	Interviews (open, unstructured, in-depth, semi-structured, open ended)	Grounded theory analysis/constant comparison/open, axial and/or selective coding (Thematic)	Liamputtong & Ezzy (2005)	Not reported	The following primary themes, each with associated secondary themes, emerged from the analysis: (1) balancing the service and support equation; (2) negotiating the rehabilitation maze; (3) working with or against 'the system'. The first theme describes the varying types and level of support received by participants during the transition phase, while the second theme highlights the difficulties participants experienced in negotiating the rehabilitation process between hospital and home. The final theme depicts the challenges experienced by participants in accessing and utilizing service support.
Turner, Fleming, Ownsworth, et al.	Mixed	2011	Australia	Not reported	School of Health and Rehabilitation Sciences	All HIC	Neuropsychological Rehabilitation	***Perceptions of recovery during the early transition phase from hospital to home following acquired brain injury: A journey of discovery***	To explore the perspectives of individuals with ABI and their family caregivers concerning recovery and adjustment during the early transition phase from hospital to home.	Adult	Mixed ABI	Australia	Phenomenology	Phenomenological	Patients AND Family members, carers, signifcant others	38	Interviews (open, unstructured, in-depth, semi-structured, open ended)	Grounded theory analysis/constant comparison/open, axial and/or selective coding (Thematic)	Liamputtong & Ezzy (2005)	Not reported	the findings highlight that while returning home was typically perceived to facilitate ongoing recovery, the process of adjusting emotionally to life at home posed a significant challenge for many participants during the transition phase. The clinical/service implications of the findings relate to the need for: (1) contextually appropriate rehabilitation options during the transition phase; and (2) the expansion of transition-based models of service delivery to include targeted psychological intervention approaches.
Winkler, Farnworth, Sloan, et al.	Mixed	2011	Australia	Occupational therapist	Summer Foundation,	All HIC	Brain Injury	***Moving from aged care facilities to community-based accommodation: Outcomes and environmental factors***	To explore the transition experiences of young people with acquired brain injury who have lived in aged care facilities and moved into community-based settings.	Adult	Mixed ABI	Australia	Grounded theory	Not reported	Patients AND Family members, carers, signifcant others AND HCPs	16	Interviews (open, unstructured, in-depth, semi-structured, open ended)	Grounded theory analysis/constant comparison/open and axial/selective coding	Not reported	Not reported	Participants identified a range of positive outcomes that resulted from the transition from aged care settings to community living environments including increased independence in everyday activities, improved well-being and a greater degree of social inclusion. Participants also identified environmental factors that they deemed as crucial to facilitating positive outcomes.
Goudman, Bruzzo, van de Sande, et al.	Mixed	2020	Belgium	Not reported	Department of Neurosurgery; Department of Physiotherapy, Human Physiology and Anatomy, Faculty of Physical Education & Physiotherapy	All HIC	Pain Practice	***Goal Identification Before Spinal Cord Stimulation: A Qualitative Exploration in Potential Candidates***	As a first step in creating patient empowerment after SCS implantation, patients' goals will be explored. Therefore, this qualitative study concerns an in-depth interview of patients eligible for SCS to explore their individual goals.	Adult	Failed back/neck surgery syndrome and spinal cord stimulation	Belgium	Qualitative	International Classification of Functioning, Disability and Health (ICF) framework	Patients	15	Interviews (open, unstructured, in-depth, semi-structured, open ended)	Thematic analysis	Tesch (1990)	Not reported	In the domain of bodily functions, all patients mentioned pain reduction, and 1 patient wanted to regain his previous sleep pattern. In the domain of activities, walking, sitting, driving a car, bending down, and picking up were the highest ranked goals. Regaining a social life was the highest ranked goal for participation. Eleven patients wanted to regain a feeling of happiness, and 5 patients wanted to focus on avoiding depression.
Engel, L. and Ewesesan, R. and Arowolo, I. and Latulipe, C. and Karpa, J. and Khan, M. N.	Mixed	2024	Canada	Unclear/not stated	Department of Occupational Therapy	All HIC	Archives of Rehabilitation Research and Clinical Translation	***Financial Capability and Financial WellBeing Challenges and Vulnerabilities of Adults Living With Acquired Brain Injury: A Pilot Survey***	To examine the financial capability and financial well-being of adults living with ABI.	Adult	Mixed ABI	CAN	Survey/Questionnaire	Non stated	Patients AND Families/carers/Significant other	57	Open ended survey questions	Framework method	Gale, et al. (2013)	Not reported	For key financial capability indicators, 13 (34%) people living with ABI felt their current knowledge and skills were insufficient, and 26 (70%) felt that ABI had affected their ability to make financial decisions or complete financial activities. Fourteen of the 19 close others have worried about the finance-related choices, skills, or behaviors of the person living with ABI, and 17 felt that ABI symptoms had affected the FC of the person living with ABI. For key FWB indicators, 22 (58%) adults living with ABI felt stressed or anxious about finances at least some of the time. Seventeen (45%) of the adults living with ABI reported having trouble making ends meet at least some of the time
Clifford, Sharpe, Khu, et al.	Mixed	2009	Canada	Not reported	Division of Neurosurgery	All HIC	Journal of Neuro-Oncology	***Gamma Knife patients' experience: Lessons learned from a qualitative study***	To explore patients' perspectives of the Gamma Knife stereotactic radiosurgery (GKSRS) process and the various stages involved.	Adult	Mixed intracranial conditions	Canada	Qualitative	Not reported	Patients	29	Interviews (open, unstructured, in-depth, semi-structured, open ended)	Grounded theory analysis/constant comparison/open, axial and/or selective coding	Bernstein, Potvin, Martin (2004)	Not reported	Seven overarching themes emerged from the data: (1) patients were satisfied with the overall treatment experience; (2) the majority of patients had a good knowledge of GKSRS; (3) the quality and amount of patient education were adequate; (4) process expectations were largely met; (5) most patients prioritized outcome over process; (6) most patients had a realistic expectation of outcomes; and (7) pain and anxiety were important issues.
Gunaratnam and Bernstein	Mixed	2016	Canada	Not reported	Division of Neurosurgery; University Health Network	All HIC	British Journal of Neurosurgery	***Patients views on priority setting in neurosurgery: A qualitative study***	To examine neurosurgical patients' views on the prioritization of patients for operating theater (OT) time on a daily basis at a tertiary and quaternary referral neurosurgery center.	Adult	Mixed	Canada	Qualitative	Not reported	Patients	37	Interviews (open, unstructured, in-depth, semi-structured, open ended)	Thematic analysis	Strauss and Corbin (1998)	Not reported	Overall, patients are supportive of the concept of a priority-setting system based on fairness, but felt that a few changes would help to improve the fairness of the current system. These changes include lowering the level of priority given to volume-funded cases and providing scheduled surgeries that were previously canceled a higher level of prioritization. Good communication, early notification, and rescheduling canceled surgeries as soon as possible were important factors that directly reflected the patients' confidence level in their doctor, the hospital, and the health care system.
Haag, Caringal, Sokoloff, et al.	Mixed	2016	Canada	Social worker	Lyle S. Hallman Faculty of Social Work	All HIC	Archives of Physical Medicine and Rehabilitation	***Being a woman with acquired brain injury: Challenges and implications for practice***	To explore the experiences of women with acquired brain injury (ABI) to gain greater insight into their general and sex- and gender-specific health and well-being concerns, and to identify areas for future research.	Adult	Mixed ABI	Canada	Qualitative; (Interpretive descriptive methodology)	Constructivist and naturalistic	Patients AND Family members, carers, signifcant others	16	Focus groups	Thematic analysis	Denzin & Lincoln (1998)	Not reported	Participants identified significant barriers to achieving optimal health and well-being for women survivors of ABI, including a lack of knowledgeable professionals. We identify 3 interrelated themes: (1) experiences shaped by gender norms and roles; (2) experiences influenced by physiological phenomena, including perceived hormone imbalances; and (3) experiences surrounding interpersonal relationships and sexuality.
Jadavji-Mithani, Venkatraghavan and Bernstein	Mixed	2015	Canada	Not reported	Division of Neurosurgery	All HIC	Canadian Journal of Neurological Sciences	***Music is beneficial for awake craniotomy patients: A qualitative study***	to better understand the overall effect of music on awake craniotomy patients, we also explored the impact of tonal structure (i.e. the influence of key change) in music on patients' mood states.	Adult	Brain tumor; Epilepsy	Canada	Qualitative	Not reported	Patients	29	Mixed: Questionnaires AND open-ended interviews	Grounded theory analysis/constant comparison/open, axial and/or selective coding (modified thematic analysis)	Corbin & Strauss (2008)	Not reported	Overall, patients enjoyed the music regardless of the key distinctions and stated they benefitted from listening to the music. No adverse reactions to the music were found. Subjects remarked that the music made them feel more at ease and less anxious before, during and after their procedure. Patients preferred either major key or minor key music but not a combination of both. Those who preferred major key pieces said it was on the basis of tonality while the individuals who selected minor key pieces stated that tempo of the music was the primary factor.

Karpa, Chernomas, Roger, et al.	Mixed	2020	Canada	Not reported	University of Brandon	All HIC	Nursing Research and Practice	***Families' Experiences Living with Acquired Brain Injury: "thinking Family" - A Nursing Pathway for Family-Centered Care***	To examine families' experiences living with acquired brain injury (ABI) using a research approach that included both the affected individual family member and the family together as a family group.	Adult	Mixed ABI	Canada	Narrative inquiry	Not reported	Patients AND Family members, carers, signifcant others	24	Mixed: Interviews (open, in-depth, semi-structured, open ended) AND ethnographic methods including: family genogram; family group observation sociogram; family ecomaps; and field notes.	Narrative analysis	Boss and Carnes (2012) Riessman (2008)	Not reported	Centered on the life stages of before the ABI event, now living with the ABI, and the future, thematic findings included: Families, a grounding force; Losses, individual and family; Family adaptive capacities; Experiences with the healthcare system-hospital to home; and A patchwork future-entering the unknown. Themes affirmed the significant impacts of ABI on individual and family members and acknowledged ABI as an ambiguous loss event.
Khabarov, Dimitropoulos and McGillicuddy	Mixed	2015	Canada	Social worker	University Health Network	All HIC	Health and Social Work	***Qualitative study: Exploring the experiences of family caregivers within an inpatient neurology and neurosurgery hospital setting***	The aim of this study was to further understanding of what it means for family caregivers to be included in their relatives' care and identify what type of care they are providing.	Adult	Neurological/ neurosurgical pathology non specific	Canada	Phenomenology	Not reported	Family members, carers, signifcant others	12	Interviews (open, unstructured, in-depth, semi-structured, open ended)	Thematic analysis	Hycner (1985)	Not reported	Upon review, the results indicated that the participants shared common experiences that were grouped into three main themes: (1) unfamiliarity with the hospital environment and procedures, (2) identifying the hidden realities of families and caregivers, and (3) strengthening collaborative dialogues and opportunities.
Knifed, July and Bernstein	Mixed	2008	Canada	Not reported	Division of Neurosurgery, Toronto Western Hospital, University Health Network	All HIC	Journal of Neurosurgery	***Neurosurgery patients' feelings about the role of residents in their care: A qualitative case study***	To explore the level of knowledge and anxiety in patients regarding residents' involvement in their surgery.	Adult	Mixed: brain tumor, spinal tumor, disc herniation	Canada	Case study	Not reported	Patients	30	Interviews (open, unstructured, in-depth, semi-structured, open ended)	Thematic analysis (modified)	Bernstein (2004)	Not reported	Six prominent themes arose from the analysis: 1) the level of knowledge about residents is low; 2) the level of anxiety about residents is low; 3) it is desirable for patients to meet the residents before surgery; 4) residents' educational needs are understood and supported; 5) anxiety was not increased by the interview; and 6) patients trust in the medical system.
Lindsay, Proulx, Maxwell, et al.	Mixed	2016	Canada	Not reported	Bloorview research institute	All HIC	Archives of Physical Medicine and Rehabilitation	***Gender and transition from pediatric to adult health care among youth with acquired brain injury: Experiences in a transition model***	To explore gender and sex differences in experiences of transitioning to adult health care among young adults with acquired brain injury (ABI) who take part in a coordinated model of transitional care.	Adult	Mixed ABI	Canada	Qualitative (descriptive)	Not reported	Patients AND Family members, carers, signifcant others	18	Interviews (open, unstructured, in-depth, semi-structured, open ended)	Grounded theory analysis/constant comparison/open and axial/selective coding	Corbin & Strauss (2008)	Not reported	Our findings highlight several commonalities and differences relative to sex and gender among young adults with ABI who are transitioning from pediatric to adult care. Both young adult men and women experienced a similar transition process and similar organization, continuity, and availability of care. Sex differences were found in relational factors (e.g., communication, family involvement, social support). Young adult men, and parents of the men, differed in their transition regarding relational factors (e.g., communication, family involvement).
Martin, Singer and Bernstein	Mixed	2003	Canada	Not reported	Department of Health Policy, Management and Evaluation	All HIC	Journal of Neurology, Neurosurgery and Psychiatry	***Access to intensive care unit beds for neurosurgery patients: A qualitative case study***	To describe the process used to decide which patients are admitted to the intensive care unit (ICU) at a hospital with special focus on access for neurosurgery patients, and evaluate it using "accountability for reasonableness".	Adult	Neurosurgical critical care	Canada	Case study	"accountability for reasonableness" - conceptual framework	HCPs (inc. NSx) AND Non-clinical providers	13	Mixed: Documentary sources AND Observations AND Interviews	Not clearly stated	Not reported	Not reported	ICU admissions were based on the referring physician's assessment of the medical need of the patient for an ICU bed. Non-medical criteria (for example, family wishes) also influenced admission decisions. Although there was an ICU bed allocation policy, patient need always superseded the bed allocation policy. ICU admission guidelines were not used. Admission decisions and reasons were disseminated to the ICU charge nurse, the bed coordinator, the ICU resident, the intensivist, and the requesting physician/surgeon by word of mouth and by written documentation in the patient's chart, but not to the patient or family. Appeals occurred informally, through negotiations between clinicians. Enforcement of relevance, publicity, and appeals was felt to be either non-existent or deficient.
Panday, Velikonja, Moll, et al.	Mixed	2021	Canada	Graduate student researcher	School of Rehabilitation Science	All HIC	Disability and Rehabilitation	***Experiences of inpatient rehabilitation from the perspective of persons with acquired brain injury***	To explore the experiences, needs, and preferences of patients from an ABI inpatient rehabilitation program in Ontario.	Adult	Mixed ABI	Canada	Qualitative	values understanding subjective experiences within a particular context	Patients	12	Interviews (open, unstructured, in-depth, semi-structured, open ended)	Iterative, inductive approach	Thorne (2016) and Thorne, Reimer-Kirkham and MacDonald-Emes (2003)	Not reported	We identified three major themes: (1) Life Rerouted - participants felt their lives diverted due to ABI, with rehabilitation seen as a way to return to pre-injury life, (2) Autonomy within Rehab highlighted the perceived importance of personal autonomy in decision-making within rehabilitation, and (3) Life (and Recovery) Go On reflected an ongoing recovery process after discharge - leading to mixed emotions. An overall message, "re-establishing personal identity is important to the recovery process," reflected theories of biographical disruption and relational autonomy.
Paniccia, Colquhoun, Kirsh, et al.	Mixed	2019	Canada	Not reported	Rehabilitation Science Institute, Bloorview Research Institute	All HIC	Disability and Rehabilitation	***Youth and young adults with acquired brain injury transition towards work-related roles: a qualitative study***	To explore the experiences of youth and young adults with acquired brain injury as they transition towards work-related roles.	Mixed	Mixed ABI	Canada	Qualitative (descriptive)	Not reported	Patients	14	Interviews (open, unstructured, in-depth, semi-structured, open ended)	Grounded theory analysis/constant comparison/open, axial and/or selective coding (Thematic)	Braun & Clarke (2014), Boeije (2002)	Not reported	Three major themes emerged related to the experience of work-related roles: (1) getting to know the new me; (2) navigating support systems; and (3) taking control of my experience. Some participants used coping strategies to acquire and/or maintain work-related roles, while others felt limited by their condition.
Rashid, Goez, Caine, et al.	Mixed	2016	Canada	Not reported	Faculty of Rehabilitation Medicine	All HIC	Journal of Pediatric Rehabilitation Medicine	***After a child's acquired brain injury (ABI): An ethnographic study of being a parent***	To explore how parental meanings, roles, and expectations are constructed from parent-child interactions in the day-to-day lives of families with a child with an ABI.	Pediatric	Mixed ABI	Canada	Ethnography	Symbolic interactionism	Family members, carers, signifcant others	10	Mixed: Interviews (open, unstructured, in-depth, semi-structured, open ended) AND observation AND field notes	Thematic analysis	Braun & Clarke (2006)	Not reported	6 themes: Getting back to normal, relying on a support system, worrying something bad may happen after the injury, going through a range of emotions following the injury, changing family dynamics after the injury, ongoing performativity. Highlighted the key importance of having a strong familial support system. Compared to many other studies, this study found that the parents did not experience extensive role changes as parents.

Samuel, Shamji and Bernstein	Mixed	2016	Canada	Not reported	Faculty of Medicine and Division of Neurosurgery	All HIC	Journal of Neurosurgery	***Neurosurgical patients' perceptions of the "surgeon+": A qualitative study***	The aim of the present study was to qualitatively examine patient values and preferences for a neurosurgeon who participates in extra clinical activities and understand the factors that influence these perceptions.	Adult	Ambulatory patients from neurosurgeons practice. Pre/ post op	Canada	Qualitative	Not reported	Patients AND Family members, carers, signifcant others	47	Interviews (open, unstructured, in-depth, semi-structured, open ended)	Grounded theory analysis/constant comparison/open, axial and/or selective coding (Thematic)	Corbin & Strauss (2008)	Not reported	Patients generally indicated that they want to feel confident in their neurosurgeon. A number of factors contribute to this confidence, including a strong positive referral from another physician. Patients are inclined to search for information pertaining to the qualifications of neurosurgeons online, and a perception of the neurosurgeon's adeptness given his or her qualifications is important for patients. Although there were some differences in patient values between those in the cranial and spinal groups, overall, neurosurgical patients tend to positively view their neurosurgeon's involvement in extra clinical duties.
Veilleux, Samuel, Yan, et al.	Mixed	2021	Canada	MD/Physician	Division of Neurosurgery, Department of Clinical Neurosciences	All HIC	Neurosurgical Focus	***Cross-sectional analysis of women in neurosurgery: a Canadian perspective***	To analyze the experiences of women faculty practicing neurosurgery across Canada to better understand and address the factors contributing to this disparity.	N/A	Neurosurgery	Canada	Survey	Not reported	NSx	19	Open ended questions	Grounded theory analysis/constant comparison/open, axial and/or selective coding (modified thematic)	Not reported	Not reported	Although the past decades have seen a steady increase of women in medicine in general, women continue to represent a minority of the physician-training staff and workforce in neurosurgery in Canada and worldwide. As such, the aim of this study was to analyze the experiences of women faculty practicing neurosurgery across Canada to better understand and address the factors contributing to this disparity.
Vogel, Kleib, Davidson, et al.	Mixed	2016	Canada	RN/Nurse	Faculty of Nursing,	All HIC	JMIR Research Protocols	***Parental Evaluation of a Nurse Practitioner-Developed Pediatric Neurosurgery Website***	To assess and evaluate whether a custom-designed health website could meet parents' health information, support, and resource needs.	Pediatric	Pediatric neurosurgical problem	Canada	Mixed methods	Not reported	Family members, carers, signifcant others	52	Mixed: Google analytics; questionnaires AND focus groups	Content analysis: Inductive	Shuyler & Knight (2003); Krueger & Casey (2008)	Not reported	There were a total of 2998 sessions and 8818 page views, with 2.94 pages viewed per session, a 56.20% bounce rate, an average session duration of 2 minutes 24 seconds, and a 56.24% new sessions rate. Results from 52 eligible surveys included that the majority of NKF users were Caucasian (90%), females (92%), aged 36-45 years (48%), with a university or college degree or diploma (69%). Half plan to use the health information. Over half reported turning to the Internet for health information and spending 2 to 4 hours a day online. The most common reasons for using the NKF website were to (1) gather information about the 2 summer camps, (2) explore the Media Center tab, and (3) stay abreast of news and events supported by NKF. Parents were unanimous in reporting that the NKF website was pleasing in color and design, very easy to use and navigate, useful, and that they would continue to access it regularly.
Wright, Medved, Woodgate, et al.	Mixed	2016	Canada	Clinical psychology student	Department of Psychology,	All HIC	Journal of Communication in Healthcare	***Narratives of acquired brain injury patients: Their experience of healthcare relationships and medical decision-making***	To obtain a better understanding of how mild to moderate ABI patients in the chronic phase of recovery	Adult	Mixed ABI	Canada	Qualitative	Not reported	Patients	11	Interviews (open, unstructured, in-depth, semi-structured, open ended)	Narrative analysis (idiographic)	Hydén & Brockmeier (2008); Gabriel (2004); Riley (2005)	Not reported	These participants told one coherent narrative with two main storylines: one in which they positively portrayed their doctors and their healthcare relationships - reporting feeling lost and needing their doctor's help; and another in which they negatively portrayed their doctors and their healthcare relationships - reporting being capable and therefore not needing their doctor's help. Although seemingly contradictory, these two storylines speak to one coherent experience in which capability served as a counter-narrative to what they perceived as a global narrative of being devalued, dismissed and patronized. Discussion: The implication of how this counternarrative affects their experience of healthcare is discussed, including implications for why doctors may want to focus on fostering a positive doctor-patient relationship by conveying that they care for and value their ABI patients, and why simple interactions may contribute to this positive relationship.
Zener and Bernstein	Mixed	2011	Canada	Not reported	Department of Surgery, Division of Neurosurgery,	All HIC	Canadian Journal of Neurological Sciences	***Gender, patient comfort and the neurosurgical operating room***	To gain insight into patients' perspective of the OR environment, including staffing and observers, the role of medical students, catheterization, exposure, and verbiage, using a qualitative needs assessment.	Adult	Craniotomy; discectomy; spinal tumor resection	Canada	Case study	Not reported	Patients	20	Interviews (open, unstructured, in-depth, semi-structured, open ended)	Grounded theory analysis/constant comparison/open, axial and/or selective coding (Thematic)	Palese et al (2008)	Not reported	Nine themes emerged: 1) perception of the intra-operative environment varies between men and women; 2) lacking awareness about observers is anxiety-provoking for women; 3) being unaware of the hands-on involvement of students is a concern for all patients; 4) disclosure of implantation of foreign and permanent materials into patients is important; 5) catheterization is anxiety provoking for women; 6) pre-operative menstruation screening may minimize embarrassment for women; 7) patients perceive extraneous conversation as a distraction for surgeons; 8) patients trust their surgeon; 9) a relationship exists between interviewer gender and patient comfort in the interview.
Peterson, Webster, Gonzalez-Lara et al.	Mixed	2021	USA	Not reported	Institute for Philosophy and Public Policy,	All HIC	BMC Medical Ethics	***Caregiver reactions to neuroimaging evidence of covert consciousness in patients with severe brain injury: a qualitative interview study***	1) What are caregivers expectations for enrolling in neuroimaging research?; 2) How do caregivers react to evidence of covert consciousness?; 3) How do caregivers react to uninformative neuroimaging results? and 4) Do caregivers generally understand the research and results?	Unclear	Severe brain injury; PDoC	Canada	Qualitative (descriptive)	Not reported	Family members, carers, signifcant others	12	Interviews (open, unstructured, in-depth, semi-structured, open ended)	Grounded theory analysis/constant comparison/open and axial/selective coding (Descriptive)	Not reported	Not reported	Twelve caregivers participated in the study; two caregivers shared surrogate decision-making status for one patient with PDoC. Twenty-one interviews were completed; one caregiver declined to participate in the post-disclosure interview. Three patients with PDoC associated with the study displayed evidence of covert consciousness. Overall, caregivers understood the neuroimaging research and results. Caregivers who received results of covert consciousness were generally pleased. However, there was some variation in expectations and reactions to these data and null results.
Botting, Phan, Rubenfeld, et al.	Mixed	2014	Canada	Not reported	Department of Critical Care Medicine	All HIC	Neurocritical care	***Using barriers analysis to refine a novel model of neurocritical care***	To understand the facilitators and barriers to this change as we developed our virtual neuro critical care model.	Adult	Neuro Critical Care	Canada	Mixed methods	Not reported	HCPs (inc. NSx) AND non-clinical providers	54	Mixed: Interviews (open, unstructured, in-depth, semi-structured, open ended) AND focus groups AND questionnaires	Content analysis	Not reported	Not reported	Seventeen barriers were proposed through an open-ended survey question. Content analysis revealed general resistance, educational challenges, workflow adjustment to a diagnosis-based rounding pattern and coordination conflicts to be the central barriers. These findings were confirmed in focus group discussions, with a lack of resources as an additional important challenge.
Gan, Gargaro, Brandys, et al.	Mixed	2010	Canada	Not reported	Bloorview Kids Rehab	All HIC	NeuroRehabilitation	***Family caregivers' support needs after brain injury: A synthesis of perspectives from caregivers, programs, and researchers***	To broaden the understanding of ABI caregiver support needs through data triangulation from multiple interview sources across different settings	Unclear	Mixed ABI (inc. trauma, medical problems or disease)	Canada and USA	Qualitative	Not reported	Family members, carers, signifcant others AND Researchers AND Non-clinical providers	55	Mixed: Focus groups AND interviews	Constant comparative method AND content analysis	Chamberlain, Camic & Yardley (2004), Strauss & Corbin (1984), Wilkinson, Joffe & Yardley (2004)	Not reported	Perspectives from those involved in receiving, providing and researching caregiver interventions following ABI were synthesized to provide a thorough, detailed depiction of the ongoing support needs of caregivers. This convergence of evidence underscores that caregiver support needs transcend geographical boundaries and must be comprehensive, accessible, long-term, and encompass education, emotional, and instrumental support

Samuel, N. and McQueen, S. A. and Barnett, R. R. and Everson, M. C. and Fiala, C. and Lau, R. and Zadeh, G.	Mixed	2023	Canada	Neurosurgeon	Department of Neurosurgery	All HIC	Journal of Surgical Education	***Parenting and Childbearing in Neurosurgical Residency: Perspectives from the United States and Canada***	To investigate residents' perspectives surrounding parenting and childbearing during neurosurgical residency in the United States and Canada.	Unclear	N/A	Canada/USA	Qualitative	Not explicitly stated	Neurosurgeons (inc. trainees/residents)	12	Focus groups	Grounded theory analysis	Charmaz, 2014	Not reported	Notable challenges included lack of formal family leave policies, time constraints, insufficient clinical human resources, physical health concerns, lack of lactation accommodations, and lack of mentorship. A subset of barriers were uncovered that stem specifically from workplace cultures, including gender norms, difficulty in asking for help, concerns for inconveniencing others, and pressures to time parental leave during research blocks. Several positive changes were identified including growing awareness and female representation, and benefits of the dual surgeon-parent identity
Xuan, G. and Juan, D. and Xurui, Z. and Fei, L.	Mixed	2024	China	Nursing graduate student	Department of Neurosurgery	All LMIC	Nursing Open	***Real emotional experience of family members of patients transported within hospital in neurosurgical intensive care unit: A descriptive qualitative study***	To understand the real experience of family members of patients in neurosurgical intensive care unit (NICU) during intra-hospital transport (IHT), explore their inner needs and provide effective intervention measures for the construction of standardized IHT plan.	Unclear	Mixed	China	Qualitative descriptive	Not explicitly stated	Family/Carer/Significant other	10	Interviews (semi-structured, open, in depth)	Phenomenological analysis (Colaizzi 7-step analysis)	Sanders (2003)	Not reported	A total of three themes and nine subthemes were extracted, namely: Experience of emotional changes at different stages (uncertainty before transfer, complex internal activity during transit, ambivalence after transfer); Perception of problems in IHT (poor doctor-patient communication, weak awareness of risk assessment, deficiencies in the transfer procedure); Consciousness of the real needs (emotional respect and closeness, stay informed of the progression of the disease, greater social support).
Liu, Liu, Wang, et al.	Mixed	2019	China	MD/Physician	Department of Neurosurgery	All LMIC	BMJ Open	***Neurosurgical enhanced recovery after surgery (ERAS) programme for elective craniotomies: Are patients satisfied with their experiences? A quantitative and qualitative analysis***	To evaluate patient satisfaction and associated predictors at discharge, as well as patient experience at 30-day follow-up, in a neurosurgical enhanced recovery after surgery (ERAS) programme.	Adult	Mixed ABI	China	Mixed methods (RCT & IPA)	Not reported	Patients	46	Mixed: Patient outcomes;AND questionnaires AND Interviews (open, unstructured, in-depth, semi-structured, open ended)	IPA	Smith et al (2009)	Not reported	Analysis on patient experience revealed five themes: information transfer, professional support, shared responsibility and active participation, readiness for discharge, and follow-up, all of which are closely related and represent positive and negative aspects.
Zhan, Yu, Chen, et al.	Mixed	2022	China	RN/Nurse	Department of Nursing,	All LMIC	Journal of Nursing Management	***Family caregivers' experiences and needs of transitional care during the transfer from intensive care unit to a general ward: A qualitative study***	To explore the family caregivers' experiences and needs of transitional care during the transfer from an intensive care unit to a general ward in China.	Adult	Mixed	China	Phenomenology	Phenomenological	Family members, carers, signifcant others	15	Interviews (open, unstructured, in-depth, semi-structured, open ended)	Phenomenological analysis	Colaizzi (1978)	SRQR	Based on data analysis, four themes were obtained: perception of transfer decision, the experience of transitional care, the obstacles to maintaining care efficiency and demand for transitional care. In order to enhance the continuity of care and improve patient safety during the transfer from an ICU to a general ward in China, priorities should be given to the implementation of effective strategies and methods, including providing psychological and emotional support, encouraging active participation of caregivers, and various communication and collaboration procedures.
Loft, M. I. and Guldager, R. and Poulsen, I.	Mixed	2022	Denmark	Other: Head of Nursing Research	Department of Neurology, Rigshospitalet; Research Unit for Nursing and Healthcare, Dept. of Public Health	All HIC	Journal of Research in Nursing	***Caring from a distance: how a COVID-19 visitor ban affects relatives when a loved one is admitted to a neurological or neurosurgical ward***	to investigate how relatives of neurological patients experienced the visitor ban and to identify potential areas for improvement.	Adult	Mixed	Denmark	Qualitative	Not explicitly stated	Family/Carer/Significant other	12	Interviews (semi-structured, open, in depth)	Reflexive thematic analysis	Braun and Clarke (2021)	Not reported	The following six themes emerged: Visitor ban as a necessary evil, Losing control and feeling checkmate, Mending the information gap, Waiting by the phone, Empathy and compassion as the core of a good relationship and Caring for a loved one from a distance.
Wolffbrandt, M. M. and Soendergaard, P. L. and Biering-Sorensen, F. and Sundekilde, L. and Kjeldgaard, A. and Schow, T. and Arango-Lasprilla, J. C. and Norup, A.	Mixed	2024	Denmark	Unclear/not stated	Neurorehabilitation Research and Knowledge Centre, Rigshospitalet	All HIC	Disability and Rehabilitation	***A manual-based family intervention for families living with acquired brain or spinal cord injury: a qualitative study of families' experiences***	To explore families' experiences of participating in a family intervention, targeting families living with the consequences of acquired brain injury (ABI) or spinal cord injury (SCI).	Adult	ABI and SCI	Denmark	Qualitative	Not explicitly stated	Patients AND Families/carers/Significant other	33	Interviews (semi-structured, open, in depth)	Reflexive thematic analysis	Braun and Clarke (2006), Braun and Clarke (2019), Braun and Clarke (2021)	Not reported	One central theme was developed "A sense of belonging together again", describing the value of the reciprocal format of the family intervention, where individuals with ABI or SCI and their family members gained new insights into each other while building up their relationship. The central theme was supported by three additional themes: "Strengthened communication and emotional control", "Acknowledging the changed life situation" and "Being seen as a whole person".
Kieffer-Kristensen and Johansen	Mixed	2013	Denmark	Child psychologist	Department of Psychology	All HIC	Brain Injury	***Hidden loss: A qualitative explorative study of children living with a parent with acquired brain injury***	To listen to and learn from children showing high levels of post-traumatic stress symptoms after parental acquired brain injury (ABI), in order to achieve an in-depth understanding of the difficulties the children face in their everyday lives and identify possible trauma-related feelings and relational losses.	Adult	Mixed ABI	Denmark	Phenomenology	Phenomenology	Family members, carers, signifcant others	14	Interviews (open, unstructured, in-depth, semi-structured, open ended)	Phenomenological analysis ('categorization of meanings')	Camic, Rhodes & Yardley (2004); Kvale & Brinkman (2009)	Not reported	All children were affected by their parents' ABI and the altered family situation. The children's expressions led the authors to identify six themes, including fear of losing the parent, distress and estrangement, chores and responsibilities, hidden loss, coping and support. The main finding indicates that the children experienced numerous losses, many of which were often suppressed or neglected by the children to protect the ill parents.
Kjaersgaard and Kristensen	Mixed	2017	Denmark	Not reported	Hammel Neurorehabilitation Center and University Research Clinic	All HIC	Brain Sciences	***Brain injury and severe eating difficulties at admission" "patient perspective nine to fifteen months after discharge: A pilot study***	To explore and interpret the way that individuals with acquired brain injury, admitted to inpatient neurorehabilitation with severe eating difficulties, experienced eating nine to fifteen months after discharge	Adult	Mixed ABI	Denmark	Qualitative	Not reported	Patients	4	Interviews (open, unstructured, in-depth, semi-structured, open ended)	Content analysis	Graneheim & Lundman (2004), Elo & Kyngäs (2008)	Not reported	Four main themes emerged from the analysis: personal values related to eating, swallowing difficulties, eating and drinking, meals and social life. Three predominating experiences were: fed by tube, "relearning" to eat, and eating meals together. The preliminary results regarding the four participants suggest that the meaning of food and being able to eat and take part in meals may be nearly the same as before the injury; however, having the ability to eat reduced or lost completely, even temporarily, was unexpected and difficult, and caused strong emotional reactions, even 18 months after injury. Time spent using a feeding tube had a negative, but not persistent, impact on quality-of-life.
ThØgersen and Glintborg	Mixed	2022	Denmark	Psychologist	Center for Developmental and Applied Psychological Science (CeDAPS), Department of Communication and Psychology	All HIC	Nordic Psychology	***Ambiguous loss and disenfranchised grief among spouses of brain injury survivors***	To explore the experience of grief among caregiving partners	Adult	Mixed ABI	Denmark	Phenomenology	phenomenological life-world perspective	Family members, carers, signifcant others	4	Interviews (open, unstructured, in-depth, semi-structured, open ended)	Phenomenological descriptive analysis	Giorgi & Giorgi (2004)	Not reported	Through the analysis, nine themes were identified: the process of becoming a caregiver, grief emotions, loss, emotionally closer relations to the partner relations, dyadic coping, unmet needs for support, future opportunities, changes over the life course, and understanding of grief. The themes are interrelated and highlight various aspects of the phenomenon of grief as experienced by caregivers. Results reveal that grief is often a reaction to ambiguous loss and is experienced as disenfranchised. Furthermore, the study indicates that caregivers' perception of grief and the situation in general were essential to the experience of grief.

Bramall, Djimbaye, Tolessa, et al.	Mixed	2014	Canada	Not reported	Division of Neurosurgery	Mixed HIC/LMIC	World Neurosurgery	***Attitudes toward neurosurgery in a low-income country: A qualitative study***	To examine the perspectives of neurosurgery patients in a low-income country with limited health care resources.	Unclear	Mixed neurosurgical conditions (ranging from back pain to intracranial tumors)	Ethiopia	Qualitative	Not reported	Patients	25	Interviews (open, unstructured, in-depth, semi-structured, open ended)	Grounded theory analysis/constant comparison/open, axial and/or selective coding	Strauss & Corbin (1998)	Not reported	The following 5 themes emerged: 1) With limited resources, many patients did not seek information outside of that obtained during the clinical encounter. 2)Patients valued direct verbal communication and deferred to the surgeon's authority. 3) Religion played an instrumental role in patient attitudes toward surgery. 4) Most patients did not feel anxious about surgery. 5) A few patients did not inform family members about their medical condition.
Debono, B. and Baumgarten, C. and Guillain, A. and Lonjon, N. and Hamel, O. and Moncany, A. H. and Magro, E.	Mixed	2023	France	Neurosurgeon	Department of Neurosurgery	All HIC	Brain and Spine	***Becoming a neurosurgeon in France: A qualitative study from the trainees' perspective***	Explored the experiences of French neurosurgical trainees and their comments on their educational system.	N/A	Education	FR	Grounded theory	Non stated	Neurosurgeons (inc. trainees/residents)	23	Interviews (semi-structured, open, in depth)	Thematic analysis (inductive)	Non stated	COREQ	Data analysis identified three superordinate themes: (1) The Trainee-Senior Dyad, where the respondents describe a similar bipolarity between trainees and faculty (trainees oscillating between those who fit into the system and those who are more reluctant to accept hierarchy, faculty using an ideal pedagogy while others refuse to help or invest in training); (2) The difficulty to learn (describing pressure exercised on trainees that can alter their motivation and degrade their training, including the impact of administrative tasks); (3) A pedagogy of empowerment (trainee' feelings about the pertinent pedagogy in the OR, ideal sequence to progress, progressive empowerment especially during the shifts, and stress of envisioning themselves as a senior neurosurgeon).
Lafiatoglou, P. and Ellis-Hill, C. and Gouva, M. and Ploumis, A. and Mantzoukas, S.	Mixed	2024	Greece	Other: health researcher	Department of Nursing, School of Health Sciences	All HIC	Journal of Clinical Nursing	***Older adults' lived experiences of physical rehabilitation for acquired brain injury and their perceptions of well-being: A qualitative phenomenological study***	To acquire an in-depth understanding of the lived experiences of older adults (65+) living with acquired brain injury who undergo or have undergone physical rehabilitation in relation to their sense of well-being during care provision within the Greek Healthcare System.	Adult	MIXED ABI	Greece	Phenomenology (interpretive)	Hermeneutics	Patients	14	Interviews (semi-structured, open, in depth)	Thematic analysis	Van Manen (1990); Braun and Clarke (2013)	COREQ	Four themes emerged from the analysis: (1) Challenges of new life situation, (2) Seeking emotional and practical support through social interaction, (3) Identifying contextual processes of rehabilitation, (4) Realising the new self.
Man	Mixed	2002	Hong Kong	Not reported	Department of Rehabilitation Sciences	N/A	International Journal of Rehabilitation Research	***Hong Kong family caregivers' stress and coping for people with brain injury***	To further explore the difficulties and problems families are facing in caring for people with brain injury. Family coping mechanisms in dealing with stress will be explored as well.	Unclear	Mixed ABI	Hong Kong	Mixed methods	Not reported	Family members, carers, signifcant others	50	Mixed: 1. Questionnaires; 2. Open interviews; 3. Long interviews	Content analysis	Not reported	Not reported	All the families' responses to questions, and the verbatim transcripts of long interviews of four randomly selected families, were used to construct themes of coping strategies. They were found to show the typical coping strategies of people facing stress, including shock and uncertainty, which were suggested to be related closely to the nature of brain injury, especially if the injury was traumatic in nature, and their difficulties in managing problems that were novel, unpredictable and global in nature (affecting physical and cognitive functions, personality change and social integration). The physical and psychological burdens involved in day-to-day caring for members with brain injury were unanimously reflected in the interviews. Further content analysis of the long interviews of four selected families showed that some families coped well and some did not. For those successful coping situations, families reported that they became empowered after the onset of their members' brain injury. They expressed possible factors leading to better adjustment, which included setting clear personal expectations, the flexibility to adjust life goals, a desire to master the situation, strong motivation, awareness of their own powerless state and willingness to ask for help from different sources.
Kilcoyne, Rogers, Thomas, et al.	Mixed	2021	UK	Speech/language therapist	Oxford Craniofacial Unit,	All HIC	The Journal of craniofacial surgery	***Craniofacial Surgery-Related Hashtag Utilization on Instagram***	To investigate the use of Instagram in relation to craniofacial surgery.	Mixed	Craniofacial surgery	International	Mixed methods (qualitative and quantitative data)	Not reported	Other	Other: 151,738 posts identified using 12 hashtags	Social Media content: Instagram hashtags	Thematic analysis	Braun (2006)	Not reported	A total number of 151,738 posts were identified using the 12 hashtags queried in the present study, with #craniofacial being the most popular hashtag (87% of posts). Parents (n1⁄425, 33%) and surgeons (n 1⁄4 25, 33%) were responsible for the majority of posts. A heterogenous group were responsible for the remaining 34% of posts. Results indicated that parents and surgeons posted significantly different images (chi-squared with Yates correlation is 25.0519, P<0.00001). Parents' posts were predominantly images of their child/patients (n 1⁄4 24) compared to posts about patients by surgeons (n 1⁄4 6). Posts by surgeons were predominantly images of professionals (n 1⁄4 14) compared to images of professionals posted by parents (n 1⁄4 1). Results of qualitative analysis of captions of posts indicated a significant difference in themes that emerged between parents and surgeons; with surgeons largely using Instagram for information giving, and parents for sharing of experiences (chi-square (n 1⁄4 108) 1⁄4 40.83, P < 0.00001). There was a significant difference (two-tailed t test, P 1⁄4 0.002) in engagement (measured by the number of likes on posts) on posts by parents (mean 1⁄4 3778) compared to posts by surgeons (mean 1⁄4 135.4)
Noohi, Peyrovi, Imani Goghary, et al.	Mixed	2016	Iran	Not reported	Physiology Research Center, Department of Medical Surgical Nursing Education, Razi School of Nursing and Midwifery	All LMIC	Consciousness and Cognition	***Perception of social support among family caregivers of vegetative patients: A qualitative study***	To explore the perception of social support among family caregivers of VS patients	Unclear	Severe brain damage	Iran	Qualitative	Not reported	Family members, carers, signifcant others AND HCPs	12	Interviews (open, unstructured, in-depth, semi-structured, open ended)	Grounded theory analysis/constant comparison/open and axial/selective coding	Corbin & Strauss (2008)	Not reported	The four categories of "Family, a supporter in all aspects," "Beautiful emanation of the nurse's role," "Revitalization via empathy and companionship," and "Defects in support," were extracted. The primary concern of participants was receiving social support which can facilitate caregiving and coping with difficulties, but there are many shortcomings in supporting these caregivers.
Rafiei, Arab, Rashidian, et al.	Mixed	2015	Iran	Not reported	Managment and health economics	All LMIC	Global journal of health science	***Factors Influencing Neurosurgeons' Decision to Retain in a Work Location: A Qualitative Study***	To determine the affecting factors from neurosurgeons' viewpoint to support policy makers in proposing a sort of evidence based retention strategies with the purpose of encouraging neurosurgeons to continue serving in their current work location even those working in remote and underserved areas.	N/A	Neurosurgery	Iran	Qualitative	Not reported	NSx	17	Interviews (open, unstructured, in-depth, semi-structured, open ended)	Framework analysis (inductive AND deductive)	Richie & Spencer (1994) Frameowrk approach	Not reported	Satisfaction with monetary incentives, availability of adequate clinical infrastructure in a community and appropriate working condition were most commonly cited factors mentioned by all participants as key reasons for retention. Furthermore elements which contributed to the quality of living condition, personal background and incentives, family convenience were emphasized by majority of them. A small number of participants mentioned opportunity for continuing learning and updating knowledge as well as supportive organizational policies as important motivators in a workplace.

Segev, Levinger and Hochman	Mixed	2018	Israel	Not reported	Sapir Academic College,	All HIC	Qualitative Health Research	***"Shared Destiny": The Dynamics of Relationships in Families of Patients With Brain Injury***	To explore the dynamics between caregivers of the family member with a brain injury during rehabilitation hospitalization, and the relationships between them and the rest of the extended family.	Adult	Mixed ABI	Israel	Phenomenology	Not reported	Family members, carers, signifcant others	20	Interviews (open, unstructured, in-depth, semi-structured, open ended)	Thematic analysis	Braun & Clarke (2006), Elo & Kyngas (2008), Hsieh & Shannon (2005)	Not reported	The importance of the relationships between family members during rehabilitation hospitalization justifies the examination undertaken in this research. Findings point at the change that took place in the relationships between family members because of the need to cope with a relative's injury. It is possible that direct intervention in the dynamics of the relationship, especially between the family of origin and the nuclear family of the injured person, can benefit extended families in coping with the crisis
Yehene, Steinberg, Gerner, et al.	Mixed	2021	Israel	Psychologist	The Academic College of Tel Aviv-Yaffo	All HIC	Qualitative Health Research	***"Concurrent Ropes and Ladders": Mapping and Conceptualizing the Emotional Loss Experience of Parents Following Pediatric Acquired Brain Injury***	To map, conceptualize, and theorize the emotional loss experienced by parents following their child's pediatric acquired brain injury (pABI)	Pediatric	Mixed acquired brain injury (CVA, tumor, brain disease, TBI)	Israel	Grounded theory	Constructivist	Family members, carers, signifcant others	47	Interviews (open, unstructured, in-depth, semi-structured, open ended)	Grounded theory analysis/constant comparison/open and axial/selective coding	Charmaz (2006)	Not reported	Codes were consolidated into five thematic categories capturing parents' emotional continuous loss experience: (a) comparing life before and after, (b) struggling to construct new realities, (c) recognizing instability and permanency, (d) adjusting and readjusting, and (e) grieving as an emotional shadow. These categories are at work simultaneously in parents' accounts, thus supporting a model of dynamic concurrency within and across their lived experiences.
Ueki, S. and Kaneda, Y. and Ozaki, A. and Kotera, Y. and Tanimoto, T. and Omoto, Y. and Kurosaki, K. and Yamazaki, H. and Yoshida, T. and Mizoue, N. and Yoshimura, H. and Hayashi, Y. and Shimamura, Y.	Mixed	2024	Japan	Medical student	Hiroshima University School of Medicine; Medical Network Systems Inc	All HIC	SN Comprehensive Clinical Medicine	***Exploring the Landscape of Home-Based Teleradiology in Japan: A Qualitative Analysis of Radiologists' and Neurosurgeons' Experiences to Elucidate Advantages, Challenges, and Future Directions***	to investigate the advantages, disadvantages, working methods, and support needs of physicians practicing teleradiology from home in Japan, as well as to explore challenges related to the COVID-19 pandemic.	Unclear	Mixed	Japan	Qualitative	Interpretivist	HCPs (Inc NSx)	15	Interviews (semi-structured, open, in depth)	Thematic analysis	Braun and Clark (2006)	Not reported	Five main themes were generated: (1) the ability to work from home according to one's lifestyle, allowing for better work-life balance; (2) reduced quality and efficiency of reading work, due to limited patient information and lack of immediate consultation; (3) self-management and reliance on available resources, including literature searches and networking with colleagues; (4) need for comfortable infrastructure facilities, such as high-quality monitors and stable internet connections; and (5) provision of resources to resolve questions and concerns, including platforms for peer discussions. Interestingly, no specific themes related to the COVID-19 pandemic were identified, suggesting that the challenges of teleradiology were not uniquely affected by the pandemic. While home-based teleradiology offers lifestyle flexibility, it presents challenges in maintaining work quality and efficiency. Physicians employ self-management strategies and rely on available resources to mitigate these challenges. The findings highlight the need for improved infrastructure and support systems to enhance the practice of home-based teleradiology in Japan. This study contributes to the growing body of literature on remote healthcare delivery and may inform policy decisions and best practices in diagnostic radiology both in Japan and internationally.
Whiffin, Smith, Esene, et al.	Mixed	2021	UK	RN/Nurse	NIHR Global Health Research Group on Neurotrauma; College of Health Psychology and Social Care	Mixed HIC/LMIC	BMJ Open	***Neurosurgeons' experiences of conducting and disseminating clinical research in low-income and middle-income countries: A reflexive thematic analysis***	To understand neurosurgeons' experiences of, aspirations for, and ability to conduct and disseminate clinical research in LMICs.	N/A	Mixed	Mixed	Qualitative	Naturalistic paradigm, interpretivist, inductive	NSx	26	Mixed: Focus groups AND semi-structured interviews	Reflexive thematic analysis	Braun & Clarke (2021)	COREQ	The analysis gave rise to five themes: The local landscape; creating capacity; reach and impact; collaborative inquiry; growth and sustainability. Each theme contained an inhibitor and stimulus to neurosurgeons conducting and disseminating clinical research, interpreted as 'the neurosurgical research potential in LMICs'. Mentorship, education, infrastructure, impact and engagement were identified as specific accelerators. Whereas lack of generalizability, absence of dissemination and dissemination without peer review may desensitize the impact of research conducted by neurosurgeons.
Samuel, Alotaibi and Lozano	Mixed	2017	Canada	MD/Physician	MD/PhD Program, Faculty of Medicine	All HIC	World Neurosurgery	***YouTube as a Source of Information on Neurosurgery***	To characterize the online video content pertaining to neurosurgery.	Unclear	Neurosurgery	N/A	Mixed methods	Not reported	Other	713	Social media content: YouTube videos	Grounded theory analysis/constant comparison/open, axial and/or selective coding (Thematic)	Green at al (2011); Bradley et al (2007)	Not reported	A total of 713 nonduplicate videos met the inclusion criteria. The overall number of views for all videos was 90,545,164. Videos were most frequently uploaded in 2016 (n = 348), with a 200% increase in uploads compared with the previous year. Of the videos that were directly relevant to clinical neurosurgery, the most frequent video categories were "educational videos" (25%), followed by "surgical and procedure overview" (20%), "promotional videos" (17%), and "patient experience" (16%). The remainder of the videos consisted primarily of unrealistic simulations of cranial surgery for entertainment purposes (20%)
Baniya, Mandira and Rana, Chanda and Dhakal, Raju and Makower, Sophie G. and Halpin, Stephen J. and Hariharan, Ram and Sivan, Manoj and Allsop, Matthew J.	Mixed	2023	Nepal	Other: Masters student	Spinal Injury Rehabilitation Center	Mixed HIC/LMIC	Inquiry : a journal of medical care organization, provision and financing	***The Experience of Limited Access to Care for Community-Based Patients With Spinal Cord Injury and Stroke in Nepal and the Potential of Telerehabilitation: A Qualitative Study***	to understand the experiences of care received and management of a disability for community-based individuals with SCI and stroke	Adult	SCI; stroke	Nepal	Qualitative	Non stated	Patients	17	Interviews (semi-structured, open, in depth)	Reflexive thematic analysis	Braun and Clark (2006)	COREQ	Four generated themes included: (i) Difficulties accessing support and perceived mismanagement following initial neurological injury; (ii) Realizing the magnitude and impact of an injury in the absence of clear routes to support; (iii) A multi-faceted symptom burden and its impact; and (iv) The nature and types of interaction with health professionals post-discharge and the potential role of telerehabilitation.
Hillebregt, C. and Trappenburg, M. and Tonkens, E.	Mixed	2022	The Netherlands	Unclear/not stated	University of Humanistic Studies	All HIC	International Journal of Care and Caring	***Living the life of the other': carers' perspectives on changes in carer strain during the rehabilitation trajectory***	explores the lived experience of carers and the adverse effects of caring throughout the different phases of the rehabilitation care trajectory in the Netherlands, based on in-depth interviews with couples where one partner has acquired brain injury or spinal cord injury.	Adult	Mixed ABI; SCI	Netherlands	Qualitative	Non stated	Patients AND Families/carers/Significant other	40	Interviews (semi-structured, open, in depth)	Other (please state)	Braun and Clarke (2006); Corbin and Strauss (1998)	Not reported	The findings reveal that carers' needs vary during the rehabilitation trajectory, particularly because of varying availability or lack of 'personal time' and 'personal space'. Acknowledging this may improve the assistance of carers, attuned to their evolving needs, and thereby contribute to improved future rehabilitation treatment.
Hamm-Faber, Engels, Vissers, et al.	Mixed	2020	The Netherlands	Not reported	Department of Pain Medicine,	All HIC	PLoS ONE	***Views of patients suffering from Failed Back Surgery Syndrome on their health and their ability to adapt to daily life and self-management: A qualitative exploration***	To explore perspectives on personal health and quality of life (QoL) in FBSS patients concerning their physical-, psychological and spiritual well-being prior to receiving an SCS system.	Adult	Spine; Spinal cord stimulation after failed back surgery syndrome	Netherlands	Qualitative	Not reported	Patients	17	Interviews (open, unstructured, in-depth, semi-structured, open ended)	Thematic analysis	Not reported	COREQ	After analyzing the interviews, three themes emerged: 1) dealing with chronic pain, 2) the current situation regarding aspects of positive health, and 3) future perspectives on health and quality of life. These themes arose from eleven categories and a hundred ninety codes.

Schipper, Visser-Meily, Hendrikx, et al.	Mixed	2011	The Netherlands	Not reported	EMGO+ Institute, VU Medical Center (VUmc), Department of Medical Humanities	All HIC	Brain Injury	***Participation of people with acquired brain injury: Insiders perspectives***	To answer the question how people with ABI experience participation and which environmental and personal factors may influence participation, as perceived by people with ABI.	Adult	Mixed ABI	Netherlands	Qualitative	Not reported	Patients	70	Mixed: Semi-structured interviews; AND focus groups AND working group	Thematic: Content analysis	Lieblich et al (1998)	Not reported	People with ABI contend that it is not the degree of participation that matters, but the quality of participation. They describe meaningful participation in terms of taking part, giving something and being someone. A model was constructed based on the experiences, which includes personal and environmental factors that, in interaction, may influence participation: participation is influenced by the process of recovery, support and treatment, the environment and society and communication and interaction. The study resulted in an overview of actions like continual care that may improve the participation of people with ABI.
Stiekema, Winkens, Ponds, et al.	Mixed	2020	The Netherlands	Neuropsychologist	Department of Psychiatry and Neuropsychology, School of Mental Health and Neuroscience; Brain Injury Center; Alzheimer Center	All HIC	Brain Injury	***Finding a new balance in life: a qualitative study on perceived long-term needs of people with acquired brain injury and partners***	To identify the perceived needs of people with acquired brain injury and their partners.	Adult	Mixed ABI	Netherlands	Qualitative	Not reported	Patients AND Family members, carers, signifcant others	36	Focus groups	Content analysis	Elo, Kyngas (2008)	Not reported	Needs were perceived on the intrapersonal, social, healthcare and societal levels, focusing on three themes: 1) Adaptation to changes, including awareness of consequences, acceptance, role changes and dealing with these; 2) Understanding from relatives/friends, professionals, institutions and society; 3) Timely, individualized care, involving information, transition to home, searching for support, peer support and support for partner/family
Van Velzen, Van Bennekom, Van Dormolen, et al.	Mixed	2011	The Netherlands	Not reported	Coronel Institute of Occupational Health	All HIC	Disability and Rehabilitation	***Factors influencing return to work experienced by people with acquired brain injury: A qualitative research study***	To describe the factors experienced by adults with moderate-to-severe acquired brain injury (ABI) as either limiting or facilitating during the process of return to work (RTW) in order to give an advice about the vocational rehabilitation process.	Adult	Mixed ABI; SCI	Netherlands	Qualitative	Not reported	Patients	12	Interviews (open, unstructured, in-depth, semi-structured, open ended)	Content analysis	Hsieh & Shannon (2005)	COREQ	Participants identified three main themes related to the concept of "sustainable employment". First, the value and impact of initial work integration; an early, multidisciplinary, person-centered work integration, with the early involvement of employers is ideal. A good match between the worker and the workplace is sought. Second, critical factors for long-term sustainable work: the main risks for persons with ABI are changing supervisors, workplace restructuring and the introduction of new technologies, while deteriorating health and the occurrence of secondary health problems are the greatest risk for persons with SCI. Third, the relevance of knowledge, experience and attitudes of professionals; Knowledge of the consequences of an ABI or SCI, the legal basis and the social security process, and the attitude of professionals towards the injured worker were considered important. The most common limiting factor was tiredness. The most common facilitating factors were the will to RTW, the ongoing recovery and the knowledge and support of the employer, colleagues, occupational physician and occupational specialist.
Martin, Levack and Sinnott	Mixed	2015	New Zealand	Not reported	Rehabilitation Teaching and Research Unit, Department of Medicine	All HIC	Disability and rehabilitation	***Life goals and social identity in people with severe acquired brain injury: an interpretative phenomenological analysis***	To explore the lived experience and perceptions of life goals from the perspective of one particular group of people – those with severe acquired brain injury, living in a long-term residential rehabilitation setting.	Adult	Mixed ABI	New Zealand	IPA	Not reported	Patients	5	Interviews (open, unstructured, in-depth, semi-structured, open ended)	IPA	Smith et al. (2009)	Not reported	Three inter-related themes emerged from this study. Social connectedness (being 'part of things') emerged as a life goal of central importance for all participants (Theme 1). However, in order to achieve this sense of belonging, the participants needed to tentatively balance the opportunities arising within their environmental milieu (Theme 2) with the interpersonal factors relating to their unchanged, changed and changing self-identity (Theme 3).
Olaleye, Zaki and Hamzat	Mixed	2021	Nigeria	Not reported	Department of Physiotherapy, Faculty of Clinical Sciences	All LMIC	South African Journal of Physiotherapy	***Expectations of individuals with neurological conditions from rehabilitation: A mixed-method study of needs***	To explore the rehabilitation expectations of individuals with neurological conditions	Adult	Mixed ABI; SCI	Nigeria	Mixed methods (Covergent design)	Not reported	Patients	8	Mixed: Questionnaire AND focus groups	Thematic analysis: Deductive	Not reported	GRAMMS	Sixty-one (58.1%) stroke survivors, 33 (31.4%) individuals with spinal cord injury (SCI) and 11 (10.5%) with traumatic brain injury (TBI) aged 46.48 ± 15.91 were surveyed. The need for social/recreational activity was the most expressed need (100%) amongst the participants. Mobility was reported as an important need constituting a barrier to enjoying life by 93 (88.6%) participants. Individuals with SCI expressed the greatest needs compared with the other two groups. Needs were significantly correlated with severity of disability ($p < 0.05$). Four overarching themes (physical health, financial, healthcare services/rehabilitation and emotional/social) representing major areas of needs emerged from the FGD data.
de Sain, A. M. and Pellikaan, L. W. M. and van Voskuilen, J. and Migdis, M. and Sommers-Spijkerman, M. P. J. and Visser-Meily, J. M. A. and Wajer, Imch	Mixed	2024	The Netherlands	Unclear/not stated	Department of experimental Psychology; Department of neurology and neurosurgery,	All HIC	Disability and Rehabilitation	***Sensory hypersensitivity after acquired brain injury: the patient perspective***	to explore patients' perceptions of sensory hypersensitivity following ABI and its impact on everyday life	Adult	Mixed ABI; sensory hypersensitivity	NL	Qualitative	Non stated	Patients	18	Interviews (semi-structured, open, in depth)	Thematic analysis (inductive)	Braun and Clarke (2006); Hsieh and Shannon (2005)	COREQ	Six themes emerged from the data: (1) definition of sensory hypersensitivity, relating to individual perceptions of sensory hypersensitivity; (2) type of sensory stimuli, relating to the variety of stimuli thatpatients may be sensitive to; (3) course, relating to changes in sensory hypersensitivity following ABI; (4) fatigue, relating to its association with sensory hypersensitivity; (5) consequences of sensory hypersensitivity, relating to the physical, social and emotional impact of sensory hypersensitivity on patients' lives; and (6) coping strategies, relating to behaviours used to cope with sensory hypersensitivity
Geard, Anne, Kirkevold, et al.	Mixed	2020	Norway	Not reported	Sunnaas Rehabilitation Hospital and University of Oslo	All HIC	Health psychology open	***How do family members of individuals with spinal cord and acquired brain injuries make sense of their experiences? A qualitative study of meaning making processes***	To explore the processes that promote coping with abruptive life changes, often experienced as life before and after injury	Adult	Mixed ABI; SCI	Norway	Qualitative	Meaning making	Family members, carers, signifcant others	10	Interviews (open, unstructured, in-depth, semi-structured, open ended)	Thematic analysis	Braun & Clarke (2006)	Not reported	Two main themes were identified: drawing upon different coping strategies and balancing family needs against personal autonomy. Making sense of an altered situation was by keeping a positive outlook, making comparisons to worst cases, engagement in activities and making room for own needs

Walker, Schlebusch and Gaede	Mixed	2021	South Africa	Not reported	Department of Family Medicine, College of Health Sciences,	All LMIC	Journal of Mind and Medical Sciences	***Support for family members who are caregivers to relatives with acquired brain injury***	To show that as the caregivers' stress levels increase, there is an increase in suicidal ideation.	Adult	Mixed ABI	South Africa	Mixed methods Cross sectional descriptive AND phenomenological, quantitative	Not stated	Family members, carers, signifcant others	80	Other: self-report procedures that were part of a structured questionnaire administered individually during the interviews.	Other: Topic analysis	Not reported	Not reported	The analysis of the qualitative data revealed five themes which were identified as the triggers of the caregivers' profound stress. Most caregivers felt that it was predominantly the patient's neuropsychological deficits, such as emotions and/or moods, cognitive ability, behavior and personality, executive function, and social factors that caused them profound stress. Conclusions. Support and education are needed to help family caregivers understand the neuropsychological impact of acquired brain injury on the patient. Once caregivers have an improved understanding and receive better support from healthcare providers, they should experience less stress and be better prepared to provide the appropriate support to patients with acquired brain injury.
Walker, Schlebusch and Gaede	Mixed	2020	South Africa	Not reported	Department of Family Medicine, College of Health Sciences	All LMIC	South African Family Practice	***The impact of stress on depression, ill health and coping in family members caring for patients with acquired brain injury***	To investigate the impact of stress on levels of depression and ill health as an indication of psychological coping	Adult	Mixed ABI	South Africa	Mixed methods	Not reported	Family members, carers, signifcant others	80	Mixed: Interviews (open, unstructured, in-depth, semi-structured, open ended) AND questionnaires AND scoring matrices	Content analysis	Not reported	Not reported	The majority of the research participants experienced high levels of stress along with an inordinate physical and mental health impact indicating that they were not able to cope up with the ongoing chronic stress of caregiving.
Pérez-de la Cruz	Mixed	2022	Spain	Not reported	Department of Nursing, Physical Therapy, and Medicine	N/A	Health Expectations	***Perceptions of recovery and rehabilitation in people with brain injury in Spain. A qualitative study***	To gain insight into ABI patients' perceptions of their condition and rehabilitation process so that physiotherapists can approach their treatment in a more comprehensive, satisfactory manner	Adult	Mixed ABI	Spain	Phenomenology (Hermeneutic)	Interpretative approach	Patients	33	Mixed: Focus groups AND semi-structured interviews	Other: framework method, also known as qualitative content analysis	Not reported	COREQ	Four themes emerged in this study: physiotherapy treatment, changes in lifestyle, patients' feelings about their condition and aspirations for the future. The participants reported that their condition had led to multiple changes in their personal and family lives that were not always positive.
Erikson, Karlsson, Borell, et al.	Mixed	2007	Sweden	University lecturer/doctoral student	Karolinska Institutet, Sweden	All HIC	OTJR Occupation, Participation and Health	***The lived experience of memory impairment in daily occupation after acquired brain injury***	To identify what characterized the lived experience of memory impairment in daily occupations during the first year after acquired brain injury	Adult	Mixed ABI	Sweden	Qualitative (descriptive AND interpretive) Empirical Phenomenological Psychological method	Not reported	Patients	4	Interviews (open, unstructured, in-depth, semi-structured, open ended)	Phenomenological analysis (Modified Empirical Phenomenological Psychological method)	Karlsson (1993)	Not reported	Findings revealed four main characteristics describing the individual's experience during year of rehab: chaotic life world, struggling for 'coherent doing' in new contexts, conscious strategies in new contexts and achieving new habits. After ABI, 'life-world' changed from a taken-for-granted existence to chaotic world difficult to understand. Routines & habits and daily activities broken down - so mostly familiar activities that were already integrated in the 'habit body' that enabled coherent 'doings' in everyday life during the year of rehab.
Häggström and Lund	Mixed	2008	Sweden	Occupational therapist	Department of Occupational Therapy	All HIC	Journal of Rehabilitation Medicine	***The complexity of participation in daily life: A qualitative study of the experiences of persons with acquired brain injury***	To describe and enhance the understanding of how adults with acquired brain injury experience participation in daily life.	Adult	Mixed ABI	Sweden	Qualitative	Not reported	Patients	11	Interviews (open, unstructured, in-depth, semi-structured, open ended)	Content analysis	Graneheim & Lundman (2004)	Not reported	The informants' experiences formed 5 categories: "Performing tasks"; "Making decisions and exerting influence"; "Being engaged in meaningful activities"; "Doing things for others"; and "Belonging". The categories that needed to be present for the informants to experience a feeling of participation varied according to their individual daily life situations. In addition, their experiences showed that a variety of conditions, related to each of the 5 categories, influenced their participation. Individuals adopted a variety of strategies to enhance their experience of participation.
Lundqvist and Samuelsson	Mixed	2012	Sweden	Not reported	Rehabilitation Medicine, Department of Clinical and Experimental Medicine, Faculty of Health Sciences	All HIC	Brain Injury	***Return to work after acquired brain injury: A patient perspective***	To increase the knowledge and understanding of significant factors for returning to work after acquired brain injury from a patient perspective.	Adult	Mixed ABI	Sweden	Not stated	Not reported	Family members, carers, signifcant others AND HCPs	19	Focus groups	Content analysis	Hsieh & Shannon (20050, Graneheim & Lundman (2004)	Not reported	Two themes were identified as significant for returning to work: Personal and Society factors. Identified meaningful units could be categorized into sub-categories, which were grouped into six main- and 14 sub-categories. The main categories were: Self-continuity, Coping, Social factors, Rehabilitation intervention, Professionalism and Health insurance. Length of treatment time was described as crucial for the rehabilitation process and for utilizing individual resources.
Matérne, Lundqvist and Strandberg	Mixed	2017	Sweden	Not reported	University Health Care Research Center, Faculty of Medicine and Health	All HIC	Work	***Opportunities and barriers for successful return to work after acquired brain injury: A patient perspective***	To increase knowledge of opportunities and barriers for a successful return to work (RTW) in patients with ABI.	Adult	Mixed ABI	Sweden	Qualitative	Hermeneutic theory of science,	Patients	10	Interviews (open, unstructured, in-depth, semi-structured, open ended)	Content analysis	Graneheim & Lundman (2004)	Not reported	Three themes that influenced RTW were identified: individually adapted rehabilitation; motivation for RTW; and cognitive and social abilities. An individually adapted rehabilitation was judged important because the patients were involved in their own rehabilitation and required individually adapted support from rehabilitation specialists, employers, and colleagues. A moderate level of motivation for RTW was needed. Awareness of the person's cognitive and social abilities is essential, in finding compensatory strategies and adaptations.
Mylén, Nilsson and Berterö	Mixed	2016	Sweden	Not reported	Department of Neuro Surgery and Neurosurgical Intensive Care	All HIC	Intensive and Critical Care Nursing	***To feel strong in an unfamiliar situation; Patients' lived experiences of neurosurgical intensive care. A qualitative study***	To explore the lived experiences of conscious patients in neurosurgical intensive care.	Adult	Unclear - not directly stated	Sweden	Phenomenology (Interpretive)	Not reported	Patients	11	Interviews (open, unstructured, in-depth, semi-structured, open ended)	Phenomenological analysis Stevick—Colaizzi—Keens method modified by Moustakas	Moustakas (1994)	Not reported	The analysis revealed three themes: To feel safe in an unfamiliar situation, to experience strains and limitations, and to be confirmed as a human being. These three themes culminated in the essence: To feel strong in an unfamiliar situation. Patients experienced a soothing environment where, despite strains, they felt safe being cared for in a ward with specialized medical treatment. When mental and physical strains decreased during the period of care, they experienced the ability to cope with the simplest tasks as a sign of regained identity.
Guidetti, Susanne, Asaba, et al.	Mixed	2009	Sweden	Occupational therapist	Department of Neurobiology, Care Sciences and Society, Division of Occupational Therapy	All HIC	The American journal of occupational therapy : official publication of the American Occupational Therapy Association	***Meaning of context in recapturing self-care after stroke or spinal cord injury***	To identify the meaning of context in recapturing self-care after a stroke or SCI	Adult	Stroke + spinal cord injury	Sweden and UK	Phenomenology (Empirical, phenomenological and psychological method)	Phenomenological life-world perspective	Patients	11	Interviews (open, unstructured, in-depth, semi-structured, open ended)	Interpretive, descriptive analysis	Karlsson (1993)	Not reported	They were analyzed by using the empirical, phenomenological, psychological method, which identified 6 main characteristics describing the role of context in recapturing self-care: (1) support from others, (2) an air of expectation, (3) extended time, (4) new daily structure, (5) therapeutic relationship as enabling possibility, and (6) gradual change in challenge. These findings showed that rehabilitation professionals play an important role in creating a context that contributes to recapturing self-care by allowing extended time, enabling patients to see possibilities, and creating expectations for them to do things on their own.

Freeman, J. and Raabe, A. and Schmitz, F. and Guttormsen, S.	Mixed	2024	Switzerland	Unclear/not stated	Department of Neurosurgery	All HIC	Bmc Medical Education	***How neurosurgeons maintain and update their professional knowledge in a self-directed learning context***	To investigate how neurosurgeons overcome the challenges of the current learning landscape to maintain and update their professional knowledge outside of their organized training, in-house curriculum or formal continual education	N/A	Education	Switzerland	Qualitative	Non stated	Neurosurgeons (inc. trainees/residents)	26	Interviews (semi-structured, open, in depth)	Thematic analysis	Witkowsky and Bingham (2021)	COREQ	One of the main findings concerns the differences between neurosurgeons regarding the self-directed learning strategies they employ, which is compounded by their level of experience. All participants recognized that new or alternative learning approaches are necessary to manage the learning landscape, and for many this concerned their use of learning digital tools. Many, however, were unsure how to change their current behavior.
Petitt, Z. and Ordonez, Y. T. and Kelkar, Y. and Shakir, M. and Ott, M. and Patel, Y. and Agwu, C. and Khalafallah, A. M. and Mullikin, A. and Tang, A. L. and Davis, J. and Ssembatya, J. M. and Choi, S. and Deng, D. D. and Headley, J. and Obiga, O. and Haglund, M. M. and Fuller, A. T.	Mixed	2024	USA	Unclear/not stated	Division of Global Neurosurgery and Neurology; Duke University Global Health Institute	Mixed HIC/LMIC	World Neurosurgery	***A Mixed-Methods Assessment of the Feasibility of Conducting Neurosurgical Clinical Research in Uganda***	to evaluate neurosurgical providers' perspectives on clinical research and documentation patterns of neurosurgical variables at Mulago National Referral Hospital.	N/A	mixed	Uganda	Mixed methods,	Not explicitly stated	HCPs (Inc NSx)	22	Focus groups	Framework analysis	Gale et al. (2013)	Not reported	Chart review showed that primary diagnosis (99.4%), pupil light response (97.6%), and computed tomography scan results (93.3%) were documented for most patients. Cranial nerve exam (61.5%), pupil size (69.9%), and time to neurosurgical intervention (45%) were documented less frequently. On average, Glasgow Coma Scale was documented for 86.6% of days hospitalized, while vital signs were documented for 12.3%. In most focus group discussions, participants identified follow-up, financing, recruitment, time, approval, and sociocultural factors as research barriers. Participants described how the current health workforce facilitates successful research. To improve research capacity, suggested strategies focused on research networks, data collection, leadership, participant recruitment, infrastructure, and implementation.
Beal, E. M. and Pelser, C. and Coates, P.	Mixed	2023	UK	Other: Researchers	Merseycare NHS Foundation Trust	All HIC	Brain Impairment	***Lockdown life-experiences of partners of individuals with an acquired brain injury during the COVID-19 pandemic: a qualitative study***	to explore partner experiences of supporting and caring for an individual with an ABI, during the lock down period, in absence of face-to-face community rehabilitation and social visits	Adult	Mixed ABI	UK	Qualitative	critical realism lens	Family/Carer/Significant other	7	Interviews (semi-structured, open, in depth)	Thematic analysis	Braun and Clark (2006)	COREQ	ABI occurs within a relational framework, which means that it has repercussions not only for the individual but also the entire family system. COVID-19 prevented family systems (living separately) from coming together which negatively impacted them; however, it also slowed life down, with many people working from home with flexible arrangements in place which participants found to be beneficial. Three main themes emerged from the interview data: partner focus, slowing down and support networks. The narratives identified the struggles of having to continue their partner's rehabilitation when face to face services could not visit the home, the importance of establishing routine, the positives of a slower paced life (due to COVID-19) that enabled them to build stronger relationships with their partners, and the difficulties of being separated fromfamily and loved ones.
Kusec, A. and Methley, A. and Murphy, F. C. and Peers, P. V. and Carmona, E. and Manly, T.	Mixed	2023	UK	Other: Masters degree in rehabilitation science	MRC Cognition and Brain Sciences Unit	All HIC	Bmc Psychology	***Developing behavioural activation for people with acquired brain injury: a qualitative interpretive description study of barriers and facilitators to activity engagement***	To explore perceptions of adults with ABI and their family members on 1) barriers to engaging in meaningful or enjoyable activities; 2) facilitators of such engagement, and 3) the likely utility of specifc behavioural activation techniques and psychoeducational topics in fostering greater engagement.	Adult	MIXED ABI	UK	Interpretive phenomenological analysis (IPA)	Constructivist	Patients AND Families/carers/Significant other	23	Interviews (semi-structured, open, in depth)	Constant comparison	Glaser (1965)	Not reported	The final constructed framework, Creating Sustainable Engagement, comprises a two-tier hierarchy. Higher- level themes concerned core perspectives of BA, regardless of BA component discussed. This included identifying optimal time windows for different BA components (Right Tool at the Right Time), that BA components should,<br>at least initially, not be burdensome or fatiguing (Perceived Effort), that emotional readiness to confront activity-mood relationships should be addressed (Emotional Impact), and that planned BA activities be consistent with individual values (Relation to Values). Lower-level themes concerned specific BA components: Of these, activity scheduling, procedures targeting avoidance, managing uncertainty and social/ communication skills were generally well-received, while mood monitoring, contingency management, and mindfulness had mixed feedback.
Bray	Mixed	2015	UK	RN/Nurse	placements and commissioning, at the Children's Trust	N/A	Nursing children and young people	***Parents' experiences of hope following a child's brain injury***	To explore the lived experiences of parents caring for their child following a severe to moderate acquired brain injury (ABI).	Pediatric	Mixed ABI	UK	Phenomenology (Heideggerian)	Naturalistic paradigm	Family members, carers, signifcant others	8	Interviews (open, unstructured, in-depth, semi-structured, open ended)	Thematic analysis	Burnard (1991)	Not reported	Every parent acknowledged the need to maintain hope. The other themes identified were: the effects on the child post ABI, the need for accurate information, emotional support, effects on the family, fear of death, transition, family accommodation and funding.
Brunsden, Kiemle and Mullin	Mixed	2017	UK	Not reported	Department of Clinical Psychology	All HIC	Neuropsychological rehabilitation	***Male partner experiences of females with an acquired brain injury: An interpretative phenomenological analysis***	To explore male partner experiences of living with a female with an ABI.	Adult	Mixed ABI	UK	IPA	Phenomenology, hermeneutics and idiography	Patients	6	Interviews (open, unstructured, in-depth, semi-structured, open ended)	IPA	Smith et al (2009)	Not reported	Four main themes captured the male partner's lived experience; "Entering the unknown world of ABI", "Imprisoned by the ABI", "Compassion without self-compassion", and "Holding on to hope". The accounts uncovered the male partner's journey following ABI; the unpredictability, sorrow, frustration, and finally acceptance and commitment to their partner. They identified the limited support available for partners following ABI; the sense of feeling forgotten by services and the need for information and support in the acute stages.
Buckland, Kaminskiy and Bright	Mixed	2021	UK	Not reported	Division of Psychology	All HIC	Neuropsychological Rehabilitation	***Individual and family experiences of loss after acquired brain injury: A multi-method investigation***	(1) What feelings of loss are experienced by those who have had an ABI? (2) What feelings of loss are experienced by the relatives of those who have had an ABI? (3) Are there differences in experiences of loss among dyadic relationships, or is loss experienced in the same way in individuals with ABI and their relatives?	Adult	Mixed ABI	UK	Multi-method (Survey AND Qualitative)	Pragmatic epistemology using inductive and deductive methods; constructivist - interpretivist epistemology	Patients AND Family members, carers, signifcant others	40	Mixed: Interviews (open, unstructured, in-depth, semi-structured, open ended) AND questionnaires	Other: Bryman's (2008) four stages of qualitative analysis	Bryman (2008)	COREQ	Five main themes emerged from the interviews: loss of person; loss of relationships; loss of activity/ability; loss of future; unclear loss. There were distinct differences qualitatively between individuals and relatives and only two dyads experienced similar loss, but there were no significant differences in loss as measured quantitatively by the BIGI. The differences between relatives' loss and individuals with ABIs' loss are discussed.
Cregan, Daisley, Ford, et al.	Mixed	2021	UK	Trainee clinical psychologist	Department of Clinical Psychology and Psychological Therapies, Norwich Medical School	All HIC	Neuropsychological Rehabilitation	***A qualitative exploration of fatherhood after acquired brain injury (ABI)***	To explore how men who were fathers before their injuries experience fatherhood after ABI.	Adult	Mixed ABI	UK	IPA	Critical realism with a phenomenologist perspective	Patients	7	Interviews (open, unstructured, in-depth, semi-structured, open ended)	IPA	Smith et al (2009)	Not reported	Four superordinate themes were drawn from all interviews through engaging with the qualitative research process: (1) what being a father means, (2) altered relationships with others, (3) becoming lost and finding their way through, and (4) renewed fatherhood. The findings show intersectionality between pre-and post-injury comparisons of self and social identities, alongside the contextual and societal identities in the subjective fathering experiences.

Ghosh-Cannell, Fisher, Ajayi, et al.	Mixed	2022	UK	Psychologist (incl. clinical, neuro-)	Department of Clinical Psychology	All HIC	Neuropsychological Rehabilitation	***The experiences of wives following acquired brain injury (ABI). A qualitative analysis exploring realizations of change following the ABI of a "loved one."***	To explore the experiences of realization of change for married women living with their husbands following ABI.	Adult	Mixed ABI	UK	IPA	Not reported	Family members, carers, signifcant others	9	Interviews (open, unstructured, in-depth, semi-structured, open ended)	IPA	Braun & Clarke (2013), Smith et al (2009)	COREQ	An Interpretative Phenomenological Analysis (IPA) was completed, arriving at two overarching themes; "bravery to face changes" and "lost and trapped in an unsolvable maze," with accompanying subthemes. Participants generally experienced realization of change gradually, in some cases finding strategies to control their exposure to distress. They often referred to "acceptance," which held varied meanings, and metaphors appeared to aid personal meaning making. Relationship changes generated both dilemmas and the feeling of being trapped.
Gibson and Watkins	Mixed	2013	UK	RN/Nurse	School of Health	All HIC	Health Expectations	***The use of formal and informal knowledge sources in patients' treatment decisions in secondary stroke prevention: Qualitative study***	To examine the use of formal and informal knowledge by patients in making decisions about carotid endarterectomy (CEA) and medical treatment after TIA/recovered stroke.	Adult	Transient ischemic attack (TIA) or stroke with carotid endarterectomy (CEA) or medical treatment	UK	Qualitative	Not reported	Patients	20	Interviews (open, unstructured, in-depth, semi-structured, open ended)	Grounded theory analysis/constant comparison/open, axial and/or selective coding	Strauss and Corbin (1990)	Not reported	Participants gathered and utilized several types of knowledge in the process of making treatment decisions: Empirical knowledge (e.g. clinical trial findings); Pathophysiologic findings (e.g., results of clinical investigations); Experiential knowledge (e.g., personal experience of stroke); Goals and values (e.g., potential impact on family); System features (e.g., apparent urgency of treatment).
Holloway, Orr and Clark-Wilson	Mixed	2019	UK	Not reported	Head First	All HIC	Brain Injury	***Experiences of challenges and support among family members of people with acquired brain injury: a qualitative study in the UK***	To explore how families are affected and integrates their views on the formal/informal support received as a consequence of Acquired brain injury	Mixed	Mixed ABI	UK	Qualitative	Not reported	Family members, carers, signifcant others	16	Interviews (open, unstructured, in-depth, semi-structured, open ended)	Thematic analysis	Braun & Clarke (2006); Guest, MacQueen, Namey (2012)	Not reported	Family members' experiences are complex, enduring and are affected by the context in which the ABI occurs as well as by formal/informal support. The grief experienced by FM is ambiguous, develops over time and FM perceive little option but to remain involved. Experience of formal and informal support is noted to vary significantly in availability and quality, poor support exacerbates difficulties and isolates family members.
Jones, Tyson, Davis, et al.	Mixed	2020	UK	clinical researcher/major trauma coordinator	Division of Nursing, Midwifery & Social Work	All HIC	BMJ Open	***Qualitative study of the needs of injured children and their families after a child's traumatic injury***	To explore the needs of children and their families after a child's traumatic injury.	Pediatric	TBI; SCI	UK	Qualitative (descriptive)	Not reported	Patients AND Family members, carers, signifcant others	32	Interviews (open, unstructured, in-depth, semi-structured, open ended)	Thematic analysis (A theoretical thematic analysis, coded to existing framework)	Braun & Clarke (2006)	Not reported	Interviews were conducted a median 8.5 months (IQR 9.3) postinjury. Injuries affected the limbs, head, chest, abdomen, spine or multiple body parts. Participants highlighted needs throughout their recovery (during and after the hospital stay). Education and training were needed to help children and families understand and manage the injury, and prepare for discharge. Information delivery needed to be timely, clear, consistent and complete, include the injured child, but take into account individuals' capacity to absorb detail. Similarly, throughout recovery, services needed to be timely and easily accessible, with flexible protocols and eligibility criteria to include injured children. Treatment (particularly therapy) needed to be structured, goal directed and of sufficient frequency to return injured children to their full function. A central point of contact is required after hospital discharge for advice, reassurance and to coordinate ongoing care. Positive partnerships with professionals helped injured children and their families maintain a sense of hope and participate in joint decision making about their care.
Jordan and Linden	Mixed	2013	UK	Not reported	Private Practice	All HIC	Brain Injury	***'It's like a problem that doesn't exist': The emotional well-being of mothers caring for a child with brain injury***	To provide an account of the experiences of mothers who care for a childhood survivor of brain injury.	Pediatric	Mixed ABI	UK	Survey	Not reported	Family members, carers, signifcant others	86	Questionnaire (essay style questions)	Thematic analysis	Braun & Clarke (2006)	Not reported	Thematic analysis identified five key themes: Perpetually Anxious, The Guilty Carer, The Labour of Caring, A Self-Conscious Apologist and Perpetually Grieving. Collectively, these themes highlight two core processes shaping mothers' caring experiences and concomitant mental well-being. First, the collective and enduring nature of caregiver burden over time. Second, the crucial role played by socio-cultural values in perpetuating caregiver burden.
Kean	Mixed	2010	UK	RN/Nurse	Nursing Studies, School of Health in Social Science,	N/A	Nursing in Critical Care	***The experience of ambiguous loss in families of brain injured ICU patients***	To explore families' experiences with critical illness in intensive care and nurses' perception of families. This article presents findings of one specific aspect, namely, families who experienced an ambiguous loss following the patient's brain injury which resulted in permanent brain damage.	Mixed	Mixed ABI	UK	Grounded theory	Constructivist	Family members, carers, signifcant others	24	Interviews (family group interview)	Grounded theory analysis/constant comparison/open and axial/selective coding	Glasser & Strauss (1967)	Not reported	Thefindingssuggestthat'theemergenceofambiguousloss'reflectsthefamilies'experienceswiththesecondtypeofambiguousloss, namely a loss that relates to a family member who was physically present but psychologically absent. 'Mapping the future' is a further dimension of this theme which underlines the impact of an ambiguous loss on everyday family life.
Khan, Doke and Boeris	Mixed	2018	UK	Not reported	Department of Neurosurgery	All HIC	World Neurosurgery	***Nurturing the Next Generation of Neurosurgeons: How Important Are Nontechnical Skills?***	The present study, therefore, queried both neurosurgical trainees and tutors regarding how much they valued NTSs and their development.	N/A	Education	UK	Mixed methods (Survey)	Not reported	NSx (trainees, tutors)	30	Questionnaire: Open & closed questions	Thematic analysis	Not reported	Not reported	The trainees valued NTSs and their development more than did their tutor counterparts ($P < 0.05$, z-testing). This was evident from both the quantitative results and the qualitative data gained from the questionnaire. Various areas for potential improvement of the curriculum were also identified.
Lond and Williamson	Mixed	2018	UK	Not reported	Division of Psychology	All HIC	Disability and Rehabilitation	***"Stuck in a loop of fear": a phenomenological exploration of carers' experiences supporting a spouse with acquired brain injury***	To examine how individuals experience and manage caregiving for a spouse or partner with ABI.	Adult	Mixed ABI	UK	Phenomenology	Stress process model of caregiving; Phenomenology	Family members, carers, signifcant others	8	Interviews (open, unstructured, in-depth, semi-structured, open ended)	IPA	Smith et al (2009)	Not reported	One theme, "Living in and beyond the loop of fear", with two subheadings is reported. Participants' attempts to manage their fears prominently defined their early caregiving. Fears were aggravated by the vulnerability of their spouse's health which partially owed to brain injury sometimes having no symptoms prior to its onset. Consequently, participants anxiously strove to prevent further harm to their spouse's health due to what they perceived as the continued "hidden" threat of brain injury. Therefore, participants became hypervigilant, leaving themselves vulnerable to burnout. Over time, some participants modified care practices and managed fears using beliefs accepting their limits to protect their spouses' health.
Lond and Williamson	Mixed	2020	UK	Not reported	Division of Psychology	All HIC	Disability and Rehabilitation	***Acceptance, grief and adaptation amongst caregivers of partners with acquired brain injury: an interpretative phenomenological enquiry***	To identify coping mechanisms and support systems that enhanced well-being.	Adult	Mixed ABI	UK	IPA	Idiographic sensibility	Family members, carers, signifcant others	8	Interviews (open, unstructured, in-depth, semi-structured, open ended)	IPA	Smith, Flowers & Larkin (2009)	Not reported	Three themes are reported—"moving through denial toward acceptance"; "confronting and managing ambiguous loss"; and "becoming an expert carer". Theme one describes participants' struggles to accept the longevity of brain injury and use of strenuous care practices to deny or fight disability; this proved counterproductive and was later remedied by individuals embracing change and making adaptations. Theme two reports how participants split their partners' identities -before and after brain injury- to help grieve for the marital relationships they lost. Theme three looks at participants' development of self-reliant attitudes to caregiving due to perceived limited state help, while embracing peer support that enhanced information and emotion-based coping.

Marcus, Cundy, Hughes-Hallett, et al.	Mixed	2014	UK	Neurosurgeon	Department of Neurosurgery	All HIC	British Journal of Neurosurgery	***Endoscopic and keyhole endoscope-assisted neurosurgical approaches: A qualitative survey on technical challenges and technological solutions***	To assess the technical challenges of neuroendoscopy, and the scope for technological innovations to overcome these barriers.	N/A	Endoscopic and keyhole endoscope-assisted neurosurgical approaches	UK	Survey	Not reported	NSx	40	Open ended questions	Other: using iterative analysis, responses were parsed for themes	Not reported	Not reported	Three clear themes emerged: 1) surgical approach and better integration with imageguidance systems (20%), 2) intra-operative visualisation and improvements in neuroendoscopy (49%), and 3) surgical manipulation and improvements in instruments (74%)
Moore and Gillespie	Mixed	2014	UK	Not reported	Department of Psychology	All HIC	Social Science and Medicine	***The caregiving bind: Concealing the demands of informal care can undermine the caregiving identity***	To compares the views of people with acquired brain injury and their main informal caregivers. To explore how caregivers felt themselves to be perceived by significant others.	Adult	Mixed ABI	UK	Other: Interpersonal Perception Method	Not reported	Patients AND Family members, carers, signifcant others	56	Other: Video recordings of the discussion during the rating task	Type unclear	Hengst et al. (2008), Macintosh et al.(2012)	Not reported	Caregivers felt that the care-receiver, family members, the general public, health services and even friends often have negative views of them. The 'caregiving bind' is proposed as a cause of caregivers' negative identity. It arises when caregivers try to protect the care-receiver's identity by concealing the extent of informal care provision, with the unintended consequence of undermining the prospects of the caregiver receiving positive social recognition for the challenging work of caregiving. The caregiving bind has implications for therapy and points to the potential of friends and health services to provide caregivers with positive social recognition.
Moreno-Lopez, Holttum and Oddy	Mixed	2011	UK	Not reported	Kent and Medway NHS and Social Partnership Trust	All HIC	Brain Injury	***A grounded theory investigation of life experience and the role of social support for adolescent offspring after parental brain injury***	To explore the experiences of adolescent offspring following parental acquired brain injury (ABI) and the role of supportive relationships in offspring coping.	Adult	Mixed ABI	UK	Grounded theory	Critical realism	Family members, carers, signifcant others	12	Interviews (open, unstructured, in-depth, semi-structured, open ended)	Grounded theory analysis/constant comparison/open, axial and/or selective coding	Strauss and Corbin (1998)	Not reported	Findings indicate that following the shock of the ABI, adolescents appeared to go through a dynamic process of reevaluation of life priorities, a sudden realization of the fragility of life, and an increase in their appreciation of the family's needs. The study's findings also show that adolescent offspring can have a protective role in the family, assisting the family with the long-term challenges that ABI may present. Alternative parental figures and peer friendships appeared to have a central role for participants, providing a sense of normality and stability to their lives, and acted as an important connection with their lives prior to their parents' ABI.
Mukherjee	Mixed	2022	UK	Not reported	Department of Academic Neurosurgery	N/A	British Journal of Neurosurgery	***What does it take to become a neurosurgeon? A mixed methods cross-sectional analysis of professional identity formation amongst neurosurgical trainees***	To assess neurosurgical trainees' perceptions of professional identity, how it develops and how it might be enhanced during the course of training.	N/A	Other	UK	Mixed methods	Not reported	NSx	15	Mixed: Interviews (open, unstructured, in-depth, semi-structured, open ended) AND questionnaires	Other: Type undefined, stages included coding, grouping, categorizing and theming	not reported	Not reported	A model has been constructed that describes professional identity formation amongst neurosurgical trainees at different stages of training, and how these feed into an aspired core identity profile of a Neurosurgeon. Based on this model, suggestions have been made to potentially improve professional identity formation amongst neurosurgical trainees.
Riley, Keeble, Yasmin, et al.	Mixed	2020	UK	Not reported	Center for Applied Psychology	All HIC	Neuropsychological Rehabilitation	***Relationship continuity and person-centered care: An exploratory mixed-methods investigation of spousal partners' responses to the challenging care needs of those with acquired brain injury***	To provide a more robust test of the suggestion that there is a link between continuity/discontinuity and a more person-centered response to challenging care needs.	Adult	Mixed ABI	UK	Mixed methods	VIPS theoretical framework (Valuing, individual, perspective, Social)	Family members, carers, signifcant others	26	Mixed: Interviews (open, unstructured, in-depth, semi-structured, open ended) AND questionnaires	Other: Interviews were coded and scored to provide a measure of the extent to which the participants' understanding, management and emotional responses showed a person-centered approach.	Not reported	Not reported	The findings supported the hypothesis. Greater continuity was significantly correlated with a more person-centered approach. Associating relationship continuity and person-centered care is a novel approach to the issue of how family relationships may impact on care quality. Person-centered care can have important benefits for both the giver and receiver of care. Whether it can be promoted through fostering a sense of continuity in the relationship merits further investigation.
Tyerman, Eccles, Gray, et al.	Mixed	2019	UK	Not reported	Division of Health Research, Faculty of Health and Medicine	All HIC	Disability and Rehabilitation	***Siblings' experiences of their relationship with a brother or sister with a pediatric acquired brain injury***	To explore siblings' experiences of their relationship with their brother or sister with acquired brain injury in order to make recommendations for health professionals working with this population.	Pediatric	Mixed ABI	UK	IPA	Phenomenological, idiographic hermeneutics	Family members, carers, signifcant others	5	Interviews (open, unstructured, in-depth, semi-structured, open ended)	IPA	Smith et al. (2009)	Not reported	The siblings in this study experienced a high level of distress with the near loss of their brother or sister. This was followed by difficulty in adjusting to the physical and psychological changes in their injured sibling and the impact on their sibling role and relationship. The changes were experienced alongside disruption to family relationships. Important clinical implications include the inclusion of siblings in their injured sibling's care and the provision of information and support for this group. Implications for Rehabilitation: Siblings of children with an acquired brain injury experience significant challenges while trying to adapt to their changing sibling relationship and feelings of disconnection with their family. This study highlights a need to work systemically with families of childhood brain injury and recognize siblings' important role in their family unit and therefore involve them in their brother/sister's care and rehabilitation. This study also highlights a need to support siblings to cope with the trauma and provide information to validate and understand their experience.
Yeates, Henwood, Gracey, et al.	Mixed	2007	UK	Not reported	Oliver Zangwill Center for Neuropsychological Rehabilitation	All HIC	Neuropsychological Rehabilitation	***Awareness of disability after acquired brain injury and the family context***	The dimension of family context for awareness of disability following acquired brain injury (ABI) is examined through a qualitative discourse analysis.	Adult	Mixed ABI	UK	Qualitative	Social Constructivism	Patients	3	Interviews (open, unstructured, in-depth, semi-structured, open ended)	Discourse analysis	Charmaz (1990); Potter & Wetherell (1987)	Not reported	The findings highlight important contextual parameters influencing the emergence of families' accounts for disability after ABI: (1) availability of sense-making resources and use of pre-injury meanings, and (2) incongruity within family sense-making and resultant orientating, disputing and contesting of accounts within families.

Sichimba, Bandyopadhyay, Ciuculete, et al.	Mixed	2022	Cameroon / Zambia	Not reported	Department of Research, Association of Future African Neurosurgeons; Michael Chilufya Sata School of Medicine	All LMIC	Frontiers in Surgery	***Neurosurgical Equipment Donations: A Qualitative Study***	To explore: (1) the need for the donation of neurosurgical equipment from the UK and Ireland to LMICs within the African continent, and (2) the ways through which neurosurgical equipment donations could meet the needs of LMIC neurosurgeons.	N/A	Neurosurgery	UK/ Ireland / Benin / Cameroon / Zimbabwe	Qualitative	Not reported	NSx	8	Interviews (open, unstructured, in-depth, semi-structured, open ended)	Grounded theory analysis/constant comparison/open, axial and/or selective coding (Thematic)	Corbin, Strauss, Strauss (2008)	Not reported	Five HIC and 3 LMIC neurosurgeons were interviewed. Five overarching themes were identified: (1) inequality of access to neurosurgical equipment, (2) identifying specific neurosurgical equipment needs, (3) importance of Organizations, (4) partnerships between LMIC and HIC Centers, and (5) donations are insufficient in isolation.
Kitzinger and Kitzinger	Mixed	2013	UK	Not reported	School of Journalism, Media and Cultural Studies	All HIC	Sociology of Health and Illness	***The 'window of opportunity' for death after severe brain injury: Family experiences***	We show how the medico-legal 'window of opportunity' for allowing the patient to die structures family experience and fails to deliver optimal outcomes for patients.	Mixed	Mixed ABI	Unclear	Qualitative	Realism / Interpretive autobiography	Patients AND Family members, carers, signifcant others	34	Interviews (open, unstructured, in-depth, semi-structured, open ended)	Thematic analysis	Braun & Clarke (2006)	Not reported	Our analysis tracks the decision-making processes that have led to the situation in which life-sustaining treatments continue to be delivered to these patients – maintaining them in a state that some families describe as a 'fate worse than death'. We show how the medico-legal 'window of opportunity' for allowing the patient to die structures family experience and fails to deliver optimal outcomes for patients. We end with some suggestions for change.
McPherson, McNaughton and Pentland	Mixed	2000	New Zealand	Not reported	School of Medicine	All HIC	International Journal of Rehabilitation Research	***Information needs of families when one member has a severe brain injury***	To address the following: a) do carers for those with severe BI need more information in post-discharge period? b) What sort of information do they require? c) Can one distinguish who needs what information? d) What are the barriers to carers getting the information they require?	Mixed	Mixed ABI; Trauma (TBI) + spontaneous ICH	Unclear - Not reported	Mixed methods	Naturalistic enquiry	Family members, carers, signifcant others	82	Mixed: Open ended interview and mixed surveys	Grounded theory analysis/constant comparison/open, axial and/or selective coding	Strauss and Corbin (1990)	Not reported	Even soon after discharge from inpatient rehabilitation, carers wanted more information. In many cases the need for information was unrelated to either the severity of injury or level of functional deficit. In addition, requests for information were in many cases not sought spontaneously, but required prompting. Such findings have implications if interventions in this field are to have the optimum chance of succeeding in providing support and assistance.
Karagiorgou, Evans and Cullen	Mixed	2018	UK	Not reported	Mental Health and Wellbeing, Institute of Health and Wellbeing	All HIC	Disability and Rehabilitation	***Post-traumatic growth in adult survivors of brain injury: a qualitative study of participants completing a pilot trial of brief positive psychotherapy***	We aimed to investigate PTG experiences in participants of a positive psychotherapy pilot trial.	Adult	Mixed ABI	Unclear - possibly UK	Qualitative	Not reported	Patients	7	Interviews (open, in-depth, semi-structured, open ended)	Thematic analysis	Braun & Clarke (2006)	Not reported	Four participants (age 1⁄4 46–62; n 1⁄4 3 male; months since injury 1⁄4 11–20) from the PPT group and three (age1⁄458–74; n1⁄42 male; months since injury1⁄49–22) from the TAU group were interviewed. Six themes were shared across both groups: personal strength, appreciation of life, relating to others, optimism/positive attitude, feeling fortunate compared to others, and positive emotional/behavioral changes. Two themes were expressed by PPT participants only: lifestyle improvements and new possibilities. One TAU participant reported spiritual change.
Carr and Fogarty	Mixed	1999	USA	RN/Nurse	School of nursing	All HIC	The Journal of Family Practice	***Families at the bedside: An ethnographic study of vigilance***	To examine the meanings, patterns, and day-to-day experience of vigilance.	Adult	Mixed ABI (traumatology)	US	Ethnography	Not reported	Family members, carers, signifcant others	16	Mixed: Interviews (open, unstructured, in-depth, semi-structured, open ended) AND observations	Type unclear (coded, categorized, clustered, named)	Not reported	Not reported	Data analysis yielded 5 categories of meaning that describe the experience of vigilance: commitment to care, emotional upheaval, dynamic nexus, transition, and resilience.
Goss, A. L. and Voumard, R. R. and Engelberg, R. A. and Curtis, J. R. and Creutzfeldt, C. J.	Mixed	2023	USA	Physician/MD	Division of Neurology, Department of Internal Medicine	All HIC	Critical Care Medicine	***Do They Have a Choice? Surrogate Decision-Making After Severe Acute Brain Injury****	To describe surrogate experiences of decision-making and provider-surrogate communication in SABI. As a secondary objective, we aimed to construct an explanatory framework by which to understand these experiences.	Adult	Mixed ABI	USA	Qualitative	Constructivist grounded theory	Family/Carer/Significant other	22	Interviews (semi-structured, open, in depth)	Grounded theory analysis	Glasser and Strauss (2012)	Not reported	Two distinct perspectives on decision-making emerged: one group of surrogates felt a clear sense of agency around decision-making, while the other group reported a more passive role in decision-making, such that they did not even perceive there being a decision to make. Surrogates in both groups identified prognostic uncertainty as the central challenge in SABI, but they managed it differently. Only surrogates who felt they were activelydeciding described time-limited trials as helpful.
Hu, F. Y. and Rowe, K. A. and O'Mara, L. M. and Bulger, A. and Bleday, R. and Groff, M. W. and Cooper, Z. and Bernacki, R. E.	Mixed	2023	USA	Physician/MD	Center for Surgery and Public Health; Department of Surgery	All HIC	Journal of the American Geriatrics Society	***Evaluation of interdisciplinary care pathway implementation in older elective surgery patients***	This qualitative study explored clinician experiences to understand influences on implementation of frailty screening and an interdisciplinary care pathway in older elective colorectal surgery and neurosurgery patients.	Adult		USA	Qualitative	Consolidated framework for implementation research	HCPs (Inc NSx)	32	Interviews (semi-structured, open, in depth)	Other: codebook based on consolidated frameowrk for implementation research	non stated	Not reported	Key themes to implementation success included strong participant belief in effectiveness of the intervention and its advantage over standard care; the importance of training, reference materials, and champions; and the need for institution-level investment in resources to amplify the impact of the intervention on patients and expand the capacity to address their needs.

Hwang, D. Y. and Bannon, S. M. and Meurer, K. and Kubota, R. and Baskaran, N. and Kim, J. and Zhang, Q. and Reichman, M. and Fishbein, N. S. and Lichstein, K. and Motta, M. and Muehlschlegel, S. and Reznik, M. E. and Jaffa, M. N. and Creutzfeldt, C. J. and Fehnel, C. R. and Tomlinson, A. D. and Williamson, C. A. and Vranceanu, A. M. and Investigators, Coma- F.	Mixed	2024	USA	Other: Neuro-intensivist	Division of Neurocritical Care, Department of Neurology,	All HIC	Neurocritical Care	***Thematic Analysis of Psychosocial Stressors and Adaptive Coping Strategies Among Informal Caregivers of Patients Surviving ICU Admission for Coma***	The present study therefore sought to use a stress and coping framework to characterize the stressors and coping behaviors of family caregivers of patients with severe acute brain injury (SABI) hospitalized in ICUs and recovering after coma.	Adult	TBI, SAH, ICH	USA	Qualitative	Non stated	Family/Carer/Significant other	30	Interviews (semi-structured, open, in depth)	Thematic analysis	Fereday and Muir-Cochrane (2006)	COREQ	We identified 18 themes within the two theory-driven domains, including ten themes describing practical, social, and emotional stressors experienced by caregivers and eight themes describing the psychological and behavioral coping strategies that caregivers attempted to enact. Nearly all caregivers described using avoidance or distraction as an initial coping strategy to manage overwhelming emotions. Caregivers also expressed awareness of more adaptive strategies (e.g., cultivation of positive emotions, acceptance, self-education, and soliciting social and medical support) but had challenges employing them because of their heightened emotional distress.
Lin-Siegler, X. and Lovett, B. J. and Wang, K. Y. and Hadis, S. and DeGaetano, A. C. and Williamson, T. and Duerr, E. and Levy, E. I.	Mixed	2024	USA	Unclear/not stated	Department of Human Development	All HIC	World Neurosurgery	***Under Pressure: Emotional Reactions to Stress of Neurosurgeons and Neurosurgical Trainees***	to conduct a feasibility study identifying job stressors and emotional responses among neurosurgeons rather than generalize findings across the profession.	N/A	N/A	USA	Qualitative descriptive	Not explicitly stated	Neurosurgeons (inc. trainees/residents)	26	Open ended survey questions	Thematic analysis	Braun and Clarke (2006)	Not reported	Participants identified 3 main stressors: 1) administrative deficiencies; 2) delivering bad news/saving lives; and 3) work-life balance. A low frequency of negative emotional responses was reported, but those reported were mainly high-arousal emotions. Limited prior training in coping strategies was also reported. We also found that residents, fellows, and faculty surgeons reported about work stressors and coping strategies differently.
Neal, C. J. and Durning, S. J. and Dharmapurikar, R. and McDaniel, K. E. and Lad, S. P. and Haglund, M. M.	Mixed	2023	USA	Neurosurgery program director	Division of Neurosurgery	All HIC	Journal of Surgical Education	***From Their Eyes: What Constitutes Quality Formative Written Feedback for Neurosurgery Residents***	to identify themes from the written feedback of SAP operative assessments and to examine if these themes influenced the neurosurgery residents' perception of feedback quality.	N/A	N/A	USA	Mixed methods,	Not explicitly stated	Neurosurgeons (inc. trainees/residents)	2968	Documentary sources	Thematic analysis	Glaser (1965)	Not reported	Qualitative analysis of the written feedback revealed five themes: Non-Specific, Specific General Observations, Key Points, Next Steps, and Independent Practice. Feedback in the Specific General Observations, Key Points, and Independent Practice categories were associated with higher level feedback than leaving the space blank ($p < 0.001$) or writing Non-Specific comments ($p < 0.001$).
Pugazenthi, S. and Johnson, G. W. and Lee, H. D. and Strahle, J. M.	Mixed	2023	USA	Unclear/not stated	Department of Neurological Surgery	All HIC	Journal of Neurosurgery	***Medical student specialty decision-making and perceptions of neurosurgery. Part 2: Role of race/ethnicity***	To evaluate the differences between URM and non-URM medical students and residents in terms of the factors that contribute to specialty decision-making and perceptions of neurosurgery.	N/A	N/A	USA	Mixed methods,	Not explicitly stated	Neurosurgeons (inc. trainees/residents)	18	Interviews (semi-structured, open, in depth)	Grounded theory analysis	Corbin and Strauss (1990)	Not reported	Of 272 respondents, 49.2% were medical students, 51.8% were residents, and 11.0% identified as URM. URM medical students considered research opportunities more than non-URM medical students in specialty decision-making ($p = 0.023$). When specialty decision-making factors were assessed, URM residents less strongly considered the technical skill required ($p = 0.023$), their perceived fit in the field ($p < 0.001$), and seeing people like them in the field ($p = 0.010$) than their non-URM counterparts when making specialty decisions. Within both medical student and resident respondent cohorts, the authors found no significant differences between URM and non-URM respondents in terms of their specialty decision-making being affected by medical school experiences such as shadowing, elective rotations, family exposure, or having a mentor in the field. URM residents were more concerned about the opportunity to work on health equity issues in neurosurgery than non-URM residents ($p = 0.005$). The predominant theme that emerged from interviews was the need for more intentional efforts to recruit and retain URM individuals in medicine and specifically neurosurgery.
Coffey, Weinstein, Cai, et al.	Mixed	2016	USA	Not reported	Center for Study of Chronic Illness and Disability, College of Health and Human Services	All HIC	Journal of Patient Experience	***Identifying and Understanding the Health Information Experiences and Preferences of Individuals With TBI, SCI, and Burn Injuries***	To identify preferred sources of health information and services for persons with traumatic brain injury, spinal cord injury, and burn injury and discover how accessibility could be improved.	Adult	TBI, SCI, burns	USA	Qualitative	Not reported	Patients	33	Interviews (open, unstructured, in-depth, semi-structured, open ended)	Other: The research team created a bank of answers and themes. This bank was developed based on the responses of a random sample of interviews	Not reported	Not reported	Participants' difficulties accessing health information varied by injury type and individually. The majority of respondents found information via the Internet and advocated its use when asked to describe their ideal health information system. Nearly all participants supported the development of a comprehensive care website. When searching for health information, participants sought doctor and support group networks, long-term health outcomes, and treatments specific to their injury.

Coffey, Cassese, Cai, et al.	Mixed	2017	USA	Not reported	Center for the Study of Chronic Illness and Disability	All HIC	Journal of Medical Internet Research	***Identifying and understanding the health information experiences and preferences of caregivers of individuals with either traumatic brain injury, spinal cord injury, or burn injury: A qualitative investigation***	To identify the preferred sources of health information for caregivers supporting individuals with injuries and to explore how access to this information could be improved.	Adult	TBI, SCI, burns	USA	Qualitative	Not reported	Family members, carers, signifcant others	32	Interviews (open, unstructured, in-depth, semi-structured, open ended)	Framework analysis	Richie et al (2003); Gale et al (2012)	Not reported	The caregivers endorsed similar behaviors and preferences when seeking and accessing health information. Medical professionals were the preferred source of information, while ease of access made the Internet the most common avenue to obtain information. The challenges faced by participants were frequently a result of limited support. In describing an ideal health system, participants expressed interest in a comprehensive care website offering support network resources, instructive services about the injury and caregiving, and injury-specific materials.
Colgan, Eddy, Aulet-Leon, et al.	Mixed	2021	USA	Psychologist (incl. clinical, neuro-)	Neurology Department, Oregon Health and Science University	All HIC	British journal of neurosurgery	***Compassion, communication, and the perception of control: a mixed methods study to investigate patients' perspectives on clinical practices for alleviating distress and promoting empowerment during awake craniotomies***	To inquire into clinical practices perceived to mitigate patients' intraoperative distress during awake craniotomies.	Adult	neuro-oncology, epilepsy	USA	Mixed methods	Not reported	Patients	14	Mixed: Interviews (open, unstructured, in-depth, semi-structured, open ended) AND questionnaires	Content analysis	Hsieh & Shannon (2005)	Not reported	Postprocedure interviews revealed very high satisfaction with the awake craniotomy and Anesthesia management and minimal levels of intraoperative pain, anxiety, and distress. The most stressful aspects of the procedure included global recognition of medical diagnosis, anxiety provoked by unfamiliar sights, sounds, and sensations, a perception of a lack of information or misinformation, and long periods of immobility. Important factors in alleviating intraoperative distress included the medical team's ability to promote patient perceptions of control, establish compassionate relationships, address unfamiliar intraoperative sensations, and deliver effective Anesthesia management.
Creutzfeldt, Schutz, Zahuranec, et al.	Mixed	2021	USA	Not reported	Department of Neurology, University of Washington; USA. Cambia Palliative Care Center of Excellence	All HIC	Journal of Palliative Medicine	***Family Presence for Patients with Severe Acute Brain Injury and the Influence of the COVID-19 Pandemic***	To explore the experiences of family members of patients with severe acute brain injury focusing on the impact of family presence in the hospital.	Adult	Mixed ABI	USA	Qualitative	Not reported	Family members, carers, signifcant others	22	Interviews (open, unstructured, in-depth, semi-structured, open ended)	Thematic analysis	Sandelowski 2012	Not reported	Four key themes highlight the role of visitation on family's ability to (1) cope by being at the bedside, (2) protect and advocate for the patient, (3) build trust with clinicians, and (4) receive emotional support in the intensive care unit. After visitation restrictions, families found ways to communicate and support virtually and wished for proactive communication from clinicians.
Degeneffe	Mixed	2015	USA	Social worker (Master of Science in Social Work)	Rehabilitation Counseling Program	N/A	Journal of Rehabilitation	***Planning for an uncertain future: Sibling and parent perspectives on future caregiving for persons with acquired brain injury***	To understand how parents and siblings prepare for the future care and support of their family members with acquired brain injuries.	Adult	Mixed ABI	USA	Survey	Not reported	Family members, carers, signifcant others	60	Open ended questions	Grounded theory analysis/constant comparison/open and axial/selective coding	Bogdan and Biklen (1998); Hodder (1994); Glaser & Strauss (1967)	Not reported	Through a grounded theory approach via the constant comparative method of text analysis, discrete themes were identified through coding open-ended survey responses on parent and sibling feelings about future sibling caregiving responsibilities and preparations for siblings to take on this role. Parent and sibling participants were largely reluctant and not prepared for siblings to assume greater future caregiving responsibilities.
Fraas and Calvert	Mixed	2009	USA	Not reported	University of New Hampshire	All HIC	American Journal of Speech-Language Pathology	***The use of narratives to identify characteristics leading to a productive life following acquired brain injury***	To determine the factors leading to successful recovery and productive lifestyles after acquired brain injury (ABI)	Adult	Mixed ABI	USA	Phenomenology	Not reported	Patients	31	Interviews (open, unstructured, in-depth, semi-structured, open ended)	Phenomenological analysis AND Thematic analysis	Seidman (1998)	Not reported	The following 4 major themes emerged from the interviews: development of social support networks, grief and coping strategies, acceptance of the injury and redefinition of self, and empowerment. The issues raised in these interviews may serve to inspire other survivors and provide them with hope and motivation as they progress through the recovery process. Suggestions on how clinicians can help to facilitate this process are discussed.
Gebhardt, McGehee, Grindel, et al.	Mixed	2011	USA	RN/Nurse	Georgia State University	All HIC	Rehabilitation Nursing	***Caregiver and nurse hopes for recovery of patients with acquired brain injury***	To (1) explore the caregiver's hope for recovery of his or her family member who has experienced an ABI, (2) compare the nurse's hopes for the patient with ABI to those of the caregiver, and (3) identify what caregivers and nurses do to maintain hope for recovery during the rehabilitation process	Mixed	Mixed ABI	USA	Qualitative	Not reported	Family members, carers, signifcant others AND HCPs	35	Mixed: Interviews (open, unstructured, in-depth, semi-structured, open ended) AND questionnaires	Thematic analysis	Miles & Hubermann (1994)	Not reported	his qualitative study validated that in some cases there was a disconnect between caregivers' and nurses' hopes for recovery. Four themes related to the caregiver's maintenance of hope were identified: "the importance of family," "taking one day at a time," "knowing the patient better," and "spiritual strength brings me through." Enhancing the perceptual congruence between nurse and caregiver hope during rehabilitation will ultimately improve patient outcomes.
Gillespie	Mixed	2019	USA	Not reported	College of Integrative Medical Sciences	N/A	The journal of pastoral care & counseling : JPCC	***A Qualitative Pilot Study of Spirituality in Long-term Recovery in Acquired Brain Injury***	To examine of the lived experience of spirituality in recovery in persons with ABI	Adult	Mixed ABI	USA	Qualitative	Not reported	Patients	16	Interviews (open, unstructured, in-depth, semi-structured, open ended) - Singular open-text question	Thematic analysis: Reflexive hermeneutic	Denzin & Lincoln (1994), Gadamer (1975)	Not reported	Studies suggest that patients' spiritual needs are not being met, and chaplains are being underutilized. Trust and a sense of presence are essential to encouraging post-traumatic growth (PTG), and, by fully utilizing the skills of our chaplain colleagues, could improve PTG.
Harrison, Seymann, Imershein, et al.	Mixed	2019	USA	Not reported	Department of Medicine	All HIC	World Neurosurgery	***The Impact of Unmet Communication and Education Needs on Neurosurgical Patient and Caregiver Experiences of Care: A Qualitative Exploratory Analysis***	To describe neurosurgical patient and caregiver perceptions of provider communication, the impact of patient education, and their understanding of information given to them throughout the neurosurgical care trajectory.	Unclear	Mixed ABI	USA	Qualitative	Not reported	Patients AND Family members, carers, signifcant others	43	Focus groups	Content analysis	Schreier (2012); Kyngas (2008)	Not reported	Forty-three patients and caregivers participated in 5 focus groups. 12 coding categories were identified to be associated with patient and caregiver communication and information needs across the neurosurgical care trajectory. Stark variations existed in how patients and caregivers described the quality of communication and patient education they received that affected their satisfaction. Preoperatively, issues arose that included Unmet preoperative information need, Difficulties processing patient education and information, Concerns regarding online information resources. Immediately post- operatively, Unmet inpatient information needs and confusion concerning team-based care was prominent. On discharge, inadequate communication about discharge delays and Unmet information needs at discharge predominated.

Lazaridis, Goldenberg, Mansour, et al.	Mixed	2022	USA	MD/Physician	Departments of Neurology and Neurosurgery, Neurocritical Care Unit	All HIC	World Neurosurgery	***What Does Coma Mean? Implications for Shared Decision Making in Acute Brain Injury***	To understand lay public understandings of the term "coma."	N/A	Mixed ABI; coma	USA	Survey	Not reported	Public/lay	206	Open ended question (one)	Grounded theory analysis/constant comparison/open, axial and/or selective coding (modified)	Tavory & Timmermans (2019)	Not reported	We analyzed 206 unique responses in order to derive emergent lay conceptualizations of coma. The following 4 themes emerged in how respondents understood coma: (1) State descriptive. (2) Marker of injury severity. (3) As in distinction (or lack thereof) from brain death or sleep. (4) Covert consciousness. For each concept, we discuss its salient elements and offer representative quotes.
Lou, Granstein, Wabl, et al.	Mixed	2022	USA	Not reported	Not reported	All HIC	Neurocritical Care	***Taking a Chance to Recover: Families Look Back on the Decision to Pursue Tracheostomy After Severe Acute Brain Injury***	To provide, among a cohort of patients who had undergone tracheostomy after severe acute brain injury (SABI), a voice to the family decision-makers regarding their experiences and retrospective evaluations of the decision.	Adult	Mixed ABI	USA	Qualitative	Not reported	Patients AND Family members, carers, signifcant others	21	Interviews (open, unstructured, in-depth, semi-structured, open ended)	Content analysis (iterative framework)	Attride-Stirling (2001); Srivastava & Hopwood (2009) Hsieh & Shannon (2005)	Not reported	As families reflected on the decision to proceed with a tracheostomy, two themes emerged. First, families did not remember tracheostomy as a choice because the uncertain chance of recovery rendered the certain alternative of death unacceptable or because they valued survival above all and therefore could not perceive an alternative to life-sustaining treatment. Second, families identified a fundamental need to receive supportive, consistent communication centering around compassion, clarity, and hope. When this need was met, families were able to reflect on the tracheostomy decision with peace, regardless of their loved one's eventual outcome.
Masterson, Brady and Miller	Mixed	2019	USA	Not reported	Department of Human-Centered Computing, School of Informatics and Computing	All HIC	Journal of Participatory Medicine	***Informational practices of post acute brain injury patients during personal recovery: Qualitative study***	To investigate (1) the current informational practices and sensemaking processes used by post acute brain injury patients during personal recovery and (2) the potential role of quality-of-life instruments in improving patient awareness of brain injury recovery, advocacy, and involvement in care used outside the clinical context.	Adult	Mixed ABI	USA	Qualitative	Essentialism/ realism	Patients	9	Mixed: Interviews (open, unstructured, in-depth, semi-structured, open ended) AND questionnaires	Thematic analysis	Braun & Clarke (2006)	Not reported	Informational practices of people with brain injury involve data collection, data synthesis, and obtaining and applying the insights to their lifestyles. Participants collected data through structured tools such as spreadsheets and wearable devices but switched to unstructured tools such as journals and blogs as changes in overall progress became more qualitative in nature. Although data collection helped participants summarize their progress better, the lack of conceptual understanding made it challenging to know what to monitor or communicate with clinicians. QoLIBRI served as an education tool in this scenario but was inadequate in facilitating reflection and sensemaking.
Muehlschlegel, Perman, Elmer, et al.	Mixed	2022	USA	MD/Physician	Departments of Neurology, Anesthesiology/Critical Care & Surgery	All HIC	Critical care explorations	***The Experiences and Needs of Families of Comatose Patients After Cardiac Arrest and Severe Neurotrauma: The Perspectives of National Key Stakeholders During a National Institutes of Health-Funded Workshop***	To elicit "on-the-ground" perspectives about the experiences and needs of families of patients with SABI.	Unclear	Mixed ABI; severe acute brain injury; Coma	USA	Qualitative	Not reported	Family members, carers, signifcant others AND HCPs	48	Mixed: Interviews (open, unstructured, in-depth, semi-structured, open ended) AND facilitated workshop discussions	Other: deductive and inductive analysis structured around an Ecological model with five domains adapted from the National Institute on Minority Health and Health Disparities (2008)	not reported	Not reported	Four major needs were identified: 1) challenges in coping with uncertainty in early prognostication, 2) inattention to physical needs of family, 3) deficits in compassionate and consistent communication, and 4) need for engagement with families as stakeholders in improving future practices. Participants' recommendations included: 1) ways to communicate more clearly and consistently, 2) better assistance with navigating resources and access to places for families to care for themselves, and 3) opportunities for families to remain connected with their loved ones, social support networks, and the clinical team.
O'Neil-Pirozzi, Lorenz, Demore-Taber, et al.	Mixed	2015	USA	Not reported	Northeastern University, Spaulding Rehabilitation Hospital and Harvard Medical School	All HIC	Brain Injury	***There will be some changes made: A survivor perspective on post-acquired brain injury residential transition***	To provide a group of chronic acquired brain injury survivors the opportunity to share their insights and experience of transitioning from one type of residential environment to another; and To provide survivor-generated professional caregiver strategies to maximize the chronic acquired brain injury survivor's experience transitioning from one type of residential environment to another and achieving positive transition outcomes based on survivor feedback.	Adult	Mixed ABI	USA	Qualitative	Six 'Transition themes' from previous outcome measurement research	Patients	21	Interviews (open, unstructured, in-depth, semi-structured, open ended)	Other: coded according to prospectively developed six transition themes.	not reported	Not reported	Participants discussed positive and negative insights and experiences regarding residential transitions. Themes of balance between support and independence, life purpose and transition to more or less structure were frequently addressed. Participants suggested caregiver-targeted strategies to facilitate successful transitions before, during and after a move.
Osborne, Juengst and Smith	Mixed	2021	USA	Not reported	Department of Physical Medicine and Rehabilitation	All HIC	British Journal of Occupational Therapy	***Identifying user-centered content, design, and features for mobile health apps to support long-term assessment, behavioral intervention, and transitions of care in neurological rehabilitation: An exploratory study***	To characterize perspectives of individuals with brain injury or stroke, their care partners, and neuro-rehabilitation clinicians to inform the design of mobile health app-based interventions that address the need for ongoing community-based health-related professional support.	Adult	Mixed ABI	USA	Qualitative	Not reported	Patients AND Family members, carers, signifcant others AND HCPs	16	Focus groups	Thematic analysis	Braun & Clarke (2006), Nowell (2017)	SRQR	Five consistent themes emerged: (a) all-in-one app with customized features; (b) communication with healthcare providers; (c) cognitive strategies; (d) app accessibility; and (e) user education. The clinician focus group reiterated these consumer focus group themes, with one additional theme: (f) logistics of clinician time and effort to provide effective app-based services to patients.
Peoples, Boone, Blumenthal-Barby, et al.	Mixed	2020	USA	Not reported	Department of Orthopedic Surgery and Scoliosis; Center for Medical Ethics & Health Policy	All HIC	Journal of Palliative Care	***How Clinician-Family Interactions Potentially Impact Clinicians' Conceptualization and Discussions Regarding Prognostic Uncertainties***	To identify factors that influence how prognostic uncertainty is viewed by physicians, as it relates to their communications with families.	N/A	Neurosurgery	USA	Qualitative	Not reported	HCPs (inc. NSx)	30	Interviews (open, unstructured, in-depth, semi-structured, open ended)	Grounded theory analysis/constant comparison/open and axial/selective coding	Neergaard et al. (2009)	Not reported	We identified 2 main factors that influence how clinicians perceive prognostic uncertainty and their perceptions about whether and why they communicate prognostic uncertainties to families: (1) Communicating Uncertainty to "Soften the Blow"; and (2) Communicating Uncertainty in Response to Clinicians' Interpretations of Surrogate Decision Makers' Perceptions of Prognostic Uncertainty. We also identified several subthemes.

Schutz, Coats, Engelberg, et al.	Mixed	2017	USA	Not reported	Vanderbilt School of Medicine	All HIC	Journal of Palliative Medicine	***Is There Hope? Is She There? How Families and Clinicians Experience Severe Acute Brain Injury***	To explore how family members, nurses, and physicians experience the palliative and supportive care needs of patients with SABI receiving care in the neuroscience intensive care unit (neuro-ICU).	Adult	Mixed ABI; Severe Acute Brain Injury	USA	Qualitative	Not reported	Family members, carers, signifcant others AND HCPs		47	Interviews (open, unstructured, in-depth, semi-structured, open ended)	Thematic analysis: Descriptive	Attride-Stirling (2001)	Not reported	Two themes were identified: (1) hope and (2) personhood. (1) Families linked prognostic uncertainty to a need for hope and expressed a desire for physicians to acknowledge this relationship. The language of hope varied depending on the participant: clinicians used hope as an object that can be given or taken away, generally in the process of conveying prognosis, while families expressed hope as an action that supported coping with their loved one's acute illness and its prognostic uncertainty. (2) Participants described the loss of personhood through brain injury, the need to recognize and treat the brain-injured patient as a person, and the importance of relatedness and connection, including personal support of families by clinicians.
Sohlberg, McLaughlin, Todis, et al.	Mixed	2001	USA	Associate Professor	Communication Disorder & Sciences Program, College of Education	All HIC	Journal of Head Trauma Rehabilitation	***What does it take to collaborate with families affected by brain injury? A preliminary model***	To report our efforts to create an accessible set of collaboration procedures for rehabilitation professionals working in clinical and educational settings with individuals with brain injury and their families	Mixed	Mixed ABI	USA	Participatory action research	Not reported	Family members, carers, signifcant others		8	Mixed: Interviews (open, unstructured, in-depth, semi-structured, open ended) AND observations	Other: categorization strategies, notes were analyzed by arranging discrete sections into larger themes and hypotheses.	Maxwell (1996)	Not reported	A 2-year, qualitative study with eight families led to the development of a preliminary model and prescriptive manual for applying collaborative principles to practice.
Tran, Back and Creutzfeldt	Mixed	2016	USA	Not reported	Harborview Medical Center,	All HIC	Neurocritical care	***Palliative Care Consultations in the Neuro-ICU: A Qualitative Study***	To (1) identify the content and key themes of the palliative care consultation, (2) explore the reason a palliative care consultation was sought, and (3) describe the most prevalent recommendations put forth from the palliative care specialists.	Adult	Neuro-ICU	USA	Qualitative	Not reported	Other	Other: 25		Documentary sources: clinical notes/electronic health records	Content analysis	Not reported	Not reported	Twenty-five neuro-ICU patients (4 %) received a PC consultation over 8 months with the most prevalent reason of clarifying goals of care. The main distinctions between patients with and those without (n = 580) a PC consultation were ICU length of stay (median 8.2 vs. 2.8 days) and death in the neuro-ICU (56 % vs. 11 %). The most prevalent themes addressed in the PC consultation notes were (1) discussing prognosis, (2) eliciting patient and family values, (3) understanding medical options, and (4) identifying conflict.
Waseem, Mazzamurro, Fisher, et al.	Mixed	2018	USA	MD/Physician	Department of Neurology,	All HIC	Journal of Neurosurgery: Pediatrics	***Parental satisfaction with being present in the operating room during the induction of anesthesia prior to pediatric neurosurgical intervention: A qualitative analysis***	To provide a qualitative analysis of parental and patient satisfaction with PPIA at the authors' institution.	Pediatric	Mixed cranial / spinal procedures	USA	Qualitative	Not reported	Family members, carers, signifcant others		42	Interviews (open, unstructured, in-depth, semi-structured, open ended)	Thematic analysis	Not reported	Not reported	The predominant themes identified with PPIA were 1) perception of induction as traumatizing or distressing to witness, 2) positive feelings regarding having been present, 3) satisfaction regarding the overall experience with surgery, 4) variable feelings in parents who decided not to attend induction, and 5) mixed feelings in the interactions with the care team. Parents expressed an array of positive, negative, and neutral impressions of the experience; however, overall, most experiences were positive. Most parents would choose PPIA again if their child required additional surgery.
Gregory, Sarah Kaytlyn and Kemp, Amy M.	Mixed	2024	USA	Other: Female undergraduate student in communication sciences and disorders	Department of Communication Sciences and Special Education,	All HIC	American journal of speech-language pathology	***Experiences of Acquired Brain Injury as Expressed Through Mask-Making: A Qualitative Analysis***	To categorize common emotional themes of people living with ABI that are expressed through mask-making.	Unclear	MIXED ABI	USA, Canada, UK	Qualitative	Non stated	Patients		1049	Other: mask making	Thematic analysis	Braun and Clark (2006)	Not reported	In total, 1,049 masks had narratives describing the visual components and meaning in the associated masks. Three major themes emerged: the multi- plicity of experiences after ABI, including positive, negative, and mixed outcomes; the expression of emotional pain and living with loss through art; and the impor- tance of positivity, purpose, and faith in the experience with ABI. Multiplicity was the central expression represented in the masks. The most commonly repre- sented emotional experiences were: emotional pain and living with loss and posi- tivity, purpose, and faith. The masks incorporated literal depictions of the ABI, visual metaphors, and intentional use of colors and shapes for self-expression.
Cicalese, K. V. and Kruszewski, K. L. and Krishnakumar, A. and Holloway, K. L.	Mixed	2024	USA	Unclear/not stated	School of Medicine	All HIC	Journal of Neurosurgery	***Experiences with pregnancy and child-rearing, advice for future mothers, and suggestions for improving the system: a qualitative study of women in neurosurgery***	Aimed to be the first interview-based study that thematically analyzed the experiences of childbearing and child-rearing by neurosurgeon mothers, as well as their advice for current and aspiring neurosurgeons considering pregnancy and starting a family.	N/A	Gender	USA; CAN	Qualitative	Not stated	Neurosurgeons (inc. trainees/residen ts)		33	Interviews (semi-structured, open, in depth)	Thematic analysis	Stirling (2001)	Not reported	Among the 33 participants, 22 (66.7%) had given birth to or adopted at least one child, had at least one stepchild, or were pregnant at the time of the interview. Three themes emerged regarding these 22 women's experiences with pregnancy and child-rearing: 1) challenges with the physiological changes of pregnancy, 2) feelings of guilt and anxiety, and 3) reliance on loved ones for childcare. Three themes emerged among these 22 women's advice for future mothers in neurosurgery: 1) set realistic expectations, 2) take control of your schedule, and 3) realize that there is no "right" time to start a family. Finally, two themes emerged among all 33 participants' suggestions for making neurosurgery more feasible for pregnancy and child-rearing: 1) revamping of on-site resources, and 2) improved guidance on family planning, childbearing, and maternity leave. The most prominent subtheme in the authors' study was a call for improved on-site daycare under the "revamping of on-site resources" theme, with a particular emphasis on 24/7 operation.
Nicol, C. and Pinkham, M. B. and Lion, K. and Foote, M. and McBean, A. and Higgins, M. and Conlon, E. and Ownsworth, T.	Neuro-oncology	2024	Australia	Other: Clinical psychology PhD candidate	School of Applied Psychology; The Hopkins Centre, Menzies Health Institute of Queensland	All HIC	Neuropsycholo gical Rehabilitation	***Individuals' perceptions of health and well-being in the context of stereotactic radiosurgery for benign brain tumour: A longitudinal qualitative investigation***	To explore individuals' perspectives of their health and well-being prior to and following GKSRS.	Adult	Gamma knife for benign brain tumour	Australia	Qualitative	Not explicitly stated	Patients		20	Interviews (semi-structured, open, in depth)	Thematic analysis	Braun and Clarke (2006); Braun and Clarke (2013)	COREQ	Three major themes characterized individuals' perceptions of their health and well-being. "Understanding my Illness and Treatment" reflected individuals' efforts to make sense of their illness and symptoms to reduce ambiguity and increase sense of control. "Experiencing Gamma Knife" related to expectations of the procedure, outcomes, daily impacts, and emotional reactions. "Adjusting one's Mindset and Coping" characterised how peoples' approaches to coping with their illness were altered over time.
Collins, Lethborg, Brand, et al.	Neuro-oncology	2014	Australia	Not reported	Center for Palliative Care, St Vincent's Hospital Melbourne	All HIC	BMJ Supportive and Palliative Care	***The challenges and suffering of caring for people with primary malignant glioma: Qualitative perspectives on improving current supportive and palliative care practices***	To understand the supportive and palliative care needs of carers of patients with high-grade primary malignant glioma, with a particular focus upon care at the end-of-life, which has hitherto been neglected.	Adult	Neuro-oncology	Australia	Qualitative	Not reported	Family members, carers, signifcant others		23	Interviews (open, in-depth, semi-structured, open ended)	Grounded theory analysis/consta nt comparison/op en, axial and/or selective coding	Glaser & Strauss (1967)	Not reported	Carers described significant needs in relation to three distinct domains: the challenge of caring; the lack of support available to carers and the suffering of caring. The need for care coordination was highlighted as it may enable a series of recommendations for improved care, including: navigation between health providers, individualized, staged information; routine, proactive telephone needs-assessment and emotional support; and early routine integration of palliative care services.

Cornwell, Dicks, Fleming, et al.	Neuro-oncology	2012	Australia	Not reported	Metro North Health Service District, Queensland Health	All HIC	Supportive Care in Cancer	***Care and support needs of patients and carers early post-discharge following treatment for non-malignant brain tumor: Establishing a new reality***	To understand the early post-discharge support services and care requirements of individuals with brain tumor and their family caregivers between short-term (2 weeks) post-discharge and medium-term (3 months) following hospital discharge.	Adult	Neuro-oncology	Australia	Narrative inquiry	Not reported	Patients AND Family members, carers, signifcant others	14	Interviews (open, unstructured, in-depth, semi-structured, open ended)	Content analysis	Graneheim & Lundman (2004)	Not reported	The overarching theme emerging from the data analysis related to patients and carers 'establishing a new reality' underpinned by three primary categories: (1) coping with available supports, (2) adjusting to routines and relationships and (3) emotional responses.
Ownsworth, Chambers, Hawkes, et al.	Neuro-oncology	2011	Australia	Psychologist (incl. clinical, neuro-)	School of Psychology and Griffith Institute for Health and Medical Research	All HIC	Neuropsycholo gical Rehabilitation	***Making sense of brain tumor: A qualitative investigation of personal and social processes of adjustment***	To investigate personal and social processes of adjustment at different stages of illness for individuals with brain tumor	Adult	Mixed brain tumor	Australia	Phenomenology	Not reported	Patients AND Family members, carers, signifcant others	32	Interviews (open, unstructured, in-depth, semi-structured, open ended)	Grounded theory analysis/constant comparison/open, axial and/or selective coding (Thematic)	Liamputtong and Ezzy (2005), Patton (2002)	Not reported	The primary theme that emerged from the analysis entailed "key sense making appraisals", which was closely related to the following secondary themes: (1) Interactions with those in the healthcare system, (2) reactions and support from the personal support network, and (3) a diversity of coping efforts. Adjustment to brain tumor involved a series of appraisals about the illness that were influenced by interactions with those in the healthcare system, reactions and support from people in their support network, and personal coping efforts. Overall, the findings indicate that adjustment to brain tumor is highly individualistic; however, some common personal and social processes are evident in how people make sense of and adapt to the illness over time. A preliminary framework of adjustment based on the present findings and its clinical relevance are discussed. In particular, it is important for health professionals to seek to understand and support individuals' sense-making processes following diagnosis of brain tumor.
Philip, Collins, Brand, et al.	Neuro-oncology	2014	Australia	Not reported	Center for Palliative Care	All HIC	Supportive Care in Cancer	***"I'm just waiting…": An exploration of the experience of living and dying with primary malignant glioma***	To understand patient experience at the end of life and document supportive and palliative care needs.	Adult	Primary malignant glioma (PMG)	Australia	Qualitative	Not reported	Patients	10	Interviews (open, unstructured, in-depth, semi-structured, open ended)	Grounded theory analysis/constant comparison/open, axial and/or selective coding	Glaser (1992)	Not reported	Despite the medical treatment and supportive care available, there remains a gap in services addressing complex existential and psychosocial needs that were markedly valued by patients. Patient experience was characterized by a pervasive loss of all that encompassed their former sense of self and a focus on immediate needs.
Variath, Climans, Edelstein, et al.	Neuro-oncology	2022	Canada	Not reported	Lawrence S Bloomberg School of Nursing	All HIC	Death Studies	***Neuro-oncology clinicians' perspectives on factors affecting brain cancer patients' access to medical assistance in dying: A qualitative study***	To explore neuro-oncology clinicians' perspectives on access to and eligibility for MAiD for patients diagnosed with brain cancer.	Adult	Brain tumors (malignant)	Australia Canada	Qualitative	Relational ethics conceptual framework	HCPs (inc. NSx)	24	Interviews (open, unstructured, in-depth, semi-structured, open ended)	Thematic: Interpretive descriptive analysis	Attride-Sterling (2001), Bell (2020), Thorne et al (1997)	Not reported	Participants described the unique challenges facing brain cancer patients, potentially resulting in their inequitable access to MAiD. The findings highlight the importance of early end-of-life conversations, advance care planning, and access to end-of-life treatment options.
Leal, da Fonseca and Landeiro	Neuro-oncology	2017	Brazil	Not reported	Departamento de Neurocirurgia	All LMIC	Acta Neurochirurgica	***Patients' perspective on awake craniotomy for brain tumors - single center experience in Brazil***	To explore the perception and tolerability of awake craniotomy in an unstudied population of brain tumor patients.	Adult	Awake craniotomy	Brazil	Qualitative	Not reported	Patients	17	Interviews (open, unstructured, in-depth, semi-structured, open ended)	Type unclear	Not reported	Not reported	Patients' thoughts were grouped into five categories: (1) overall perception: no patient considered awake craniotomy a bad experience, and most understood the rationale behind it. They were positively surprised with the surgery; (2) memory: varied from nothing to the entire surgery; (3) nega-tive sensations: in general, it was painless and comfortable. Remarks concerning discomfort on the operating table were made; (4) postoperative recovery: perception of the postoperative period was positive; (5) previous surgical experiences versus awake craniotomy: patients often preferred awake surgery over other surgery under general anesthesia, including craniotomies.
Baba, McCradden, Rabski, et al.	Neuro-oncology	2020	Canada	Not reported	Division of Neurosurgery	All HIC	Neuro-Oncology Practice	***Determining the unmet needs of patients with intracranial meningioma - A qualitative assessment***	This study describes patient-identified limitations to their lives after discovery and treatment of their meningioma, as well as support and limitations amenable to improvement to meningioma patients' experiences of seeking treatment and recovery.	Adult	Meningioma	Canada	Qualitative	Not reported	Patients AND Family members, carers, signifcant other AND HCPs (inc. HCPs)	50	Interviews (open, unstructured, in-depth, semi-structured, open ended)	Grounded theory analysis/constant comparison/open, axial and/or selective coding (Thematic inductive)	Braun & Clarke (2006), Charmaz (2006)	Not reported	Thematic analysis revealed the 'unmet needs of meningioma patients in the post-operative phase' and 4 overarching themes: (1) access to targeted postoperative care, (2) financial struggles for patients and their families, (3) lack of information specific to meningiomas and postsurgical management, and (4) lack of psychosocial support.
Glavassevich, Thomas and Galloway	Neuro-oncology	1989	Canada	RN/Nurse	Department of nursing	n/a	Axone (Dartmouth, N.S.)	***Informational needs of patients who undergo excision of an acoustic neuroma***	To describe the informational needs of individuals who undergo excision of an acoustic neuroma.	Adult	Acoustic neuroma	Canada	Survey	Not reported	Patients	21	Open ended questions	Content analysis	Not reported	Not reported	Content analysis indicated that the majority of subjects experienced tiredness, depression, headache, and dryness of eyes and mouth in the postoperative and convalescent phases. The actual illness experience persisted much longer than the subjects had expected. Subjects expressed explicit informational needs related to self-management after the surgery.
Hayhurst, Mendelsohn and Bernstein	Neuro-oncology	2011	Canada	Not reported	Division of Neurosurgery	All HIC	Canadian Journal of Neurological Sciences	***Low grade glioma: A qualitative study of the wait and see approach***	To explore the impact of low grade glioma diagnosis to address concerns regarding uncertainty of the diagnosis and role of wait and see from the patient's perspective.	Adult	Brain tumors (Low grade glioma)	Canada	Qualitative	Not reported	Patients	24	Interviews (open, unstructured, in-depth, semi-structured, open ended)	Grounded theory analysis/constant comparison/open, axial and/or selective coding (modified)	Kennedy (2006); Strauss & Corbin (1998); Pope, Mays & Ziebland (2000)	Not reported	Analysis of the interview transcripts yielded five overarching themes: Patients expressed initial devastation followed by acceptance and low anxiety; absence of symptoms mitigates anxiety concerning the possibility of progression, Patients would prefer to defer surgery until there is progression or a chance in their quality of life; Anxiety is reduced by trust in the physician; Quality of life is not affected by the diagnosis as fear of morbidity from intervention is greater than the fear of uncertainty
Holliman and Bernstein	Neuro-oncology	2012	Canada	Not reported	Division of Neurosurgery	All HIC	British Journal of Neurosurgery	***Patients' perception of error during craniotomy for brain tumor and their attitudes towards pre-operative discussion of error: A qualitative study***	To investigate patients' perceptions of potential medical error during craniotomy for brain tumor and whether this influenced their decision to consent.	Adult	Brain tumor; craniotomy	Canada	Qualitative	Not reported	Patients	35	Interviews (open, unstructured, in-depth, semi-structured, open ended)	Grounded theory analysis/constant comparison/open, axial and/or selective coding (Thematic)	Strauss & Corbin (1990)	Not reported	Analysis revealed seven overarching themes: (i) views on what constituted medical error were well formed; (ii) to err is human; (iii) protocols exist to prevent error; (iv) trust in one's surgeon is important; (v) patients' belief that they can influence the likelihood of error was variable; (vi) concern with treating the disease trumps worry over possible errors; and (vii) the usefulness of discussing potential error was variable.

Jagadeesh and Bernstein	Neuro-oncology	2014	Canada	Not reported	Division of Neurosurgery	All HIC	Acta Neurochirurgica	***Patients' anxiety around incidental brain tumors: A qualitative study***	To examine how patients are told about their incidental finding as well as anxiety until the neurosurgical consultation and afterward.	Adult	Brain tumor	Canada	Qualitative	Not reported	Patients	32	Interviews (open, in-depth, semi-structured, open ended)	Grounded theory analysis/constant comparison/open, axial and/or selective coding (Thematic)	Hayhurst, Mendelsohn & Bernstein (2011)	Not reported	The level of patient satisfaction for the initial breaking of the news averaged 4.1 (range 1–5). Four themes were identified: (1) emotional stress over incidental findings are partially dependent on how the news was communicated; (2) breaking worrisome news is best done in person, but tele- phone communication can sometimes be acceptable; (3) patients are divided about how much information they wish to get about incidental findings before going for an MRI; (4) waiting for the neurosurgical consultation is a stressful time without adequate support.
Kasper, Hart, Samuel, et al.	Neuro-oncology	2022	Canada	Medical Student	Faculty of Medicine	All HIC	BMC Psychology	***Anxiety and depression in patients with intracranial meningioma: a mixed methods analysis***	To investigate the mental health burden in patients with meningiomas who have undergone surgical resection or serial observation, so as to identify and enhance awareness of gaps in care.	Adult	Intracranial Meningioma	Canada	Mixed methods (explanatory sequential)	Not reported	Patients	8	Mixed: Interview (type unclear) AND questionnaire	Grounded theory analysis/constant comparison/open, axial and/or selective coding (Thematic)	Pope, Van Royen,, Baker (2002)	Not reported	Thirty patients with intracranial meningiomas met inclusion criteria. The cohort's mean age was 56.01 years and 66.67% were women (n = 20). Fourteen underwent surgery; sixteen were treated conservatively with observation. The average time since diagnosis of the sample was 37.6 months. Prevalence of mild to severe symptoms of anxiety was 28.6% amongst surgical management patients and 50% for active surveillance patients (p = 0.325). The prevalence of mild to severe symptoms of depression was 7.14% amongst surgical management patients and 6.25% for active surveillance patients (p = 0.533). Emerging themes from eight interviews reveal the influence of resilience, uncertainty and time, social support, interactions with medical experts, and difficulties during recovery on mental health.
Khu, Doglietto, Radovanovic, et al.	Neuro-oncology	2010	Canada	MD/Physician	Division of Neurosurgery	All HIC	Journal of Neurosurgery	***Patients' perceptions of awake and outpatient craniotomy for brain tumor: A qualitative study - Clinical article***	To explore patients' perceptions about awake and outpatient craniotomy.	Adult	Awake craniotomy/outpatient craniotomy for tumor	Canada	Qualitative	Not reported	Patients	27	Interviews (open, unstructured, in-depth, semi-structured, open ended)	Grounded theory analysis/constant comparison/open, axial and/or selective coding (Modified thematic)	Strauss & Corbin (1990)	Not reported	The following 6 overarching themes emerged from the data: 1) patients had a positive experience with awake craniotomy; 2) patient satisfaction with outpatient surgery was high; 3) patients understood the rationale be- hind awake surgery; 4) patients were surprised that brain surgery can be done on an outpatient basis; 5) trust in one's surgeon was important; and 6) patients were more concerned about the disease than the procedure.
Li, Cashell, Lee, et al.	Neuro-oncology	2020	Canada	Not reported	Radiation Medicine Program; Department of radiation	All HIC	Journal of Medical Imaging and Radiation Sciences	***Patient perspectives on frame versus mask immobilization for gamma knife stereotactic radiosurgery***	To assess patient experiences and perspectives following Gamma Knife (GK) stereotactic radiosurgery (SRS) using frame versus mask immobilization.	Adult	Gamma Knife, stereotactic radiosurgery	Canada	Qualitative (descriptive)	Not reported	Patients	12	Interviews (open, unstructured, in-depth, semi-structured, open ended)	Qualitative descriptive analysis	Parse (2021); Hsieh & Shannon (2005)	Not reported	Fifteen patients were consented; 12 were successfully interviewed (3 lost due to deteriorating health status). Interviews ranged from 30 to 60 min in duration. The most common patient concern regarding the frame was pain (9 patients), while the primary concerns with the mask system were the ability to remain still (6 patients) and claustrophobia (4 patients). Eleven patients chose the mask as their preferred choice in terms of their overall experience. Two themes emerged during the interviews that spoke to patient satisfaction with each process: unexpected pain with frame placement; and tightness experienced while wearing the mask during treatment.
Lipsman, Zener and Bernstein	Neuro-oncology	2009	Canada	Neurosurgical Resident	Division of Neurosurgery	All HIC	Bioethics	***Personal identity, enhancement and neurosurgery: A qualitative study in applied neuroethics***	1) to gauge the attitudes of neurosurgery patients towards identity change as a result of surgery, 2) to examine whether identity change is a concern for patients pre-operatively, and 3) to examine general patient attitudes towards surgery to treat psychiatric disease as well as surgery to alter maladaptive and normal human traits.	Adult	Gamma Knife Radiosurgery; DBS	Canada	Qualitative	Not reported	Patients	27	Interviews (type unclear)	Grounded theory analysis/constant comparison/open and axial/selective coding (Modified thematic)	Strauss & Corbin (1998)	Not reported	Themes: 1.Fear of identity loss, prior to both maximally and minimally invasive procedures, is not a concern for patients about to undergo neurosurgical operations. 2. When choosing between a threat to identity and a threat to physical existence, patients will almost always choose to prolong physical existence despite the risk to personal identity or personality. 3.Given sufficiently stringent controls, surgery on the brain to control treatment refractory psychiatric disease is viewed as ethical by most patients. 4.Surgery with the explicit aim to alter identity, modify or enhance either normal or maladaptive traits is viewed as ethically dubious. The correction of a personality 'defect' however, is viewed as more acceptable than a change to an already normal personality. 5.Even when participating in procedures that involve a significant risk to body and mind, patients believe that individual patient choice should be respected if informed consent is complete.
McCradden, Baba, Saha, et al.	Neuro-oncology	2020	Canada	Post-doctoral research fellow	Division of Neurosurgery ,	All HIC	CMAJ open	***Ethical concerns around use of artificial intelligence in health care research from the perspective of patients with meningioma, caregivers and health care providers: a qualitative study***	To investigate current perspectives on ethical issues surrounding artificial intelligence (AI) in health care.	Adult	Meningioma	Canada	Qualitative	Not reported	Patients AND Family members, carers, signifcant other AND HCPs (inc. NSx)	30	Interviews (vignette based)	Content analysis	Hsieh & Shannon (2005)	Not reported	We interviewed 30 participants: 18 patients, 7 caregivers and 5 health care providers. For each question, a variable number of responses were recorded. The majority of participants endorsed nonconsented use of health data but advocated for disclosure and transparency. Few patients and caregivers felt that allocation of health resources should be done via computerized output, and a majority stated that it was inappropriate to delegate such decisions to a computer. Almost all participants felt that selling health data should be prohibited, and a minority stated that less privacy is acceptable for the goal of improving health. Certain caveats were identified, including the desire for deidentification of data and use within trusted institutions.
Mir, Dirks, Mason, et al.	Neuro-oncology	2014	Canada	Not reported	Division of Neurosurgery	All HIC	Acta Neurochirurgica	***Are patients open to elective re-sampling of their glioblastoma? A new way of assessing treatment innovations***	To examine patient acceptability of re-sampling surgery for glioblastoma multiforme (GBM) electively post-therapy or at asymptomatic relapse.	Adult	Brain tumors (Glioblastoma)	Canada	Qualitative	Not reported	Patients	30	Interviews (open, unstructured, in-depth, semi-structured, open ended)	Grounded theory analysis/constant comparison/open, axial and/or selective coding (Modified thematic analysis)	Strauss and Corbin (1990)	Not reported	The results of the study suggest that about two thirds of the patients offered the surgery on a routine basis would be interested, and half of the patients would agree to the surgery as part of a clinical trial. Several overarching themes emerged, some of which include: patients expressed ethical concerns about offering financial incentives or compensation to the patients or surgeons involved in the study; patients were concerned about appropriate communication and full disclosure about the procedures involved, the legalities of tumor ownership and the use of the tumor post-surgery; patients may feel alone or vulnerable when they are approached about the surgery; patients and their families expressed immense trust in their surgeon and indicated that this trust is a major determinant of their agreeing to surgery.

Mir and Bernstein	Neuro-oncology	2016	Canada	Not reported	Division of Neurosurgery	All HIC	British Journal of Neurosurgery	***Are neurosurgeons prepared to electively resample glioblastoma in patients without symptomatic relapse? A qualitative study***	To examine neurosurgeons' and neuro-oncologists' perceptions of resampling surgery for glioblastoma multiforme electively, post-therapy or at asymptomatic relapse	N/A	Brain tumors (Glioblastoma)	Canada	Qualitative	Not reported	HCPs (inc. NSx)	30	Interviews (open, unstructured, in-depth, semi-structured, open ended)	Grounded theory analysis/constant comparison/open, axial and/or selective coding (Modified thematic analysis)	Strauss and Corbin (1990)	Not reported	Over half of the participants were interested in doing this within a clinical trial. About a quarter of the participants would be willing to consider routine resampling surgery if: (1) a resection were done rather than a simple biopsy; (2) they could wait until the patient becomes symptomatic and (3) there was a preliminary in vitro study with existing tumor samples to be able to offer patients some trial drugs. The remaining quarter of participants was entirely against the trial. Participants also expressed concerns about resource allocation, financial barriers, possibilities of patient coercion and the fear of patients' inability to offer true informed consent.
Ravishankar and Bernstein	Neuro-oncology	2014	Canada	Not reported	Division of Neurosurgery	All HIC	Journal of Religion and Health	***Religion Benefiting Brain tumor Patients: A Qualitative Study***	Is religion as a coping mechanism beneficial for patients before, during and after craniotomy?	Adult	Benign or malignant brain tumor	Canada	Case study	Not reported	Patients	36	Interviews (open, unstructured, in-depth, semi-structured, open ended)	Grounded theory analysis/constant comparison/open, axial and/or selective coding (Thematic)	Bernstein et al (2004)	Not reported	4 themes emerged: 1) Religion significantly benefitted neurosurgical patients 2) neurosurgical patients did not require a dedicated religious room in the hospital 3) neurosurgical patients required religious resources such as leaders and/or groups 4) patients were not in favor of their physician engaging in the religious ritual. Most patients found religion to be an effective coping mechanism, offering them strength, comfort and hope through the surgery.
Rozmovits, Khu, Osman, et al.	Neuro-oncology	2010	Canada	Not reported	Division of Neurosurgery,	All HIC	Journal of Neuro-Oncology	***Information gaps for patients requiring craniotomy for benign brain lesion: A qualitative study***	To determine the information needs of a subset of neurosurgical patients.	Adult	Mixed ABI; Benign brain lesion; (tumor, AVM, unruptured aneurysm); Craniotomy	Canada	Case study	Not reported	Patients	25	Interviews (open, unstructured, in-depth, semi-structured, open ended)	Grounded theory analysis/constant comparison/open, axial and/or selective coding	Strauss & Corbin (1998) and Pope, Mays, Ziebland, (2000)	Not reported	Six overarching themes emerged from the data: (1) the amount of information patients want varies; (2) the type of information needed is not limited to information about treatment options and risks; (3) patients engage in independent information seeking for a variety of reasons; (4) patients consider compassion from their surgeon as important; (5) direct communication with the surgeon post-operatively is very important; and (6) patients' information needs are greatest post-operatively. Many patients felt that the amount and quality of information they received was not sufficient, particularly regarding post-operative recovery and long-term life issues, leading many to do their own research.
Vierhout, Daniels, Mazzotta, et al.	Neuro-oncology	2017	Canada	Not reported	Toronto Western Hospital	All HIC	Current Oncology	***The views of patients with brain cancer about palliative care: A qualitative study***	To explore the thoughts of brain cancer patients about palliative care, their opinions about early palliative care, and their preferred care setting.	Adult	Brain tumor	Canada	Grounded theory	Not reported	Patients	39	Interviews (open, unstructured, in-depth, semi-structured, open ended)	Grounded theory analysis/constant comparison/open, axial and/or selective coding	Strauss & Corbin (1998)	Not reported	Patients would prefer to receive palliative care in the home. Increased time with caregivers and family are the main appeals of home care. Patients express dissatisfaction with brief and superficial interactions with health care providers. Patients believe that palliative care can contribute to their emotional well-being. Patients are open to palliative care if they believe that it will not diminish optimism. There is a preconceived idea that palliative care is directly linked to active dying, and that supposed link generates fear in some patients. Patients prefer to be educated about palliative care as an option early in their illness, even if they are fearful of it
Wong, Mendelsohn, Nyhof-Young, et al.	Neuro-oncology	2011	Canada	Not reported	Division of Neurosurgery	All HIC	Supportive Care in Cancer	***A qualitative assessment of the supportive care and resource needs of patients undergoing craniotomy for benign brain tumors***	To evaluate the supportive care and resource needs of patients undergoing craniotomy for benign brain tumors.	Adult	Benign brain tumor; Craniotomy	Canada	Qualitative	Not reported	Patients	29	Interviews (open, unstructured, in-depth, semi-structured, open ended)	Thematic analysis	Pope, van Royen & Baker (2002)	Not reported	Twenty-nine patients (20 women, 20-88 years of age) with World Health Organization grade I brain tumors (25 meningioma) were interviewed. Five overarching themes emerged: (1) need for formal support from diagnosis onwards; (2) complexity of supportive needs during postoperative recovery; (3) importance of regular long-term monitoring by physicians; (4) influence of psychosocial factors on supportive needs; and (5) existence of barriers to equal access to available supports.
Wyness, Durity, and Durity	Neuro-oncology	2002	Canada	RN/Nurse	School of Nursing,	All HIC	Axone	***Narratives of patients with skull base tumors and their family members: lessons for nursing practice***	To examine patients' and family members' perceptions of their pre-operative education and information need related to surgery for a skull base tumor.	Adult	Skull base tumors	Canada	Qualitative (descriptive)	Not reported	Patients AND Family members, carers, signifcant others	33	Interviews (type unclear)	Content analysis AND secondary narrative analysis	Not reported	Not reported	Patients and family members portrayed experiences, revealed emotions related to hearing the news, and shared ways of coping. Interviews with patients and their family members that contained clear narratives were identified. From these interviews, three sets of narratives were selected. Each of the narratives was analyzed to determine how the patients and their key family members articulated and made sense of the diagnosis and surgical treatment of a skull base tumor. The themes of a sense of comfort, the known is better than the unknown, waiting for news of the surgical outcome, and quality of recovery emerged from the analysis. Lessons learned about the individuality of patients' and family members' needs and approaches to support effective coping were identified.
Yu and Bernstein	Neuro-oncology	2011	Canada	Not reported	Division of Neurosurgery	All HIC	Journal of Neuro-Oncology	***Brain tumor patients' views on deception: A qualitative study***	To explore neuro-oncology patients' attitudes towards dilemmas in which they may feel deceived, and with that information make recommendations on what steps physicians can take to avoid breaking that trust.	Unclear	Brain tumor	Canada	Case study	Not reported	Patients	32	Interviews (open, unstructured, in-depth, semi-structured, open ended)	Grounded theory analysis/constant comparison/open, axial and/or selective coding (Modified thematic)	Strauss & Corbin (1990)	Not reported	Five prominent themes arose from the analysis: (1) patients are hesitant about trainees working on their case, but they are more open to it if they expect the occurrence ahead of time; (2) patients wish to know the exact details when an error has occurred, even if it is of inconsequential effect for them; (3) patients generally prefer to know exactly what the doctor knows, even if nothing can be changed; (4) patients expect physicians to provide them with all the options and resources available; and (5) there are special cases in which patients accept a delay in knowing. Most neuro-oncology patients trust their physicians to make the best decisions for them, but that does not mean they would accept subtle forms of deception. Patients prefer to have all the information necessary in order to make their own decision.
Bernstein, Potvin and Martin	Neuro-oncology	2004	Canada	Neurosurgeon	Division of Neurosurgery	All HIC	Canadian Journal of Neurological Sciences	***A qualitative study of attitudes toward error in patients facing brain tumor surgery***	To determine what patients' views are toward the existence of medical error and how it might affect them.	Adult	Brain tumor	Canada	Qualitative	Not reported	Patients	30	Interviews (open, in-depth, semi-structured, open ended)	Grounded theory analysis/constant comparison/open, axial and/or selective coding (Modified thematic analysis)	Strauss and Corbin, (1998)	Not reported	Three overarching themes emerged from the data: 1) trust in the patient's surgeon was of paramount importance; 2) patients' views toward medical error varied between fear and vulnerability to no concern; and 3) discussion of error was felt to be beneficial as it could help the medical profession decrease future errors and could help dispel the patient's fear and anxiety about the upcoming surgery.

Bramall and Bernstein	Neuro-oncology	2014	Canada	Not reported	Division of Neurosurgery	All HIC	Canadian Journal of Neurological Sciences	***Improving information provision for neurosurgical patients: A qualitative study***	To examine the information-seeking patterns of patients and suggest ways to optimize the communication of medical information, specifically within the context of neurosurgery.	Adult	Benign or malignant tumors	Canada	Qualitative	Not reported	Patients	31	Interviews (open, unstructured, in-depth, semi-structured, open ended)	Grounded theory analysis/constant comparison/open, axial and/or selective coding	Strauss & Corbin 1998	Not reported	Three major themes relating to information-seeking by neurosurgical patients were identified: 1) almost all patients searched for information on the Internet; 2) in addition to characterizing the tumor as benign or malignant, patients sought additional information such as the location of the tumor in the brain; and 3) patients with malignant tumors were less likely to seek information online and more likely to consider alternative therapies. To improve the provision of information to neurosurgical patients, physicians can 1) offer to review imaging results with patients; 2) promote an environment open to questions; 3) provide information in a forthright manner, avoiding the use of medical jargon; and 4) consider guiding patients to reliable Internet sites and facilitating written records of consultations.
Du, K. and Pan, Y. Y. and Yan, W. W.	Neuro-oncology	2023	China	Unclear/not stated	Emergency Center, The First Affiliated Hospital of Zhengzhou University	All LMIC	Journal of Pediatric Nursing-Nursing Care of Children & Families	***Qualitative descriptive study during the COVID-19 pandemic on the needs of informal caregivers of Chinese minors who underwent craniopharyngioma surgery***	to investigate the needs of informal caregivers of children with craniopharyngioma following cerebral surgery	Paediatric	Craniopharyngioma	China	Qualitative descriptive	Non stated	Family/Carer/Significant other	21	Interviews (semi-structured, open, in depth)	Content analysis (Inductive)	Fereday & Muir-Cochrane (2006); Moser & Korstjens (2018)	Not reported	Four themes and 14 sub-themes were extracted from the results of the study, including needs for relieving psychological stress (including psychological pressure on both minors and on caregivers); requirement for on-campus assistance (physical activity, eliminating verbal violence in schools, special education needs for child, healthcare services provided by school hospitals); demands for medical help (acquiring medical knowledge, need for medication management, convenience and reliable access to medical services, need for technological development, expectations of multidisciplinary cooperation, the necessity of health review reminders); and the desire for financial aid (charity platform assistance, inclination of government policy).
Maier, A. D. and Nordentoft, S. and Mathiesen, T. and Guldager, R.	Neuro-oncology	2024	Denmark	Unclear/not stated	Department of Neurosurgery	All HIC	Palliative & Supportive Care	***The experience of living with malignant meningioma***	to explore how patients perceived quality of daily life after a malignant meningioma diagnosis.	Adult	Malignant meningioma	Denmark	Qualitative	Not explicitly stated	Patients	8	Interviews (semi-structured, open, in depth)	Thematic analysis	Braun and Clarke (2006)	Not reported	Eight patients were interviewed. The analysis revealed 4 overarching themes: (1) perceived illness and cause of symptoms, (2) identity, roles, and interaction, (3) threat and uncertainty of the future, and (4) belief in authority. The perceived quality of daily life is negatively impacted by the disease. Patients experience a shift in self-concept and close interactions, and some struggle with accepting a new everyday life. Patients have a high risk of discordant prognostic awareness in relation to health-care professionals.
Brennum, Maier, Almdal, et al.	Neuro-oncology	2015	Denmark	Not reported	Copenhagen Neurosurgery	All HIC	Acta neurochirurgica	***Primo non nocere or maximum survival in grade 2 gliomas? A medical ethical question***	To explore whether patients and experts universally share the primo non nocere dogma, with the paramount importance ascribed to safeguarding neurological functions.	Adult	Brain tumors (Grade 2 Glioma)	Denmark	Qualitative	Not reported	Patients AND HCPs (inc. NSx) AND ethics expert	15	Focus groups	Other: exploratory and open-ended approach	MacNaghten & Myers (2004)	Not reported	Both patients and experts accepted the premise of balancing neurological function versus longevity. Some patients would accept an increased risk of permanent neurological deficits in order to obtain a chance of increased survival. There was a significant variance in what constituted "quality of life" both between patients and for the individual patient over time.
Guldager, Hansen and Ziebell	Neuro-oncology	2021	Denmark	Not reported	Department of Neurosurgery	All HIC	Acta Neurochirurgica	***Past, present and future, the experience of time during examination for malignant brain tumor: a qualitative observational study***	To gain deeper insight into the perception of time from the perspective of patients with brain cancer as they pass through the Danish Integrated Brain Cancer Pathway at a university hospital in Denmark.	Unclear	Brain tumors (malignant);	Denmark	Qualitative (descriptive)	Not reported	Patients AND Family members, carers, signifcant others	6	Observations (Shadowing)	Content analysis	Graneheim & Lundman (2004)	Not reported	Through one constructed case, three perspectives of time were identified. The patient's perception of time during his illness, the healthcare system's perception of time and, finally, an ethical time perspective. The analysis showed a discrepancy between patients' and healthcare professionals' perception of time. Furthermore, the results revealed an ethical time dimension.
Vedelø, Sørensen, and Delmar	Neuro-oncology	2018	Denmark	RN/Nurse	Department of Neurosurgery	All HIC	Journal of Clinical Nursing	***Patients' experiences and care needs during the diagnostic phase of an integrated brain cancer pathway: A case study***	To identify and describe patients' experiences and care needs throughout the diagnostic phase of an integrated brain cancer pathway.	Adult	Brain tumor	Denmark	Case study	Not reported	Patients	4	Mixed: Interviews (open, unstructured, in-depth, semi-structured, open ended) AND observations	Phenomenological analysis (Systematic text condensation)	Malterud (2012, 2017)	Not reported	Four major themes were identified: information needs, balancing hope and reality while trying to perceive the unknown reality of brain cancer, not knowing what to expect and participants' perceptions of the relationship with the healthcare providers. The analysis revealed that participants were in risk of having unmet information needs and that contextual factors seemed to cause fragmented care that led to feelings of uncertainty and loss of control.
Eydoux, Castinetti, Authier, et al.	Neuro-oncology	2021	France	Not reported	Gynecology-Obstetric and Reproductive Medicine	All HIC	Clinical Endocrinology	***Women's perceptions of femininity after craniopharyngioma: a qualitative study***	To assess the impact of a craniopharyngioma history on femininity and relationships in women.	Adult	Craniopharyngioma	France	Grounded theory	Inductive	Patients	15	Interviews (open, unstructured, in-depth, semi-structured, open ended)	Grounded theory analysis/constant comparison/open, axial and/or selective coding	Lejeune (2014)	Not reported	Three main themes were identified: (a) apparent changes leading to altered self-perception that may impact on femininity and generate lower self-esteem; (b) managing the hidden disabilities of the disease inducing a need for permanent control; and (c) building parenthood and couple relationships: coping with sexual dysfunction and infertility. The study highlighted alterations in self-perception and femininity due to body change and disability resulting from CP treatment, impacting both couple and social relationships. Interviewing women who underwent CP surgery at different ages highlighted specific needs and different expectations of medical professionals which emphasize the importance of offering both global and personalized care
Völz, D. and Grabenweger, R. and Best, M. C. and Hau, P. and Jones, K. F. and Linker, R. and Paal, P. and Bumes, E.	Neuro-oncology	2024	Germany	Medical student	Department of Neurology and Wilhelm Sander-NeuroOncology Unit	All HIC	Supportive Care in Cancer	***"Not me!" a qualitative, vignette-based study of nurses' and physicians' reactions to spiritual distress on neuro-oncological units***	to analyze the attitudes and behavior of nurses and physicians when confronted with spiritual distress in these patients.	Unclear	Primary malignant brain tumour	Germany	Survey	Not explicitly stated	HCPs (Inc NSx)	143	Survey (Vignette-based survey)	Reflexive thematic analysis	Braun & Clarke (2022)	Not reported	A total of 143 nurses and physicians working in neurological and neurosurgical wards in 46 hospitals participated in the survey. The participants questioned if the ability to provide spiritual care can be learned or is a natural skill. Spiritual care as a responsibility of the whole team was highlighted, and the staff reflected on the appropriate way of involving spiritual care experts. The main limitations to spiritual care were a lack of time and not viewing spiritual engagement as part of the professional role. Some were able to personally benefit from spiritual conversations with patients, but many participants criticized the perceived emotional burden while expressing the imminent need for specific training and team reflection.
Raju and Krishna Reddy	Neuro-oncology	2018	India	Not reported	Department of Psychiatric Social Work, National Institute of Mental Health and Neurosciences	All LMIC	Indian Journal of Palliative Care	***Perspectives of glioblastoma patients on death and dying: A qualitative study***	To explore GBM patients perspectives on death and dying during admission	Adult	Brain tumors (Glioblastoma)	India	Qualitative	Not reported	Patients	31	Interviews (open, in-depth, semi-structured, open ended)	Thematic analysis	Not reported	Not reported	Four themes: Understanding about illness, personal views and feelings on death and dying, coping with fear of death and dying, need for early preparation to face death and dying fears. There was poor understanding about illness from patients, and unmet psychosocial needs. They recommend early death preparation programs.

Mansur, Oswari, Perdana Wahjoepramono, et al.	Neuro-oncology	2018	Canada	Not reported	Faculty of Medicine	Mixed HIC/LMIC	World Neurosurgery	***Awake Craniotomy in a Low- to Middle-Income Country: A Sustainability Analysis***	To evaluate the sustainability of teaching awake craniotomy in Indonesia, where the first mission was done 14 years ago, using a mixed-methods approach.	N/A	Brain tumor; awake craniotomy, Education	Indonesia	Mixed methods (Questionnaires ; Qualitative)	Not reported	NSx (Neurosurgeons , neurosurgical residents)		33	Mixed: Interviews (open, unstructured, in-depth, semi-structured, open ended) AND questionnaires	Other: axial coding and grouped into themes	Strauss & Corbin (1998)	Not reported	Thirty-three of 88 respondents (41.3%) indicated that they still perform awake craniotomy. Although 87.3% felt that it was beneficial for patients and resource sparing, less than a quarter of them felt they had sufficient exposure/training in awake craniotomy. Almost all of them wanted further training and to maintain a relationship with international mentors. Four themes emerged about the factors that affect how we teach awake craniotomy in an LMIC: 1) the sustainability of a teaching mission is dependent on a culture of information sharing and 2) the support of multiple health care providers; 3) hospital structure affects how changes are implemented; and 4) health care professionals in Indonesia value opportunities for international training. Regarding the uptake of awake craniotomy in Indonesia, there are sociocultural factors that affect patients' receptiveness to surgery and the national insurance plan restricts the provision of neurosurgical care.
Hamidi, Karmur, Sperrazza, et al.	Neuro-oncology	2020	USA	Not reported	Arizona College of Osteopathic Medicine	All HIC	Journal of Neurosurgery	***Guidelines for optimal utilization of social media for brain tumor stakeholders***	To establish platform-specific social media guidelines by investigating how stakeholders in the brain tumor community utilize Facebook, Twitter, and YouTube	Unclear	Brain tumor	International	Mixed methods (Descriptive statistics; Qualitative)	Not reported	Other	Other: 50 most recent posts on Facebook and Twitter		Social Media Content: Facebook, Twitter, YouTube	Thematic analysis	Not reported	Not reported	Facebook (67 pages and 304,581 likes) was predominantly used by organizations (64% of pages). Top themes on Facebook, Twitter, and YouTube were charity and fundraising (67% of pages), education and research (72% of accounts), and experience sharing and support seeking (48% of videos, 60% of views, and 82% of user engagement), respectively. On Facebook, only the presence of other concurrent platforms influenced a page's performance (rho = 0.59) and popularity (rho = 0.61) ($p < 0.05$). On Twitter, the number of monthly tweets (rho = 0.66) and media utilization (rho = 0.78) were significantly correlated with increased popularity and performance (both $p < 0.05$). Personal YouTube videos (30% of videos and 61% of views) with the theme of experience sharing and support seeking had the highest level of engagement (60% of views, 70% of comments, and 87% of likes).
Lanini, Tringali and Lauro Grotto	Neuro-oncology	2022	Italy	Psychologist (incl. clinical, neuro-)	Department of Health Sciences	All HIC	Frontiers in Psychology	***Psychological Needs and Resources of the Staff in a Pediatric Neurosurgery Ward: A Phenomenological-Hermeneutic Study***	To explore experiences of the medical professionals engaged in the cure of children with brain tumors, as well as their families, across different phases of the treatment (diagnosis, active treatment, relapse, transition to terminality, or remission).	Pediatric	Brain tumor	Italy	Phenomenology	Hermeneutic phenomenology	HCPs (Neurosurgical unit staff inc. surgeons)		23	Interviews (open, unstructured, in-depth, semi-structured, open ended)	IPA	Mantovani and Spagnolli, (2003); Smith, (2003); Reid et al., (2005)	COREQ	A complex picture of personal, professional as well as organizational, and institutional demands emerge from the data. The first overarching themes was: The psychological needs expressed by the participants with the following subthemes: need to receive psychological support for staff and for the patients, need for frankness, need for emotional boundaries, need for clinical and ethical boundaries, need to leave room for hope, need to express sadness. The second overarching theme was: The resources and motivational drives experienced by the participants with the following subthemes: Acknowledgement and safeguard mechanisms within the equipe dynamics, the chance to learn from experience, the "fundamental childhood", knowing that the bond made was not vain.
Palese, Skrap, Fachin, et al.	Neuro-oncology	2008	Italy	RN/Nurse	School of Nursing,	All HIC	Cancer Nursing	***The experience of patients undergoing awake craniotomy: In the patients' own words. A qualitative study***	To reveal participants' experiences before, during, and immediately after awake craniotomy.	Adult	Brain tumor; craniotomy	Italy	Phenomenology	Not reported	Patients		21	Interviews (open, unstructured, in-depth, semi-structured, open ended)	Phenomenological analysis	Streubert, Streubert and Carpenter (2003); Silverman (2001); Sandelowski & Barroso (2002)	Not reported	Awake craniotomy is a complex andsubjective experience, the behavioral patterns of the subjects interviewed canbe attributed to the instinct of self-preservation, the ability to participate duringthe procedure helping the surgeon in avoiding brain damage; to be in controlof the situation; and to reassure themselves and others. It is most importantfor a healthcare team to understand the patients' experiences. Immediatelybefore and after surgery, the patients seem to concentrate more on keepingtheir emotions in check and focus on the risk of subsequent defect or disability,rather than on the brain cancer and what it may imply on life. During the surgery,they become particularly involved in the task: they feel directly responsiblefor the results of surgery relating this to the effectiveness of collaboration withthe neurosurgeons.
Neve, Soulier, Hendriksma, et al.	Neuro-oncology	2021	The Netherlands	MD/Physician	Department of Otorhinolaryngology and Head and Neck Surgery	All HIC	European Archives of Oto-Rhino-Laryngology	***Patient-reported factors that influence the vestibular schwannoma treatment decision: a qualitative study***	To identify factors that influence a patient's decision for a particular management strategy.	Adult	Vestibular schwannoma	Netherlands	Qualitative	Not reported	Patients		18	Interviews (open, unstructured, in-depth, semi-structured, open ended)	Framework analysis	Smith & Firth (2011); Gale at al., (2013)	SRQR	Ten themes were identified that influenced the decision, classified as either medical or patient-related. The medical themes that emerged were: tumor characteristics, the physician's recommendation, treatment outcomes and the perceived center's experience. The patient-related themes were: personal characteristics, anxiety, experiences, cognitions, logistics and trust in the physician.
Dinka, Nyce and Timpka	Neuro-oncology	2009	Sweden	Not reported	Department of Computer and Information Science	All HIC	Artificial Intelligence in Medicine	***Situated cognition in clinical visualization: The role of transparency in Gamma Knife neurosurgery planning***	The aim of this study was to investigate how the clinical use of visualization technology can be advanced by the application of a situated cognition perspective.	N/A	Gamma Knife Radiosurgery	Sweden	Qualitative	The basic theoretical grounding employed was mainly Barley's and Wenger's work	Other: NSx AND physicists		6	Mixed: Field observations; AND Semi-structured interviews	Inductive	Not reported	Not reported	The users' ability to perform cognitive tasks was found to be reduced each time visualizations incongruent with the particular user's perception of clinical reality were used. The main issue here was a lack of transparency, i.e. a black box problem where machine representations "stood between" users and the cognitive tasks they wanted to perform. For neurosurgeons, transparency meant their previous experience from traditional surgery could be applied, i.e. that they were not forced to perform additional cognitive work. From the view of the physicists, on the other hand, the concept of transparency was associated with mathematical precision and avoiding creating a cognitive distance between basic patient data and what is experienced as clinical reality. The physicists approached clinical visualization technology as though it was a laboratory apparatus-one that required continual adjustment and assessment in order to "capture" a quantitative clinical reality.
Ung, Olofsson, Björkman, et al.	Neuro-oncology	2019	Sweden	Not reported	Institute of Health and Care Sciences; Center for Person-Centered Care; Department of Medicine	All HIC	Endocrine Connections	***The pre-and postoperative illness trajectory in patients with pituitary tumors***	To explore patient experiences during pre- and postoperative care and recovery after pituitary surgery in patients with a pituitary tumor.	Adult	Pituitary tumors	Sweden	Qualitative	Interpretive approach	Patients		16	Interviews (open, in-depth, semi-structured, open ended)	Other: systematic cycle of four steps.	Fleming, Gaidys, & Robb (2003)	Not reported	Suffering a pituitary tumor was overwhelming for many patients and struggling with existential issues was common. Patients expressed loneliness and vulnerability before and after surgery. How professionals handled information in connection with diagnosis greatly affected the patients. Other patients with the same diagnosis were experienced as the greatest support. Normalization of bodily symptoms and relationships with others were reported during postoperative recovery. However, a fear that the tumor would return was present.

Wideheim, Edvardsson, Tåhlson, et al.	Neuro-oncology	2002	Sweden	RN/Nurse	Department of Caring Sciences	All HIC	Cancer Nursing	***A family's perspective on living with a highly malignant brain tumor***	To describe what it like to live with a highly malignant brain tumor from a family perspective	Adult	Brain tumors (malignant);	Sweden	Qualitative	Not stated	Patients AND Family members, carers, signifcant others	8	Interviews (conversational style)	Content analysis: Inductive	Berelson (1971), Baxter (1991), Polit & Hungler (1995)	Not reported	The present work is an introductory study in a research project concerning the consequences of living with a brain tumor for the patient and the next of kin. The results confirm those of earlier studies that falling ill with a highly malignant brain tumor causes a drastic change in the patterns and routines of daily life. Roles and relations within the family are greatly affected. The results indicate that the family experiences grief and anxiety and a fear of death, as described earlier in the literature. The results may increase the understanding of the families' problems and their strategies for coping with learning the diagnosis and many problems in daily life. To provide a basis for a care program involving a family perspective, future studies will include more families and will cover the entire course of the illness
Nollen, J. M. and Brunsveld-Reinders, A. H. and Peul, W. C. and van Furth, W. R.	Neuro-oncology	2023	The Netherlands	Unclear/not stated	Department of Neurosurgery	All HIC	Bmj Open	***Patient perspectives on indwelling urinary catheters and fluid balances after transsphenoidal pituitary surgery: a qualitative study***	To explore the perceptions and experiences of patients who underwent transsphenoidal pituitary gland and (para)sellar tumour surgery regarding indwelling urinary catheters (IDUCs) and the postoperative fluid balance.	Adult	Transphenoidal pituitary surgery	The Netherlands	Qualitative	Not explicitly stated	Patients	12	Interviews (semi-structured, open, in depth)	Thematic analysis	Cooper et al. (2012)	Not reported	Five major themes emerged: (1) conflicting information and preoperative expectations, (2) IDUCs perceived as patient-friendly during bedrest, particularly for women, (3) little room for patients' opinions, (4) physical and emotional limitations and (5) fluid balance causes confusion. Information regarding IDUC placement and fluid balance given to patients both preoperatively and postoperatively did not meet their expectations, which led to confusion and uncertainty. The IDUC was perceived as preferable if bedrest was mandatory, preferred particularly by women. Patient could not mobilise freely due to the IDUC and felt ashamed, judged by others and dependent on nurses.
van de Belt, Nijmeijer, Grim, et al.	Neuro-oncology	2018	The Netherlands	Not reported	Radboudumc REshape Innovation Center,	All HIC	World Neurosurgery	***Patient-Specific Actual-Size Three-Dimensional Printed Models for Patient Education in Glioma Treatment: First Experiences***	To develop a better understanding of what patients actually value (or fear) in the application of 3D models in patient education. to identify what facilitators and barriers actually exist for the use of the models during consultation and the positive and negative effects patients perceive.	Adult	Brain tumors (Glioma)	the Netherlands	Qualitative	Framework for information and communication technologies in healthcare and Donabedian model for quality of care	Patients	11	Interviews (open, unstructured, in-depth, semi-structured, open ended)	Other: Text analysis	Not reported	Not reported	Models were successfully created for all 11 participants. There were 18 facilitators and 8 barriers identified. The model improved patients' understanding about their situation; patients reported that it was easier to ask their neurosurgeon questions based on their model and that it supported their decision about preferred treatment. A perceived barrier for using the 3D model was that it could be emotionally confronting, particularly in an early phase of the disease. Positive effects were related to psychological domains, including coping, learning effects, and communication.
Tastan, Kose, Iyigun, et al.	Neuro-oncology	2011	Turkey	RN/Nurse	School of Nursing,	All LMIC	Journal of Neuroscience Nursing	***Experiences of the relatives of patients undergoing cranial surgery for a brain tumor: A descriptive qualitative study***	We asked 10 relatives of patients who had undergone cranial surgery for a brain tumor to describe their experiences during the perioperative period and home care.	Adult	Brain tumor; Cranial surgery	Turkey	Phenomenology (Descriptive)	Not reported	Family members, carers, signifcant others	10	Interviews (open, unstructured, in-depth, semi-structured, open ended)	Phenomenological analysis	Colaizzi (1978)	Not reported	The data obtained from the patients' relatives were evaluated using Colaizzi's analysis method and divided into three categories and eight themes: (a) personal feelings (first reactions, decision for surgery, first meeting with the patient after surgery, ambiguity), (b) management of the changes (management of the side effects of the tumor, management of role and behavioral changes, management of care at home, social support), and (c) need for knowledge about managing the disease process. We found that brain tumor surgery can be more frightening for patients and their relatives than other surgical interventions. Also, because the patient requires prolonged postoperative care, the patient's family plays an important role at every stage of the patient's treatment and care.
Nicklin, E. and Phang, I. and Short, S. C. and Hoogendoorn, P. and Boele, F. W.	Neuro-oncology	2023	UK	Other: Qualitative researcher	Leeds Institute of Medical Research	All HIC	Neuro-Oncology Practice	***Patient and caregiver return to work after a primary brain tumor***	to explore, in-depth, the occupational expectations, experiences, and satisfaction of patients who RTW after a BT diagnosis and treatment, those not able to, and their family caregivers.	Adult	Primary brain tumour	UK	Qualitative	Interpretivism / constructivism	Patients AND Families/carers/ Significant other	23	Interviews (semi-structured, open, in depth)	Reflexive thematic analysis	Braun and Clarke (2019)	COREQ	Five themes were developed: (1) Early (adjustments and) expectations: "Thought I would be back at work the following Monday"; pre-treatment patients wanted to be better informed about potential recovery time and side-effects. (2) Drivers to RTW: "Getting my life back on track"; RTW was seen as a symbol of normality and also dictated by financial pressures. (3) Experiences returning to work: "It's had its ups and downs": patients who had successfully returned were supported by employers financially, emotionally, and practically. (4) Required support: "He had surgery and that was it": suggested support included a back-to-work scheme and comprehensive financial support. (5) Caring and paid work: The "juggling act": carer's work was significantly impacted; often reducing/increasing their working hours while managing increasing caring demands.
Rimmer, B. and Finch, T. and Balla, M. and Dutton, L. and Williams, S. and Lewis, J. and Gallagher, P. and Burns, R. and Araujo-Soares, V. and Menger, F. and Sharp, L.	Neuro-oncology	2024	UK	Unclear/not stated	Population Health Sciences Institute	all hic	Health Expectations	***Understanding supported self-management for people living with a lower-grade glioma: Implementation considerations through the lens of normalisation process theory***	to identify and understand what might influence the implementation of SMS for people with LGG	Adult	Glioma	UK	Qualitative	Normalisation process theory	Patients AND clinicians	53	Interviews (semi-structured, open, in depth)	inductive open coding followed by a deductive mapping approach	Not stated	Not reported	We generated supporting evidence for all four NPT constructs and related subconstructs, namely: 'Coherence', 'Cognitive participation', 'Collective action' and 'Reflexive monitoring'. Data from HCPs and people with LGG clearly demonstrated that effective SMS constitutes a collective activity. Key implementation considerations included: ensuring awareness of, and access to, support; building strong HCP-support recipient relationships; and careful inclusion of close family and friends. We identified pertinent challenges, such as identifying support needs (influenced by the extent to which those with LGG engage in help-seeking), resistance to support (e.g., technology literacy), training for HCPs and HCP cooperation.
Fletcher, Nair, MacNiven, et al.	Neuro-oncology	2012	UK	Doctoral student	Clinical Psychology / Institute of Work, Health and Organizations	All HIC	British Journal of Health Psychology	***An interpretative phenomenological analysis of the patient experience of awake craniotomy: Brain tumor diagnosis to discharge***	To explore the lived experience of having undergone an AC in the United Kingdom.	Adult	Awake craniotomy	UK	IPA	Critical realism	Patients	7	Interviews (open, unstructured, in-depth, semi-structured, open ended)	IPA	Smith (1996) and Smith, Flowers, Larkin (2009)	Not reported	Participants appeared to be satisfied with, and tolerant of, the AC experience. Three superordinate themes: use of self-preservation strategies prior to and during AC, a bizarre yet pleasant operation experience, and the need for more concrete information prior to surgery were identified. These themes appeared to be embedded in a core theme: relationship with the neurosurgeon.
Llewellyn, Neerkin, Thorne, et al.	Neuro-oncology	2018	UK	Trained ethnographer with social science background	Marie Curie Palliative Care Research Department, Division of Psychiatry	All HIC	BMJ Open	***Social and structural conditions for the avoidance of advance care planning in neuro-oncology: A qualitative study***	To elicit key social and structural conditions contributing to the avoidance of Advanced Care Planning (ACP) in neuro-oncology.	N/A	Brain tumor	UK	Qualitative	Not reported	HCPs (inc. NSx)	15	Interviews (open, unstructured, in-depth, semi-structured, open ended)	Framework analysis	Lewis & Ritchie 2003	Not reported	Participants recognized the importance of ACP but few had ever completed formal ACP documentation. We identified eight key factors, which we suggest comprise three main conditions for avoidance: (1) difficulties being a highly emotive, time-intensive practice requiring the right 'window of opportunity' and (2) presence and availability of others; (3) ambiguities in ACP definition, purpose and practice. Combined, these created a 'culture of shared avoidance'.

Nixon and Narayanasamy	Neuro-oncology	2010	UK	RN/Nurse	School of Biomedical Sciences	All HIC	Journal of clinical nursing	***The spiritual needs of neuro-oncology patients from patients' perspective***	To gain insights into the spiritual needs of neuro-oncology patients and determine their implications for practice.	Unclear	Brain tumor (glioma or anaplastic meningioma)	UK	Survey (Critical Incident Technique)	Not reported	Patients	21	Open ended questions	Other: Template analysis	Crabtree & Miller (1992)	Not reported	Nurses reported some awareness of their patients' spiritual needs during their stay on neurosurgical units although some used expressions approximating what could be described as spiritual needs. Patients' spiritual needs were identified as: need to talk about spiritual concerns, showing sensitivity to patients' emotions, responding to religious needs; and relatives' spiritual needs included: supporting them with end of life decisions, supporting them when feeling being lost and unbalanced, encouraging exploration of meaning of life, and providing space, time and privacy to talk. Participants appeared largely to be in tune with their patients' spiritual needs and reported that they recognized effective strategies to meet their patients' and relatives' spiritual needs. However, the findings also suggest that they don't always feel prepared to offer spiritual support for neuro-oncology patients.
Tresman, Brown, Fraser, et al.	Neuro-oncology	2016	UK	Not reported	Faculty of Medical Sciences,	All HIC	Pediatric Blood and Cancer	***A School Passport as Part of a Protocol to Assist Educational Reintegration After Medulloblastoma Treatment in Childhood***	To explore school-return experiences to create a more structured school reintegration protocol for children post medulloblastoma.	Pediatric	Brain tumor (Medulloblastoma)	UK	Qualitative	Not reported	Family members, carers, signifcant others AND HCPs AND non-clinical providers	27	Mixed: Qualitative questionnaires AND interview (semi-structured) AND focus groups	Thematic analysis	Braun & Clarke (2013)	Not reported	This study uncovered the following four main subjects: (1) Information sharing; (2) education and empowerment (of educational professionals (EP) and parents); (3) communication between parents, HCPs and EPs; and (4) long-term difficulties. Implementation of a standardized protocol delivered within the structure of a school passport document would aid uniform follow-up. The proposed multistage protocol includes early communication and reintegration planning followed by meetings at school re-entry. Follow-up meetings are suggested to reduce information loss and reassess the child's needs. Hospital support at school transitions, inclusion of school data in long-term clinical follow-up and long-term rehabilitation are also recommended. Each stage would be supported by school passport documentation and would facilitate school and parental empowerment, paramount to the long-term sustainability of successful schooling.
Palmisciano, Jamjoom, Taylor, et al.	Neuro-oncology	2020	UK / Italy	MD/Physician	Department of Neurosurgery; Department of Surgery	All HIC	World Neurosurgery	***Attitudes of Patients and Their Relatives Toward Artificial Intelligence in Neurosurgery***	To evaluate attitudes of patients and their relatives regarding use of AI in neurosurgery.	Adult	Brain tumor	UK	Mixed methods survey	Not reported	Patients	20	Open ended questions	Other: analyzed qualitatively looking for major themes	Not reported	Not reported	Five themes were identified: interpretation of imaging (4/20; 20%), operative planning (5/20; 25%), real-time alert of potential complications (10/20; 50%), partially autonomous surgery (6/20; 30%), and fully autonomous surgery (3/20; 15%).
Ward-Smith	Neuro-oncology	1997	USA	RN/Nurse	Brain Tumor Institute,	All HIC	The Journal of neuroscience nursing : journal of the American Association of Neuroscience Nurses	***Stereotactic radiosurgery for malignant brain tumors: the patient's perspective***	To describe the experience and meaning of the treatment of stereotactic radiosurgery from the patient's perspective.	Adult	Brain tumors (malignant); Stereotactic radiosurgery	US	Phenomenology	Phenomenological	Patients	8	Interviews (open, unstructured, in-depth, semi-structured, open ended)	Phenomenological analysis	Colaizzi (1978)	Not reported	Using Colaizzi's phenomenological technique, five potential themes emerged from the interviews obtained. They are; (1) A willingness of patients to undergo treatment again, (2) self-directed education regarding the treatment method, (3) an ability to verbalize the prognosis for their tumor type, (4) the lack of discomfort during treatment and (5) the fact that this treatment was not originally a treatment option.
Lepola, Toljamo, Aho, et al.	Neuro-oncology	2001	Finland	RN/Nurse	University of Oulu,	All HIC	The Journal of neuroscience nursing : journal of the American Association of Neuroscience Nurses	***Being a brain tumor patient: a descriptive study of patients' experiences***	To describe the experience of being a patient with a brain tumor	Adult	Brain tumor	USA	Qualitative	Not reported	Patients	8	Interviews (open, unstructured, in-depth, semi-structured, open ended)	Content analysis	Polit & Hungler (1995)	Not reported	Preoperatively, some patients had a fearless and calm attitude towards their illness, while others were fearful and depressed. Postoperatively, the patients' body images changed, and they were concerned about their future. They perceived their care as matter-of-fact and friendly both before and after the surgery, and they thought their basic needs were met. Some patients would have wanted more psychological support, especially after the surgery, while others found the psychological support adequate. The patients were willing to participate in decision making about their care, and they trusted the professional skill and competence of the nurses. Suggested improvements in care were to minimize the atmosphere of urgency and hurry, appoint a primary nurse for each patient, and give more attention to after-care.
Chang, J. J. and Amano, A. and Brown-Johnson, C. and Chu, O. and Gates-Bazarbay, V. and Wipff, E. and Kling, S. M. R. and Alhadha, M. and Fernandez-Miranda, J. C. and Vilendrer, S.	Neuro-oncology	2024	USA	Physician/MD	Division of Endocrinology	All HIC	Journal of Clinical and Translational Endocrinology	***Patient and caregiver perspectives of fluid discharge protocols following pituitary surgery***	to understand the factors necessary for successful implementation of fluid restriction and discharge care protocols following transsphenoidal surgery	Adult	pituitary and sellar lesions; transsphenoidal surgery	USA	Qualitative	Non stated	Patients AND Families/carers/ Significant other	19	Interviews (semi-structured, open, in depth)	Other: Matrix analysis (Inductive)	Miles, Huberman and Saldana (2020); Averill (2002)	Not reported	Most patients and caregivers perceived fluid restriction protocols as acceptable and feasible when indicated. Facilitators to the protocols included clear communication about the purpose of and strategies for fluid restriction, access to the care team, and involvement of patients' caregivers in care discussions. Barriers included patient confusion about differences in the care plan between teams, physical discomfort of fluid restriction, increased burden of tracking fluids during recovery, and lack of clarity surrounding desmopressin prescriptions
Kenton, N. R. and Estafanous, M. and Itamura, K. and Filus, A. and Gowrinathan, S. and Martin, N. A. and Sivakumar, W. and Barkhoudarian, G. and Byrne, P. J. and Kochhar, A.	Neuro-oncology	2023	USA	Other: MPH	Center for Outcomes Research and Education	All HIC	Jama Otolaryngology-Head & Neck Surgery	***Patient Perception of Education, Care Coordination, and Psychological Distress After Developing Facial Paralysis A Qualitative Study***	To (1) identify patient preparedness for developing facial paralysis and how well their care is coordinated following its development and (2) present in their own words outcomes of facial paralysis in terms of physical health, emotional health, self-perception, and social interactions.	Adult	Vestibular schwannoma	USA	Qualitative	Non stated	Patients	12	Interviews (semi-structured, open, in depth)	Thematic analysis	Braun and Clarke (2006)	SRQR	Four major themes were identified: (1) lack of sufficient patient education about the diagnosis of facial paralysis; (2) lack of appropriate care coordination related to facial paralysis; (3) changes in physical and emotional health following facial paralysis; and (4) changes in social interactions and external support following facial paralysis.

Cutillo, Zimmerman, Davies, et al.	Neuro-oncology	2020	USA	Doctoral student (Psychology)	Department of Psychology	All HIC	Journal of Neurosurgery: Pediatrics	***Caregiver-provider communication after resection of pediatric brain tumors***	This study aims to reveal communication styles of neurosurgery team members with caregivers of children with a newly diagnosed brain tumor requiring neurosurgery, and to identify areas for improvement in the future.	Pediatric	brain tumor	USA	Qualitative	Not reported	Family members, carers, signifcant others	22	Interviews (open, unstructured, in-depth, semi-structured, open ended)	Phenomenological analysis	Not reported	Not reported	During caregiver interviews (N = 22), several domains were discussed including communicating the diagnosis to the patient and siblings, to the rest of the family/support network, and with the neurosurgery team. Regarding parent-neurosurgeon communication, 82% of caregivers identified at least one positive aspect and 55% identified at least one negative aspect of communication. Caregivers who provided positive feedback appreciated that their neurosurgeon was thorough (73%), direct (27%), or compassionate (14%). They also valued when providers would speak "on my level" (18%) and would speak directly to the patient (27%). In terms of negative feedback, caregivers identified miscommunications (32%), discussing the diagnosis in front of the child before feeling prepared to do so (14%), and a lack of clarity about expectations, medications, or treatment (32%).
Cutillo, Zimmerman, Davies, et al.	Neuro-oncology	2019	USA	Doctoral student (Psychology)	Department of Psychology	All HIC	Journal of Neurosurgery: Pediatrics	***Coping strategies used by caregivers of children with newly diagnosed brain tumors***	The goal of this study was to determine what strategies caregivers use to cope with the stress of a child who has recently undergone surgical treatment for a newly diagnosed brain tumor. Results will be used to improve psychosocial assessments and treatments provided to these families during initial hospitalization.	Pediatric	brain tumor	USA	Mixed methods (concurrent triangulation design)	Not reported	Family members, carers, signifcant others	22	Mixed: Interviews (open, unstructured, in-depth, semi-structured, open ended) AND questionnaires	Phenomenological analysis	Not reported	Not reported	Caregivers identified several adaptive coping strategies, such as active coping, acceptance coping, emotion-focused coping, spiritual coping, social support, and posttraumatic growth. Maladaptive coping strategies were mostly categorized as avoidant coping. Caregivers endorsed multiple different coping strategies (mean of 4.59 strategies per caregiver). No clinical or demographic variables, whether the tumor was benign or malignant, or the DT score correlated with caregiver endorsement of maladaptive coping strategies.
Foust Winton, Draucker and Von Ah	Neuro-oncology	2021	USA	RN/Nurse	Department of Community and Health Systems, Indiana University School of Nursing	All HIC	Cancer nursing	***Pain Management Experiences Among Hospitalized Post craniotomy Brain Tumor Patients***	To describe how patients who have undergone a craniotomy for brain tumor removal experience pain management while hospitalized.	Adult	Brain tumor; Craniotomy	USA	Qualitative (descriptive)	Not reported	Patients	27	Interviews (open, unstructured, in-depth, semi-structured, open ended)	Content analysis	Miles, Huberman & Saldana (1994)	COREQ	Their pain experiences varied on two dimensions: salience of pain during recovery and complexity of pain management. Based on these dimensions, three distinct types of pain management experiences were identified: 1) pain-as-non-salient, routine pain management experience; 2) pain-as-salient, routine pain management experience; and 3) pain-as-salient, complex pain management experience. Many post-craniotomy patients experience their pain as tolerable and/or pain management as satisfying and effective, others experience pain and pain management as challenging.
Foust Winton, Draucker and Von Ah	Neuro-oncology	2021	USA	RN/Nurse	Department of Community and Health Systems, Indiana University School of Nursing	All HIC	Clinical nurse specialist CNS	***Pain Quality Among Hospitalized Postcraniotomy Brain Tumor Patients***	To describe how persons diagnosed with a brain tumor who have had a craniotomy describe the quality of their pain after surgery.	Adult	Brain tumor; Craniotomy	USA	Qualitative (descriptive)	Not reported	Patients	27	Interviews (open, unstructured, in-depth, semi-structured, open ended)	Content analysis	Miles, Huberman & Saldana (1994)	Not reported	Participants described the quality of their pain with six different types of descriptors: pain as pressure, pain as tender or sore, pain as stabbing, pain as throbbing, pain as jarring, and pain as itching. Participants' descriptions of their pain quality after surgery provide a different understanding than do numerical pain ratings. Clinicians should use questions to explore patients' individual pain experiences, seeking to understand the quality of patients' pain and their perceptions.
Hocking, Quast, Brodsky, et al.	Neuro-oncology	2017	USA	Not reported	School of Medicine	All HIC	Supportive Care in Cancer	***Caregiver perspectives on the social competence of pediatric brain tumor survivors***	To obtain caregiver perspectives on survivor social competence and identify pertinent risk and resistance factors.	Pediatric	Brain tumor	USA	Mixed methods	Social competence model	Family members, carers, signifcant others	45	Mixed: Focus groups AND Surveys AND confirmatory individual interviews	Content analysis	Hsieh & Shannon (2005)	Not reported	Qualitative content Analyzes resulted in three themes that were illustrative of the model of social competence. Themes included (1) the impact of survivor sequelae on social function; (2) the role of family in evaluating and promoting survivor social development; and (3) the match between the survivor's social context and developmental needs. Quantitative data supported the associations between survivor social skills, survivor executive function, and family functioning.
Hricik, Donovan, Bradley, et al.	Neuro-oncology	2011	USA	Research associate	School of Nursing	All HIC	Oncology nursing forum	***Changes in caregiver perceptions over time in response to providing care for a loved one with a primary malignant brain tumor***	To examine how family members of patients with a primary malignant brain tumor transition into the caregiver role and how their perceptions of this transition change over time.	Adult	Primary Brain tumor	USA	Qualitative (descriptive)	Pittsburgh Mind-Body Center's common pathways model adapted by Sherwood et al. (2008)	Family members, carers, signifcant others	10	Interviews (open, unstructured, in-depth, semi-structured, open ended)	Content analysis	Patton (2002)	Not reported	Caregivers described difficulties stemming from the patient's tumor-related dysfunction and changes in their familial, occupational, and social roles. Support from family and friends was vital to caregivers' emotional health, but shock and fear were evident in all interviews. Becoming subsumed in the care situation was described as enmeshment. Caregivers reported difficulty in communicating with healthcare providers. When looking at change over time, three major themes emerged: Patient Changes: The New Normal; Caregiver Adjustments; and Accessing Support.
Schubart, Kinzie and Farace	Neuro-oncology	2008	USA	Not reported	Clinical Informatics Program, Department of Public Health Sciences	All HIC	Neuro-Oncology	***Caring for the brain tumor patient: Family caregiver burden and unmet needs***	We studied the specific challenges that family caregivers face when caring for patients experiencing the significant neurocognitive and neurobehavioral disorders associated with brain tumors	Adult	Brain tumor	USA	Grounded theory	Not reported	Family members, carers, signifcant others	25	Interviews (open, unstructured, in-depth, semi-structured, open ended)	Grounded theory analysis/constant comparison/open and axial/selective coding AND cross-case analysis	Glasser & Strauss (1967)	Not reported	We found that the family caregivers in this study provided extraordinary uncompensated care involving significant amounts of time and energy for months or years and requiring the performance of tasks that were often physically, emotionally, socially, or financially demanding. They were constantly challenged to solve problems and make decisions as care needs changed, yet they felt untrained and unprepared as they struggled to adjust to new roles and responsibilities. Because the focus was on the patient, their own needs were neglected. Because caregiver information needs are emergent, they are not always known at the time of a clinic visit. Physicians are frequently unable to address caregiver questions, a situation compounded by time constraints and cultural barriers.
Green, Demchuk and Newcommon	Neurovascular	2015	Not reported	RN/Nurse	not stated	N/A	Canadian journal of neuroscience nursing	***Aggressive surgical interventions for severe stroke: Impact on quality of life, caregiver burden and family outcomes***	To gain a better understanding of personal and social consequences of surviving aggressive surgical intervention for malignant MCA infarction, lobar, hemorrhage, and poor-grade subarachnoid hemorrhage.	Adult	Malignant MCA infarction, lobar, hemorrhage, and poor-grade subarachnoid hemorrhage.	Canada	Mixed methods (Convergent parallel design)'	Not reported	Patients AND Family members, carers, signifcant others	40	Mixed: Interviews (open, unstructured, in-depth, semi-structured, open ended) AND questionnaires	Content analysis	Hsieh & Shannon (2005)	Not reported	Qualitatively, the overarching theme in the interviews centered on survival, change, and loss. At the time of the stroke event, caregivers/family focused entirely on the patient living or dying. As time progressed, other issues became prominent. Participants spoke of change with respect to personal and interpersonal relationships, internal family function, external social and community reintegration, and loss with respect to one's old self, confidence and independence.

Ganesh, A. and Beland, B. and Jewett, G. A. E. and Campbell, D. J. T. and Varma, M. and Singh, R. J. and Al-Sultan, A. and Wong, J. H. and Menon, B. K.	Neurovascular	2024	Canada	Physician/MD	Calgary Stroke Program, Department of Clinical Neurosciences, University of Calgary Cumming School of Medicine	All HIC	Canadian Journal of Neurological Sciences	***Physician Approaches to Antithrombotic Therapies for Recently Symptomatic Carotid Stenosis***	to explore the approaches of stroke physicians to antithrombotic management of patients with symptomatic carotid stenosis	N/A	Carotid stenosis	Canada, the United States, United Kingdom, Australia, Spain, Germany, Zimbabwe, Jamaica, the Czech Republic, and India	Qualitative descriptive	Non stated	HCPs (Inc NSx)		22	Interviews (semi-structured, open, in depth)	Content analysis	Chun Tie, Birks, Francis (2019)	COREQ	Important themes revealed from our analysis included limitations of existing clinical trial evidence, competing surgeon versus neurologist/internist preferences, and the choice of antiplatelet therapy while awaiting revascularization. There was a greater concern for adverse events while using multiple antiplatelet agents (e.g., dual-antiplatelet therapy (DAPT)) in patients undergoing carotid endarterectomy compared to carotid artery stenting. Regional variations included more frequent use of single antiplatelet agents among European participants. Areas of uncertainty included antithrombotic management if already on an antiplatelet agent, implications of nonstenotic features of carotid disease, the role of newer antiplatelet agents or anticoagulants, platelet aggregation testing, and timing of DAPT.
Masuda, Oishi and Yamamoto	Neurovascular	2014	Japan	RN/Nurse	School of Nursing	All HIC	Nursing Research	***Uncertainty in patients with unruptured intracranial aneurysms undergoing endovascular surgery: A qualitative and inductive study***	To clarify the nature of the uncertainty experienced by unruptured intracranial aneurysms (UIA) patients who elect to undergo endovascular surgery.	Adult	unruptured intracranial aneurysms	Japan	Qualitative	Not reported	Patients		31	Interviews (open, unstructured, in-depth, semi-structured, open ended)	Content analysis	Krippendorff (1980)	Not reported	Six categories were derived from the analysis to describe the uncertainty experienced by UIA patients who undergo intravascular surgery: Nature of the Disease, Treatment Characteristics, Information, Decision-making, Course of the Future, and Living with UIA.
Stone	Neurovascular	2007	Canada	Not reported	Department of Sociology	N/A	Journal of clinical nursing	***Patient concerns posthaemorrhagic stroke: a study of the Internet narratives of patients with ruptured arteriovenous malformation***	To identify and describe the experiences and concerns of a sample of young haemorrhagic stroke patients who experienced a ruptured arteriovenous malformation and determine whether there are gender differences.	Adult	Hemorrhagic stroke; Arterio-venous malformation	N/A - internet search	Qualitative	Not reported	Patients	Other: 83		Narratives published on an Internet site	Content analysis	Manning & Cullum-Swan (1994)	Not reported	Five main categories of topics were identified: 'symptoms', 'doctors and hospitals', 'rehabilitation and recovery', 'disabilities' and 'miscellaneous reflections'. The latter category contained the sub-categories 'arteriovenous malformation website', 'thanks', 'life now' and 'feelings'. Narratives varied greatly regarding how much each topic was addressed, but there were few gender differences. Overall, most attention was paid to discussing doctors and hospitals and most writers also paid significant attention to the importance of being able to share experiences with other survivors.
Dammann, Abla, Al-Shahi Salman, et al.	Neurovascular	2021	Germany	MD/Physician	Department of Neurosurgery and Spine Surgery	All HIC	Journal of neurosurgery	***Surgical treatment of brainstem cavernous malformations: an international Delphi consensus***	To seek consensus about surgical management aspects of brainstem cavernous malformations	Adult	brainstem cavernous malformations	Neurosurgeons from several countries	Delphi/Consensus	Not reported	NSx		22	Open ended questions	Content analysis	Downe-Wamboldt (1992)	Not reported	Twenty-two (76%) of 29 experts participated in the consensus. Qualitative analysis (content analysis) of an initial open-ended question survey resulted in 99 statements regarding surgical treatment of BSCM. By using a multistep survey with 100% participation in each round, consensus was reached on 52 (53%) of 99 statements. These were grouped into 4 categories: 1) definitions and reporting standards (7/14, 50%); 2) general and patient-related aspects (11/16, 69%); 3) anatomical-, timing of surgery-, and BSCM-related aspects (22/37, 59%); and 4) clinical situation-based decision-making (12/32, 38%). Among other things, a consensus was reached for surgical timing, handling of associated developmental venous anomalies, handling of postoperative BSCM remnants, assessment of specific anatomical BSCM localizations, and treatment decisions in typical clinical BSCM scenarios.
Ryu, Yang, Choi et al.	Neurovascular	2020	Republic of Korea	Not reported	College of Nursing	All HIC	Japan journal of nursing science : JJNS	***Lived experiences of adult patients with moyamoya disease: A qualitative case study***	To understand the daily lived experiences of adult moyamoya disease patients.	Adult	Moyamoya disease - cerebrovascular occlusive disease	South Korea	Case study	Not reported	Patients		14	Interviews (open, unstructured, in-depth, semi-structured, open ended)	Phenomenological descriptive analysis	Colaizzi's 7 step process (no reference)	Not reported	Participants' experiences were divided into three themes and eight sub-themes. "Having an unexpected disease that suddenly struck my life" refers to confusion and depression due to the diagnosis of the unexpected illness; "being occasionally anxious about the illness" describes patients' uncertainty about the disease and worrying about passing the disease on to their child; and "living with the disease by going through the disease experience" refers to the process of accepting and adapting to the illness.
von Vogelsang, Ann-Christin and Nymark, Carolin and Pettersson, Susanne and Jervaeus, Anna	Neurovascular	2023	Sweden	Unclear/not stated	Department of Neurosurgery & Department of Clinical Neuroscience	All HIC	Disability and rehabilitation	***"My head feels like it has gone through a mixer" - a qualitative interview study on recovery 1Â year after aneurysmal subarachnoid hemorrhage***	To describe patients' perceived and expected recovery 1 year after aneurysmal subarachnoid hemorrhage (aSAH).	Adult	aneurysmal subarachnoid haemorrhage	Sweden	Qualitative	Naturalism	Patients		16	Interviews (semi-structured, open, in depth)	Content analysis	Graneheim and Lundman (2004, 2017)	COREQ	The analysis resulted in two categories and seven subcategories. The category "A spectrum of varying experiences of recovery" includes four subcategories describing physical recovery, mental recovery, alterations in social life, and perceived possibilities to return to normality. Some informants felt that life was almost as before, while others described a completely different life, including a new view of self, altered relationships, not being able to return to work, and effects on personal finances. The category "A spectrum of reflections and expectations of recovery" comprises three subcategories depicturing that expectations of recovery were influenced by existential thoughts, describing what they based own expectations of recovery on, and how expectations from others influenced them.
Ericsson, Hult and Kumlien	Neurovascular	2018	Sweden	RN/Nurse	Department of Health Science/ Department of Care Science	All HIC	Journal of Perianesthesia Nursing	***Patients' Experiences During Carotid Endarterectomy Performed Under Local Anesthesia***	To describe patients' experiences undergoing a carotid endarterectomy (CEA) under local anesthesia.	Adult	Carotid endarterectomy (CEA)	Sweden	Qualitative	Not reported	Patients		15	Interviews (open, unstructured, in-depth, semi-structured, open ended)	Content analysis (manifest and latent)	Graneheim & Lundman (2004)	Not reported	Undergoing CEA under local anesthesia entails enduring stress with no possibility of withdrawal. Patients' lack of understanding of local anesthesia and experiencing pain and discomfort caused feelings of stress. The surgery resulted in a loss of control; patients had to surrender their autonomy to someone else. The nurse anesthetist was the link to the world outside the operating room (OR), and that nurse conveyed feelings of safety and security during the surgery.

Hedlund, Zetterling, Ronne-Engstrom, et al.	Neurovascular	2010	Sweden	RN/Nurse	Department of Public Health and Caring Sciences and Department of Neuroscience Psychiatry	All HIC	Journal of clinical nursing	***Perceived recovery after aneurysmal subarachnoid hemorrhage in individuals with or without depression***	To describe what patients with no or only minor neurological deficits after aneurysmal subarachnoid hemorrhage (SAH) perceived to be important for recovery, and perceived consequences of the illness.	Adult	Subarachnoid hemorrhage; Depression	Sweden	Qualitative (descriptive)	Not reported	Patients	20	Interviews (open, unstructured, in-depth, semi-structured, open ended)	Content analysis	Graneheim & Lundman (2004)	Not reported	Two patterns were identified. One pattern revealed that informants without depression experienced a 'confident perception of recovery', which included perceptions of meaningfulness. Another pattern revealed that depressed informants experienced a 'pessimistic perception of recovery', which included perceptions of hopelessness. Expectations regarding care after departure from the neurointensive care unit were not met.
Johannessen	Neurovascular	2014	Sweden	Not reported	Department of Sociology and Human Geography	N/A	Social Science and Medicine	***The narrative (re)production of prestige: How neurosurgeons teach medical students to valorize diseases***	Based on a fieldwork study of the teaching of neurosurgery at a Norwegian university hospital, this paper shows how notions of disease prestige are (re)produced through neurosurgeons' telling of disease narratives in medical education.	N/A	Subarachnoid hemorrhage	Sweden	Ethnography (Narrative approach)	Interpretivism/ disease prestige	HCPs (inc. NSx)	74 observed, 1 interviewed 13 times	Mixed: Observations AND Key informant interview	Grounded theory analysis/constant comparison/open and axial/selective coding	Strauss and Corbin, (1998)	Not reported	The analysis presents their prestigious narrative of subarachnoid hemorrhage (SAH), a rare form of stroke, which neurosurgeons presented as an acute and potentially lethal but curable disease. In contrast to perceivably more ordinary diseases, their portrayal of SAH references heroic narratives on a more abstract cultural level, casting neurosurgeons as masculine and extraordinary lifesavers, able to act where others fall short.
Persson, Törnbom, Sunnerhagen, et al.	Neurovascular	2017	Sweden	Physiotherapist	Department of Clinical Neuroscience, Institute of Neuroscience and Physiology	All HIC	PLoS ONE	***Consequences and coping strategies six years after a subarachnoid hemorrhage - A qualitative study***	To explore experiences of the care and rehabilitation as well as the consequences and strategies used to cope with everyday life six years post SAH.	Adult	Subarachnoid hemorrhage	Sweden	Qualitative (descriptive)	Not reported	Patients	16	Interviews (open, unstructured, in-depth, semi-structured, open ended)	Thematic analysis	Braun & Clarke (2006)	COREQ	Two major themes from the analysis, both including four sub-themes, were identified; these themes were consequences of the SAH and coping strategies. Participants were grateful to have survived the SAH and most were satisfied with their acute medical care. If discharged directly from the neurosurgical unit participants can feel abandoned. In contrast, participants who were referred to a rehabilitation clinic felt supported and informed. Cognitive problems, such as impaired memory and mental fatigue, were reported as still present six years post SAH. Coping strategies were; receiving support from family, society, employers, or technical equipment. At work, talking to colleagues and to taking breaks were common. Participants described hiding their symptoms from employers and friends, as well as trying to continue doing tasks in the same manner as prior to the SAH. If this was not possible, some refrained from doing these tasks. They went through a mourning process, fear, and worries.
Von Vogelsang, Wengström, Svensson, et al.	Neurovascular	2014	Sweden	RN/Nurse	Department of Neurobiology, Care Sciences and Society	All HIC	Journal of Clinical Nursing	***Transitional experiences in patients following intracranial aneurysm rupture***	To describe changes and transitions in everyday life in the first two years following an intracranial aneurysm rupture.	Adult	Subarachnoid hemorrhage	Sweden	Mixed methods (Exploratory, equal priority parallel analysis)	Not reported	Patients	88	Questionnaire: Open and close-ended questions	Content analysis	Graneheim & Lundman (2004)	Not reported	A majority of participants perceived changes in their everyday lives during the first two years following aneurysm rupture, and the changes were ongoing with little differences reported between 6 months and 2 years after the onset. Internal changes, or transitions, were revealed within changes in personality, changed social roles and relationships and changed abilities and behavior.
Göcking, B. and Biller-Andorno, N. and Brandi, G. and Gloeckler, S. and Glässel, A.	Neurovascular	2023	Switzerland	Nurse/RN/RGN	Institute of Biomedical Ethics and History of Medicine	All HIC	International Journal of Environmental Research and Public Health	***Aneurysmal Subarachnoid Hemorrhage and Clinical Decision-Making: A Qualitative Pilot Study Exploring Perspectives of Those Directly Affected, Their Next of Kin, and Treating Clinicians***	To explore the experience and impact of aSAH in Switzerland on the informed medical decision-making process from three perspectives: affected persons, next of kin, and treating clinicians.	Adult	aSAH	Switzerland	Qualitative	Non stated	Patients AND Families/carers/ Significant other AND clinicians	14	Interviews (semi-structured, open, in depth)	Thematic analysis	Braun and Clarke (2006); Braun and Clarke (2013)	Not reported	Qualitative analysis revealed five main themes from the perspective of clinicians: emergency care, diagnosis and treatment, outcomes, everyday life in the ICU, and decision-making; seven main themes were identified for AFs and NoK: the experience of the aSAH, diagnosis and treatment, outcomes, impact on loved ones, identity, faith, religion and spirituality, and decision-making. Perspectives on decision-making were compared, and, whereas clinicians tended to focus their attention on determining treatment, AFs and NoK valued participation in shared decision-making processes
Nobels-Janssen, E. and Abma, I. L. and de Ridder, I. R. and Haeren, R. H. L. and Hertog, M. H. and Nanda, D. and van der Pol, B. and Verhagen, W. I. M. and Bartels, R. H. M. A. and van der Wees, P. J. and Boogaarts, H. D.	Neurovascular	2023	The Netherlands	Unclear/not stated	Department of Neurology; Department of Neurosurgery	All HIC	BMC neurology	***The SOS-SAH questionnaire in clinical practice: a multi-method evaluation study***	to evaluate the perceived impact of using the SOS-SAH in daily clinical practice for patients after aSAH, as well as to explore potential barriers to further implementation.	Adult	aSAH	The Netherlands	Mixed methods,	Not explicitly stated	HCPs (Inc NSx)	22	Interviews (semi-structured, open, in depth)	Thematic analysis	Not stated	Not reported	The survey did not reveal any differences between the usual-care group and the post-implementation group on the scales of the patient experience survey. After implementation of the SOS-SAH, the number of symptoms discussed during consultation did not increase. The interviews suggest that the SOS-SAH may improve the preparation of patients by providing them with greater insight into their complaints and by raising issues for the consultation. It could also enhance the structure and efficiency of consultation, in addition to improving communication about issues that matter to patients. All patients and healthcare professionals recommended continuing the use of the SOS-SAH in daily practice.
Urlings, J. and Abma, I. and Aquarius, R. and Aalbers, M. and Bartels, R. and Maal, T. and Henssen, D. and Boogaarts, J.	Neurovascular	2023	The Netherlands	Researcher	Department of Neurosurgery	All HIC	Frontiers in Bioengineering and Biotechnology	***Augmented reality-The way forward in patient education for intracranial aneurysms? A qualitative exploration of views, expectations and preferences of patients suffering from an unruptured intracranial aneurysm regarding augmented reality in patient education***	to explore the views, expectations and preferences of patients with an unruptured intracranial aneurysm regarding the use of AR in patient education.	Adult	Unruptured intracranial aneurysm	the Netherlands	Qualitative	Not explicitly stated	Patients	17	Interviews (semi-structured, open, in depth)	Thematic content analysis	non stated	not reported	Seventeen interviews were conducted. The views, expectations and preferences of patients regarding patient education with AR could be subdivided into 15 categories, which could be grouped into 4 general themes: 1) experiences with current patient education, 2) expectations of AR in patient education, 3) opportunities and limitations of AR, and 4) out-of-hospital use of an AR application. Patients' expectations were predominantly positive regarding improving patients' understanding of their medical situation and doctor-patient communication.
Jelen, M. B. and Clarke, R. E. and Jones, B. and Toma, A. K. and Pandit, A. S.	Neurovascular	2023	UK	Unclear/not stated	Department of Psychology and Language Sciences University College London London UK	All HIC	Stroke-Vascular and Interventional Neurology	***Psychological and Functional Impact of a Small Unruptured Intracranial Aneurysm Diagnosis: A Mixed-Methods Evaluation of the Patient Journey***	we aim to characterize the early small unruptured intracranial aneurysm (sUIA) patient journey and evaluate their psychosocial status.	Adult	unruptured aneurysm	UK	Mixed methods,	Non stated	Patients	33	Interviews (semi-structured, open, in depth)	Thematic analysis	Braun and Clark (2006)	GRAMMS	Thematic analysis of interview responses (n=33) identified 5 recurrent themes underpinning the sUIA patient experience: referral, diagnosis and information sharing, imaging surveillance, psychological impairment and coping strategies, and activities of daily living, all of which contributed to patient concerns.

Wade, J. and Farrar, N. and Realpe, A. X. and Donovan, J. L. and Forsyth, L. and Harkness, K. A. and Hutchinson, P. J. A. and Kitchen, N. and Lewis, S. C. and Loan, J. J. M. and Stephen, J. and Salman, R. A. and collaboration, Care pilot trial	Neurovascular	2024	UK	Unclear/not stated	Bristol Medical School	All HIC	Eclinicalmedicine	***Addressing barriers and identifying facilitators to support informed consent and recruitment in the Cavernous malformations A Randomised Effectiveness (CARE) pilot phase trial: insights from the integrated QuinteT recruitment intervention (QRI)***	to identify barriers and facilitators to recruitment and how and why these arose. Working with the chief investigators and trial management group, we addressed barriers and facilitators with corresponding actions to improve informed consent and recruitment.	Mixed	Cavernous malformations	UK	Qualitative	Not explicitly stated	Mixed (patients, investigators, HCPs)	144	Mixed/multiple: Interviews, workshops	Thematic, content or conversation analysis	Braun and Clarke (2006), Krippendorf (1980), Wade (2009)	COREQ	Barriers identified included how usual care practices made equipoise challenging, multi-disciplinary teams sometimes overrode recruiter equipoise and logistical issues rendered symptomatic cavernoma diagnosis and assessment for stereotactic radiosurgery challenging. Facilitators identified included the preparedness of some neurosurgeons' to offer surgery to people otherwise offered medical management alone, multi-disciplinary team equipoise, and effective information provision presenting participation as a solution to equipoise regarding management. Actions, before and during recruitment, to improve inclusivity of site screening, approach and effectiveness of information provision resulted in 72 participants recruited following a 5-month extension, exceeding the target of 60 participants.
Jarvis	Neurovascular	2002	UK	RN/Nurse	Dept of Neurosurgery	N/A	British journal of nursing	***Recovering from subarachnoid hemorrhage: patients' perspective***	To illuminate the experience of recovery from SAH	Adult	Subarachnoid hemorrhage	UK	Qualitative	Not reported	Patients	8	Interviews (open, in-depth, semi-structured, open ended)	Thematic analysis: Deductive, inductive, then validation	Ely et al (1997)	Not reported	As a result, the study offers an insight into how common psychological difficulties may impact on a person's ability to make a full and complete recovery following SAM In conclusion, the main themes generated by the data are presented and recommendations for practice in meeting the needs of participants are offered.
Mc Lernon, Werring, and Terry	Neurovascular	2021	UK	Not reported	School of Health and Social Care	All HIC	Neurocritical Care	***Clinicians' Perceptions of the Appropriateness of Neurocritical Care for Patients with Spontaneous Intracerebral Hemorrhage (ICH): A Qualitative Study***	To qualitatively explore perceptions of neurocritical care in relation to the expected functional outcome for ICH patients.	Adult	Intracranial hemorrhage	UK	Qualitative	Not reported	HCPs (inc. NSx)	21	Interviews (open, unstructured, in-depth, semi-structured, open ended)	Reflexive thematic analysis	Braun & Clarke (2006, 2013)	SRQR	We conducted 21 semi-structured interviews and identified five key themes: (1) prognostic uncertainty (2) subjectivity of good versus poor outcome (3) perceived inappropriate care (PIC) situations (including for frail elderly patients) (4) challenging nature of decision-making (5) clinician distress.
McKenna, Wilson, Caldwell, et al.	Neurovascular	2013	UK	Psychologist (incl. clinical, neuro-)	Queen's University Belfast	All HIC	Disability and rehabilitation	***Decompressive hemicraniectomy following malignant middle cerebral artery infarctions: a mixed methods exploration of carer experience and level of burden***	To explore the experiences and sense of burden of family carers of survivors of malignant middle cerebral artery infarctions who had undergone decompressive hemicraniectomy.	Adult	Decompressive hemicraniectom y	UK	Mixed methods (Convergent parallel design)	Not reported	Family members, carers, signifcant others	6	Mixed: Interviews (open, unstructured, in-depth, semi-structured, open ended) AND questionnaires	Thematic: Content analysis	Braun & Clarke (2006)	Not reported	While carers experienced many losses, their overall sense of burden was not outside 'Average' limits, nor did they experience clinically significant symptoms of depression. All carers identified methods of coping with the demands of caregiving. These included intrapersonal, interpersonal and practical strategies. All carers apart from one were able to identify areas of post-traumatic growth.
Heidenreich, S. and Trapali, M. and Krucien, N. and Phillips-Beyer, A.	Neurovascular	2023	UK	Unclear/not stated	Patient-Centered Research	All HIC	Frontiers in Neurology	***Clinicians' preferences for managing aneurysmal subarachnoid hemorrhage using endothelin receptor antagonists***	This study explored the preferences of clinicians involved in the management of aSAH post aneurysm repair, the benefit-risk of ERAs from the clinicians' perspective, and the trade-offs clinicians are willing to make in risks of adverse events to obtain a reduction in the risk of DCI	N/A	SAH	UK, USA	Mixed methods,	Non stated	HCPs (Inc NSx)	10	Mixed/multiple: Interviews AND online survey	Other: content and thematic analysis	non stated	Not reported	Clinicians were willing to accept certain increased risks of adverse events for a reduced risk of DCI after aSAH.
Alotaibi, Samuel, Wang, et al.	Neurovascular	2017	Canada	Not reported	Division of Neurosurgery, Institute of Medical Science, Faculty of Medicine	All HIC	World neurosurgery	***The Use of Social Media Communications in Brain Aneurysms and Subarachnoid Hemorrhage: A Mixed-Method Analysis***	To evaluate the use of social media and the online communications regarding brain aneurysms.	Unclear	Subarachnoid hemorrhage	Unclear	Mixed methods	Not reported	Other	Other: 50 posts and comments from each title category	Social media content: Facebook, Twitter, YouTube	Grounded theory analysis/constant comparison/open, axial and/or selective coding (modified thematic analysis)	Corbin & Strauss (2008)	Not reported	Six prominent themes emerged from the coded data of posts and comments: inspiration and motivation (27.7%), providing and sharing information (26.3%), requesting information (14.4%), seeking emotional support (12.1%), admiration (8.3%), and loss and grief (8.3%).
Feler, Tan, Sammann, et al.	Neurovascular	2019	USA	Not reported	Yale University School of Medicine	All HIC	World Neurosurgery	***Decision Making Among Patients with Unruptured Aneurysms: A Qualitative Analysis of Online Patient Forum Discussions***	To understand the perspectives and experiences in medical decision making for patients selecting management for UIAs.	Unclear	Unruptured aneurysms	Unclear - assumed international	Internet-based ethnography	Not reported	Patients	Other: 110	Online patient forum (user accounts)	Thematic analysis AND constant comparison	Braun & Clarke (2012)	Not reported	Patients 1) felt fortunate for diagnosis with UIA but were challenged by decision making and concern for rupture, 2) desired treatment by providers with large case volumes, clear communication, and an unbiased approach to decision making, 3) acted on qualitative understandings of individual risk, 4) considered psychological, social, and clinical factors in forming preferences for management, 5) sought information for purposes other than informing decision making, and 6) regained control through decision-making processes. Newly diagnosed patients explored treatment options using online forums. They faced ambiguity in identifying optimal management, creating apprehension and decisional conflict
Finn, Campbell Britton, Rosenberg, et al.	Neurovascular	2019	USA	Not reported	Yale University School of Medicine	All HIC	Journal of Stroke and Cerebrovascular Diseases	***A Qualitative Study of Risks Related to Interhospital Transfer of Patients with Nontraumatic Intracranial Hemorrhage***	To describe providers' perceptions of safety threats during inter-hospital transfer for patients with nontraumatic intracranial hemorrhage.	Unclear	Nontraumatic intracranial hemorrhage (inc. SAH)	USA	Qualitative	Not reported	HCPs (inc. NSx)	21	Interviews (open, unstructured, in-depth, semi-structured, open ended)	Grounded theory analysis/constant comparison/open and axial/selective coding	Thorne (2000) and Guest, MacQueen, Namey (2012)	COREQ	The predominant impediments to safe, high quality neurocritical care transitions between hospitals are insufficient communication, gaps in clinical practice, and lack of IHT structure. Insufficient communication highlights the unique communication challenges specific to inter-hospital transfer, which overlay and compound known intra-hospital communication barriers. Gaps in clinical practice revolves primarily around the provision of neurocritical care for this patient population, often subject to resource availability, by receiving hospital emergency medicine providers. Lack of structure outlines providers' questions that emerge when institutions fail to identify process channels, expectations, and accountability during complex neurocritical care transitions

Ing, Vento, Nakagawa, et al.	Neurovascular	2014	USA	Social worker	Neuroscience Institute	All HIC	Hawai'i journal of medicine & public health : a journal of Asia Pacific Medicine & Public Health	***A qualitative study of transportation challenges among intracerebral hemorrhage survivors and their caregivers***	To identify common driving and transportation barriers among patients with intracerebral hemorrhage (ICH) and their caregivers in the Honolulu community.	Adult	Intracerebral hemorrhage	USA	Qualitative	Not reported	Patients AND Family members, carers, signifcant others	21	Interviews (open, unstructured, in-depth, semi-structured, open ended)	Content analysis: Inductive	Padgett (2008)	Not reported	Participants reported that they needed transportation to attend to their recovery and remain safe. Informal transportation was desired, yet not always available to patients. A local paratransit service for people with disabilities was the most common form of alternative transportation used by patients; however, they reported difficulty obtaining this method of transportation. Participants with no other option used costly, private transportation. Most ICH survivors expressed great challenges with the available transportation services that are essential to their reintegration into the community after hospitalization. Greater effort to provide transportation options and eligibility information to the ICH patients and their caregivers may be needed to improve their post-discharge care.
Ray, Wong, Finn, et al.	Neurovascular	2022	USA	Not reported	Yale school of medicine	All HIC	Journal of Patient Safety	***Improving Safety and Quality during Interhospital Transfer of Patients with Nontraumatic Intracranial Hemorrhage: A Simulation-Based Pilot Program***	To describe the use of an innovative simulation methodology engaging transfer staff, clinicians, and stakeholders to refine and facilitate the adoption of a standardized IHT protocol for transferring patients with neurovascular emergencies.	Adult	Intracranial hemorrhage	USA	Phenomenology	Not reported	HCPs (inc. NSx)	24	Mixed: Telephone based simulations and focus groups	Thematic analysis (inductive)	Not reported	COREQ	Thematic analysis identified 3 IHT-specific themes: (1) challenges unique to multispecialty critical illness, (2) interdisciplinary relationships and dynamics, and (3) communication and information processing for IHT. Three quality improvement initiatives emerged from the debriefings: standardized communication checklist, early acceptance protocol, and structure for telephone-based care handoffs.
Brito, White, Thomacos, et al.	Other	2021	Australia	Occupational Therapist	Department of Occupational Therapy	All HIC	Disability and Rehabilitation	***The lived experience following free functioning muscle transfer for management of pan-brachial plexus injury: reflections from a long-term follow-up study***	1. Investigate the lived-experience of patients following free-functioning muscle transfers for management of traumatic, pan-brachial plexus injuries.<br>2. Better understand issues during recovery and implications for rehabilitation with this population.	Adult	Nerve; Pan-brachial plexus injury	Australia	IPA	Not reported	Patients	5	Interviews (open, unstructured, in-depth, semi-structured, open ended)	IPA	Smith & Osborn (2008)	Not reported	Three interrelated themes were generated from the data. The first theme 'Experience of health care systems' captures the participants' reflections of their post-injury experience and health care received. The second 'Psychosocial considerations' consists of emotional responses, relationship disturbance, and coming to terms with the permanence of their changed arm. The last theme, 'Creating a new self-identity', relates to the participants experience of adjustment to their new circumstances.
Mooney, Hewitt and Hahn	Other	2021	Australia	Not reported	School of Allied Health, Faculty of Health Sciences	All HIC	Disability and Rehabilitation	***Nothing to lose: a phenomenological study of upper limb nerve transfer surgery for individuals with tetraplegia***	To understand the experience of surgery on the lives of individuals with tetraplegia 18 months post-surgery	Adult	Nerve; Upper limb nerve transfer for tetraplegia	Australia	Phenomenology	Not reported	Patients	8	Interviews (open, unstructured, in-depth, semi-structured, open ended)	Phenomenological analysis (Colaizzi)	Carpenter (2013), King & Valle (1978)	Not reported	for individuals with tetraplegia, hope to regain lost upper limb function forms a core consideration in the decision to have surgery. For clinicians supporting patient's decision, balancing hope with the realities of surgery is important. Even small changes in upper limb function had an important influence on participant's confidence in social situations through enhanced participation in a range of everyday activities
De Matteo, Bain, Gjertsen, et al.	Other	2014	Canada	Not reported	McMaster Children's Hospital; School of Rehabilitation Science; CanChild Center for Childhood Disability Research	All HIC	Canadian Journal of Plastic Surgery	***Wondering and waiting' after obstetrical brachial plexus injury: Are we underestimating the effects of the traumatic experience on the families?***	To investigate the impact of having a child with OBPI on the family and whether the Impact on Family Scale (IoFS) can assist in addressing family concerns.	Pediatric	Nerve; Obstetric brachial plexus injury	Canada	Mixed methods (Triangulation method with equal quantitative/qualitative priority.)	Not reported	Family members, carers, signifcant others	18	Mixed: Interviews (open, unstructured, in-depth, semi-structured, open ended) AND questionnaires	Content analysis	Creswell (2003)	Not reported	Themes that emerged from the interviews included traumatic birthing experience, wondering and waiting, and experiencing surgery.
Khu, Bernstein and Midha	Other	2011	Canada	MD/Physician	Division of Neurosurgery	All HIC	Canadian Journal of Neurological Sciences	***Patients' perceptions of carpal tunnel and ulnar nerve decompression surgery***	This study aimed to explore patients' satisfaction and other aspects of the overall experience with this type of surgery.	Adult	Nerve; ulnar decompression surgery; carpal tunnel surgery	Canada	Qualitative	Not reported	Patients	30	Interviews (open, unstructured, in-depth, semi-structured, open ended)	Grounded theory analysis/constant comparison/open, axial and/or selective coding (Modified thematic)	Strauss & Corbin (1990)	Not reported	Four overarching themes emerged from the data: (1) most patients did not perceive their condition to be serious; (2) patients were satisfied with the overall surgical experience; (3) the outcome was more important to patients than the process; and (4) majority of patients had a realistic expectation of outcomes.
Wibroe, Ingersgaard, Larsen, et al.	Other	2021	Denmark	PhD student (specialty not reported)	Department of Neurosurgery and Pediatric Department	All HIC	Acta Neurochirurgica	***Living with the cerebellar mutism syndrome: long-term challenges of the diagnosis***	To explore the patients' experiences related to the sequelae of CMS, to identify challenges and needs regarding support and rehabilitation in the period of growing from child to adult and to add perspectives for future developments of supportive care and rehabilitative guidelines.	Pediatric	Cerebellar mutism syndrome	Denmark	Qualitative (descriptive)	Not reported	Patients	10	Interviews (open, unstructured, in-depth, semi-structured, open ended)	Thematic analysis	Braun & Clarke (2006)	COREQ	Four main themes were identified and highlight the rehabilitative need for focus on verbal and non-verbal communication skills in addition to the physical impairments. We found that brain tumor survivors with CMS can benefit from social and educational rehabilitation, straightforward and truthful information, support in structuring their everyday lives and increased public knowledge of CMS.
Cadotte, Sedney, Djimbaye, et al.	Other	2014	Canada	Neurosurgeon	Division of Neurosurgery, Department of Surgery, University Health Network	Mixed HIC/LMIC	World Neurosurgery	***A qualitative assessment of the benefits and challenges of international neurosurgical teaching collaboration in Ethiopia***	To explore the perspectives of Ethiopian and international neurosurgeons on the development of a sustainable academic neurosurgery teaching unit in Addis Ababa, Ethiopia.	N/A	Education	Ethiopia, Canada, US, Norway	Case study	Not reported	NSx	21	Interviews (open, unstructured, in-depth, semi-structured, open ended)	Grounded theory analysis/constant comparison/open, axial and/or selective coding (modified thematic analysis)	Not reported	Not reported	Several themes emerged that encompass the collective perspective of surgeons who engage in partnerships with the ultimate aim of improving neurosurgical capacity in Ethiopia and local neurosurgeons and trainees. Surgeons describe a rewarding cross-cultural experience the ultimate aim of which is to improve the delivery of neurosurgical care in Ethiopia by assisting in the development and delivery of a residency training program that teaches modern neurosurgical principles and operative techniques. In all cases, such partnerships involve the collaborative efforts of neurosurgeons who trained and worked within a well-established program contained within a functional health care system and Ethiopian neurosurgeons working toward developing similar academic and public health infrastructure.
Adjierteh, E. N. M. and Agbinko-Djogbalar, B. and Lamptey, R. and Pekyi-Boateng, P. K. and Adu, K. O. and Abu-Bonsrah, N.	Other	2024	Ghana	Unclear/not stated	Medical School	Mixed HIC/LMIC	Postgraduate Medical Journal of Ghana	***Factors Influencing the medical student's interest and career choice in neurosurgery***	To determine the factors influencing interest and a career choice in neurosurgery among final year medical students.	N/A	Education	Ghana	Mixed methods,	Non stated	Neurosurgeons (inc. trainees/residents)	3	Open ended survey questions	Thematic analysis	Non stated	Not reported	About 90% of the students expressed interest in neurosurgery. Interest in neurosurgery (70%) and the will to make an impact in the specialty (75%) were the top factors for the 13.8% of students who said they would apply for neurosurgical residency, while the long duration of training deterred 63% of respondents. The students had poor exposure to the clinical aspects of the specialty. Practicing neurosurgeons placed a high premium on student interest in the field.

Verma, Yardi, Puri, et al.	Other	2019	India	Not reported	Department of Physiotherapy, Physiotherapy School & Center	All LMIC	Hand Therapy	***Patients' perception of quality of life after traumatic brachial plexus injury - a qualitative study***	Understanding of an individual patient's perception through their journey of treatment for a traumatic brachial plexus injury	Adult	Nerve; traumatic brachial plexus injury	India	IPA	Not reported	Patients	13	Interviews (open, in-depth, semi-structured, open ended)	IPA	Pietkiewicz & Smith (2014)	Not reported	Eleven subthemes under the overarching theme of patient perception were identified. These subthemes illustrate perception of each individual participants' journey of treatment for traumatic brachial plexus injury.
Giöstad, A. and Carlsson, I. K. and Dahlin, L. B. and Nyman, E.	Other	2024	Sweden	Unclear/not stated	Department of Biomedical and Clinical Sciences,	All HIC	Plos One	***Experience of living with chronic pain in conjunction with surgery for ulnar nerve entrapment at the elbow-A qualitative study***	To explore patients' experiences of living with chronic pain in conjunction with surgery for ulnar nerve entrapment, its consequences in daily life and the coping strategies used to manage it.	Adult		Sweden	Qualitative descriptive	Non stated	Patients	10	Interviews (semi-structured, open, in depth)	Content analysis	Hsieh and Shannon, (2005); Erlingsson and Brysiewicz, (2017)	Not reported	The analysis revealed seven main categories: "Physical symptoms/impairments" and "Mood and emotions"comprise symptoms caused by ulnar nerve entrapment at the elbow and chronic pain; "Consequences in daily life" includes challenges and obstacles in every- day life, impact on leisure activities and social life; "Struggling with self-image" embraces experiences closely related to identity; "Coping strategies" covers adaptive resources; "Experience of relief "describes perceived improvements; "Key message for future care" comprises important aspects for healthcare providers to consider.
Lee, C. Y. and Lee, C. H. and Lai, H. Y. and Yau, S. Y.	Other	2024	Taiwan	Unclear/not stated	Department of Neurosurgery	All HIC	World Neurosurgery	***An Investigation of Patient Preferences and Gender Dynamics of Neurosurgeon Selection in Taiwan: A Mixed-Method Study***	To explore patients' preferences for the physicians' gender in the field of neurosurgical care, aiming to understand its influence on patient satisfaction and experiences	Adult	not specified	Taiwan	Mixed methods,	Non stated	Patients	60	Mixed/multiple: Interviews AND structured questionnaire	Thematic analysis	non stated	Not reported	Qualitative insights indicate that, although professional skill and experience are primary factors in selecting a neurosurgeon, a latent gender preference exists, influenced by perceived privacy, empathetic care, and the surgical finesse associated with female neurosurgeons.
Ashraf, Mohammad and Ismahel, Hassan and Shah, Devansh and Middleton, Eilidh Elizabeth Stewart and Gardee, Ameerah and Chaudhary, Attika and Salloum, Laulwa Al and Evans, Vivienne and Nelson-Hughes, Meaghan and Cheng, Yihui and Goonewardena, Eranga and Ball, Emma and Minnis, Meghan and Anyaegbunam, Gregory Kosisochukwu and Salim, Omar and Bashir, Aneesah Bashir	Other	2024	UK	Unclear/not stated	Glasgow Neuro Society, Wolfson School of Medicine	All HIC	Asian journal of neurosurgery	***Shaping Perceptions and Inspiring Future Neurosurgeons: The Value of a Hands-On Simulated Aneurysm Clipping Workshops at a Student-Organized Neurosurgical Conference***	This study assesses the impact of a hands-on simulated aneurysm clipping workshop on medical students and junior doctors' perceptions of neurosurgery at a student-organized neurosurgical conference.	N/A	Education	UK	Survey/Questionnaire	Non stated	Neurosurgeons (inc. trainees/residents)	96	Open ended survey questions	Thematic analysis	Non stated	Not reported	Thematic analysis revealed that delegates valued the hands-on experience, exposure to microsurgery, and interactions with consultant neurosurgeons. Thirty-six of the 96 delegates (37.5%) expressed that the workshop dispelled preconceived fears surrounding neurosurgery and improved understanding of a neurosurgeon's day-to-day tasks. Several delegates initially apprehensive about neurosurgery were now considering it as a career.
Ashwood, Jerosch-Herold and Shepstone	Other	2019	UK	Occupational therapist	School of Health Sciences	All HIC	Journal of Hand Therapy	***Learning to live with a hand nerve disorder: A constructed grounded theory***	To explore the lived experience of a hand nerve disorder and in particular the impact on body structure/ function, activities, and participation.	Adult	Nerve; Peripheral nerve disorders of the hand	UK	Grounded theory	Constructivist grounded theory	Patients	14	Mixed: Interviews (open, unstructured, in-depth, semi-structured, open ended) AND Visual photograph method	Grounded theory analysis/constant comparison/open, axial and/or selective coding (Conceptual codes were generated by applying the WHO ICF as an analytic scheme)	Charmaz (2014)	Not reported	The impact of hand nerve disorders forms part of a wider narrative on adaptation. A process of "struggling" and then "overcoming" was experienced. This was followed by an interior aspect of adaptation described as "accepting." This gave rise to participants "transforming," being changed as a result of the journey that they had been on.
Bandyopadhyay, Moudgil-Joshi, Norton, et al.	Other	2020	UK	Not reported	Oxford University Global Surgery Group	All HIC	British journal of neurosurgery	***Motivations, barriers, and social media: a qualitative study of uptake of women into neurosurgery***	To explore how social media could be utilized to influence an individual's motivation to pursue a neurosurgical career, an emerging topic area. The focus of this study was on women interested in neurosurgery.	N/A	Neurosurgery	UK	Qualitative	Not reported	Other: Pre-neurosurgical training	30	Interviews (open, unstructured, in-depth, semi-structured, open ended)	Grounded theory analysis/constant comparison/open, axial and/or selective coding (Thematic)	Corbin, Strauss & Strauss (2008)	Not reported	Thirty women participated in the study. Four overarching themes were identified: (1) mentorship, (2) testimony from other women doing neurosurgery, (3) social media as a means of increasing interest in neurosurgery as a career choice, and (4) real-life exposure to the specialty.
Brown, Johnson, Gilbert, et al.	Other	2018	UK	Not reported	Peripheral Nerve Injury Unit	All HIC	Hand Therapy	***The lived experience of motor recovery of elbow flexion following Oberlin nerve transfer: A qualitative analysis***	To give an account through the voice of patients who have undergone surgery with successful restoration of ability to flex the elbow against resistance.	Adult	Nerve, Brachial plexus injuries	UK	Qualitative	Not reported	Patients	6	Focus groups	Other: Using a qualitative approach	Colaizzi (1978)	COREQ	Four main themes were identified as being important components of the lived experience: 'pain', 'patience and positive thought', 'functionality and daily lifestyle' and 'the biceps muscle' itself. Each theme was identified to have several subthemes and constituent parts.

Elliott, Ochieng, Jepson, et al.	Other	2021	UK	Research Fellow in Qualitative Methodology Research.	National Institute for Health Research Bristol Biomedical Research Center, Surgical Innovation Theme, Center for Surgical Research	All HIC	BMJ Open	***"Overnight, things changed. Suddenly, we were in it": A qualitative study exploring how surgical teams mitigated risks of COVID-19***	This paper presents the experiences and innovations developed by international surgical teams during the early stages of the Covid-19 pandemic to attempt to mitigate risk.	Unclear	Surgery (including NSx)	UK	Qualitative	Not reported	HCPs (inc. NSx)	43	Interviews (open, unstructured, in-depth, semi-structured, open ended)	Grounded theory analysis/constant comparison/open, axial and/or selective coding	Glaser & Strauss (1967)	SRQR	Surgical teams sought to mitigate COVID-19 risks by modifying their current practice with an abundance of strategies and innovations. Communication and teamwork played an integral role in how teams adapted, although participants reflected on the challenges of having to improvise in real time. Uncertainties remained about optimal surgical practice and there were significant tensions where teams were forced to balance what was best for patients while contemplating their own safety. The perceptions of risks during a pandemic such as COVID-19 can be complex and context dependent.
Jerosch-Herold, Mason and Chojnowski	Other	2008	UK	Occupational therapist	School of Allied Health Professions	All HIC	Journal of Hand Therapy	***A Qualitative Study of the Experiences and Expectations of Surgery in Patients with Carpal Tunnel Syndrome***	To explore the impact of carpal tunnel syndrome (CTS) on individuals and their expectations of surgical decompression to identify what outcome domains need to be assessed in future clinical trials.	adult	Nerve; Carpal tunnel syndrome	UK	Phenomenology	Phenomenological	Patients	9	Interviews (open, unstructured, in-depth, semi-structured, open ended)	Thematic: content analysis	Patton (2007), Grbich (2007)	Not reported	Although they recognized the consequences of this disorder were minor in comparison to more serious diseases, patients expressed distress at the impact of this disorder on their quality of life and ex- pressed hope that surgery would address this. The assessment of outcomes of surgical decompression of CTS needs to include measures of symptom resolution as well as of activity limitation and participation restriction.
Newington, Brooks, Warwick, et al.	Other	2019	UK	Physiotherapist	Arthritis Research UK – MRC Center for Musculoskeletal Health and Work, MRC Lifecourse Epidemiology Unit, Faculty of Medicine; Hand Therapy	All HIC	BMC Musculoskeletal Disorders	***Return to work after carpal tunnel release surgery: A qualitative interview study***	To explore the return to work experiences of patients who had recently undergone CTR.	Adult	Carpal tunnel release surgery	UK	Qualitative	Not reported	Patients	14	Interviews (open, unstructured, in-depth, semi-structured, open ended)	Framework method	Ritchie and Spencer (1994)	COREQ	Three key themes were identified. Theme 1 Centered on the level of functional disability experienced immediately after surgery. There was an expectation that CTR would be a 'minor' procedure, but this did not match the participants' experiences. Theme 2 explored the desire for validation for the time away from work, with participants recalling a need to justify their work absence to themselves as well as to their employers. Theme 3 focused on the participants' reflections of handing their return to work and function, with many reporting uncertainties about what constituted appropriate activity loads and durations. There was a desire for specific information relating to individual work roles.
Koller, G. M. and Kann, M. R. and Pugazenthi, S. and Koneru, S. and Bhavsar, S. and Strahle, J. M.	Other	2024	USA	Other: Bachelor of Science	Department of Neurosurgery,	All HIC	Journal of Neurosurgery-Pediatrics	***Patient and caregiver perceptions of Chiari malformation: a qualitative analysis of online discussion boards***	To identify the primary concerns expressed on these discussion boards regarding Chiari malformation type I (CM) and to help guide clinicians in understanding patient challenges in the treatment of CM.	Unclear	Chiari malformation	USA	Qualitative	Not explicitly stated	Patients AND Families/carers/ Significant other	400 posts	Online discussion boards	Grounded theory analysis	Corbin and Strauss (1990)	Not reported	Analysis of 400 discussion board posts identified four distinct themes raised by CM patients and their caregivers: the path to diagnosis, symptoms experienced, surgical intervention, and high emotional burden.
Barton, Arant, Blucher, et al.	Other	2021	USA	Not reported	Department of Orthopedic Surgery	All HIC	The Journal of bone and joint surgery. American volume	***Clinician Experiences in Treatment Decision-Making for Patients with Spinal Metastases: A Qualitative Study***	To evaluate the decision-making process for treatment of spinal metastases from the clinician's perspective, including a multi-disciplinary cohort of physicians.	Unclear	Neurosurgery	USA	Qualitative	Not reported	HCPs (inc. Nsx)	14	Mixed: Focus group AND semi-structured interviews.	Thematic analysis	Braun & Clarke (2006)	Not reported	The thematic analysis revealed that numerous factors influence provider-based decision-making for patients with spinal metastases, including clinical elements of the disease process, treatment guidelines, patient preferences and dynamics of the multi-disciplinary care team. The most prominent feature that resonated across all interviews was the importance of multidisciplinary care and the necessity of cohesion between a team of diverse healthcare providers. Respondents emphasized aspects of care team dynamics, including effective communication and intimate knowledge of team-member preferences, as necessary for the development of appropriate treatment strategies. Participants maintained that the primary role in decision-making should remain with the patient.
Conforti, Yaghmour, Hamstra, et al.	Other	2018	USA	Not reported	Milestone Research and Evaluation, Accreditation Council for Graduate Medical Education, Chicago, Illinois	All HIC	Journal of Surgical Education	***The Effect and Use of Milestones in the Assessment of Neurological Surgery Residents and Residency Programs***	To determine the effect of the Accreditation Council for Graduate Medical Education Milestones on the assessment of neurological surgery residents. To determine the feasibility, acceptability, and utility of this new framework in making judgments of progressive competence, its implementation within programs, and the influence on curricula.	N/A	Education	USA	Mixed methods	Not reported	NSx (program directors, residents)	34	Mixed: Interviews (open, unstructured, in-depth, semi-structured, open ended) AND questionnaires	Grounded theory analysis/constant comparison/open, axial and/or selective coding	Strauss & Corbin (1998); Charmaz (2006); Robert Wood Johnson Foundation (2008)	Not reported	Response themes were categorized into 2 groups: outcomes of the Milestones implementation process, and facilitators and barriers. Because of Milestones implementation, participants reported changes to the quality of the assessment process, including the ability to identify struggling residents earlier and design individualized improvement plans. Some programs revised their curricula based on training gaps identified using the Milestones. Barriers to implementation included limitations to the adoption of a developmental progression model in the context of rotation block schedules and misalignment between progression targets and clinical experience. The shift from time-based to competency-based evaluation presented an ongoing adjustment for many programs. Organized preparation before clinical competency committee meetings and diverse clinical competency committee composition led to more productive meetings and perceived improvement in promotion decisions.
Dy, Brogan, Rolf, et al.	Other	2021	USA	MD/Physician	Department of Orthopedic Surgery	All HIC	Bone and Joint Open	***A qualitative study of life satisfaction after surgery for adult traumatic brachial plexus injury***	To better understand patients' overall life satisfaction after BPI, with the goal of identifying areas that can be addressed in future delivery of care.	Adult	Nerve, Brachial plexus injury; peripheral nerve	USA	Qualitative	Not reported	Patients	15	Interviews (open, unstructured, in-depth, semi-structured, open ended)	Thematic analysis (inductive AND deductive)	Beaton & Clark (2009)	Not reported	Among the 15 patients interviewed, the following themes emerged: 1) happiness and life satisfaction were noted despite limitations in physical function; 2) quality of social support influences life satisfaction during recovery from BPI; and 3) social participation and having a sense of purpose impact life satisfaction during recovery from BPI.
Dy, Brogan, Rolf, et al.	Other	2021	USA	MD/Physician	Department of Orthopedic Surgery	All HIC	Journal of Hand Therapy	***Being a patient the rest of my life' The influence of patient participation during recovery after brachial plexus injury***	To better understand the recovery course after surgical reconstruction for BPI, we used qualitative interviews and focused on common points of frustration for patients.	Adult	Nerve, Brachial plexus injury; peripheral nerve	USA	Qualitative (interpretive description)	Not reported	Patients	15	Interviews (open, unstructured, in-depth, semi-structured, open ended)	Thematic analysis (inductive AND deductive)	Beaton & Clark (2009)	Not reported	We interviewed 15 BPI patients at median 13 months after surgery (range: 6-43 months). Our analysis revealed: (1) BPI patients expressed variable degrees of participation during recovery, with the indeterminate state of function making it difficult to adjust to life after BPI. (2) The uncertainty while waiting for improved function is frustrating to BPI patients, with many patients expressing concern for activities and moments they are missing due to injury. (3) While many BPI patients feel left out of decision-making, those who felt engaged in the process expressed less frustration and more acceptance of their status.
Subramanian, Mahajan, Sommaruga, et al.	Other	2018	USA	Not reported	Yale School of Medicine	All HIC	World Neurosurgery	***The Subjective Experience of Patients Undergoing Shunt Surgery for Idiopathic Normal Pressure Hydrocephalus***	To examine the subjective experience of patients treated for iNPH, to identify the challenges patients face and to improve patient outcomes and satisfaction.	Adult	Hydrocephalus (Idiopathic Normal Pressure)	USA	Grounded theory	Not reported	Patients	31	Interviews (open, unstructured, in-depth, semi-structured, open ended)	Grounded theory analysis/constant comparison/open, axial and/or selective coding	Strauss and Corbin (1998)	Not reported	Thirty-one patients who underwent shunt surgery for iNPH were interviewed to reach saturation of themes. Seven themes were identified: 1) long preoperative course causes morbidity; 2) the decision to have shunt surgery is easy to make; 3) patients primarily desire to gain independence; 4) patients show variable levels of anxiety; 5) comorbid conditions interfere with postoperative assessment; 6) patients stand by their decision to have shunt surgery; and 7) outside information is used before surgery.

Cadieux, Healy, Petrusa, et al.	Other	2021	USA / Canada	Neurosurgeon	Harvard Medical School, Department of Neurosciences	All HIC	Medical Teacher	***Implementation of competence by design in Canadian neurosurgery residency programs***	This sequential explanatory mixed-methods study evaluated potential benefits and pitfalls of CBD (competence by design) in Canadian neurosurgery residency education.	N/A	Education	USA	Mixed methods (sequential explanatory)	Not reported	NSx (Residents)	8	Mixed: Interviews (open, unstructured, in-depth, semi-structured, open ended) AND questionnaire	Grounded theory analysis/constant comparison/open, axial and/or selective coding (Thematic)	Not reported	Not reported	Theme 1. Meaning of CBD to first-year neurosurgery residents; Theme 2. Feasibility of using EPAs in neurosurgery; Theme 3. Positive learning experience with feedback; Theme 4. Importance of assessor variability on EPAs
Canty, Breitbart, Siegel, et al.	Paediatric	2019	Canada	Not reported	Division of Neurosurgery	All HIC	Child's Nervous System	***The role of social media in selective dorsal rhizotomy for children: information sharing and social support***	We aimed to assess the use of social media by SDR stakeholders both quantitatively and qualitatively, as well as study their global footprint on popular social media platforms.	Pediatric	selective dorsal rhizotomy, cerebral palsy	International	Mixed methods	Not reported	Other	Other: Social media content: 50 posts, tweets, and comments	Facebook, Twitter, YouTube	Grounded theory analysis/constant comparison/open, axial and/or selective coding (Thematic)	Harris (2015)	Not reported	Qualitative analysis identified seven categories of comments: emotional support and forming connections (22.34%), sharing information and advice (15.96%), appreciation and successes (31.91%), challenges and difficulties (8.51%), advertising/ offering services (4.79%), inequities and access (4.79%), and social media as a second opinion (11.7%).
Crombag, Sacco, Stocks, et al.	Paediatric	2021	Belgium	Not reported	Department of Development and Regeneration Cluster Woman and Child, Biomedical Sciences, KU Leuven/ Elizabeth Garrett Anderson Institute of Women's Health	All HIC	Prenatal Diagnosis	***We did everything we could' a qualitative study exploring the acceptability of maternal-fetal surgery for spina bifida to parents***	To explore the concepts and strategies parents employ when considering maternal-fetal surgery (MFS) as an option for the management of spina bifida (SB) in their fetus, and how this determines the acceptability of the intervention.	Pediatric	Spina bifida; maternal-fetal surgery	Belgium, UK	Qualitative	Framework of Acceptability	Family members, carers, signifcant others	24	Interviews (open, unstructured, in-depth, semi-structured, open ended)	Other: Qualitative analysis	Dierckx et al (2012)	COREQ	To parents opting for MFS, the intervention was perceived as an opportunity that needed to be taken. Feelings of parental responsibility drove them to do anything in their power to improve their future child's situation. Expectations seemed to be realistic yet were driven by hope for the best outcome. None expressed decisional regret at any stage, despite substantial impact and, at times, disappointing outcomes. For the small group of participants, who decided to opt for termination of pregnancy (TOP), MFS was not perceived as an intervention that substantially could improve the quality of their future child's life.
Tavares, Ukawa, Filho, et al.	Paediatric	2020	Brazil	Not reported	Department of Nursing, Botucatu Medical School	All LMIC	World Neurosurgery	***Evaluating Educational Material from the Perspective of Informal Caregivers of Children with Hydrocephalus: A Qualitative Study***	To evaluate whether educational material about hydrocephalus and its treatment previously developed by health professionals corresponded with the daily life of caregivers of children with hydrocephalus.	Pediatric	Hydrocephalus	Brazil	Qualitative	Not reported	Family members, carers, signifcant others	32	Interviews (open, unstructured, in-depth, semi-structured, open ended)	Content analysis: Bardin content analysis methodological framework	Gondim & Bendassolli (2014)	COREQ	The educational material adequately represented caregivers' experience regarding daily life, surgery experiences, and care needed by children with hydrocephalus. In addition, the educational material may help to identify the signs and symptoms of ventriculoperitoneal shunt. However, the material did not address the limitations of children disabled with hydrocephalus in daily life.
Dupont, Dominique and LariviÃ¨re-Bastien, DanaÃ« and Caron, Jeffrey G. and Beaudoin, Cindy and Gravel, Jocelyn and Gagnon, Isabelle and Burstein, Brett and Beaudin, Myriam and Rose, Sean C. and Yeates, Keith O. and Beauchamp, Miriam H.	Paediatric	2023	Canada	Unclear/not stated	Department of Psychology; 2 CHU Sainte-Justine Research Center	All HIC	Journal of pediatric psychology	***"What If?": Caregivers' Experiences Following Early Childhood Concussion***	To capture the experience of caregivers of young children with concussion to inform clinical practice in this distinct developmental group.	Paediatric	mTBI	CAN	Qualitative	Non stated	Family/Carer/Significant other	50	Interviews (semi-structured, open, in depth)	Thematic analysis (inductive)	Braun& Clarke (2006)	Not reported	Four main themes were identified: (1) visible changes associated with caregiver concerns, (2) a roller-coaster of emotions after the injury, (3) healthcare providers' role in addressing the need for reassurance, and (4) the need for better information after the injury.
Ebeye, Tega and Hussain, Ayeh and Brennan, Erin and Kulkarni, Abhaya V. and Forrest, Christopher R. and Riesel, Johanna N.	Paediatric	2024	Canada	Unclear/not stated	Temerty Faculty of Medicine	All HIC	Pediatric neurosurgery	***The Caregiver Burden of Helmet Therapy following Endoscopic Strip Craniectomy: A Phenomenological Qualitative Study***	To generate practical information that may be useful for caregivers and providers navigating treatment options in the management of both positional plagiocephaly and craniosynostosis.	Paediatric	craniosynostosis; plagiocephaly; craniectomy	CAN	Phenomenology (Descriptive)	Non stated	Family/Carer/Significant other	14	Interviews (semi-structured, open, in depth)	Thematic analysis	Morrow, Rodriguez, King (2015)	Not reported	Emerging themes revealed five domains of caregiver burden: emotional, cognitive, physical, psychosocial, and financial. No caregiver felt the therapy was too burdensome to complete. Caregivers of both groups also expressed positive aspects of HT related to support from the team, the noninvasive nature of treatment, and the outcomes of therapy. Furthermore, caregivers report overall satisfaction with the process, stating willingness to repeat the treatment with subsequent children if required.
Fouladirad, Cheong, Singhal, et al.	Paediatric	2022	Canada	Not reported	Division of Neurosurgery	All HIC	Journal of neurosurgery. Pediatrics	***A qualitative study of transitioning patients with hydrocephalus from pediatric to adult care: fear of uncertainty, communication gaps, independence, and loss of relationships***	To better understand the challenges faced by adolescent patients and their parents or caregivers during the transition process	Mixed	Hydrocephalus	Canada	Qualitative	Not reported	Patients AND Family members, carers, signifcant others	40	Interviews (open, unstructured, in-depth, semi-structured, open ended)	Content analysis	Braun & Clarke (2006)	Not reported	Four overarching themes relating to the process of transitioning from pediatric to adult hydrocephalus care for patients and their caregivers were identified from the data: 1) achieving independence, 2) communication gaps, 3) loss of significant relationships and environment, and 4) fear of uncertainty. Overall, patients with hydrocephalus and their families are dissatisfied with the process of transitioning. This study identified common themes and concerns among this cohort that may form the basis of an improved transition model for youth with hydrocephalus as they become adults.

Kuta, Curry, McNeely, et al.	Paediatric	2020	Canada	Not reported	Division of Otolaryngology	All HIC	BMJ Open	***Understanding families' experiences following a diagnosis of non-syndromic craniosynostosis: A qualitative study***	To provide an in-depth qualitative description of families' experiences with craniosynostosis.	Pediatric	Craniosynostosis	Canada	Qualitative	Not reported	Family members, carers, signifcant others	12	Interviews (open, unstructured, in-depth, semi-structured, open ended)	Thematic analysis	Attride-Stirling (2001); Braun & Clarke (2006)	Not reported	Three main themes (six subthemes) emerged from the preoperative interviews: frustration with diagnostic delays (parental intuition and advocacy, hope for improved awareness), understanding what to expect (healthcare supports, interest in connecting with other families) and justifying the need for corrective surgery (influence of the surgeon, struggle with cosmetic indications). Two main themes (four subthemes) were drawn from the postoperative interviews: overcoming fear (the role of healthcare professionals, transition home) and relief (reduction in parental anxiety, cosmetic improvements).
Letourneau, Neufeld, Drummond, et al.	Paediatric	2003	Canada	RN/Nurse	Social Support Research Program	All HIC	Axone	***Deciding on surgery: supporting parents of infants with craniosynostosis***	To: 1) Explore the process of parental decision-making regarding surgery for isolated craniosynostosis 2) Determine what information/support strategies would facilitate parental decision-making	Pediatric	craniosynostosis	Canada	Qualitative (descriptive)	Family centered care	Family members, carers, signifcant others	18	Focus groups	Thematic: content analysis	Not reported	Not reported	Four themes encapsulated the process of parental decision-making. The decision for some parents was agonizing. However, others found the decision relatively straightforward. In all cases, parents spent time thinking and gathering information. Certain critical events helped parents make their decision. Parents identified a number of strategies that would be helpful to the decision-making process.
Pedersen, S. H. and Gustafsen, S. D. and Juhler, M. and Guldager, R.	Paediatric	2024	Denmark	Unclear/not stated	Department of Neurosurgery	All HIC	Brain and Spine	***Parent perception of telemetric intracranial pressure monitoring in children - A qualitative case study***	to create a bridge between the clinician's idea of the telemetric ICP sensor and the patient and parents' personal perceptions of living with the implanted sensor, and thus systematically clarify patient and parent perceptions of system utility and how a long-term implant influence daily life of the patient and family.	Pediatric	ICP monitoring; hydrocephalus	Denmark	Case study	Not explicitly stated	Family/Carer/Significant other	3	Focus groups	Thematic analysis	Graneheim et al. (2004)	Not reported	Three parents participated. Based on thematic analysis, three themes were created: 'Daily living with telemetric ICP monitoring', 'Parenting a child with a CSF disorder', and 'The healthy sibling'. The ICP sensor provided the parents with security and made them trust their intuition, while the possibility of home monitoring ensured stability for the entire family and had a calming effect on healthy siblings. Home monitoring was seen as the system's greatest advantages, whereas size, weight, and functionality of the external monitoring equipment were highlighted as disadvantages.
Granek, Shapira, Roth, et al.	Paediatric	2022	Canada	Not reported	School of Health Policy and Management	All HIC	Pediatric Neurosurgery	***Factors That Influence Intraoperative Decision-Making among Pediatric Neurosurgeons: A Grounded Theory Study***	To explore the factors that influence intraoperative decisions when pediatric neurosurgeons encounter something unexpected or uncertain during surgery.	Pediatric	Neurosurgery	International	Grounded theory	Not reported	NSx	26	Interviews (open, unstructured, in-depth, semi-structured, open ended)	Grounded theory analysis/constant comparison/open and axial/selective coding	Charmaz (2006)	Not reported	Pediatric neurosurgeons reflected on 6 factors while operating in order to come to a decision about how to proceed when they encountered an uncertainty or complication. The study findings resulted in a conceptual model that describes how concrete data including biological and technological factors and contextual data including emotional/relational factors, surgeon factors, and cultural factors influence risk assessment when making an intraoperative decision during surgery.
Granek, Shapira, Constantini, et al.	Paediatric	2021	Canada	Not reported	Faculty of Health, School of Health Policy and Management	All HIC	British Journal of Neurosurgery	***Every patient is like my child': pediatric neurosurgeons' relational and emotional bonds with their patients and families***	To explore the relational and emotional components of the surgeon-patient relationship from the perspective of practicing pediatric neurosurgeons in the field.	Pediatric	Neurosurgery	International	Grounded theory	Not reported	NSx	26	Interviews (open, unstructured, in-depth, semi-structured, open ended)	Grounded theory analysis/constant comparison/open, axial and/or selective coding	Charmaz (2006)	Not reported	Pediatric neurosurgeons find meaning, joy and pleasure in the relationships they form with their patients and their families, while also experiencing difficult and painful emotions when these patients do not do well. Four themes emerged from the analysis that include having a relational attachment to patients, forming bonds with the parents/caregivers of these patients, dealing with patient suffering, death and complications, and communicating bad news to parents.
Granek, Shapira, Constantini, et al.	Paediatric	2021	Canada	Not reported	School of Health Policy and Management	All HIC	Journal of Neurosurgery: Pediatrics	***Pediatric neurosurgeons' philosophical approaches to making intraoperative decisions when encountering an uncertainty or a complication while operating on children***	To explore approaches to intraoperative decision-making in pediatric neurosurgeons when they encounter unexpected events, uncertainties, or complications while operating on children.	Pediatric	Neurosurgery	International	Grounded theory	Not reported	NSx	26	Interviews (open, unstructured, in-depth, semi-structured, open ended)	Grounded theory analysis/constant comparison/open, axial and/or selective coding	Charmaz (2006)	Not reported	When asked to discuss the strategies they used to make intraoperative decisions, neurosurgeons reported three distinct approaches that formed a philosophy of practice. This included the theme of professional practice-with the subthemes of preparing for uncertainty, doing no harm, being creative and adaptive, being systematic, and working on teams. The second theme pertained to patient and caregiver practices-with the subthemes of shared decision-making and seeing the whole patient. The third theme involved surgeon practice-with the subthemes of cultivating self-awareness and learning from experience.
Granek, Shapira, Constantini, et al.	Paediatric	2021	Canada	Not reported	School of Health Policy and Management	All HIC	World Neurosurgery	***How Do Pediatric Neurosurgeons Make Intraoperative Decisions?***	To explore the processes by which pediatric neurosurgeons make intraoperative decisions when they encounter something unexpected or uncertain while they are operating.	Pediatric	Neurosurgery	International	Grounded theory	Not reported	NSx	26	Interviews (open, unstructured, in-depth, semi-structured, open ended)	Grounded theory analysis/constant comparison/open, axial and/or selective coding	Charmaz (2006)	Not reported	Pediatric neurosurgeons (PNs) described a complex process that existed along a spectrum in making intraoperative decisions. Three types of response processes emerged from the analysis: 1) internal processing, with the themes of getting oneself under control and performing control for the surgical team; 2) action processes that included the themes of stabilizing the patient, responding intuitively/automatically when making decisions, and shifting surgical strategies; and 3) analytical processing that involved assessing the situation, consulting with colleagues and the family of the patient when making intraoperative decisions.
Granek, Shapira, Roth, et al.	Paediatric	2021	Canada	Not reported	School of Health Policy and Management	All HIC	Journal of Surgical Education	***Can Good Intraoperative Judgement Be Taught?: Pediatric Neurosurgeons' Pedagogical Approaches to Training Residents on Intraoperative Decision-Making***	To explore how pediatric neurosurgeons train residents in developing intraoperative decision-making judgement.	Pediatric	Neurosurgery	International	Grounded theory	Not reported	NSx	26	Interviews (open, unstructured, in-depth, semi-structured, open ended)	Grounded theory analysis/constant comparison/open, axial and/or selective coding	Charmaz (2006)	Not reported	Pediatric Neurosurgeons used a variety of training approaches that included pre-surgery discussions, didactic communication during surgery, post-surgery debriefing, allowing residents to model and observe their own intraoperative behaviors, using case studies to teach, and ongoing mentorship. In addition, they encouraged residents to ask for help when needed and emphasized the importance of empathy as a surgeon. Challenges to training residents included the notion that decision-making could only be learned through personal experience, the trainee's personality, and an over-reliance on algorithms and standardized medicine.
Capjon and Bjork	Paediatric	2010	Norway	Not reported	Department of Child Neurology	All HIC	Developmental Neurorehabilitation	***Ambulant children with spastic cerebral palsy and their parents' perceptions and expectations prior to multilevel surgery***	(1) What knowledge, motivation and experiences in everyday life influence parents and children in their choice of multilevel surgery? (2) How do parents and children experience cooperation with and between various levels of the health service and school during the pre-operative phase?	Pediatric	Spastic cerebral palsy	Norway	Qualitative	Not reported	Patients AND Family members, carers, signifcant others	22	Interviews (open, unstructured, in-depth, semi-structured, open ended)	Other: Qualitative analysis	Kvale (1997)	Not reported	Everyday life of the children and their parents was vulnerable. The degree to which children strived for social acceptance and normality increased their pain. Deteriorating physical capacity resulted in pain and fatigue and was the parents' and children's main motivation for the operation. Although the parents were ambivalent to the operation they mediated hope and cautious optimism about a better life for their children.

Claude, Juvenal and Hawkes	Paediatric	2012	Republic of Congo	Not reported	HEAL Africa, Goma, Democratic Republic of Congo	Mixed HIC/LMIC	Maternal and Child Nutrition	***Applying a knowledge-to-action framework for primary prevention of spina bifida in tropical Africa***	To define the local disease burden of neural tube defects, then to design, implement and evaluate a culturally tailored educational intervention in eastern Democratic Republic of Congo	Mixed	congenital malformation (spina bifida)	Republic of Congo	Mixed methods	Not reported	Family members, carers, signifcant others	48	Mixed: Focus groups AND questionnaires	Thematic analysis	Not reported	Not reported	A descriptive case series of 27 patients undergoing surgery for spina bifida demonstrated a short-term mortality of 15% and long-term disability in survivors. A survey of knowledge, attitudes and practices demonstrated a low level of folate awareness (53%) among women of reproductive age. Focus group discussions revealed exotic aetiologic views, significant gender issues and several barriers to folate use. A culturally tailored radio broadcast and an educational video were designed and produced locally based on qualitative and quantitative findings. Evaluation of the video documented high levels of viewer satisfaction and unequivocal knowledge gain.
Zerpe, A. S. and Ramklint, M. and Nowinski, D. and Ã–ster, C.	Paediatric	2024	Sweden	Nurse/RN/RGN	Department of Surgical Sciences, Plastic and Maxillofacial Surgery	All HIC	Journal of Pediatric Nursing-Nursing Care of Children & Families	***Parental satisfaction with hospital care for children with non-syndromic craniosynostosis: A mixed-method study***	to investigate factors influencing parents' satisfaction with hospital care for children with craniosynostosis during hospitalization for surgery.	Pediatric	Craniosynostos is	Sweden	Mixed methods (convergent, parallel design)	Not explicitly stated	Family/Carer/Sig nificant other	20	Mixed/multiple: Interviews AND structured questionnaire	Content analysis	Elo and Kyngas (2008)	Not reported	Parents' assessment of the overall quality of care was high (mean 87%, range 10–100%). They were most satisfied in the domain staff attitudes and less satisfied with information routines and participation. Content analysis of the interviews gave two overarching themes: Factors that parents experienced as facilitating good quality of care and Factors that parents experienced as impeding good quality of care.
Dangsomboon and Jirapaet	Paediatric	2017	Thailand	Not reported	Faculty of Nursing	All LMIC	Journal of Health Research	***Experiences of caregivers having children with craniosynostosis using distractor devices***	To examine the lived experience of caregivers having children with craniosynostosis using distractor devices from their point of view.	Pediatric	craniosynostosi s	Thailand	Phenomenology	Philosophy of Husserl	Family members, carers, signifcant others	14	Interviews (open, in-depth, semi-structured, open ended)	Thematic: Content analysis	Colaizzi's method	Not reported	A thematic content analysis of the interview data revealed five major themes of caregivers' experiences. Those were as follows: 1) learning and following the hospital advice, 2) facing difficulties when returning home, 3) challenging in taking care of the moving child, 4) having mixed feelings and 5) having support and help from others and build-up of inner strength.
Chugh, Deepti and Waite, Gillian and Harniess, Phillip and Oulton, Kate and Wray, Jo and Cawker, Stephanie	Paediatric	2024	UK	Unclear/not stated	Physiotherapy Department	All HIC	Physical & occupational therapy in pediatrics	***'I Didn't Know What Was Going to Happen': Children's and Young People's Experiences and Their Involvement Before and After Selective Dorsal Rhizotomy***	To explore CYP's experiences and gain insight into their involvement during SDR and intensive rehabilitation.	Pediatric	Cerebral palsy; Selective Dorsal Rhizotomy	UK	Qualitative	Social constructivist	Patients	5	Interviews (semi-structured, open, in depth)	Thematic analysis	Braun & Clarke (2006)	Not reported	Children and young people are reliant on their parents to make decisions and inform them of the selective dorsal rhizotomy process. Experiences of living with cerebral palsy and its management are centered on their routine social, psychological, and physiological challenges. Individual characteristics and attributes of CYP have an impact on how they cope with the rehabilitation burden and adjust to their changing levels of function and participation.
Silva, Wijesinghe, Mundil, et al.	Paediatric	2019	UK	Not reported	Department of Neurosurgery	All HIC	Child's Nervous System	***Consent in Pediatric neurosurgery: adequacy of documentation and parental perspectives***	We aimed to prospectively evaluate the consenting process in our department to assess adequacy of documentation and parental perspectives.	Pediatric	Neurosurgery	UK	Qualitative (descriptive)	Not reported	Family members, carers, signifcant others	50	Questionnaire	Content analysis	Ellamushi, Khan, Kitchen (2000)	Not reported	All patients understood the primary diagnosis and type of surgery. Procedure-specific risks were understood by 98% and 84% could remember the mentioning of general risks of surgery. Only a minority of parents (24%) could recollect that alternative options of management including no treatment were discussed. In cases where relevant, laterality was only documented in 56% of consent forms. All patients felt that an informed decision regarding consent to surgery was made. However, 12% suggested areas where further improvement could be made in the timing of consent and the way information could be better provided.
Smith, Cheater and Bekker	Paediatric	2015	UK	RN/Nurse	Department of Health Studies, Division of Maternal and Child Health	All HIC	Health expectations : an international journal of public participation in health care and health policy	***Parents' experiences of living with a child with hydrocephalus: a cross-sectional interview-based study***	To explore parents' experiences of living with a child with hydrocephalus and their decisions when they suspect shunt malfunction.	Pediatric	Hydrocephalus	UK	Qualitative	Not reported	Family members, carers, signifcant others	25	Interviews (open, unstructured, in-depth, semi-structured, open ended)	Framework analysis	Smith & Firth (2011)	Not reported	Three concepts, 'uncertainty', 'developing expertise', and 'a normal life', were identified. These concepts were dynamic in nature as parents learned through experience, adapted to changes in their child's health status and made decisions about their needs. Uncertainty because of the unpredictability and life-threatening nature of shunt malfunction dominated parents' accounts. Through experience, parents learned to differentiate between symptoms that suggested a shunt problem and those of other childhood illnesses, but perceived their expertise was not always valued by health professionals or used to inform clinical decisions. Decisions about where or when to seek advice related to prior experiences of health-care services and minimizing disruption for the whole family.
Barnes, K. and Zimmerman, K. and Herbey, I. and Arynchyna-Smith, A. and May, B. and Wessinger, C. A. and Dreer, L. E. and Thompson, L. and Ivankova, N. V. and Rozzelle, C. J. and Johnston, J. M. and Blount, J. P. and Rocque, B. G.	Paediatric	2023	USA	Physician/MD	Department of Pediatrics, Division of Neurology, Department of Neurosurgery, Division of Pediatric Neurosurgery	All HIC	Journal of Neurosurgery-Pediatrics	***Understanding and identifying the needs of parent caregivers of children with hydrocephalus: a qualitative study***	To inform directions for clinical practice and research targeting pediatric patients with hydrocephalus and their caregivers	Pediatric	Hydrocephalus	USA	Qualitative	Non stated	Family/Carer/Sig nificant other	18	Interviews (semi-structured, open, in depth)	Thematic analysis (Inductive)	Guest, MacQueen, and Namey (2011)	Not reported	Five major themes emerged: 1) coping with the diagnosis, 2) received support, 3) hydrocephalus management, 4) implications for intervention, and 5) psychosocial stressors for caregivers. A top priority was balanced, trustworthy information delivered with compassion and updated throughout the child's life. Caregivers described a variety of coping strategies, but a majority reported a need for support in processing complex emotions and dealing with the uncertainty of their child's hydrocephalus. Most agreed that having a caregiver support network, medical professionals available for referrals and questions, and referrals to support services and therapies would facilitate feeling supported and providing the best care for their children.
Colon, G. D. R. and Merrell, S. B. and Mahaney, K. B. and Poon, D. C. and Maher, C. O. and Prolo, L. M.	Paediatric	2024	USA	Unclear/not stated	Departments of Neurosurgery and Pediatrics	All HIC	Journal of Neurosurgery-Pediatrics	***Language-discordant care in pediatric neurosurgery: parent and provider perspectives on challenges and multilevel solutions to reduce disparities***	to identify challenges faced by pediatric neurosurgery providers and Spanish-speaking parents communicating with a language barrier and propose solutions to address those challenges.	Paediatric	Mixed NSx	USA	Phenomenology	Not stated	Mixed: Parents AND NSx AND HCPs AND non-clinicians	20	Interviews (semi-structured, open, in depth)	Thematic analysis	Braun and Clarke (2006)	SRQR	Three challenges were identified. 1) Compared with English-speaking parents, providers noted that Spanish-speaking parents were less likely to ask questions or raise new concerns. Concurrently, Spanish-speaking parents expressed a desire to better understand their child's future medical needs, care, and development. 2) There is a dearth of high-quality resources available in the Spanish language to supplement patient and parent neurosurgical education. 3) Both parents and providers invariably prefer in-person interpreters; however, their availability is limited.
Yoder, E. M. and Davies, S. C. and Montgomery, M. and Lundine, J. P.	Paediatric	2024	USA	Unclear/not stated	Department of Counselor Education and Human Services	All HIC	Disability and Rehabilitation	***Exploring the care coordination experiences of professionals and caregivers of youth with acquired brain injuries in rural areas***	to explore the experiences of rural adults caring for children with ABIs	Paediatric	MIXED ABI	USA	Qualitative	Not explicitly stated	Mixed (Caregivers; school staff, medical professionals)	12	Interviews (semi-structured, open, in depth)	Framework analysis	Gale et al. (2013)	Not reported	Themes in their accounts include difficulty navigating complex situations, support from small communities, isolation and loneliness, the need for more professional education about ABI, and feelings of hope. Barriers to quality care coordination include navigating complex situations, access to transportation, and a lack of communication and education from healthcare agencies. Facilitators of rural care coordination include support from small communities and interagency communication.

Bemmels, Biesecker, Schmidt, et al.	Paediatric	2013	USA	Doctoral student	Department of Educational Psychology	All HIC	Cleft Palate-Craniofacial Journal	***Psychological and social factors in undergoing reconstructive surgery among individuals with craniofacial conditions: An exploratory study***	To explore the diversity of reconstructive surgery experiences; to understand the psychological and social outcomes resulting from these procedures; to highlight the range of parental influence in decision making around childhood surgery for CFCs; and to share participant recommendations for parents of children with CFCs considering surgery.	Mixed	craniofacial conditions	USA	Qualitative (secondary analysis)	Not reported	Patients	38	Interviews (open, unstructured, in-depth, semi-structured, open ended)	Other: Systematic line by line coding	Strauss and Corbin, (1998); Sandelowski (2000)	Not reported	Dominant themes included undergoing surgery to reduce stigmatization, the psychological and social implications of the interventions, outcome satisfaction, parental involvement in decision making about surgery, and recommendations for parents considering surgery for their children with craniofacial conditions. Experiences with reconstructive surgery varied, with some participants expressing surgical benefits and others, disillusionment.
Squitieri, Larson, Chang, et al.	Paediatric	2013	USA	MD/Physician	Department of Surgery, division of plastic and reconstructive surgery	All HIC	Plastic and Reconstructive Surgery	***Medical decision-making among adolescents with neonatal brachial plexus palsy and their families: A qualitative study***	To explore and describe the medical decision-making process among children and adolescents with NBPP from the patient and family perspective using qualitative research methods.	Adolescents	Nerve; Neonatal Brachial Plexus Palsy	USA	Grounded theory	Not reported	Patients AND Family members, carers, signifcant others	36	Interviews (open, unstructured, in-depth, semi-structured, open ended)	Grounded theory analysis/constant comparison/open, axial and/or selective coding	Beaton & Clark (2009); Shauver & Chung (2010)	Not reported	Medical decision-making among adolescents with NBPP and their families is multifaceted and individualized, comprised of both patient and system dependent factors. Four codes pertaining to the medical decision-making process were identified: 1) knowledge acquisition, 2) multidisciplinary care, 3) adolescent autonomy, and 4) patient expectations and treatment desires. Overall, parental decision-making was heavily influenced by system dependent factors, while adolescents largely based their medical decision-making on individual treatment desires to improve function and/or aesthetics.
Squitieri, Larson, Chang, et al.	Paediatric	2013	USA	MD/Physician	Department of Surgery, division of plastic and reconstructive surgery,	All HIC	Journal of Hand Surgery	***Understanding Long-Term Outcomes and Patient Expectations Among Adolescents with Neonatal Brachial Plexus Palsy: A Qualitative and Quantitative Study***	To explore the long-term QOL and patient expectations among adolescents with NBPP using qualitative and quantitative approaches	Adolescents	Nerve; Neonatal Brachial Plexus Palsy	USA	Grounded theory	Not reported	Patients AND Family members, carers, signifcant others	36	Mixed: Interviews (open, unstructured, in-depth, semi-structured, open ended) AND questionnaire	Grounded theory analysis/constant comparison/open, axial and/or selective coding	Beaton & Clark (2009); Shauver & Chung (2010)	Not reported	Through qualitative analysis we identified the following factors contributing to overall QOL from the patient and parent perspective: social impact and peer acceptance, emotional adjustment, aesthetic concerns and body image, functional limitations, physical and occupational therapy, finances, pain, and family dynamics. Despite residual impairment, most adolescents and their parents reported a good overall QOL according to quantitative outcome measures, with adolescents reporting slightly higher QOL than their parents. However, both adolescents and their parents report relatively modest satisfaction with their current condition and express expectations for improvement in multiple areas.
Tapia, Drizin, Ore, et al.	Paediatric	2017	USA	Not reported	Division of Plastic Surgery	All HIC	Annals of Plastic Surgery	***Qualitative methods in the development of a bilingual and bicultural quality of life outcomes measure for pediatric patients with craniofacial conditions***	To develop the items and support the content validity of a comprehensive patient and parent-reported outcomes measure.	Pediatric	craniofacial conditions	USA	Qualitative	CFC-associated HRQoL	Patients AND Family members, carers, signifcant others	127	Interviews (open, unstructured, in-depth, semi-structured, open ended)	Content analysis (line by line)	Not reported	Not reported	Six final bilingual and bicultural scales based on the domains derived from the literature review, expert opinion, and in-depth interviews were developed: (1) "Social Impact," (2) "Psychological Function," (3) "Physical Function," (4) "Family Impact," (5) "Appearance," And (6) "Finding Meaning." Some cultural differences were identified: in contrast to children from Mexico and other developing nations, families from the United States did not report public harassment or extremely negative public reactions to patients' CFC. Religion and spirituality were common themes in interviews of Spanish-speaking subjects but less common in interviews of English-speaking subjects.
Torgerson, Munoz, Kostick, et al.	Paediatric	2021	USA	Not reported	Center for Medical Ethics and Health Policy, Baylor College of Medicine,	All HIC	Neuromodulation : journal of the International Neuromodulation Society	***Clinical and Psychosocial Factors Considered When Deciding Whether to Offer Deep Brain Stimulation for Childhood Dystonia***	To identify and examine the factors considered by pediatric movement disorder specialists before offering DBS.	Pediatric	Childhood Dystonia; Deep brain stimulation	USA	Qualitative	Not reported	HCPs (inc. NSx)	29	Interviews (open, unstructured, in-depth, semi-structured, open ended)	Thematic: Content analysis	Not reported	Not reported	Clinicians identified nine main factors. Five of these were classified primarily as clinical factors: early intervention and younger age (raised by 86% of respondents), disease progression and symptom severity (83%), etiology and genetic status (79%), clinicians' perceived risks and benefits of DBS for the patient (79%), and exhaustion of other treatment options (55%). The remaining four were classified primarily as psychosocial factors: social and family support (raised by 97% of respondents), patient and caregiver expectations about outcomes and understanding of DBS treatment (90%), impact of dystonia on quality of life (69%), and financial resources and access to care (31%).
Williams, Eriksson, Piantino, et al.	Paediatric	2018	USA	MD/Physician	Division of Pediatric Critical Care, Department of Pediatrics,	All LMIC	Journal of pediatric intensive care	***Long-term Sequelae of Pediatric Neurocritical Care: The Parent Perspective***	To determine outcomes important to patients and families, identify barriers to care, and identify potential interventions to improve outcomes.	Pediatric	Mixed acquired brain injury (TBI, stroke, meningitis, or encephalitis)	USA	Qualitative	Not reported	Family members, carers, signifcant others	16	Focus groups	Thematic: network analysis	Attride-Stirling (2001)	Not reported	Three global themes were identified: (1) PNCC is an intense emotional experience for the whole family; (2) PNCC survivorship is a chronic illness; and (3) PNCC has a significant psychological and social impact. Survivors and their families suffer physical, emotional, psychological, cognitive, and social impairments for many years after discharge. Parents in this study highlighted the emotional and psychological distress in survivors and families after PNCC, in contrast to most PNCC research focusing on physical outcomes. Several barriers to care were identified with potential implications on survivor outcomes, including limited pediatric resources in rural settings, perceived lack of awareness of PICS among medical providers, and the substantial financial burden on families. Parents desire improved education surrounding PICS morbidities for families and medical providers, improved communication with primary care providers after discharge, access to educational materials for patients and families, direction to mental health providers, and family support groups to assist them in dealing with morbidities and accessing appropriate resources.
Kostick-Quenet, K. M. and Kalwani, L. and Torgerson, L. N. et al	Paediatric	2023	USA	Other: medical anthropologist, qualitative method expert	Center for Medical Ethics and Health Policy	All HIC	Stereotactic and Functional Neurosurgery	***Deep Brain Stimulation for Pediatric Dystonia: Clinicians' Perspectives on the Most Pressing Ethical Challenges***	to identify ethical challenges and potential solutions to ensure responsible use of DBS in pediatric populations.	Paediatric	DBS, dystonia	USA, UK, France, Canada	Qualitative	Non stated	HCPs (inc. NSx)	29	Interviews (semi-structured, open, in depth)	Thematic content analysis	Boyatzis (1998)	Not reported	Using thematic content analysis to explore salient themes, clinicians identified four pressing concerns: (1) uncertainty about risks and benefits of pDBS (22/29; 72%) that poses a challenge to informed decision-making; (2) ethically navigating decision-making roles (15/29; 52%), including how best to integrate perspectives from diverse stakeholders (patient, caregiver, clinician) and how to manage surrogate decisions on behalf of pediatric patients with limited capacity to make autonomous decisions; (3) information scarcity effects on informed consent and decision quality (15/29; 52%) in the context of patient and caregivers' expectations for treatment; and (4) narrow regulatory status and access (7/29; 24%) such as the lack of FDA-approved indications that contribute to decision-making uncertainty and liability and potentially limit access to DBS among patients who may benefit from it.

Simpamba, Struthers and Mweshi	Paediatric	2016	South Africa	Not reported	Department of Physiotherapy	All LMIC	African journal of disability	***Access to health care for children with neural tube defects: Experiences of mothers in Zambia***	To explore the experiences of mothers accessing health care who had recently given birth to a child with a neural tube defect.	Pediatric	Neural tube defect	Zambia	Not stated	Four dimensions of the Access to Care Framework,	Family members, carers, signifcant others	20	Interviews (open, unstructured, in-depth, semi-structured, open ended)	Thematic analysis	Green & Thorogood (2009)	Not reported	Five themes emerged: access to health care, access to transport, access to information, concerns about family and support needs. Barriers to access to health care included geographical barriers and barriers linked to availability. Geographical barriers were related to distance between home and the health Center, and referral between health facilities. Barriers to availability included the lack of specialist health workers at various levels, and insufficient hospital vehicles to transport mothers and children to the tertiary level hospital. The main barrier to affordability was the cost of transport, which was alleviated by either family or government support. Acceptability of the health services was affected by a lack of information, incorrect advice, the attitude of health workers and the beliefs of the family.
Wilson, Saling, Kincade, et al.	Skull-base	1998	Australia	Not reported	Department of Neuropsychology	All HIC	Epilepsia	***Patient expectations of temporal lobe surgery***	To examine expectations of postoperative quality of life expressed by patients undergoing anterior temporal lobectomy (ATL) for the control of intractable seizures.	Mixed	Temporal lobe surgery	Australia	Qualitative	Not reported	Patients	60	Interviews (open, unstructured, in-depth, semi-structured, open ended)	Content analysis	Not reported	Not reported	A range of expectations were expressed about postoperative outcome. These were classified into 11 posthoc categories. Patients who perceived the operation as a success tended to endorse 'practical' expectations (i.e., driving, employment, activities) preoperatively, rather than expectations of a psychologic or social nature (i.e., self-change, relationships). These patients experienced fewer postoperative seizures and psychosocial difficulties. In contrast, a perceived lack of success was associated with greater emphasis on psychosocial expectations preoperatively. These patients experienced a greater number of perceived postoperative psychosocial difficulties, and more postoperative seizures.
De Almeida, Vescan, Witterick, et al.	Skull-base	2015	Canada	Not reported	Department of Otolaryngology-Head and Neck Surgery	All HIC	Journal of Neurological Surgery, Part B: Skull Base	***Changes experienced in quality of life for skull base surgical patients: A qualitative case study***	Skull base tumors are associated with quality of life (QOL) changes. A qualitative case-study approach may help better understand patients' experiences.	Adult	Skull base tumors	Canada	Case study	Post-positivist	Patients	34	Focus groups	Content analysis	template style of analysis' by Miles and Huberman (1994); Yin (1994)	Not reported	The 34 participants (19 men, 15 women; mean age: 48 years, standard deviation: 14 years) had mixed reactions to their diagnosis ranging from relief to fear. Participants reported physical and nonphysical changes in QOL with some variation in physical complaints by tumor location. Several major themes emerged from the analysis. Skull base tumors are associated with fear and frustration, loss of physical senses and self-identity, social isolation, and coping mechanisms.
Edem, Banton, Bernstein, et al.	Skull-base	2013	Canada	Not reported	Division of Neurosurgery	All HIC	British Journal of Neurosurgery	***A prospective qualitative study on patients' perceptions of endoscopic endonasal transsphenoidal surgery***	To explore patients' perceptions on endoscopic transsphenoidal surgery.	Adult	Skull base surgery; transsphenoidal surgery	Canada	Qualitative	Not reported	Patients	23	Interviews (open, unstructured, in-depth, semi-structured, open ended)	Grounded theory analysis/constant comparison/open, axial and/or selective coding (modified thematic analysis)	Kennedy & Lingard (2006)	Not reported	Seven overarching themes were identified: (1) Patients had a positive surgical experience; (2) patients were satisfied with the results of the procedure; (3) patients were initially surprised that neurosurgery could be performed endonasally; (4) patients expected a cure and to feel better after the surgery; (5) many patients feared that something might go wrong during the surgery; (6) patients were psychologically prepared for the surgery; (7) most patients reported receiving adequate pre-op and post-op information.
Lwu, Edem, Banton, et al.	Skull-base	2012	Canada	Not reported	Division of Neurosurgery	All HIC	Acta neurochirurgica	***Quality of life after transsphenoidal pituitary surgery: a qualitative study***	To compare the patients' perceptions of their postoperative recovery periods following microscopic and endoscopic procedures.	Adult	Pituitary tumor; Rathke's cleft cyst; transsphenoidal resections	Canada	Qualitative	Not reported	Patients	27	Interviews (open, unstructured, in-depth, semi-structured, open ended)	Thematic analysis (modified)	Not reported	Not reported	The following five overarching themes emerged from the data: (1) the endoscopic procedure was better tolerated than the microscopic procedure and was the preferred approach by 22 out of 27 patients should they require another surgery in the future; (2) most patients did not know that they had undergone two different surgical approaches; (3) other than an unpleasant malodorous smell, rhinologic complications (including drainage, crusting, and alterations in smell) following the endoscopic procedures were compa-rable to those following the microscopic procedures; (4) the patient's postoperative experience after the microscopic procedure had an impact on his/her expectations of the endoscopic procedure; (5) any significant pain or discomfort experienced from either procedure was mainly related to the nasal packing or fascia lata graft donor site.
Wihlidal, Bysice, Rammal, et al.	Skull-base	2022	Canada	Not reported	Head and Neck Surgery, Schulich School of Medicine and Dentistry	All HIC	Facial Plastic Surgery & Aesthetic Medicine	***Thematic Analysis of Canadian Patient-Reported Outcomes in Facial Nerve Paralysis: A Combined Interpretive Description and Modified Delphi Approach***	To elicit patient-identified themes of importance pertaining to disease course in facial nerve dysfunction from a variety of etiologies.	Adult	Facial Nerve Paralysis	Canada	Qualitative (Interpretive description) AND modified Delphi	Not reported	Patients	24	Mixed: Interviews (open, unstructured, in-depth, semi-structured, open ended) AND modified Delphi	Grounded theory analysis/constant comparison/open, axial and/or selective coding	Not reported	Not reported	Upon thematic analysis of 315 codable phrases, 33 codes were sorted into six domains. In descending order: smiling, facial symmetry, surgical access, self-consciousness, eye care, eating, lip movement, eye closure, beverage consumption, speech, chewing, drooling, eyebrow raise, mouth closure, and ptotic vision limitations were identified as the most important aspects of disease course. Care experience, defined as areas of interaction with the health care system in which patients felt strongly about their care or outcome, was the most important domain to participants.
Zerpe, Nowinski, Ramklint, et al.	Skull-base	2022	Sweden	RN/Nurse	Department of Surgical Sciences	All HIC	Journal for Specialists in Pediatric Nursing	***"When the surgery was over, I felt like the worst part had passed": experiences of parents of children with craniosynostosis***	To explore parents' experiences of hospital care after their child's craniosynostosis surgery and their perception of support during the year after discharge.	Pediatric	Nonsyndromic craniosynostosis	Sweden	Qualitative	Not reported	Family members, carers, signifcant others	19	Interviews (open, unstructured, in-depth, semi-structured, open ended)	Content analysis: Inductive	Elo & Kyngas (2008)	Not reported	The analysis yielded six categories with subcategories as follows: (1) cared for and confident: the hospital staff was perceived as kind, professional, and reliable. (2) Alone and abandoned: sometimes, parents found it hard to initiate contact with professionals during hospitalization and after discharge. (3) The importance of information: thorough information was perceived as essential and the need for information varied during postsurgery period. (4) Feelings of worry: some parents remained worried about risks during recovery and were concerned about comorbidities and development. (5) Alright after all: parents felt that the worst part had been before surgery. (6) The need for support: parents were generally satisfied with the support offered and they often received support from family and friends, or other parents through social media/online forums.

Zerpe, Nowinski, Ramklint, et al.	Skull-base	2020	Sweden	RN/Nurse	Department of Surgical Sciences	All HIC	Journal of Craniofacial Surgery	***Parents' Experiences of Their Child's Craniosynostosis and the Initial Care Process***	To investigate Swedish parents' experiences of having a child with craniosynostosis and their perceptions of the initial care process.	Pediatric	Non-syndromic craniosynostosis	Sweden	Qualitative	Not reported	Family members, carers, signifcant others	20	Interviews (open, unstructured, in-depth, semi-structured, open ended)	Thematic analysis	Braun & Clarke (2006)	Not reported	A thematic data analysis revealed 6 themes presented in a timeline following the parents' journey from detection of their child's abnormal skull shape to waiting for surgery: Detection of the abnormal skull shape, thoughts, and feelings before the appointment with the craniofacial team, an appointment with the craniofacial team, searching the Internet and social media, waiting for surgery, and suggestions for improvement. Although meeting with the craniofacial team was considered informative, parents expressed concerns about surgery and their infant's long-term prognosis were evident. Most parents had no previous knowledge about craniosynostosis and craniofacial syndromes and wished for more information already at the time of its detection. The Internet was used both at the time of suspicion that something was wrong with the child and later to learn about risks and consequences, alternative treatments and prognosis.
Stewart, K. and Price, G. and Kelderman, J. and Carman, S. and Imms, C. and Wallen, M.	Spinal surgery	2024	Australia	Unclear/not stated	Kids Rehab, The Children's Hospital	All HIC	Disability and Rehabilitation	***Caregiver perspectives of scoliosis surgery for children with cerebral palsy: a qualitative study***	To explore the perspectives of primary caregivers of children with cerebral palsy (CP) who had spinal surgery for scoliosis.	Mixed	Scoliosis surgery of patients with cerebral palsy	Australia	Qualitative	Interpretivism	Family/Carer/Significant other	14	Interviews (semi-structured, open, in depth)	Thematic analysis	Braun & Clarke (2022)	Not reported	Four themes were identified emerged. Life with a child with CP underpinned all experiences which were founded on familiarity with their child, medical procedures, and hospitalisation. Three subthemes were parents are the experts in knowing their child, children are vulnerable, and impact on caregivers. Theme 2 involved the significance of decision making to proceed with surgery. Theme 3 underscored a need to be prepared for the surgical journey and, in Theme 4, participants spoke of needing to expect the unexpected.
Marr, Leonard, Torode, et al.	Spinal surgery	2015	Australia	Not reported	School of Physiotherapy and Exercise Science	All HIC	Child: Care, Health and Development	***Spinal fusion in girls with Rett syndrome: Post-operative recovery and family experiences***	To understand more about treatment effectiveness and acceptability to families, the current study recruited families from the ARSD and explored the course of their daughter's recovery following discharge from hospital and family perspectives.	Mixed	Spine; Rett syndrome	Australia	Not stated	Not reported	Family members, carers, signifcant others	25	Interviews (open, unstructured, in-depth, semi-structured, open ended)	Content analysis	Liamputtong (2009).	Not reported	Pain and energy levels, appetite, mood and coinciding health issues influenced their daughter's post-operative recovery. The majority of girls recovered preoperative sitting (88%), standing (81%) and walking (80%) by 12 months. The decision to proceed with surgery was associated with feelings of fear, obligation, relief and guilt for families. Development of complications, poor support and feelings of isolation increased their emotional burden whereas adequate information and discharge preparation, confidence in self and staff, and balancing personal needs with their daughter's care relieved this burden.
Gorgon, Maka, Kam, et al.	Spinal surgery	2022	Australia / Philippines	Physiotherapist	Sydney School of Health Sciences, Faculty of Medicine and Health & Department of Physical Therapy	Mixed HIC/LMIC	Health Expectations	***Needs assessment for health service design for people with back pain in a hospital setting: A qualitative study***	To determine patients' perceived needs and barriers to best-practice back pain care, and potential solutions to better address care needs.	Adult	Spine; Back pain	Australia	Qualitative (descriptive)	Interpretive description approach	Patients	24	Mixed: In-depth interviews AND focus groups	Thematic analysis	Braun & Clarke (2006, 2019)	COREQ	The analysis identified three key themes with several subthemes around what service designers needed to understand in helping people with back pain in this setting: (1) This is who I am; (2) It's not working for me; and (3) What I think I need.
Vigouroux, M. and Newman, G. and Amja, K. and Hovey, R. B.	Spinal surgery	2023	Canada	Unclear/not stated	Department of Integrated Studies in Education, Faculty of Education and Institute for Gender, Sexuality and Feminist Studies, Faculty of Arts	All HIC	Frontiers in Pain Research	***"He told me my pain was in my head": mitigating testimonial injustice through peer support***	To explore the effects and mechanisms of gender-specific peer support in disrupting this trajectory to adverse outcomes.	Adolescents	Scoliosis	Canada	Qualitative	Intersectionality and Testimonial Injustice	Patients	16	Interviews (semi-structured, open, in depth)	Applied philosophical hermeneutics	Hovey (2022)	Not reported	They found that the study participants had their pain narratives reinterpreted by the adults in their lives, including their parents and healthcare practitioners, leading them to question and doubt their own experiences.These negative outcomes were mitigated through the peer support they received and offered from Curvy Girls. Participants reported having gained confidence and a sense of belonging after they joined this group, allowing them to better cope with their condition more effectively in different facets of their lives.
Hersht, Massicotte and Bernstein	Spinal surgery	2007	Canada	Neurosurgeon	Division of Neurosurgery	All HIC	Canadian journal of surgery. Journal canadien de chirurgie	***Patient satisfaction with outpatient lumbar microsurgical discectomy: a qualitative study***	To investigate patient satisfaction with the experience of outpatient lumbar microdiscectomies	Mixed	Spine; microdiscectomy	Canada	Case study	Not reported	Patients	28	Interviews (open, unstructured, in-depth, semi-structured, open ended)	Grounded theory analysis/constant comparison/open, axial and/or selective coding (Modified thematic)	Sheldon (1998); Strauss & Corbin (1998)	Not reported	Analysis of the interviews yielded several overarching themes: 1) patients are surprised that back surgery can be done on an outpatient basis; 2) the amount and quality of information they receive is satisfactory; 3) the overall experience is positive; 4) trust in one's surgeon is important; and 5) some patients have significant back pain in the early postoperative period.
Li, Larche, Vickers, et al.	Spinal surgery	2022	Canada	Not reported	McGill University; Shriners Hospitals for Children-Canada,	All HIC	Journal of Patient Experience	***Experience and Management of the Adverse Effects of Analgesics After Surgery: A Pediatric Patient Perspective***	To better understand the AEs of analgesics from the perspective of adolescent patients with idiopathic scoliosis after spinal surgery.	Pediatric	Spine, idiopathic scoliosis	Canada	Qualitative (descriptive)	Not reported	Patients	7	Interviews (open, unstructured, in-depth, semi-structured, open ended)	Content analysis	Neergaard et al (2009)	Not reported	Overall, participants most frequently reported gastrointestinal and cognitive AEs, with constipation being the most persistent and bothersome. The pediatric participants used a combination of 3 strategies to mitigate analgesic AEs, namely pharmacologic, nonpharmacologic, and reduction of analgesic intake. Participants demonstrated a lack of understanding of AEs and involvement in their own care.
MacCulloch, Donaldson, Nicholas, et al.	Spinal surgery	2009	Canada	Not reported	The Hospital for Sick Children	All HIC	Scoliosis	***Towards an understanding of the information and support needs of surgical adolescent idiopathic scoliosis patients: A qualitative analysis***	To identify health-specific needs for online information and support for patients with adolescent idiopathic scoliosis who have had or anticipate having spinal surgery.	Adolescents	Spine, scoliosis	Canada	Not stated	Not reported	Patients	11	Mixed: Focus groups or individual interviews	Content analysis	Not reported	Not reported	Two focus groups consisting of 8 adolescents (1 male, 7 female) and subsequent individual interviews with 3 adolescents (1 male, 2 female) yielded a range of participant concerns, in order of prominence: (1) recovery at home; (2) recovery in hospital; (3) post-surgical appearance; (4) emotional impact of surgery and coping; (5) intrusion of surgery and recovery of daily activities; (6) impact of surgery on school, peer relationships and other social interactions; (7) decision-making about surgery; (8) being in the operating room and; (9) future worries.
Manna, Mortenson, Kardeh, et al.	Spinal surgery	2022	Canada	Not reported	Faculty of Medicine	All HIC	PM & R : the journal of injury, function, and rehabilitation	***Patient perspectives and self-rated knowledge of nerve transfer surgery for restoring upper limb function in spinal cord injury***	To explore the perspectives of patients with chronic SCI (>2 years from injury) on nerve transfer surgery, and to determine if an educational intervention improved participants' perceived knowledge levels about the procedure.	Adult	Spine, SCI	Canada	Mixed methods (Embedded design)	Not reported	Patients	10	Interviews (open, unstructured, in-depth, semi-structured, open ended)	Content analysis	Hsieh & Shannon (2005)	COREQ AND GRAMMS	Regaining upper limb function was a priority for all participants. Although most participants had heard of nerve transfer, none were offered it at the time of their SCI, and only two stated that they had any peers who had undergone the procedure. The educational module significantly increased self-rated scores on understanding of nerve transfer ($p < .05$). Although all participants were open to nerve transfer after the educational module, they described weighing different factors, including (1) potential for loss versus gain of function, (2) inadequate knowledge about nerve transfer, (3) recovery time, and (4) determining their eligibility for the surgery.

Rehman, Syed, Wiercioch, et al.	Spinal surgery	2019	Canada	MD/Physician	Dept of Health Research Methods, Evidence and Impact	All HIC	Spine	***Discrepancies between patient and surgeon expectations of surgery for sciatica: A challenge for informed decision making?***	Compare the perceptions of patients and surgeons regarding the risks and benefits of lumbar decompressive surgery for sciatica following a consultation meeting.	Adult	Spine; Lumbar decompression	Canada	Qualitative (descriptive)	Not reported	Patients AND HCPs (inc. surgeons)	18	Interviews (open, unstructured, in-depth, semi-structured, open ended)	Thematic: content analysis	Not reported	Not reported	Our analysis revealed that most patients were satisfied with the consultation despite limited understanding of lumbar decompressive surgery. We found discrepancies between patients' preoperative expectations and understanding of information provided by surgeons and what surgeons believed they had conveyed. Surgeons and patients disagreed on how much information is needed about postsurgical activity modifications and long-term outcomes to make a decision about whether or not to undergo surgery, with patients desiring more information. As a result, for most patients, the decision-making process extended beyond the information provided by surgeons and incorporated information from family members, friends, family doctors, and the internet.
Samuel, Bernstein, Alotaibi, et al.	Spinal surgery	2017	Canada	MD/Physician	Division of Neurosurgery, Toronto western hospital; Department of Surgery, Faculty of Medicine	All HIC	Neuromodulation	***Patient Perspectives Regarding Ethics of Spinal Column Stimulators in the Surgical Management of Persistent Postoperative Neuropathic Pain***	To better understand perspectives of patients with persistent postoperative neuropathic pain (PPNP) and assess perceptions of the ethical issues surrounding their structural spinal surgeon also performing spinal cord stimulation (SCS).	Adult	Spine; Persistent Post operative neuropathic pain	Canada	Qualitative	Not reported	Patients AND Family members, carers, signifcant others	20	Interviews (open, unstructured, in-depth, semi-structured, open ended)	Grounded theory analysis/constant comparison/open, axial and/or selective coding (modified thematic analysis)	Not reported	Not reported	The range of the duration of participants' preoperative symptoms varied from one month to more than 20 years, and was primarily back dominant (13/20). The median time since patients most recently underwent spinal surgery was three years. The majority of patients (15/20) do not view their current condition of PPNP as a failure of their initial spine surgeon. The most commonly reported reason for this is that patients trusted their physician and clear communication between the physician and the patient, prior to their surgery, ensured an understanding of the goals of the procedure. Nearly unanimously (19/20), patients did not perceive an ethical problem with a surgeon performing a structurally corrective spinal surgery and subsequently also implanting a SCS device if the same patient that develops medically refractory PPNP.
Zahrai, Bhanot, Mei, et al.	Spinal surgery	2020	Canada	MD/Physician	Division of Orthopedic Surgery	All HIC	Canadian Journal of Surgery	***Surgeon clinical practice variation and patient preferences during the informed consent discussion: A mixed-methods analysis in lumbar spine surgery***	To determine practice variations among spine surgeons in regard to the disclosure of potential adverse events during informed consent discussions for lumbar microdiscectomy and to determine which topics patients perceived to be valuable in the consent discussion.	N/A	Spine; lumbar microdiscectomy	Canada	Mixed methods	Not reported	NSx	24	Focus groups	Grounded theory analysis/constant comparison/open, axial and/or selective coding (Thematic, content analysis)	Krippendorff (2004)	Not reported	The number of potential adverse events not routinely discussed was greater among orthopedic surgeons than among neurosurgeons (relative risk 1.83; 95% confidence interval 1.22–2.73; p = 0.003). Three preoperative patients, 7 postoperative patients, 6 attending spine surgeons, 3 spine fellows and 5 orthopedic residents participated in the semi-structured interviews. The interviews identified gaps in information provided to patients, particularly on topics relating to postoperative care such as expected recovery time, activity restrictions and need for a caregiver.
Young, Siden and Tredwell	Spinal surgery	2007	Canada	Not reported	School of Nursing	All HIC	Journal of Telemedicine and Telecare	***Post-surgical telehealth support for children and family care-givers***	To evaluate the relative effectiveness of telephone and videophone follow-up for children and families after a child's scoliosis surgery	Pediatric	Spine; Scoliosis	Canada	Grounded theory	Not reported	Patients AND Family members, carers, signifcant others	86	Other: Follow-up call and interview (type unclear)	Grounded theory analysis/constant comparison/open and axial/selective coding	Charmaz (2003); Glaser (1978); Strauss & Corbin (1998)	Not reported	The results showed that videophone and telephone use provided care continuity for patients and their families following a child's back surgery. The relative effect of the videophone and telephone technology depended on the fit between the characteristics of the patients and families and the capacities of the technology. When implementing telehealth for follow-up care, a participatory process is recommended to ensure a fit between user characteristics and technology.
Yu, C. and Luo, C. M. and Song, C. P.	Spinal surgery	2023	China	Unclear/not stated	Department of Urology	All LMIC	Bmc Musculoskeletal Disorders	***Symptoms and coping of patients with dysphagia after anterior cervical spine surgery: a qualitative study***	To explore the subjective symptoms, psychological characteristics and coping strategies of patients with dysphagia after anterior cervical spine surgery, so as to provide the basis for formulating strategies to help patients with dysphagia solve clinical practice problems and to improve their quality of life after surgery.	Adult	Dysphagia after anterior cervical spine surgery	China	Interpretive phenomenology	Not explicitly stated	Patients	22	Interviews (semi-structured, open, in depth)	Phenomenological analysis (Colaizzi 7-step analysis)	Deng (2020)	Not reported	A total of 22 (10 females and 12 males) patients, with years old ranging between 33 and 78 years were interviewed. When analysing the data, the following 3 categories were extracted from the participant interviews: "Subjective symptoms, Coping style and impact on social life". The 3 categories consist of 10 sub-categories.
Andersen, Birkelund, Andersen, et al.	Spinal surgery	2019	Denmark	Not reported	Spine Surgery and Research, Center for Shared Decision, Institute of Regional Health Research	All HIC	Spine	***Factors affecting patient decision-making on surgery for lumbar disc herniation***	to explore from a patient perspective what affects patients' decisions on whether or not to have surgery for symptomatic LDH.	Adult	Spine; Lumbar disc herniation	Denmark	Phenomenology (Hermeneutic)	Shared Decision Making	Patients	14	Interviews (open, unstructured, in-depth, semi-structured, open ended)	Other: meaning condensation method	Kvale & Brinkmann (2015)	COREQ	Four main themes that could directly or indirectly influence the patients' decision-making process were identified: A) Patient information: patients' conceptions about treatment were not always based on sufficient information; B) Accelerated workflows: some patients needed time to process the information given, which may be limited due to accelerated workflows; C) Power imbalance: patients can be reluctant to challenge the system, as they do not want to offend, which can be seen as a power imbalance between clinicians and patients; and D) Personal past experience: experience, about treatment options from, i.e., close relatives, can impact patients thoughts about possible treatments.
Strom, Hobye, Laursen, et al.	Spinal surgery	2019	Denmark	RN/Nurse	Elective Surgery Center	All HIC	Journal of Medical Internet Research	***Lumbar spine fusion patients' use of an internet support group: Mixed methods study***	To describe the characteristics of users of an ISG and thematically explore the content of ISG interactions in Danish patients undergoing instrumented LSF because of degenerative spine disorders.	Adult	Spine; Lumbar spinal fusion secondary to degenerative spinal disorders	Denmark	Mixed methods	Not reported	Patients	48	Online support group message board	Content analysis (Inductive)	Schreier (2012)	Not reported	7 thematic categories: Social recognition; Experience of pain or use of pain medication; Experience of physical activity or rehabilitation; Expression of psychosocial wellbeing; Expression of everyday activities; Advising on and exploring the internet support group; Employment
Debono, B. and Lonjon, G. and Guillain, A. and Moncany, A. H. and Hamel, O. and Challier, V. and Diebo, B. and Spine Res Community, S. R. C.	Spinal surgery	2024	France	Unclear/not stated	Paris-Versailles Spine Center; Ramsay Sante-Hopital	All HIC	Spine Journal	***Spine surgeons facing second opinions: a qualitative study***	to explore surgeons' perceptions on the impact of second opinions on their interactions with their patients	N/A	Spine	FR	Grounded theory	Non stated	Mixed: NSx and OrthoSx	24	Interviews (semi-structured, open, in depth)	Thematic analysis (inductive)	Non stated	COREQ	Data analysis identified five overarching themes based on recurring elements in the interviews: (1) analysis of the patient's motivations for seeking a second opinion; (2) impaired trust and disloyalty; (3) ego, authority, and surgeon image; (4) management of a consultation recourse (measurement and ethics); and (5) the second opinion as an avoidance strategy.

Guillain, Antoine, Moncany, et al.	Spinal surgery	2020	France	Not reported	Sorbonne Study Group on Methods of Sociological Analysis of the Sorbonne (GEMASS), Sorbonne University and SiRIC CURAMUS (Integrated Cancer Research Site)	All HIC	Acta neurochirurgica	***Spine neurosurgeons facing the judicialization of their profession: disenchantment and alteration of daily practice-a qualitative study***	To identify the impact of the judicialization of medicine in the practice of spine neurosurgeons.	N/A	Spine	France	Grounded theory	Not reported	NSx		23	Interviews (open, in-depth, semi-structured, open ended)	Grounded theory analysis/constant comparison/open, axial and/or selective coding	Starks & Brown Trinidad (2007)	COREQ	Data analysis identified five superordinate themes that were based on items that recurred in interviews: (1) private practice of spinal surgery (high-risk surgery based on frequent functional symptoms, in an unfavorable medicolegal context); (2) societal transformation of the doctor-patient relationship (new societal demands, impact of the internet and social network); (3) judicialization of spine surgery (surgeons' feelings about the frequency and motivation of the complaints they receive, and their own management of them); (4) coping strategies (identification and solutions for "at risk" situations and patients); and (5) professional disenchantment (impact of these events on surgeons' daily practice and career planning).
Masi, Couraud, Daste, et al.	Spinal surgery	2021	France	Not reported	Service de Rééducation et de Réadaptation de l'Appareil Locomoteur et des Pathologies du Rachis; Unit of Physical Medicine and Rehabilitation	All HIC	European Journal of Physical and Rehabilitation Medicine	***Development of a new patient-reported outcome measure assessing activities and participation in people with lumbar spinal stenosis: The cochin spinal stenosis 19-item questionnaire***	To develop a new patient-reported outcome measure assessing activities and participation in people with lumbar spinal stenosis (LSS)	Adult	Spine; lumbar spinal stenosis	France	Mixed methods (Surveys; Qualitative)	Not reported	Patients		20	Mixed: Interviews (open, unstructured, in-depth, semi-structured, open ended) AND questionnaires	Thematic: content analysis	Not stated	COREQ	Concepts collected from patients generated a 48-item provisional questionnaire. Overall, 63/200 (31.5%) patients completed the pro-visional questionnaire. Item reduction resulted in a 19-item questionnaire, the Cochin Spinal Stenosis 19-item (CSS-19) questionnaire. Principal component analysis extracted 3 factors. In confirmatory analysis, factor 1 influenced all items. We found convergent validity with low back pain, LSS-specific disability and divergent validity with mental health-related quality of life. Cronbach α coefficient (95% CI) was 0.96 (0.94; 0.97). ICC was 0.90 (0.70; 0.97). Bland and Altman analysis found no systematic trend for test-retest.
Koch, Pfandler, Stefan, et al.	Spinal surgery	2019	Germany	Not reported	Institute and Outpatient Clinic for Occupational, Social, and Environmental Medicine	All HIC	Surgical Innovation	***Say, What Is on Your Mind? Surgeons' Evaluations of Realism and Usability of a Virtual Reality Vertebroplasty Simulator***	To develop and apply a classification system of surgeon-reported experience during operation of a VR vertebroplasty simulator.	N/A	Spine, vertebroplasty	Germany	Mixed methods (Think aloud protocols based on video recordings, poststudy questionnaires, expert evaluations, and digital performance data)	Not reported	HCPs (inc. NSx)		13	Mixed - Video data, questionnaires, Surgeons' Intraoperative Performance (Simulator-Based), Surgeon's Intraoperative Performance (Expert-Based)	Content analysis	Elo (2008)	Not reported	Overall, 244 comments on realism and usability of the vertebroplasty simulator were collected. This included positive and negative remarks, questions, and specific suggestions for improvement. Further findings included surgeons' approval of the realism and usability of the simulator and the observation that the haptic feedback of the VR patient's anatomy requires further improvement. Surgeon- reported evaluations were not associated with performance decrements.
Lam, A. K. H. and Fung, O. H. Y. and Kwan, C. and Cheung, J. P. Y. and Luk, K. D. K. and Chiu, A. Y. Y. and Descarreaux, M. and Szeto, G. P. Y. and Wong, A. Y. L.	Spinal surgery	2022	Hong Kong	Unclear/not stated	Department of Rehabilitation Sciences, Department of Orthopaedics and Traumatology	All HIC	Archives of Rehabilitation Research and Clinical Translation	***The Concerns and Experiences of Patients With Lumbar Spinal Stenosis Regarding Prehabilitation and Recovery After Spine Surgery: A Qualitative Study***	To improve our understanding of patients' perspectives regarding: (1) the decision-making and prehabilitation before lumbar spinal stenosis (LSS) surgery and (2) their postoperative experiences.	Adult	Lumbar spinal stenosis	Hongkong	Qualitative	Non stated	Patients		25	Interviews (semi-structured, open, in depth)	Thematic analysis	Kiger and Varpio (2020)	Not reported	Thematic analysis was conducted to identify 4 themes inductively: (1) sources of infor- mation about LSS surgery; (2) factors affecting the surgical decision-making; (3) attitudes toward prehabilitation; and (4) postoperative recovery.
Li, L. L. C. and Wong, A. Y. L. and Kawchuk, G. N.	Spinal surgery	2022	China	Unclear/not stated	Department of Rehabilitation Sciences	Mixed HIC/LMIC	Chiropractic & Manual Therapies	***An exploratory study to understand how people use Twitter to share experiences or information about spinal stenosis***	to identify tweets that are related to spinal stenosis on Twitter, and to categorize them into common themes.	Unclear	Spinal stenosis	International	Mixed methods,	Not explicitly stated	Mixed: Patients; lay users; HCPs; researchers	362 tweets		Other please state	Thematic analysis	Kiger and Varpio (2020)	Not reported	Of 510 identified tweets, 362 tweets met the selection criteria. Five themes were identified: (1) compromised physical, psychological, and social wellbeing (n = 173); (2) diverse treatment options (n = 69); (3) coping strategies (n = 30); (4) dissemination of scientific information (n = 86); and (5) health policy (n = 4). Most of the tweets revealed negative impacts of spinal stenosis on patients' physical and psychosocial wellbeing. People with spinal stenosis shared their experiences and sought helps from others, while some people used Twitter to disseminate relevant information and research findings.
Paulson, A. E. and Martus, J. E. and Mencio, G. A. and Louer, C. R.	Spinal surgery	2024	USA	Unclear/not stated	Department of Orthopedic Surgery	All HIC	Spine Deformity	***Information exchange on adolescent scoliosis discussion forums among patients and caregivers: a thematic analysis***	to (1) review posts from scoliosis discussion forums to establish common themes related to the care experience of patients with scoliosis and (2) understand how common themes vary among pediatric and adult patients, as well as caregivers.	Adolescents	Scoliosis	International	Grounded theory	Not explicitly stated	Patients AND Families/carers/ Significant other		911	Online discussion boards	Grounded theory analysis	Strauss and Corbin (1990)	Not reported	Analysis of 911 posts revealed five central themes. The two most common themes among patients ≥ 18 and caregivers involve seeking out emotional support and information about surgical treatment. Patients < 18 frequently sought out emotional support but were also largely interested in information about bracing. The most prevalent theme among all contributors involved seeking out emotional support. There was very little medical misinformation found within posts.
Christine, Marco, Louis-Rachid, et al.	Spinal surgery	2020	Switzerland	Not reported	Division of General Medical Rehabilitation/Division of Clinical Pharmacology and Toxicology	All HIC	European spine journal	***Clinicians' views about the experience of disability due to low back pain. Results from a focus group study***	To examine how clinicians involved in the care of patients with lower back pain describe and define disability, its associated changes, and rehabilitation.	Adult	Spine; Low back pain	International (Europe, Middle East, North Africa)	Qualitative	Not reported	HCPs (inc. NSx)		26	Focus groups	Other: Group discussion, consensus, and mind mapping software used in parallel to aggregate responses.	Not reported	Not reported	Disability was viewed as a major source of physical limitations, difficulties in performing daily activities, associated with emotional distress, and raising legitimacy issues. Changes in roles engaged the social component. Considered from the patients' perspective, negative emotions and social issues were emphasized, along with the patients' resources. For rehabilitation, the participants emphasized patient-centered care, teamwork, and objectives for care.
Ghorbani, F. and Kamali, M. and Ranjbar, H. and Kamyab, M. and Razavi, H. and Babaee, T. and Vitiello, R.	Spinal surgery	2024	Iran	Other: PhD candidate	Department of Orthotics and Prosthetics, Rehabilitation Research Center, School of Rehabilitation Sciences,	Mixed HIC/LMIC	Plos One	***Brace compliance process in adolescents with spinal deformities: A qualitative study***	This study aimed to understand the brace compliance process for adolescents with spinal deformities through a qualitative approach.	Adolescents	spinal deformities	Iran	Qualitative descriptive	interpretive framework	Patients AND Families/carers/ Significant other AND clinicians		74	Interviews (semi-structured, open, in depth)	Content analysis	Graneheim and Lundman	COREQ	Based on the analysis of the current qualitative research, adolescents with spinal deformities experience extensive challenges in the treatment process, which can affect the results and brace intervention efficacy. The current research findings showed that every adolescent goes through similar but unique conditions during the treatment. The importance of considering each adolescent's specific conditions and characteristics and providing functional solutions and support was understood to help them navigate critical situations more quickly and achieve effective treatment outcomes.

Parvar, S. Y. and Mojgani, P. and Lankarani, K. B. and Poursaeed, F. and Jahromi, L. S. M. and Mishra, V. and Abbasi, A. and Shahabi, S.	Spinal surgery	2024	Iran	Medical student	Health Policy Research Center, Institute of Health	Mixed HIC/LMIC	Bmc Public Health	***Barriers and facilitators to reducing low-value care for the management of low back pain in Iran: a qualitative multi-professional study***	to explore the perceptions of service providers regarding the facilitators and barriers to reducing LVC in the management of LBP in Iran.	Unclear	Low back pain	Iran	Qualitative descriptive	Not explicitly stated	HCPs (Inc NSx)	20	Interviews (semi-structured, open, in depth)	Content analysis	Not stated	SRQR	Thirty-nine sub-themes, with 183 citations, were identified as barriers, and 31 sub-themes, with 120 citations, were defined as facilitators. Facilitators and barriers to reducing LVC for LBP, according to the interviewees, were categorized into five themes, including: (1) individual provider characteristics; (2) individual patient characteristics; (3) social context; (4) organizational context; and (5) economic and political context. The ten most commonly cited barriers included unrealistic tariffs, provider-induced demand, patient distrust, insufficient time allocation, a lack of insurance coverage, a lack of a comprehensive referral system, a lack of teamwork, cultural challenges, a lack of awareness, and defensive medicine. Barriers such as adherence to clinical guidelines, improving the referral system, improving the cultural status of patients, and facilitators such as strengthening teamwork, developing an appropriate provider-patient relationship, improving the cultural status of the public, motivating the patients, considering an individualized approach, establishing a desirable payment mechanism, and raising the medical tariffs were most repeatedly stated by participants.
Babamohamadi, Negarandeh and Dehghan-Nayeri	Spinal surgery	2011	Iran	Not reported	Department of Nursing, Faculty of Nursing and Midwifery	All LMIC	Spinal Cord	***Coping strategies used by people with spinal cord injury: A qualitative study***	To explore the approach used by Iranians chronically affected by SCI to cope with their chronic illness complications in its cultural context using a qualitative approach and to provide some culturally based foundations for their care.	Adult	Spine; SCI	Iran	Qualitative (part of a grounded theory PhD)	Not reported	Patients	18	Interviews (open, in-depth, semi-structured, open ended)	Content analysis AND constant comparative method	Sandelowski 2000	Not reported	During the data analysis, three coping strategies, including seeking help from religious beliefs (understanding the disease as a divine fate and as a spiritual combat), hope and making efforts towards independence/self-care appeared.
Khazaeipour, Abouie, Zarei, et al.	Spinal surgery	2018	Iran	MD/Physician	Brain and Spinal Cord Injury Research Center, Neuroscience Institute	Mixed HIC/LMIC	Neurosciences	***Personal, family and societal educational needs assessment of individuals with spinal cord injury in Iran***	To explore individuals' perception of the personal, family and societal educational needs following a spinal cord injury)SCI(.	Adult	Spine, SCI	Iran	Survey (open & Closed)	Not reported	Patients	61	Questionnaire: Open & closed questions	Thematic analysis	Boyatzis (1998); Auerbach & Silverstein (2003); Saldana (2014)	Not reported	Following a thematic analysis of the patient's perceived educational needs, 3 themes and 14 subthemes were identified. The 3 themes included personal, family, and societal educational perceived needs. Within personal educational needs, there were 7 subthemes which included personal independence and transportation, financial independence, life skills modification, knowledge about SCI, prevention of SCI complications, relationships and sexual function, and psychological adjustments. Among family educational needs, the 3 subthemes were caregiver skills and communication, first aid and emergency skills, and emotional and psychological support. For societal educational needs, the 4 subthemes described were social integration, interpersonal communication skills, SCI awareness and injury prevention, sympathize while avoiding pity.
Khazaeipour, Nikbakht-Nasrabadi, Mohammadi, et al.	Spinal surgery	2018	Iran	MD/Physician	Brain and Spinal Cord Injury Research Center, Neuroscience Institute	All LMIC	Spinal Cord	***The childbearing experience of women with spinal cord injury in Iran: a phenomenological study***	This study investigated the experience of pregnancy and childbirth in women with spinal cord injury.	Adult	Spine, Pregnant women with SCI	Iran	IPA	Not reported	Patients	8	Interviews (open, unstructured, in-depth, semi-structured, open ended)	IPA	Van Manen (2016)	Not reported	Five main themes have emerged from data analysis: "revivification", "fear and concern of motherhood with SCI", "flawed health care system", "maternal experience under a supportive umbrella", and "strengthening spirituality and religious belief".
Maasoumi, Zarei, Emami Razavi, et al.	Spinal surgery	2017	Iran	Not reported	Brain and Spinal Cord Injury Research Center.	All LMIC	Trauma Monthly	***How Iranian women with spinal cord injury understand sexuality***	This study focused on the sexual understanding in a sample of Iranian women with SCI.	Adult	Spine	Iran	Not stated	Not reported	Patients	24	Interviews (open, unstructured, in-depth, semi-structured, open ended)	Thematic analysis	Clarke & Braun (2013)	Not reported	According to participation viewpoints, the following three main themes were explored: the dilemma that lead to limited sexual activity, seeking positive sexual adjustment, and the lack of client-based sexuality education in the rehabilitation process.
Merghati-Khoei, Maasoumi, Zarei, et al.	Spinal surgery	2017	Iran	Not reported	Brain and Spinal Cord Injury Research Center; Iranian National Center for Addiction Studies	Mixed HIC/LMIC	Topics in Spinal Cord Injury Rehabilitation	***How do Iranian people with spinal cord injury understand marriage?***	To explore the understandings of Iranian adults with SCI about marriage	Adult	Spinal Cord Injury	Iran	Qualitative	Not reported	Patients	53	Interviews (open, unstructured, in-depth, semi-structured, open ended)	Thematic analysis	Braun & Clarke (2013)	Not reported	"Marriage" was thematized in outer and inner scenarios. The outer scenario was explored in terms of physical disability identified as a seminal determinant in successful marriage. "Attractiveness," "able body for breadwinning," "sexually active," and "reproduction" were dominant concepts extracted from the participants' narratives. The participants' inner scenarios revealed that marriage would be welcomed if a potential partner accepted them as a "whole person" regardless of their SCI condition.
Motyer, Kiely and Fitzgerald	Spinal surgery	2022	Ireland	PhD Psychology candidate	School of Psychology	All HIC	Journal of Pediatric Psychology	***Adolescents' Experiences of Idiopathic Scoliosis in the Presurgical Period: A Qualitative Study***	To explore the psychosocial experiences of adolescents with idiopathic scoliosis during the presurgical stage of treatment	Adolescents	Spine; Adolescent idiopathic scoliosis	Ireland	Qualitative	Not reported	Patients	14	Interviews (open, unstructured, in-depth, semi-structured, open ended)	Reflexive thematic analysis	Braun & Clarke (2006, 2019)	O'Brien et al (2014)	Four key themes were generated from the analysis. "Proceeding with Caution" described adolescents' adaptation to the physical impact of their AIS, while "Am I Different?" encompassed adolescents' perceptions of their changing appearance and visibility of their condition. "An Emotional Journey" captured the rollercoaster of emotions from shock at diagnosis to the daunting realization of the severity of their condition, while knowing others with AIS could ease the emotional burden. Finally, adolescents' concerns and expectations about their prospective surgery were captured by the theme "No Pain, No Gain", whereby they were often keen to put surgery behind them.
Ferrari, S. and Cedraschi, C. and Mapelli, N. and Baram, A. and Costa, F. and Gatti, R. and Fornari, M.	Spinal surgery	2023	Italy	Physiotherapist	Department of Biomedical Sciences	All HIC	Disability and Rehabilitation	***Thoughts and concerns of patients at hospital discharge after lumbar spine surgery. A qualitative study***	To investigate the beliefs, expectations, thoughts and concerns of patients undergoing spinal surgery for lumbar degenerative pathology at the time of discharge from the hospital, before returning home.	Adult	Low back pain	Italy	Qualitative	Non stated	Patients	28	Interviews (semi-structured, open, in depth)	Content thematic analysis	Ritchie and Spencer (1994); Bradley, Curry, Devers (2007); Braun and Clarke (2006); Clarke and Braun (2020)	COREQ	The patients were satisfied with the surgeons' preoperative explanations and description of expected prognosis. However, they were disappointed with the lack of information at hospital discharge, in particular regarding practical and behavioral recommendations. The patients expressed clear concerns about being left alone to deal with possible complications or difficulties they may encounter when returning home.
Henssen, Scheepers, Kurt, et al.	Spinal surgery	2018	The Netherlands	Not reported	Anatomy	All HIC	Pain Practice	***Patients' Expectations on Spinal Cord Stimulation for Failed Back Surgery Syndrome: A Qualitative Exploration***	To understand the expectations of patients receiving spinal cord stimulation	Adult	Spine	Netherlands	Qualitative	Not reported	Patients	13	Mixed: Interviews (open, unstructured, in-depth, semi-structured, open ended) AND questionnaires	Content analysis: Inductive, iterative	Not reported	Not reported	Analysis of the interviews revealed that the expected outcomes of SCS could be categorized into 13 categories which could be grouped into grouped into 6 general themes: (1) physical well-being, (2) social well-being, (3) material well-being, (4) emotional well-being, (5) development and activity, and (6) constraints of the procedure of SCS.

Hofstede, Marang-van de Mheen, Wentink, et al.	Spinal surgery	2013	The Netherlands	Not reported	Department of Medical Decision Making	All HIC	Implementation Science	***Barriers and facilitators to implement shared decision making in multidisciplinary sciatica care: A qualitative study***	To identify barriers and facilitators perceived by patients and professionals for SDM implementation in multidisciplinary sciatica care.	Adult	Nerve	Netherlands	Qualitative	Grol and Wensing (2004) framework of barriers and facilitators	Patients AND HCPs (inc. NSx)	62	Mixed: Interviews (open, unstructured, in-depth, semi-structured, open ended) AND focus groups	Content analysis	Hsieh & Shannon (2005)	Not reported	Professionals reported 53 barriers and 5 facilitators, and patients 35 barriers and 18 facilitators for SDM in sciatica care. Professionals perceived most barriers at the level of the organizational context, and facilitators at the level of the individual professional. Patients reported most barriers and facilitators at the level of the individual professional. Several barriers and facilitators correspond with barriers and facilitators found in the literature (e.g., lack of time, motivation) but also new barriers and facilitators were identified. Many of these new barriers mentioned by both professionals and patients were related to the multidisciplinary setting, such as lack of visibility, lack of trust in expertise of other disciplines, and lack of communication between disciplines.
Malomo, Aminu, Adeolu, et al.	Spinal surgery	2019	Nigeria	Not reported	Department of Sociology	All LMIC	West African journal of medicine	***Assessment of the Holistic Model of Neurosurgical Patient Care at the University College Hospital (UCH), Ibadan***	To assess the effectiveness of the holistic model of care and identified the challenges facing this model of healthcare delivery.	Unclear	Spine	Nigeria	Not stated	Not reported	HCPs (inc. NSx)	18	Interviews (open, unstructured, in-depth, semi-structured, open ended)	Not clearly stated	Not reported	Not reported	The study found that patients and relations have immensely benefitted from the model of care through psychosocial support. The major challenges facing holistic ward round (HWR) were logistic, timing and common problems found in the Nigerian healthcare system.
Navarrete-ZampaÃ±a, M. D. and FernÃ¡ndez-Baillo, N. and Pizones, J. and SÃ¡nchez-MÃ¡rquez, J. M. and SellÃ¡n-Soto, M. C.	Spinal surgery	2023	Spain	Unclear/not stated	Pediatría, cirugía pediátrica; Red ENSI-Espana	All HIC	Enfermeria Clinica	***The post-surgical transition in adolescents who have idiopathic scoliosis. A qualitative study***	To know the process experienced by adolescents suffering from idiopathic scoliosis when undergoing surgery to correct the physical deformity.	Adolescents	Idiopathic scoliosis	Spain	Qualitative	Symbolic interactionism (Taylor and Bogdan (1987))	Patients	22	Interviews (semi-structured, open, in depth)	Analysis in Progress method	Taylor and Bogdan (1987)	Not reported	Patients with adolescent idiopathic scoliosis present with a complex simultaneous health/illness and developmental transition. The main inhibitory conditions of the transition are the meanings about: their identity, social, beliefs about surgery, ignorance about the pathology, the surgical process, and their recovery. As facilitating conditions, we find: a positive attitude towards physical, aesthetic, and social change, socioeconomic level, and family support.
Pérez-Grueso, F. J. S. and Moreno-Manzanaro, L. and Pizones, J.	Spinal surgery	2024	Spain	Neurosurgeon	Spine Unit, Department of Orthopaedic Surgery	All HIC	Spine Deformity	***The reunion with my patients: their journey and experience 30 years after their intervention for adolescent idiopathic scoliosis via CD instrumentation***	to collect the experience and current attitude of those patients, now adults, operated on for adolescent idiopathic scoliosis (AIS) more than 25 years ago with CD instrumentation (CDI).	Adolescents	Idiopathic scoliosis	Spain	Qualitative	Interpretive	Patients	100	Interviews (semi-structured, open, in depth)	Interpretive phenomenological analysis	Smith (2011)	Not reported	We contacted 103 patients, 100 agreed to participate. Mean age was 47.5 ± 3.3, mean follow-up was 30.9 ± 2.7 years. Three fundamental concerns stood out: discomfort with self-image; low back pain with daily activities; and lack of spinal flexibility. 50% were engaged in continuous physical exercise, and only some referred limitations with load-bearing work. Patients commonly described negative memories of the conservative treatment, but positive memories of the surgical process. In general, there was a good adaptation to social life (occupation, social and family relationships). Two-thirds were married, and 65 women had offspring. A frequent concern was the excess of radiographs over the years, and three developed breast cancer.
Bonilla Carrasco and Solano Ruiz	Spinal surgery	2016	Spain	RN/Nurse	Hospital Traumatología y Rehabilitación	All HIC	Texto e Contexto Enfermagem	***Idiopathic adolescent scoliosis: Living with a physical deformity***	To explain the experience of having a body deformity diagnosed as idiopathic adolescent scoliosis.	Adolescents	Spine	Spain	Phenomenology (Hermeneutic)	Hermeneutical orientation	Patients	12	Interviews (open, unstructured, in-depth, semi-structured, open ended)	Other: categories established, taking into account the objectives of the initial study.	Not reported	Not reported	The youth defined their scoliosis based on how they perceived their deformity. They spoke of pain and deformity as characteristic symptoms of suffering, and explained how this symptom affected their social relationships. Their deformity was associated with words such as "horrible", "shame", "complex" and "problem." It is concluded that the symptom most referred is pain and the biggest concern of the of the youth was their body aesthetic and feelings associated with it. They attempt to solve this problem by adapting the way they dress and through surgery. Surgery can resolve the body deformity but not self-perception of their body image.
Cano, Gestoso, Kovacs, et al.	Spinal surgery	2014	Spain	Not reported	Unidad de Bioestadı ́stica Clı ́nica	All HIC	Disability and Rehabilitation: An International, Multidisciplinary Journal	***The perceptions of people with low back pain treated in the Spanish National Health, and their experience while undergoing a new evidence-based treatment A focus group study***	To explore the perceptions of people with low back pain (LBP) treated within the Spanish National Health Service, and their experience while undergoing a new evidence-based treatment ("neuroreflexotherapy").	Adult	Spine; Low back pain	Spain	Qualitative	Critical realist paradigm	Patients	32	Focus groups	Content analysis (Inductive)	Duggleby (2005)	Not reported	Results: Subacute and chronic LBP curtails daily activities, reduces quality of life (QoL) and self-esteem, and is experienced as a stigma. Patients want to be treated with respect and empathy by clinicians who refrain from judging them. New treatments trigger hope, but also fear and mistrust. Most patients experiencing a clinically relevant improvement resume daily activities, and report improvement in QoL, self-esteem and emotional wellbeing.
Navarrete-Zampaña, Sellán-Soto and Díaz-Martínez	Spinal surgery	2019	Spain	Not reported	Enfermera pediátrica	All HIC	Enfermeria Clinica	***Painful experience in adolescents undergoing surgical correction of scoliosis***	To learn about the post-surgical pain experience in adolescents undergoing surgical correction of Adolescent Idiopathic Scoliosis (AIS).	Adolescents	Spine; Adolescent Idiopathic Scoliosis	Spain	Phenomenology (Interpretive)	Orem's theoretical framework	Patients	7	Interviews (open, unstructured, in-depth, semi-structured, open ended)	Content analysis (interpretation and comparison)	Not reported	Not reported	The categories established were: influential factors, values and ideas, coping mechanisms and improvement areas. Painful experience is a determining factor during recovery, influenced by the individuals themselves, as well as by the support received, and the environment. Their main values and ideas about the process are based on pre-surgical information, which they consider insufficient. In order to cope, they use of distraction, relaxation and drug consumption. According to the informants, increasing the information they are given about the process, facilitating visits and incorporating complementary techniques would improve pain control.
Jamieson, F. and Rasmussen-Barr, E.	Spinal surgery	2024	Sweden	Other: Physiotherapist	Karolinska Institutet, Department of Neurobiology, Care Sciences and Society, Division of Physiotherapy	All HIC	Musculoskeletal Science and Practice	***How do information and physiotherapy affect health-related quality of life among patients with spinal stenosis undergoing decompression surgery: A qualitative study***	We aimed to investigate the experiences of patients with LSS undergoing decompression surgery regarding their pre- and post-surgery perceptions of HRQOL and the pre-and post-operative information and physiotherapy.	Adult	Lumbar spinal stenosis	Sweden	Qualitative	Non stated	Patients	12	Interviews (semi-structured, open, in depth)	Content analysis	Graneheim and Lundman (2004)	COREQ	Four distinct categories with nine associated subcategories were identified: Patients' feelings of safety and empowerment are enhanced by healthcare professionals; Divided perceptions of information and physiotherapy in a group context; Health-related quality of life is associated with patients' perceived physical capacity; Patients' optimism and concerns influence health-related quality of life.

Lindbäck, Y. and Carlfjord, S.	Spinal surgery	2024	Sweden	Physiotherapist	Unit of Physiotherapy	All HIC	Musculoskeletal Science and Practice	***Experiences from pre-surgery physiotherapy and thoughts about future exercise among patients with disc herniation or spinal stenosis: A qualitative study***	To describe patients' pre- and post-surgery experiences after a pre-surgery physiotherapy intervention, and their thoughts about future exercise and self-management.	Adult	Disc herniation or spinal stenosis	Sweden	Qualitative	Constructivist	Patients	18	Interviews (semi-structured, open, in depth)	Content analysis	Patton (2014), Hsieh and Shannon (2005)	COREQ	Three categories emerged: 1) "Personal experiences from pre-surgery participation", described how participation was perceived as challenging and sometimes stressful, but wellness improved. Cooperation with the physiotherapist was considered crucial and gave confidence. 2) "Attitudes to exercise", described exercise as an action of prevention and rehabilitation that demands motivation. Exercise was perceived to be good for you, physically but also improving mental health and other systems. 3) "Future physical activity - individual responsibility", described the return to former activities and potential challenges for the future. New knowledge was perceived to have changed the prerequisites for exercise and increased security in every-day physical activities.
Knutsson, Jong, Sayed-Noor, et al.	Spinal surgery	2022	Sweden	MD/Physician	Department of Surgical and Perioperative Science	All HIC	Orthopedic Reviews	***Waiting for lumbar spinal stenosis surgery: suffering and a possibility to discover coping abilities***	To describe aspects of suffering related to being a person with LSS and how suffering is managed before LSS surgery.	Adult	Spine, lumbar spinal stenosis	Sweden	Qualitative	Not reported	Patients	18	Interviews (open, unstructured, in-depth, semi-structured, open ended)	Content analysis (Used salutogenic model as a sensitizing concept)	Graneheim & Lundman (2004), Antonovsky (1979, 1987) & Oliveira (2015)	Not reported	The suffering from LSS before surgery included the main theme of experiencing an impaired physical and social life and struggling to be believed and taken seriously. This had coping strategies to manage symptoms before surgery: a good physician-patient relationship alleviates the burden of long waiting times; ways to manage pain and disability; ambiguous expectations and hope for recovery, and; ways to handle concerns before surgery).
Limback-Svensson, Kjellby Wendt, Thomeé, et al.	Spinal surgery	2013	Sweden	Not reported	Department of Orthopedics; Department of Physiotherapy and Occupational Therapy	All HIC	Journal of rehabilitation medicine	***Patients' experience of health three years after structured physiotherapy or surgery for lumbar disc herniation***	To describe the experience of health among pa-tients 3 years after treatment with a structured physiothera-py model or surgery for lumbar disc herniation	Adult	Spine	Sweden	Qualitative	Not reported	Patients	20	Interviews (open, unstructured, in-depth, semi-structured, open ended)	Content analysis	Krippendorff (2012)	Not reported	Findings were grouped into two themes: feeling of well-being and feeling of ill-being. In the group treated with structured physiotherapy there were a high number of codes in the feeling of well-being theme. In the group treated with surgery there were a high number of codes in the feeling of ill-being theme.
Ramstrom, Bunketorp-Kall and Wangdell	Spinal surgery	2021	Sweden	Not reported	Center for Advanced Reconstruction of Extremities, Dept of hand surgery	All LMIC	Disability and Rehabilitation	***The impact of upper limb spasticity-correcting surgery on the everyday life of patients with disabling spasticity: a qualitative analysis***	To explore patient perspectives of their experiences of daily life after spasticity-correcting surgery for UL (upper limb) spasticity resulting from spinal cord injury or stroke.	Adult	Spine; SCI & Stroke	Sweden	Phenomenology	Not reported	Patients	8	Interviews (open, unstructured, in-depth, semi-structured, open ended)	Phenomenological analysis	Alexandersson (1994)	COREQ	5 themes emerged: bodily changes, improved occupational performance, regained control, enhanced interpersonal interactions, enhanced psychological wellbeing. All participants believed treatment beneficial.
Rullander, Isberg, Karling, et al.	Spinal surgery	2013	Sweden	RN/Nurse	Faculty of Medicine, Department of Clinical Sciences, Pediatrics	All HIC	Pain Management Nursing	***Adolescents' Experience with Scoliosis Surgery: A Qualitative Study***	This article reports a study of adolescents' narrated experiences of undergoing scoliosis surgery.	Mixed	Spine; Scoliosis	Sweden	Qualitative	Not reported	Patients	6	Interviews (open, unstructured, in-depth, semi-structured, open ended)	Content analysis: Downe-Wamboldt 1992	Graneheim & Lundman (2004); Miles & Huberman (1994)	Not reported	The three main categories of experience were emotional, physical, and social. The emotional aspects that emerged were fear, nightmares, nervousness, and helplessness. These had a great impact on adolescents' well-being before, during, and after the hospital visit. The physical aspects were mobilization, scars, different hip levels, pain, nausea, appetite, and urinary catheter. These aspects caused much discomfort, mostly during the hospital visit. The social aspects were friends, power, coaching and comfort, and sports. Some of the social aspects had a strong negative impact on the adolescents' well-being mostly after the hospital visit.
Rullander, Jonsson, Lundstrom, et al.	Spinal surgery	2013	Sweden	RN/Nurse	Department of Nursing,	All HIC	Orthopedic Nursing	***Young people's experiences with scoliosis surgery: A survey of pain, nausea, and global satisfaction***	To describe how a cohort of young people and their parents retrospectively rate postoperative pain and nausea and describe their experiences of scoliosis surgery.	Mixed	Spine; Scoliosis	Sweden	Cohort study	Not reported	Patients AND Family members, carers, signifcant others	116	Semi-structured questionnaire	Content analysis: Deductive	Elo & Kyngas (2008)	Not reported	A total of 51 patients (59%) and 65 parents (75%) answered the questionnaires. Out of the completed questionnaires, 41 had idiopathic, 23 neuromuscular, and 6 other types of scoliosis. Postoperative patient-rated pain was severe 7.3 (median, interquartile range 5-8.4, visual analogue scale 0-10 cm), and the severe pain lasted for 5 (median, 2.7-7.0) days. Nausea was rated to a median of 5 (1.1-7.3) and lasted for a median of 3 (1-5.2) days. Global satisfaction was rated to a median of 3.2 (1.5-5.2). Postoperative pain was the most prominent issue, and present pain was found in 51% of respondents. Nausea and loss of appetite were common during the entire hospital stay. Waiting for the nurses' assistance, lack of control, and technical failures with the analgesia equipment caused discomfort. Parents experienced a lack of confidence in the nurses and felt helpless to support their child or relieve the child's suffering.
Witkam, Kurt, van Dongen, et al.	Spinal surgery	2021	The Netherlands	Not reported	Department of Anesthesiology, Pain and Palliative Medicine	All HIC	Neuromodulation	***Experiences From the Patient Perspective on Spinal Cord Stimulation for Failed Back Surgery Syndrome: A Qualitatively Driven Mixed Method Analysis***	To qualitatively and quantitatively map the FBSS patients' experiences with SCS and the effects of SCS on low back pain caused by FBSS.	Adult	Spine; Spinal Cord Stimulation; Failed Back Surgery Syndrome	the Netherlands	Mixed methods	Not reported	Patients	13	Interviews (open, unstructured, in-depth, semi-structured, open ended)	Thematic analysis	Braun & Clarke (2003)	Not reported	Seven themes regarding patients' experiences, subdivided into 15 categories, were identified, including an understudied theme within this field of research, Spiritual Well-Being. "Acceptance" and "coping" emerged as pre-eminent motifs throughout these themes. Moreover, the realization of patients' expectations were variable throughout the presented themes. According to the BPI Questionnaire, four out of 13 patients (31%) had significant pain relief (≥50%). Seven out of 13 (54%) reported a ≥50% increase regarding enjoyment of life.
Mistry, J. and White, L. and Baraks, K. and Davis, C. and Parikh, P. and Schabrun, S. and Heneghan, N. and Noblet, T. and Walton, D. and Rushton, A.	Spinal surgery	2024	Canada / UK	Advanced practice physiotherapist	School of Physical Therapy; St Georges Hospital NHSFT	All HIC	BMC Musculoskeletal Disorders	***Patient lived experiences of functioning and disability following lumbar discectomy: a secondary analysis of qualitative data***	To explore patient lived experiences of functioning and disability following lumbar discectomy.	Adult	Lumbar discectomy	UK	Interpretive phenomenological analysis (IPA)	Interpretivist and International Classification of Functioning	Patients	9	Interviews (semi-structured, open, in depth)	Interpretive phenomenological analysis	Smith (2008)	SRQR	Nine participants met the eligibility criteria and their interview transcripts were analysed. Patient lived experiences of functioning and disability were captured by three overarching themes: Immediate impact following surgery, Multiple roads to recovery over 1 year, and Functioning influenced by personal loci of control. Each theme consisted of three subthemes which were subsequently mapped onto the ICF. Three subthemes mapped to the ICF's body component, 1 to activity and participation and 3 to environment. Two subthemes themes did not map onto the ICF.

Rushton, Jadhakhan, Masson, et al.	Spinal surgery	2020	Canada / UK	Not reported	School of Physical Therapy, Faculty of Health Sciences; Center of Precision Rehabilitation for Spinal Pain, School of Sport, Exercise and Rehabilitation Sciences	All HIC	PLoS ONE	***Patient journey following lumbar spinal fusion surgery (FuJourn): A multiCenter exploration of the immediate post-operative period using qualitative patient diaries***	To capture and understand the immediate recovery journey of patients following lumbar spinal fusion surgery and explore the interacting constructs that shape their journey.	Adult	Spine; Lumbar spinal fusion surgery	UK	IPA	hermeneutic, interpretative	Patients	28	Other: Patient diaries	IPA	Smith & Flowers (2009)	SRQR & COREQ	Participants provided diverse and vivid descriptions of recovery experiences. Three distinct recovery trajectories were identified: meaningful recovery (engagement in physical and functional activities to return to functionality/mobility); progressive recovery (small but meaningful improvement in physical ability with increasing confidence); and disruptive recovery (limited purpose for meaningful recovery). Important interacting constructs shaped participants' recovery including their pain experience and self-efficacy.
Alsaif, Hanan and Goodwin, Peter C. and Callaghan, Michael J. and Sudell, Lindsay and O'Neill, Terence W. and Yeowell, Gillian	Spinal surgery	2023	UK	Other: Physiotherapist	Centre for Epidemiology Versus Arthritis, Faculty of Biology, Medicine, And Health	All HIC	Musculoskeletal science & practice	***Patient and healthcare provider experience and perceptions of a preoperative rehabilitation class for lumbar discectomy: A qualitative study***	to develop an understanding of patient and HCP experiences and views of preoperative rehabilitation for LD, including an exploration of why patients do not attend.	Adult	Low back pain; lumbar discectomy	UK	Qualitative	Interpretivist paradigm	Patients AND clinicians	20	Mixed/multiple: Interviews AND focus groups	Thematic analysis	Braun and Clarke (2012)	COREQ	The preoperative class was a valuable service for both patients and HCPs. It provided a solution to staffing and time pressures. It provided the required education and exercise content helping the patients along their surgery pathway. Travel distance, transportation links, parking difficulty and cost, lack of knowledge about the class aims, and previous negative experiences were barriers to patient attendance.
Talbot, R. and Higham, R. and Croft, J. and Ainsworth, G. and Brown, S. and Kelly, R. and Stocken, D. and Thomson, S. and Rousseau, N.	Spinal surgery	2024	UK	Qualitative researcher	Clinical Trials Research Unit, Leeds Institute of Clinical Trials Research	All HIC	Trials	***Rapid qualitative analysis of recruitment obstacles in the FORVAD (Posterior Cervical Foraminotomy surgery versus Anterior Cervical Discectomy surgery in the treatment of cervical brachialgia) randomised, controlled trial***	to understand the experiences of healthcare professionals who participated in the FORVAD Trial, with the aim of informing future research in this area.	Adult	Cervical brachialgia	UK	Qualitative	Normalisation process theory	HCPs (Inc NSx)	18	Interviews (semi-structured, open, in depth)	Thematic analysis	Vindrola-Padros (2020)	Not reported	Four main themes were generated in the data analysis: (1) individual vs. community equipoise; (2) trial set-up and delivery; (3) identifying and approaching patients; and (4) timing of randomisation. The objectives of the FORVAD trial made sense to participants and they supported the idea that there was clinical or collective equipoise regarding the two FORVAD interventions; however, many surgeons had treatment preferences and lacked individual equipoise. The site which had most recruitment success had adopted a more structured process for identification and recruitment of patients, whereas other sites that adopted more "ad hoc" screening strategies struggled to identify patients. Randomisation on the day of surgery caused both medico-legal and practical concerns at some sites.
Thomson, S. and Ainsworth, G. and Selvanathan, S. and Kelly, R. and Collier, H. and Mujica-Mota, R. and Talbot, R. and Brown, S. T. and Croft, J. and Rousseau, N. and Higham, R. and Al-Tamimi, Y. and Buxton, N. and Carleton-Bland, N. and Gledhill, M. and Halstead, V. and Hutchinson, P. and Meacock, J. and Mukerji, N. and Pal, D. and Vargas-Palacios, A. and Prasad, A. and Wilby, M. and Stocken, D.	Spinal surgery	2023	UK	Unclear/not stated	Department of Neurosurgery	All HIC	Health Technology Assessment	***Posterior cervical foraminotomy versus anterior cervical discectomy for Cervical Brachialgia: the FORVAD RCT***	To explore patients' experiences of the FORVAD trial and their reasons for taking part, and explored staff experiences of recruiting to the FORVAD trial and neurosurgery trials in general.	Adult	Cervical brachialgia	UK	Mixed methods (RCT with rapid qualititative study)	Normalisation process theory	Patients AND clinicians	20	Interviews (semi-structured, open, in depth)	Rapid thematic analysis	Vindrola-Padros (2020)	Not reported	Three main themes were identified in the data analysis: equipoise in the FORVAD trial and in neurosurgical trials, organisation and implementation, and integration of clinical and recruitment pathways.
Davis, Vincent, Henley, et al.	Spinal surgery	2013	UK	Not reported	Department of Surgery and Cancer	All HIC	Journal of Evaluation in Clinical Practice	***Exploring the care experience of patients undergoing spinal surgery: A qualitative study***	To explore the patient experience of the surgical journey from decision to operate, to hospitalization, discharge and subsequent recovery.	Adult	Spine; Spinal stenosis	UK	Qualitative	Not reported	Patients	7	Focus groups	Thematic analysis	Not reported	Not reported	Patients identified nine main 'needs' they felt played an integral part in enhancing the patient experience including the need for reduced waiting times, for better information and preparation, to be proactive, to speak up and ask questions, to feel safe and to be treated with dignity and respect; and the need for ongoing support, human contact, and; continuity of care.
Goodwin, Wright, Allan, et al.	Spinal surgery	2015	UK	Not reported	Department of Health Professions	All HIC	BMJ open	***Evidence-based development of a post-surgical lumbar discectomy leaflet intervention: a Delphi consensus study***	To produce free, expert-informed postoperative information for lumbar discectomy patients, satisfying UK National Health Service Information Standards.	Unclear	Spine; Lumbar discectomy	UK	Mixed methods (Delphi; focus groups)	Not reported	Patients AND HCPs (inc. NSx)	51	Mixed: Delphi AND Focus groups	Delphi	Not reported	Not reported	Response rates of 85%, 26% and 35% were achieved for the Delphi rounds. Ten clinicians and six patients participated in the focus groups. Consensus for leaflet sections was achieved in round 1 and content in round 3. The focus groups informed further revisions. A consensually agreed, Information Standard compliant, patient lumbar discectomy leaflet was produced containing: (1) normal spine anatomy; (2) anatomy disc herniation and surgery; (3) back protection strategies and (4) frequently asked questions. Illustrations of exercises enable tailoring to the individual patient.
Honeyman and Davison	Spinal surgery	2016	UK	RN/Nurse	James Cook University Hospital	All HIC	Nursing children and young people	***Patients' experience of adolescent idiopathic scoliosis surgery: a phenomenological analysis***	To explore how adolescents interpret their perioperative experience of scoliosis surgery.	Adolescents	Spine; Scoliosis	UK	Phenomenology (Interpretive)	Interpretivism	Patients	6	Interviews (open, unstructured, in-depth, semi-structured, open ended)	Thematic analysis	Not reported	Not reported	Four themes were identified: shock, fears and worries; parental interaction; coping; and motivation and positivity.

Low, Burgess and Wainwright	Spinal surgery	2019	UK	Not reported	Therapy Outpatient Department	All HIC	Medicina (Lithuania)	***A critical analysis of the exercise prescription and return to activity advice that is provided in patient information leaflets following lumbar spine surgery***	To critically Analyze the current postoperative aspects of rehabilitation (exercise prescription and return to normal activity) that are provided in patient information leaflets in England as part of an evaluation of current practice following lumbar spine surgery.	N/A	Low back pain	UK	Mixed methods	Not reported	Other	Other: 32	Documentary sources: patient leaflets	Content analysis	Wainwright & Burgess (2018); Dixon-Woods (2001); Grime & Ong (2007)	Not reported	Thirty-two patient information leaflets on lumbar surgery were sourced (fusion, n = 11; decompression, n = 15; all lumbar procedures, n = 6). Many of the exercises prescribed within the leaflets were not based on evidence of clinical best practice and lacked a relationship to functional activity. Return to normal activity advice was also wide ranging, with considerable variation in the recommendations and definitions provided.
Ryan, Pope and Roberts	Spinal surgery	2020	UK	Physiotherapist	Physiotherapy service; School of Health Sciences,	All HIC	BMJ Open	***Why managing sciatica is difficult: Patients' experiences of an NHS sciatica pathway. A qualitative, interpretative study***	To explore how people experience being managed for sciatica within an National Health Service (NHS) pathway.	Adult	Spine; Sciatica	UK	Qualitative; (Interpretive)	Not reported	Patients	14	Interviews (open, unstructured, in-depth, semi-structured, open ended)	Thematic analysis	Braun & Clarke (2006)	COREQ	A series of problems with the local pathway (insufficient transparency and information; clinician-led decisions; standardized management; restricted access to specialist care; and a lack of collaboration between services) made it difficult for patients to access the management they perceived necessary. Patients were therefore required to be independent and proactive or have agency. This was, however, difficult to achieve (due to the impact of sciatica and because patients lacked the necessary skills, funds and support) and together with the pathway issues, this negated patients' capability to manage sciatica. Theme 1) Problems with the pathway Theme 2) Required Agency Theme 3) The burden of Agency
Ryan, Eldabe, Chadwick, et al.	Spinal surgery	2019	UK	Reader in physiotherapy	School of Health and social care	All HIC	Neuromodulation	***An Exploration of the Experiences and Educational Needs of Patients With Failed Back Surgery Syndrome Receiving Spinal Cord Stimulation***	To explore the experience of spinal cord stimulation (SCS) for patients with failed back surgery syndrome (FBSS).	Adult	Spine; Failed back surgery syndrome; Spinal cord stimulation	UK	Qualitative	Not reported	Patients	12	Interviews (open, unstructured, in-depth, semi-structured, open ended)	Thematic analysis	Braun & Clarke (2006)	Not reported	Six themes were identified; 1) What should I expect? 2) Varied outcomes, 3) Understanding pain and this new treatment, 4) Experiences of the SCS journey, 5) Getting used to the device, and 6) Finding out what I need to know. Participants' expectations were varied and the procedures were broadly viewed as minor surgery. Participants' expectations about SCS were not limited to pain relief and included reductions in medication, better sleep, and increased physical activity. Participants' understanding of pain and how SCS purports to work was limited. Throughout the process, practical challenges were identified such as the surgical wound management and battery recharging. Participants received information from multiple sources and identified a range of key information needs including a quick-start guide on how to operate the device and a list of dos and don'ts.
Taylor-Robinson and Trovato	Spinal surgery	2021	UK	Not reported	Department of surgery	All HIC	Patient Preference and Adherence	***Semi-elective cervical disc replacements for cervical myelopathy: A qualitative study***	We relate the experience of a medically qualified patient in having disc prolapse at three cervical levels and what it was like to experience a lonely and difficult post-surgical recovery.	Adult	Nerve; Cervical Myelopathy	UK	Not stated	Not reported	Patients	1	Interviews (open, in-depth, semi-structured, open ended)	Thematic analysis	Not reported	Not reported	Eventually, after much deliberation and countless scans, he was slated for a further operation, a full 8 months after the first. This time, large foraminotomies were made to release the still trapped nerves, using a posterior approach.5 While the classic teaching is that it is more difficult to recover from this type of operation than the anterior approach, the patient found himself back at work within 6 weeks and shortly afterwards, in Abu Dhabi once more. He reflected "I had come full circle and had learnt much about the resilience that our patients need in order to survive and eventually flourish.
Williamson, Bulley and Coutts	Spinal surgery	2008	UK	Physiotherapist	Physiotherapy Department,	All LMIC	Disability and Rehabilitation	***What do patients feel they can do following lumbar microdiscectomy? A qualitative study***	To gain insight into patients' experiences of physiotherapy and activity choices during this period.	Adult	Spine; Lumbar microdiscectomy	UK	Qualitative	Constructivism	Patients	8	Interviews (open, unstructured, in-depth, semi-structured, open ended)	Thematic analysis	Not reported	Not reported	Three major themes were derived inductively from the data. The first, 'wish for precise movement boundaries,' described participants reduced levels of activity postoperatively as being related to high levels of anxiety about the surgery and fear of re-injury. The second theme suggested that physiotherapy failed to help participants explore their potential for activity. The final theme described post-operative fatigue.
Bull and Grogan	Spinal surgery	2010	UK	Trainee health psychologist	Center for Health Psychology	All HIC	Journal of Health Psychology	***Children having spinal surgery to correct scoliosis: A qualitative study of parents' experiences***	To develop an understanding of the experiences of parents whose children have scoliosis, in relation to diagnosis, surgical correction and recovery.	Pediatric	Spine; Adolescent idiopathic scoliosis	UK & US	IPA	Not reported	Family members, carers, signifcant others	13	Mixed: Interviews (open, unstructured, in-depth, semi-structured, open ended) AND questionnaires	IPA	Smith & Osborn (2007)	Not reported	Five themes emerged: 'Information'; 'Parenting role'; Confidence in professionals'; 'Pain'; and 'Effect on life'. Findings suggested that parents need appropriate information and support from health professionals throughout their experience to help minimize uncertainty and distress and that pain management is a major source of stress to parents.
Garrity, Singer, Ward, et al.	Spinal surgery	2020	USA	Medical student	Division of General Pediatrics	All HIC	Journal of patient experience	***Parent Perspectives on Short-Term Recovery After Spinal Fusion Surgery in Children With Neuromuscular Scoliosis***	To assess the postoperative experiences of children with neuromuscular scoliosis and their families to provide a roadmap for a more comprehensive conversation about recovery and to better prepare future parents and patients considering spinal fusion.	Pediatric	Neuromuscular scoliosis; Spinal fusion	United States	Qualitative	Not reported	Family members, carers, signifcant others	18	Interviews (open, unstructured, in-depth, semi-structured, open ended)	Grounded theory analysis/constant comparison/open, axial and/or selective coding	Not reported	Not reported	Five themes emerged among families when reflecting back on the postoperative recovery: (1) communicating and making shared decisions regarding postoperative care in a patient- and family-centered manner, (2) setting hospital discharge goals and being ready for discharge, (3) planning for transportation from hospital to home, (4) acquiring supports for caregiving at home after discharge, and (5) anticipating a long recovery at home. Important family perceptions were elicited about the recovery of children from spinal fusion for neuromuscular scoliosis that will inform better perioperative planning for clinicians, future patients, and their families.
Gregory, M. E. and Truelove, A. and Ahmad, F. and Corwin, D. and Tzimenatos, L. and Oglesbee, S. J. and Herman, M. J. and Leonard, J. C.	Spinal surgery	2023	USA	Other: Associate professor	Department of Health Outcomes and Biomedical Informatics	All HIC	Journal of the American College of Emergency Physicians Open	***Decision-making for pediatric cervical spine imaging after blunt trauma: Investigating team dynamics in the emergency department***	to examine team dynamics related to evaluation of pediatric trauma patients and decision-making around cervical spine imaging and how this may affect implementation of clinical decision support for pediatric cervical spine imaging.	Paediatric	Cervical spine injury	USA	Phenomenology	framework on health care teams, constructivist/ interpretivist paradigm.	Mixed HCPs: Inc. NSx	48	Interviews (semi-structured, open, in depth)	Thematic analysis (Deductive)	Braun and Clarke (2006) and Braun and Clarke (2012)	SRQR	Overall, emergency physicians and trauma surgeons indicate being generally responsible for pediatric cervical spine imaging deci- sions. Conflict often occurs between these specialties due to differential weighting of concerns for missing an injury versus avoiding radiation exposure. Participants described a lack of trust and unclear roles regarding ownership for the final imag- ing decision. Nurses commonly described low psychological safety that prohibits them from participating in the decision-making process.

Brintz, C. E. and Coronado, R. A. and Schlundt, D. G. and Jenkins, C. H. and Bird, M. L. and Bley, J. A. and Pennings, J. S. and Wegener, S. T. and Archer, K. R.	Spinal surgery	2023	USA	Unclear/not stated	Department of Anesthesiology, Vanderbilt University Medical Center,	All HIC	Spine	***A Conceptual Model for Spine Surgery Recovery A Qualitative Study of Patients' Expectations, Experiences, and Satisfaction***	To develop a conceptual model for Spine Surgery Recovery in order to better understand why patients undergo lumbar spine surgery and what factors influence patient satisfaction.	Adult		USA	Qualitative	Non stated	Patients	32	Mixed/multiple: Interviews AND focus groups	Other: inductive/deductive qualitative analysis approach	Azungah (2018); Bingham, Witkowsky (2021); Fereday, Muir-Cochrane (2006)	Not reported	A total of 1,355 coded quotes were analyzed. The decision to have lumbar spine surgery was influenced by chronic pain impact on daily function, pain coping, and patient expectations. Results demonstrated that fulfilled expectations and setting realistic expectations are key factors for patient satisfaction after surgery, while less known constructs of accepting limitations, adjusting expectations, and optimism were found by many patients to be essential for a successful recovery. Emotional factors of fear, anxiety, and depression were important aspects of pre- and post-surgical experiences.
Burton, W. and Salsbury, S. A. and Goertz, C. M.	Spinal surgery	2024	USA	Unclear/not stated	Division of Preventive Medicine; Osher Center for Integrative Health	All HIC	Bmc Health Services Research	***Healthcare provider perspectives on integrating a comprehensive spine care model in an academic health system: a cross-sectional survey***	to better understand healthcare clinician perceptions of potential barriers and facilitators to the integration of guideline-concordant spine care services for patients with low back pain	Adult	LBP	USA	Survey/Questionnaire	Non stated	Mixed HCPs: Inc. NSx	21	Open ended survey questions	Content analysis	Hsieh and Shannon (1995)	Other: STROBE	Qualitative analysis yielded 6 themes regarding comprehensive spine care: 1) Patient expectations/satisfaction, 2) Clinician experiences, 3) Comprehensive/ multidisciplinary spine care, 4) Appropriate role of surgery, 5) Referral processes/patient access, and 6) Administrative concerns. Patient expectations and satisfaction emerged as influential drivers of clinicians' treatment decisions.
Desai, R. H. and L'Hotta, A. and Kennedy, C. and James, A. S. and Stenson, K. and Curtin, C. and Ota, D. and Kenney, D. and Tam, K. and Novak, C. and Fox, I.	Spinal surgery	2023	USA	Other: Occupational Therapist	Program in Occupational Therapy	All HIC	Topics in Spinal Cord Injury Rehabilitation	***Caregiving for People With Spinal Cord Injury Undergoing Upper Extremity Reconstructive Surgery: A Prospective Exploration of Lived Experiences, Perioperative Care, and Change Across Time***	To investigate the perspectives and needs of caregivers of people with cervical spinal cord injury.	Adult	Spine	USA	Qualitative	Non stated	Family/Carer/Significant other	23	Interviews (semi-structured, open, in depth)	Thematic analysis	Vaismoradi, Turunen and Bondas (2015)	Not reported	The surgeries often brought hope and motivation for caregivers. Caregivers reported increased burden immediately following surgery (less for the NT compared to TT subgroup) yet no long-term changes in the amount and type of care they provided. NS caregivers discussed social isolation, relationship dysfunction, and everyday challenges
Lentz, T. A. and Stephens, B. F. and Abtahi, A. M. and Schwarz, J. and Schoenfeld, A. J. and Rhoten, B. A. and Block, S. and O'Brien, A. and Archer, K. R.	Spinal surgery	2023	USA	Other: PhD-trained investigator with qualitative interview experience	Department of Orthopaedic Surgery; Duke Clinical Research Institute	All HIC	Bmc Medical Informatics and Decision Making	***Leveraging web-based prediction calculators to set patient expectations for elective spine surgery: a qualitative study to inform implementation***	This study evaluated provider and patient perspectives on clinical implementation of web-based prediction calculators developed using national prospective spine surgery registry data from the Quality Outcomes Database.	Adult	not specified	USA	Qualitative	Non stated	Other: Mixed stakeholders	58	Interviews (semi-structured, open, in depth)	Other: qualitative template analysis	non stated	Not reported	Health care providers, administrators and patients overwhelmingly supported the use of the calculators to help set realistic expectations for surgical outcomes. Some clinicians had questions about the validity and applicability of the calculators in their patient population. A consensus was that the calculators needed seamless integration into clinical workflows, but there was little agreement on best methods for selecting which patients to complete the calculators, timing, and mode of completion. Many interviewees expressed concerns that calculator results could influence payers, or expose risk of liability. Few patients expressed concerns over additional survey burden if they understood that the information would directly inform their care.
Sedney, C. L. and Okakpu, U. and Dekeseredy, P. and Pollini, R. A. and Rosenow, J. M. and Haggerty, T.	Spinal surgery	2024	USA	Unclear/not stated	Neurosurgery	All HIC	Cureus Journal of Medical Science	***Back Pain in the Era of Opioid Restriction and Implications for Neurosurgeons Based on Qualitative Findings From a Rural State***	to investigate the impacts of opioid prescribing restrictions through a sequential, mixed methods study evaluating prescription trends and stakeholder experiences.	Adult	Back pain	USA	Qualitative	Mixed methods interpretivism	Patients AND clinicians	50	Interviews (semi-structured, open, in depth)	Content analysis	Hsieh and Shannon (2005)	Not reported	Five theoretical domains relevant to the treatment of back pain emerged, describing the prevalence of opioid use, barriers to access care, the importance of opioids for function in resource-poor rural areas, disconnected and siloed care, and patient views on the impacts of pain care gaps and solutions. Spinal pain care in rural WV is complex due to identified challenges. Care siloing factors in suboptimal spinal pain care. Future work should define, implement, and assess the real-world effectiveness of treatment paradigms for the full spectrum of surgical and non-surgical back pain complaints. Neurosurgeons should be present in this arena.
Barnard, Albright, Morrato, et al.	Spinal surgery	2013	USA	Not reported	Children's Outcomes Research Program, Children's Hospital	All HIC	Health and Social Care in the Community	***Pediatric spinal fusion surgery and the transition to home-based care: Provider expectations and carer experiences***	To better understand the surgical experience and evaluate the impact on carers during the CAPSS (Care Pathway for Spinal Surgery) program	Pediatric	Spine	USA	Qualitative	Not reported	Family members, carers, signifcant others AND HCPs (inc. surgeons)	30	Interviews (open, unstructured, in-depth, semi-structured, open ended)	Content analysis	Hsieh & Shannon (2005)	Not reported	Results showed the program was effective at improving preoperative surgical evaluation and helping families to anticipate some aspects of the surgical experience and hospital discharge. However, the impact of spinal fusion surgery and the subsequent transition to home-based care was profoundly emotional for patients and their carers. Our data indicate that program providers underestimated the extent of emotional trauma experienced by patients and families, particularly during the at-home recovery process. The data also suggest meaningful differences in providers' and carers' expectations for surgery. Carers' disappointment with their recovery experiences and the perceived lack of post-discharge support impacted their interpretations of and perspectives on their surgical experience.
Bove, Lynch, Ammendolia, et al.	Spinal surgery	2018	USA	Physical Therapist	Department of physical therapy	All HIC	Spine Journal	***Patients' experience with nonsurgical treatment for lumbar spinal stenosis: a qualitative study***	To assess the opinions of participants in a randomized clinical trial of nonsurgical LSS treatments regarding the interventions they received, factors contributing to adherence to the interventions, and methods of outcomes assessment.	Adult	Lumber Spinal Stenosis	USA	Qualitative	Not reported	Patients	50	Focus groups	Grounded theory analysis/constant comparison/open, axial and/or selective coding	Not reported	Not reported	Several themes were evident across all treatment groups. First, patients prefer individualized treatment that is tailored to their specific impairments and functional limitations. They also want to learn self-management strategies to rely less upon formal health care providers. Participants consistently stated that exercise improved their pain levels and physical function. However,they noted that these effects are temporary, so commitment to exercising long-term is important. Common barriers to completing the assigned LSS treatment included transportation issues and other comorbid health conditions. All three treatment groups cited perceived treatment benefit as a strong facilitator to continuing treatment. In addition, the ability of the health care provider to relate to the patient and listen to the patient's concerns was a common facilitator. Within the community-based group exercise treatment arm, most individuals continued group exercise after study completion, and social support was often mentioned as a facilitator to continuing treatment. Medical care was most often associated with minimal to no effect of treatment.

Etingen, Carbone, Guihan, et al.	Spinal surgery	2021	USA	Not reported	Center of Innovation for Complex Chronic Healthcare (CINCCH), Health Services Research and Development Service, Department of Veterans Affairs	All HIC	Journal of Spinal Cord Medicine	***Lower extremity fracture prevention and management in persons with spinal cord injuries and disorders: The patient perspective***	To describe patient experiences with fracture prevention and management among persons with spinal cord injuries/disorders (SCI/D).	Adult	Spine (veterans)	USA	Qualitative	Not reported	Patients	32	Interviews (open, unstructured, in-depth, semi-structured, open ended)	Grounded theory analysis/constant comparison/open, axial and/or selective coding	Boyatzis (1998), Strauss & Corbin (1998)	Not reported	Patients with SCI/D report lacking substantive knowledge about bone health and/or fracture prevention, and following fracture, feel unable and/or hesitant to resume pre-fracture participation. They may not feel as engaged as they would like to be in establishing fracture treatment plans. They may benefit from ongoing discussions with providers about risks and benefits of fracture treatment options and consideration of subsequent function and participation, to ensure patients preferences are considered.
Harris, Muller, Shauver, et al.	Spinal surgery	2017	USA	MD/Physician	Section of Plastic Surgery, Department of Surgery	All HIC	Journal of Hand Surgery	***Checkpoints to Progression: Qualitative Analysis of the Personal and Contextual Factors That Influence Selection of Upper Extremity Reconstruction Among Patients With Tetraplegia***	To provide a comprehensive understanding of patients' experiences and identify points on the pathway where intervention will be most impactful to improve UER utilization.	Adult	Spine; SCI	USA	Qualitative: (cross-sectional observational)	Not reported	Patients	19	Interviews (open, unstructured, in-depth, semi-structured, open ended)	Grounded theory analysis/constant comparison/open and axial/selective coding	Corbin & Strauss (1990); Tolley et al (2016; Boeije (2002).	Not reported	Results—Our study yielded a conceptual model that describes the characteristics common to all patients who undergo UER. Patients who selected reconstruction proceeded stepwise through a shared sequence of steps: 1) functional dissatisfaction 2) awareness of UER and 3) acceptance of surgery. Patients' ability to meet these criteria was determined by three checkpoints: how well they coped, their access to information, and the acceptability of surgery. Extremely positive or negative coping prevented patients from moving from the Coping to the Information Checkpoint; thus, they remained unaware of UER and did not undergo surgery. A lack of knowledge regarding reconstruction was the strongest barrier to surgery among our participants.
Kleiber and Adamek	Spinal surgery	2013	USA	RN/Nurse	University of Iowa College of Nursing	All HIC	Journal of Clinical Nursing	***Adolescents' perceptions of music therapy following spinal fusion surgery***	To explore adolescents' memories about music therapy after spinal fusion surgery and their recommen- dations for future patients.	Adolescents	Spine, spinal fusion for adolescent idiopathic scoliosis	USA	Qualitative	Not reported	Patients	8	Interviews (open, unstructured, in-depth, semi-structured, open ended)	Content analysis (inductive)	Elo & Kyngäs (2007)	Not reported	The eight participants were 13–17 years of age and had surgery between 2–24 months previously. The overarching themes identified from the interviews were relaxation and pain perception, choice and control, therapist interaction and preoperative information. Participants stated that music therapy helped with mental relaxation and distraction from pain. It was important to be able to choose the type of music for the therapy and to use self-control to focus on the positive. Their recommendation was that future patients should be provided with information preoperatively about music therapy and pain management. Participants recommended a combination of auditory and visual information, especially the experiences of previous patients who had spinal fusion and music therapy.
Mancuso, Rigaud, Wellington, et al.	Spinal surgery	2021	USA	Not reported	Research Division; Department of Medicine	All HIC	European Spine Journal	***Qualitative assessment of patients' perspectives and willingness to improve healthy lifestyle physical activity after lumbar surgery***	To assess patients' perspectives about postoperative physical activity.	Adult	Spine, lumbar surgery	USA	Qualitative	Not reported	Patients	260	Mixed: Interviews (open, unstructured, in-depth, semi-structured, open ended) AND questionnaires	Grounded theory analysis/constant comparison/open and axial/selective coding (inductive comparative)	Pawluch and Neitermann (2010), Morse (2015), Malterud (2001), Mays and Pope (2000)	Not reported	Mean age was 63, and 53% were men. Thirty-nine percent thought walking was good for the spine, particularly among those with less fear-avoidance, less disability, and less complex surgery. Spine benefits were cited (42%) for the short-term ("faster recovery") and long-term ("decreases chances of another surgery"), particularly by younger patients. To increase activity, patients suggested spine-specific techniques (35%, "use railings") and advised caution (24%, "pace yourself"). The major deterrent was persistent back pain (36%) particularly for those with worse disability, and more depressive symptoms, anxiety, complex surgery, and fear-avoidance. Our findings consistently fit with the Social Cognitive Theory of health behavior which posits a dynamic three-way interaction of personal factors, environmental influences, and behavior.
McCarthy, Bove, Piva, et al.	Spinal surgery	2020	USA	Doctoral candidate	US Army-Baylor University Doctoral Program in Physical Therapy	All HIC	Journal of Orthopaedic and Sports Physical Therapy	***A qualitative study of preparation for lumbar spinal stenosis surgery: Perceptions of patients and physical therapists***	To gain the perspectives of patients who underwent lumbar spinal stenosis (LSS) surgery and physical therapists who treat spine-related disorders regarding rehabilitation and other care prior to LSS surgery.	Adult	Spine, lumbar spinal stenosis	USA	Qualitative (Interpretive description)	Not reported	Patients AND HCPs	26	Focus groups	Other: Interpretive description approach	Thurston et al (2014)	Not reported	Analyzes revealed 4 themes within the discussions: (1) desire for helpful information, (2) benefits of preoperative rehabilitation, (3)downfalls of preoperative rehabilitation, and (4) desire for coordinated care. Varying opinions on preoperative physical therapy between patients and physical therapists were discussed, revealing that similar numbers of participants held positive and negative perceptions of preoperative physical therapy. A desire for more thorough preoperative education and care was expressed by both groups.
Peterson, Mesa, Halpert, et al.	Spinal surgery	2021	USA	Physical therapist	The Motive Physical Therapy Specialists and Arizona School of Health Sciences	All HIC	Musculoskeletal Science and Practice	***How people with lumbar spinal stenosis make decisions about treatment: A qualitative study using the Health Belief Model***	To explore the beliefs of people with LSS and how they make decisions about treatment.	Adult	Lumbar spinal stenosis	USA	Qualitative	Health Belief Model	Patients	12	Interviews (open, unstructured, in-depth, semi-structured, open ended)	Content analysis	Hsieh & Shannon (2005)	COREQ	Twelve patients (mean age 75.3 years, range 63–87 years, 9 female, 6 with previous LSS surgery) participated. The Health Belief Model appeared useful for explaining decisions about treatment. Perceived threat of LSS was higher in those who had surgery. Patients who decided on surgery perceived themselves as more susceptible to surgery, often because of pathoanatomical beliefs. These patients had lower perceived control over symptoms and the treatment decision itself. Although patients saw benefit in conservative treatment because of its lower risk and ability to foster self-management, many had no or poor education and reported previous experiences with ineffective conservative treatment.
Schwartz, Ayandeh and Finkelstein	Spinal surgery	2015	USA	Not reported	DeltaQuest Foundation; Departments of Medicine and Orthopedic Surgery	All HIC	Health and quality of life outcomes	***When patients and surgeons disagree about surgical outcome: investigating patient factors and chart note communication***	To investigate indirect effects of physician-patient communication by examining the relationship between a physician-patient mismatch in perceived outcomes and content in the medical record's clinical note.	Adult	Spine	USA	Mixed methods	Not reported	Patients AND HCPs (inc. surgeons)	172	Mixed: Questionnaire AND clinical chart notes	Content analysis	Not reported	Not reported	Patient and surgeon agreed in 76% of cases and disagreed in 24% of cases. Patients who assessed their outcome worse than their surgeons tended to be less educated and involved in litigation. They also tended to report worsened mental health and leg pain. Content analysis revealed group differences in surgeon communication patterns in the chart notes related to how symptom change was emphasized, how follow-up was described, and a specific word reference. Specifically, disagreement was predicted by using "much" to emphasize the findings and noting long-term prognosis. Agreement was predicted by use of positive emphasis terms, having an "as-needed" follow-up plan, and using "happy" in the chart note

Whitebird, Solberg, Norton, et al.	Spinal surgery	2020	USA	Not reported	School of Social Work, Morrison Family College of Health	All HIC	Journal of patient-centered research and reviews	***What Outcomes Matter to Patients After Joint or Spine Surgery?***	What are the most important outcomes patients hope for from joint replacement or spinal surgery? To what extent were the scores they received on current PROMs (Oswestry Disability Index, Oxford Knee Score, Oxford Hip Score) useful to them? What information about their personal situation (i.e., personal contextual information) should their doctors be aware of in providing them with the best patient-centered care?	Adult	Spine; Spinal surgery; hip and knee replacement	USA	Qualitative	Not reported	Family members, carers, signifcant others	65	Interviews (open, unstructured, in-depth, semi-structured, open ended)	Content analysis	Hseich & Shannon (2005)	Not reported	Patients identified desired outcomes that were unique and important to them. Their preferred outcomes focused in the areas of freedom from pain, getting back to their normal life, and returning to an active lifestyle. Patients cared more about their individual preferred outcomes, which had more meaning for them, than a standardized PROM score. Patients also identified particular contextual situations that their care team was assumed to know about but that may not have been known.
Block, H. and George, S. and Hunter, S. C. and Bellon, M.	Traumatology	2024	Australia	Other: Occupational Therapist	Caring Futures Institute, College of Nursing and Health Sciences,	All HIC	Disability and Rehabilitation	***Family experiences of the management of challenging behaviours after traumatic brain injury in the acute hospital setting***	This study explored experiences of the management of challenging behaviours after traumatic brain injury (TBI) in the acute hospital setting from the perspectives of family members.	Adult	TBI	Australia	Interpretive phenomenology	The Ecological Systems Theory (EST)	Family/Carer/Significant other	10	Interviews (semi-structured, open, in depth)	Thematic analysis	Braun and Clark (2006); Nowell, Norris, White (2017)	COREQ	Four primary themes were identified: 1) The hospital environment; 2) Hospital staffing; 3) Identifying and preventing triggers, and 4) Family support and information.
Carrier, S. L. and Ponsford, J. and McKay, A.	Traumatology	2024	Australia	Other: clinical neuropsychology PhD candidate	School of Psychological Sciences; Monash-Epworth Rehabilitation Research Centre	All HIC	Neuropsychological Rehabilitation	***Family experiences of supporting a relative with agitation during early recovery after traumatic brain injury***	to explore the experiences of family members who were providing support for patients who were hospitalized, agitated and in PTA following moderate-severe TBI.	Adult	TBI; agitation, PTA	Australia	Qualitative descriptive	Constructionist epistemology	Family/Carer/Significant other	24	Mixed/multiple: Interviews AND focus groups	Reflexive thematic analysis (Inductive)	Braun & Clarke (2019)	COREQ	The themes identified from the interviews reflected factors considered important for improving patient recovery including: (1) family contributions to patient care; (2) expectations of the health care service; (3) supporting families to support patients.
Costello, R. S. and Downing, M. G. and Ponsford, J.	Traumatology	2024	Australia	Unclear/not stated	Monash Epworth Rehabilitation Research Centre; School of Psychological Sciences,	All HIC	Disability and Rehabilitation	***The experience of traumatic brain injury in a culturally and linguistically diverse sample in Australia***	to qualitatively explore the experience of injury, rehabilitation, and longer-term recovery in individuals from a culturally and linguistically diverse background following TB	Adult	TBI	Australia	Qualitative	critical realist	Patients	15	Interviews (semi-structured, open, in depth)	Reflexive thematic analysis (Inductive)	Braun and Clarke (2019)	COREQ	It was demonstrated that: (a) the cognitive and behavioural consequences of TBI were accompanied by stigma and loss of independence; (b) participants held many beliefs related to their TBI, ranging from bad luck to acceptance. Participants' personal values and beliefs provided strength and resilience, with many viewing the injury as a positive event in their lives; (c) participants were appreciative of the high standard of care they received in hospital and rehabilitation, although communication barriers were experienced; (d) many participants identified with Australian culture, and few believed their cultural background negatively impacted their experience of TBI; (e) external support, particularly from family, was considered central to recovery.
Elbourn, E. and Brassel, S. and Steel, J. and Togher, L.	Traumatology	2024	Australia	Speech pathologist	The University of Sydney	All HIC	International Journal of Language and Communication Disorders	***Perceptions of communication recovery following traumatic brain injury: A qualitative investigation across 2 years***	to examine the perspectives of individuals with severe TBI towards their communication, recovery and illness narratives from subacute through to post-acute care (6 months, 1 year and 2 years post-injury).	Adult	TBI	Australia	Qualitative	Non stated	Patients	12	Interviews (semi-structured, open, in depth)	Reflexive thematic analysis	Braun, et al. (2017)	Not reported	Three overarching themes were identified: experiences of communication recovery are diverse (Theme 1), varied experiences of recovery and rehabilitation (Theme 2), and continuous and lifelong journey of recovery (Theme 3). Primary communication concerns included presence of anomia, dysarthria, conversational topic difficulties, impacts of fatigue and memory difficulties. Illness narratives revealed the importance of re-establishing a sense of self and the perceived importance of a strong social network post-injury
Fitts, M. and Cullen, J. and Barney, J.	Traumatology	2023	Australia	Unclear/not stated	Institute for Culture and Society; Menzies School of Health Research; Australian Institute of Tropical Health and Medicine	All HIC	Australian Social Work	***Barriers Preventing Indigenous Women with Violence-related Head Injuries from Accessing Services in Australia***	to examine and understand what factors can prevent women from accessing hospital and support services following a TBI from family violence	Adult	TBI; Family violence	Australia	Qualitative	decolonising theoretical framework	Mixed: patients and community stakeholders	118	Mixed/multiple: Interviews AND focus groups	Thematic analysis (inductive)	Braun & Clarke (2022)	Not reported	Thematic analysis identified four key factors influencing women's access to health care: all women fear child removal; fear of escalating violence; prioritisation of other competing demands; and insufficient awareness of the signs of brain injury. Given child protection systems perpetuate cycles of discrimination based on poverty and structural inequalities that have generated fear and contributed to the reluctance of women to engage with services, child protection processes and practices need to be transformed to consider the impact of head injury on the everyday lives of women.
Fitts, M. and Soldatic, K.	Traumatology	2024	Australia	Unclear/not stated	Institute for Culture and Society; Menzies School of Health Research; Australian Institute of Tropical Health and Medicine	All HIC	Health Sociology Review	***Temporalities of emergency: the experiences of Indigenous women with traumatic brain injury from violence waiting for healthcare and service support in Australia***	to explore the temporality of waiting and its implications for violence-related TBI emergency care as a critical component of the larger project.	Adult	TBI; Family violence	Australia	Qualitative	decolonising theoretical framework	Mixed: Community service providers	88	Mixed/multiple: Interviews AND focus groups	Thematic analysis	Braun and Clarke (2013); Denzin and Lincoln (2011)	Not reported	The qualitative data suggest that hospital spaces and responses that specifically consider patients' psychological wellbeing, safety and security are essential components of healthcare for Indigenous women with TBI from family violence. Themes: Waiting for triage, medical monitoring and social services; Indigenous women's resistance to waiting.
Fitts, M. S. and Cullen, J. and Kingston, G. and Wills, E. and Soldatic, K.	Traumatology	2022	Australia	Unclear/not stated	Institute for Culture and Society; Menzies School of Health Research; Australian Institute of Tropical Health and Medicine	All HIC	International Journal of Environmental Research and Public Health	***"I Don't Think It's on Anyone's Radar": The Workforce and System Barriers to Healthcare for Indigenous Women Following a Traumatic Brain Injury Acquired through Violence in Remote Australia***	To identify community-based service provider perspectives on workforce and system-related barriers affecting pre-screening and healthcare access for Aboriginal and Torres Strait Islander women after experiencing a potential TBI following family violence	Adult	TBI; Family violence	Australia	Qualitative	decolonising theoretical framework	Mixed: Patients, hospital staff and community service providers	38	Mixed/multiple: Interviews AND focus groups	Thematic analysis (inductive)	Braun and Clarke (2006)	Not reported	The results highlighted various workforce barriers that affected pre-screening and diagnostic assessment including limited access to specialist neuropsychology services and stable remote primary healthcare professionals with remote expertise. There were also low levels of TBI training and knowledge among community-based professionals.
Hickey, Lyndal and Anderson, Vicki and Jordan, Brigid	Traumatology	2022	Australia	Unclear/not stated	The Royal Children's Hospital; Department of Paediatrics; Clinical Sciences Research, Murdoch Children's Research Institute	All HIC	Health & social care in the community	***Australian parent and sibling perspectives on the impact of paediatric acquired brain injury on family relationships during the first 6 weeks at home***	This study explores the impact of paediatric acquired brain injury (ABI) on family relationships	Paediatric	TBI	Australia	Survey/Questionnaire	Non stated	Family/Carer/Significant other	29	Open ended survey questions	Reflexive thematic analysis	Braun and Clark (2006)	Not reported	Family members (parents and siblings) described four themes: (1) negative changes in sibling interactions; (2) role changes arising from an increase in parental expectations of non-injured siblings; (3) family system challenges in balancing needs within the parent-child dyad and sibling subsystems; and (4) supporting emotional responses within the family system.

Hwang, J. H. A. and Downing, M. and Ponsford, J. L.	Traumatology	2024	Australia	Other: PhD candidate in neuropsychology	Monash-Epworth Rehabilitation Research Centre, School of Psychological Sciences,	All HIC	Neuropsychological Rehabilitation	***"Maybe if this was addressed sooner, maybe things might be different in our relationship. I don't know. But who knows?" Sexuality after TBI and its place in healthcare: A qualitative exploration of survivors' experiences***	To qualitatively explore TBI-related impacts on sexuality, experiences of help-seeking, and preferences of individuals with moderate-to-severe TBI around addressing sexuality with and receiving support from their health professionals.	Adult	TBI	Australia	Qualitative	Non stated	Patients	20	Interviews (semi-structured, open, in depth)	Reflexive thematic analysis	Braun and Clark (2022)	Not reported	Three broad themes were identified through reflexive thematic analysis of interview transcripts. First, individuals differed significantly at the start of their journeys in personal attributes, TBI-associated impacts, and comfort levels in discussing sexuality. Second, journeys, feelings, and perspectives diverged based on the nature of post-TBI sexuality. Third, whilst responses to changes and preferences for support varied widely, individuals felt that clinicians were well-placed to help them navigate this area of their lives.
McCausland, K. and Thomas, E. and Bullen, J. and Hill-Wall, T. and Norman, R. and Cowen, G.	Traumatology	2024	Australia	Other: Research fellow	Collaboration for Evidence, Research and Impact in Public Health, School of Population Health	All HIC	Health Promotion Journal of Australia	***Heads up on concussion: Aboriginal and Torres Strait Islander peoples' knowledge and understanding of mild traumatic brain injury***	we investigated the knowledge of concussion among Aboriginal and Torres Strait Islander peoples residing in Western Australia (WA), with the aim of better understanding the need for improved education and service provision for this vulnerable group.	Adult	mTBI	Australia	Qualitative	social constructivism	Public/lay	24	Interviews (semi-structured, open, in depth)	Reflexive thematic analysis	Braun and Clarke (2006)	SRQR	A good knowledge of modes of concussion injury was identified in these participants. However, they identified difficulty differentiating this injury from other injuries or medical conditions. Multiple factors contributed to a reluctance to seek assessment and further management of a potential concussion. Multiple strategies to enhance education and presentation for assessment were suggested by participants.
Nguyen, J. V. K. and Willmott, C. and Ponsford, J. and Davies, K. and Makdissi, M. and Drummond, S. P. A. and Reyes, J. and Makovec Knight, J. and Peverill, T. and Brennan, J. H. and McKay, A.	Traumatology	2024	Australia	Other: PhD candidate	Turner Institute for Brain and Mental Health, School of Psychological Sciences; Monash-Epworth Rehabilitation Research Centre	All HIC	Disability and Rehabilitation	***Moving forward on the road to recovery after concussion: participant experiences of interdisciplinary intervention for persisting post-concussion symptoms***	to explore participant experiences of i-RECOveR, an interdisciplinary intervention for PPCSs and its impact on symptoms, daily function, and concussion beliefs.	Adult	mTBI; persisting post-concussion symptoms	Australia	Qualitative	Not explicitly stated	Patients	13	Interviews (semi-structured, open, in depth)	Reflexive thematic analysis	Braun and Clarke (2022)	SRQR and COREQ	Three themes reflected participants' treatment journeys from concussion to life after treatment: (1) Dissatisfaction with Previous Consultations, reflected personal experiences prior to commencing treatment; (2) Perceived Active Ingredients of Intervention, reflected participant experiences of i-RECOveR; and (3) Impact of Interdisciplinary Intervention, reflected a range of positive changes after completing i-RECOveR.
O'Reilly, K. and Wilson, N. J. and Kwok, C. and Peters, K.	Traumatology	2023	Australia	Nurse/RN/RGN	Western Sydney University	All HIC	Journal of Clinical Nursing	***An exploration of women's sexual and reproductive health following traumatic brain injury***	To develop a gendered understanding of sexual and reproductive health for Australian women following traumatic brain injury.	Adult	TBI	Australia	Mixed methods,	Pragmatism	Patients	20	Interviews (semi-structured, open, in depth)	Thematic analysis	Braun and Clarke (2016)	GRAMMS	Women reported changes in menstruation, reproduction, and sexual activity. Results identified that following traumatic brain injury, routine reproductive, and sexual health screening were neglected. Women revealed a personal reticence to discuss sexual health in a holistic sense and appeared more comfortable discussing reproductive health rather than sex for pleasure. Additionally, they perceived there was a reticence by clinicians to discuss sexual health as part of their rehabilitation.
Ownsworth, T. and Bates, A. and Watter, K. and Morgan, C. and Bell, R. and Griffin, J. and Turner, B. and Kennedy, A. and Kendall, M. and Adams, B. and Gibson, E. and Hakala, T. and Mitchell, J.	Traumatology	2024	Australia	Unclear/not stated	The Hopkins Centre, Menzies Health Institute Queensland; School of Applied Psychology	All HIC	Health Expectations	***Reclaiming Agency in Care Decisions and Barriers From the Perspectives of Individuals With Acquired Brain Injury and Their Family Members***	to understand what constitutes self-advocacy and associated barriers and facilitators throughout hospital transitions and into the community.	Adult	MIXED ABI	Australia	Qualitative	Informed by a phenomenological approach	Patients AND Families/carers/ Significant other	25	Interviews (semi-structured, open, in depth)	Framework analysis	Pope (2000), Ritchie and Spencer (1994), Ritchie and Spencer (2003)	Not reported	Self-advocacy reflects the process of reclaiming agency or people's efforts to exert influence over care decisions after ABI. Agency varies along a continuum, often beginning with impaired processing of the self or environment (loss of agency) before individuals start to understand and question their care (emerging agency) and ultimately plan and direct their ongoing and future care (striving for agency). This process may vary across individuals and contexts. Barriers to self-advocacy for individuals with ABI include neurocognitive deficits that limit capacity and desire for control over decisions, unfamiliar and highly structured environments and lack of family support. Facilitators include neurocognitive recovery, growing desire to self-advocate and scaffolded support from family and clinicians.
Sansonetti, D. and Fleming, J. and Patterson, F. and Lannin, N. A.	Traumatology	2024	Australia	Unclear/not stated	School of Health and Rehabilitation Sciences; Occupational Therapy Department	All HIC	Neuropsychological Rehabilitation	***Profiling self-awareness in brain injury rehabilitation: A mixed methods study***	to identify differences and similarities in patterns of self-awareness between patients with different brain injury diagnoses, and provide a clinical account of how individuals with ABI describe changes to themselves arising from brain injury.	Mixed	TBI, stroke, hypoxia	Australia	Mixed methods,	Self-awareness Conceptualization Framework	Patients	173	Interviews (semi-structured, open, in depth)	Content analysis; Thematic analysis	Neuendorf (2017), Braun and Clarke (2013)	Not reported	Individuals identified a range of brain injury-related impairments across domains, with greatest difficulty noted with linking impairments to functional implications and setting realistic goals. There were similarities and distinct differences in the expression of changes across diagnostic groups. Two main themes that aligned with self-awareness theory were identified from the data: 1/ Development of self-awareness; and 2/ Dimensions of self-awareness. These interrelated themes demonstrated the multifaceted nature of the clinical presentation of self-awareness, and highlight the need for an individualized approach to cognitive rehabilitation.
Wills, E. and Fitts, M.	Traumatology	2024	Australia	Unclear/not stated	Institute for Culture and Society	All HIC	Health Expectations	***Listening to the Voices of Aboriginal and Torres Strait Islander Women in Regional and Remote Australia About Traumatic Brain Injury From Family Violence: A Qualitative Study***	to gain insight into Indigenous women's personal and family perspectives regarding violence-related traumatic brain injury (TBI), including impacts on life, as well as decision-making processes about healthcare access and engagement.	Adult	TBI	Australia	Qualitative	Not explicitly stated	Mixed (Patients and community members, including family members or carers of TBI patients)	46	Mixed/multiple: Interviews, focus groups	Thematic analysis	Braun and Clarke (2006)	Not reported	Three themes were conceptualised based on the data and research aims: interweaving of the past and the present-ways women experience brain injury; factors that inform decision-making to access healthcare; and managing everyday changes that result from TBI from family violence. Indigenous women described living with a range of symptoms following repeated head injuries including problems with memory, cognition and concentration. A range of strategies to manage long-term symptoms of TBI were used by Indigenous women and when they did seek healthcare, Indigenous women were required to navigate a range of barriers.
Zhang, E. and Steel, J. and Togher, L. and Fromm, D. and MacWhinney, B. and Bogart, E.	Traumatology	2024	Australia	Unclear/not stated	University of Sydney	All HIC	Journal of speech, language, and hearing research : JSLHR	***Insights From Important Event Recounts Told by People With Traumatic Brain Injury***	to investigate quantitative and qualitative changes in important event recounts produced by a group of people with severe TBI up to 2 years postinjury.	Mixed	TBI	Australia	Mixed methods,	Not explicitly stated	Patients	34	Monologic discourse tasks (free speech, recounts, picture descriptions)	Framework analysis	Tuthill (2020)	Not reported	The content analysis revealed categories of (a) childhood events, (b) family and relationships, (c) career and education, and (d) grief and loss. Topics at 6 months focused on childhood events and holidays, whereas career and education predominated at 24 months.

Alston, Jones and Curtin	Traumatology	2012	Australia	Not reported	Social Work, Monash University	All HIC	Australian Social Work	***Women and Traumatic Brain Injury: "It's not visible damage"***	To explore the experiences of women with TBI.	Adult	TBI	Australia	Qualitative	Poststructuralist feminist analysis	Patients	11	Interviews (open, in-depth, semi-structured, open ended)	Grounded theory analysis/constant comparison/open, axial and/or selective coding	Not reported	Not reported	Altered Sense of Self, Body Image, and Being Able to "Pass" as Normal; Power, Control, and Social Isolation; Caring and Scripted Gender Roles
Analytis, Warren and Ponsford	Traumatology	2020	Australia	Not reported	Monash-Epworth Rehabilitation Research Center, Turner Institute for Brain and Mental Health, Brain Injury and Rehabilitation Program, School of Psychological Sciences.	All HIC	Brain Injury	***The sibling relationship after acquired brain injury (ABI): perspectives of siblings with ABI and uninjured siblings***	To explore the sibling relationship from the perspective of siblings with ABI and uninjured siblings across childhood, adolescence and adulthood.	Mixed	Mixed ABI	Australia	Qualitative	Critical realist ontology and interpretivist epistemology	Patients AND Family members, carers, signifcant others	39	Interviews (open, unstructured, in-depth, semi-structured, open ended)	Reflexive thematic analysis	Braun et al (2019)	Not reported	Four themes were identified: Living with ABI; Being normal siblings; Being part of a family; Experiencing social stigma of ABI. ABI was a traumatic event which differentiated siblings but increased understanding helped accommodate its impact. This impact was experienced within perceptions of typical sibling relationships as involving closeness and conflict. The family context shaped relationships, with parents mediating across the lifespan, while siblings' partners and children contributed to adult sibling relationships. Finally, social stigma led to distance but also protectiveness between siblings.
Armstrong, Coffin, McAllister, et al.	Traumatology	2019	Australia	Not reported	School of Medical & Health Sciences	All HIC	Brain Impairment	***I've got to row the boat on my own, more or less': Aboriginal Australian experiences of traumatic brain injury***	(i) to highlight issues faced by Australian Aboriginal male brain injury survivors and (ii) to highlight the real life consequences of the high incidence of TBI and inadequacy of treatment for this population.	Adult	TBI	Australia	Case study	Not reported	Patients	5	Mixed: Interviews (open, unstructured, in-depth, semi-structured, open ended) AND medical file review	Thematic analysis	Sandelowski, 2000; Taylor & Bogdan, 1994	Not reported	Common themes included: significant long-term life changes; short-term and long-term dislocation from family and country as medical intervention and rehabilitation were undertaken away from the person's rural/remote home; family adjustments to the TBI including permanent re-location to a metropolitan area to be with their family member in residential care; challenges related to lack of formal rehabilitation services in rural areas; poor communication channels; poor cultural security of services; and lack of consistent follow-up.
Armstrong, Coffin, Hersh, et al.	Traumatology	2021	Australia	Not reported	School of Medical & Health Sciences	All HIC	Disability and Rehabilitation	***"You felt like a prisoner in your own self, trapped" the experiences of Aboriginal people with acquired communication disorders***	(i) to explore the attitudes and experiences of Aboriginal people with brain injury after both stroke and brain trauma across WA, (ii) to explore their experiences of having a communication disorder in everyday life, the support/ rehabilitation services accessed, and how such services could be improved, and (iii) to utilize the above to inform new models of service delivery.	Adult	TBI	Australia	Qualitative	Bronfenbrenner's socioecological model	Patients AND Family members, carers, signifcant others	49	Interviews (open, unstructured, in-depth, semi-structured, open ended) - yarning method	Thematic analysis (data were approached using a framework based on Bronfenbrenner's socioecological model)	Not reported	Not reported	Overall themes related to communication (both related to the communication disorder and general healthcare interactions), health and social contexts, recovery, and support, being away from family and country, knowledge and beliefs about brain injury, and follow-up.
Bellon, Crocker, Farnden, et al.	Traumatology	2015	Australia	Not reported	Disability and Community Inclusion, School of Health Sciences	All HIC	Brain Impairment	***Family Support Needs Following Acquired Brain Injury Across Metropolitan and Regional/Remote South Australia***	To identify and compare, family support needs following an acquired brain injury (ABI) in metropolitan and regional/remote areas in order to inform the development of a state-wide family peer support network.	Adult	Mixed ABI	Australia	Mixed methods	Not reported	Patients AND Family members, carers, signifcant others	197	Mixed: Open ended questions AND follow-up focus group	Content analysis	Graneheim & Lundman (2004)	Not reported	Thematic analysis of open-ended survey responses and focus group transcripts revealed 15 areas of needed support. Although all themes were identified by both geographic groups, regional/remote participants commented more frequently on the need for coordinated, accessible and tailored services. A strong focus was placed on the need for counselling and emotional support, as well as family support groups from both major city and regional/remote participants. Each support was reviewed to identify those which could be augmented through peer-supports, including: emotional support; family support groups; ABI information; family social activities; help to navigate the system; early supports (within the first year of ABI); and self-advocacy training.
Brassel, Kenny, Power, et al.	Traumatology	2016	Australia	Speech and language pathology research student	Faculty of Health Sciences	All HIC	Brain Injury	***Conversational topics discussed by individuals with severe traumatic brain injury and their communication partners during sub-acute recovery***	To investigate the nature and patterns of conversational topics discussed by individuals with severe TBI and familiar communication partners at 3 and 6 months post-injury, and to examine changes occurring in conversational topics during sub-acute recovery.	Adult	TBI	Australia	Qualitative (descriptive)	Interpretive paradigm, naturalistic paradigm	Patients AND Family members, carers, signifcant others	44	Other: 10 minute casual conversations	Content analysis (inductive, manifest and latent)	Graneheim & Lundman (2004)	COREQ	Three main conversational themes were identified: connecting; re-engaging; and impacts of injury. The nature of topics related to these themes changed over time to reflect participants' sub-acute rehabilitation experiences. Most conversational dyads maintained similar conversational and topic patterns during sub-acute recovery.
Brown, Whittingham, Sofronoff, et al.	Traumatology	2013	Australia	Not reported	The School of Psychology	All HIC	Brain Injury	***Parenting a child with a traumatic brain injury: Experiences of parents and health professionals***	To explore the experiences, challenges and needs of parents of children with traumatic brain injury (TBI) in order to inform future intervention research through incorporation of participant knowledge and experience.	Pediatric	TBI	Australia	Qualitative	Not reported	Family members, carers, signifcant others AND HCPs	15	Mixed: Focus group OR interview	Thematic analysis (inductive)	Braun & Clarke (2006)	Not reported	Participants reported that, beyond the impact of the injury on the child, TBI affects the entire family. Parents need to adjust to and manage their child's difficulties and can also experience significant emotional distress, relationship discord and burden of care, further adding to the challenges of the parenting role. Parents can feel isolated and the importance of empowerment, support and information was emphasized. Coping styles of disengagement and avoidance were often reported, despite acknowledgement that these were not beneficial.
Chamberlain	Traumatology	2006	Australia	RN/Nurse	Lecturer, Critical Care Studies, School of Nursing and Midwifery	n/a	Journal of advanced nursing	***The experience of surviving traumatic brain injury***	To describe the experience of surviving traumatic brain injury as narrated by individuals 1 year after injury	Adult	TBI	Australia	Mixed methods (Extended Glasgow Outcome Score; Qualitative interviews)	Not reported	Patients	60	Interviews (open, unstructured, in-depth, semi-structured, open ended)	Content analysis	Berg (1989) Burnard (1991)	Not reported	Twelve per cent returned to full preinjury functioning, 35% had residual psychosocial and physical sequelae, 38% had significant restrictions in lifestyle and work capacity, and 15% were unable to care for themselves throughout the 24-hour period. The common narratives were classified into five categories: 'regret and grief within self'; 'insensitivity of health professionals'; 'invisibility of self'; 'stranded self'; and 'recovery in self'. These self-narratives reflected renewed ways to view the self, which were conceptualized to be intact 'in spite of' or to be worthwhile 'because of' the traumatic brain injury.
Checklin and Stephens	Traumatology	2021	Australia	Not reported	Speech Pathology Rehabilitation	All HIC	Brain Impairment	***Pictures and a thousand words: The experiences of significant others whose loved ones have a severe brain injury who are being discharged home***	To investigate the experiences of significant others when their loved one with a severe acquired brain injury (ABI) is being discharged from an ABI-specific rehabilitation unit.	Adult	Mixed ABI	Australia	Qualitative	Not reported	Family members, carers, signifcant others	8	Mixed: Interviews (open, unstructured, in-depth, semi-structured, open ended) AND drawings method	Thematic analysis	Braun & Clarke (2006)	Not reported	Five themes were identified (Change, Mixed feelings, Support of family and friends, Journey and Staff interactions).

Checklin, Fernon, Soumilas, et al.	Traumatology	2020	Australia	Not reported	Speech Pathology Department	All HIC	Disability and rehabilitation	***What is it like to have your loved one with a severe brain injury come to rehabilitation? The experiences of significant others***	To investigate the experiences of significant others when their loved one with a severe brain injury is admitted to acquired brain injury (ABI) rehabilitation.	Adult	Mixed ABI	Australia	Qualitative	Not reported	Family members, carers, signifcant others	9	Mixed: Interviews (open, unstructured, in-depth, semi-structured, open ended) AND drawing method	Thematic analysis	Braun & Clarke (2006)	Not reported	Seven themes were identified from the two sources of data. Themes included: Trauma; Relief; Interactions; Change; Grief and loss; Journey, and Uncertainty.
Di Battista, Godfrey, Soo, et al.	Traumatology	2014	Australia	Psychologist (incl. clinical, neuro-)	Child Neuropsychology, Murdoch Children's Research Institute, Australia	All HIC	Journal of Rehabilitation Medicine	***"In my before life": Relationships, coping and post-traumatic growth in adolescent survivors of a traumatic brain injury***	To explore the individual, adolescent phenomenology of quality of life after traumatic brain injury.	Adolescents	TBI	Australia	Qualitative	Not reported	Patients	10	Interviews (open, unstructured, in-depth, semi-structured, open ended)	IPA AND and Multi-threaded DNA	Smith & Osborn (2003)	Not reported	Two major findings: (1) perceived quality of life was not automatically impacted by a traumatic brain injury, but when it was, the directionality of impact (positive, negative) varied depending on the life-domain; (2) changes in ability post-traumatic brain injury were attributed to the injury (more often cognitive and physical changes) or to a sense of normal maturation processes (72% and 28%, respectively). Attribution processing permeated themes of personal and social discrepancies, which also yielded themes of: altered family and relationships, roles, responsibilities, independence, coping and post-traumatic growth. All participants reported a happy life at the time of interview.
Douglas	Traumatology	2020	Australia	Speech pathologist and clinical neuropsycholog ist,	Living with Disability Research Center, College of Science, Health and Engineering	N/A	Neuropsycholo gical Rehabilitation	***Loss of friendship following traumatic brain injury: A model grounded in the experience of adults with severe injury***	To understand the post-injury experience of friendship from the perspective of adults with severe TBI.	Adult	TBI	Australia	Mixed methods (Convergent design)	Constructivist	Patients	23	Mixed: Interviews (open, unstructured, in-depth, semi-structured, open ended) AND questionnaires	Grounded theory analysis/consta nt comparison/op en, axial and/or selective coding	Charmaz, (2006)	Not reported	Exploratory correlations between number of friends and quality of life, depression and strong-tie support revealed significant associations of moderate to large effects. The post-injury experience of friendship was broadly conceptualized as "going downhill" with four overlapping phases: losing contact, being misunderstood, wanting to share and hanging on.
Downing, Hicks, Braaf, et al.	Traumatology	2021	Australia	Not reported	Monash Epworth Rehabilitation Research Center (MERRC) and School of Psychological Sciences; Turner Institute for Brain and Mental Health	All HIC	Neuropsycholo gical Rehabilitation	***Factors facilitating recovery following severe traumatic brain injury: A qualitative study***	To examine positive factors identified for recovery by individuals who had sustained severe TBI three years earlier.	Adult	TBI	Australia	Qualitative	Critical realist perspective	Patients AND Family members, carers, signifcant others	25	Interviews (open, unstructured, in-depth, semi-structured, open ended)	Reflexive thematic analysis	Braun & Clarke (2006)	Not reported	Using reflexive thematic analysis, three themes were identified as positive for recovery after a TBI. Having a support network included social supports such as family and friends, and receiving other funded/non-funded assistance towards improving independence and participation. Being positive and engaged included being able to participate, being positive, using compensatory strategies, and becoming fit, healthy and happy. Getting good care included patients perceiving they had a comprehensive and good quality hospital experience, and access to multidisciplinary outpatient services.
Downing, Hicks, Braaf, et al.	Traumatology	2021	Australia	Not reported	Monash Epworth Rehabilitation Research Center (MERRC) and School of Psychological Sciences	All HIC	Disability and Rehabilitation	***"It's been a long hard road" challenges faced in the first three years following traumatic brain injury***	To investigate challenges to recovery identified by individuals who sustained severe TBI three years earlier or their close others (COs), as well as suggestions for managing these challenges.	Adult	TBI	Australia	Qualitative	Critical realist perspective	Patients AND Family members, carers, signifcant others	25	Interviews (open, unstructured, in-depth, semi-structured, open ended)	Reflexive thematic analysis	Braun & Clarke (2006)	Not reported	Challenges experienced across all timeframes included: lack of information and poor communication, pre-existing conditions, missed injuries, and issues with medical staff, and continuity of care. From acute care onwards, there were TBI-related consequences, issues with coping and emotional adjustment, negative outlook, insufficient treatment, lack of support for COs, and issues with compensation and funding for rehabilitation needs. Some challenges were unique to a specific timeframe (e.g., over-stimulating ward setting during acute care, and limited or unsupportive families once injured individuals went home). Suggestions for managing some of the challenges were provided (e.g., information provision, having peer supports).
Fitts, Bird, Gilroy, et al.	Traumatology	2019	Australia	Not reported	College of Public Health, Medical and Veterinary Sciences	All HIC	Brain Impairment	***A Qualitative Study on the Transition Support Needs of Indigenous Australians following Traumatic Brain Injury***	To understand the lived experiences of Indigenous Australians during the 6 months post-discharge, identify the help and supports accessed during transition and understand the gaps in service provision or difficulties experienced	Adult	TBI	Australia	Qualitative	Not reported	Patients	11	Interviews (open, unstructured, in-depth, semi-structured, open ended)	Thematic analysis	Braun & Clarke (2006)	Not reported	While some of the transition experiences for Indigenous Australians were similar to those found in other populations, the transition period for Indigenous Australians is influenced by additional factors in hospital and during their recovery process. Lack of meaningful interaction with treating clinicians in hospital, challenges managing direct contact with multiple service providers and the injury-related psychological impacts are some of the factors that could prevent Indigenous Australians from receiving the supports they require to achieve their best possible health outcomes in the long term. A holistic approach to care, with an individualized, coordinated transition support, may reduce the risks for re-admission with further head injuries.
Gould, Hicks, Hopwood, et al.	Traumatology	2019	Australia	Psychologist (incl. clinical, neuro-)	Monash-Epworth Rehabilitation Research Center, Epworth Healthcare and Monash Institute of Cognitive and Clinical Neurosciences, School of Psychological Sciences	All HIC	Neuropsycholo gical Rehabilitation	***The lived experience of behaviors of concern: A qualitative study of men with traumatic brain injury***	To explore the lived experience of BoC in individuals with TBI, their close others and clinicians	Adult	TBI	Australia	Qualitative	Critical realism	Patients AND Family members, carers, signifcant others AND HCPs	25	Interviews (open, unstructured, in-depth, semi-structured, open ended)	Thematic analysis	Braun & Clarke (2006, 2013)	Not reported	Frequent and persistent BoC were reported and the key themes identified included the brain injury, control, environment, mood, identity, social relationships, and meaningful participation. Whilst the brain injury contributed to BoC in all cases, the way the other themes manifested and interacted was variable.
Knox, Douglas and Bigby	Traumatology	2015	Australia	Not reported	Department of Human Communication Sciences	All HIC	Brain Injury	***'The biggest thing is trying to live for two people': Spousal experiences of supporting decision-making participation for partners with TBI***	To understand how the spouses of individuals with severe TBI experience the process of supporting their partners with decision-making.	Adult	TBI	Australia	Grounded theory	Constructivism	Family members, carers, signifcant others	4	Interviews (open, in-depth, semi-structured, open ended)	Grounded theory analysis/consta nt comparison/op en and axial/selective coding	Charmaz (2006)	Not reported	Two main themes emerged from the data. The first identified the saliency of the relational space in which decision-making took place. The second revealed the complex nature of decision-making within the spousal relationship.

Knox, Douglas and Bigby	Traumatology	2016	Australia	Speech Pathologist	Department of Human Communication Sciences, Faculty of Health Sciences	All HIC	Neuropsychological Rehabilitation	***"I won't be around forever": Understanding the decision-making experiences of adults with severe TBI and their parents***	To explore processes used by adults with severe TBI and their parents in making decisions about life after injury.	Adult	TBI	Australia	Grounded theory	Constructivism	Patients AND Family members, carers, signifcant others	8	Interviews (open, unstructured, in-depth, semi-structured, open ended)	Grounded theory analysis/constant comparison/open and axial/selective coding	Charmaz (2006, 2008), Corbin and Strauss (2008)	Not reported	In line with this construct, two central themes described processes of joint decision making within parent – adult child relationships after severe TBI over time: (1) making decisions with parental support, and (2) reducing parental involvement.
Lefkovits, Hicks, Downing, et al.	Traumatology	2020	Australia	Not reported	School of Psychological Sciences	All HIC	Neuropsychological Rehabilitation	***Surviving the "silent epidemic": A qualitative exploration of the long-term journey after traumatic brain injury***	To qualitatively investigate the experience of TBI many years post-injury, beginning with reflections on acute recovery through to living long-term with the injury.	Adult	TBI	Australia	Qualitative	Not reported	Patients	30	Interviews (open, unstructured, in-depth, semi-structured, open ended)	Thematic analysis	Braun & Clarke (2006)	Not reported	Results demonstrated that: (a) although some participants reported full recovery, several experienced persistent physical, cognitive and emotional problems that impacted their independence, employment and interpersonal relationships; (b) early rehabilitation was very helpful, but some participants experienced difficulties accessing ongoing services; (c) family and social support were important to recovery; (d) most participants drew upon inner strength to find positives in their experience.
Liddle, Fleming, McKenna, et al.	Traumatology	2011	Australia	Not reported	Division of Occupational Therapy, School of Health and Rehabilitation Sciences	All HIC	Disability and Rehabilitation	***Driving and driving cessation after traumatic brain injury: Processes and key times of need***	To explore the processes and experiences of driving and driving cessation after TBI with a focus on what could help to improve outcomes.	Adult	TBI	Australia	Phenomenology (Descriptive)	Not reported	Patients AND Family members, carers, signifcant other AND HCPs AND non-clinical providers	35	Interviews (open, unstructured, in-depth, semi-structured, open ended)	Thematic analysis	Langdridge (2007); Patton, (2002)	Not reported	The findings reveal experiences of the process of driving and driving cessation contextualized within experiences of the accident and treatment process. Participants identified key times of need in relation to driving: being told about driving restrictions, understanding driving restrictions, the 'on hold' period, and returning to driving.
Liddle, Fleming, McKenna, et al.	Traumatology	2012	Australia	Occupational therapist	School of Health and Rehabilitation Sciences	All HIC	Australian Occupational Therapy Journal	***Adjustment to loss of the driving role following traumatic brain injury: A qualitative exploration with key stakeholders***	To examine loss of the driving role and to explore the outcomes associated with driving cessation from the perspectives of key people involved within the process: people with TBI, their family members and involved health professionals.	Adult	TBI	Australia	Phenomenology (Descriptive)	Not reported	Patients AND Family members, carers, signifcant other AND HCPs	35	Interviews (open, unstructured, in-depth, semi-structured, open ended)	Grounded theory analysis/constant comparison/open and axial/selective coding	Patton (2002)	Not reported	This article focuses on two themes, each with three subthemes. Being stuck: needs related to driving cessation had subthemes: (i) an emotional time, (ii) being normal and (iii) participation without driving. The second theme, A better way: suggestions to improve outcomes had subthemes: (i) information, (ii) support and trying it out and (iii) their family member's roles and needs.
Maskell, Chiarelli and Isles	Traumatology	2007	Australia	Not reported	Discipline of Physiotherapy, Faculty of Health	All HIC	Brain Injury	***Dizziness after traumatic brain injury: Results from an interview study***	To better define the nature of the symptomatology and the impact that dizziness has on the TBI survivor.	Adult	TBI	Australia	Qualitative	Not reported	Patients AND Family members, carers, signifcant others	18	Mixed: Focus groups AND interviews	Descriptive analysis	Ritchie, Spencer & O'Connor (2003)	Not reported	The data confirmed that dizziness is difficult for TBI survivors to define and describe and it consists of multiple symptoms. Dizziness also appears to be associated with significant functional difficulties. Additionally, many of the participants of this study reported falling. Carers reported a number of observable signs of dizziness and indicated that they believed they were able to tell when the person they cared for was dizzy.
McCluskey, Johnson and Tate	Traumatology	2007	Australia	Not reported	University of Sydney	All HIC	Brain Impairment	***The process of care management following brain injury: A grounded theory study***	To explore how decisions about care and support were made following brain injury, when an individual had received third party compensation and to identify factors or conditions that influenced care.	Adult	TBI	Australia	Grounded theory	Not reported	Family members, carers, signifcant others AND HCPs AND non-clinical providers	51	Interviews (open, unstructured, in-depth, semi-structured, open ended)	Grounded theory analysis/constant comparison/open and axial/selective coding	Glaser & Strauss (1967)	Not reported	All 14 people with brain injury had received 24-hour support at the time of hospital discharge; at follow-up, only 8 received 24-hour support. Care decision-making and care maintenance occurred as an iterative process. Participants made decisions about the best living situation, configuration of carers and level of care, and then strived to maintain stable care. Care decision-making involved three key strategies (gathering information, appraising alternatives, and enacting a decision). Care maintenance also involved three key strategies (monitoring, managing conflict and supporting carers). Mutual risk tolerance and the presence of a case manager were necessary conditions for good care management, and enabled a person with brain injury to spend time alone, take low-level risks and experience autonomy.
McIntyre, Ehrlich and Kendall	Traumatology	2020	Australia	Not reported	Synapse Australia Ltd.	All HIC	Disability and Rehabilitation	***Informal care management after traumatic brain injury: perspectives on informal carer workload and capacity***	(1) what is the nature of the work being performed by informal carers of people with TBI, and (2) what are the factors that impact carer capacity to carry out that work?	Adult	TBI	Australia	Qualitative	Not reported	Patients AND Family members, carers, signifcant others	42	Interviews (open, unstructured, in-depth, semi-structured, open ended)	Thematic analysis	Braun & Clarke (2006)	Not reported	Results revealed two main themes and eight subcategories: (1) The nature of informal care: describing informal care management work, (personal assistant work; care provider work; family support work; and emotional self-regulation work), and (2) Mediating factors that impacted people's capacity to manage workload (carer intrinsic factors; injured person characteristics; family circumstances; and changes over time.)
Mealings and Douglas	Traumatology	2010	Australia	Post-graduate research student	Community Integration Team, Epworth Rehabilitation	All HIC	Brain Impairment	***'School's a big part of your life.: Adolescent perspectives of their school participation following traumatic brain injury***	To hear the stories of three male adolescent students as they reflected on their own experiences of what it was like to go back to school after sustaining a severe TBI.	Adult	TBI	Australia	Grounded theory	Social interactionism	Patients	3	Interviews (open, unstructured, in-depth, semi-structured, open ended)	Grounded theory analysis/constant comparison/open, axial and/or selective coding	Browne (2004); Patton, 2002	Not reported	Three key themes emerged from the interviews: the adolescent student's sense of self, changes the students noted; and supports the students identified. A tentative model illustrating the relationship between these areas was developed. It is anticipated that this model will assist clinicians and educators to develop a holistic picture of a student's school participation from transition to ongoing school life
Mealings, Douglas and Olver	Traumatology	2021	Australia	Not reported	School of Allied Health, & Epworth Rehabilitation	All HIC	Brain Injury	***The student journey: Living and learning following traumatic brain injury***	To gain further understanding of the experiences of students participating in secondary and tertiary education following TBI: exploring academic and non- academic factors, as well as changes in experiences over time	Adult	TBI	Australia	Grounded theory	Constructivist, symbolic interactionism	Patients	12	Interviews (open, unstructured, in-depth, semi-structured, open ended)	Grounded theory analysis/constant comparison/open, axial and/or selective coding	Charmaz (2006, 2014)	Not reported	Students' participation experiences were unique and varied with different timelines and outcomes, however they shared many similar critical points. We interpreted their experiences as a student journey traveling through four significant landscapes, "Choosing to study", "Studying", "Deciding what to do", "Making the next step." Journeys involved complex processes of living and learning. Moving along the pathway was not always smooth or straightforward
Mealings, Douglas and Olver	Traumatology	2020	Australia	Not reported	University College of Science Health and Engineering / Epworth Healthcare	All HIC	Neuropsychological Rehabilitation	***Is it me or the injury: Students' perspectives on adjusting to life after traumatic brain injury through participation in study***	To explore the role that participating in education plays in self-conceptualization for students after sustaining TBI; in recognition of research evidence suggesting participation in everyday life plays an important role in shaping the experience of self following TBI	Adult	TBI	Australia	Grounded theory	Constructivist	Patients	12	Interviews (open, unstructured, in-depth, semi-structured, open ended)	Grounded theory analysis/constant comparison/open, axial and/or selective coding	Charmaz (2006, 2014)	Not reported	A significant theme of "Is it Me or is it the Injury? "emerged from the context of students' descriptions of self, "Me" and thoughts about their injury, "The Injury." This emergent theme was indicative of the complex processes involved in adjusting and reshaping identity that arose from students' participation in education. The concepts identified in this research suggest that clinicians and educators must adopt a comprehensive, holistic and flexible approach to supporting students that can be adapted to reflect the individual and dynamic processes involved.

Minney, Roberts, Mathias, et al.	Traumatology	2019	Australia	Psychologist (incl. clinical, neuro-)	School of Psychology	All HIC	Brain Injury	***Service and support needs following pediatric brain injury: perspectives of children with mild traumatic brain injury and their parents***	To provide a qualitative examination of the service and support needs of children who have had a mild traumatic brain injury (mTBI), and their parents, in order to improve clinical services.	Pediatric	TBI	Australia	Qualitative	Not reported	Patients AND Family members, carers, signifcant others	18	Interviews (open, unstructured, in-depth, semi-structured, open ended)	Thematic analysis	Braun & Clarke (2006, 2013)	Not reported	Post-injury needs were reflected in four main themes: Communication; Family Burden; Continuity of Care; and Social and Community Support. These themes reflected children's and parents' needs for information, emotional/social/community support, and follow-up care. Both the children's and parents' needs, and the extent to which they were met, appeared to be related to the severity and duration of the child's PCS.
Nalder, Fleming, Cornwell, et al.	Traumatology	2012	Australia	Occupational therapist & PhD candidate	School of Health and Rehabilitation Sciences	All HIC	Brain Impairment	***Linked lives: The experiences of family caregivers during the transition from hospital to home following traumatic brain injury***	To understand the experiences of family caregivers during the transition from hospital to home, defined as the first six months postdischarge.	Adult	TBI	Australia	Qualitative (phase 2 of a sequential explanatory mixed methods design)	Interpretive paradigm; Life course theory	Family members, carers, signifcant others	10	Interviews (open, unstructured, in-depth, semi-structured, open ended)	Framework analysis	Ritchie & Lewis (2003)	Not reported	The overarching theme was that caregivers wished to move past the injury. This desire to move forward stemmed from a realisation of how their life had changed and the weight of the care responsibility. Caregivers were also aware of how the life of the individual with a TBI had changed and hoped for a return to normality (by regaining independence, engaging in meaningful occupation and having meaningful relationships).
O'Callaghan, McAllister and Wilson	Traumatology	2012	Australia	Not reported	The University of Queensland	All HIC	Brain injury	***Insight vs readiness: factors affecting engagement in therapy from the perspectives of adults with TBI and their significant others***	To look beyond the development of self-awareness and insight in order to explore the concept of readiness as it relates to clients' experiences of engaging with therapy	Adult	TBI	Australia	Qualitative	Interpretive paradigm	Patients AND Family members, carers, signifcant others	23	Interviews (open, unstructured, in-depth, semi-structured, open ended)	Thematic analysis	Attride-Sterling (2001), Morse (1995)	Not reported	The results of this study indicated that participants believed their ability to engage in therapy was related to the degree to which they were aware of and accepted their impairments and motivated to engage in rehabilitation. Participants reported that the rate and degree to which they developed awareness varied according to the type of impairments they experienced (i.e. physical vs cognitive). Participants also stated that one way to augment their awareness was to compare their new vs old selves and to compare their recovery to those of others with a similar impairment. Finally, participants reported that it was important to be able to access services when they had accepted their impairment and were ready to engage in therapy.
O'Reilly, Wilson, Kwok, et al.	Traumatology	2021	Australia	RN/Nurse	Western Sydney University	All HIC	Journal of Advanced Nursing	***Women's tenacity following traumatic brain injury: Qualitative insights***	To explore perspectives of Australian women who had sustained a traumatic brain injury to develop a gendered understanding of their experiences.	Adult	TBI	Australia	Qualitative (findings from a Mixed methods study)	Critical social theory	Patients	20	Interviews (open, unstructured, in-depth, semi-structured, open ended)	Thematic analysis	Braun & Clarke (2013, 2016, 2019)	Not reported	Two overarching themes Loss-A life once lived and Realigning Self-A new way of being were identified. Women discussed many losses due to injury, loss of income and potential earnings, loss in relationships and loss of identity. These all took time to reconfigure in their lives, as they adjusted to a range of ongoing impairments from the traumatic brain injury.
Robson, Ziviani and Spina	Traumatology	2005	Australia	Not reported	Princess Alexandra Hospital,	All HIC	Brain Impairment	***Personal Experiences of Families of Children with a Traumatic Brain Injury in the Transition from Hospital to Home***	To explore the experiences and perceptions of parents of children with TBI during this important transition time with the aim to better inform health-workers involved in discharge management and rehabilitation.	Pediatric	TBI	Australia	Phenomenology	phenomenological & inductive	Family members, carers, signifcant others	6	Interviews (open, unstructured, in-depth, semi-structured, open ended)	Thematic: Content analysis (inductive)	Patton (1990).	Not reported	The themes which emerged related to the emotional journey experienced by the family, the impact of significant relationships in the process of adjustment, along with service provision and coping strategies. These themes are highlighted with respect to how they can influence intervention strategies. Implications for service referral and the communication style of health professionals are also addressed.
Simpson, Mohr and Redman	Traumatology	2000	Australia	Not reported	Brain Injury Rehabilitation Unit	All HIC	Brain Injury	***Cultural variations in the understanding of traumatic brain injury and brain injury rehabilitation***	To research cultural variations in the understanding of TBI and the rehabilitation process, interviewing 39 people with TBI and family members from Italian, Lebanese and Vietnamese backgrounds.	Adult	TBI	Australia	Qualitative	Not reported	Patients AND Family members, carers, signifcant others	39	Interviews (open, unstructured, in-depth, semi-structured, open ended)	Thematic analysis	Patton (1990); Miles & Huberman (1994)	Not reported	Findings suggest there is a universal experience of TBI that transcends individual cultures. Study participants valued attentiveness, friendliness and guidance from rehabilitation staff. Family support was not always available to the person with TBI due to family conflict. Generally, people with TBI and family members valued the assistance of health interpreters facilitating their communication with rehabilitation staff. People with TBI from all three cultures experienced problems of stigma and social isolation.
Tam, McKay, Sloan, et al.	Traumatology	2015	Australia	Not reported	School of Psychological Sciences	All HIC	Brain Injury	***The experience of challenging behaviors following severe TBI: A family perspective***	To understand how family caregivers of individuals with TBI perceive challenging behaviors and their impact on the TBI individual's community integration and family functioning.	Adult	TBI	Australia	Qualitative	Not reported	Family members, carers, signifcant others	6	Interviews (open, unstructured, in-depth, semi-structured, open ended)	Thematic analysis	Braun and Clarke (2006, 2013)	Not reported	The results revealed that family caregivers adopted a broader definition of challenging behavior than that used by professionals and these behaviors impacted on the community integration of the individual with TBI, most notably leading to poor social relationships. Challenging behaviors were viewed as a key source of distress and burden for family caregivers and they used many different strategies to manage the behaviors.
Ward, Shum, Dick, et al.	Traumatology	2004	Australia	Not reported	School of Applied Psychology and Neuropsychology Clinic,	All HIC	Brain Injury	***Interview study of the effects of Pediatric traumatic brain injury on memory***	To investigate the effects of traumatic brain injury (TBI) on children's day-to-day memory functioning.	Pediatric	TBI	Australia	Qualitative	Not reported	Family members, carers, signifcant others	13	Interviews (open, in-depth, semi-structured, open ended)	Content analysis	Berg (2001)	Not reported	Over half of the children experienced explicit (past recall) and prospective memory (future intentions) loss, but few experienced implicit memory (e.g. procedural) loss. Further, parents utilized their own interventions in minimizing their children's memory disabilities.
Gantner, D. and Cooper, D. J. and Finfer, S. and Bragge, P.	Traumatology	2022	Australia	Other: Intensive care clinician researcher	Australian and New Zealand Intensive Care Research Centre	All HIC	Neurocritical Care	***Determinants of Adherence to Best Practice in Severe Traumatic Brain Injury: A Qualitative Study***	To identify factors that infuence the adherence to evidence in the interdisciplinary acute management of patients with sTBI, with focus on choice of resuscitation fluids and decisions to perform surgery to treat intracranial hypertension, from the perspective of medical practitioners responsible for sTBI care.	N/A	TBI	Australia, European Union, North America	Qualitative	theoretical domains framework	HCPs (Inc NSx)	25	Interviews (semi-structured, open, in depth)	Thematic analysis	Non stated	SRQR	Key TDF domains were environmental context and resources, social influences, and beliefs about consequences. Evidence-aligned management of patients with sTBI is perceived to be facilitated by admission to academic research-oriented hospitals, development of local practice protocols, and interdisciplinary collaboration. Determinants of specific practices varied and included health policy change for fluid resuscitation and development of patient-centered goals for surgical decision-making.
Laic, R. A. G. and Vander Sloten, J. and Depreitere, B.	Traumatology	2023	Belgium	Unclear/not stated	Biomechanics Section,	All HIC	Brain and Spine	***In-depth assessment of quality of life and real life impact of mild traumatic brain injury in elderly by means of a focus group study***	To qualitatively investigate the difficulties that elderly patients with a mild TBI suffer after injury and how those affect their QoL, in order to better understand the impact of TBI on elderly patients' lives.	Adult	TBI	Belgium	Qualitative	Non stated	Patients	6	Focus groups	Thematic analysis	Dierckx de Casterle (2012)	Not reported	Three themes emerged from the analysis: functional disturbances and symptoms, daily life after TBI, and life quality, feelings and satisfaction. The most reported factors that deteriorated QoL 1–5 years post-TBI in our cohort were the lack of support from partners and families, changes in self-perception and social life, tiredness, balance disturbances, headache, cognitive deterioration, changes in physical health, senses' disturbances, changes in sexual life, sleep problems, speech disturbances and dependence for daily life activities.
Verlinde, L. and Verlinde, F. and Van Doren, S. and De Coninck, D. and Toelen, J.	Traumatology	2024	Belgium	Unclear/not stated	Medicine	All HIC	Injury Prevention	***Cycle safe or cycle cool? Adolescents' views on bicycle helmet use and injury prevention campaigns in Belgium***	to understand the facilitators and barriers to bicycle helmet use by adolescents and their perspectives on injury prevention campaigns.	Adolescents	Head injury	Belgium	Qualitative	Not explicitly stated	Public/lay	84	Focus groups	Thematic analysis	Castleberry (2018), Kiger (2020)	Not reported	Four key themes regarding adolescents' views on safe cycling practices emerged from the analysis: external motivation, internal motivation, factors specific to the helmet and the cycling environment. The main barriers to bicycle helmet use identified by adolescents were peer pressure, appearance and discomfort. The perceived risks of cycling without a helmet among adolescents were low. Mandatory bicycle helmet laws and non-legislative programmes were considered to be an effective strategy by the study participants. Parental strategies, including strict parental rules and parental helmet use, further contributed to wear a bicycle helmet.

Mbakile-Mahlanza, Manderson, Downing, et al.	Traumatology	2017	Australia	Psychologist (incl. clinical, neuro-)	School of Psychological Sciences	All HIC	Disability and Rehabilitation	***Family caregiving of individuals with traumatic brain injury in Botswana***	The present study reported focused on the experience of caregiving for individuals with TBI in Botswana. It also aimed to examine levels of caregiver anxiety and depression, and their association with functional outcome in their TBI relative.	Adult	TBI	Botswana	Mixed methods (Concurrent design)	Not reported	Family members, carers, signifcant others	18	Mixed: Interviews (open, unstructured, in-depth, semi-structured, open ended) AND questionnaires	Thematic analysis	Strauss and Corbin (1990)	Not reported	The study included 26 participants with moderate to severe TBI, and a total of 18 caregivers were recruited. Caregivers commonly reported receiving limited information regarding their relatives' injuries and management methods. Heavy caregiving demands were placed on them, with little support from the healthcare system. A significant proportion of caregivers experienced anxiety and depression, which was associated with lower functional independence in their injured relative. Somewhat more spouses than parents reported clinically significant anxiety levels. Other consequences of caregiving included social isolation and limited support from the wider community as well as financial difficulties. Despite these stresses caregivers tended to accept their caregiving role. Cultural factors such devotion to their families and faith and belief in God moderated bur-<br>den and distress.
Mbakile-Mahlanza, Manderson and Ponsford	Traumatology	2015	Australia	Psychologist (incl. clinical, neuro-)	School of Psychological Sciences	All HIC	Neuropsychological Rehabilitation	***The experience of traumatic brain injury in Botswana***	To explore the experiences of TBI in Botswana.	Adult	TBI	Botswana	Mixed methods (only qualitative reported)	Not reported	Patients AND Family members, carers, signifcant others AND HCPs	71	Interviews (open, unstructured, in-depth, semi-structured, open ended)	Grounded theory analysis/constant comparison/open, axial and/or selective coding	Corbin & Strauss (1990)	Not reported	Thematic analysis indicated several themes: Injury-related changes, attributions and beliefs about the cause of the injury, family reactions, attitudes, and resources. Participants described the common injury-related effects of TBI. Many participants attributed their injury to supernatural causes. Immediate family members of participants with TBI expressed a sense of love and devotion towards the injured person. Communication was characterized by inadequate information given to those injured and their caregivers. Provision of care was impeded by insufficient staff, limited supplies and lack of training of nurses. The current healthcare system would therefore appear to be ill equipped to meet the needs of TBI survivors in Botswana.
Mbakile-Mahlanza, Manderson and Ponsford	Traumatology	2017	Australia	Psychologist (incl. clinical, neuro-)	School of Psychological Sciences,	All HIC	Neuropsychological Rehabilitation	***Cultural beliefs about TBI in Botswana***	To understand and develop a framework for explaining the experiences of TBI sufferers and their families in Botswana.	Adult	TBI	Botswana	Mixed methods (Primarily driven by qualitative method)	Not reported	Patients AND Family members, carers, signifcant others AND HCPs	71	Mixed: Interviews (open, unstructured, in-depth, semi-structured, open ended) AND questionnaires	Grounded theory analysis/constant comparison/open, axial and/or selective coding	Corbin & Strauss (1990)	Not reported	Results indicated that participants considered TBI to be chronic in nature and accompanied by serious consequences. Participants held positive attitudes about the manageability of symptoms despite having little understanding about TBI and its consequences. People with TBI tended to report fewer symptoms than did their caregivers. In addition, although some participants held concrete beliefs about the causes of injury, many participants attributed the injury to supernatural causes. Religious interpretations were also commonly held. Although age appeared to be associated with beliefs, no significant relationships existed between demographic factors and beliefs about the injury.
Barkley, Spece, Barros, et al.	Traumatology	2021	USA	MD/Physician	Department of Neurological Surgery	Mixed HIC/LMIC	Journal of Neurosurgery	***A mixed-methods needs assessment of traumatic brain injury care in a low- And middle-income country setting: building neurocritical care capacity at two major hospitals in Cambodia***	To use both quantitative and qualitative assessment data to establish a comprehensive approach to inform capacity-development initiatives for TBI care at two hospitals in an LMIC, Cambodia.	Unclear	TBI	Cambodia	Mixed methods	Not reported	HCPs (inc. NSx) AND non-clinical providers	29	Mixed: Interviews (open, unstructured, in-depth, semi-structured, open ended) AND questionnaires	Thematic analysis (Theoretical)	Braun & Clarke (2006, 2013)	Not reported	Analysis of the qualitative data obtained from interviews revealed a need for continuing educational initiatives for staff, increased surgical and critical care supplies and equipment and infrastructure development. The analysis further elucidated barriers to care, such as challenges with time availability for experienced providers to educate incoming healthcare professionals, issues surrounding prehospital are, maintenance of donated supplies, and patient poverty.
Ahmadi, Reihaneh and Lim, Hajin and Mutlu, Bilge and Duff, Melissa and Toma, Catalina and Turkstra, Lyn	Traumatology	2022	Canada	Unclear/not stated	Department of Psychology, Neuroscience & Behaviour	All HIC	JMIR rehabilitation and assistive technologies	***Facebook Experiences of Users With Traumatic Brain Injury: A Think-Aloud Study***	to use the think-aloud method to characterize the ways people with TBI accessed and used social media websites, including challenges they faced.	Adult	TBI	CAN	Other (please state)	Meshi et al framework	Patients	8	Other: Think aloud method	Other: Open coding	Traum and Heeman (1996)	Not reported	The open coding analysis revealed 6 types of challenges reported by participants with TBI, including difficulty with language production<br>and interpretation, attention and information overload, perceptions of negativity and emotional contagion, insufficient guidance to use Facebook, concerns about web-based scams and frauds, and general accessibility concerns.
Grewal, J. and Citton, K. and Sing, G. and Biagioni, J. B. and Schmidt, J.	Traumatology	2024	Canada	Unclear/not stated	Rehabilitation Science Graduate Program,	All HIC	Plos One	***Priorities for quality of life after traumatic brain injury***	This study aims to understand the priorities for QOL after TBI using a group consensus building method.	Adult	TBI	CAN	Qualitative	Non stated	Patients	16	Mixed/multiple: Semi-structured questionnaires AND Focus groups	Thematic analysis	Braun and Clark (2006)	Not reported	Phase one included three expert participants who outlined the complexity and importance of QOL after TBI. Phase two included 34 participants with TBI who described broad priorities for QOL including social support, employment, and accessible environments. Phase three included 13 participants with TBI who identified seven priorities for QOL: ensuring basic needs are met, participating in everyday life, trusting a circle of care, being seen and accepted, finding meaning in relationships, giving back and advocating, and finding purpose and value. In phase four, four expert participants confirmed the QOL priorities.
Hendryckx, C. and Couture, M. and Gosselin, N. and Nalder, E. and Gagnon-Roy, M. and Thibault, G. and Bottari, C.	Traumatology	2024	Canada	Other: PhD candidate, neuropsychologist	Department of Psychology; Centre for Interdisciplinary Research in Rehabilitation; Institut universitaire sur la réadaptation en déficience physique; Center for Advanced Research in Sleep Medicine	All HIC	Neuropsychological Rehabilitation	***A window into the reality of families living long term with challenging behaviours after a TBI***	1) to identify the coping strategies used by people with TBI living in the community and their family caregivers to manage challenging behaviours; and 2) to describe the similarities and differences between strategies used by people with TBI and caregivers within dyads/triads	Adult	TBI	CAN	Qualitative descriptive	non stated	Patients AND Families/carers/ Significant other	26	Interviews (semi-structured, open, in depth)	Other: Inductive analysis	Miles et al. (2014)	COREQ	Participants' strategies were proactive (prevention), reactive (response), or retroactive (aftercare). Most strategies were described by caregivers. Some of them were effective and lasting, others not, reflecting how they adapted their approaches over time. Families put in place various strategies in their life's journey, such as giving feedback or adapting the environment.

Hendryckx, C. and Couture, M. and Gosselin, N. and Nalder, E. and Gagnon-Roy, M. and Thibault, G. and Bottari, C.	Traumatology	2024	Canada	Other; PhD candidate, neuropsychologist	Department of Psychology; Centre for Interdisciplinary Research in Rehabilitation; Institut universitaire sur la réadaptation en déficience physique; Center for Advanced Research in Sleep Medicine	All HIC	Neuropsychological Rehabilitation	***The dual reality of challenging behaviours: Overlapping and distinct perspectives of individuals with TBI and their caregivers***	This study aimed to (1) explore and confirm the perspective of individuals with TBI living in the community and their family caregivers on behaviours they consider challenging and, (2) identify overlapping or distinct views on challenging behaviours.	Adult	TBI	CAN	Qualitative descriptive	epistemological paradigm of pragmatic realism	Patients AND Families/carers/ Significant other	26	Interviews (semi-structured, open, in depth)	Other: inductive qualitative analysis	Miles et al. (2014)	COREQ	Challenging behaviours most frequently reported by all participants were aggressive/impulsive behaviours, inappropriate social behaviours, and behavioural manifestations of cognitive impairments. Overlapping perspectives were identified regarding aggressive behaviours. Distinctions exist as inappropriate social behaviours and cognitive difficulties were mainly reported by caregivers.
Hibi, A. and Bilbily, A. and Cusimano, M. D. and Krishnan, R. G. and Tyrrell, P. N.	Traumatology	2024	Canada	Unclear/not stated	Institute of Medical Science; Department of Medical Imaging; Division of Neurosurgery	All HIC	Canadian Journal of Neurological Sciences	***Impact of Automated Prognostication on Traumatic Brain Injury Care: A Focus Group Study***	(1) gain insight into what current healthcare providers' expect from TBI prognostication and current practices around prognostication, (2) ascertain stakeholder perspectives on the utilization of machine learning-based TBI prognostication, and (3) identify the existing gaps/barriers and facilitators to the implementation of machine learning-based TBI prognostication	N/A	TBI	CAN	Qualitative	Non stated	Mixed stakeholders	10	Focus groups	Thematic analysis	Chapman et al. (2015)	Not reported	The study captured diverse perceptions and interests in TBI prognostication across clinical specialties. Notably, certain clinicians who currently do not prognosticate expressed an interest in doing so independently provided they had access to ML support. Concerns included ML's accuracy and the need for proficient ML researchers in clinical settings. The consensus suggested using ML as a secondary consultation tool and promoting collaboration with internal or external research resources. Participants believed ML prognostication could enhance disposition planning and standardize care regardless of clinician expertise or injury severity. There was no evidence of perceived bias or interference during the discussions.
Hickling, Andrea and Mah, Katie and Al-Hakeem, Hiba and Scratch, Shannon E.	Traumatology	2023	Canada	Other: Occupational Therapist	Bloorview Research Institute; Department of Occupational Science and Occupational Therapy	All HIC	Journal of interprofessional care	***Exploring the experiences of youth with persistent post-concussion symptoms and their families with an interprofessional team-based assessment***	The objective of this qualitative descriptive study was to explore the experiences of youth with prolonged post-concussion symptoms (PPCS) and their parents who participated in an interdisciplinary team-based assessment (ITA) at a children's rehabilitation hospital in Ontario, Canada.	Paediatric	TBI	CAN	Qualitative descriptive	Non stated	Patients AND Families/carers/ Significant other	15	Interviews (semi-structured, open, in depth)	Content analysis	Hsieh and Shannon, 2005	Not reported	Results suggest that the ITA serves as a context for meaningful therapeutic interactions whereby youth, their parents, and the interprofessional team establish and build therapeutic relationships, engage in dialogue emphasizing collaboration, prioritize the young person rather than the injury, and co-create an individualized treatment plan.
Lian, L. and Coupland, R. and Tylinski Sant'ana, T. and Colantonio, A. and Mollayeva, T.	Traumatology	2024	Canada	Occupational therapist	Department of Occupational Sciences & Occupational Therapy	All HIC	Journal of Head Trauma Rehabilitation	***Community Integration Challenges of Men and Women After Traumatic Brain Injury: A Reflexive Thematic Analysis of Lived Experiences Through a Gender Lens***	To investigate community integration (CI) challenges following traumatic brain injury (TBI) through a gender lens.	Adult	Moderate-severe TBI	Canada	Qualitative	Interpretivist paradigm	Patients	42	Interviews (semi-structured, open, in depth)	Reflexive thematic analysis	Braun and Clarke (2021)	Not reported	Three overarching themes emerged: (1) lack of a "graduated home plan": difficulty enacting gendered roles and responsibilities at home, (2) "Something that I cannot handle": mismatch between occupational demands and abilities, and (3) "Slipping away": disrupted connections with self and others. Men consistently voiced struggles with and inability to fulfill household tasks, whereas women detailed ways of adapting domestic responsibilities because of postinjury limitations. Productivity challenges resonated with a substantial number of participants, and a gendered pattern was observed: More women reported an inability, whereas more men reported a limited ability to meet occupational demands. Both genders conveyed disruptions in recreational and leisure activities, although women predominantly discussed challenges relating to social activities and men a tendency to avoid social situations because of anticipated stigma and/or shame. Across all themes, a pervasive sense of diminished community contributions and disrupted identities was observed.
Omar, S. and Williams, C. C. and Bugg, L. B. and Colantonio, A.	Traumatology	2024	Canada	Unclear/not stated	Rehabilitation Sciences Institute	All HIC	Bmc Health Services Research	***"Somewhere along the line, your mask isn't going to be fitting right": institutional racism in Black narratives of traumatic brain injury rehabilitation across the practice continuum***	to examine the rehabilitation narratives of Black TBI survivors, family caregivers, and rehabilitation providers and use critical race theory as a conceptual framework to understand how anti-Black racism manifests in those experiences.	Adult	TBI	Canada	Narrative inquiry	Critical constructivism and critical race theory	Families/carers/ Significant other AND HCPs	14	Interviews (semi-structured, open, in depth)	Reflexive thematic analysis	Braun and Clarke (2006), Braun and Clarke (2019), Braun and Clarke (2021)	COREQ	Themes captured how racism becomes institutionalized in TBI rehabilitation: (1) the institutional construction of deficient Black bodies, (2) the institutional construction of rehabilitation access, (3) the institutional investment in resisting and approximating whiteness in rehabilitation practice, and (4) the institutional construction of deficient Black futures.
Purther, H. and Nitert-Brown, C. and Ferdosi, D. V. and Ho, E. S.	Traumatology	2023	Canada	Unclear/not stated	Department of Occupational Science and Occupational Therapy	All HIC	Hand Therapy	***Intersection of physical and mental health of youth with brachial plexus birth injuries: A qualitative study***	To understand the perspectives of youth with BPBI and their caregivers on the barriers and facilitators of addressing mental health concerns, to inform practice guidelines, and promote meaningful participation within this population.	Mixed	Brachial plexus injury	Canada	Qualitative	Social constructionist / interpretivist	Patients AND Families/carers/ Significant other	17	Interviews (semi-structured, open, in depth)	Reflexive thematic analysis	Braun (2008), Braun and Clarke (2021)	Not reported	A purposeful sample of nine youth with BPBI between 10 to 20 years and eight caregivers participated in in-depth interviews. The interviews were semi-structured and an average of 60 min (35–85 min) long. Three themes emerged from these data: (i) physical disability identity and mental health; (ii) pursuit of "normal" body image; and (iii) paradox of advocacy. Findings illuminated the intersection of physical and mental health in these youth and provides actionable practice recommendations. Areas of need were identified including mental health support around the challenges of advocacy, body image, surgery, and preparation for the 'aging out' process for youth and their families.
Pye, A. and Stanton, S. and Bristol, S. and Chapman, K. M. and Berger, M. J.	Traumatology	2023	Canada	Medical student	Faculty of Medicine	All HIC	Plastic Surgery	***Patient Perspectives on Interdisciplinary Peripheral Nerve Trauma Care***	to assess patients' perceptions of the effectiveness of delivery of care and health care information in an interdisciplinary PNI clinic.	Adult	Brachial plexus injury	Canada	Mixed methods,	Not explicitly stated	Patients	20	Mixed/multiple (please state)	Content analysis	Hsieh and Shannon (2005)	Not reported	Of the 20 participants, 65% were male, 35% were female and the mean age was 42.6 ± 17.8. Median scores of 4 were obtained for the patient understanding of the testing purposes, test results, nerve recovery after PNI, and surgical decision-making (full understanding = 5). On improving the clinical experience, 58% indicated no improvements were necessary, while 17% indicated the clinic felt rushed or overwhelming. When asked about positive aspects of their clinical experience, 64% appreciated the team approach to care, 27% valued the informative nature of the clinic, and 27% appreciated the progress they felt when providers at the clinic performed nerve testing.

Shepherd, H. A. and Heming, E. and Reed, N. and Caron, J. G. and Yeates, K. O. and Emery, C. A.	Traumatology	2024	Canada	Occupational therapist	Department of Occupational Science and Occupational Therapy	All HIC	Journal of School Health	***Describing High School Stakeholders' Preferences for a Return-to-School Framework Following Concussion***	To describe the preferences of high school students, parents, and educators for a Return-to-School Framework for adolescents following a concussion.	Adolescents	mTBI	Canada	Qualitative	Pragmatism	Mixed: Patients, parents, educators	26	Interviews (semi-structured, open, in depth)	Content analysis	Hsieh and Shannon (2005)	Not reported	We organized the data into 4 main themes: (1) purpose of the Return-to-School Framework; (2) format and operation of the Return-to-School Framework; (3) communication about a student's concussion; and (4) necessity of concussion education for students and educators. A Return-to-School Framework following concussion should be developed in consultation with families, educators, and students and supports should be tailored to each student.
Shepherd, Heather A. and Kolstad, Ashley T. and Caron, Jeffrey G. and Reed, Nick and Yeates, Keith Owen and Schneider, Kathryn J. and Black, Amanda M. and Emery, Carolyn A.	Traumatology	2024	Canada	Occupational therapist	Sport Injury Prevention Research Centre, Faculty of Kinesiology; Alberta Children's Hospital Research Institute; Hotchkiss Brain Institute; O'Brien Institute for Public Health	All HIC	Neuropsychological rehabilitation	***Unravelling the web: Experiences of adolescents returning to school following a concussion***	To explore the perspectives of 20 adolescents (ages 14-18) returning to school after a concussion.	Adolescents	mTBI	Canada	Qualitative	Interpretive	Patients	20	Interviews (semi-structured, open, in depth)	Reflexive thematic analysis	Braun and Clarke (2019)	Not reported	Five interconnected themes emerged with returning to school and accessing school supports: (1) concussion symptoms affected adolescents' schoolwork; (2) access to academic accommodations eased adolescents' return to school; (3) having supportive and understanding friends, family, and teachers facilitated adolescents' return to school; (4) communication amongst school stakeholders was desired, but often lacking; and (5) feeling anxious, frustrated, and sad with the return to school process. Adolescents' experiences were multifaceted and many factors contributed to their return to school experiences. Our findings can inform our understanding of the experiences of adolescents returning to school following concussion and can inform the development of concussion management supports at schools.
Souesme, G. and Poulin, V. and Ethier, A. and Grenier, M. and Sirois, M. J. and Beaulieu-Bonneau, S. and De Guise, E. and Lamontagne, M. E. and Hudon, C. and Emond, M. and Ouellet, M. C.	Traumatology	2024	Canada	Unclear/not stated	Centre interdisciplinaire de recherche en readaptation et integration sociale (Cirris), Institut de readaptation en deficience physique	All HIC	Rehabilitation Psychology	***Challenges and Facilitators in the Experience of Caregiving for an Older Adult With Traumatic Brain Injury: A Longitudinal Qualitative Study in the First-Year Postinjury***	: To obtain a better understanding of the factors which complicate or facilitate the adjustment of caregivers after traumatic brain injury (TBI) in older adults. R	Adult	TBI	Canada	Qualitative	Not explicitly stated	Family/Carer/Significant other	65	Interviews (semi-structured, open, in depth)	Thematic analysis	Braun and Clarke (2013)	COREQ	Participants mentioned almost as many facilitators as challenges at each time point. Among the facilitators, we found the following themes: receiving social support, having access to rehabilitation, improvement of the injured loved one's health condition, returning to live at home, having access to home services, feeling useful, effective communication, and having time for oneself. The challenges identified were: health issues in the injured loved one, psychological impact on the caregiver, assuming a new role, relationship strain, and decrease in activities and outings.
Toccalino, D. and Haag, H. and Nalder, E. and Chan, V. and Moore, A. and Colantonio, A. and Wickens, C. M.	Traumatology	2024	Canada	Doctoral student	Institute of Health Policy, Management and Evaluation; Acquired Brain Injury Research Lab	All HIC	Plos One	***"A whole ball of all-togetherness": The interwoven experiences of intimate partner violence, brain injury, and mental health***	to explore the BI- and mental health-related needs and experiences of IPV survivors from the perspectives of survivors and service providers with the objective of developing knowledge translation materials to raise awareness and support survivors and service providers in addressing these concerns.	Adult	Brain injury	Canada	Qualitative descriptive	Interpretive	Patients AND clinicians	24	Mixed: Interviews and focus groups	Thematic analysis	Braun and Clarke (2006, 2021)	Not reported	Across interviews, participants spoke about IPV, BI, and mental health as being complex and interrelated experiences that have impacts across the survivor's life and extend well beyond the abusive relationship. Because of the underrecognized nature of BI in IPV, finding and accessing care requires persistence that survivors spoke of as being like "a full-time job." The benefit of making meaningful connections, particularly with other survivors, was highlighted.
van Ierssel, Jacqueline and O'Neil, Jennifer and King, Judy and Zemek, Roger and Sveistrup, Heidi	Traumatology	2023	Canada	Physiotherapist	Children's Hospital of Eastern Ontario Research Institute	All HIC	The Journal of head trauma rehabilitation	***Clinician Perspectives on Providing Concussion Assessment and Management via Telehealth: A Mixed-Methods Study***	To examine clinician perspectives regarding the use of telehealth for concussion assessment and management.	Mixed	Mild TBI	Canada	Mixed methods (explanatory sequential)	Not explicitly stated	HCPs (Inc NSx)	25	Mixed: Survey and focus groups	Thematic analysis	Braun and Clark (2006)	Not reported	Clinicians strongly agreed that telehealth could be utilized to obtain a clinical history (96%), assess mental status (88%), and convey a diagnosis (83%) on initial assessment; to take a focused clinical history (80%); to monitor functional status (80%) on follow-up; and to manage symptoms using education on rest (92%), planning and pacing (92%), and sleep recommendations (91%); and to refer to a specialist (80%). Conversely, many clinicians believed telehealth was unsuitable to perform a complete neurologic examination (48%), cervical spine (38%) or vestibular assessment (61%), or to provide vestibular therapy (21%) or vision therapy (13%). Key benefits included convenience, provision of care, and patient-centered approach. General and concussion-specific challenges included technology, quality of care, patient and clinician characteristics, and logistics. Strategies to overcome identified challenges are presented.
Acorn and Roberts	Traumatology	1992	Canada	RN/Nurse	Assistant Professor, School of nursing	All HIC	The Journal of neuroscience nursing	***Head injury: impact on the wives***	The impact of the head injury on the wife of a survivor	Unclear	TBI	Canada	Survey	Role theory	Family members, carers, signifcant others	12	Open ended questions	Content analysis (latent)	Field & Morse (1985)	Not reported	Content analysis was used to analyze data with the themes of role changes, emotional impact of the injury, hope and need for support emerging. Implications for practice and research include need for support groups with nurses as facilitators, the importance of fostering hope, and need for education of health care professionals.
Campbell, Plourde, Hartling, et al.	Traumatology	2022	Canada	RN/Nurse	Faculty of Nursing	All HIC	Journal for specialists in pediatric nursing : JSPN	***"You Can't Fix Your Brain": Exploring concussion experiences of children and parents***	To explore the experiences, information needs and preferences of children who have had a concussion and their parents who have cared for them.	Pediatric	TBI (concussion)	Canada	Qualitative (descriptive)	Not reported	Patients AND Family members, carers, signifcant others	25	Interviews (open, unstructured, in-depth, semi-structured, open ended)	Thematic analysis (inductive)	Braun & Clarke (2006)	COREQ	Four major themes were identified: (1) mechanism of injury and concussion symptoms experienced by children, (2) parent concerns, emotions, and health care experience with child's concussion, (3) concussions affect more than just your head and, (4) health information seeking, and preferences of parents and children related to concussion. Children and their parents have unique experiences, information needs and preferences regarding concussion.
Chandler, Sun and Racine	Traumatology	2017	Canada	Not reported	Faculty of Law	N/A	AJOB Empirical Bioethics	***Online public reactions to fMRI communication with patients with disorders of consciousness: Quality of life, end-of-life decision making, and concerns with misdiagnosis***	Recently, the news media have reported on the discovery of covert awareness and the establishment of limited communication using a functional magnetic resonance imaging (fMRI) neuroimaging technique with several brain-injured patients thought to have been in a vegetative state. This discovery has raised many ethical, legal, and social questions related to quality of life, end-of-life decision making, diagnostic and prognostic accuracy in disorders of consciousness, resource allocation, and other issues. This project inquires into the public responses to these discoveries.	Unclear	Traumatic brain injury ("brain injured" unclear if TBI only)	Canada	Qualitative	Not reported	Public/Lay	4	Online articles or blog posts	Thematic analysis	Not reported	Not reported	Among the most frequent public reactions revealed in the online comments were discussions of the quality of life of patients with disorders of consciousness, whether life-sustaining treatment should be withdrawn (and whether the fMRI communication technique should be used to ask patients about this), and misgivings about the accuracy of diagnosis in disorders of consciousness and brain death.

Choudhury, Kolstad, Prajapati, et al.	Traumatology	2020	Canada	Not reported	Patient and Community Engagement Research Program, O'Brien Institute of Public Health, Cumming School of Medicine	All HIC	Health expectations	***Loss and recovery after concussion: Adolescent patients give voice to their concussion experience***	To understand the experiences of recovery from the perspective of adolescent patients of concussion and to present the findings through their voices	Adolescents	TBI (concussion)	Canada	Qualitative	Not reported	Patients	7	Mixed: Interviews (narrative) AND focus groups	Grounded theory analysis/constant comparison/open and axial/selective coding AND constructivist narrative analysis	Glaser (1992), Glaser & Strauss (1967), Riessman, (1993)	Not reported	Participants experience continuing difficulty 1-5Â years after treatment with cognitive, emotional, social and mental well-being. The overriding experience among older adolescents (17-20) is a sense of irreversibility of the impact of concussion in all these areas.
Cusimano, Korman, Carpino, et al.	Traumatology	2021	Canada	Neurosurgeon	Division of Neurosurgery, St. Michael's Hospital, Toronto	All HIC	Neurotrauma reports	***The Temporal Relations of Traumatic Brain Injury, Victimization, Aggression, and Homelessness: A Developmental Trajectory***	To understand the temporal occurrences of events over the life course that contribute to vulnerabilities to TBI, victimization, aggression, and homelessness.	Adult	TBI	Canada	Qualitative	Not reported	Patients	33	Interviews (open, unstructured, in-depth, semi-structured, open ended)	Grounded theory analysis/constant comparison/open, axial and/or selective coding (Thematic)	Glasser & Strauss (1967); Strauss & Corbin (1990); Denzin & Lincoln (19940; Boeije (2002).	Not reported	Twenty-five of 33 (76%) participants had a self-reported history of TBI. Seventy-six percent of TBI events occurred before the onset of homelessness. Assault was the most common mechanism of TBI. During childhood, TBI was a frequently reported event, and parent- or guardian-related physical and sexual abuse were also accentuated with peer abuse, which may have contributed to a unique developmental trajectory. Aggressive behaviors were reported more commonly in persons who previously endured physical, sexual, and emotional victimization early in childhood.
Cusimano, Korman, Kazolis, et al.	Traumatology	2021	Canada	MD/Physician	Division of Neurosurgery, St. Michael's Hospital, Toronto	All HIC	Neurotrauma reports	***Stumblers and Tumblers: Two Pathways to "Unintentional" Fall-Related Traumatic Brain Injury***	To examine whether life events and psychosocial antecedents, such as early adverse childhood experiences, play a role in the occurrence of non-intentional fall-related TBI.	Adult	TBI	Canada	Qualitative	Not reported	Patients	27	Interviews (open, unstructured, in-depth, semi-structured, open ended)	Grounded theory analysis/constant comparison/open and axial/selective coding	Strauss & Corbin (1990); Glaser & Strauss 1967; Charmaz 2006	Not reported	The results reveal that childhood family conflict and peer-influenced risky behaviors may have contributed to poorer mental and physical health in adulthood, which in turn contributed to injuries. Respondents whose behaviors did not play a direct role in their injury event were labeled "Stumblers." These patients' falls were seen as being related to unfortunate unique environmental and situational factors and could colloquially be described as "accidental falls." We also identified a distinct group of patients who had a cumulative life experience starting in early childhood that contributed to a pattern of riskier behaviors, ultimately culminating in a fall-related TBI. The second group of patients were labeled "Tumblers" as they chose to participate in risky activities, regardless of whether they considered them to be risky, which ultimately led to the fall-related TBI. This group was identified by a purposeful volitional state that sought out the "opportunity for accidental fall." Childhood family conflict and peer-influenced risky behaviors were important precursors to mental and physical health states in this group.
Cusimano, Topolovec-Vranic, Zhang, et al.	Traumatology	2017	Canada	MD/Physician	Division of Neurosurgery, St. Michael's Hospital, Toronto	All HIC	Clinical journal of sport medicine : official journal of the Canadian Academy of Sport Medicine	***Factors Influencing the Underreporting of Concussion in Sports: A Qualitative Study of Minor Hockey Participants***	To identify factors contributing to underreporting of concussion in adolescent athletes.	Adolescents	TBI (concussion)	Canada	Qualitative	Constructivist-interpretive paradigm	Other: Patients AND comparison group	61	Interviews (open, unstructured, in-depth, semi-structured, open ended)	Thematic analysis	Charmaz (2006); Denzin & Lincoln 2011	Not reported	Aspects of hockey culture such as an overemphasis on winning games and upheld misperceptions about the risks associated with concussion were identified as relevant to the underreporting of concussions. Various factors relevant to the underreporting of concussions include player's motivation to win, group membership dynamics such as a player's role as the team's "enforcer," coaches' own motivation to win to further their own opportunities in the sport, and parents' personal financial interest or alternative agenda in terms of time commitments and their child's future career prospects.
Davis, Gemeinhardt, Gan, et al.	Traumatology	2003	Canada	Not reported	West Park Healthcare Center	All HIC	Brain Injury	***Crisis and its assessment after brain injury***	To develop a measure to assess crisis after acquired brain injury.	Adult	Mixed ABI	Canada	Mixed methods (Questionnaire; Qualitative, triangulation)	Not reported	Patients AND Family members, carers, signifcant others AND HCPs	60	Focus groups	Content analysis	Strauss,(1987)	Not reported	The six themes derived from the content analysis led to the creation of the measure, with versions for individuals who have an ABI, family members and professionals. Test-retest reliability results (n = 40) were adequate.
D'Souza, Fabricius, Amodio, et al.	Traumatology	2022	Canada	Not reported	Rehabilitation Sciences Institute, Acquire Brain injury and Society Laboratory, Acquired Brain Injury Research Laboratory	All HIC	Neuropsychological Rehabilitation	***Men's gendered experiences of rehabilitation and recovery following traumatic brain injury: A reflexive thematic analysis***	To explore how gender is related to men's post-TBI perceptions and behaviors in rehabilitation and recovery.	Adult	TBI	Canada	Qualitative	Interpretivist paradigm/ Connell's (2005) theory of hegemonic masculinity.	Patients	22	Interviews (open, unstructured, in-depth, semi-structured, open ended)	Reflexive thematic analysis	Braun & Clarke (2006, 2013)	15-point checklist criteria provided by Braun and Clarke	Three key themes were identified: (1) 'I'm a man, I'm a rock': Undermining treatment, (2) 'I'm going to face that challenge': Facilitation of recovery, and (3) 'I don't feel as useful as a guy as I was before': Perceptions on return to work.
Dubuc, Gagnon-Roy, Couture, et al.	Traumatology	2019	Canada	Not reported	School of Rehabilitation	All HIC	Australian Occupational Therapy Journal	***Perceived needs and difficulties in meal preparation of people living with traumatic brain injury in a chronic phase: Supporting long-term services and interventions***	To examine the perceived needs and difficulties regarding meal preparation in individuals with severe TBI living in the community. This is done in an effort to justify long-term community-based interventions offered to the TBI population with regard to a task involving many safety issues.	Mixed	TBI	Canada	Qualitative (descriptive)	Not reported	Patients	5	Interviews (open, unstructured, in-depth, semi-structured, open ended)	Other: Inductive analysis	Miles, Huberman, and Saldana (2013),	Not reported	Participants living alone were all involved in meal preparation to diverse levels. Only two participants lived with a family member. Six categories of perceived needs were identified, of which two emerged as priorities: (i) Need for recipes to be compatible with cognitive abilities, knowledge and energy level to optimize motivation and (ii) Need to adapt complexity of grocery shopping to cognitive abilities and knowledge. The main difficulty expressed by participants was to manage their motivation to cook when tired, as it tends to diminish and fade when the person is exhausted.

Duff	Traumatology	2006	Canada	RN/Nurse	School of Nursing	N/A	Axone	***Family impact and influence following severe traumatic brain injury***	To present findings concerning families from a prospective study using grounded theory methodology that was conducted on two acute care neurosurgical units in Toronto.	Adult	TBI	Canada	Grounded theory	Not reported	Family members, carers, signifcant others	25	Mixed: Interviews (open, unstructured, in-depth, semi-structured, open ended) AND clinical conversations AND observations	Other (please state): Reports using grounded theory methodology but no grounded theory analysis described	Not reported	Not reported	Negotiating is the core variable or central concern of families who have a close relative in a state of post-comatose unawareness or minimal responsiveness following a severe traumatic brain injury. Family members negotiate with each other, with members of the health care team, and with others such as insurance agents, case managers, and representatives of the justice system on behalf of the injured individual. Family members also negotiate the intricacies of the health care system.
Duff	Traumatology	2002	Canada	RN/Nurse	Centennial College	N/A	Axone	***Codman Award paper. Family concerns and responses following a severe traumatic brain injury: a grounded theory study***	The focus of this paper is to present an inductively derived substantive theory, "Negotiating Uncertainty". The theory details the most salient concerns of families who had a close relative who had suffered a severe TBI and how they dealt with the experience during the uncertain trajectory of illness that followed coma.	Adult	TBI	Canada	Grounded theory	Not reported	Patients AND Family members, carers, signifcant others	36	Mixed: Interviews (open, unstructured, in-depth, semi-structured, open ended) AND clinical conversations AND observations	Grounded theory analysis/constant comparison/open and axial/selective coding	Not reported	Not reported	"Negotiating Uncertainty" has 4 steps: Willing Survival, Attending to Snow White, Reconstructing the Person, and Making it Better
Fabricius, D'Souza, Amodio, et al.	Traumatology	2020	Canada	Not reported	Acquired Brain Injury & Society Research Lab, Toronto Rehabilitation Institute	All HIC	Qualitative health research	***Women's Gendered Experiences of Traumatic Brain Injury***	To address the gaps noted above by answering the question, "How does gender influence women's experiences of TBI?"	Adult	TBI	Canada	Qualitative	Not reported	Patients	19	Interviews (open, unstructured, in-depth, semi-structured, open ended)	Thematic analysis	Braun & Clarke (2006, 2013)	Not reported	Three themes were constructed: Gender prevails considers choosing to do gender over complying with physician advice; Consequences of TBI impeding performativity explores how women frame themselves as terrible people for being unable to do gender post-TBI; and Perceptions of receiving care looks at gendered caregiving expectations. These results broadly align with research on how doing gender influences recovery and health outcomes
Gagnon, Lin and Stergiou-Kita	Traumatology	2016	Canada	Not reported	Department of Occupational Science and Occupational Therapy	All HIC	Disability and Rehabilitation	***Family members facilitating community re-integration and return to productivity following traumatic brain injury-motivations, roles and challenges***	To explore the experiences of family members in supporting community re-integration and return to productive occupations of the traumatic brain injury (TBI) survivor in order to: (i) describe family members' supportive roles, (ii) determine challenges family members experience in supporting the TBI survivor; and (iii) identify supports that family members require to maintain and enhance their roles.	Unclear	TBI	Canada	Qualitative (descriptive)	Not reported	Family members, carers, signifcant others	14	Interviews (open, unstructured, in-depth, semi-structured, open ended)	Thematic analysis	Braun & Clarke (2006)	Not reported	Family members expressed strong motivation and engaged in six key roles to support TBI survivors: researcher, case manager, advocate, coach, activities of daily living (ADL)/instrumental ADLs and emotional supporter. Personal and family stressors and challenges navigating the health care system were perceived as challenges in meeting demands of their supportive roles. Stigma also presented a barrier to successful community and vocational re-integration. Subsequently, family members desired more education related to the functional implications of TBI, to be connected to health care and community resources, and sought a greater family-Centered care approach.
Gagnon, Swaine, Champagne, et al.	Traumatology	2008	Canada	Not reported	Faculté de médecine, Département d'administration de la santé, Université de Montréal	All HIC	Brain Injury	***Perspectives of adolescents and their parents regarding service needs following a mild traumatic brain injury***	To explore the specific service needs of adolescents (12-18 years) after a mTBI.	Adolescents	TBI	Canada	Phenomenology	Not reported	Patients AND Family members, carers, signifcant others	30	Interviews (open, unstructured, in-depth, semi-structured, open ended)	Phenomenological analysis	Corbin & Strauss (2008)	Not reported	All adolescents and parents expressed the need to receive information about the injury, its expected recovery and when to return to activities. Many adolescents reported wanting to be seen rapidly, by professionals who genuinely care about them and who acknowledge that they have specific needs that differ from those of younger children. Parents and, to a lesser degree, adolescents think that enhanced communication between the healthcare and school systems would be beneficial following a mTBI to assist in returning to demanding academic activities.
Gauvin-Lepage and Lefebvre	Traumatology	2010	Canada	RN/Nurse	Faculty of Nursing	All HIC	Brain Injury	***Social inclusion of persons with moderate head injuries: The points of view of adolescents with brain injuries, their parents and professionals***	To explore the perceptions of adolescents, their parents and professionals as to the social inclusion of adolescents who have suffered a moderate traumatic brain injury (TBI).	Adolescents	TBI	Canada	Qualitative descriptive (Ecological)	Constructivist	Patients AND Family members, carers, signifcant other AND HCPs AND Non-clinical providers	11	Mixed: Interviews (open, unstructured, in-depth, semi-structured, open ended) AND focus groups	Content analysis	Patton (2002)	Not reported	The results show that the perceptions of adolescents, as well as their parents, affect different aspects of their lives, such as the adolescent's personal experiences, the family, friends, the environment and school. A great number of repercussions were indeed noted, which facilitate and sometimes limit the social inclusion of these adolescents. In general, the professionals shared the same perceptions, but added some ideas that did not come up in interviews with the adolescents and their parents.
Gendreau and De La Sablonnière	Traumatology	2014	Canada	Not reported	Department of Psychology	All HIC	Disability and Rehabilitation	***The cognitive process of identity reconstruction after the onset of a neurological disability***	To explore the cognitive process by which personal identity is reconstructed following disability onset.	Adult	TBI; SCI	Canada	Qualitative	Postpostivism + Cognitive-Developmental Model of Social Identity Integration	Patients	10	Interviews (open, unstructured, in-depth, semi-structured, open ended)	Thematic analysis	Braun & Clarke (2006)	Not reported	As suggested by the CDMSII, following the accident, participants initially tend to emphasize distinctions between their pre- and post-injury conditions. Eventually, individuals are able to create cognitive connections between pre-injury self-knowledge and how they understand their new condition. Finally, in the last stage of the identity integration process, the various identity components are recognized as part of the self. Organizing data based on the three stages of this theory was found to resonate with participants and aided the comprehension of how every stage in the identity reconstruction process is intertwined with the need for continuity through life.
Ghandour, Hould, Fortier, et al.	Traumatology	2020	Canada	Not reported	Institut National d'excellence en Santé et en Services Sociaux (INESSS) and Center Intégré de Santé et de Services Sociaux de Chaudière-Appalaches	All HIC	Patient	***Adapting two American Decision Aids for Mild Traumatic Brain Injury to the Canadian Context Using the Nominal Group Technique***	To create a consensus among Canadian mild traumatic brain injury and emergency medicine experts on modifications required to adapt two American decision aids about head CT use for adult and Pediatric mild traumatic brain injury to the Canadian context.	Mixed	TBI (concussion)	Canada	Qualitative	Not reported	Other: Mixed stakeholders	21	Other: Consensus meeting - 'Nominal Group Technique'	Thematic analysis	Braun & Clarke (2006)	SRQR	Participants highlighted the need to clarify the purpose of the decision aids, the nature of the problem being addressed and the target population. The tools require sociocultural adaptations, better identification of their target population, better description of head CT utility, advantages and related risks, modification of the visual and written representation of the risk of brain injury and head CT use, and locally adapted, patient follow-up plans.

Hamour, Mendez, Biron, et al.	Traumatology	2019	Canada	Not reported	Department of Otolaryngology-Head and Neck Surgery	All HIC	Clinical Otolaryngology	***Development of the Alberta facial clinical evaluation scale: A patient-centered outcomes instrument for facial nerve paralysis***	To identify patient domains of concern and subsequently, develop a point-of-care questionnaire for clinical use.	Adult	Nerve; Facial nerve injury	Canada	Mixed methods (Questionnaires ; Grounded theory)	Not reported	Patients	20	Mixed: Interviews (open, unstructured, in-depth, semi-structured, open ended) AND focus groups AND questionnaires	Grounded theory analysis/constant comparison/open, axial and/or selective coding	Not reported	Not reported	Patients identified a total of 16 themes encompassing both functional and psychological deficits related to their facial nerve injury. From these findings, a 25-item Likert-type scale, the A-FaCE scale, was developed for clinical use.
Hunt, De Saint-Rome, Di Salle, et al.	Traumatology	2020	Canada	Not reported	Head Injury Clinic, Trauma and Neurosurgery Program	All HIC	Canadian Journal of Neurological Sciences	***Mapping Stakeholder Perspectives on Engagement in Concussion Research to Theory***	To identify potential benefits, challenges, and motivators to engaging in research by gathering the perspectives of adults with lived experience of concussion.	Unclear	TBI (concussion)	Canada	Survey	Not reported	Patients AND Family members, carers, signifcant others AND HCPs AND researchers	60	Open ended questions	Grounded theory analysis/constant comparison/open, axial and/or selective coding (Thematic)	Corbin & Strauss (2008)	Not reported	Four themes regarding benefits to engagement emerged: first-hand account, meaningful recovery, research relevance, and better understanding of gaps. Three forces inhibited engagement: environmental barriers, injury-related constraints, and personal deterrents. Four enablers supported engagement: focus on positive impact, build connections, create a supportive environment, and provide financial assistance.
Karpman, Wolfe and Vargo	Traumatology	1986	Canada	Psychologist (incl. clinical, neuro-)	University of Alberta	All HIC	Journal of Applied Rehabilitation Counseling	***The psychological adjustment of adult clients & their parents following closed-head injury***	To explore the process of the psychological adjustment of 10 head-injured Ss, aged 17–24 yrs, and their parents.	Mixed	TBI	Canada	Qualitative	Not reported	Patients AND Family members, carers, signifcant others	20	Interviews (open, unstructured, in-depth, semi-structured, open ended)	Other: Analyzed for common themes and ideas related to coping and adjustment	Colaizzi (1978); Collier & Kuiken (1976); Vargo (1984)	Not reported	Descriptive analysis of semi structured interviews revealed 15 prominent patient issues, including memory loss, social isolation, and uncertainty about the future. Among 12 issues of concern to parents were overprotectiveness, financial problems, and emotional strain. Most of the Ss were still dependent on family members 2–5 yrs following injury, indicating the importance of early intervention in helping patients to resume an active life.
Keenan and Joseph	Traumatology	2010	Canada	RN/Nurse	Trauma Program at The Ottawa Hospital	All HIC	Canadian journal of neuroscience nursing	***The needs of family members of severe traumatic brain injured patients during critical and acute care: a qualitative study***	To identify the needs expressed by family members, as patients with severe brain injury progress through their recovery	Adult	TBI	Canada	Qualitative	Not reported	Family members, carers, signifcant others	25	Interviews (open, in-depth, semi-structured, open ended)	Thematic analysis	Braun & Clarke (2006)	Not reported	Family members identified a variety of needs during the acute hospitalization period. Thematic analysis at Time 1 identified four main themes that described the trajectory of the families' experiences: getting the news, uncertainty, making sense of the news and moving on. At Time 2, themes of the family experience included uncertainty, looking for progress, transition and letting go/building a new connection. Themes that identified the needs of families included managing life, involvement in care, and holding on to hope. Support required by the family included the need for information, professional support and community support. Families had intensive needs in the acute phase of the injury and their needs changed over time
Kita, Mallory, Hickling, et al.	Traumatology	2020	Canada	Not reported	Concussion Center, Bloorview Research Institute	All HIC	Brain injury	***Social support during youth concussion recovery***	To enhance our understanding of meaningful social support from youth perspectives and inform the development of recommendations for the provision of social support following a concussion.	Adolescents	TBI (concussion)	Canada	Qualitative (descriptive)	Not reported	Patients	10	Interviews (open, unstructured, in-depth, semi-structured, open ended)	Thematic analysis	Braun & Clarke (2006)	Not reported	Close friends, youth with personal history of concussion, and parents were identified as key providers of meaningful social support during concussion recovery. Participants identified specific examples of support provided by each group. Close friends built a sense of social inclusion that mitigated feelings of social isolation. Youth with a personal history of concussion used their lived experiences to communicate empathy and validate the participant's challenges. Parents assisted with practical challenges (e.g. accessing accommodations) by leveraging their "adult power".<br>Participants identified that lack of understanding of their lived experiences was a key barrier to receiving support. They proposed solutions focused on education initiatives highlighting personal accounts from youth with concussion, and specific examples of how peers can help.
Lefebvre and Levert	Traumatology	2012	Canada	Not reported	Faculty of Nursing	All HIC	Brain Injury	***The close relatives of people who have had a traumatic brain injury and their special needs***	To paint a picture of the needs of people close to individuals with a TBI and the services offered to answer these needs, from the point of view of the individuals with a TBI and health professionals.	Adult	TBI	Canada	Qualitative	Reflective Practice through Partnership	Family members, carers, signifcant others AND HCPs	48	Focus groups	Content analysis: Topical	Mayer & Deslauriers (2000)	Not reported	The results show that people close to individuals with a TBI need information on the health problem, specifically with regard to the diagnostic, the prognostic, and the factors that influence it, as well as the steps towards rehabilitation, and care and services. The results show that close ones need specific, quality services and continuity of services.
Lefebvre, Pelchat, Swaine, et al.	Traumatology	2005	Canada	Not reported	Montreal Center for Interdisciplinary Research on Rehabilitation	All HIC	Brain Injury	***The experiences of individuals with a traumatic brain injury, families, physicians and health professionals regarding care provided throughout the continuum***	To investigate the experiences of individuals who had sustained a traumatic brain injury, their families and the physicians and health professionals involved, from the critical care episodes and subsequent rehabilitation.	Adult	TBI	Canada	Qualitative	Not reported	Patients AND Family members, carers, signifcant others AND HCPs (inc. NSx) AND non-clinical providers	47	Interviews (open, unstructured, in-depth, semi-structured, open ended)	Not clearly stated	Merton et al. (1990)	Not reported	Results revealed the difficulties encountered by the different people involved, from the standpoint of the readjustment of the individual with the TBI and their family, the relationships among the various actors and the continuity of care.
Lefebvre and Levert	Traumatology	2012	Canada	Not reported	Faculty of Nursing,	All HIC	Journal of trauma nursing : the official journal of the Society of Trauma Nurses	***The needs experienced by individuals and their loved ones following a traumatic brain injury***	To explore the needs of individuals and their loved ones throughout the continuum of care and services, from the point of view of everyone affected by the experience of a TBI, including individuals, their loved ones, and the health care professionals involved in their care.	Adult	TBI	Canada	Qualitative	Not reported	Patients AND Family members, carers, signifcant others AND clinicians (HCPs)	150	Focus groups	Thematic: content analysis	Paterson et al. (2001)	Not reported	Despite regional differences, the results demonstrate participants' very similar perceptions regarding the needs such as information, support, and a collaborative relationship with health care professionals experienced by individuals with TBIs and their loved ones. These needs change throughout the stages of care. The fulfillment of these needs play a determining role throughout the adaptation process of individuals with TBIs and their loved ones. Health care professionals must adopt a personalized approach to respond to needs related to the evolution of information, support, and relationships.

Mollayeva, Bordignon, Ishtiaq, et al.	Traumatology	2021	Canada	Not reported	KITE-Toronto Rehabilitation Research Institute, University Health Network	All HIC	Disability and Rehabilitation	***Knowledge of sex and gender and related information needs in patients with traumatic brain injury: in-depth interview study***	To gain an understanding of biological sex and social gender phenomena experienced by patients with traumatic brain injury in recovery, and to understand the educational needs of this group.	Adult	TBI	Canada	Qualitative (descriptive)	Not reported	Patients	40	Interviews (open, unstructured, in-depth, semi-structured, open ended)	Content analysis - Thematic	Creswell (2007), Bernard & Ryan (2010)	COREQ	Three overarching themes that limited patients' knowledge and subsequent efforts to obtain information on the topic emerged: (1) the complexity of sex and gender subject matters, (2) patients' dependence on others, and (3) uncertainty about the course of recovery. The first diminished the patient's desire to seek further information. Dependence on others and trust that others were in a better position to use the information, compelled patients to redirect the need for education to clinicians, significant others, and the public on the unique post-injury experiences of men and women. Uncertainty about what to expect in the acute phases, and a feeling of identity loss in the chronic phases, established patients' desire to seek out only the information they believed to be necessary for them to carry on with life.
Nalder, Zabjek, Dawson et al.	Traumatology	2018	Canada	Not reported	Department of Occupational Science and Occupational Therapy & Rehabilitation Sciences Institute, University of Toronto	All HIC	Canadian Journal of Neurological Sciences	***Research Priorities for Optimizing Long-term Community Integration after Brain Injury***	To provide consensus on the research priorities necessary for improving long-term community integration of individuals with traumatic brain injury (TBI) and their caregivers.	N/A	TBI	Canada	Qualitative Consensus (World cafe methodology)	Social constructivist paradigm	Patients AND Family members, carers, signifcant others AND HCPs AND non-clinical providers	54	Other: World Cafe discussions	Content analysis	Hsieh & Shannon (2005)	Not reported	The consensus on prioritized research directions included developing interventions to optimize the functioning and participation of individuals with TBI, reducing caregiver burden, and evaluating how emerging technology can facilitate delivery of care.
Paterson, Kieloch and Gmiterek	Traumatology	2001	Canada	RN/Nurse	School of Nursing	All HIC	Rehabilitation nursing : the official journal of the Association of Rehabilitation Nurses	***'They never told us anything': postdischarge instruction for families of persons with brain injuries***	To report on an analysis of why some families of survivors of traumatic brain injury (TBI) do not perceive that they were prepared for the postdischarge experience, despite discharge planning and teaching by rehabilitation hospital staff and third-party insurance adjusters.	Adult	TBI	Canada	Qualitative (Interpretive description)	Naturalistic inquiry; Human Systems Model	Family members, carers, signifcant others AND HCPs AND non-clinical providers	40	Mixed: Interviews (open, unstructured, in-depth, semi-structured, open ended) AND focus groups	Other: Inductive analysis	Lincoln & Guba (1985)	Not reported	Most family members did not recall being taught about what to expect or resources available to them. Healthcare professionals and insurance adjusters, however, stated that extensive discharge planning and multidisciplinary teaching conferences with patients and their families had been held before the patients were discharged. Reasons for such a discrepancy in perceptions are suggested. Implications of these findings for healthcare professionals who conduct discharge teaching in rehabilitation facilities are identified.
Shankar, Nicholas, Mrazik, et al.	Traumatology	2018	Canada	Social Worker	University of Calgary	All HIC	SAGE Open	***Transition From Pediatric to Adult Services: Challenges for Family Caregivers of Young Adults With Traumatic Brain Injury***	What are the lived experiences and challenges faced by family caregivers as they help their young adult TBI survivor transition to services and supports for adults?	Adolescents	TBI	Canada	Phenomenology (Descriptive)	Not reported	Family members, carers, signifcant others	15	Interviews (open, unstructured, in-depth, semi-structured, open ended)	Phenomenological analysis	Colaizzi (1978)	Not reported	Fifteen themes emerged from the analysis and these are discussed under two broad domains: (a) caregivers' experiences and challenges with their relative at the time of transition; (b) caregivers' challenges with services and supports systems during transition. Findings suggest that there is a wide gap between what caregivers need and what is available to them to support their young adult relative through the transition process and beyond. Most caregivers reported that the transition phase is overwhelming and has a profound and pervasive impact on their lives.
Smith and Smith	Traumatology	2000	Canada	RN/Nurse	Faculty of Nursing	All HIC	Care Management Journals	***No map, no guide: Family caregivers' perspectives on their journeys through the system***	To gain an understanding of the experiences of primary caregivers of traumatic brain injury survivors as they attempted to obtain and coordinate services.	Unclear	TBI	Canada	Qualitative	Not reported	Family members, carers, signifcant others	8	Interviews (open, unstructured, in-depth, semi-structured, open ended)	Content analysis	Not reported	Not reported	Four main themes were identified: the search for information, trust, and understanding; the search for support; the need to speak on behalf of the survivor; and navigating the system. Although faced with overwhelming obstacles and irreversible changes in their own lives, the caregivers in this study did not seek to relinquish their role as primary caregiver. However, it was apparent that these individuals urgently needed support to enable them to continue to bear the physical, social, and financial costs of their caregiving responsibilities.
Souesme, Voyer, Gagnon, et al.	Traumatology	2021	Canada	Not reported	Interdisciplinary Center for Research in Rehabilitation and Social Integration	All HIC	Disability and Rehabilitation	***Barriers and facilitators linked to discharge destination following inpatient rehabilitation after traumatic brain injury in older adults: a qualitative study***	To identify facilitators and barriers associated with returning home for older adults having received inpatient rehabilitation after traumatic brain injury (TBI).	Adult	TBI	Canada	Qualitative	Not reported	Patients AND Family members, carers, signifcant others AND HCPs	15	Mixed: Interviews (open, unstructured, in-depth, semi-structured, open ended) AND focus groups	Thematic analysis	Braun & Clarke (2013)	COREQ	Main facilitators to returning home highlighted by all participants were: (1) Patient's adequate health condition and functional status, (2) Access to health and other services at home, (3) Availability of help from a family caregiver. Conversely, if one of these factors was not met, it represented a barrier. Other facilitators identified were (4) Attachment to one's home, (5) Feeling of commitment toward a loved one, (6) Having the possibility of going through a transitional phase, (7) United front between the patient and the family caregiver towards a return home. Additional barriers to returning home included: (8) Incongruent perspectives, and (9) Unclear knowledge about available health and other services at home.
Teel, Caron and Gagnon	Traumatology	2022	Canada	Not reported	School of Physical and Occupational Therapy	All HIC	Journal of Science and Medicine in Sport	***Higher parental stress is significantly related to longer clinical recovery times in concussed children: A mixed-methods study***	We hypothesized that children with parents experiencing high stress or with pre-existing anxiety would take significantly longer to recover from concussion.	Pediatric	TBI (concussion)	Canada	Mixed methods (Convergent parallel design)	Not reported	Patients AND Family members, carers, signifcant others(Only parents completed the follow-up interviews)	12	Mixed: Interviews (open, unstructured, in-depth, semi-structured, open ended) AND questionnaires	Thematic analysis	Braun & Clarke (2006)	Not reported	Parental anxiety was not significantly related to either the child's time in clinic (P = 0.27) or recovery time (P = 0.41). Conversely, higher perceived parental stress was related to longer recovery time (Hazard Ratio: 2.162, 95% CI: 1.075, 4.348; p = 0.03) for the injured child, with similar results for time in clinic (Hazard Ratio: 1.883, 95% CI: 0.966, 3.668, p = 0.06). During the interview, parents expressed their stress was directly tied to their child's symptoms and overall functioning and varied throughout recovery.
Todd, Bhalerao, Vu, et al.	Traumatology	2018	Canada	Not reported	Department of Psychiatry	All HIC	PloS one	***Understanding the psychiatric effects of concussion on constructed identity in hockey players: Implications for health professionals***	To investigate the effect of concussion and psychiatric illness on athletes and their caregivers.	Mixed	TBI (concussion)	Canada	Grounded theory	Interpretivist paradigm	Other: Ice hockey stakeholders	20	Interviews (open, unstructured, in-depth, semi-structured, open ended)	Grounded theory analysis/constant comparison/open, axial and/or selective coding	Strauss & Corbin 1990	Not reported	From this analysis, a common biographical theme emerged whereby the subject's identity as a hockey player, constructed early in life over many years, was disrupted by concussion. Furthermore, some players underwent a biographical deconstruction when they experienced post-concussive mental illness, which was amplified by isolation, stigma from peers, and lack of a clear life trajectory. Many players obtained support from family and peers and were able to recover, as evidenced by the biographical reconstruction of their identity post-hockey concussion.

van Iersse l, O'Neil, Sveistrup, et al.	Traumatology	2021	Canada	Not reported	Children's Hospital of Eastern Ontario Research Institute	All HIC	Disability and rehabilitation	***A qualitative study of persons with persistent post concussion symptoms and clinicians with concussion expertise to inform the development of a concussion-specific questionnaire***	To explore the experiences of adults with persistent post concussion symptoms and clinicians to inform the development of a concussion-specific questionnaire.	Adult	TBI (concussion)	Canada	Qualitative (descriptive)	Ontological critical realism and epistemological constructionism	Patients AND HCPs (inc. NSx)	51	Mixed: Interviews (open, unstructured, in-depth, semi-structured, open ended) AND focus groups	Thematic analysis	Braun & Clarke (2006)	Not reported	Three overarching themes emerged from the data: Functioning, Environmental and Personal Factors, and Capacity. Functioning mapped closely onto Activities and Participation within the ICF. Contextual factors, both Environmental and Personal, had a significant influence on functioning following concussion. Capacity was a unique finding that described how long a person is able to engage in a task before the onset or worsening of symptoms.
Vanderbyl and Gélinas	Traumatology	2017	Canada	RN/Nurse	Jewish General Hospital	All HIC	Pain Management Nursing	***Family Perspectives of Traumatically Brain-Injured Patient Pain Behaviors in the Intensive Care Unit***	To describe what behaviors family caregivers deemed relevant to pain for patients with a traumatic brain injury with an altered level of consciousness in the intensive care unit.	Adult	TBI	Canada	Mixed methods	Not reported	Family members, carers, signifcant others	7	Mixed: Interviews (open, unstructured, in-depth, semi-structured, open ended) AND questionnaires	Content analysis	Graneheim & Lundman (2004)	Not reported	Family caregivers were able to provide rich descriptions of a number of behaviors they observed in their loved ones that were perceived to be relevant indicators of pain, such as muscle tension and key facial expressions and body movements. Several factors influenced how behaviors were interpreted by family, including personal medical beliefs and intimate knowledge of the patient's history.
Wildgoose, Diep, Rendely, et al.	Traumatology	2022	Canada	MD/Physician	Sunnybrook Health Sciences Center	All HIC	Canadian family physician Medecin de famille canadien	***Barriers to and facilitators of return to learning following a sport-related concussion: Perspectives of female secondary school students***	To identify barriers to and facilitators of return to learning for female secondary school students following a sport-related concussion, and to identify critical junctures on the injury-to-recovery continuum that can be targeted to enhance the RTL process.	Adolescents	TBI (concussion)	Canada	Grounded theory	Not reported	Patients	10	Interviews (open, unstructured, in-depth, semi-structured, open ended)	Grounded theory analysis/constant comparison/open and axial/selective coding	Not reported	Not reported	Barriers to RTL included a lack of a graduated RTL process, students' own internal stress, poor communication of expectations, lack of concussion education, and inadequate support from teachers. Facilitators of RTL included academic accommodations and having a primary contact person within the school system. Owing to inconsistent implementation, the impact of the Green Folder intervention as a facilitator of RTL remains unknown.
Baldeo, D'Souza, Haag, et al.	Traumatology	2022	Canada	Not reported	Acquired Brain Injury Research Lab/ Toronto Rehabilitation Institute (KITE)	All HIC	Disability and rehabilitation	***A thematic analysis of patients' and their informal caregivers' gendered experiences in traumatic brain injury***	How is gender expressed in the perceived experiences of patients with traumatic brain injury and their informal caregivers?	Adult	TBI	Canada	Qualitative	Critical realist	Patients AND Family members, carers, signifcant others	15	Interviews (open, unstructured, in-depth, semi-structured, open ended)	Reflexive thematic analysis	Braun & Clarke (2014)	COREQ	Three specific themes relating to gender were identified: (1) Gender designations of "man" and "woman" reflects how participants related to their socially assigned roles; (2) Post-injury performativity of gender reflects how participants' roles, behaviors, and expressions transformed post-injury; and (3) Gender in giving and receiving care reflects participants' understanding of care and support, and positive and negative gendered experiences in these contexts.
Zhang, J. J. and Li, Y. Q. and Gu, Y. D. and Fei, Y. Y. and Yang, G. P. and Gu, Y. and Xu, X. J.	Traumatology	2022	China	Unclear/not stated	Department of Neurosurgery	All LMIC	Acta Neurochirurgica	***Status and influencing factors of disease uncertainty among family caregivers of patients with moderate and severe craniocerebral injury: a quantitative and qualitative study***	to understand the state of the uncertainty of the disease of family caregivers of patients with moderate and severe craniocerebral injury in China, to analyze the influencing factors, and to explore the specific resource of the uncertainty of the disease combined with qualitative study.	Unclear	Craniocerebral injury	China	Mixed methods,	Not explicitly stated	Family/Carer/Significant other	17	Interviews (semi-structured, open, in depth)	Phenomenological analysis (Colaizzi 7-step analysis)	Sanders (2003)	Not reported	The qualitative research focuses on two main topics: the sources of disease uncertainty among family caregivers of patients with moderate and severe craniocerebral injury and experience to cope with the situation.
Hou, Huang, Prakash, et al.	Traumatology	2013	China	Not reported	Department of Psychology	Mixed HIC/LMIC	Annals of Indian Academy of Neurology	***Infrequent near death experiences in severe brain injury survivors - A quantitative and qualitative study***	Aimed at collecting this valuable information (...memory of post-traumatic coma...) from survivors of severe head injury after a prolonged coma.	Adult	TBI	China	Mixed methods (Surveys; Interpretative phenomenological analysis)	critical realism and the social cognition paradigm.	Patients	3	Mixed: Questionnaire AND IPA Interview	IPA (idiographic)	Smith & Osborn (2003)	Not reported	We found that contrary to earlier incidence reports, NDEs in post head injury patients were markedly low. Only 3 out of 86 of the patients recruited had a clear and confident experience of NDE. We conducted a qualitative study to explore further into these experiences. IPA of these 3 patients revealed four master themes: 1. Unique light visions 2. Intense feelings of astonishment, pleasure, and fear 3. The sense of helplessness 4. Supernatural but rationality of experience.
Hindhede, A. L.	Traumatology	2024	Denmark	Other: Associate professor, senior researcher; experienced researcher in qualitative methodology and public health	Department of Public Health; Center for Health Research	N/A	Health Expectations	***Struggling with capital: Recovery after severe traumatic brain injury among working-age individuals in Denmark***	What types of resources do Danish working-age severe TBI survivors accumulate during rehabilitation? And how do these resources relate to their own sense of recovery?	Adult	TBI	Denmark	Qualitative	Bourdieu's capital theory	Patients AND Families/carers/Significant other	30	Interviews (semi-structured, open, in depth)	Other: Thematic analysis abductive approach	Tavory and Timmermans (2014); Thompson (2022)	Not reported	During the initial phases of rehabilitation (acute and subacute), survivors invest in their physical bodies and acquire physical capital. However, they encounter a range of complex barriers when attempting to convert this capital into the resources necessary for re-entering the workforce or pursuing education. These difficulties are linked to the lack of specialized community services in the later phases of their rehabilitation trajectory.
Hindhede, Anette Lykke and Poulsen, Ingrid	Traumatology	2022	Denmark	Other: social science researcher	Department of Culture and Learning	All HIC	Disability and Rehabilitation: An International, Multidisciplinary Journal	***The value of social networks to individuals with a severe traumatic brain injury: A mixed methods approach***	In this study we ask: what is the nature and strength of the social relations of working-age individuals who have survived a severe TBI? How does the accident affect their social relations up to five years after the accident in terms of changes in responsibilities, and relationship roles?	Adult	TBI	Denmark	Mixed methods,	Critical realist ontology	Patients AND Families/carers/Significant other	53 patients completed the survey; interviews were conducted with 4 patients and their families	Mixed/multiple: Surveys AND Interviews	Thematic analysis	non stated	Not reported	Close relatives experienced a dramatic change in the nature of their relationships with the survivor. They also struggled greatly with the rehabilitation health system, which in many cases affected their own careers.
Graff, Christensen, Poulsen, et al.	Traumatology	2018	Denmark	Not reported	Department of Anesthesia, Center of Head and Orthopedics, Rigshospitalet, University of Copenhagen and Trauma Center and Acute Admission, Rigshospitalet	All HIC	Disability and Rehabilitation	***Patient perspectives on navigating the field of traumatic brain injury rehabilitation: a qualitative thematic analysis***	To provide an understanding of the lived experience of rehabilitation in adults with traumatic brain injury (TBI) from hospital discharge up to four years post-injury.	Adult	TBI	Denmark	Phenomology	Hermeneutical phenomenological approach	Patients	12	Interviews (open, unstructured, in-depth, semi-structured, open ended)	Thematic analysis	Braun & Clarke (2006)	Not reported	Three main themes emerged during analysis: A new life, Family involvement, and Rehabilitation impediments. These themes and their sub-themes described the patient perspective of TBI and rehabilitation post hospitalization. Participants reassessed their values and found a new life after TBI. Family caregivers negotiated rehabilitation services and helped the participant to overcome barriers to rehabilitation. Although participants were entitled to TBI rehabilitation, they had to fight for the services they were entitled to.

Graff, Deleu, Christiansen, et al.	Traumatology	2021	Denmark	Not reported	Department of Anesthesia, Center of Head and Orthopedics, University Hospital Rigshospitalet, Copenhagen University Hospital and Danish Concussion Center	All HIC	Neuropsychological Rehabilitation	***Facilitators of and barriers to return to work after mild traumatic brain injury: A thematic analysis***	To examine RTW from the perspective of those who have experienced persistent PCS following mTBI and to assess how relevant actors either facilitate or obstruct a successful RTW.	Adult	TBI (concussion)	Denmark	Phenomenology	Hermeneutical phenomenological approach	Patients	22	Interviews (open, unstructured, in-depth, semi-structured, open ended)	Thematic analysis	Braun & Clarke (2006)	Not reported	Three main themes emerged. (1) Worker–employer relationship: Workplace accommodations such as decreased working hours, modified working conditions, and support from co-workers were lacking. (2) The role of the general practitioner: The general practitioner was lacking treatment and referral opportunities and failed to provide the patient with relevant and individualized guidance. (3) Municipal case management: Participants perceived being met with distrust by social workers, follow-up assessments were too frequent, unnecessary, and did not target concussion, and rehabilitation was referred too late.
Guldager, Willis, Larsen, et al.	Traumatology	2019	Denmark	Not reported	RUBRIC (Research Unit on Brain Injury Rehabilitation Copenhagen), Department of Neurorehabilitation, Traumatic Brain Injury Unit, Copenhagen University Hospital and Department of Learning and Philosophy	All HIC	Journal of Clinical Nursing	***Relatives' strategies in subacute brain injury rehabilitation: The warrior, the observer and the hesitant***	To explore the experience of the rehabilitation process from the perspectives of relatives of patients with a traumatic brain injury. To identify relatives' strategies and practices in the rehabilitation process as evidenced in meetings with providers.	Adult	TBI	Denmark	Qualitative	Bourdieu & Accardo	Family members, carers, signifcant others	11	Mixed: Interviews (open, unstructured, in-depth, semi-structured, open ended) AND observations	Content analysis	Graneheim & Lundman (2004)	Not reported	Drawing on Bourdieu's concept of strategy, three relative positions were identified, the warrior, the observer and the hesitant. These positions illustrate how different relative positions and their related dispositions influence the strategies used by relatives of patients with a severe traumatic brain injury evidenced in how they act, participate and relate to both the patient and the providers during the course of rehabilitation.
Abrahamson, Jensen, Springett, et al.	Traumatology	2017	UK	Not reported	Senior Lecturer	All HIC	Disability and Rehabilitation	***Experiences of patients with traumatic brain injury and their carers during transition from in-patient rehabilitation to the community: a qualitative study***	To explore the experiences of individuals who have had a severe traumatic brain injury (TBI) and their carers in the first month post-discharge from in-patient rehabilitation into living in the community.	Adult	TBI	England	Qualitative	Critical realism	Patients AND Family members, carers, signifcant others	19	Interviews (open, unstructured, in-depth, semi-structured, open ended)	Thematic analysis	Braun & Clarke (2006)	Not reported	Firstly, perceptions of support were mixed but many patients and carers felt unsupported in the inpatient phase, during transitions between units and when preparing for discharge. Secondly, they struggled to accept a new reality of changed abilities, loss of roles and loss of autonomy. Thirdly, early experiences post-discharge exacerbated fears for the future.
Lindlöf, J. and Turunen, H. and Coco, K. and Huhtakangas, J. and Verhaeghe, S. and Välimäki, T.	Traumatology	2024	Finland	Nurse/RN/RGN	Department of Nurisng Science	All HIC	Journal of Advanced Nursing	***Empowering Support for Family Members of Patients With Traumatic Brain Injury During the Acute Care: Insights From Family Members and Nurses***	To investigate the perceptions of family members (FMs) of patients with traumatic brain injury (TBI) and nurses on empowering support and its implementation during the acute phase within Finnish neurosurgical and neurological care in hospital settings, focusing on identifying similarities and differences in their viewpoints.	Adult	TBI	Finland	Qualitative descriptive	Conceptual framework of empowering support	Families/carers/ Significant other AND HCPs	18	Other: Word Café method	Other (please state)	Leino-Kilpi et al. (2005), Timmermans and Tavory (2012), Lindlöf et al. (2023)	COREQ	Four main themes were identified: (1) FMs' diverse information and guidance needs of TBI, treatment and its impact on family life, (2) support based on empowering FMs in participation, competence and decision-making, (3) empowering FMs through collaborative nursing practices and interprofessional support, and (4) internal and external hospital support enhancing and promoting the empowerment of FMs.
Jourdan, Bahrami, Azouvi, et al.	Traumatology	2019	France	Not reported	Department of Physical Medicine and Rehabilitation	All HIC	Brain Injury	***Practitioners' opinions on traumatic brain injury care pathways in Finland and France: different organizations, common issues***	To compare TBI pathways of care and practitioners' views on quality of care issues in two large European areas: Varsinais-Suomi, Finland and Ile-de-France, France.	Unclear	TBI	Finland/France	Qualitative	Donabedian's structure and process model.	HCPs (inc. NSx)	10	Interviews (open, unstructured, in-depth, semi-structured, open ended)	Thematic analysis	Not reported	Not reported	Main differences in organization of care pathways for people with TBI were related to financing modalities, number of pathway alternatives, inpatient versus outpatient rehabilitation, and indirect versus direct referrals to rehabilitation. Similar categories of issues were raised in the two settings. Issues in structures involved availability of services, financial access, and heterogeneity of expertise. Issues in processes involved diagnosis and follow-up, training regarding cognitive impairments, decision-making for referrals, transition delays, and care pathways of very severely affected patients.
Burkadze, Chikhladze, Lobzhanidze, et al.	Traumatology	2021	Georgia	Not reported	Department of Public Health, Faculty of Medicine	Mixed HIC/LMIC	Journal of injury & violence research	***Brain injuries: health care capacity and policy in Georgia***	To identify current TBI data collection practices and capacity in Georgia, focusing on pre-hospital, hospital, and rehabilitation treatment.	Unclear	TBI	Georgia	Not stated	Not reported	HCPs (inc. NSx)	10	Interviews (open, unstructured, in-depth, semi-structured, open ended)	Other: coded to identify themes	Not reported	Not reported	Pre-hospital triage protocols were not routinely used to match patient treatment needs with hospital capacity. All hospitals provided specialist care for TBI 24 hours/day. MRI was available at only three (30%) centers, and in-hospital rehabilitation units were available in only one (10%). No center used a defined protocol for treating TBI patients and no national protocol exists.
Krenz, Timmermann, Gorbunova, et al.	Traumatology	2021	Germany	Not reported	Department of Medical Psychology and Medical Sociology	All HIC	PLoS ONE	***Health-related quality of life after pediatric traumatic brain injury: A qualitative comparison between children's and parents' perspectives***	To investigate in a first exploratory inspection whether and to what extent the perceptions of HRQoL after pediatric TBI coincide or differ between the children and adolescents after TBI and their parents.	Pediatric	TBI	Germany	Qualitative	Not reported	Patients AND Family members, carers, signifcant others	45	Focus groups	Content analysis	Mayring (2010)	Not reported	The Analyzes led to 32 subcategories, which were assigned to six main theoretically based HRQoL categories. Many agreements exist between the C&A's and parents' perspectives within the main categories, however their focus on HRQoL differs, especially concerning age-related contents. Parents of the youngest participant group already focus on topics such as autonomy, whereas this only becomes relevant for C&A from the age of eight years on. Interestingly, even 5-year-old children were able to discuss their HRQoL, which indicates the importance of a self-report instrument.
Mohammed, R. S. and Boateng, E. A. and Amponsah, A. K. and Kyei-Dompim, J. and Laari, T. T.	Traumatology	2023	Ghana	Nurse/RN/RGN	Department of Nursing	All LMIC	Plos One	***Experiences of family caregivers of people with spinal cord injury at the neurosurgical units of the Komfo Anokye Teaching Hospital, Ghana***	To explore the experiences of family caregivers of individuals with SCI in Ghana.	Unclear	SCI	Ghana	Qualitative descriptive	Not explicitly stated	Family/Carer/Significant other	10	Interviews (semi-structured, open, in depth)	Phenomenological analysis	Colaizzi (n.d.)	COREQ	In all, 4 main themes emerged from the analysis of data (1) becoming a caregiver, (2) roles of the caregiver, (3) the burden of caregiving, and (4) coping strategies. The family caregivers provided vital assistance to their relatives with SCI and experienced physical and financial burdens as a result of the care. Due to the strains involved in the caregiving process, family caregivers adopted various strategies to cope with the situation.

Man	Traumatology	2002	Hong Kong	Not reported	The Hong Kong Polytechnic University	N/A	Brain Injury	***Family caregivers' reactions and coping for persons with brain injury***	To examine the impact of brain injury on the family and the complex array of factors that appears to be related to effective family coping and their independence. The reactions of families under stress and their coping strategies are summarized.	Mixed	TBI	Hong Kong	Mixed methods (Questionnaires; Descriptive statistics; Qualitative content analysis)	Not reported	Family members, carers, signifcant others		50	Mixed: Interviews with open ended questions AND Questionnaires	Content analysis	Not stated	Not reported	A total of 50 family members were successfully recruited for interview. They were found to show typical coping strategies, including shock and uncertainty, which are suggested to be relating closely to the nature of brain injury and the difficulties in managing it. The physical and psychological burdens involved in caring for members with brain injury were also reflected. Content analysis of the long interviews of four selected families showed that it was not every family that coped well. Possible factors leading to better adjustment, such as clear personal expectations, a desire to master the situation, strong motivation, flexibility to adjust life goals and awareness of one's own powerless state are proposed.
Kanmani and Raju	Traumatology	2019	India	Not reported	Departments of Psychiatric Social Work and Psychiatry, National Institute of Mental Health and Neurosciences	All LMIC	Journal of Neurosciences in Rural Practice	***Caregiver's psychosocial concerns and psychological distress in emergency and trauma care setting***	To explore the psychosocial distress and caregivers' concerns in emergency and trauma care (ETC) setting.	Adult	TBI	India	Mixed methods	Not reported	Family members, carers, signifcant others		50	Mixed: Focus group AND questionnaires	Thematic analysis	Not reported	Not reported	In the quantitative analysis, caregivers' mean age was found to be 45 (mean = 45.00 ± 13.83) years. Caregivers had experienced mild depression (13.36 ± 3.07), moderate anxiety (13.70 ± 3.03), and minimum stress (13.66 ± 2.98) levels. Qualitative results identified the following themes: difficulty in accessing timely care, uncertainty about the prognosis and future, family concerns and financial constraints, personal feelings and personal needs, and supportive care. Chi-square test revealed that there was no significant association between gender and depression ($\chi2 = 2.381$ $P < 0.12$), anxiety ($\chi2 = 0.01$ $P < 0.92$), and stress ($\chi2 = 0.235$ $P < 0.61$) levels of caregivers.
Gupta, Saksham, Khajanchi, et al.	Traumatology	2020	USA	Not reported	Department of Neurosurgery	Mixed HIC/LMIC	Asian journal of neurosurgery	***Traumatic Brain Injury in Mumbai: A Survey of Providers along the Care Continuum***	To identify provider-perceived themes related to TBI care in Mumbai.	Unclear	TBI	India	Qualitative	Not reported	HCPs (inc. NSx)		50	Interviews (open, unstructured, in-depth, semi-structured, open ended)	Thematic analysis	Joffe (2004)	Not reported	Four major themes emerged: Workforce, equipment, financing care, and the family and public role. These themes were often discussed in the context of their effects on increasing or decreasing complications and delays. Participants developed adaptations when managing shortcomings in these thematic areas. These adaptations included teamwork during workforce shortages and resource allocation when equipment was limited among others.
Carrier, S. L. and Ponsford, J. and McKay, A.	Traumatology	2023	Australia	Other: PhD student with clinical expereince	Turner Institute for Brain and Mental Health; Monash-Epworth Rehabilitation Research Centre	All HIC	Disability and Rehabilitation	***Managing agitation during early recovery following traumatic brain injury: qualitative interviews with clinicians***	1. What strategies do clinicians use to manage common agitated behaviours? 2. How effective are the strategies currently in use? 3. What are the challenges and facilitators of working with agitated patients?	Unclear	TBI; agitation	International	Qualitative descriptive	Non stated	Mixed HCPs: Inc. NSx		33	Interviews (semi-structured, open, in depth)	Thematic analysis (inductive)	Braun and Clarke (2013)	SRQR	The central theme of the interviews was the effective management of agitation, consisting of three sub-themes: managing the safety of staff and patients, reducing triggers for agitation, and implementing behavioural principles for managing agitation. Two overarching themes influenced the effective management of agitation: clinician-related factors and systemic factors.
Smith, B. G. and Whiffin, C. J. and Esene, I. N. and Karekezi, C. and Bashford, T. and Khan, M. M. and Solla, D. J. F. and Devi, B. I. and Paiva, W. S. and Servadei, F. and Hutchinson, P. J. and Kolias, A. G. and Figaji, A. and Rubiano, A. M.	Traumatology	2022	UK	Other: MB-PhD candidate	Division of Neurosurgery, Department of Clinical Neurosciences	Mixed HIC/LMIC	Plos One	***Neurotrauma clinicians' perspectives on the contextual challenges associated with traumatic brain injury follow up in low-income and middle-income countries: A reflexive thematic analysis***	to explore the specific contextual challenges of, and possible solutions to improve, long-term follow-up and outcome data collection following TBI in countries identified as low- or middle-income as defined by the World Bank	n/a	TBI	International	Qualitative	Naturalistic constructivist	Neurosurgeons (inc. trainees/residents)		18	Interviews (semi-structured, open, in depth)	Reflexive thematic analysis	Braun and Clarke (2006, 2014, 2019, 2021)	COREQ	18 neurosurgeons from 13 countries participated in this study, and data analysis gave rise to five themes: Clinical Context: What must we understand?; Perspectives and Definitions: What are we talking about?; Ownership and Beneficiaries: Why do we do it?; Lost to Follow-up: Who misses out and why?; Processes and Procedures: What do we do, or what might we do? The collection of long-term outcome data plays an imperative role in reducing the global burden of neurotrauma. Therefore, this was an exploratory study that examined the contextual challenges associated with long-term follow-up in LMICs. Where technology can contribute to improved neurotrauma surveillance and remote assessment, these must be implemented in a manner that improves patient outcomes, reduces clinical burden on physicians, and does not surpass the comprehension, capabilities, or financial means of the end user. Future research is recommended to investigate patient and family perspectives, the impact on clinical care teams, and the full economic implications of new technologies for follow-up.
Godwin, Chappell and Kreutzer	Traumatology	2014	USA	Not reported	Department of Physical Medicine and Rehabilitation	All HIC	Brain Injury	***Relationships after TBI: A grounded research study***	To develop a framework for conceptualizing and assessing couples after TBI	Unclear	TBI	International	Grounded theory	Not reported	Patients AND Family members, carers, signifcant other AND HCPs	Other: 45		Documentary sources: blog entries, narratives, memoirs, clinician authored documents	Grounded theory analysis/constant comparison/open, axial and/or selective coding	Corbin and Strauss (2008), Glaser (1965, 1978), Dye et al. (2000), Kolb (2012)	Not reported	Five primary themes emerged: Ambiguous Losses, Identity Reformations, Tenuous Stability, Non Omnes Moriar and The New Us. From these, two grounded theories were developed: Relational Coring and Relational Recycling.
Adineh, M. and Molavynejad, S. and Elahi, N. and Jahani, S. and Mohsen, S.	Traumatology	2023	Iran	Other: PhD student	Nursing Care Research Center in Chronic Diseases, School of Nursing and Midwifery	All LMIC	Jundishapur Journal of Chronic Disease Care	***"I Was Lying on a Bed Just Like the Wounded in the War": A Qualitative Study Explaining the Experiences of Brain Injury Patients Hospitalized in ICU***	To explain the experiences of brain injury patients hospitalized in the ICU.	Adult	ICU; EDH;SAH; SDH; DAI	Iran	Qualitative	Non stated	Patients		14	Interviews (semi-structured, open, in depth)	Content analysis	Graneheim and Lundman (2004)	Not reported	Data analysis indicated 238 compact semantic units (initial codes). Based on their similarities and differences, these codes were organized into 22 subcategories, 6 categories, and 2 themes, including "the suffering body and soul" and "illumination in the dark".
Alimohammadi, E. and Arast, A. and Vlaisavljevic, Z. and Abdi, A. and Ramadhan, H.	Traumatology	2023	Iran	Neurosurgeon	Imam Reza Hospital, Kermanshah University of Medical Sciences	Mixed HIC/LMIC	Sage Open Medicine	***The experiences of the caregivers caring for the patients in persistent vegetative state due to traumatic brain injury***	To investigate the family care givers experiences of caring for the "persistent vegetative state patients, following the TBI	Adult	TBI; PVS	Iran	Phenomenology	Non stated	Family/Carer/Significant other		12	Interviews (semi-structured, open, in depth)	Phenomenological analysis (Colaizzi 7-step analysis)	Colaizzi; Morrow, Rodriguez, and King (2015)	Not reported	Five themes include "uncountable struggles/challenges," "looking for peace," "therapeutic concerns," "preserving the connection," and "unheard sounds."
Nayeri, Ahmadi Chenari, Esmaeili, et al.	Traumatology	2022	Iran	Professor Nursing & Midwifery	Nursing and Midwifery Care Research Center, School of Nursing and Midwifery	Mixed HIC/LMIC	Nursing Science Quarterly	***Caring for Patients in a Coma Following a Traumatic Brain Injury in Iran***	To better understand the processes and meaning of caring for persons in a coma following a TBI in Iran	Adult	TBI	Iran	Grounded theory	Not reported	Patients AND Family members, carers, signifcant other AND HCPs		20	Interviews (open, unstructured, in-depth, semi-structured, open ended)	Grounded theory analysis/constant comparison/open, axial and/or selective coding	Corbin & Strauss (2008)	Not reported	The main themes that were identified were chaos and confusion, inconsistent quality, multilateral support, and improving care. The processes of caring for persons in a coma following a TBI were found to be an ever-changing, multidimensional, context-dependent process.

Nayeri, Esmaeili, Farsi, et al.	Traumatology	2020	Iran	RN/Nurse	Nursing and Midwifery Care Research Center	All LMIC	Journal of family medicine and primary care	***Taking care experiences of improved comatose patients with traumatic brain injury and their families***	To describe the care-taking experiences of coma patients and their families during and after treatment.	Adult	TBI	Iran	Qualitative	Not reported	Patients AND Family members, carers, signifcant others	14	Interviews (open, in-depth, semi-structured, open ended)	Content analysis	Elo & Kyngäs (2008)	Not reported	The results showed four main themes and eight categories. Themes included "crisis," "comprehensive support," communication," and "unprofessional care." Patients and families experience a crisis during hospitalization and after discharge. Therefore, patients and families need psychological support. Informing families and communication decrease the conflicts between healthcare personnel and the family. Medical staff must be careful about their statements and behaviors during comatose patients' care because they understand the care process.
SohrabiAsl, Ghodsi, Arejan, et al.	Traumatology	2021	Iran	Not reported	Sina Trauma and Surgery Research Center	Mixed HIC/LMIC	Chinese Journal of Traumatology - English Edition	***Reasons for delayed spinal cord decompression in individuals with traumatic spinal cord injuries in Iran: A qualitative study from the perspective of neurosurgeons***	To determine the reasons for the observed decompression surgery delay in Iran from the perspective of neurosurgeons.	Unclear	Spine, Spinal cord injury	Iran	Qualitative (Encounter context themes methodological device)	Interpretivism paradigm	NSx	12	Interviews (open, unstructured, in-depth, semi-structured, open ended)	Content analysis	Krippendorff (2018)	SRQR	The findings of the current study suggest that patient-related factors constitute more than half of the codes extracted from the interviews. Overall, the type of injury, presence of polytrauma, and surgeons' wrong attitude are the main factors causing delayed spinal cord decompression in Iranian patients from the perspective of neurosurgeons. Other notable factors include delay in transferring patients to the trauma center, delay in availability of necessary equipment, and scarce medical personnel.
O'Keeffe, Dunne, Nolan, et al.	Traumatology	2020	Ireland	Neuropsycholo gist	School of Psychology; Department of Clinical Psychology	All HIC	Brain Injury	***"The things that people can't see" The impact of TBI on relationships: an interpretative phenomenological analysis***	To explore the impact of TBI on couple relationships, from the perspective of both injured and uninjured partners in the relationship	Adult	TBI	Ireland	IPA	Not reported	Patients AND Family members, carers, signifcant others	11	Interviews (open, unstructured, in-depth, semi-structured, open ended)	IPA	Smith, Flowers & Larkin (2012)	Not reported	The three major themes emerged. Broken Bonds: "those special things just between the two of us" captures the emotional fallout from TBI on each individual and on the relationship; New Dynamics: "like oil and water" describes the effect of individual changes on relationship dynamics in general, on sexuality, conflict and family life; Moving Forward Together: "We figure it out" describes coping strategies in maintaining relationships post-TBI including hope, time, understanding TBI and positive reappraisal
Pistarini, Aiachini, Coenen, et al.	Traumatology	2011	Italy	Not reported	Coordinator Center: IRCCS S. Maugeri Foundation, Neurorehabilitatio n Unit	All HIC	Disability and Rehabilitation	***Functioning and disability in traumatic brain injury: The Italian patient perspective in developing ICF Core Sets***	To explore the aspects of functioning and health relevant to patients with traumatic brain injury (TBI) and to the caregivers of TBI patients explicitly involved in the preliminary study for the Development of the ICF Core Set for TBI using a qualitative research method.	Unclear	TBI	Italy	Qualitative	International Classification of Functioning, Disability and Health (ICF)	Patients AND Family members, carers, signifcant others	74	Mixed: Interviews (open, unstructured, in-depth, semi-structured, open ended) AND focus groups	Other: Meaning condensation procedure	Kvale (1996)	Not reported	Eighteen focus groups and five single interviews were performed. Forty-one patients participated in 10 focus groups. Thirty-three caregivers participated in eight focus group and six caregivers underwent a single interview. Saturation at 10%% cutoff was reached. Based on 10 patient focus groups, we linked the concepts to 144 2nd level ICF different categories. In the eight focus group with caregiver we linked the concepts to129 2nd level categories. In the single interviews, we could link the concepts only to Environmental factors and we linked to 25 ICF different categories.
Fumiyo, Sumie, Akiko, et al.	Traumatology	2009	Japan	Professor (specialty not reported)	Katayanagi Advanced Research Laboratories, Tokyo University of Technology	All HIC	Journal of Neuroscience Nursing	***Psychosocial adjustment process of mothers caring for young men with traumatic brain injury: Focusing on the mother-son relationship***	To elucidate the psychosocial adjustment process of mothers for 5 years following the injury to facilitate the social rehabilitation of both mother and son	Mixed	TBI	Japan	Qualitative (descriptive)	Not reported	Family members, carers, signifcant others	13	Interviews (open, unstructured, in-depth, semi-structured, open ended)	Grounded theory analysis/consta nt comparison/op en, axial and/or selective coding	Kinoshita (2003)	Not reported	56 concepts were identified, 18 categories and then 8 core categories were semantically created. The 8 core categories were (a) avoid contact with son, (b) support son based on mother's own desire, (c) support son alone without external support, (d) realize mother's care limits and seek external support sources, (e) request and utilize external support, (f) support and work toward son's independence while monitoring his interactions with others, (g) mother considers own feelings and reenters society, and (h) strive for continued care of son with mother's acquired energy and efficient management skills. These were grouped into the following five stages: (a) avoidance, (b-c) closed, (d) support seeking, (e-f) withdrawal, and (g-h) reconstruction.
Ishikawa, Suzuki, Okumiya, et al.	Traumatology	2011	Japan	RN/Nurse	Department of Nursing	All HIC	Rehabilitation Nursing	***Experiences of family members acting as primary caregivers for patients with traumatic brain injury***	To present an overall picture of the caregiving experiences of families caring for patients with TBIs to provide nursing support to families in this situation.	Adult	TBI	Japan	Qualitative	Not reported	Family members, carers, signifcant others	15	Interviews (open, unstructured, in-depth, semi-structured, open ended)	Content analysis	Berelson, (1952)	Not reported	One thousand eighteen recording units were grouped into seven categories: (1) spending time with the patient with TBI and understanding invisible disabilities, (2) evaluating medical professionals' handling of the case, (3) devoting oneself to caring for the patient with TBI despite feeling psychological distress, (4) accepting the disability and constructing a care system, (5) seeking out and participating in specialized treatment (rehabilitation) for TBIs, (6) hoping for better understanding and creation of an appropriate response system for TBIs, and (7) making efforts to influence people around the caregiver and those in similar circumstances to promote deeper understanding of TBIs.
Kohler, Nwe Myint, Wynn, et al.	Traumatology	2022	UK	Not reported	Division of Anesthesia, Department of Medicine	Mixed HIC/LMIC	BMJ open	***Systems approach to improving traumatic brain injury care in Myanmar: a mixed-methods study from lived experience to discrete event simulation***	To develop a mixed-method approach to understand the TBI pathway based on the lived experience of local people, supported by quantitative methodologies and to determine potential improvement targets.	Adult	TBI	Myanmar	Mixed methods (Healthcare system modelling using discrete event simulation (DES), Soft Sytems methodology)	Constructivism	Patients AND Family members, carers, signifcant other AND HCPs((inc. NSx)	104	Other: narrative data (workshops and interviews) supplemented by participatory diagramming	Other: Narratives were analyzed to identify key areas of concern, along with the development of a consensus understanding of the system features which were central to these.	Not reported	GRAMMS	The TBI pathway was outlined, with system boundaries defined around the management of TBI once admitted to the neurosurgical unit. Retrospective data showed 18% mortality, 71% discharge to home and an 11% referral rate. DES was used to investigate the system, showing its vulnerability to small surges in patient numbers, with critical points being CT scanning and observation ward beds. This explorative model indicated that a modest expansion of observation ward beds to 30 would remove the flow-limitations and indicated possible consequences of changes.
Donker-Cools, Schouten, Wind, et al.	Traumatology	2018	The Netherlands	Not reported	Academic Medical Center, Department: Coronel Institute of Occupational Health	All HIC	Disability and Rehabilitation	***Return to work following acquired brain injury: the views of patients and employers***	To investigate which factors are experienced as facilitators of or barriers to return to work (RTW), or as solutions to RTW-problems, by patients with acquired brain injury (ABI) and by employers.	Adult	Mixed ABI	Netherlands	Qualitative	Not reported	Patients AND Non-clinical providers	17	Interviews (open, unstructured, in-depth, semi-structured, open ended)	Thematic analysis	Not reported	COREQ	Both patients and employers distinguished patient-related and work-related facilitators. When questioned about barriers, both patients and employers emphasized the importance of work-related factors such as sensory overload at the workplace and condition-related factors such as fatigue. Patients regarded poor guidance and support as barriers, but employers did not. Employers and patients suggested that solutions to RTW-problems were work-related, if necessary backed up by professional supervision. Patients also mentioned the need for understanding and acceptance of the limitations resulting from ABI.

Hoogerdijk, Runge and Haugboelle	Traumatology	2011	The Netherlands	Occupational therapist (Student)	Rehabilitation Center Heliomare, Department of Occupational Therapy	All HIC	Scandinavian Journal of Occupational Therapy	***The adaptation process after traumatic brain injury An individual and ongoing occupational struggle to gain a new identity***	To understand better how individuals with traumatic brain injury make sense of their adaptation process and their performance of occupations within this process.	Adult	TBI	Netherlands	Narrative inquiry	Not reported	Patients	4	Interviews (open, in-depth, semi-structured, open ended)	Narrative analysis	Polkinghorne (1996) Molineux and Rickard (2003)	Not reported	The results indicate that the adaptation process following traumatic brain injury is (1) a necessary struggle to gain a new identity; (2) facilitated by engagement in familiar occupations in familiar environments; (3) a protracted learning process that continues long after rehabilitation ends; (4) individual and situated.
Roodbeen, Lugtenberg, Pöstges, et al.	Traumatology	2022	The Netherlands	Not reported	Tilburg School of Social and Behavioral Sciences, Tranzo Scientific Center for Care and Wellbeing Research Department, Breuer & Intraval Research and Consultancy	All HIC	BMJ open	***Experiences of recovery and posthospital care needs of working-age adults after physical trauma: a qualitative focus group study***	To explore experiences of recovery after physical trauma and identify long-term needs for posthospital care	Adult	TBI; Monotrauma, Polytrauma	Netherlands	Phenomenology	Phenomonological	Patients	39	Focus groups	Thematic analysis	Braun & Clarke (2006)	SRQR	Despite differences in type and severity of their injuries, participants all struggled with the impact that trauma had on various aspects of their lives. They experienced recovery as an unpredictable and inconstant process aimed at resuming a meaningful life. Work was often perceived as an important part of recovery, though the value attributed to work could change over time. Participants struggled to bring the difficulties they encountered in their daily lives and at work to the attention of healthcare professionals (HCPs). While posthospital care needs varied between and across groups, all people stressed the need for flexible access to person-Centerd, multidisciplinary care and support after hospital discharge.
Mulligan, T. and Barker-Collo, S. and Gibson, K. and Jones, K.	Traumatology	2023	New Zealand	Unclear/not stated	Clinical Training Programme, School of Psychology	All HIC	Brain Injury	***You only get one brain: adult reflections on coping and recovery after traumatic brain injury in adolescence***	To analyze retrospective qualitative accounts of young adults who had sustained a TBI in adolescence to explore coping and recovery processes specific to this developmental stage.	Adolescents	TBI	New Zealand	Qualitative	Crtiical realism	Patients	13	Interviews (semi-structured, open, in depth)	Thematic analysis	Braun (2008), Braun and Clarke (2017)	Not reported	Thematic data analysis produced two key categories of themes relating to recovery processes: (1) Individual factors impacting coping, with themes of learning to cope with difficulties, seeking acceptance and balance, and finding meaning; and (2) Social factors impacting coping, which included themes of feeling included, relying on family, professionals didn't get it, and lacking someone who understands.
Salmon, D. M. and Walters, S. and Brown, J. and Kerr, Z. Y. and Clacy, A. and Keung, S. and Sullivan, S. J. and Register-Mihalik, J. and Whatman, C. and Sole, G. and Badenhorst, M.	Traumatology	2024	New Zealand	Unclear/not stated	Injury Prevention and Player Welfare, New Zealand Rugby; New Zealand Sports Performance Research Institute	All HIC	International Journal of Sports Science & Coaching	***<i>Managing concussion in the real world</i>: Stakeholder perspectives of New Zealand Rugby's concussion management pathway***	to explore key stakeholders' perceptions of the concussion management pathway, with the aim to inform policy and practice	Mixed	Mild TBI	New Zealand	Qualitative descriptive	Pragmatism	Mixed (please state in comments)	123	Interviews (semi-structured, open, in depth)	Framework analysis	Gale et al. (2013)	Not reported	Themes were organized according to the principles of realist process evaluation that considers contextual factors and mechanisms influencing a program's operation to produce specific outcomes. Contextual factors influencing the concussion management pathway's implementation included governing bodies' support, existing local resources, general concussion attitudes, or concussion severity. The optimal functioning of the concussion management pathway (mechanism) was influenced by (i) pathway resources, (ii) roles and relationships, (iii) buy-in and support towards the concussion management pathway, and (iv) diligence and communication. Outcomes identified included (i) hitting the target (optimally managed and enhanced awareness) or (ii) missing the mark (dissatisfaction or management gaps). Overall, participants found the concussion management pathway valuable. However, the acceptability of certain policy-related aspects and the underlying attitudes associated with these perceptions, are some areas requiring further investigation and support. Tailoring programs according to end-users' perceptions is crucial in developing context-sensitive interventions appropriate for a specific setting. These findings may act as a foundation for investigations of concussion management in other settings.
VanSolkema, M. and McCann, C. M. and Dixon, L. and Horne, J. and Barker-Collo, S. and Foster, A.	Traumatology	2024	New Zealand	Speech and language pathologist	School of Psychology (Speech Science); ABI Rehabilitation New Zealand Ltd	All HIC	Brain Impairment	***An exploration of families' lived experiences of attention-related communication difficulties following traumatic brain injury***	To explore families' experiences of living with someone who has attention and communication difficulties following a traumatic brain injury (TBI)	Adult	TBI	New Zealand	Qualitative	Critical realism and Contextualism	Family/Carer/Significant other	11	Interviews (semi-structured, open, in depth)	Reflexive thematic analysis	Braun and Clarke (2021, 2022)	Not reported	Eleven family members (including wives, mothers, a father, and a daughter) were interviewed. Two themes were generated that explored the lived experiences of the family. The first, adjustment to the new normal, outlines the changes and adaptations the families made to live with and support their loved one's attention and communication difficulties. The second theme, the load the family carries, reviews the two polarities of facilitation and support along with the burden the family endures following the injury.
MacLeod, Surgenor, Levack, et al.	Traumatology	2020	New Zealand	Not reported	Department of Orthopedic Surgery & Musculoskeletal Medicine	All HIC	Brain Impairment	***Patient and clinician experiences of a computerized cognitive battery for use after concussion: A preliminary qualitative study***	To find out more about the experiences of people with concussion who had been assessed in a research setting with a computerized cognitive battery, the NIHTB-CB.	Adult	TBI (concussion)	New Zealand	Qualitative (descriptive)	Not reported	Patients AND HCPs AND Non-clinical providers	10	Interviews (open, unstructured, in-depth, semi-structured, open ended)	Thematic analysis	Thomas (2006); Braun & Clarke (2006)	Not reported	Participants described both positive and negative experiences with the NIHTB-CB and using qualitative description, their experiences were organised into three broad themes: (1) using technology for cognitive testing made sense, (2) there were some cultural relevance questions and (3) cognitive testing after concussion could have challenges. They were positive about the computerised format and range of domains assessed for the concussion context but identified the contextual relevance of some content as having potential to impact on performances.
McPherson, Fadyl, Theadom, et al.	Traumatology	2018	New Zealand	Not reported	The Health Research Council of New Zealand / Center for Person Centered Research	All HIC	Journal of head trauma rehabilitation	***Living Life After Traumatic Brain Injury: Phase 1 of a Longitudinal Qualitative Study***	To explore what helps and hinders recovery and adaptation after disabling traumatic brain injury (TBI) and make recommendations for improving service responsiveness	Mixed (16+)	TBI (concussion)	New Zealand	Qualitative (descriptive)	Charmaz's constructivist approach	Patients AND Family members, carers, signifcant others	66	Interviews (open, unstructured, in-depth, semi-structured, open ended)	Grounded theory analysis/constant comparison/open and axial/selective coding	Glaser & Strauss (1967)	Not reported	Traumatic brain injury produced a complex set of challenges in keeping up with life, and understanding what having a TBI means for, and to, me. This period encompassed a tangled fit and misfit in life as brain injury did not occur in isolation. People had to actively change some aspects of life and yet allow other changes to happen. Valued supports from others included being looked out for and having someone to help drive the process. improved services delivery and better outcomes may result if we respond to the person within his or her context; listen, believe, and acknowledge the person's story through our actions; and avoid assumptions about aspects of life that mean most to people and who/what may help best.
Nandigam, Symonds, Kayes, et al.	Traumatology	2010	New Zealand	Not reported	AUT University, Auckland	All HIC	CHINZ Conference Proceedings	***Mobile phone user interface design for patients with traumatic brain injury***	To determine the kind of mobile phone that would be most acceptable for use by people with traumatic brain injury	Unclear	TBI	New Zealand	Qualitative	Not reported	Patients	4	Interviews (open, unstructured, in-depth, semi-structured, open ended)	Content analysis	Patton (2002)	Not reported	Based on the findings from this study, it is recommended that a mobile phone interface developed for therapeutic use with TBI patients should incorporate the following features: a) soft finger touch; b) large buttons; c) icons supported by titles; and d) a single level menu structure.

Snell, Martin, Surgenor, et al.	Traumatology	2017	New Zealand	Not reported	Concussion Clinic; Department of Orthopaedic Surgery and Musculoskeletal Medicine	All HIC	Disability and Rehabilitation	***What's wrong with me? seeking a coherent understanding of recovery after mild traumatic brain injury***	To generate patient perspectives of MTBI recovery, integrating these with quantitative investigation to isolate factors that might contribute to divergent MTBI outcomes	Adult	Traumatic brain injury (Concussion)	New Zealand	Mixed methods (Case-control, only qualitative reported)	Pragmatist epistemology	Patients	10	Interviews (open, unstructured, in-depth, semi-structured, open ended)	Thematic analysis	Thomas (2006); Braun and Clarke (2006)	Not reported	Three themes: Social scaffolding; validation; what the heck is going on? Participants regardless of recovery status identified the importance of having a coherent understanding of their injury and recovery. Factors facilitating coherence included social support, validation, reassurance, accessing credible evidence-based information and having a pathway to wellness. Findings suggested that coherence could be a helpful umbrella construct worthy of examination in future MTBI research. This construct appears broad and able to cope with the complexity of individual experiences after injury. Implications for rehabilitation Sense of coherence may be a helpful umbrella construct that can facilitate resilience and positive recovery beliefs and expectations after mild traumatic brain injury. Reassurance, validation, and social support appear important and may facilitate injury recovery. Focus on the experiences of people recovering from mild traumatic brain injury may help to refine recovery models and understandings and thus provide more effective intervention targets.
Snell, Martin, Surgenor et al	Traumatology	2019	New Zealand	Not reported	Concussion Clinic; Department of Orthopedic Surgery and Musculoskeletal Medicine	All HIC	Disability and Rehabilitation	***Wrestling with uncertainty after mild traumatic brain injury: a mixed methods study***	Tp explore the intersection between mild traumatic brain injury (MTBI) recovery experiences and injury understandings, using both quantitative and qualitative methods	Mixed	TBI (concussion)	New Zealand	Mixed methods (case control study AND qualitative interviews)	Not reported	Patients	10	Mixed: Interviews (open, unstructured, in-depth, semi-structured, open ended) AND patient outcomes	Thematic analysis (inductive)	Thomas (2006); Braun and Clarke (2006)	Not reported	The quantitative Analyzes revealed differences between the two groups in terms of injury recovery understandings and expectations. The qualitative Analyzes suggested that achieving consistency across information sources was important. By tracing threads back and forth between the component datasets, we identified a super-ordinate meta-theme that captured participants' experiences of wrestling with uncertainty about their recovery and the impacts in terms of heightened anxiety, confusion, and feelings of invalidation.
Wharewera-Mika, Cooper, Kool, et al.	Traumatology	2016	New Zealand	Psychologist (incl. clinical, neuro-)	The Flying Doctors	All HIC	Clinical Child Psychology and Psychiatry	***Caregivers' voices: The experiences of caregivers of children who sustained serious accidental and non-accidental head injury in early childhood***	To describe the experiences of caregivers of children who sustained a serious head injury (particularly non-accidental head injury) before the age of 2 years.	Mixed	TBI	New Zealand	Qualitative	Not reported	Family members, carers, signifcant others	21	Interviews (open, unstructured, in-depth, semi-structured, open ended)	Thematic analysis	Braun & Clarke (2006)	Not reported	The study's findings reveal the broad impact of serious childhood head injury on caregivers, specifically the significant distress and burden brought about through lack of information, challenges in accessing support and inconsistent care. Recommendations for developing a quality 'model of care' and improving ease of access to supports for caregivers are provided.
Løvstad, Solbrække, Kirkevold, et al.	Traumatology	2018	Norway	Not reported	Department of Research	All HIC	Brain Injury	***"It gets better. It can´t be worse than what we have been through." Family accounts of the minimally conscious state***	To explore family life when a family member is in a chronic minimally conscious state (MCS). Experiences with the health care system were also explored.	Unclear	TBI	Norway	Qualitative (inspired by narrative approaches)	Not reported	Family members, carers, signifcant others	5	Focus groups	Thematic analysis (inductive)	Braun & Clarke (2006)	Not reported	Three main themes emerged. Each family reflected on the challenges of maintaining family unity. They also revealed how they had dealt with the ambiguity and severity of the situation, including the communication with the health care system. Finally, they described their journeys back toward a normal, everyday family life.
Robertsen, Helseth and Førde	Traumatology	2021	Norway	MD/Physician	Division of emergencies and critical care, Department of research and development	All HIC	BMC Medical Ethics	***Inter-physician variability in strategies linked to treatment limitations after severe traumatic brain injury; proactivity or wait-and-see***	To seek insight into clinicians' strategies concerning unresolved prognostic uncertainty and their ethical reasoning on the issue of limitation of life-sustaining treatment in patients with minimal or no signs of neurological improvement after severe traumatic brain injury in the later trauma hospital phase.	Adult	TBI	Norway	Qualitative	Not reported	HCPs (inc. NSx)	18	Interviews (open, unstructured, in-depth, semi-structured, open ended)	Thematic analysis	Specified that they did not use a pre-defined theoretical framework.	Not reported	A divide between proactive and wait-and-see strategies emerged. Notwithstanding the hospital's strong team culture, inter-physician variability with regard to ethical reasoning and preferred strategies was exposed. All the physicians emphasized the importance of team—family interactions. Nevertheless, their strategies differed: (1) The proactive physicians were open to consider limitations of life-sustaining treatment when the prognosis was grim. They initiated ethical discussions, took leadership in clarification and deliberation processes regarding goals and options, saw themselves as guides for the families and believed in the necessity to prepare families for both best-case and worst-case scenarios. (2) The "wait-and-see" physicians preferred open-ended treatment (no limitations). Neurologically injured patients need time to uncover their true recovery potential, they argued. They often avoided talking to the family about dying or other worst-case scenarios during this phase.
Robertsen, Helseth, Laake, et al.	Traumatology	2019	Norway	MD/Physician	Department of Anesthesiology, Division of Emergencies and Critical Care, Department of Clinical Medicine	All HIC	Scandinavian Journal of Trauma, Resuscitation and Emergency Medicine	***Neurocritical care physicians' doubt about whether to withdraw life-sustaining treatment the first days after devastating brain injury: An interview study***	To explore physicians' doubt related to decisions to withhold or withdraw life sustaining treatment within the first 72 after devastating brain injury and to identify the strategies used to address doubt	Adult	TBI	Norway	Qualitative	Not reported	HCPs (inc. NSx)	18	Interviews (open, unstructured, in-depth, semi-structured, open ended)	Thematic analysis	Braun & Clarke (2008, 2014)	Not reported	All physicians described feelings of doubt. The degree of doubt and how they dealt with it varied. Common strategies used across specialties when dealing with uncertainty and doubt were: 1) provision of treatment trials 2) Using time as a coping strategy 3) Collegial counselling and interdisciplinary consensus seeking 4) Framing decisions as purely medical.
Slettebo, Caspari, Lohne, et al.	Traumatology	2009	Norway	RN/Nurse	Faculty of Nursing Education; Faculty of Health and Sport	All HIC	Journal of Advanced Nursing	***Dignity in the life of people with head injuries***	To determine how people who suffer from head injuries perceive respect for their dignity and to discover what patients mean by the concept of 'dignity'	Adult	TBI	Norway	Qualitative (descriptive)	Edlund's theoretical framework of Absolute Dignity & Relative dignity	Patients	14	Interviews (open, unstructured, in-depth, semi-structured, open ended)	Content analysis (three levels of interpretation)	Kvale (1997); Edlund (2002)	Not reported	Patients experienced their dignity as maintained when they were taken seriously, received appropriate information and were reality-oriented. They experienced their dignity as violated if they had been neglected or had encountered healthcare personnel who lacked knowledge, were skeptical about their stories, and where the patient experienced extra burden when they were mistrusted. The importance of adequate information was underscored. As interviewees said, head injuries do not show on the outside and people with head injuries do not have a high status in society.
Solvang, Heiaas, Romsland, et al.	Traumatology	2021	Norway	Sociologist	Oslo Metropolitan University	All HIC	Health	***The unexpected other: Challenges and strategies after acquired impairment***	This article addresses the intricate relations between the altered body, personal ambitions, and social surroundings in the first 2-3 years after an injury. The conceptual framework centers around the process of change, applying concepts such as the unexpected other and biographical reconstruction.	Adult	TBI; SCI; multi trauma	Norway	Qualitative	Biographical disruption	Patients	21	Interviews (open, unstructured, in-depth, semi-structured, open ended)	Thematic: narrative analysis	Reissman (2008); Braun & Clarke (2006)	Not reported	Consequences of the injury took place at a carnal level where fatigue is something completely different from becoming exhausted and where elimination of body waste takes place through practices novel to the injured person. Living with impairment also took place at the level of social interaction. Here, family relations, shame, and establishing a new identity seemed profoundly important. This article makes two novel contributions. First, it emphasizes more than previous studies do that the daily management of altered body functions is more important for independence and wellbeing. Second, the article identifies the narrative about the accident as an important issue for injured people to settle.

Sveen, Ostensjo, Laxe, et al.	Traumatology	2013	Norway	Not reported	Department of Physical Medicine and Rehabilitation	All HIC	Disability and Rehabilitation	***Problems in functioning after a mild traumatic brain injury within the ICF framework: The patient perspective using focus groups***	To describe problems in body functions, activities, and participation and the influence of environmental factors as experienced after mild traumatic brain injury (TBI), using the ICF framework. To compare our findings with the Brief and Comprehensive ICF Core Sets for TBI.	Adult	TBI	Norway	Qualitative	International Classification of Functioning Disability and Health (ICD framework	Patients	17	Focus groups	Other: Analyzed using the ICF	Kvale (1999)	Not reported	One-hundred and eight second-level categories derived from the interview text, showing a large diversity of TBI-related problems in functioning. Problems in cognitive and emotional functions, energy and drive, and in carrying out daily routine and work, were frequently reported. All ICF categories reported with high-to-moderate frequencies were present in the Brief ICF Core Set and 84% in the Comprehensive ICF Core Set. The reported environmental factors mainly concerned aspects of health and social security systems, social network and attitudes towards the injured person.
Brunner, Palmer, Togher, et al.	Traumatology	2019	Australia	Not reported	University of Technology	All HIC	Proceedings of the 52nd Hawaii International Conference on System Sciences	***Content analysis of tweets by people with Traumatic Brain Injury (TBI): Implications for rehabilitation and social media goals***	To determine the ways people with TBI use Twitter to communicate, the socio-linguistic features of cognitive-communication disability evident in their tweets, and any aspects of their use of Twitter that could inform the development of social media rehabilitation goals for people with TBI.	Adult	TBI	Not reported	Mixed methods (Dann's content classification; Computational analysis; Qualitative content analysis)	Not reported	Patients	6	Social Media Content: Twitter	Content analysis (Inductive, computational analysis)	Hemsley et al (2014)	Not reported	The results reflected that participants used Twitter for: (i) supporting others, including people with TBI; (ii) discussing society and culture, popular issues, news, and personal interests; (iii) connecting with others; (iv) sharing their experiences of life after TBI; (v) knowledge via exchanging information; and (vii) advocacy. 'Emotional expression', and 'connection' were common threads running across themes. Attending to the expressions of people with TBI on Twitter provides important insights into their lived experiences and could inform the development of user-centered cognitive-communication and social participation goals for people with TBI.
Delehanty and Kieren	Traumatology	1998	Canada	Psychologist (incl. clinical, neuro-)	not stated	All HIC	Journal of Cognitive Rehabilitation	***Family perceptions of health professionals in family problem solving after brain injury***	To determine the roles health care professionals played in family problem solving after brain injury	Adult	TBI	Not reported	Grounded theory	Not reported	Family members, carers, signifcant others	16	Mixed: Interviews (open, unstructured, in-depth, semi-structured, open ended) AND questionnaires AND a video-taped group problem-solving interaction	Grounded theory analysis/constant comparison/open, axial and/or selective coding	Glasser & Strauss, (1976), Struass (1987), Strauss and Corbin (1990)	Not reported	Family members' reflections on their experiences in family problem-solving after brain injury suggested that the roles health care professionals played in this relationship had both beneficial and limiting aspects. Five role themes are discussed: intrusive guide, alternative idea generator, boundary maintainer, family stability touchstone, and advocate.
Gauvin-Lepage	Traumatology	2022	Canada	RN/Nurse	University of Montreal and Research Center of the Sainte-Justine University Hospital	N/A	Brain Injury	***Traumatic Brain Injury in Adolescence and the Family Resilience Process: A Case Study***	To better understand the family resilience process following a severe traumatic brain injury during adolescence.	Adolescents	TBI	Not reported	Case study	Humanistic model of nursing care	Family members, carers, signifcant others	2	Interviews (open, unstructured, in-depth, semi-structured, open ended)	Content analysis	Miles, Huberman & Saldana (2013)	Not reported	The data analysis yielded six themes as well as four subthemes that illustrate this family's resilience process. The most important factors that emerged are (a) family characteristics (i.e., a fighter personality, cultural and spiritual beliefs, presence of hope, keeping a sense of humor), (b) support of family members, (c) support of friends, (d) practicing sports and leisure activities, (e) back-to-school support, and (f) feeling helpful to the adolescent.
Allen, Hevey, Carton, et al.	Traumatology	2021	Ireland	Not reported	School of Psychology	All HIC	Disability and rehabilitation	***Life is about "constant evolution": the experience of living with an acquired brain injury in individuals who report higher or lower posttraumatic growth***	To explore the experience of living with an Acquired Brain Injury (ABI) in individuals who report higher or lower posttraumatic growth (PTG).	Adult	Mixed ABI	Not reported	Multi-method (Questionnaires; qualitative comparative analysis)	Post-traumatic growth theory/critical realist	Patients	14	Interviews (open, unstructured, in-depth, semi-structured, open ended)	Reflexive thematic analysis	Braun & Clarke (2012, 2013)	Not reported	Four themes emerged. The first two themes: "In my mind I was fine" surviving in aftermath of acquiring a brain injury and The everyday as "derailing" capture the transition process from an initial rehabilitation state characterized by neuropsychological and avoidance coping, towards active rebuilding for PTG. Internal building blocks for PTG and Growing in the social world: "you need to have that social connection" elaborate on the internal (e.g., acceptance, integration of the pre and post-injury self) and external (e.g., social relationships) factors seen to facilitate or obstruct PTG.
Stansfield	Traumatology	1991	Not reported	RN/Nurse	Not stated	N/A	Axone	***Adaptation of family members of brain-injured patients: perceptions of the hospitalization event***	The family's perception of their experience during the first week of the patient's hospitalization	Adult	Unclear, neurotrauma and head injuries mentioned in abstract but not the main text	Not reported	Qualitative (descriptive)	Double ABCX model of family crisis	Family members, carers, signifcant others	17	Interviews (open, unstructured, in-depth, semi-structured, open ended)	Other: Generic-type and differentiation-type	Not reported	Not reported	The family's perceptions of the hospitalization experience included: first hearing the news, first seeing the patient, the roles of nurse and physicians, the hospital culture, the family's physical needs, their time perspective, the ambiguity of the situation, belonging to a community of sufferers and the family's expectations for the patient's recovery. The findings of this study underline the importance of nurses as supporters of these families in their adaptation to a life-threatening event.
Stanescu and Romer	Traumatology	2012	Romania	Not reported	National School of Political Studies and Public Administration	All HIC	Procedia	***What Are the Main Coping Strategies Used by Adolescents with Traumatic Brain Injured Parent? An Interpretative Phenomenological Analysis***	To integrate the varied range of emotions associated with parental illness, children will approach various coping strategies, directed either toward managing their inner world, or solving practical problems. The purpose of this qualitative study of adolescents having one parent with traumatic brain injury is to explore the children's coping process	Adult	TBI	Not reported	IPA	Not reported	Family members, carers, signifcant others	5	Observations	Phenomenological analysis	Smith & Osborn (2003); Smith (2004)	Not reported	The coping styles used cover close strategies, but also avoidance strategies such as distraction or resignation. The research brings a new insight on the topic using "first hand" information.
Al-Adawi, Al-Busaidi, Al-Adawi, et al.	Traumatology	2012	Oman	Not reported	Department of Behavioral Medicine	All HIC	SAGE Open	***Families coping with disability due to brain injury in Oman: Attribution to belief in spirit infestation and Ensorcellment***	To describe the reactions and adaptation of caregivers in Oman.	Adult	TBI	Oman.	Case study	Not reported	Family members, carers, signifcant others	6	Interviews (open, unstructured, in-depth, semi-structured, open ended)	IPA	Smith, Harre, and Van Langenhove (2001); Larkin, Watts, and Clifton (2006).	Not reported	The caregivers interpreted their relatives' predicament through the local idiom of distress involving belief in spirit infestation and ensorcellment.
Bajwa, Hamid, Iqbal, et al.	Traumatology	2021	Pakistan	Medical student	King Edward Medical University	All LMIC	Annals of King Edward Medical University Lahore Pakistan	***Changing Presentation of Traumatic Brain Injuries in a Tertiary Hospital Lahore Following Enforcement of Motorcycle Helmet Laws - A Mixed-Method Study***	To observe change in traumatic brain injury (TBI) presentation after enforcement of a motorcycle helmet law and to explore perspectives of stakeholders on road safety measures.	Mixed	TBI	Pakistan	Mixed methods	Not reported	Other: traffic police officers; neurosurgeons; motorcyclists	12	Mixed: Interviews (open, unstructured, in-depth, semi-structured, open ended) AND patient record review	Content analysis	Not reported	Not reported	After interviewing stakeholders, the main theme identified was "Enforcement of road safety measures remains partial and inconsistent". Changes in healthcare burden and challenges in implementing legislature were noted.

Hamarova, M. and Minarovicova, K. and Rac, I. and Leka, K. and Akimjak, A.	Traumatology	2024	Slovakia	Unclear/not stated	St. John Paul II. Institute of Missiology and Tropical Health,	All HIC	Acta Missiologica	***THE SIMILARITY OF ATTITUDES AND ACTIONS OF VINCENT DE PAUL AND LOUISE DE MARILLAC: SELECTED ASPECTS OF PERSON-CENTRED CARE DURING A PERIOD OF COUNSELLING IN A HOSPITAL ENVIRONMENT***	The aim of the research was to ascertain the support patients would require from social workers acting as therapists. The research question guiding the achievement of the research goal was as follows: What are the expectations of patients with traumatic brain injury regarding the provision of this support?	Adult	TBI	Slovakia, Poland, Serbia	Qualitative	Non stated	Patients	30	Interviews (semi-structured, open, in depth)	Other: Unclear	non stated	Not reported	Traumatic brain injury patients would welcome counselling from a social worker – therapist even in a hospital setting. They expect counselling to be provided with genuine respect and empathy.
Talbot, K. J. and Krüger, E. and Pillay, B. S.	Traumatology	2023	South Africa	Unclear/not stated	Department of Speech-Language Pathology and Audiology, Faculty of Humanities	All LMIC	African Journal of Disability	***Experiences of acquired brain injury one-month post-discharge from acute hospitalisation***	To describe perceived experiences of individuals with ABI, and their significant others, regarding rehabilitation services and returning to daily activities, one-month post-discharge from acute hospitalisation.	Adult	MIXED ABI; TBI	South Africa	Qualitative	Realism	Patients AND Families/carers/ Significant other	12	Interviews (semi-structured, open, in depth)	Thematic analysis	Braun and Clarke (2013)	Not reported	Six main themes emerged that best described participants' experiences; two of which were shared between individuals with ABI and their significant others (SO). Individuals with an ABI acknowledged recovery as their priority and highlighted the importance of patience. The need for counselling and additional support from healthcare professionals and peers arose. The SO expressed a need for written information, improved communication from healthcare professionals, and education regarding the implications of an ABI. The coronavirus disease 2019 (COVID-19) pandemic negatively influenced all participants' overall experiences, mainly because of termination of visiting hours. Psychosocial intervention would have been beneficial to all participants. Faith influenced most participants' attitudes towards recovery and adapting post-ABI.
Broodryk and Pretorius	Traumatology	2015	South Africa	Not reported	Department of Psychology	All LMIC	African journal of disability	***Initial experiences of family caregivers of survivors of a traumatic brain injury***	To explore the challenges that family caregivers face during the initial stages of recovery of a relative who has sustained a TBI.	Adult	TBI	South Africa	Qualitative	Not reported	Family members, carers, signifcant others	12	Interviews (open, unstructured, in-depth, semi-structured, open ended)	Thematic analysis	Braun & Clarke (2006)	Not reported	Three main themes that relate to the experiences of caregivers during the initial stages of their relative's recovery emerged through data analysis. These themes were: (1) 'shock' at hearing the news; (2) negative experiences in hospital; and (3) frustrating interactions with healthcare professionals. All the participants reported that the accident that caused their relative to sustain a TBI was a traumatic experience for them.
Chembeni and Nkomo	Traumatology	2017	South Africa	Post-graduate student	Postgraduate student, Department of Social Work	All LMIC	Social Work (South Africa)	***Challenges experienced by survivors of traumatic brain injuries and their families***	To determine the challenges experienced by survivors of traumatic brain injury (TBI) and their families in South Africa	Unclear	TBI	South Africa	Qualitative	Not reported	Patients AND Family members, carers, signifcant others	12	Interviews (open, in-depth, semi-structured, open ended)	Thematic: Content analysis	Creswell's process for qualitative data analysis in Schurink, Fouché & De Vos (2011)	Not reported	Four themes emerged: challenges experienced by TBI survivors and their families/caregivers; stigma and stereotypes associated with TBI or disabilities in communities; perceived roles of communities and the government to improve the lives of people with TBI; and effectiveness of the rehab services provided at Headway
Crewe-Brown, Stipinovich and Zsilavecz	Traumatology	2011	South Africa	Not reported	Department of Communication Pathology, University of Pretoria.	N/A	The South African journal of communication disorders. Die Suid-Afrikaanse tydskrif vir Kommunikasiea fwykings	***Communication after mild traumatic brain injury--a spouse's perspective***	To examine communication after MTBI from the perspective of the spouse.	Adult	TBI	South Africa	Case study	Not reported	Family members, carers, signifcant others	2	Interviews (open, unstructured, in-depth, semi-structured, open ended)	Discourse analysis	Jaworski & Coupland, (1999)	Not reported	The results show that both participants perceived changes in the communication of their spouse following the MTBI. The results further show that MTBI affected communication of the two individuals in different ways.
Mokhosi and Grieve	Traumatology	2004	South Africa	Not reported	Psychology Unit, School of Social Sciences	All LMIC	South African Journal of Psychology	***African families' perceptions of traumatic brain injury***	To explore the perceptions of traumatic brain injury (TBI) held by a group of 22 brain-injured individuals and their caregivers living in rural African communities.	Mixed	TBI	South Africa	Qualitative	Not reported	Patients AND Family members, carers, signifcant others	44	Interviews (open, unstructured, in-depth, semi-structured, open ended)	Thematic analysis	Not reported	Not reported	While there are similarities between these findings and those in other parts of the world regarding the cognitive, emotional, personality and behavioral changes experienced by brain-injured individuals, the perceived aetiology of brain injury differed according to the individuals' worldview. Most of the participants believed that the accidents considered in this study as well as the injuries they gave rise to were the result of witchcraft, ancestral anger, God's will or thwasa. The reactions of family members to caring for, and living with, a brain-injured person appeared to be influenced by specific social practices and local cultural beliefs. It is important to incorporate these beliefs into the rehabilitation process
Shaheed Soeker, Van Rensburg and Travill	Traumatology	2012	South Africa	Not reported	Department of Occupational Therapy	All LMIC	Work (Reading, Mass.)	***Individuals with traumatic brain injuries perceptions and experiences of returning to work in South Africa***	To identify the central concepts of a model that would facilitate the return to work process of individuals with brain injury. However for the purpose of this paper there will be a focus only on the barriers and facilitators that influence the return to work process.	Adult	TBI	South Africa	Phenomenology	Interpretive	Patients	10	Mixed: Interviews (open, in-depth, semi-structured, open ended) AND Observations	Other: comprehending, synthesizing (decontextualizing), theorizing and recontextualizing.	Morse & Field (2002)	Not reported	The results of the study revealed that the participants experienced a sense of loss of function after the brain injury, a fear of the future and loss of confidence in their worker roles. The participants also indicated that by means of adapting occupational routines an actively engaging in rehabilitation they developed confidence in their worker roles
Ahman, Saveman, Styrke, et al.	Traumatology	2013	Sweden	MD/Physician	Department of community medicine and rehabilitation	All HIC	Journal of rehabilitation medicine	***Long-term follow-up of patients with mild traumatic brain injury: a mixed-method study***	To characterize the long-term consequences of mild traumatic brain injury regarding post-concussion symptoms, post-traumatic stress, and quality of life; and to investigate differences between men and women.	Adult	TBI (concussion)	Sweden	Mixed methods	Not reported	Patients	10	Interviews (open, unstructured, in-depth, semi-structured, open ended)	Content analysis	Graneheim & Lundman (2004)	Not reported	The interviews revealed that some patients still had disabling post-concussion symptoms and consequences in many areas of life 11 years after the injury event.
Falk, von Wendt and Klang	Traumatology	2008	Sweden	Not reported	Department of Woman and Child Health, Neuropediatric Research Unit, karolinska institute	All HIC	Patient Education and Counseling	***Informational needs in families after their child's mild head injury***	To characterize familial information needs following Pediatric mild traumatic brain injury	Pediatric	TBI	Sweden	Survey	Not reported	Family members, carers, signifcant others	57	Open ended question (one)	Content analysis	Krippendorff (2004) and Weber (1990)	Not reported	This analysis revealed two types of needs, i.e., a need for information concerning the head injury itself and how to provide care, as well as a need for reassurance and support in sharing and coping with the emotional burden.<br><br>Despite differences in the severity of the child's head injury and requirement for hospitalization, all the families expressed the same informational needs but also the need for emotional support.

Hallberg, Hallberg, Johansson, et al.	Traumatology	2005	Sweden	Psychologist (incl. clinical, neuro-)	School of Social and Health Sciences,	All HIC	Scandinavian Journal of Caring Sciences	***Daily living with hyperacusis due to head injury 1 after a treatment program at the hearing clinic***	To gain a deeper understanding of the quality of daily living of persons with hyperacusis after a traumatic head injury 1 year after these persons had participated in a treatment program at a Swedish hearing clinic	Mixed	Traumatic brain injury (Hyperacusis secondary to head injury)	Sweden	Qualitative	Not reported	Patients	21	Interviews (open, unstructured, in-depth, semi-structured, open ended)	Grounded theory analysis/constant comparison/open, axial and/or selective coding	Glaser & Strauss (1967)	Not reported	Five emergent categories were labelled moderating vulnerability, awareness of restrictions, conditioned participation, structuring daily life and controlling mood changes. The core category, moderating vulnerability, describes the necessary balancing act between activity and recovery. The informants were aware of their new restrictions and managed daily life by structuring and planning each day in detail to minimize exposure to sensory stimuli. They had learned to prioritize their activities and, thereby, rationed their time. The evaluated program appears to have positive effects and facilitates patients' adjustment process to hyperacusis with relatively restricted costs for the society.
Högberg, Eriksson, Högberg, et al.	Traumatology	2020	Sweden	Not reported	Department of Women's and Children's Health	All HIC	PLoS ONE	***Parents' experiences of seeking health care and encountering allegations of shaken baby syndrome: A qualitative study***	To explore parents' experiences of seeking health care for their children and instead being accused by healthcare professionals of Shaken Baby Syndrome/Abusive Head Trauma (SBS/AHT), being reported to Social Services, undergoing judiciary processing, and the impact of these events on family (dis)integration.	Pediatric	TBI; Shaken baby syndrome	Sweden	Qualitative	Not reported	Family members, carers, signifcant others	12	Interviews (open, unstructured, in-depth, semi-structured, open ended)	Content analysis	Graneheim & Lundman (2004); Graneheim, Lindgren & Lundman (2017)	Not reported	An overarching theme 'Fighting for protection of their child after being trapped by doctors' and four sub-themes were developed to reflect the parents' experiences, reactions and interpretations. The first sub-theme, 'Being accused of injuring the child', illuminated the shock experienced when seeking care and instead being accused of being a perpetrator. The second, 'Chaos and powerlessness', refers to the emotions experienced when losing custody of the child and being caught in the enforcement of legislation by the authorities. The third, The unified fight against the doctors' verdict ,'illustrates the parents' fight for innocence, their worry for the lost child, and their support and resistance. The fourth, 'The wounded posttraumatic growth', describes the emotions, grief, panic, anxiety, and chal- lenges in reuniting the family, but also the parents' reflections on personal growth. Unani- mously, they had experienced the authorities' inability to reconsider, and expressed a deep mistrust of Pediatric care.
Jumisko, Lexell and Söderberg	Traumatology	2007	Sweden	RN/Nurse	Luleå University of Technology,	All HIC	Journal of Family Nursing	***Living with moderate or severe traumatic brain injury: The meaning of family members' experiences***	To elucidate the meaning of family members' experiences of living with an individual with moderate or severe TBI.	Adult	TBI	Sweden	Qualitative	Phenomenological hermeneutic	Family members, carers, signifcant others	8	Interviews (open, unstructured, in-depth, semi-structured, open ended)	Phenomenological analysis (hermeneutic interpretation)	Ricoeur (1976)	Not reported	A phenomenological hermeneutic interpretation (Ricouer, 1976) of the data reveal that family members struggle with their own suffering while showing compassion for the injured person. Their willingness to assume care for the injured person is derived from their feeling of natural love and the ethical demand to be responsi- ble for the other. Hope and natural love from close relatives, the afflicted person, and other family members give the family members strength. It is important that professionals pay more attention to the suffering of close relatives.
Jumisko, Lexell and Söderberg	Traumatology	2009	Sweden	RN/Nurse	Division of Nursing, Department of Health Science	All HIC	Journal of Clinical Nursing	***The meaning of feeling well in people with moderate or severe traumatic brain injury***	To elucidate the meaning of feeling well for people with moderate or severe traumatic brain injury.	Adult	TBI	Sweden	Qualitative	Phenomenological hermeneutic interpretation	Patients	8	Interviews (open, unstructured, in-depth, semi-structured, open ended)	Phenomenological analysis (hermeneutic interpretation)	Ricoeur (1976)	Not reported	The meaning of feeling well for people with moderate or severe traumatic brain injury was that the initially unfamiliar life with traumatic brain injury became familiar. This included finding strength, regaining control over everyday life, being close to someone and being good enough. People with traumatic brain injury felt well when they became reconciled with the circumstances of their life and created a new entity in that life, in which their complete health had been lost.
Jumisko, Lexell and Söderberg	Traumatology	2007	Sweden	RN/Nurse	Division of Nursing, Department of Health Science	All HIC	Disability and rehabilitation	***The experiences of treatment from other people as narrated by people with moderate or severe traumatic brain injury and their close relatives***	The aim of this study was to describe the treatment from other people as experienced by people with moderate or severe traumatic brain injury (TBI) and their close relatives.	Adult	TBI	Sweden	Qualitative	Naturalism	Patients AND Family members, carers, signifcant others	20	Interviews (open, unstructured, in-depth, semi-structured, open ended)	Thematic analysis	Sandelowski M 2000	Not reported	The results were described by the means of two themes: being excluded and missing confirmation. People with TBI and their close relatives had experiences of being avoided, being ruled by the authorities, being met with distrustfulness and being misjudged. They also searched for answers and longed for the right kind of help. People who listened to them, believed them and tried to understand and help them were appreciated.
Stenberg, Stalnacke and Saveman	Traumatology	2022	Sweden	Not reported	Department of Community Medicine and Rehabilitation, Rehabilitation Medicine	All HIC	Disability and Rehabilitation	***Family experiences up to seven years after a severe traumatic brain injury - family interviews***	To explore the experiences of being a family with one member suffering from severe traumatic brain injury (STBI) up to 7 years earlier through narrative family interviews.	Adult	TBI	Sweden	Qualitative (Interpretive)	Not reported	Patients AND Family members, carers, signifcant others	47	Interviews (open, unstructured, in-depth, semi-structured, open ended)	Content analysis	Graneheim, Lundman (2004)	Not reported	"From surviving STBI towards stability, through the unknown, into a new everyday life and a new future as a family" characterized the implicit message. The results revealed two categories both with three subcategories. The first category characterized the rapid change from a normal everyday life to one of uncertainty and finally to one of stability, and the second category described how it is to adapt as a family after STBI.
Trulsson, Johansson, Jansson, et al.	Traumatology	2003	Sweden	Sociologist and doctoral student	Nordic School of Public Health	All HIC	Journal of Health Psychology	***Struggling for a new self: In-depth interviews with 21 patients with hyperacusis after an acute head trauma***	To gain a better understanding of what suffering from hyperacusis means.	Adult	TBI	Sweden	Grounded theory	Not reported	Patients	21	Interviews (open, unstructured, in-depth, semi-structured, open ended)	Grounded theory analysis/constant comparison/open, axial and/or selective coding	Glaser & Strauss (1967); Strauss & Corbin (1998)	Not reported	As a consequence of the trauma, the patients became psychosocially vulnerable and suffered from a variety of symptoms: hypersensitivity to sounds, difficulties with concentrating and remembering, increased anxiety and sensitivity to stress. Structuring everyday life and using a variety of coping strategies was necessary for reducing life stress and symptoms in the process of adjusting to the changed life situation.
Engstrom and Soderberg	Traumatology	2011	Sweden	RN/Nurse	Department of Health Science	All HIC	Journal of Neuroscience Nursing	***Transition as experienced by close relatives of people with traumatic brain injury***	To describe the process of transition experienced by the close relatives of people with TBI.	Unclear	TBI	Sweden	Qualitative (Interpretive description)	Not reported	Family members, carers, signifcant others	5	Interviews (open, in-depth, semi-structured, open ended)	Qualitative interpretive method	Thorne, Kirkham, and Mac Donald-Emes (1997)	Not reported	The findings of the analysis are presented in 4 categories: the starting point of the transitions, transitions in pattern of daily life, transitions in relationship, and transitions in social life. The transitions of daily life for close relatives began suddenly as the person with TBI was injured unexpectedly. The relatives could feel lonely as former friends were gone or avoided them. How the person with TBI was met by other people strongly affected how the close relatives felt. Although they struggled to lessen the dependence of the person with TBI on them, they also felt anxious about how things would be if close relatives were no longer there for that person.
Siripituphum, Songwathana, Khupantavee, et al.	Traumatology	2020	Thailand	RN/Nurse	Faculty of Nursing	Mixed HIC/LMIC	Journal of Health Science and Medical Research	***Caring for Thai traumatic brain injury survivors in a transitional period: What are the barriers?***	To explore the situations and experiences of Thai traumatic brain injury (TBI) caregivers and nurses who care for TBI patients during their transition from hospital to home	Adult	TBI	Thailand	Qualitative (descriptive)	Not reported	Family members, carers, signifcant others AND HCPs	14	Mixed: Interviews (open, unstructured, in-depth, semi-structured, open ended) AND focus groups AND observations	Thematic: content analysis	Morse & Field (1995); Cavanagh (1997)	Not reported	nurses. These included: (1) inadequate discharge teaching information, (2) less time in caregiver's supervision and support, (3) lack of a comprehensive discharge plan, (4) lack of coordination and communication in follow-up care, (5) less confidence in providing care without support at home, and (6) poor availability of resources and time for consultation. Conclusion: Caregivers require more information and supportive care in order to enhance their adaptation in taking care of TBI patients in the long term. Developing a tele-nursing based caregiver transitional support program among TBI caregivers is suggested.

Thongsook, Sutawatnatcha, Saenprasarn	Traumatology	2022	Thailand	RN/Nurse	Faculty of nursing	All LMIC	Natural Volatiles & Essential Oils	***Experience of self care, family care and quality of life of the posttraumatic brain injury patient for hospital accreditation in a selected accreditation hospital***	To study self care, Family Care and quality of life of post traumatic Brain Injury patients.?	Adult	TBI	Thailand	Qualitative	Not reported	Patients AND Family members, carers, signifcant others	30	Interviews (open, unstructured, in-depth, semi-structured, open ended)	Colaizzi's data analysis	Colaizzi (1978)	Not reported	The results showed that Patients and their families define self-care for traumatic brain injury as obstacles in one's life and it affects the normal lifestyle that has changed from the original. unable to help himself as he used to in the early stages both in terms of eating exercise, rest, excretion, social interaction personal hygiene and stress management However, an interesting observation is that if there is a good hospital discharge plan, This will enable brain injury patients to be able to care for and manage themselves more. In terms of family care of the post traumatic brain injury patients were taken care of closely. In other words, each family was concerned about taking care of the post traumatic brain Injury patients in which the family members responsible for providing meals for them, maintaining their good health, handling the family, financial status and taking the post traumatic Brain Injury patients to friends and places
Marzolla, M. C. and Thielen, H. and Hurks, P. and Borghans, L. and van Heugten, C.	Traumatology	2024	The Netherlands	Other: Researcher MSc	Faculty of Psychology and Neuroscience and Limburg Brain Injury Centre	All HIC	Neuropsycholo gical Rehabilitation	***Qualitative data on triggers and coping of sensory hypersensitivity in acquired brain injury patients: A proposed model***	to identify triggers and coping strategies for sensory hypersensitivity towards incoming sensory information after ABI.	Adult	MIXED ABI	the Netherlands	Qualitative	Not explicitly stated	Patients	19	Interviews (semi-structured, open, in depth)	Thematic analysis (inductive)	Braun and Clarke (2006)	Not stated	Inductive thematic analysis revealed five themes: (1) A mismatch between resources and demands, (2) Altered experience of ordinary stimuli, (3) It affects all aspects of living, (4) Avoid, approach, accept, (5) It's highly heterogeneous. A model explaining the impact of triggers on subjective SHS after ABI is proposed, which states that SHS arises from a mismatch between the demands of a sensory environment (triggers) and the available biopsychosocial resources of an individual to meet these demands. The elicited SHS can affect and be affected by levels of fatigue, which limits the resources and creates a loop. Coping strategies can act on various stages of this model, i.e., to reduce the mismatch and potentially alter the loop. This model can contribute to the identification of mechanisms behind SHS in ABI patients and other populations, ultimately leading to evidence-based treatments.
Meyling, C. G. and Verschuren, O. and Rentinck, I. C. M. and van Driel, D. and te Slaa, E. and Engelbert, R. H. and Gorter, J. W.	Traumatology	2024	The Netherlands	Other: HCP with experience in paediatric physical therapy	UMC Utrecht Brain Center and Center of Excellence for Rehabilitation Medicine; Department of Paediatric Rehabilitation; Institute of Human Movement Studies, Master Program Physiotherapy Specialization Paediatric Physiotherapy	All HIC	Disability and Rehabilitation	***"Your brain can't wait": perspectives of children and adolescents with acquired brain injury and their parents on physical rehabilitation during the subacute phase***	To explore the perspectives of children and adolescents with acquired brain injury (ABI) and their parents with respect to physical rehabilitation during the subacute phase.	Paediatric	MIXED ABI	The Netherlands	Qualitative	Not explicitly stated	Patients AND Families/carers/ Significant other	26	Interviews (semi-structured, open, in depth)	Thematic analysis (Inductive)	Boeije (2009)	Not reported	Six themes were identified: 1) beliefs of physical rehabilitation, 2) content of physical rehabilitation, 3) tailored care, 4) impact of context, 5) communication and 6) transition. The importance of intensive physical practice was widely supported. The positive can-do mentality was emphasised to create an atmosphere of hope, meaning that every effort would be made to achieve maximum recovery. Intensive involvement of parents is considered essential during subacute rehabilitation including an open and mutual dialogue about the focus of rehabilitation, therapy goals and future participation in their own environment.
Kruithof, Traa, Karabatzakis, et al.	Traumatology	2018	The Netherlands	Not reported	Department Trauma TopCare	All HIC	Journal of Trauma Nursing	***Perceived Changes in Quality of Life in Trauma Patients: A Focus Group Study***	To gain more insight into changes in perceived QoL after trauma via a direct exploration of the patients' point of views.	Adult	Traumatic brain injury, lower extremity trauma, & other trauma	The Netherlands	Qualitative	Not reported	Patients	20	Focus groups	Grounded theory analysis/consta nt comparison/op en, axial and/or selective coding	Boeije (2010) Glaser & Strauss (1967)	Not reported	In the first month post trauma, physical limitations, independency, pain, and anxiety predominated. Later, patients experienced problems with acceptance. The patients' feelings of the need to have control over their own situation, their own expectations, and a social network were related to QoL. Compared with the other patient groups, TBI patients reported more psychosocial consequences, and elderly patients reported more difficulties in performing (social) activities. Quality of health care was considered an important aspect in the patients' perceived QoL, and adequate aftercare was missed according to the patients.
Kisembo, H. N. and Malumba, R. and Sematimba, H. and Ankunda, R. and Nalweyiso, I. D. and Malwadde, E. K. and Rutebemberwa, E. and Kasasa, S. and Salama, D. H. and Kawooya, M. G.	Traumatology	2024	Uganda	Unclear/not stated	Makerere University, College of Health Sciences, School of Medicine; Department of Radiology	All LMIC	African Journal of Emergency Medicine	***Understanding the factors that influence CT utilization for mild traumatic brain injury in a low resource setting - a qualitative study using the Theoretical Domains Framework***	The primary objective was to explore imaging referrers' beliefs about factors influencing CT utilization in mTBIs using Theoretical Domains Framework in Uganda. Differences in the factors influencing computed tomography scan ordering behavior across specialties, levels of experience, and hospital category were also explored.	Unclear	mTBI	Uganda	Qualitative	Theoretical domains framework	HCPs (inc. NSx)	11	Interviews (semi-structured, open, in depth)	Thematic analysis	non stated	Not reported	Identified factors within skills domain involved IRs' clinical assessment and decision-making abilities, while beliefs about capabilities and consequences encompassed their confidence in diagnostic abilities and perceptions of CTS risks and benefits. The environmental context and resources domain addressed the availability of CT scanners and financial constraints. The knowledge domain elicited IRs' understanding of clinical guidelines and evidence-based practices while social influences considered peer influence and institutional culture. For memory, attention & decision processes domain, IRs adherence to guidelines and intentions to order CT scans were cited.

Petitt, Z. and Ordonez, Y. T. and Agwu, C. and Ott, M. and Shakir, M. and Mullikin, A. A. and Davis, J. and Khalafallah, A. M. and Tang, A. and Shalita, C. and Ssembatya, J. M. and Deng, D. D. and Headley, J. and Obiga, O. and Haglund, M. M. and Fuller, A. T.	Traumatology	2024	USA	Researcher	Division of Global Neurosurgery and Neurology; Duke University Global Health Institute	Mixed HIC/LMIC	Plos One	***Exploring the feasibility of pupillometry training and perceptions of potential use for intracranial pressure monitoring in Uganda: A mixed methods study***	to assess the feasibility of pupillometry for noninvasive ICP monitoring for patients with TBI.	N/A	TBI	Uganda	Mixed methods,	Not explicitly stated	HCPs (Inc NSx)	19	Interviews (semi-structured, open, in depth)	Framework analysis	Gale et al. (2013)	Not reported	Most participants (79%) reported a positive perception of pupillometry. Participants described the value of pupillometry in the care of patients during examination, monitoring, and intervention delivery. Commonly discussed concerns included pupillometry's cost, understanding, and maintenance needs. Perceived implementation challenges included device availability and contraindications for use. Participants suggested offering continued education and engaging hospital leadership as implementation strategies. During training, the average learning time was 13.5 minutes (IQR 3.5), and the measurement time was 50.6 seconds (IQR 11.8). Paired t-tests to evaluate accuracy showed no statistically significant difference in comparison measurements.
Nwosu, Spears, Pate, et al.	Traumatology	2020	USA	MD/Physician	School of Medicine	All HIC	Annals of Global Health	***Influence of caretakers' health literacy on delays to traumatic brain injury care in Uganda***	To determine the factors that impact TBI patient caretakers' health literacy and examine how these factors influence delays in care.	Unclear	TBI	Uganda	Qualitative	Not reported	Family members, carers, signifcant others	27	Interviews (open, unstructured, in-depth, semi-structured, open ended)	Content analysis (Codes assigned to three delays framework by Thaddeus and Maine, (1994)	Not reported	COREQ	The main health literacy themes identified were Extrinsic, Intrinsic and Health System Factors. Nine sub-themes were identified: Government Support, Community Support, Financial Burdens, Lack of Medical Resources, Access to Health Information, Physician Support, Emotional Challenges, Navigational Skills, and Understanding of Health Information. These components were found to influence the delays to care to varying degrees. Financial Burdens, Government Support, Emotional Challenges, Physician Support and Lack of Medical Resources were recurring factors across the three delays.
Anderson, E. and White, A. and Hardwicke, J.	Traumatology	2022	UK	Other: Qualitative researcher	School of Sport, Health and Community	All HIC	Behavioral Sciences	***A Qualitative Exploration of Parents' Perceptions of Risk in Youth Contact Rugby***	to explore the perceptions of risk among parents who enrol their children in contact rugby, and how theyunderstand and manage this risk.	Paediatric	mTBI	UK	Qualitative	Non stated	Family/Carer/Significant other	34	Interviews (semi-structured, open, in depth)	Thematic analysis	Braun and Clark (2016)	SRQR	A thematic analysis of data suggests that parents used two primary cognitive strategies to process the risk they consented to with their children's participation in rugby; (1) minimalizing rugby risk to be equivalent to less injurious sports; and (2) elevating physical and social advantages above what they think other sports are capable of providing.
Benn, Y. and Jayes, M. and Casassus, M. and Williams, M. and Jenkinson, C. and McGowan, E. and Conroy, P.	Traumatology	2023	UK	Other: Psychology and Speech and Language Therapy researchers	Department of Psychology	All HIC	Neuropsychological Rehabilitation	***A qualitative study into the experience of living with acalculia after stroke and other forms of acquired brain injury***	To explore the impact of acalculia on the lives of stroke and other forms of brain injury survivors with acalculia and their carers	Adult	Mixed ABI	UK	Qualitative descriptive	interpretivist framework	Patients AND Families/carers/ Significant other	23	Interviews (semi-structured, open, in depth)	Thematic analysis (Inductive and deductive descriptive thematic analysis)	Braun and Clark (2006)	Not reported	Three main themes were identified: Awareness and Diagnosis; Emotional and Practical Impact (independence); Support, Coping Strategies and Selftraining. Participants and carers repeatedly referred to the lack of awareness and treatment for acalculia and the impact acalculia has had on their lives and independence. Practical impacts included managing money, making appointments, using timetables, organizing social activities and employment, and managing medication
Di Basilio, D. and King, L. and Lloyd, S. and Michael, P. and Shardlow, M.	Traumatology	2024	UK	Unclear/not stated	Division of Health Research, School of Health and Medicine	All HIC	Frontiers in Digital Health	***Asking questions that are "close to the bone": integrating thematic analysis and natural language processing to explore the experiences of people with traumatic brain injuries engaging with patient-reported outcome measures***	to explore the views, opinions and experiences of completing PROMs in a sample of individuals with TBIs	Adult	TBI	UK	Qualitative	Non stated	Patients	16	Interviews (semi-structured, open, in depth)	Thematic analysis (inductive) AND Sentiment and emotion analysis	Braun and Clarke (2017)	Not reported	The TA of the data revealed six key themes regarding the experiences of individuals with TBIs in completing PROMs. Participants expressed varying levels of understanding and engagement with PROMs, with factors such as cognitive impairments and communication difficulties influencing their experiences. Additionally, insightful suggestions emerged on the barriers to the completion of PROMs, the factors facilitating it, and the suggestions for improving their contents and delivery methods. The sentiment analyses performed using NLP techniques allowed for the retrieval of the general sentimental and emotional "tones" in the participants' narratives of their experiences with PROMs, which were mainly characterised by low positive sentiment connotations. Although mostly neutral, participants' narratives also revealed the presence of emotions such as fear and, to a lesser extent, anger
Gamgee, J. and Runacres, J. and Norman, A. and Pradhan, P.	Traumatology	2023	UK	Unclear/not stated	School of Psychology,	All HIC	Family Journal	***The Secondary Impact of Mild Traumatic Brain Injury: An Interpretative Phenomenological Analysis of the Experiences of Family Members***	To explore the experiences of family members of people with mTBI symptoms that persist beyond 3 months, to investigate the importance of giving information to family members through analyzing their lived experience, and to undertake a comparative analysis between the findings of the original, TBI-focussed Townshend and Norman (2018) study and the current mTBI-focussed one.	Adult	mTBI	UK	Interpretive phenomenological analysis (IPA)	Hermeneutic realism, and an interpretative epistemology	Patients	4	Interviews (semi-structured, open, in depth)	Interpretive phenomenological analysis	Smith and Osborn (2008)	Not reported	Following an interpretative phenomenological analysis (IPA), three superordinate themes were identified: (1) Going round in circles, (2) The second secondary impact, and (3) Dialogue with myself. Findings indicate that families of people with mTBI, with symptoms beyond 3 months, may experience many of the same challenges as families of people with moderate or severe TBI, albeit at a lesser intensity
Hudson, C. and Radford, K. and Kettlewell, J.	Traumatology	2022	UK	Other: Masters student	Centre for Rehabilitation and Ageing Research	All HIC	International Journal of Environmental Research and Public Health	***A Qualitative Study to Understand the Impact of Caring for Traumatic Injury Survivors***	To explore the impact of caring on family and caregiver finances, employment, social life, and psychological wellbeing	Unclear	TBI	UK	Qualitative	The Roy Adaptation Model; Interantional Classification of Functioning, Disability, and Health	Family/Carer/Significant other	10	Interviews (semi-structured, open, in depth)	Thematic analysis	Braun and Clarke (2006)	Not reported	Key themes included (1) financial im- pact/employment issues, (2) relationships and support and (3) psychological impact. Most carers did not receive professional support with daily care post-discharge. Carers' employers responded posi- tively, supporting them even after extensive leave. Carers received inconsistent communication whilst visiting trauma survivors in hospital; carers with healthcare experience were favoured. Navigating and receiving benefits was complex. Some carers found it difficult to accept the trauma survivor's injury, whilst others focused on achieving goals.

Smith, R. M. and Burgess, C. and Tahtis, V. and Marsden, J. and Seemungal, B. M.	Traumatology	2023	UK	Unclear/not stated	Brain and Vestibular Group, Centre for Vestibular Neurology	All HIC	Bmj Open	***Why are patients with acute traumatic brain injury not routinely assessed or treated for vestibular dysfunction in the UK? A qualitative study***	to identify and explore any healthcare professional barriers or facilitators to managing vestibular dysfunction in aTBI.	Adult	TBI and vestibular dysfunction	UK	Qualitative	Not explicitly stated	HCPs (Inc NSx)	28	Interviews (semi-structured, open, in depth)	Framework approach	Gale et al. (2013)	COREQ	Vestibular assessment and treatment were not routinely undertaken by trauma ward staff. Uncertainty regarding responsibility for vestibular management on the trauma ward was perceived to lead to gaps in patient care. Interestingly, the term dizziness was sometimes perceived as an 'invisible' and vague phenomenon, leading to difficulties identifying or 'proving' dizziness and a tendency for making non-specific diagnoses. Barriers to routine assessment and treatment included limited knowledge and skills, a lack of local or national guidelines, insufficient training and concerns regarding the practical aspects of managing vestibular dysfunction. Of current trauma ward staff, therapists were identified as appropriate healthcare professionals to adopt new behaviours regarding management of a common form of vestibular dysfunction (benign paroxysmal positional vertigo). Strategies to support this behaviour change include heightened clarity around role, implementation of local or national guidelines, improved access to training and multidisciplinary support from experts in vestibular dysfunction.
Sparkes, A. C.	Traumatology	2023	UK	Researcher	Carnegie School of Sport	N/A	Qualitative Research in Sport, Exercise and Health	***The second I got the phone call, everything changed.' Exploring the temporal experiences of the spouses and partners of spinal cord injured sportsmen***	to explore the experiences of three women whose male spouses/partners have become disabled due to a SCI received whilst playing rugby	Adult	SCI	UK	Qualitative	Constructionism	Family/Carer/Significant other	5	Interviews (semi-structured, open, in depth)	Thematic narrative analysis	Riessman (2008)	Not reported	A thematic narrative analysis revealed how this event instigates a temporal-relational disruption that catapults these women into living in, by and through different types of time that operate in a multi-dimensional manner to shape how they construct their identities and come to understand themselves and others with a past, and a present, that has consequences for their future. The implications of this process for health care professionals in supporting those who face similar sets of circumstance are considered.
Stubbs, D. J. and Khanna, S. and Davies, B. M. and Vivian, M. E. and Bashford, T. and Adatia, K. and Chen, P. and Clarkson, P. J. and McGlennan, C. and Indurawage, L. and Patel, M. and Tyagunenko, R. and Burnstein, R. and Menon, D. K. and Hutchinson, P. J. and Joannides, A. and Team, Senior- C.	Traumatology	2024	UK	Unclear/not stated	Division of Anaesthesia	All HIC	Age and Ageing	***Challenges and patient outcomes in chronic subdural haematoma at the level of a regional care system A multi-centre, mixed-methods study from the East of England***	to clarify patient outcome in non-specialist centres following NSU discharge for cSDH surgery and to understand key system challenges.	Adult	Chronic subdural haematoma	UK	Mixed methods,	Not explicitly stated	Patients AND clinicians	250 surveys; 14 workshop participants	Mixed: Survey and focussed workshop discussions	Thematic analysis	Not stated	Not reported	Data on 381 patients referred for cSDH surgery from six centres was reviewed. One hundred and fifty-six (41%) patients were repatriated following surgery. Sixty-one (39%) of those repatriated suffered an inpatient complication (new infection, troponin rise or renal injury) following NSU discharge, with 58 requiring institutional discharge or new care. Surveys for staff (n = 42) and patients (n = 209) identified that resourcing, communication, and inter-hospital distance posed care challenges. This was corroborated through workshop discussions with stakeholders from two institutions.
Ankrett, Smithson, Limond, et al.	Traumatology	2022	UK	Not reported	College of Life and Environmental Sciences	All HIC	Neuropsychological Rehabilitation	***Understanding and supporting peer relationships in adolescents with acquired brain injury: A stakeholder engagement study***	(a) to develop a collaborative understanding of peer relationships for adolescents with ABI; and (b) to seek the views of adolescents and other key stakeholders on what might be needed to improve them, and what the intervention goals might be.	Adolescents	Mixed ABI	UK	Qualitative	Social constructionist position, intervention mapping framework	Patients AND Family members, carers, signifcant others AND HCPs	16	Mixed: Focus groups AND individual interviews	Thematic analysis	Braun & Clarke (2006, 2013)	Not reported	The analysis yielded 11 themes, grouped into two domains. The first, understanding peer relationship difficulties, included themes from “exclusion and a need to belong”, to “loss of past self”. The second, supporting peer relationships, comprised themes of “building understanding” and “meaningful social connection”, amongst others. A logic model of stakeholder experiences of peer relationship difficulties was constructed. Difficulties with peers can increase vulnerability to feelings of loneliness, shame, and hopelessness for adolescents post-ABI. Stakeholders described that a meaningful intervention would be multi-layered, targeting change within the adolescent’s environment and within the adolescent themselves. The presented logic model provides a framework for future intervention development.
Bodley-Scott and Riley	Traumatology	2015	UK	Not reported	School of Psychology	All HIC	Brain Impairment	***How Partners Experience Personality Change after Traumatic Brain Injury-Its Impact on Their Emotions and their Relationship***	To explore how spouses/partners experience social, emotional and behavioral changes in persons following traumatic brain injury (TBI), with a particular focus on their emotional impact and the effect on the couple relationship.	Adult	TBI	UK	IPA	Phenomenological	Family members, carers, signifcant others	5	Interviews (open, unstructured, in-depth, semi-structured, open ended)	IPA	Smith et al (2009)	Not reported	Themes describe the direct emotional impact of living with the changes as well as the emotional impact of attempts to manage and make sense of the changes (identity change, managing the changes and making sense of the changes). The impact on the couple relationship is described under the themes of feeling love and receiving love. Changes led three of the participants to experience their partner as having been replaced by a new person; they actively disliked this new person; they felt unable to love the new person in the same way as the old person; and their love was defined in terms of a caring relationship, rather than a spousal relationship.
Braine	Traumatology	2011	UK	RN/Nurse	lecturer at the School of Nursing and Midwifery	N/A	Journal of Neuroscience Nursing	***The experience of living with a family member with challenging behavior post acquired brain injury***	To elucidate the lived experience of challenging behavior among family members of persons with ABI and to describe these experiences to make them visible to others.	Mixed	Mixed ABI	UK	Phenomenology (Descriptive)	Phenomenological	Family members, carers, signifcant others	5	Interviews (open, unstructured, in-depth, semi-structured, open ended)	Phenomenological analysis	Giorgi (1985), Colaizzi (1978)	Not reported	Analysis and descriptions from the five participants revealed seven interrelated themes; one theme described the challenging behaviors of the people with ABI, and six themes described the experiences of the family members (emotional turmoil that these behaviors engendered, a profound sense of loss, concerns for their future and for the future of the person with ABI, a sense of loneliness, the effect on family functioning, and the family members' coping and adapting to the behaviors).
Brewin and Lewis	Traumatology	2001	UK	Occupational Therapist	West Park Rehabilitation Unit	All HIC	International Journal of Therapy and Rehabilitation	***Patients' perspectives of cognitive deficits after head injury***	To investigate whether cognitive deficits were experienced by patients with head injuries who are commonly discharged from hospital following a short period of observation, and second, to establish, through the patients' own experiences, how these problems impact on everyday activities.	Adult	TBI	UK	Phenomenology	Not reported	Patients	9	Mixed: Interviews (open, unstructured, in-depth, semi-structured, open ended) AND questionnaires	Other: Hycner's framework	Hycner's framework (1985)	Not reported	Six themes emerged which were common to all or most of the participants interviewed. These themes are described incorporating the participants' own words where possible to express the essence of the experiences. These are consistent with the clinical manifestations of cognitive deficits as experienced by the patients: The impact of cognitive deficits on work activities; The impact of cognitive deficits on communication; The impact of cognitive deficits on social activities; The impact of cognitive deficits on domestic activities; The impact of cognitive deficits on driving and using transport

Brunger, Ogden, Malia, et al.	Traumatology	2014	UK	Not reported	Department of Psychology	All HIC	Brain Injury	***Adjusting to persistent post-concussive symptoms following mild traumatic brain injury and subsequent psycho-educational intervention: A qualitative analysis in military personnel***	(i) to explore how military personnel with mTBI experience their injury and subsequent symptom presentation; and (ii) to assess the impact of an evidence-based psychoeducational intervention (Phase 2) for persistent PCS from the perspective of its patients.	Adult	TBI (veterans)	UK	Qualitative	Critical realist	Patients	16	Interviews (open, unstructured, in-depth, semi-structured, open ended)	Thematic analysis (inductive)	Braun & Clarke (2006)	Not reported	Participants described their mTBI in terms of: (1) onset; (2) subsequent symptom experience; (3) recovery; and (4) acceptance. All participants reported a significant degree of confusion and chaos in the aftermath of their traumatic event. These themes highlighted how, following enrolment onto the Phase 2 intervention at DMRC Headley Court, participants reported being (largely) able to manage PPCS. Further, many reported acceptance of their condition and described how they had managed to re-establish a sense of order.
Clark, Stedmon and Margison	Traumatology	2008	UK	Psychologist (incl. clinical, neuro-)	Cornwall Partnership Trust	All HIC	Clinical Child Psychology and Psychiatry	***An exploration of the experience of mothers whose children sustain traumatic brain injury (TBI) and their families***	To describe and explore the lived experience of mothers whose children, aged 0-16 years, have sustained a traumatic brain injury (TBI).	Pediatric	TBI	UK	IPA	Interpretative lens	Family members, carers, signifcant others	10	Interviews (open, unstructured, in-depth, semi-structured, open ended)	IPA	Smith and Osborn (2003)	Not reported	The analysis generated themes of 'changes to and loss of the past child', 'effects on mother's health', 'mother's process of coping and support', 'changed roles', 'effects on the whole family' and 'contact with services'.
Conneeley	Traumatology	2012	UK	occupational therapist	Coventry University, Faculty of Health and Life Sciences	N/A	Brain Impairment	***Transitions and brain injury: A qualitative study exploring the journey of people with traumatic brain injury***	To explore transitions from hospital to the home over a period of one year.	Adult	TBI	UK	Phenomenology	Interpretive/ sociological paradigm,	Patients AND Family members, carers, signifcant other AND HCPs	50	Interviews (open, unstructured, in-depth, semi-structured, open ended)	Thematic analysis	Braun & Clarke, (2006)	Not reported	Themes identified within the data included returning home, getting back to normal, moving forward and the role of rehabilitation in the transitional period. Further subthemes were also identified including issues of life-course disruption, self-identity, status and reconstruction.
Conneeley	Traumatology	2003	UK	occupational therapist	Coventry University, Faculty of Health and Life Sciences	N/A	British Journal of Occupational Therapy	***Quality of life and traumatic brain injury: A one-year longitudinal qualitative study***	To gain an understanding of the experience of quality of life by those affected by traumatic brain injury.	Adult	TBI	UK	Phenomenology	Not reported	Patients AND Family members, carers, signifcant other AND HCPs	36	Interviews (open, unstructured, in-depth, semi-structured, open ended)	Other: themes and categories formed to describe the data	Not reported	Not reported	When the data were Analyzed, several factors involved in the subjective experience of quality of life were identified. These included a sense of wellbeing, functional status, personal autonomy and the need for the acceptance of disability.
Conneeley	Traumatology	2003	UK	Occupational therapist	Coventry University, Faculty of Health and Life Sciences	N/A	British Journal of Occupational Therapy	***Social integration following traumatic brain injury and rehabilitation***	To examine the issues involved in social integration for those affected by traumatic brain injury, following a period of rehabilitation.	Adult	TBI	UK	Qualitative	Not reported	Patients AND Family members, carers, signifcant other AND HCPs	36	Interviews (open, unstructured, in-depth, semi-structured, open ended)	Other: themes and categories formed to describe the data	Polit et al (2001)	Not reported	When the data were Analyzed at the time of the final interview, two respondents reported social isolation. Although many others felt that the level of social contact was that of their choice, several issues were discussed that affected social relationships. These included the impact of impairments, the social response of others and the fact that social networks change naturally over time irrespective of injury or disability. When the data were considered from a sociological perspective, the themes of self-identity, master status and stranger status emerged.
Deb, Bryant, Morris, et al.	Traumatology	2007	UK	Not reported	Division of Neuroscience, Department of Psychiatry	All HIC	Neuropsychiatric Disease and Treatment	***Development and psychometric properties of the Carer - Head Injury Neurobehavioral Assessment Scale (C-HINAS) and the Carer - Head Injury Participation Scale (C-HIPS): Patient and family determined outcome scales***	Develop and assess the psychometric properties of the Carer - Head Injury Participation Scale (C-HIPS) and its biggest factor the Carer - Head Injury Neurobehavioral Assessment Scale (C-HINAS). Furthermore, the aim was to examine the inter-informant reliability by comparing the self-reports of individuals with traumatic brain injury (TBI) with the carer reports on the C-HIPS and the C-HINAS.	Adult	TBI	UK	Mixed methods (Qualitative; Questionnaire)	Not reported	Patients AND Family members, carers, signifcant others	59	Mixed: Interviews (open, unstructured, in-depth, semi-structured, open ended) AND field testing of questionnaire	Other: coding process was akin to that described by Strauss and Corbin	Strauss and Corbin, (1990)	Not reported	Results: All individual 49 items of the C-HIPS and their total score showed good test-retest reliability (0.95) and internal consistency (0.95). Comparisons with the MPAI-3 and GOSE found a good correlation with the MPAI-3 (0.7) and a moderate negative correlation with the GOSE (-0.6). Factor analysis of these items extracted a 4-factor structure which represented the domains 'Emotion/Behavior' (C-FUNAS),'Independence/ Community Living', 'Cognition', and 'Physical'. The C-HINAS showed good internal consistency (0.92), test-retest reliability (0.93), and concurrent validity with one MPAI subscale (0.7). Assessment of inter-informant reliability revealed good correspondence between the reports of the patients and the carers for both the C-HIPS (0.83) and the C-HINAS (0.82).
Gilworth, Eyres, Carey, et al.	Traumatology	2008	UK	Not reported	Academic Unit of Musculoskeletal and Rehabilitation Medicine	All HIC	Journal of Rehabilitation Medicine	***Working with a brain injury: Personal experiences of returning to work following a mild or moderate brain injury***	To explore expectations and experiences of the return to work process through individual interviews, and to gain further insight into the experiences of those who had not been able to return, something which has not previously been reported in any detail.	Adult	TBI	UK	Qualitative	Not reported	Patients	33	Interviews (open, unstructured, in-depth, semi-structured, open ended)	Thematic analysis	Weber (1990)	Not reported	Key emerging issues for participants were the invisibility of their injury, continuing symptoms affecting their ability to do their job and lack of advice and guidance on returning to work. Return to work support systems were considered to be poorly coordinated and managed.
Gosling and Oddy	Traumatology	1999	UK	Not reported	Pain Management Unit	All HIC	Brain Injury	***Rearranged marriages: Marital relationships after head injury***	To explore sexual relationships following head injury following from the point of view of the non-injured spouse.	Adult	TBI	UK	Mixed methods	Not reported	Patients AND Family members, carers, signifcant others	36	Mixed: Interviews (open, unstructured, in-depth, semi-structured, open ended) AND questionnaires	Thematic analysis	Henwood & Pidgeon (1995)	Not reported	The female partners reported both marital and sexual satisfaction as lower following injury. They rated their current marital satisfaction as significantly less than their brain injured partners. The quantitative part of the study revealed major role changes experienced by the women, with many comparing their new role to that of a parent with total decision making responsibility. The incompatibility of this role with that of sexual partner was mentioned by many. A tendency for the males to express gratitude but not to communicate their feelings was described by many women. Most women were resigned to the expectation that there would be little change in the future and, for most, the only positive aspect of the relationship was a sense of commitment and continuing companionship.
Grayson, Brady, Togher, et al.	Traumatology	2021	UK	Speech pathologist	NMAHP Research Unit; UK Community Brain Injury Team	All HIC	Brain Injury	***The impact of cognitive-communication difficulties following traumatic brain injury on the family; a qualitative, focus group study***	To identify how families experience cognitive-communication difficulties following Traumatic Brain Injury (TBI).	Adult	TBI	UK	Qualitative	Not reported	Family members, carers, signifcant others	15	Focus groups	Thematic analysis	Braun & Clarke (2006)	COREQ	Cognitive-communication difficulties were found to impact upon family functioning and psychological wellbeing for several years post-injury. Changes to social cognition, insight and the "filter switch" of the person following TBI were key areas of distress. Participants highlighted the need for information about communication changes to be provided at several time points post-injury. The need for peer support from other families with experience of cognitive-communication difficulties was also identified.

Kirk, Fallon, Fraser, et al.	Traumatology	2015	UK	Not reported	School of Nursing Midwifery and Social Work	All HIC	Child: Care, Health and Development	***Supporting parents following childhood traumatic brain injury: A qualitative study to examine information and emotional support needs across key care transitions***	To examine parents' experiences and support needs following a childhood TBI from the time of the accident to their child's discharge home.	Pediatric	TBI	UK	Qualitative	Not reported	Family members, carers, signifcant others	29	Interviews (open, unstructured, in-depth, semi-structured, open ended)	Thematic analysis	Ritchie et al. (2003)	Not reported	Parents had unmet information and emotional support needs across the care trajectory from the time of the accident to their child's return home. Information needs related to the impact of the TBI on their child; current and future treatment/rehabilitation plans; helping their child and managing their behavior; accessing services/support. They lacked information and support for care transitions. In different settings parents faced particular barriers to having their information needs met. Parents' felt they needed emotional support in coming to terms with witnessing the accident and the loss of their former child. Lack of community support related not only to service availability but to a general lack of understanding of the impact of TBI on children, particularly when this was invisible. Overall parents felt unsupported in coping with children's behavioral and psychological difficulties.
Makela	Traumatology	2017	UK	MD/Physician	Not reported	N/A	Subjectivity	***"They brought you back to the fact you're not the same": Sense of self after traumatic brain injury***	This paper considers contexts following traumatic brain injury, exploring what may be at stake when dominant expectations predict a 'lost' or 'broken' self.	Adult	TBI	UK	Narrative inquiry	Social constructivist paradigm	Patients AND Family members, carers, signifcant others	2	Interviews (open, in-depth, semi-structured, open ended)	Narrative analysis (inductive and holistic)	Not reported	Not reported	The power relations portrayed confront this man's narrative attempts to align his present and pre-injury self, including standard assessments delineating change, administered by healthcare professionals. I consider a need for greater attention to interaction-generated disruption to sense of self, within contemporary conceptualizations of 'person-centered care'.
McMullan, Retzer, Slade, et al.	Traumatology	2020	UK	Not reported	Center for Patient Reported Outcomes Research	All HIC	Brain Injury	***Care providers' and patients' attitudes toward using electronic-patient reported outcomes to support patients with traumatic brain injury: a qualitative study (PRiORiTy)***	To (a) identify residual symptoms and deficits resulting from a traumatic brain injury (TBI) and impact on patients' and their families' quality of life; (b) explore views and experience of care providers, researchers, patients, and carers of using PROMs; and (c) explore their attitudes toward reporting symptoms and impacts on an electronic platform.	Adult	TBI	UK	Qualitative (descriptive)	Not reported	Patients AND Family members, carers, signifcant others AND HCPs AND non-clinical providers AND researchers	28	Interviews (open, unstructured, in-depth, semi-structured, open ended)	Thematic analysis	Not reported	COREQ	Symptoms and long-term impacts of TBI included cognitive problems, difficulties functioning, anxiety, and depression. PROMs were seen as improving knowledge of residual symptoms and their impact post-TBI but not always accurately reflecting patients' residual problems. Challenges to completing PROMs were cognitive impairment and lack of insight into condition. Perceived advantages of an electronic platform included easy data collection; flexibility; improving workflow; and the ability to send/ receive feedback and reminders easily. Suggested features of an electronic platform included simple layout, lay language, short questions, few items on the screen, and capability to send/receive feedback and additional information
Morris, Prior, Deb, et al.	Traumatology	2005	UK	Psychologist (incl. clinical, neuro-)	Section of Clinical & Health Psychology	All HIC	BMC Family Practice	***Patients' views on outcome following head injury: A qualitative study***	To identify aspects of outcome considered important by survivors of traumatic head injury.	Mixed (16+)	TBI	UK	Qualitative	Not reported	Patients	32	Interviews (open, unstructured, in-depth, semi-structured, open ended)	Other: Analyzed qualitatively	Not reported	Not reported	Aspects of outcome mentioned by head injury survivors which have received less attention previously included: specific difficulties with group conversations; changes in physical appearance due to scarring or weight change; a sense of loss for the life and sense of self that they had before the injury; and negative reactions of others, often due to lack of understanding of the consequences of injury amongst both family and general public
Riley and Hagger	Traumatology	2015	UK	Not reported	School of Psychology	All HIC	Brain Injury	***Disclosure of a stigmatized identity: A qualitative study of the reasons why people choose to tell or not tell others about their traumatic brain injury***	To investigate what goals influence the decisions of people with a traumatic brain injury to disclose (or not to disclose) information about their brain injury.	Adult	TBI	UK	Phenomenology	Phenomenological	Patients	10	Interviews (open, unstructured, in-depth, semi-structured, open ended)	Thematic analysis	Braun & Clarke (2006)	Not reported	Study focused on disclosure to people other than immediate family and close friends. Reasons for not disclosing included concern about negative reaction from others, feelings of shame about the injury, wanting to avoid getting distressed, wanting to fit in, lack of interest from others, and perception that the stress associated with the act of disclosing outweighed the benefits. Reasons for disclosing included obtaining emotional and practical support from others, need to explain their behavior to others, and giving others the benefit of their experience. Experience of negative and stigmatizing reactions from others was common.
Riley and Balloo	Traumatology	2016	UK	Not reported	School of Psychology	All HIC	Cogent Psychology	***Maternal narratives about their child's identity following acquired brain injury***	To explore differences in how mothers perceive the identity of their child after acquired brain injury and the emotions associated with these different perceptions	Pediatric	Mixed ABI	UK	Narrative inquiry	Content and context not structure or performance	Family members, carers, signifcant others	5	Interviews (open, unstructured, in-depth, semi-structured, open ended)	Thematic analysis (content related TA)	Braun & Clarke (2006)	Not reported	Three general narratives are described: a child with problems, in which the problems of the child dominated the perception of the child's identity and the post-injury child was viewed as fundamentally different from the pre-injury child; an improving child, in which the child's progress and achievements figured prominently, and the post-injury child was viewed as having an identity continuous with that of the pre-injury child; and an improved child, in which the post-injury child was viewed as fundamentally different and improved compared to the pre-injury child. These narratives were associated with different emotional responses: A child with problems was associated with a sense of burden, grief and anxiety about the future. These emotions were relatively absent from the other two narratives, and an improving child was associated with a sense of relief, pride and optimism.
Swift and Wilson	Traumatology	2001	UK	Not reported	Center for Ethics in Medicine	All HIC	Brain Injury	***Misconceptions about brain injury among the general public and non-expert health professionals: An exploratory study***	To investigate the lack of knowledge and misconceptions concerning brain injury, as perceived by those with experience of the condition.	Adult	Mixed ABI	UK	Qualitative	Not reported	Patients AND Family members, carers, signifcant other AND HCPs	22	Interviews (open, unstructured, in-depth, semi-structured, open ended)	IPA	Smith (1995)	Not reported	According to participants, inaccurate and inadequate knowledge about brain injury is common among the general public and among health professionals without expertise in the field of brain injury. The major themes that emerged from the analysis were: inaccurate beliefs about recovery time and possible extent of recovery from brain injury; lack of awareness of the diversity of problems it can cause, particularly the existence of behavioral and cognitive sequelae; misconceptions about the capabilities of brain-injured people depending on the visibility or invisibility of their disability: and misidentification of brain-injured individuals as mentally ill or learning disabled.
Townshend and Norman	Traumatology	2018	UK	Not reported	School of Psychology,	All HIC	Family Journal	***The Secondary Impact of Traumatic Brain Injury: An Interpretative Phenomenological Analysis of the Experiences of Family and Friends***	To understand the lived experiences of family members and friends of individuals with traumatic brain injury	Adult	TBI	UK	Phenomenology	Not reported	Family members, carers, signifcant others	11	Interviews (open, unstructured, in-depth, semi-structured, open ended)	IPA	Smith & Osborn (2008)	Not reported	Several strong and interconnected themes emerged from the interviews, clustering into four superordinate themes: "continuity and discontinuity"; "damage, loss, and grief"; "roles and responsibility"; and "coping and not coping." The study findings identify the importance of support and information provision for family members and friends of those with TBI to help them manage the long-term impact of TBI.
Wales, Sidebotham and Hawley	Traumatology	2019	UK	Not reported	The Children's Trust ,	All HIC	Brain Injury	***Self-awareness following a traumatic brain injury in childhood: a developmental perspective***	To understand the self-awareness of deficits from a developmental perspective	Pediatric	TBI	UK	Mixed methods (Multiple case study + mixed methods)	Not reported	Patients AND Family members, carers, signifcant others AND Non-clinical providers	15	Mixed: Interviews (open, unstructured, in-depth, semi-structured, open ended) AND questionnaires	Thematic analysis (inductive AND deductive)	Braun & Clarke (2006)	Not reported	Within-case and cross-case analysis identified interrupted development of self-awareness following TBI. CYP ratings differed most from parent/teacher ratings in the social and behavioral domains. In relation to the school/learning and physical domains, CYP ratings differed most from normative children's data. The younger children had greater degree of ratings discrepancy across all domains. Seven key themes were aspirations, beliefs, being different, brain injuries, characteristics, participation, and interpersonal interactions and relationships.

Whiffin, Bailey, Ellis-Hill, et al.	Traumatology	2015	UK	RN/Nurse	University of Derby	All HIC	Journal of Advanced Nursing	***Narratives of family transition during the first year post-head injury: Perspectives of the non-injured members***	To explore the narratives created by non-injured family members in relation to themselves and their family in the first year after head injury.	Adult	TBI	UK	Case study	Constructivist and Narrative Theory	Family members, carers, signifcant others	9	Interviews (open, unstructured, in-depth, semi-structured, open ended)	Narrative analysis	Riessman & Quinney (2005); Gergen & Gergen (1983)	Not reported	Five interwoven narratives were identified: trauma, recovery, autobiographical, suffering and family. The narrative approach emphasized that the year post-head injury was a turbulent time for families, who were active agents in the process of change.
Whiffin, Ellis-Hill, Bailey, et al.	Traumatology	2019	UK	Not reported	College of Health and Social Care, University of Derby, Derby, UK	All HIC	Neuropsycholo gical Rehabilitation	***We are not the same people we used to be: An exploration of family biographical narratives and identity change following traumatic brain injury***	To describe the narrative structures used by uninjured members of a family to understand change.	Adult	TBI	UK	Qualitative	Social constructivism and narrative theory	Family members, carers, signifcant others	9	Interviews (open, unstructured, in-depth, semi-structured, open ended)	Narrative analysis	Riessman & Quinney, (2005); Creswell, 2013)	Not reported	Data that referred directly to the recovery of the injured relative revealed how the uninjured family members understood identity and change. The findings showed that family members had a lot of narrative work to do post-injury; which means they needed to understand their own sense of self post-injury as well as making sense of the identity and character of their injured relative. It is this narrative understanding of change that is the focus of this paper. Examples of the four narrative structures are presented below starting with biographical attendance, then moving on to biographical disruption, biographical continuity, and finally biographical reconstruction, which is used to examine how family members' own identities are challenged alongside that of the injured person. During this discussion, the consequences of biographical reconstruction when not all family members share the same experience is discussed in terms of "narrative misalignment". Narrative misalignment is defined as a lack of symmetry between people, events, and experiences and the impact this has on their interpretation. Extracts of data are presented below from family members that illustrate these narrative structures and the context in which family members were speaking.
Grayson, Brady, Togher, et al.	Traumatology	2020	UK	Not reported	NMAHP Research Unit; UK Community Brain Injury Team	All HIC	International Journal of Language and Communication Disorders	***A survey of cognitive-communication difficulties following TBI: are families receiving the training and support they need?***	To identify the family needs for cognitive-communication difficulties following TBI and to explore whether current services are meeting these needs.	Adult	TBI	UK and Australia	Mixed methods (survey)	Not reported	Family members, carers, signifcant others	102	Open ended question (one)	Content analysis	Creswell & Creswell (2018)	Not reported	Respondents rated information about expected recovery from cognitive-communication difficulties and training in helpful strategies as their most important needs. The majority of respondents (more than 60%) were not satisfied that any of their cognitive-communication needs had been fully met and high levels of unmet need remained evident at three years or more post-injury. Written information, communication partner training and counselling were identified as key supports.
Brunner, Palmer, Togher, et al.	Traumatology	2019	Australia	Speech pathologist	Speech Pathology, Graduate School of Health	All HIC	International Journal of Language and Communication Disorders	***I kind of figured it out': the views and experiences of people with traumatic brain injury (TBI) in using social media-self-determination for participation and inclusion online***	To determine the views and experiences of adults with TBI and cognitive-communication disability on using social media, specifically: (1) the nature of their social media experience; (2) barriers and facilitators to successful use; and (3) strategies that enabled their use of social media.	Mixed	TBI	Unclear	Qualitative	Not reported	Patients	13	Interviews (conversational style)	Grounded theory analysis/constant comparison/open and axial/selective coding (iterative thematic analysis)	Braun & Clarke (2006); Tracey (2013)	Not reported	Participants used several social media platforms including Facebook, Twitter, Instagram and virtual gaming worlds. All but one participant used social media several times each day and all used social media for social connection. Five major themes emerged from the data: (1) getting started in social media for participation and inclusion; (2) drivers to continued use of social media; (3) manner of using social media; (4) navigating social media; and (5) an evolving sense of social media mastery. In using platforms in a variety of ways, some participants developed an evolving sense of social media mastery. Participants applied caution in using social media, tended to learn through a process of trial and error, and lacked structured supports from family, friends or health professionals. They also reported several challenges that influenced their ability to use social media, but found support from peers in using the social media platforms.
Morris, Daluiski and Dy	Traumatology	2016	USA	MD/Physician	Department of Orthopedic Surgery, Division of Hand and Upper Extremity Surgery	All HIC	Journal of Hand Surgery-American Volume	***A Thematic Analysis of Online Discussion Boards for Brachial Plexus Injury***	To explore if a thematic analysis of posts from BPI Internet discussion groups would reveal common themes related to the BPI patient experience, providing topics for patient education and counseling.	Unclear	Nerve; Brachial plexus injury	Unclear - Not reported	Qualitative	Not reported	Patients AND Family members, carers, signifcant others	50	Social Media content: Online discussion boards	Grounded theory analysis/constant comparison/open, axial and/or selective coding (Thematic)	Gooden & Winefield (2007); Sullivan (2003)	Not reported	A total of 328 posts from the 2 leading discussion boards were analyzed. Investigators reached a consensus on themes for all posts. One central theme focused on emotional aspects of BPI. Four other central themes regarding information support were identified: BPI disease, BPI treatment, recovery after BPI treatment, and process of seeking care for BPI.
Carlozzi, Kratz, Sander, et al.	Traumatology	2015	USA	Psychologist (incl. clinical, neuro-)	Physical medicine and rehab	All HIC	Archives of Physical Medicine and Rehabilitation	***Health-related quality of life in caregivers of individuals with traumatic brain injury: Development of a conceptual model***	To identify aspects of health-related quality of life (HRQOL) that are relevant to caregivers of individuals with traumatic brain injury (TBI) and to propose an integrated conceptual framework based on this information	Adult	TBI	US	Qualitative	Not reported	Family members, carers, signifcant others	55	Focus groups	Grounded theory analysis/constant comparison/open, axial and/or selective coding (Inductive, deductive)	Glasser & Strauss (1967), Strauss & Corbin, (1998)	Not reported	Qualitative analysis indicated that caregivers were most concerned about their social health (42% of comments). Other important issues were emotional health (34%), physical health (11%), cognitive health (3%), and feelings of loss (9%; feelings of loss related to changes in the future/potential of the care recipient or related to the caregiver). Areas of concern that were discussed that were specific to the caregiver and not fully evaluated by existing patient-reported outcomes (PROs) included feelings of loss, anxiety related to the caregiver role (reinjury concerns, worry about leaving the person alone, etc.), and caregiver strain (burden, stress, feeling overwhelmed, etc.).
Carlozzi, Lange, French, et al.	Traumatology	2018	USA	Not reported	Physical medicine and rehab	All HIC	Journal of Head Trauma Rehabilitation	***A Latent Content Analysis of Barriers and Supports to Healthcare: Perspectives from Caregivers of Service Members and Veterans with Military-Related Traumatic Brain Injury***	To identify barriers and supports that caregivers of individuals with military-related traumatic brain injury (TBI) encounter when navigating the military healthcare system; this information will be used as the foundation of a new patient-reported outcome measure.	Adult	TBI (veterans)	US	Qualitative	Not reported	Family members, carers, signifcant others	45	Focus groups	Content analysis (latent)	Catanzaro (1998), Hsieh & Shannon (2005)	Not reported	Latent content analysis indicated that caregivers discussed barriers (66%) and supports (34%) to obtaining care within the military healthcare system and the community. Caregivers most frequently discussed SMVs' interactions with healthcare, their own interactions with healthcare, family care, and community organizations.
Carson	Traumatology	1993	USA	RN/Nurse	College of Nursing	N/A	The Journal of neuroscience nursing : journal of the American Association of Neuroscience Nurses	***Investing in the comeback: parent's experience following traumatic brain injury***	To identify a qualitatively-generated theory describing the parent's experience following a brain-injured child's return to the home setting.	Mixed	TBI	US	Mixed methods (Surveys; Grounded theory)	Not reported	Family members, carers, signifcant others	20	Interviews (open, unstructured, in-depth, semi-structured, open ended)	Grounded theory analysis/constant comparison/open and axial/selective coding	Not reported	Not reported	All the brain-injured offspring had survived moderate to severe traumatic brain injury, were living with at least one parent, and were aged 17-34 years. Investing in the Comeback is the generated three-phase theory. These three phases--centering on, fostering independence and seeking stability describe the work of parents living with brain-injured offspring.

Malhi, S. K. and Welch-West, P. and Koo, A. M. and Fogarty, J. and Lazosky, A.	Traumatology	2023	Canada	Neuropsychologist	London Health Sciences Centre	All HIC	Neuropsychological Rehabilitation	***Who gets to decide?: Substitute decision making following severe brain injury with communication impairment***	to better understand the decision-making process of their substitute decision makers	Adult	Severe brain injury	USA	Qualitative	Not explicitly stated	Family/Carer/Significant other	4	Interviews (semi-structured, open, in depth)	Thematic analysis	Not stated	Not reported	SDMs were performing various roles ranging from making all decisions to simply being the vocal advocate (for the augmentative and alternative communication users). SDMs described heuristics they used to make decisions, and all identified auditory comprehension capabilities as being an important element for decision making.
Krefting	Traumatology	1989	Canada	Not reported	Department of Occupational Therapy	N/A	Occupational Therapy Journal of Research	***Reintegration into the community after head injury: The results of an ethnographic study***	To re-examine long-term recovery from head injury using ethnography	Mixed (16+)	TBI	USA	Ethnography	Not reported	Patients AND Family members, carers, signifcant others AND non-clinical providers	21 (at least + families, neighbors, teachers,, friends)	Mixed: Semi-structured interviews AND Observation AND Document analysis	Thematic analysis AND content analysis	Not reported	Not reported	Three code words are: concealment, blind spots and redefinition. These are ways in which persons with head injury and their families cope with loss of self-identity and establish a new sense of self-identity and personhood. In response to the loss of self-identity, they build a facade, developing an image they would like to have. Facades provide a sense of identity that meets the expectation of one's self and of others. Recasting some or all of the disability's negative features in more of a positive light makes a seemingly intolerable situation bearable and a productive future possible. These strategies also provide persons with head injuries with a sense of control over the disability.
Sullivan, Wysong and Yang	Traumatology	2022	Ireland / USA	Not reported	Discipline of Children's Studies, School of Education, College of Arts, Social Sciences, & Celtic Studies,	All HIC	The Journal of school health	***Concussion Recovery in Children and Adolescents: A Qualitative Study of Parents' Experiences***	To explore parents' experiences with and perceptions of their child's recovery from concussion, particularly with regards to the return-to-school process.	Pediatric	TBI (concussion)	USA	Qualitative	Not reported	Family members, carers, signifcant others	11	Interviews (open, unstructured, in-depth, semi-structured, open ended)	Grounded theory analysis/constant comparison/open, axial and/or selective coding	Holton (2007); Saldaña (2015); Moghaddam (2006)	Not reported	We identified seven themes during data analysis, which we divided into two categories: at home (prior to school re-entry) and at school (during the school reintegration process). The at home category included four themes: (1) physical and cognitive rest; (2) child-led decision-making; (3) the role of parent-child communication in recovery; and (4) feelings about returning to school following concussion. The at school category included three themes: (1) availability and utilization of academic accommodations; (2) return-to-school policies and procedures; and (3) support from school officials and teachers.
Wongvatunyu and Porter	Traumatology	2008	Thailand	RN/Nurse	Ramathibodi School of Nursing,	All LMIC	Journal of Family Nursing	***Changes in family life perceived by mothers of young adult TBI survivors***	To describe perceived changes the seven mothers reported in family life 6 months or more after their young adult child had suffered a TBI.	Adult	TBI	USA	Phenomenology	Phenomenological	Family members, carers, signifcant others	7	Interviews (open, in-depth, semi-structured, open ended)	Phenomenological descriptive analysis	Porter (1995, 1998)	Not reported	Most of the mothers described their families as being close prior to the injury. Due to the TBI and its aftermath, the mothers perceived that the life320Journal of Family Nursing of the family had changed and that it would never be the same again. Across the sample, data pointed to five different changes perceived by the mothers. Most of the changes pertained to interpersonal relationships, but one perceived change pertained to financial matters. The mothers realized that the changes in the life of the family following the TBI would linger and prob-ably be permanent.
Wongvatunyu and Porter	Traumatology	2005	Thailand	RN/Nurse	Ramathibodi School of Nursing,	Mixed HIC/LMIC	Journal of Nursing Scholarship	***Mothers' experience of helping young adults with traumatic brain injury***	To describe mothers' experience of helping young adults with traumatic brain injury (TBI).	Adult	TBI	USA	Qualitative (descriptive)	Phenomenological	Family members, carers, signifcant others	7	Interviews (open, unstructured, in-depth, semi-structured, open ended)	Other: inter-subjective dialogue	Porter (1998)	Not reported	Five phenomena that were structures of the experience were discerned, discussed with participants to obtain their feedback, and compared to the relevant literature. The five phenomena of the mothers' experiences were: reconnecting my child's brain, considering my child's safety, making our lives as normal as possible, dealing with our biggest problem, and advocating for my child.
Wongvatunyu and Porter	Traumatology	2008	Thailand	RN/Nurse	Ramathibodi School of Nursing,	Mixed HIC/LMIC	Qualitative Health Research	***Helping young adult children with traumatic brain injury: The life-world of mothers***	To explore the life-world of 7 mothers whose young adult children had suffered a moderate or severe TBI at least 6 months earlier	Adult	TBI	USA	Phenomenology	Phenomenological	Family members, carers, signifcant others	7	Interviews (open, in-depth, semi-structured, open ended)	Phenomenological analysis	Porter (1995)	Not reported	Conducting three interviews with each mother, we discerned five features of life-world: having a child who survived a TBI as a young adult, perceiving that life has really changed, having sufficient support/feeling bereft of any help, believing that my child is still able, and believing that I can help my child. Compared to the literature, findings led to more definitive practice implications about postinjury uncertainty and maternal role change.
Cogan, A. M. and Bailie, J. M.	Traumatology	2023	USA	Other: Occupational Therapist	Division of Occupational Science and Occupational Therapy	All HIC	Military Medicine	***Therapeutic Relationship in mTBI Rehabilitation: The Disparity Between the Illness Experience and Clinical Definitions***	to (1) explore disparities between military service members and rehabilitation clinicians about the clinical diagnosis and illness experience of mTBI and (2) identify barriers to the establishment of a positive therapeutic relationship	Adult	mTBI	USA	Qualitative descriptive	Kleinman's paradigm of incongruence	Patients AND clinicians	24	Mixed/multiple: Interviews AND focus groups	Other: Kleinman's framing of clinical diagnosis and illness experience	Kleinman (1988)	Not reported	Three themes reflected the potential breakdowns in the therapeutic relationship. The first theme, clinical expectations for post-injury recovery versus patients' experience of ongoing disability, reflects the inconsistency between clinicians' expectations of symptom resolution within 90 days following mTBI and service members' experiences of symptoms that worsened over several months or years. The second theme, symptom attribution to mental health conditions versus tissue injury, describes the difficulty in attributing symptoms to the physical impact of the mTBI or mental health diagnoses that may also stem from the injury event. The third theme, suspected malingering versus valid disability, describes clinicians' reports of frustration with cases in which they suspected malingering for secondary gains in contrast with service members' feelings that their problems were not taken seriously by clinicians.
Fins, J. J. and Wright, M. S. and Shulman, K. S. and Henderson, J. M. and Schiff, N. D.	Traumatology	2023	USA	Unclear/not stated	Division of Medical Ethics; Solomon Center for Health Law & Policy	All HIC	Cambridge Quarterly of Healthcare Ethics	***Subject and Family Perspectives from the Central Thalamic Deep Brain Stimulation Trial for Traumatic Brain Injury: Part II***	we report on interviews conducted postoperatively after a successful trial of thalamic stimulation which met targeted milestones for improved executive dysfunction in chronic brain injury with at least a 10% improvement in completion time on Trail Making Test-Part B, which evaluates executive function.	Adult	TBI; DBS	USA	Qualitative	Non stated	Patients AND Families/carers/ Significant other	19	Interviews (semi-structured, open, in depth)	Grounded theory (Deductive and inductive)	Charmaz (2001); Strauss and Corbin (1990)	Not reported	Interviews following surgery and the stimulation trial revealed the challenge of adaptation to improvements in cognitive function and emotional regulation as well as altered (and restored) relationships and family dynamics. These improvements exposed barriers to social reintegration made relevant by recoveries once thought inconceivable. The study's success sparked concerns about post-trial access to implanted devices, financing of device maintenance, battery replacement, and on-going care. Most subjects and families identified the need for supportive counseling to adapt to the new trajectory of their lives

Gomez, Douglas and Glang, Ann and Haarbauer-Krupa, Juliet and Bull, Rachel and Tucker, Paula and Ratcliffe, Jonathan and Hall, Alex and Gioia, Gerard A. and Jain, Shabnam and Sathian, Usha and Simon, Harold K. and Wright, David	Traumatology	2023	USA	Unclear/not stated	Center on Brain Injury Research and Training, Department of Psychology	All HIC	NeuroRehabilitation	***Stakeholder perspectives on navigating the pediatric concussion experience: Exploring the needs for improved communication across the care continuum***	To 1) learn how information is conveyed between healthcare and school systems after a student sustains a concussion and 2) examine the barriers and facilitators to communication between healthcare professionals, school professionals and families.	Paediatric	TBI	USA	Qualitative	Critical realist ontology and epistemology	Mixed: parents, HCPs, educators, coaches	45	Focus groups	Thematic analysis	Braun and Clarke (2006)	Not reported	We identified four key themes within focus group data: (1) lack of effective communication between hospital and outpatient healthcare providers to school personnel; (2) parents who were strong advocates had improved communication with healthcare professionals and garnered more accommodations for their children; (3) non-school professionals and families were often confused about who the point of contact was at a given school; and (4) differing experiences for athletes vs. non-athletes.
Jones, T. M. and Bhanji, A. and Osman, S. and Cai, X. C. and Garfinkel, S. and Weinstein, A. A.	Traumatology	2022	USA	Unclear/not stated	Department of Health Administration and Policy	All HIC	Brain Injury	***Experiences of caregivers and individuals living with traumatic brain injury in accessing health information: a qualitative investigation***	To understand the experiences of individuals with TBI and caregivers in finding and using TBI-related health information and to understand potential interest in participating in TBI-related research.	Adult	TBI	USA	Qualitative	Constructivist	Patients AND Families/carers/ Significant other	24	Interviews (semi-structured, open, in depth)	Other: Dedictive coding technique	Kyngas, 2020	Not reported	Three major themes emerged from the analyses: 1) processes and resources for finding TBI-related health information, 2) reliability of information, and 3) participation in research. Study participants described using the internet, consulting with healthcare professionals, reading research articles, and seeking out information from other individuals with TBI or caregivers to search for information. Participants also shared their experiences related to evaluating the reliability of information and the impact of individuals with TBI and caregivers participating on research teams.
Jung, W. and Vogel, M. and Figuracion, K. C. F. and Byun, E. and Thompson, H.	Traumatology	2024	USA	Nurse/RN/RGN	RESILIENCE Center, School of Nursing	All HIC	Rehabilitation Nursing	***The Perceived Meaning of Traumatic Brain Injury for Older Adults: A Longitudinal-Multiple Case Study***	The aim of this study was to explore the perceived meaning of traumatic brain injury (TBI) over the first-year postinjury among older adults and to explore if and how meaning changes.	Adult	TBI	USA	Case study	Non stated	Patients	12	Interviews (semi-structured, open, in depth)	Thematic analysis	Braun and Clarke (2006)	SRQR	Four themes were identified: gratitude, vulnerability and dependence, slowing down and being more careful, and a chance for reflecting on life. Most participants' perceptions of their TBI remained either consistently positive or negative over the first-year postinjury.
Kersey, Jessica and Rice, Hannah and Connor, Lisa Tabor and Fields, Beth and Hammel, Joy	Traumatology	2024	USA	Other: Occupational Therapist	Program in Occupational Therapy	All HIC	The Journal of head trauma rehabilitation	***"They Just Love Me"- An Examination of Social Support Experiences and Values Among People With TBI***	The objective of this study was to describe the social support structures and experiences of adults with TBI.	Adult	TBI	USA	Mixed methods,	Non stated	Patients	16	Mixed/multiple: Survey AND Semi structured interviews	Reflexive thematic analysis	Braun and Clarke (2006; 2022)	Not reported	Interview themes fell under 2 grand themes: structure of social networks (social networks are made up of families; proximity influences the type of support) and quality of social support (commitment vs indifference; doing things with and for others adds meaning; and "they just love me")
Kreitzer, N. and Adeoye, O. and Wade, S. L. and Kurowki, B. G. and Thomas, S. and Gillespie, L. and Bakas, T.	Traumatology	2023	USA	Physician/MD	Department of Emergency Medicine	All HIC	Journal of Head Trauma Rehabilitation	***Iterative Development of the Caregiver Wellness After Traumatic Brain Injury Program (CG-Well)***	(1) To iteratively design a web/phone-based intervention to support caregivers of adults acutely following traumatic brain injury (TBI), Caregiver Wellness (CG-Well), and (2) to obtain qualitative and quantitative feedback on CG-Well from experts and caregivers to refine the intervention.	Adult	TBI	USA	Delphi/Consensus	Not explicitly stated	Families/carers/ Significant other AND clinicians	31	Mixed/multiple: Modified Delphi approach AND Interviews	Content analysis	Saldana (2016)	COREQ	Strengths and weaknesses within the themes of "acceptable," "appropriate," and "accurate" were documented (Table 2). Experts had more comments than caregivers acknowledging the website was intended for patients likely to survive their TBI, with little information acknowledging withdrawal of care decisions or end of life care. Compared to caregivers, experts had more comments to improve diversity and accessibility of the website. The most commonly coded positive statements were in the theme, "Acceptable," under the code, "Technology," meaning participants found the website to be user-friendly.
Kreitzer, N. and Murtaugh, B. and Creutzfeldt, C. and Fins, J. J. and Manley, G. and Sarwal, A. and Dangayach, N.	Traumatology	2023	USA	Other: trained in neurocritical care and neurological rehabilitation; qualitative researchers	Department of Emergency Medicine	All HIC	Frontiers in Human Neuroscience	***Prognostic humility and ethical dilemmas after severe brain injury: Summary, recommendations, and qualitative analysis of Curing Coma Campaign virtual event proceedings***	The purpose of this manuscript is to describe the themes that emerged from a a webinar that discussed topics focused on prognostic uncertainty, communicating prognosis to family members/caregivers, gaps within healthcare systems, and research infrastructure as it relates to patients experiencing SABI.	Adult	TBI	USA	Qualitative	Non stated	Mixed HCP (Inc. NSx)	not stated	Other: qualitative analysis of a webinar	Thematic analysis	Braun and Clark (2006)	Not reported	We coded 168 qualitative excerpts within the transcript. Two main themes were discussed: (1) the concept of prognostic uncertainty in the acute setting, and (2) lack of access to and evidence for quality rehabilitation and specialized continuum of care efforts specific to coma research. Within these two main themes, we found 5 sub-themes, which were broken down into 23 unique codes. The most frequently described code was the need for clinicians to acknowledge our own uncertainties when we discuss prognosis with families, which was mentioned 13 times during the webinar.
Lovette, Brenda C. and Kanaya, Millan R. and Grunberg, Victoria A. and McKinnon, Ellen and Vranceanu, Ana-Maria and Greenberg, Jonathan	Traumatology	2024	USA	Speech and language pathologist	MGH Institute of Health Professions	All HIC	Neuropsychological rehabilitation	***"Alone in the dark": A qualitative study of treatment experiences among young adults with a recent concussion and anxiety***	to understand patients' 1) experiences with treatments offered by health care providers; 2) experiences with attempted concussion management strategies; and 3) needs after their injury.	Adult	mTBI	USA	Qualitative	Not explicitly stated	Patients	17	Interviews (semi-structured, open, in depth)	Framework analysis	Fereday & Muir-Cochrane (2016), Gale et al. (2013)	Not reported	Findings provide insight into recommended treatments (e.g., active/avoidant strategies, accommodations, referrals), attempted strategies (e.g., lifestyle changes, pacing, relationships, acceptance-based coping skills), and patient needs (e.g., education, accommodations, referrals for cognitive and emotional skills). Participants frequently expressed that treatment recommendations were confusing and difficult to implement. They initiated non-prescribed strategies that helped promote recovery and expressed a desire for more interdisciplinary treatment and education on concussions.

Manglani, Heena R. and Lovette, Brenda C. and Grunberg, Victoria A. and Frieder, Jesse and Vranceanu, Ana-Maria and Greenberg, Jonathan	Traumatology	2024	USA	Psychologist	Center for Health Outcomes and Interdisciplinary Research (CHOIR), Department of Psychiatry	All HIC	Archives of physical medicine and rehabilitation	***"I Wish I Had That!": A Qualitative Analysis of Psychosocial Treatment Preferences Among Young Adults With Recent Concussion and Anxiety***	To assess psychosocial treatment preferences and factors that may affect treatment participation among young adults with a recent concussion and co-occurring anxiety.	Adult	mTBI: mild anxiety	USA	Qualitative	Not explicitly stated	Patients	17	Interviews (semi-structured, open, in depth)	Thematic analysis (hybrid deductive-inductive)	Braun and Clarke (2006)	Not reported	We identified 4 domains characterizing participants' perceptions of and preferences for treatment. (1) Program content: Participants preferred a program early after injury that included psychoeducation and coping skills (eg, activity pacing, deep breathing, mindfulness). (2) Therapeutic processes: Participants preferred a person-centered approach in which clinicians normalized anxiety postconcussion and reassured them of recovery. (3) Program logistics: Participants endorsed that a brief, virtual program would be acceptable. They preferred access to program components through multiple modalities (eg, audio, video) and accommodations to manage concussion symptoms. (4) Barriers and facilitators to participation: Barriers included acute concussion symptoms (eg, screen sensitivity), time constraints, and forgetting sessions. Facilitators included a program that is flexible (format, scheduling), personalized (self-chosen mode for reminders, measure of accountability), and accessible (ie, advertising through health care professionals or social media).
Martindale-Adams, J. L. and Zuber, J. and Burns, R. and Nichols, L. O.	Traumatology	2023	USA	Unclear/not stated	Department of Preventive Medicine	All HIC	Neurorehabilitation	***Caring again: Support for parent caregivers of wounded, ill, and/or injured adult children veterans***	This two-arm randomized clinical trial compared interventions to help parent caregivers improve their depression, anxiety, and burden and manage care by decreasing troubling and concerning behaviors.	Adult	TBI	USA	Mixed methods,	Not explicitly stated	Family/Carer/Significant other	82	Open ended survey questions (Caregiver self reports)	Thematic analysis	Glaser and Strauss (1967), Bernard (2006) and Maxwell (1996)	Not stated	There were 163 parent caregivers, mostly mothers. During six months, participants in both arms improved significantly in depression, anxiety, burden, and reported veteran troubling and concerning behaviors. REACH caregivers showed a group by time improvement in concerning behaviors. Benefits included resources, self-reflection, not feeling alone, new skills, improved self-efficacy, and helping others. Specific concerns include exclusion from military and veteran care briefings and concern for the future.
Mazzeo, M. and Hernan, G. and Veerubhotla, A.	Traumatology	2023	USA	Medical student	Department of Rehabilitation Medicine	All HIC	Frontiers in Neuroscience	***Usability and ease of use of long-term remote monitoring of physical activity for individuals with acquired brain injury in community: a qualitative analysis***	to understand patient-reported aspects of the feasibility of using physical activity monitors for long-term use in community-dwelling individuals with acquired brain injury.	Adult	MIXED ABI	USA	Qualitative	Not explicitly stated	Patients	10	Interviews (semi-structured, open, in depth)	Framework analysis	Bryman and Burgess (1994) and Goldsmith (2021)	COREQ	This pilot study found that patients with acquired brain injury faced challenges specific to their functional limitations and that the activity monitors worn on the waist or wrist may be better suited in this population.
McCart, M. and Todis, B. and Gomez, D. and Glang, A.	Traumatology	2023	USA	Unclear/not stated	Center on Brain Injury Research and Training, Department of Psychology	All HIC	Neurorehabilitation	***School experiences following traumatic brain injury: A longitudinal qualitative study***	To better understand the experiences of students and parents in the education system following TBI.	Paediatric	TBI	USA	Qualitative	Not explicitly stated	Family/Carer/Significant other AND Teachers	23	Mixed/multiple: Interviews (semi-structured, open, in depth) AND Observations	Reflexive thematic analysis	Braun and Clarke (2006), Clarke and Braun (2013)	Not reported	From these data, three themes were identified: lack of student tracking year to year, lack of educator training, and conflicting views between educators and parents about students' needs. These factors ultimately led to parent frustration and eventually conflict and deteriorating relationships between parents and educators.
Meixner, C. and O'Donoghue, C. R. and Erickson, A.	Traumatology	2024	USA	Unclear/not stated	Department of Graduate Psychology, Counselor Education	All HIC	Journal of Humanistic Counseling	***Navigating disenfranchised grief: Women's experiences caring for family with brain injury***	TO investigate the triumphs and obstacles associated with their lived experiences—including counseling and other supports	Adult	MIXED ABI	USA	Narrative inquiry and constructivist grounded theory	Constructivist	Family/Carer/Significant other	20	Interviews (semi-structured, open, in depth)	Grounded theory analysis	Bowers (1998), Charmaz (2014), Glaser and Strauss (1967), Lincoln and Guba (1985)	Not reported	Our analyses yielded four intersected categories reinforced by a guiding perspective of disenfranchised grief: (1) experiencing dissonance, (2) navigating ambiguous loss, (3) falling in and out of isolation, and (4) losing and reclaiming personal identity.
Molina-Vicenty, I. L. and Borras-FernÃ¡ndez, I. C. and Quintana, Y. and Robles-Gierbolini, E. and Canales-Emanuelli, C. and Srivastava, G. and PagÃ¡n-Ramos, M. and Vega-Debien, G. and Jovet-Toledo, G. and Pope, C. and Davis, B. and George-Felix, C. A. and Betances-Arroyo, G. S. and Nazario-MartÃ-nez, R.	Traumatology	2024	USA	Unclear/not stated	ACOS/Research & Development Service, VA Caribbean Health Care System; School of Medicine	All HIC	Military Medicine	***Enhancing Access Through Language-Tailored Approach in Telehealth and Veterans Video Connect: Traumatic Brain Injury (TBI) Veterans Satisfaction Assessment***	To explore telehealth/Veterans Video Connect satisfaction for Spanish/English TBI screening.	Adult	TBI	USA	Mixed methods,	Not explicitly stated	Mixed (please state in comments)	22	Mixed/multiple: Focus groups + semi-structured interviews	Content analysis	Marshall and Rossman (2011)	COREQ	On CVT (0-5 scale), overall satisfaction averaged 4.50 (English) and 4.69 (Spanish). Lowest scoring item for English users was easy video connection (4.25), while unclear expectations had the lowest Spanish score (3.60). For TSUQ, overall mean scores were 4.50 (English) and 4.67 (Spanish), with improved health post-telehealth having the lowest average (English 3.33, Spanish 3.67). Qualitatively, Veterans and providers noted strengths like access and communication but weaknesses around connectivity, care delays, and privacy. Differences emerged regarding convenience (Veterans) versus operational barriers (providers). There was a strong positive correlation for Spanish surveys and a moderate correlation for English surveys (r = 0.71 Spanish surveys, r = 0.69 English surveys) between TSUQ and CVT for individual respondents.
O'Donoghue, C. R. and Meixner, C.	Traumatology	2024	USA	Other: academic faculty in psychology and in communication sciences and disorders	Department of Communication Sciences and Disorders	All HIC	Qualitative Health Research	***Community Caring for a Family Member With Brain Injury: Women's Lived Experiences***	to explore the lived experiences of women caring for loved ones with brain injury	Adult	TBI	USA	Narrative inquiry AND grounded theory	Constructivist	Family/Carer/Significant other	20	Interviews (semi-structured, open, in depth)	Constant comparison	Charmaz (2006), Glaser and Strauss 1967)	Not reported	Findings revealed an overarching theme surrounding the intractable challenges and enduring triumphs of caregiving. This discovery further sub-divided into four themes. Two themes focused on traversing a fragmented system of care and managing the burden of caregiving. The remaining two entailed finding supports in family and friends and leveraging professional skillset(s) to optimize caregiving. The recommendations garnered from the women's experiences included enhanced caregiver education with follow-up post-medical care, expanding services into the community to support caregivers and their family members appropriately, and building compassionate networks of women living the caregiving experience. Consistent with a desire-based paradigm, leveraging relationships to triumph over the challenges provided this group of women a voice to promote effective care for self, loved ones, and others.

Oyesanya, T. O. and Ibemere, S. O. and Loflin, C. and McReynolds, V. and Anaya, B. and Huang, M. C. L. and Gonzalez-Guarda, R. and Strauman, T. J. and Bettger, J. P.	Traumatology	2023	USA	Unclear/not stated	Department of School of Nursing	All HIC	Brain Injury	***"If you respect me, you are respecting my culture": methods and recommendations for personalizing a TBI transitional care intervention***	to describe use of personalization to tailor a TBI transitional care intervention for various racial/ethnic groups.	Adult	tbi	USA	Qualitative descriptive	Individual and Family SelfManagement Theory (IFSMT)	Patients AND Families/carers/ Significant other AND HCPs	40	Focus groups	Content analysis	Hsieh and Shannon (2005)	SRQR	Three personalization-related themes emerged: 1) what is important to me, 2) finding someone to deliver the intervention who can adapt to my needs, and 3) respect over culture. Findings informed personalization strategies within our final manual.
Palusak, Cara and Dart, Libby and Ciccia, Angela and Nagele, Drew and Lundine, Jennifer P.	Traumatology	2024	USA	Unclear/not stated	Heritage College of Osteopathic Medicine	All HIC	Journal of pediatric rehabilitation medicine	***Caregiver and student perspectives on school services for students with traumatic brain injury during the COVID-19 pandemic***	to identify unique challenges created by COVID-19 school closures for students with traumatic brain injury (TBI) and their families with relation to special education accommodations, therapy services, social support systems, and mental health complications.	Paediatric	TBI	USA	Qualitative	Not explicitly stated	Patients AND Families/carers/ Significant other	41	Interviews (semi-structured, open, in depth)	Reflexive thematic analysis	Braun and Clarke (2006); Braun and Clarke (2019); Braun and Clarke (2022)	Not reported	Central themes encompassing the student- and caregiver-reported challenges and advantages of COVID-19 school closures were changes in [1] education delivery, special education services, and accommodations for children with TBI, and [2] social relationships for students with TBI and their caregivers.
Pinnow, D. and Causey-Upton, R. and Meulenbroek, P.	Traumatology	2022	USA	Speech and language pathologist	Department of Communication Sciences and Disorders	All HIC	Scientific Reports	***Navigating the impact of workplace distractions for persons with TBI: a qualitative descriptive study***	To explore the experiences of seven individuals with TBIs and how they perceived workplace distractions to impact their productivity	Adult	TBI	USA	Qualitative descriptive	phenomenology methods	Patients	7	Interviews (semi-structured, open, in depth)	Thematic analysis	Creswell & Poth (2018), Creswell (2009), Moustakas (1994)	Not reported	Main findings centered around what environmental distractions impacted work performance, the farther-reaching consequences of distractibility, strong emotional feelings and worry about perceived work performance associated with distractibility, mitigating distractibility through "gaming the attentional system", and utilizing music as a distraction masker to enhance task performance. In light of this study's findings, researchers, and clinicians are encouraged to consider the wider impact of distractions on persons with TBI. The real-life accounts documented in this study will assist researchers and clinicians to account for the impact of environmental distractions in rehabilitation and support employment for persons with TBI.
Roberson, C. J. and Hagues, R. and White, B. and Robbins, D.	Traumatology	2024	USA	Unclear/not stated	Social Work Department	All HIC	Family Relations	***Addressing caregiver needs of brain injury survivors through telehealth: A pilot qualitative study***	to identify effective means for delivering services to address the unique needs of caregivers for traumatic brain injury (TBI) survivors.	Adult	TBI	USA	Qualitative	Not explicitly stated	Family/Carer/Significant other	8	Focus groups	Inductive approach	Patton (2014)	Not reported	Qualitative analysis identified key themes: isolation, boundary issues, challenges of therapy, and the benefits of telehealth counseling.
Skladman, Rachel and Francoisse, Caitlin A. and L'Hotta, Allison J. and Novak, Christine B. and Curtin, Catherine M. and Ota, Doug and Stenson, Katherine C. and Tam, Katharine and Kennedy, Carie R. and James, Aimee and Fox, Ida K.	Traumatology	2024	USA	Medical doctor	Division of Plastic and Reconstructive Surgery	All HIC	Plastic and reconstructive surgery	***Upper Extremity Surgery in Cervical Spinal Cord Injury: A Prospective Comparative Mixed Methods Study***	to assess long-term changes in upper extremity function between surgical (TT or NT) and nonsurgical groups through a comprehensive mixed-methods approach.	Adult	Cervical spine injury	USA	Mixed methods,	Not explicitly stated	Patients AND Families/carers/ Significant other	169	Interviews (semi-structured, open, in depth)	Qualitative text analysis	Bernard (2002)	Not reported	Thirty-one participants had quantitative data across all time points: no surgery (n = 14), TT (n = 7), and NT (n = 10). SCIM scores improved in TT and NT groups compared with the no-surgery group ($P < 0.05$). The 36-Item Short Form Health Survey scores did not differ among groups. Qualitative data analysis (n = 168 interviews) corroborated SCIM findings: surgical participants and their caregivers reported improvement in transfers and ability to perform activities of daily living, including grooming and self-catheterization. Improved use of electronics and ability to operate a motor vehicle were also reported. Postoperative therapy was identified as a critical component of achieving gains.
Unruh, D. and Gomez, D. and Slocumb, J. and McCart, M. and Davies, S. and Haarbauer-Krupa, J. and Glang, A.	Traumatology	2023	USA	Unclear/not stated	Secondary Special Education & Transition, Special Education & Clinical Services Department	All HIC	Journal of School Health	***Stakeholder Perspectives on the School Experiences of Students With Traumatic Brain Injury: The Effects of COVID-19 Pandemic on Service Delivery***	to explore the school experiences of students with TBI. Thematic qualitative analyses were used.	Mixed	TBI	USA	Qualitative	Critical realism	Mixed: Patients, families, educators, healthcare professionals	70	Mixed: Interviews and focus groups	Thematic analysis	Braun and Clark (2006)	COREQ	Key themes from the analysis include (a) incidence of brain injuries decreased; (b) screen time for students with TBI exacerbated symptoms; (c) COVID protocols at school made it difficult for educators to identify and provide accommodations for students with TBI; (d) COVID protocols at school could inadvertently exacerbate mental health difficulties after a TBI; and (e) COVID-related logistics increased the time between an injury and return to school or return to play.
Woods, L. R. and Zachry, A. H.	Traumatology	2023	USA	Unclear/not stated	Department of Occupational Therapy	All HIC	International Journal of Therapy and Rehabilitation	***Factors that support and hinder recovery and adjustment after traumatic brain injury: a qualitative multiple case study***	to gain an understanding of the shared perspectives of parental caregivers and their adolescent children as to what factors supported and hindered recovery and adjustment after traumatic brain injury.	Adolescents	TBI	USA	Qualitative multiple case study	Not explicitly stated	Patients AND Families/carers/ Significant other	9	Interviews (semi-structured, open, in depth)	Thematic analysis; content analysis	Kohlbacher (2006), Bengtsson (2016)	Not reported	Seven themes were identified from the interviews: lack of educational resources about traumatic brain injury; poor consistency of care; caregiver stress and guilt; disconnection and isolation; connection, empathy and social support; being high achieving and resourceful; and authentic relationships between adolescents with traumatic brain injury and their parents.
Adams and Dahdah	Traumatology	2016	USA	Not reported	Hope After Brain Injury/ Center for Counseling & Enrichment	All HIC	NeuroRehabilitation	***Coping and adaptive strategies of traumatic brain injury survivors and primary caregivers***	To explore the needs and deficits of adult traumatic brain injury (TBI) survivors and primary caregivers; and to identify their self-initiated coping and adaptive strategies.	Adult	TBI	USA	Phenomenology	Not reported	Patients AND Family members, carers, signifcant others	17	Interviews (open, unstructured, in-depth, semi-structured, open ended)	Type unclear	Not reported	Not reported	Patience and understanding, support, and professional help were identified by TBI survivors and caregivers as being their most relevant needs. Self-reported deficits included short-term memory loss (STM), fatigue, anger, and personality changes, and the strategies that TBI survivors and caregivers identified tended to address their problems with these specific day-to-day deficits. Problem focused, emotion focused, and avoidant coping were utilized to some degree in their adjustment to home life and activities of daily living. Participants offered suggestions for mental health professionals addressing how to more effectively work with brain injury survivors and their primary caregivers.

Aitken, Mele and Barrett	Traumatology	2004	USA	MD/Physician	the Department of Pediatrics, University of Arkansas for Medical Sciences	All HIC	Archives of physical medicine and rehabilitation	***Recovery of injured children: parent perspectives on family needs***	To study the broader impact of emotional and financial burdens of injury in children on their families, along with solutions to these problems.	Pediatric	TBI	USA	Qualitative	Not reported	Family members, carers, signifcant others	16	Focus groups	Thematic analysis	Strauss and Corbin (1990); Morgan and Krueger (1998) Crabtree and Miller (1999)	Not reported	Overall satisfaction with care was high. Parents expressed concerns with communication, access to care, and the transition both within the phases of medical care and from medical care into the community. Consistent financial and social stressors were voiced, along with the need for peer support during all phases of care.
Bedell, Cohn and Dumas	Traumatology	2005	USA	Occupational therapist	Department of occupational therapy	All HIC	American Journal of Occupational Therapy	***Exploring parents' use of strategies to promote social participation of school-age children with acquired brain injuries***	To understand parents' perspectives about the strategies they use to promote social participation of their school-age children with acquired brain injuries in home, school, and community life.	Pediatric	Mixed ABI	USA	Qualitative (descriptive)	Family Centered philosophy	Family members, carers, signifcant others	19	Interviews (open, unstructured, in-depth, semi-structured, open ended)	Content analysis AND constant comparison	Bogdan & Biklen 2003, Strauss & Corbin 1990	Not reported	Parents needed time to allow the recovery process to unfold for themselves and their children and developed strategies that fit into or assisted with managing family routines. Over time parents developed insight into the activity demands and their child's potential success to participate in desired activities. Based on these insights, parents used "anticipatory planning," which involved previewing upcoming events and activities and using strategies to promote positive and prevent negative experiences for their children. Specific strategies that parents used to promote social participation were classified into three categories: Creating opportunities, teaching skills, and regulating cognitive and behavioral function.
Bennett, Marsh, Maertens, et al.	Traumatology	2019	USA	MD/Physician	Department of Pediatrics, Section of Pediatric Critical Care	All HIC	Pediatric Critical Care Medicine	***Decision-Making about Intracranial Pressure Monitor Placement in Children with Traumatic Brain Injury***	To identify the decisional needs of multidisciplinary clinician stakeholders.	Pediatric	intracranial pressure monitoring	USA	Qualitative	Not reported	HCPs (inc. NSx)	28	Mixed: Interviews (open, unstructured, in-depth, semi-structured, open ended) AND baseline survey AND photo shoot	Content analysis: open-coding using an emergent rather than an a priori method	Hsieh & Shannon (2005)	Not reported	Emergent themes included gaps in medical knowledge and information available for decision-making, differences in perspective between clinical specialties, and ethical implications of decision-making about intracranial pressure monitoring. Experienced clinicians described less difficulty with decision-making overall.
Bond, Draeger, Mandleco, et al.	Traumatology	2003	USA	RN/Nurse	Assistant professor, College of Nursing	All HIC	Critical care nurse	***Needs of family members of patients with severe traumatic brain injury. Implications for evidence-based practice***	To explore the needs of patients' families through individual interviews during the course of the patients' stay in the ICU.	Adult	TBI	USA	Qualitative (descriptive)	Not reported	Family members, carers, signifcant others	7	Interviews (type unclear)	Content analysis	Not reported	Not reported	Content analysis indicated four themes: Need to know; need for consistency; need for involvement in care; need to make sense of experience.
Bradley, Haun, Powell-Cope, et al.	Traumatology	2020	USA	Not reported	Research Service, James A. Haley Veterans' Hospital	All HIC	Brain Injury	***Qualitative assessment of the use of a smart phone application to manage post-concussion symptoms in Veterans with traumatic brain injury***	To assess (1): barriers and facilitators to use of the Concussion Coach app and (2) differences in use of the app among patient groups who experienced PCS progression versus improvement, or who were low-use users of the app.	Adult	TBI (veterans; concussion)	USA	Qualitative	Not reported	Patients	35	Interviews (open, unstructured, in-depth, semi-structured, open ended)	Content analysis	Hsieh & Shannon (2005)	Not reported	Primary facilitators were the app's ease of use, privacy, availability/ portability, and increased self-efficacy of the user. Primary barriers were a lack of sufficient app training, stigma, overly "basic" content, and symptom severity. While there were not prominent differences among user groups, users whose symptom severity improved described using more modules of the app than users who declined. Low-users attributed not using the app to content that was too basic or to their forgetting.
Brickell, Cotner, French, et al.	Traumatology	2020	USA	Not reported	Defense and Veterans Brain Injury Center and National Intrepid Center of Excellence	All HIC	Rehabilitation Psychology	***Severity of military traumatic brain injury influences caregiver health-related quality of life***	To examine the influence of traumatic brain injury (TBI) severity on the health-related quality of life of caregivers providing care to service members/ veterans (SMV) following a TBI.	Adult	TBI (veterans)	USA	Qualitative	Not reported	Family members, carers, signifcant others	30	Focus groups	Grounded theory analysis/constant comparison/open and axial/selective coding	Glasser & Strauss (1967)	Not reported	Eleven themes emerged: no time for self/needs last (83.3%), poor physical health (80.0%), increased stress/anxiety (76.7%), social isolation/loneliness (66.7%), lack of access to services (50.0%), impact on family life (46.7%), sleep issues (46.7%), finances/employment (36.7%), depression (30.0%), exhaustion (30.0%), and anger (16.7%). Exploratory pairwise comparisons revealed a higher proportion of the moderate/severe/penetrating TBI group endorsed 7 of the 11 themes (no time for self/needs last, increased stress/anxiety, impact on family life, sleep issues, finances/employment, exhaustion, anger, and i]increased stress/anxiety) compared with the other 2 groups.
Carlozzi, Brickell, French, et al.	Traumatology	2016	USA	Psychologist (incl. clinical, neuro-)	Department of Physical Medicine and Rehabilitation	All HIC	Journal of Rehabilitation Research and Development	***Caring for our wounded warriors: A qualitative examination of health-related quality of life in caregivers of individuals with military-related traumatic brain injury***	To develop a conceptual framework that captures aspects of health-related quality of life (HRQOL) for caregivers of individuals with military-related traumatic brain injury (TBI).	Adult	TBI (veterans)	USA	Qualitative	Not reported	Family members, carers, signifcant others	45	Focus groups	Grounded theory analysis/constant comparison/open and axial/selective coding (Descriptive)	Kisala & Tulsky (2010)	Not reported	Qualitative frequency analysis indicated that caregivers most frequently discussed social health (44% of comments), followed by emotional (40%) and physical health (12%). Areas of discussion that were specific to this population included anger regarding barriers to health services (for caregivers and service members), emotional suppression (putting on a brave face for others even when things are not going well), and hypervigilance (controlling one's behavior/environment to prevent upsetting the servicemember). Caring for wounded warriors with TBI is a complex experience that positively and negatively affects HRQOL. While some aspects of HRQOL can be evaluated with existing measures, evaluation tools for other important components do not exist.
Chhuom and Thompson	Traumatology	2021	USA	Social worker	School of social work	All HIC	The Journal of neuroscience nursing : journal of the American Association of Neuroscience Nurses	***Older Spousal Dyads and the Experience of Recovery in the Year After Traumatic Brain Injury***	To describe the impact of experiencing a mild/moderate TBI on the older adult as well as the significant other in the year post injury.	Adult	TBI	USA	Case study: Multiple design	Not reported	Patients AND Family members, carers, signifcant others	6	Interviews (open, unstructured, in-depth, semi-structured, open ended)	Content analysis AND cross-dyad comparison	Yin (2003)	Not reported	The identified themes were struggling to accept limitations and acceptance, gratitude, fear and concern, frustration/anger/guilt/resentment, broader social support, emotional coping, and the presence or absence of broader social support.
Chwalisz Stark-Wroblewski	Traumatology	1996	USA	Not reported	Department of Psychology,	All HIC	Applied Neuropsychology	***The subjective experiences of spouse caregivers of persons with brain injuries: A qualitative analysis***	The subjective experiences of spouse caregivers were qualitatively examined in essays provided spontaneously by 27 spouses of persons with brain injuries.	Adult	Mixed ABI	USA	Qualitative	Not reported	Family members, carers, signifcant others	27	Other: Essay	Content analysis: Inductive, typological	Miles & Huberman (1994)	Not reported	These essay data appear to reflect issues most salient to caregivers and ideas that were not adequately tapped by the traditional empirical study of caregiver burden during which these data were provided. Some themes present in the data provide support for findings of previous burden research, whereas others represent new areas for further investigation.
Cover, Roiger and Zwart	Traumatology	2018	USA	Not reported	Department of Health and Nutritional Sciences, South Dakota State University	All HIC	Journal of athletic training	***The Lived Experiences of Retired Collegiate Athletes With a History of 1 or More Concussions***	To examine the lived experiences of retired collegiate athletes with a history of 1 or more concussions to discern individual concussion histories, knowledge and perceptions of concussions, and postconcussion quality of life	Adult	TBI (concussion)	USA	Qualitative	Not reported	Patients (college athletes)	14	Interviews (open, unstructured, in-depth, semi-structured, open ended)	Other: consensual qualitative review	Hill et al (2005)	Not reported	Participants sustained their first concussion during adolescence and often experienced difficulties transitioning back into the post concussion academic environment. Judgments of injury severity were clearly evident in participants' knowledge and perceptions of concussions. Participants experienced an array of emotional, physical, cognitive, and social challenges during the immediate post concussion period but did not feel their concussion history decreased their current quality of life.

Davis, Maclagan, Ferguson, et al.	Traumatology	2017	USA	Professor of Teaching and Professor of Applied Linguistics	University of North Carolina	All HIC	Journal of Interactional Research in Communication Disorders	***What we can learn from mismatched and unexpected responses to questions in interviews with people who have traumatic brain injury***	To Analyze mismatched and unexpected responses to questions in a survey administered to 13 people with traumatic brain injury or their relatives.	Adult	TBI	USA	Linguistic methods	Not reported	Patients AND Family members, carers, signifcant others	25	Mixed: Interviews (open, unstructured, in-depth, semi-structured, open ended) AND questionnaires	Discourse analysis	Johnstone, (2007)	Not reported	Rather than giving simple one word or yes-no answers, the respondents provided unexpected narrative answers that went beyond the scope of the survey questions. The analysis identifies the types of narratives used by the respondents and highlights the ways in which they expressed their concerns about their life post-injury.
Degeneffe and Bursnall	Traumatology	2015	USA	Social worker (Associate Clinical Social Worker)	Rehabilitation Counseling Program, San Diego State University	All HIC	Social Work (United States)	***Quality of professional services following traumatic brain injury: Adult sibling perspectives***	To examine the quality and availability of professional supports provided to individuals with traumatic brain injury (TBI) and their families.	Unclear	TBI	USA	Survey	Not reported	Family members, carers, signifcant others	267	Open ended questions	Grounded theory analysis/constant comparison/open and axial/selective coding	Strauss & Corbin (1990)	Not reported	The siblings' comments suggested that the system-level response to TBI serving injured people and their families was inadequate, that many professionals lacked the skills and understanding to provide effective services, and that professionals did not provide sufficient information to the people with TBI or their families. However, most siblings endorsed a positive view of at least one of the professional services provided.
Degeneffe and Olney	Traumatology	2008	USA	Not reported	Rehabilitation Counseling Program	All HIC	Rehabilitation Counseling Bulletin	***Future concerns of adult siblings of persons with traumatic brain injury***	To examine future concerns conveyed by adult siblings who provided regular caregiving support to their brothers and sisters with traumatic brain injury.	Unclear	TBI	USA	Survey	Not reported	Family members, carers, signifcant others	280	Open ended questions	Grounded theory analysis/constant comparison/open and axial/selective coding	Strauss and Corbin, (1998)	Not reported	Common themes of such concerns were related to the caregiving siblings' roles as future caregivers and their perceptions of the quality of professional care provided to their injured family members. Also, participants endorsed concerns for their injured siblings' independence, recovery, and relationships with family and friends.
Degeneffe and Olney	Traumatology	2010	USA	Social worker (Associate Clinical Social Worker)	Department of Administration, Rehabilitation, and Postsecondary Education	All HIC	Brain Injury	***'We are the forgotten victims': Perspectives of adult siblings of persons with traumatic brain injury***	To determined how the lives of a cross-national sample of adult siblings had changed since their brothers and sisters incurred a traumatic brain injury.	Unclear	TBI	USA	Survey	Not reported	Family members, carers, signifcant others	272	Open ended questions	Grounded theory analysis/constant comparison/open and axial/selective coding	Denzin & Lincoln (1994)	Not reported	Three themes emerged: (1) 'Family impacts' such as growing up with a sibling with TBI and impact on family closeness; (2) 'Caring' including caring for and about the family member; and (3) 'Making sense of the experience' through adjustment and personal development.
Degeneffe, Gagne and Tucker	Traumatology	2013	USA	Social worker (Associate Clinical Social Worker)	Rehabilitation Counseling Program	All HIC	Journal of Applied Rehabilitation Counseling	***Family systems changes following traumatic brain injury: Adult sibling perspectives***	To gain an understanding of family outcome, adjustment, and needs following traumatic brain injury (TBI) from the perspectives of adult siblings.	Unclear	TBI	USA	Survey	Not reported	Family members, carers, signifcant others	277	Open ended questions	Grounded theory analysis/constant comparison/open and axial/selective coding	Bogdan and Biklen (1998); Hodder (1994); Glaser & Strauss (1967)	Not reported	Responses were grouped into four themes including a) the consequences of providing care, b) family system impacts, c) evolved attitudes and beliefs, and d) how families provide care. From the perspective of adult siblings, TBI presents outcomes which both strengthen and compromise family systems.
Diener, Kirby, Sumsion, et al.	Traumatology	2021	USA	Not reported	Department of Family & Consumer Studies	All HIC	Disability and Rehabilitation	***Community reintegration needs following Pediatric brain injury: perspectives of caregivers and service providers***	To explore the processes of community reintegration of children and families at least one year following a Pediatric brain injury from the perspective of caregivers and outpatient/community service providers.	Pediatric	TBI	USA	Qualitative	Bronfenbrenner's ecological systems theory	Family members, carers, signifcant others AND HCPs AND Non-clinical providers	22	Interviews (open, unstructured, in-depth, semi-structured, open ended)	Thematic analysis: deductive & inductive	Braun & Clarke (2014), Coffey & Atkinson (1996), Brantlinger et al (2005)	Not reported	Themes from both providers and caregivers indicated additional supports needed at all levels of Bronfenbrenner's ecological systems theory (i.e., individual, microsystem, mesosystem/ exosystem, macrosystem, chronosystem). Participants felt that several characteristics would be needed in an ideal service system following Pediatric brain injury including: community solutions (it takes a village), long-term approaches to care, and new financial approaches.
Eismann, Pearl, Theuerling, et al.	Traumatology	2019	USA	Not reported	Cincinnati Children's Hospital Medical Center	All HIC	Child Abuse and Neglect	***Feasibility study of the calm baby gently program: An educational baby book intervention on safe practices related to infant crying***	To assess the feasibility of the Calm Baby Gently educational baby book intervention for promoting safe practices related to infant crying in an effort to prevent AHT.	Pediatric	TBI; child abuse	USA	Mixed methods (Survey)	Not reported	Family members, carers, signifcant others	754	Open ended questions	Thematic analysis	Braun & Clarke (2006)	Not reported	Of the 819 caregivers (78%) who received the book, 92% (754) read it, and 51% (421) had another caregiver read it. Caregivers considered the book approachable, understandable, validating, and helpful for improving knowledge and skills related to infant crying. The book was rated more helpful by caregivers of younger age, male gender, and non-white race. Controlling for age, gender, and race, caregivers who read the book were more confident ($p = 0.033$) and had more knowledge on how to respond appropriately to infant crying ($p = 0.019$) than caregivers who had not read it.
Eliacin, Fortney, Rattray et al.	Traumatology	2018	USA	Not reported	Department of Veterans Affairs Medical Center	All HIC	Brain Injury	***Access to health services for moderate to severe TBI in Indiana: patient and caregiver perspectives***	We examined patient and caregiver experiences of health services for moderate to severe traumatic brain injury (TBI) in Indiana, to provide an understanding of their lived experiences and to inform policies and practices for health services delivery for TBI.	Adult	TBI	USA	Qualitative	Constructivist	Patients AND Family members, carers, signifcant others	62	Interviews (open, unstructured, in-depth, semi-structured, open ended)	Grounded theory analysis/constant comparison/open and axial/selective coding (constructivist)	Charmaz (2006)	Not reported	Three interrelated themes emerged from the data and characterized participants' experiences accessing health services for TBI: 1) inadequate access to health services; 2) unavailability of specialized, age-appropriate, and long-term health services; and 3) transportation barriers to health services that limit access to care and amplify rural health disparities for patients.
Eliacin, Fortney, Rattray, et al.	Traumatology	2021	USA	Not reported	Department of Veterans Affairs Medical Center	All HIC	Health & Social Care in the Community	***Patients' and caregivers' perspectives on healthcare navigation in Central Indiana, USA after brain injury***	To document the experiences of patients with traumatic brain injury (TBI) and their caregivers in navigating health systems for TBI care.	Adult	TBI	USA	Qualitative	Not reported	Patients AND Family members, carers, signifcant others	62	Interviews (open, unstructured, in-depth, semi-structured, open ended)	Grounded theory analysis/constant comparison/open, axial and/or selective coding	Charmaz (2006); Mils, Bonner, Francis (2006)	Not reported	Participants discussed three significant challenges about navigating health services for TBI care: lack of support for care navigation, financial barriers, and communication barriers. Participants described how navigating outpatient healthcare services for TBI remains complex and emphasized the need for ongoing care navigation support throughout the care continuum. They detailed the long-term financial burden of TBI including high treatment costs, limited insurance coverage, and the emotional toll that financial stress has on their ability to navigate healthcare services for ongoing TBI-related needs. They also discussed how ineffective patient-provider communication and lack of reliable, timely and comprehensive health information about TBI limited their engagement in and navigation of TBI health services.

Erlick, Vavilala, Jaffe, et al.	Traumatology	2021	USA	Medical student	School of Medicine and Dentistry	All HIC	Journal of Neurotrauma	***Provider Perspectives on Early Psychosocial Interventions after Pediatric Severe Traumatic Brain Injury: An Implementation Framework***	To understand healthcare provider perspectives and create a framework for incorporating provider perspectives of best practice early psychosocial interventions to improve experiences and outcomes after severe pediatric TBI.	Pediatric	TBI	USA	Qualitative	Consolidated Framework for Implementation Research	HCPs (inc. NSx)	23	Interviews (open, unstructured, in-depth, semi-structured, open ended)	Content analysis	Not reported	Not reported	Providers recommended a dual approach of institutional and individual factors contributing to an effective framework for addressing psychosocial needs, and recommended interventions in 3 domains: presenting coordinated clear messages to caregivers; reducing logistical and emotional burden of care transitions; assessing and addressing caregiver needs/concerns. Healthcare workers want to incorporate certain trauma-informed and family-centered practices at each stage of treatment to improve experiences of caregivers and outcomes for pediatric patients with severe TBI.
Fox, Hoben, Komaie, et al.	Traumatology	2021	USA	Not reported	Division of Plastic & Reconstructive Surgery, Department of Surgery, Washington University School of Medicine	All HIC	Disability and Rehabilitation	***Nerve transfer surgery in cervical spinal cord injury: a qualitative study exploring surgical and caregiver participant experiences***	To investigate perceptions of surgical participants and their caregivers regarding novel nerve transfer surgery to restore upper extremity function in cervical level spinal cord injury	Adult	Spine; SCI	USA	Qualitative	Not reported	Patients	7	Interviews (open, unstructured, in-depth, semi-structured, open ended)	Thematic analysis	Bernard (2011)	Not reported	Participants had limited information about procedures to improve function after spinal cord injury. When discussing their choice to undergo nerve (as compared to traditional tendon) transfer surgery, they describe a desire to avoid post-operative immobilization. Barriers included the pre-operative testing, cost and inconvenience of travel for surgery, and understanding complex health information related to the procedure. While expectations matched descriptions of outcomes among participants and were generally positive, caregivers expressed disappointment. The long time interval for gains in function to be realized and relatively incremental gains achieved were frustrating to all. People with cervical spinal cord injury and their caregivers need more information about options to restore function and about realistic range of improvements with treatment. Further work to mitigate barriers and develop health information materials around nerve transfer surgery may improve medical decision making around and appropriate use of this newer treatment option
Franzblau, Maynard, Chung, et al.	Traumatology	2015	USA	Not reported	Section of Plastic Surgery, Department of Surgery, University of Michigan	All HIC	Journal of Neurosurgery	***Medical treatment decision making after total avulsion brachial plexus injury: A qualitative study***	To describe how and why patients elect to pursue or forego surgical reconstruction, identify the barriers precluding adequate information transfer, and determine whether these patients are satisfied with their treatment choices over time	Adult	Nerve; Brachial plexus injury	USA	Grounded theory	Not reported	Patients	12	Interviews (open, unstructured, in-depth, semi-structured, open ended)	Grounded theory analysis/constant comparison/open, axial and/or selective coding	Beaton & Clark (2009); Mays & Pope (2000); Pope, Ziebland, Mays (2000)	Not reported	our decision factors emerged from our analysis: desire to restore function, perceived value of functional gains, weighing the risks and costs of surgery, and having concomitant injuries. Lack of insurance coverage (4 patients), delayed diagnosis (3 patients), and insufficient information regarding treatment (4 patients) prevented patients from making informed decisions and accessing care. Three individuals, all of whom had decided against reconstruction, had regrets about their treatment choices. Patients with pan plexus avulsion injuries are missing opportunities for reconstruction and often not considering the long-term outcomes of surgery. As more Americans gain health insurance coverage, it is very likely that the number of patients able to pursue reconstruction will increase. The authors recommend implementing clinical pathways to help patients meet critical points in care within the ideal timeframe and using a patient- and family-centered care approach combined with patient decision aids to foster shared decision making, increase access to information, and improve patient satisfaction with decisions. These measures could greatly benefit patients with BPI while reducing costs, improving efficiency, and generating better outcomes
Franzblau, Shauver and Chung	Traumatology	2014	USA	Not reported	Department of Surgery, Section of Plastic Surgery, University of Michigan Medical School	All HIC	Journal of Hand Surgery	***Patient satisfaction and self-reported outcomes after complete brachial plexus avulsion injury***	To examine patient-reported outcomes, including satisfaction, and to understand the patient perspective.	Adult	Nerve; Complete brachial plexus avulsion	USA	Mixed methods (Questionnaires; Qualitative)	Not reported	Patients	12	Interviews (open, unstructured, in-depth, semi-structured, open ended)	Grounded theory analysis/constant comparison/open, axial and/or selective coding	Corbin & Strauss (2008)	Not reported	Twelve patients participated in this study. Of the 7 participants who had reconstructive surgery, 4 felt their expectations had been met and 5 were satisfied with their outcomes. Reconstruction did not produce statistically significant improvements in upper extremity function, pain, or work ability. All patients reported dissatisfaction with upper extremity ability, and 9 expressed hope for innovative treatments (e.g., stem cell therapy, nerve reinsertion) that could potentially provide better outcomes than existing procedures and enable return to work.
Gill, Sander, Robins, et al.	Traumatology	2011	USA	Not reported	Department of Disability and Human Development	All HIC	Journal of Head Trauma Rehabilitation	***Exploring experiences of intimacy from the viewpoint of individuals with traumatic brain injury and their partners***	To explore qualitatively the experience of intimacy from the viewpoint of persons with traumatic brain injury (TBI) and their intimate partners.	Adult	TBI	USA	Qualitative	Not reported	Patients AND Family members, carers, signifcant others	18	Interviews (open, unstructured, in-depth, semi-structured, open ended)	Grounded theory analysis/constant comparison/open, axial and/or selective coding	Patton (2001) and Jeon (2004)	Not reported	Factors that were perceived as helping relationships remain strong included unconditional commitment, spending time together, open communication, a strong preinjury relationship, bonding through surviving the injury together, social support, family bonds, spirituality, experience with overcoming hardship, and coping skills. Factors that were perceived as barriers to intimacy included injury-related changes, emotional reactions to changes, sexual difficulties, role conflict and strain, family issues, social isolation, and communication issues.
Graves, Moore, Kehoe, et al.	Traumatology	2020	USA	Not reported	Washington State University College of Nursing Spokane and Harborview Injury Prevention and Research Center	All HIC	Journal of pediatric nursing	***Family Hardship Following Youth Concussion: Beyond the Medical Bills***	To examine the economic hardship facing families following concussion.	Adolescents	TBI (concussion)	USA	Qualitative	Not reported	Patients AND Family members, carers, signifcant others	34	Interviews (open, unstructured, in-depth, semi-structured, open ended)	Content analysis	Framework Method (Gale et al. 2013)	Not reported	In addition to direct health care expenses (e.g. copays and deductibles), families of youth with concussion faced indirect costs associated with tutoring and transportation to medical appointments, in some cases over long distances. Financial cost-sharing for concussion care varied widely across participants.
Greenberg, Otun, Kyaw, et al.	Traumatology	2022	USA	Not reported	Department of Neurological Surgery, Washington	All HIC	Applied Clinical Informatics	***Usability and Acceptability of Clinical Decision Support Based on the KIIDS-TBI Tool for Children with Mild Traumatic Brain Injuries and Intracranial Injuries***	To evaluate the acceptability and usability of an electronic CDS tool for managing children with mTBI and intracranial injuries.	Pediatric	TBI	USA	Mixed methods	Not reported	HCPs (inc. NSx)	20	Mixed: Interviews (open, unstructured, in-depth, semi-structured, open ended) AND questionnaire	Content analysis AND thematic analysis	Yen & Bakken (2012), Yen et al. (2018), Nowell et al. (2017)	Not reported	Among the 20 participants, most worked at teaching hospitals (80%), freestanding children's hospitals (95%), and level-1 trauma centers (75%). During the two prototype updates, problems with clarity of terminology and navigating through the CDS interface were identified and corrected. Corresponding to these changes, the number of usability problems decreased from 35 in phase 1 to 8 in phase 3 and the number of mistakes made decreased from 18 (phase 1) to 2 (phase 3). Through the survey, participants found the tool easy to use (90%), useful for determining a patient's level of care (95%), and likely to improve resource use (90%) and patient safety (79%). Interview themes related to the CDS's ability to support evidence-based decision-making and improve clinical workflow proposed implementation strategies and potential pitfalls.

Greenberg, Otun, Nasraddin, et al.	Traumatology	2021	USA	Neurosurgeon	Department of Neurological Surgery, Washington University School of Medicine	All HIC	BMC Medical Informatics and Decision Making	***Electronic clinical decision support for children with minor head trauma and intracranial injuries: a sociotechnical analysis***	To investigate the sociotechnical influences on implementing electronic CDS to guide clinicians' decisions regarding the need for ICU admission, and to investigate alternative uses of electronic CDS among children with minor head trauma and intracranial injuries.	Pediatric	TBI	USA	Qualitative	Sociotechnical model	HCPs (inc. NSx) AND non-clinical providers	32	Focus groups	Thematic analysis	Nowell (2017)	Not reported	Through inductive thematic analysis, we identified five primary themes: (1) clinical impact; (2) stakeholders and users; (3) tool content; (4) clinical practice integration; and (5) post-implementation evaluation measures. Participants generally supported using CDS to determine an appropriate level-of-care for these children. However, some had mixed feelings regarding how the tool could best be used by different specialties (e.g. use by neurosurgeons versus non-neurosurgeons). Feedback from the interviews helped refine the tool content and also highlighted potential technical and workflow barriers to address prior to implementation.
Hammond, Davis, Cook, et al.	Traumatology	2016	USA	MD/Physician	Indiana University School of Medicine and Rehabilitation	All HIC	Journal of Head Trauma Rehabilitation	***A conceptual model of irritability following traumatic brain injury: A qualitative, participatory research study***	To describe the experience (thoughts and feelings) of irritability from the perspectives of multiple people living with or affected by the problem, and to develop a conceptual model of irritability.	Adult	TBI	USA	Grounded theory	Constructivist	Patients AND Family members, carers, signifcant others AND HCPs AND researchers	44	Focus groups	Grounded theory analysis/constant comparison/open, axial and/or selective coding	Charmaz (2000), Glaser & Strauss (1967), Strauss & Corbin (1990)	Not reported	A conceptual model emerged in which irritability has 5 dimensions: affective (related to moods and feelings); behavioral (especially in areas of self-regulation, impulse control, and time management); cognitive-perceptual (self-talk and ways of seeing the world); relational issues (interpersonal and family dynamics); and environmental (including environmental stimuli, change, disruptions in routine, and cultural expectations).
Hammond, Davis, Cook, et al.	Traumatology	2012	USA	Not reported	Carolinas Rehabilitation, Carolinas Healthcare System and Department of Physical Medicine and Rehabilitation	All HIC	Brain Injury	***Relational dimension of irritability following traumatic brain injury: A qualitative analysis***	To examine the experience of irritability within the family system from the perspective of people with TBI and spouses of people with TBI.	Adult	TBI	USA	Qualitative	Constructivist	Patients AND Family members, carers, signifcant other AND HCPs AND non-clinical providers	44	Focus groups	Grounded theory analysis/constant comparison/open, axial and/or selective coding	Charmaz (2000), Glaser & Strauss (1967), Strauss & Corbin (1990)	Not reported	Several theories emerged regarding irritability with respect to spousal relations, all based on the overarching theory that irritability in people with TBI has a strong relational component involved in triggering, experiencing and preventing irritability. Sub-theories supporting this include: (1) irritability breeds further irritability, (2) spousal responses can trigger irritability among persons with TBI and vice versa, (3) difficulties making emotional connections may incite negative interactions, (4) expectations of others may contribute to irritable behavior, and (5) communication breakdowns may provoke irritability.
Hammond, Davis, Hirsch, et al.	Traumatology	2021	USA	MD/Physician	Department of Physical Medicine and Rehabilitation; School of Medicine and Rehabilitation	All HIC	Archives of Physical Medicine and Rehabilitation	***Qualitative Examination of Voting Empowerment and Participation Among People Living With Traumatic Brain Injury***	To examine political participation after traumatic brain injury (TBI).	Adult	TBI	USA	Qualitative	Not reported	Patients AND Family members, carers, signifcant others	85	Mixed: Interviews and observation	Grounded theory analysis/constant comparison/open, axial and/or selective coding	Glaser & Strauss (1967); Strauss & Corbin (1990)	Not reported	Four themes emerged from the data: (1) people with TBI have barriers to voting; (2) the voting process can be improved for people with TBI; (3) voting is the responsibility of members of society; and (4) voting is one way we have a voice in society.
Hammond, Davis, Whiteside, et al.	Traumatology	2011	USA	MD/Physician	Carolinas Rehabilitation, Carolinas Healthcare System	All HIC	Journal of Head Trauma Rehabilitation	***Marital adjustment and stability following traumatic brain injury: A pilot qualitative analysis of spouse perspectives***	To examine how a spouse who has experienced TBI affects the marital relationship.	Adult	TBI	USA	Qualitative	Constructivist	Family members, carers, signifcant others	10	Focus groups	Grounded theory analysis/constant comparison/open, axial and/or selective coding	Charmaz (2000), Glaser & Strauss (1967), Strauss & Corbin (1990), Gottman (1994), Buehlman & Gottman (1996)	Not reported	This study suggests that, after TBI, changes in family dynamics and the way spouses perceive those dynamics affect movement toward pulling together or pulling apart.
Harrison, Hunter, Thomas, et al.	Traumatology	2017	USA	Not reported	Rehabilitation Sciences, Kentucky Appalachian Rural Rehabilitation Network (KARRN)	All HIC	Disability and Rehabilitation	***Living with traumatic brain injury in a rural setting: supports and barriers across the continuum of care***	To increase understanding of the lived experience of people with TBI and caregivers in rural regions of Kentucky across the continuum of their care and 2) provide their perspectives on barriers and facilitators of optimal function and well-being.	Adult	TBI	USA	Qualitative (descriptive)	Not reported	Patients AND Family members, carers, signifcant others	19	Interviews (open, unstructured, in-depth, semi-structured, open ended)	Content analysis	Sandelowski (2000)	Not reported	TBI and emergency care - Barriers included lack of neurologists on call in rural settings, inadequate knowledge base among practitioners, distrust in local healthcare systems. Acute care - Both positive and negative views of interactions with healthcare providers. Lack of sensitivity and emotional support vs a lot of attention from staff all the time in ICU. Distance to hospital played a significant role. Inpatient rehabilitation - frustration, scare and difficulty of losing independence, however rehabilitation helped with gaining independence over ADLs. Difficulty with depending on those around them. Generally positive feedback about rehabilitation. More rural facilities seemingly had worse rehabilitation services subjectively. Transitioning home - loss of physical ability, difficulty with less structured routine and constant support, lack of knowledge of TBI in communities. Financial burdens. Social isolation.
Hartman, DePoy, Francis, et al.	Traumatology	2000	USA	Not reported	University of Maine	All HIC	Social Work in Health Care	***Adolescents with special health care needs in transition: Three life histories***	To examine the service and support needs of adolescents with special health care needs who are transitioning to adulthood.	Adolescents	TBI	USA	Case study	Not reported	Family members, carers, signifcant others	3	Interviews (open, unstructured, in-depth, semi-structured, open ended)	Thematic and taxonomic analysis	DePoy & Gitlin, (1998).	Not reported	Six major themes emerged in the analysis: (1) Begetting a service system, (2) Pathology or not pathology, (3) Educational stability vs. interruptions, (4) Role blurring of parents and providers, (5) Private life made public, and (6) Independence vs. burden.
Hunt and Harris	Traumatology	2017	USA	Assistant Professor and Athletic Training Graduate Program Coordinator	School of Health and Kinesiology	All HIC	Journal of allied health	***Psychological Impact of Cognitive Rest Following Sport-Related Concussion on Low Socioeconomic Status Adolescent Patients***	To describe the psychological impact of cognitive rest in adolescents recovering from concussion from sport- related concussion.	Adolescents	TBI (concussion)	USA	Qualitative	Not reported	Patients	8	Focus groups	Content analysis	Not reported	Not reported	Common themes included: 1) sport as social support, and 2) boredom-complicated recovery following cognitive rest.
Hux, Bush, Zickefoose, et al.	Traumatology	2010	USA	Not reported	Barkley Memorial Center	All HIC	Brain Injury	***Exploring the study skills and accommodations used by college student survivors of traumatic brain injury***	To explore how best to apply accommodations and learning strategies, how much survivors embrace them, the extent to which they facilitate college success or how they evolve over time for TBI survivors.	Adult	TBI	USA	Case study: Multiple design	Constructivist	Patients AND Family members, carers, signifcant others AND HCPs AND Non-clinical providers	17	Mixed: Artifacts AND Field notes; AND semi-structured interviews.	Grounded theory analysis/constant comparison/open and axial/selective coding	Corbin & Strauss (2008)	Not reported	Findings suggest challenges, learning strategies, future perspectives and the need for academic accommodations fluctuate and evolve for many years post-injury. Findings also suggest some survivors perceive their learning capabilities more positively than do others associated with them, leading to questions about whether over-accommodation occurs and the potential negative ramifications associated with this practice.

Hyatt, Davis and Barroso	Traumatology	2014	USA	RN/Nurse	Walter Reed National Military Medical Center	All HIC	Military medicine	***Chasing the care: soldiers experience following combat-related mild traumatic brain injury***	To describe the rehabilitation experiences of soldiers with a history of mTBI and their spouses.	Adult	TBI (veterans)	USA	Grounded theory	Not reported	Patients AND Family members, carers, signifcant others	18	Interviews (open, unstructured, in-depth, semi-structured, open ended): Individual & joint	Grounded theory analysis/constant comparison/open and axial/selective coding	Corbin & Strauss (2008), Charmaz (2006)	Not reported	The majority of the soldiers and spouses who participated (16/18) indicated that uncertain prognosis and symptom management were the greatest challenges of post- mTBI. Other challenges, such as delayed diagnosis, limited access to mental health care, and difficulty navigating an unfamiliar military health care system was also reported.
Hyatt, Davis and Barroso	Traumatology	2015	USA	RN/Nurse	Center for Nursing Science & Clinical Inquiry	All HIC	Journal of Nursing Scholarship	***Finding the New Normal: Accepting Changes After Combat-Related Mild Traumatic Brain Injury***	To explore family reintegration experiences, as described by married dyads, following a combat-related mTBI.	Adult	TBI (veterans)	USA	Grounded theory	Not reported	Patients AND Family members, carers, signifcant others	18	Interviews (open, unstructured, in-depth, semi-structured, open ended): Individual & joint	Not clearly stated	Charmaz, (2006); Pandit, (1996|); Strauss & Corbin, (1990, 1998).	Not reported	The overarching theme of the reintegration experience is described as finding the "new normal." A new normal was defined by participants as the couple's new, post-mTBI expectation of the family unit or family routine. Some participants indicated that they had accepted the post-mTBI changes and were working toward this new normal, whereas others indicated these changes were unacceptable and continued their efforts to return to pre-injury functioning.
Iadevaia, Roiger and Zwart	Traumatology	2015	USA	Athletic trainer (ATC)	Department of Health and Nutritional Sciences	All HIC	Journal of Athletic Training	***Qualitative examination of adolescent health-related quality of life at 1 year postconcussion***	To investigate the perceptions of adolescent student-athletes and their parents regarding the adolescents' HRQOL 1 year after sport-related concussion.	Adolescents	TBI (concussion)	USA	Qualitative	Not reported	Patients AND Family members, carers, signifcant others	14	Interviews (open, unstructured, in-depth, semi-structured, open ended)	Other: Qualitative consensual review	Robson (2002)	Not reported	Four major themes emerged from the interviews: (1) significant effect of symptoms, (2) feelings of frustration, (3)influence on school attendance and activities, and (4) nature of interpersonal and team relationships. Participants indicated that the physical symptoms of the concussion substantially affected their emotional and academic function. The influence of the concussion on social interactions seemed to depend on the nature of interpersonal relationships.
Jimenez, Fuentes, Frias-Garcia, et al.	Traumatology	2020	USA	MD/Physician	Harborview Injury Prevention and Research Center	All HIC	Hospital pediatrics	***Transitions to Outpatient Care After Traumatic Brain Injury for Hispanic Children***	To explore Hispanic parents' experiences during child's transitions of care after TBI.	Pediatric	TBI	USA	Qualitative	Gelberg and Andersen's behavioral model	Family members, carers, signifcant others	15	Interviews (open, unstructured, in-depth, semi-structured, open ended)	Thematic: Content analysis	Not reported	Not reported	Fifteen mothers, mostly from rural areas and with limited English proficiency, participated in the study. Obtaining outpatient rehabilitation was difficult. Barriers included lack of therapists and clinical providers close to home, worsened by insufficient transportation and other support resources; poor understanding of child's illness and treatments; and suboptimal communication with clinicians and school administrators. Facilitators included interpreter use, availability of Spanish written information, and receipt of inpatient rehabilitation. Parents of patients discharged to inpatient rehabilitation reported that observing therapies, receiving school discharge plans by hospital-teachers, and coordination of care were facilitators to access outpatient treatments and to support school return. Parents of children discharged from the hospital from acute care reported need of legal services to obtain school services.
Johnson	Traumatology	1995	USA	RN/Nurse	School of nursing	N/A	The Journal of neuroscience nursing : journal of the American Association of Neuroscience Nurses	***One family's experience with head injury: a phenomenological study***	Describe the lived experience of one family with traumatic brain injury	Unclear	TBI	USA	Phenomenology	Rogers (1970) - science of unitary human beings	Patients AND Family members, carers, signifcant others	7	Interviews (open, in-depth, semi-structured, open ended)	Other: Searching for phrases that captured the essence of expereince	Van Manen (1990)	Not reported	Three themes emerged: "Helplessness and the need to hope", "Need to be informed and involved" and "impact of intubation/extubation." The victims unique perspective was captured by themes: "going home", "concern for others" and "piecing it together"
Jones, Quinn, Mazor, et al.	Traumatology	2021	USA	Not reported	Department of Neurology,	All HIC	Neurocritical Care	***Prognostic Uncertainty in Critically Ill Patients with Traumatic Brain Injury: A Multicenter Qualitative Study***	To examine themes of uncertainty in physician communication of prognosis and surrogate goals-of-care decision-making for critically ill patients with traumatic brain injury (TBI).	Adult	TBI	USA	Qualitative (secondary analysis)	Not reported	Family members, carers, signifcant others AND HCPs (expert physicians)	36	Interviews (open, unstructured, in-depth, semi-structured, open ended)	Inductive (on the major theme of uncertainty)	Not reported	Not reported	Nearly all surrogates (15 of 16) and physicians (19 of 20) recognized the emotional burden of uncertainty in the decision-making process for surrogates. More than three quarters of surrogates (13 of 16) described instances in which a lack of information regarding their loved one's disease or prognosis created uncertainty in their decision-making process, identifying both positive and negative instances of prognostic communication by physicians. We found that physicians used one of three strategies to communicate prognostic uncertainty to surrogates: leaving no room for uncertainty, honesty about uncertainty, and range of possibilities. These strategies did not meet the communication preferences of the majority of surrogates, with more than a third of decision-makers (6 of 15) being frustrated by too much ambiguity about prognosis as well as the failure to acknowledge the existence of uncertainty.
Kao and Stuifbergen	Traumatology	2004	USA	RN/Nurse	School of Nursing, College of Health and Human Services	All HIC	The Journal of neuroscience nursing	***Love and load--the lived experience of the mother-child relationship among young adult traumatic brain-injured survivors***	To describe the meaning of the experience of the relationship between young adult traumatic brain injury (TBI) survivors and their mothers using a phenomenological approach.	Mixed	TBI	USA	Phenomenology	Not reported	Patients AND Family members, carers, signifcant others	24	Interviews (open, unstructured, in-depth, semi-structured, open ended)	Phenomenological analysis	Colaizzi (1978)	Not reported	Survivors acquired the sense of being abnormal from various sources, including social pressures, dynamics within the family, and intrapersonal changes. Mothers adopted both positive and negative actions during the period of uncertainty and often struggled to balance protecting their children and letting them become independent. They also struggled to maintain harmonious relationships with people both inside and outside of the family. Sometimes, survivors' parents marital relationships were at risk.
Keck, Creaghead, Turkstra, et al.	Traumatology	2017	USA	Not reported	University of Cincinnati, Department of Communication Sciences and Disorders	All HIC	Journal of Communication Disorders	***Pragmatic skills after childhood traumatic brain injury: Parents' perspectives***	To characterize pragmatic deficits after childhood traumatic brain injury (TBI) within the home environment social contexts where they occur.	Pediatric	TBI	USA	Qualitative (descriptive)	Not reported	Family members, carers, signifcant others	10	Mixed: Interviews (open, in-depth, semi-structured, open ended) AND questionnaires	Other: analyzed using a deductive framework	Pope, Ziebland, & Mays (2000)	Not reported	Overall, mothers primarily described their children with TBI as exhibiting average or near average pragmatic skills at home, but nine observed some pragmatic deficits and/or social behavior problems. There were four in-home social contexts in which pragmatic deficits were observed. Emergent themes also included outside-of-the home social contexts and social behavior problems. There was some overlap of pragmatic deficit and social behavior problem themes among contexts, but many deficits were context specific. This study's pragmatic deficit themes expanded on prior childhood TBI pragmatic investigations by identifying contexts in and outside of the home in which pragmatic deficits may occur after TBI.
Keegan, Müller, Ball,et al.	Traumatology	2022	USA	Not reported	Speech-Language Pathology, School of Rehabilitation Sciences	All HIC	Neuropsychological rehabilitation	***Anger and aspirations: Linguistic analysis of identity after traumatic brain injury***	To investigate how language was used to create and establish identities for three participants with a severe TBI.	Adult	TBI	USA	Case study	Not reported	Patients	3	Interviews (open, in-depth, semi-structured, open ended)	Other: Topic analysis	Mentis and Prutting (1991)	Not reported	Topic analysis revealed that topics of anger and of aspirations were commonly discussed by participants and their caregivers, and a detailed analysis of identity construction within these exchanges is presented. The data highlights how lack of independence and autonomy is associated with anger, and how aspirations and goal setting may facilitate positive outcomes.

King, Beehler, Vest, et al.	Traumatology	2018	USA	Not reported	VA Western New York Healthcare System–Buffalo	All HIC	Rehabilitation Psychology	***Qualitative exploration of traumatic brain injury-related beliefs among U.S. military veterans***	To explore cognitive, affective, and experiential factors that inform veterans' traumatic brain injury (TBI)-related beliefs.	Adult	TBI (veterans)	USA	Qualitative (descriptive)	Leventhal et al.'s (1997) illness perception model	Patients	22	Mixed: Interviews (open, unstructured, in-depth, semi-structured, open ended) AND questionnaires	Content analysis	Hsieh & Shannon (2005)	Not reported	Results were organized according to Leventhal et al.'s (1997) illness perception model, including veterans' self-reports regarding: (a) knowledge of TBI, labels, and symptoms (identity); (b) etiology (cause); (c) the biopsychosocial impact of TBI (consequences); (d) symptom chronicity (timeline); and (e) recovery expectancy and management strategies (controllability). Participants identified common causes of TBI, as well as acute symptoms. Uncertainty was present with regard to TBI nomenclature, recovery expectancies and trajectories, and the impact of co-occurring mental health diagnoses.
Koehmstedt, Lydick, Patel, et al.	Traumatology	2018	USA	Not reported	Center for the Study of Chronic Illness and Disability, College of Health and Human Services	All HIC	PLoS ONE	***Health status, difficulties, and desired health information and services for veterans with traumatic brain injuries and their caregivers: A qualitative investigation***	aims to provide detailed and comprehensive information on health service and information utility as well as preferred information and services in an ideal health model.	Adult	TBI (veterans)	USA	Qualitative	Not reported	Patients AND Family members, carers, signifcant others	22	Interviews (open, unstructured, in-depth, semi-structured, open ended)	Framework analysis	Gale et al. (2013)	Not reported	Participants from both groups reported difficulties finding community supportive services (support groups) in local communities. Most participants identified the need for an advocate or point-person to help guide them to needed services and provide ongoing support in the post-acute health care recovery phase. Caregivers and Veterans desired a more personalized recovery plan from their medical professionals. When describing their ideal health information and services model most identified interactivity and twenty-four-hour availability as essential components.
Kratz, Sander, Brickell, et al.	Traumatology	2017	USA	Psychologist (incl. clinical, neuro-)	Baylor College of Medicine & Harris Health System, TIRR Memorial Hermann's Brain Injury Research Center,	All HIC	Neuropsycholo gical Rehabilitation	***Traumatic brain injury caregivers: A qualitative analysis of spouse and parent perspectives on quality of life***	To examine how family caregivers of individuals with traumatic brain injury (TBI) describe their quality of life in the context of their caregiving role.	Adult	TBI	USA	Qualitative	Not reported	Family members, carers, signifcant others	52	Focus groups	Thematic analysis (Matrix)	Braun & Clarke, (2006), Catanzaro, (1988), Graneheim & Lundman, (2004), Hsieh & Shannon, (2005); Miles, Huberman, & Saldana (2014), Averill, (2002), Miles et al. (2014)	Not reported	Thematic content analysis was used to identify two main meta-themes: Caregiver Role Demands and Changes in Person with TBI. Prominent subthemes indicated that caregivers are (a) overburdened with responsibilities, (b) lack personal time and time for self-care, (c) feel that their life is interrupted or lost, (d) grieve the loss of the person with TBI, and (e) endorse anger, guilt, anxiety, and sadness. Caregivers identified a number of service needs. A number of subthemes were perceived differently by partner versus parent caregivers. The day-to-day responsibilities of being a caregiver as well as the changes in the person with the TBI present a variety of challenges and sources of distress for caregivers.
Kreitzer, Bakas, Kurowski, et al.	Traumatology	2019	USA	MD/Physician	Department of Emergency Medicine	All HIC	Journal of Head Trauma Rehabilitation	***The Experience of Caregivers Following a Moderate to Severe Traumatic Brain Injury Requiring ICU Admission***	To determine the unmet needs and concerns, burden, and description of health-related quality of life issues of informal caregivers of patients who survive a moderate to severe TBI.	Adult	TBI	USA	Qualitative	Not reported	Patients AND Family members, carers, signifcant others	32	Interviews (open, unstructured, in-depth, semi-structured, open ended)	Thematic: content analysis	Bakas et al (2009)*	Not reported	Three themes were identified in the qualitative analysis: caregiver burden, caregiver health-related quality of life, and caregiver needs for information and support. The importance of each theme differed based on the timing after injury with caregiver needs for information and support being most important at 72 hours after injury, and caregiver quality of life emerging as an important theme about 1 month after injury and caregiver burden from 3 months onwards.
Landau and Hissett	Traumatology	2008	USA	Not reported	Linking Human Systems, LLC, Boulder, CO.	All HIC	Families, Systems and Health	***Mild Traumatic Brain Injury: Impact on Identity and Ambiguous Loss in the Family***	(1)To explore the relationship between identity ambiguity on the part of the MTBI (mild TBI) subject and the partner and/or family's sense of loss of other and boundary ambiguity. (2) To Explore the Correlation of Identity Ambiguity, Boundary Ambiguity in This Situation of Ambiguous Loss. (3)To explore whether MTBI results in relational breakdown and subsequent separation or divorce because of ambiguous loss.	Adult	TBI	USA	Qualitative	Not reported	Patients AND Family members, carers, signifcant others	6	Interviews (open, in-depth, semi-structured, open ended)	Not clearly stated	Not reported	Not reported	Persons dealing with the numerous long-term cognitive and physical problems accompanying mild traumatic brain injury (MTBI) may develop a profound sense of "loss of self." This loss of self, manifest as identity ambiguity on the part of the MTBI subject may be strongly correlated with perceptions of boundary ambiguity on the part of the subject and family resulting from the situation of ambiguous loss. Early recognition and intervention may prevent relational and marital breakdown as well as unnecessary distress to family members.
Layman, Dijkers and Ashman	Traumatology	2005	USA	Not reported	Department of Physical Medicine and Rehabilitation	All HIC	Brain injury	***Exploring the impact of traumatic brain injury on the older couple: 'yes, but how much of it is age, I can't tell you …'***	To qualitatively explore the partner relationships of older couples faced with traumatic brain injury (TBI).	Adult	TBI	USA	Participatory action research	Not reported	Patients AND Family members, carers, signifcant others AND Comparison group	21	Interviews (open, unstructured, in-depth, semi-structured, open ended)	Content analysis	Sandelowski (1991); Downe-Wamboldt (1992); Patton (1990)	Not reported	Variability of relationship experiences (relationship relatedness and persistence of the partnership) was shown across groups. Aspects of relationship commitment to the partnership were revealed. Many individuals with TBI and their partners attributed various relationship changes to ageing and age-related issues and not to the TBI.
Lew, Kraft, Pogoda, et al.	Traumatology	2011	USA	Not reported	Defense and Veterans Brain Injury Center	All HIC	Journal of Rehabilitation Research and Development	***Prevalence and characteristics of driving difficulties in Operation Iraqi Freedom/Operation Enduring Freedom combat returnees***	To document and describe driving difficulties in a cohort of veterans seen within a VA Polytrauma Network Site (PNS) outpatient clinic and to determine how TBI and PTSD, two common clinical diagnoses among this cohort, are associated with self-reported driving difficulties.	Adult	TBI (veterans)	USA	Survey	Not reported	Patients	60	Comments in questionnaire AND chart review	Not clearly stated	Not reported	Not reported	Self-reported driving problems were common among OIF/OEF returnees. Respondents who had a diagnosis of PTSD (with or without TBI) reported the most severe driving difficulties since returning from deployment. The association between PTSD and driving problems warrants further investigation

Libin, Schladen, Scholten, et al.	Traumatology	2017	USA	Not reported	Washington DC Veterans Affairs Medical Center, Washington, DC, and Georgetown University Medical Center	All HIC	American Journal of Orthopsychiatry	***Perspectives of veterans with mild traumatic brain injury on community reintegration: Making sense of unplanned separation from service***	To understand how veterans with mTBI (mild TBI) perceive the experience of departure from the military, subsequent rehabilitation, and reentry into civilian life.	Adult	TBI (veterans)	USA	Phenomenography	Phenomenographic	Patients	8	Interviews (open, unstructured, in-depth, semi-structured, open ended)	Not clearly stated	Miles, Huberman & Saldana (2014)	Not reported	Two distinct patterns of thinking about community reintegration emerged. The first pattern was characterized by the perception of a need to fade one's military identity. The second pattern, conversely, advanced the perception of a need to maintain the integrity of one's military identity though living in a civilian world. These perceptions may be linked to individuals' roles while in the military and whether violent acts were committed in carrying out the mission of service, acts not consonant with positive self-appraisal in the civilian world. The crisis of unplanned, involuntary separation from the military was universally perceived as a crisis equal to that of the precipitating injury itself. The perception that civilians lacked understanding of veterans' military past and their current transition set up expectations for interactions with health care providers, as well as greatly impacting relationships with friend and family. Our veterans' shared perceptions support existing mandates for greater dissemination of military culture training to health care providers serving veterans both at VA and military facilities as well as in the civilian community at large.
Lorenz	Traumatology	2010	USA	Not reported	Institute for Behavioral Health, Heller School for Social Policy and Management	N/A	Sociology of Health and Illness	***Discovering a new identity after brain injury***	This paper seeks to close the gap in the literature on the lived experience of an acquired brain injury survivor 17 years after her injury by exploring her search for a new identity, as seen through her photographs and our conversation about them.	Adult	TBI	USA	Narrative inquiry	Not reported	Patients	1	Mixed: Interviews (open, in-depth, semi-structured, open ended) AND Photovoice	Narrative analysis	Riesman (2007)	Not reported	Study findings reveal that identity issues of importance to brain injury survivors can include (1) learning the new, post-brain injury self, and (2) building a new identity whose multiple, partial identities include (a) the new brain injured self, (b) an old self (with its residual strengths), and (c) a self who does meaningful activities (e.g. parenting, partnering, art, gardening, volunteering, helping others, or paid work). Study results suggest that using visual research methods can help to put biographical disruption such as brain injury into perspective as a life lived.
Lundine, Utz, Jacob, et al.	Traumatology	2019	USA	Research-clinician	Department of Speech and Hearing Science; Division of Clinical Therapies and Inpatient Rehabilitation Program	All HIC	Journal of Pediatric Rehabilitation Medicine	***Putting the person in person-centered care: Stakeholder experiences in pediatric traumatic brain injury***	To describe the perspectives and experiences of survivors of pediatric traumatic brain injury (pTBI), their caregivers, and the medical professionals who serve them.	Pediatric	TBI	USA	Participatory action research	Not reported	Patients AND Family members, carers, signifcant others AND clinicians (HCPs)	13	Interviews (open, unstructured, in-depth, semi-structured, open ended)	Thematic: Content analysis	Braun & Clarke (2006)	COREQ	This study found that survivors of pTBI and their caregivers commonly noted later developing issues, social challenges, and difficulty in post-rehabilitation transitions. In addition, both caregivers and medical professionals reported the need for improved follow-up with survivors, evidence-based research, and medical and therapy providers with specific training on pTBI.
Marsh, Matlock, Maertens, et al.	Traumatology	2020	USA	Research assistant	Adult and Child Consortium for Health Outcomes Research and Delivery Science (ACCORDS)	All HIC	Journal of Neurosurgery: Pediatrics	***Parental involvement in decision making about intracranial pressure monitor placement in children with traumatic brain injury***	To identify the perspectives and decisional or information needs of parents whose child sustained a TBI and may require an ICP monitor.	Pediatric	TBI	USA	Qualitative	Not reported	Family members, carers, signifcant others AND HCPs (inc. NSx)	38	Interviews (open, unstructured, in-depth, semi-structured, open ended)	Content analysis	Hsieh & Shannon (2005)	Not reported	Overall, the authors found concordance between and among parents and clinicians about parental involvement in ICP monitor decision making. Parents and clinicians agreed that decision making about ICP monitoring in children who have suffered TBI is not and should not be shared between the parents and clinicians. The concordance was represented in 3 emergent themes. Parents wanted transparency, communication, and information (theme 2), but the life-threatening context of this decision (theme 1) created an environment where all involved reflected a clear preference for paternalism (theme 3).
Melnick, Shafer, Rodulfo, et al.	Traumatology	2015	USA	MD/Physician	Department of Emergency Medicine	Mixed HIC/LMIC	Academic Emergency Medicine	***Understanding overuse of computed tomography for minor head injury in the emergency department: A triangulated qualitative study***	To identify nonclinical, human factors that promote or inhibit the appropriate use of CT in patients presenting to the emergency department (ED) with minor head injury.	Unclear	TBI (concussion)	USA	Qualitative	Not reported	Patients AND HCPs (emergency care providers)	44	Mixed: Focus groups AND semi structured interviews AND observations	Grounded theory analysis/constant comparison/open and axial/selective coding	Bradley, Curry, Devers (2007)	Not reported	Five core domains emerged from the analysis: establishing trust, anxiety (patient and provider), constraints related to ED practice, the influence of others, and patient expectations. Key themes within these domains included patient engagement, provider confidence and experience, ability to identify and manage patient anxiety, time constraints, concussion knowledge gap, influence of health care providers, and patient expectations to get a CT. Despite high-quality evidence informing use of CT in minor head injury, multiple factors influence the decision to obtain CT in practice. Identifying and disseminating approaches and designing systems that help clinicians establish trust and manage uncertainty within the ED context could optimize CT use in minor head injury
Miley, Fisher, Moscato, et al.	Traumatology	2020	USA	Not reported	Division of Pediatric Rehabilitation Medicine	All HIC	Disability and Rehabilitation	***A mixed-methods analysis examining child and family needs following early brain injury***	To understand child and family needs following TBI in early childhood	Pediatric	TBI	USA	Mixed methods (Convergent parallel design)	Not reported	Family members, carers, signifcant others	22	Mixed: Focus groups AND key informant interviews, AND questionnaires	Thematic analysis (inductive)	Krueger (1998)	Not reported	Results indicated some families continue to experience unresolved concerns relating to the child's injury, caregiver wellbeing, and the family system after early TBI, including notable variation in caregiver reported psychological distress and parenting stress. Caregivers noted unmet needs post-injury, such as child behavior management and caregiver stress and coping. Early TBI can have a long-term impact on the child, caregivers, and family system. Addressing the needs of the whole family system in intervention and rehabilitation efforts may optimize outcomes following early TBI.
Moore, Kiatchai, Ayyagari, et al.	Traumatology	2017	USA	Not reported	School of Social Work	All HIC	Brain Injury	***Targeted areas for improving health literacy after traumatic brain injury***	To develop a framework to identify targeted areas for improving health literacy for caregivers after traumatic brain injury (TBI).	Unclear	TBI	USA	Qualitative	Not reported	Family members, carers, signifcant others	23	Interviews (open, unstructured, in-depth, semi-structured, open ended)	Content analysis	Bradley, Currey & Devers (2007), Hsieh & Shannon (2005), Graneheim & Lundman (2004)	Not reported	Four types of communication patterns were identified: formal, informal, indirect and caregiver-initiated. Informal and caregiver-initiated communication were the most common. All caregivers reported confusion about their family member's condition, care plan or discharge plan. Caregivers were not able to define commonly used terms in discharge instructions, and were confused by formatting and medical language. Caregivers were not aware of expected caregiving roles upon discharge. Conceptualizing findings within a family-Centered care model, we offer specific strategies to improve health communication and caregiver capacity building to enhance health literacy.
Moore, Robinson, Mink, et al.	Traumatology	2015	USA	Not reported	Harborview Injury Prevention and Research Center; School of Social Work	All HIC	Pediatric Critical Care Medicine	***Developing a family-centered care model for critical care after pediatric traumatic brain injury***	To examine the family experience of critical care after pediatric traumatic brain injury in order to develop a model of specific factors associated with family-centered care.	Pediatric	TBI	USA	Qualitative	Not reported	Family members, carers, signifcant others	15	Interviews (open, unstructured, in-depth, semi-structured, open ended)	Content analysis	Graneheim & Lundman (2004)	Not reported	Three major themes emerged: 1) thorough, timely, compassionate communication, 2) capacity building for families, providers, and facilities, and 3) coordination of care transitions. Participants reported valuing detailed, frequent communication that set realistic expectations and prepared them for decision making and outcomes. Areas for capacity building included strategies to increase provider cultural humility, parent participation in care, and institutional flexibility. Coordinated care transitions, including continuity of information and maintenance of partnerships with families and care teams, were highlighted. Participants who were not primarily English speaking reported particular difficulty with communication, cultural understanding, and coordinated transition

Moskowitz, Quinn, Khan, et al.	Traumatology	2018	USA	Not reported	Department of Neurology	All HIC	MDM Policy and Practice	***Should We Use the IMPACT-Model for the Outcome Prognostication of TBI Patients? A Qualitative Study Assessing Physicians' Perceptions***	To examine physicians' perceptions, and use of the IMPACT-model, the most widely validated ciTBI outcome model, and explored physicians' preferences for communicating prognostic information towards families.	N/A	TBI	USA	Qualitative	Not reported	HCPs (inc. NSx)	20	Interviews (open, unstructured, in-depth, semi-structured, open ended)	Type unclear	Thurston et al (2014)	Not reported	Only 12 physicians (60%) expressed awareness of the IMPACT-model; two stated that they "barely" knew the model. Seven physicians indicated using the model at least some of the time in clinical practice, although none used it exclusively to derive a patient's prognosis. Four major themes emerged: the IMPACT-model is intended for research but should not be applied to individual patients; mistrust in the IMPACT-model derivation data; the IMPACT-model is helpful in reducing prognostic variability among physicians; concern that statistical models may mislead families about a patient's prognosis
Oyesanya, Arulselvam, Thompson, et al.	Traumatology	2021	USA	Not reported	Shepherd Center, Crawford Research Institute; School of Nursing	All HIC	Disability and Rehabilitation	***Health, wellness, and safety concerns of persons with moderate-to-severe traumatic brain injury and their family caregivers: a qualitative content analysis***	To explore health, wellness, and safety concerns after discharge home from inpatient rehabilitation from the perspectives of persons with TBI who are ≥75% independent in activities of daily living and their family caregivers.	Adult	TBI	USA	Qualitative	Not reported	Patients AND Family members, carers, signifcant others	27	Interviews (open, unstructured, in-depth, semi-structured, open ended)	Content analysis	Hsieh & Shannon (2005)	COREQ	Seven themes related to health, wellness, and safety encompassed participants' experience. Health themes included: (1) attempting to manage medications and (2) navigating mental health difficulties. Wellness themes included: (1) working to stay physically active, (2) dealing with sleep and sleeplessness, and (3) adjusting to changing social relationships. Safety themes were: (1) addressing mobility challenges and (2) compensating for complications with cognitive functioning.
Oyesanya and Bowers	Traumatology	2017	USA	RN/Nurse	Shepherd Center, Crawford Research Institute	All HIC	Journal of Family Nursing	***Managing Visitors During the Hospital Stay: The Experience of Family Caregivers of Patients With Traumatic Brain Injury***	To describe family caregivers' experience of visitors while the patient with moderate-to-severe TBI is hospitalized	Adult	TBI	USA	Qualitative	Not reported	Family members, carers, signifcant others	16	Interviews (open, unstructured, in-depth, semi-structured, open ended)	Grounded theory analysis/constant comparison/open and axial/selective coding	Strauss and Corbin (1998)	Not reported	Findings showed family caregivers manage welcome and unwelcome visitors throughout the hospital stay to protect the patient's physical and emotional safety and to conserve their own energy. Staff had limited involvement in management of unwelcome visitors. These findings have practice implications for educating hospital staff about the providing family nursing and assisting families to manage unwelcome visitors and policy implications for improving hospital visiting policies.
Oyesanya and Bowers	Traumatology	2017	USA	RN/Nurse	Shepherd Center	All HIC	Qualitative Health Research	***"I'm Trying to Be the Safety Net": Family Protection of Patients with Moderate-To-Severe TBI during the Hospital Stay***	To describe family caregivers' experience of protecting patients with TBI during the hospital stay	Adult	TBI	USA	Grounded theory	Symbolic interactionism	Family members, carers, signifcant others	16	Interviews (open, unstructured, in-depth, semi-structured, open ended)	Grounded theory analysis/constant comparison/open, axial and/or selective coding	Strauss and Corbin (1998)	Not reported	Findings showed family caregivers worked to protect the patient's physical and emotional safety, using the following strategies: (a) influencing the selection of staff, (b) breaking the patient's bad habits, (c) anticipating how to orchestrate the home environment; (d) connecting on an emotional level, (e) managing visitors, and (f) connecting on an emotional level. The findings have practice implications for educating interdisciplinary health care providers about the experience of family caregivers and for developing an adversarial alliance between health care providers and family caregivers during the hospital stay to improve support provided to them.
Oyesanya, Loflin, Harris, et al.	Traumatology	2021	USA	RN/Nurse	School of Nursing	All HIC	Clinical Rehabilitation	***"Just tell me in a simple way": A qualitative study on opportunities to improve the transition from acute hospital care to home from the perspectives of patients with traumatic brain injury, families, and providers***	To identify areas to improve the transition from acute hospital care to home for patients with traumatic brain injury and their families.	Adult	TBI	USA	Qualitative (descriptive)	Not reported	Patients AND Family members, carers, signifcant other AND HCPs	36	Interviews (open, unstructured, in-depth, semi-structured, open ended)	Content analysis	Hsieh & Shannon (2005)	Not reported	Findings showed patients, families, and providers recommend three areas for improvement in the transition home from acute hospital care, described in three themes. Theme 1 was "improving patient and family education," with the following sub-themes: (a) TBI-related information and (b) discharge preparation. Theme 2 was "additional provider guidance," with the following sub-themes: (a) communication about patient's recovery timeline and (b) recovery roadmap development. Theme 3 was "increasing systems-level support," with the following sub-themes: (a) scheduling follow-up appointments, (b) using a patient navigator, (c) creating a provider follow-up structure, (d) linking pre-discharge care with post-discharge resources, and (e) addressing social issues.
Oyesanya, Thompson, Arulselvam, et al.	Traumatology	2021	USA	RN/Nurse	School of Nursing; Shepherd Center	All HIC	Assistive Technology	***Technology and TBI: Perspectives of persons with TBI and their family caregivers on technology solutions to address health, wellness, and safety concerns***	To investigate views of technology in the context of managing one's everyday life from the perspectives of persons with TBI and their family caregivers.	Adult	TBI	USA	Qualitative	Not reported	Patients AND Family members, carers, signifcant others	27	Interviews (open, unstructured, in-depth, semi-structured, open ended)	Content analysis	Hsieh & Shannon (2005) and Elo & Kyngäs (2008)	COREQ	The major themes that emerged were: 1) views of technology, 2) influence of technology on daily life, 3) desired technology solutions to address health, wellness, and safety concerns, and 4) barriers to adopting technology solutions. To address ongoing concerns, persons with TBI and family caregivers expressed a desire to have a patient portal to share information and communicate with providers, motion sensing devices to provide guidance for mobility, on-demand access to in-home rehabilitation therapy, access to a "social gym" where the persons with TBI could virtually exercise and interact with others, and technologies that help with sleep and facilitate peer support
Oyesanya, Harris Walker, Loflin, et al.	Traumatology	2021	USA	Not reported	School of Nursing	All HIC	Journal of Integrated Care	***Negotiating the transition from acute hospital care to home: perspectives of patients with traumatic brain injury, caregivers and healthcare providers***	To explore experiences transitioning home from acute hospital care from perspectives of younger traumatic brain injury (TBI) patients, family caregivers and healthcare providers (HCPs).	Adult	TBI	USA	Qualitative (descriptive)	Not reported	Patients AND Family members, carers, signifcant other AND HCPs	36	Interviews (open, unstructured, in-depth, semi-structured, open ended)	Content analysis	Hsieh & Shannon (2005)	Not reported	The transition from hospital to home was described as a negotiation, finding a way through these obstacles: (1) preparing for discharge home during acute hospital care; (2) navigating transitions in healthcare and health; (3) addressing recovery concerns, and (4) setting goals to return to normal. Factors influencing the negotiation process included social support, health-related knowledge or training, coping mechanisms, financial stability, and home environment stability.
Padilla	Traumatology	2003	USA	Occupational therapist	Department of Occupational Therapy	N/A	American Journal of Occupational Therapy	***Clara: A phenomenology of disability***	To describe the lived experience of disability of a woman who sustained a head injury 21 years ago.	Adult	TBI	USA	Phenomenology	Phenomenological	Patients	1	Mixed: Interviews (open, unstructured, in-depth, semi-structured, open ended) AND emails	Phenomenological analysis (iterative process of analytical induction)	Moustakas (1994) and Karlsson (1995).	Not reported	The themes of nostalgia, abandonment, and hope emerged through this collaborative process. A shift in view of life was noted in the participant as the reflective process of this study unfolded, suggesting a phenomenological collaboration between patient and therapist may engender a more genuine connection in which personal meaning is authentically the cornerstone of occupation-centered treatment.
Pasek, Locasto, Reichard, et al.	Traumatology	2015	USA	RN/Nurse	Pain/Pediatric Intensive Care Unit, Children's Hospital of Pittsburgh of University of Pittsburgh Medical Center (UPMC)	All HIC	Clinical nurse specialist CNS	***The headache electronic diary for children with concussion***	To (1) develop a prospective, real time, age-appropriate, and appealing prototype of an electronic headache pain diary for children and (2) evaluate the clinical feasibility and utility of the diary for the assessment and documentation of concussion headache.	Adolescents	TBI (concussion)	USA	Mixed methods	Not reported	Patients	28	Interviews (open, unstructured, in-depth, semi-structured, open ended)	Thematic (descriptive)	Not reported	Not reported	The phase 1 sample recommended a diary with calendar and clock functions, head views, menus (e.g., pain descriptors), soft colors, a choice of pain assessment scales, and the ability to personalize it. All of the children thought that the new Headache Electronic Diary for Children With Concussion (HED-CC) would be helpful to track their headache and reported that other children with concussions would be likely to use it. Participants recommended improvement of the head views and clock function.

Portanova, Dreesmann, Moore, et al.	Traumatology	2021	USA	RN/Nurse	University of Washington	All HIC	Pain Management Nursing	***Pain and Symptoms after Mild Traumatic Brain Injury: Should Technology Play a Role in Self-Management?***	To explore self-management strategies in order to inform design of an informatics tool to support self-management of pain and symptoms following mTBI.	Adult	TBI	USA	Qualitative (descriptive)	Not reported	Patients	7	Interviews (open, unstructured, in-depth, semi-structured, open ended)	Qualitative descriptive analysis	Sandelowski (2000), Neergaard (2009), Elliot and Timulak (2005)	Not reported	Seven female participants completed interviews. Participants described difficulty coping with symptoms, limited access to or knowledge of treatments and trial and error with compensatory strategies. These challenges often led to difficulty keeping up with work, school and other commitments. All participants indicated that they were interested in a tool that addresses pain, memory and concentration. The main features that participants wanted were pain and symptom tracking as well as suggestions based on tracking information. Sharing their recovery with others, including customizing what was to be shared, was wanted as a feature in a future tool. An app on the phone was the most popular platform, but voice recognition was also recommended to reduce screen time.
Powell, Gomez, Gau, et al.	Traumatology	2020	USA	Not reported	Center on Brain Injury Research and Training	All HIC	Brain Injury	***A survey of the training experiences and needs of paraprofessionals serving adults with brain injury***	1) What types of paraprofessionals receive for working with adults with brain injury 2) What are the barriers and challenges to training paraprofessionals 3) What are the challenges paraprofessionals face in working with this population 4) What are the most important paraprofessional training topics 5) Do paraprofessionals have the knowledge and skills needed to serve this population?	Adult	TBI	USA	Survey	Not reported	Patients AND Family members, carers, signifcant other AND HCPs	136	Open ended questions AND follow up interviews	Other: reviewed qualitatively for common themes and unique topics.	Not reported	Not reported	1)Paraprofessionals most frequently received on the job training, workshops and self study. preferences for training included workshops, on the job training, small groups, certified brain injury specialist training and on-line learning. Most frequently identified barriers were time and funding. 2)Challenges to training included frequent staff turnover, lack of well-developed training, increasing need for services and reduced resources, lack of experience in training and population mix with other conditions. 3)Challenges faced when working with brain-injured patients were grouped into themes: cognitive-communication impairments, behavioral challenges, psycho-emotional changes, co-occurring disorders, effects on the person and their daily life, person-centered approaches, paraprofessional knowledge and skills and adequate resources and support 4) The most important training topics for paraprofessionals were communication and behavior changes, impact on daily life and how to assist in getting back to daily life. Most important topics for patients were mental health, impact on daily life, behavior challenges. Family members identified self-identity, changes in thinking and getting back to daily life as top priorities. 5) Professionals felt paraprofessionals had significantly lower levels of paraprofessional knowledge and confidence compared to paraprofessionals themselves.
Quinn, Moskowitz, Khan, et al.	Traumatology	2017	USA	Not reported	Department of Neurology (Neurocritical Care)	All HIC	Neurocritical Care	***What Families Need and Physicians Deliver: Contrasting Communication Preferences Between Surrogate Decision-Makers and Physicians During Outcome Prognostication in Critically Ill TBI Patients***	To explore key communication preferences and practices by stakeholders (surrogates and physicians) for the outcome prognostication during goals-of-care discussions for ci-TBI patients	Adult	TBI	USA	Qualitative	Deductive and inductive	Other: Surrogate decision makers AND HCPs (inc. NSx)	36	Interviews (open, unstructured, in-depth, semi-structured, open ended)	Other: deductive and inductive.	Not reported	Not reported	Identified major themes by surrogate and physician. Only one concordant theme ("no false hope"). Discordant themes around the role of uncertainty (surrogates = frustrating; physicians = natural part of communication) and numeric estimates (physicians prefer qualitative terms, surrogates prefer numeric estimates). Physicians felt prediction scores are research tools and should not be applied to individual patients. Surrogates desired acceptance of decisions once made and prognostication with compassion.
Rao, Bechtold, Arciniegas, et al.	Traumatology	2020	USA	Subspecialist in Behavioral Neurology & Neuropsychiatry	Dept Psychiatry and behavioral sciences	All HIC	Brain Injury	***Establishment of a Patient-Centered Outcomes Research Network for Individuals with TBI and Neuropsychiatric Symptoms***	To determine facilitators and barriers in management of neuropsychiatric symptoms after TBI; identify strategies for maintaining a TBI PCOR network; enumerating research topics related to TBI neuropsychiatry; highlighting policy changes related to TBI neuropsychiatry	Adult	Traumatic brain injury; Neuropsychiatric conditions post TBI	USA	Qualitative	Not reported	Patients AND Family members, carers, signifcant others AND HCPs AND non-clinical providers	29	Focus groups	Other: Codes based on themes that emerged from the data on four a priori determined domains	Not reported	Not reported	Participants identified barriers such as: stigma and poor insurance coverage. Facilitators in management included treatment focused on education and a comprehensive caregiver plan.
Rawlins, Johnson, Register-Mihalik, et al.	Traumatology	2020	USA	Not reported	University of Georgia	All HIC	Military medicine	***United States Air Force Academy Cadets' Perceived Costs of Concussion Disclosure***	To explore the factors influencing concussion reporting in US air force academy cadets	Adult	TBI (concussion)	USA	Mixed methods	Hermeneutic phenomenological approach	Other: Patients AND Comparison group	34	Interviews (open, unstructured, in-depth, semi-structured, open ended)	Other: 5 cycle process leading to codebook formation	Anderson (2011); Wertz et al (2011)	Not reported	8 themes were generated. The article focuses mainly on one: The perceived costs following a concussion theme, and its subthemes - perceived costs to physical fitness, military career aspirations, pilot qualifications, sport, reputation, academics, lack of time.
Rennie and Goforth	Traumatology	2020	USA	Not reported	University of New Mexico Health Services Center	All HIC	Rural Special Education Quarterly	***A Qualitative Examination of Social Changes and Challenges in Rural Adolescents With Traumatic Brain Injury***	To explore adolescents' social domain, cognitive and social ability, quality of life, and the changes they experienced following the injury. Given the lack of research focusing on factors associated with being in rural communities, we also explored social and community supports, resources, and service utilization, and perceived benefits of obtained services.	Adolescents	TBI	USA	Grounded theory (flexible application)	Pragmatic epistemological position	Patients AND Family members, carers, signifcant others	7	Mixed: In-depth interviews AND questionnaires	Grounded theory analysis/constant comparison/open, axial and/or selective coding	Bryman (2001)	Not reported	Four umbrella themes emerged, which were personal change, environmental response, social needs, and facilitators and inhibitors of intentional changes (FIICs). These themes, and their subthemes, capture both assets and needs as they develop over time and provide guidance for both researchers and practitioners working with adolescents who have a TBI.
Roscigno and Swanson	Traumatology	2011	USA	RN/Nurse	College of nursing	All HIC	Qualitative Health Research	***Parents' experiences following children's moderate to severe traumatic brain injury: A clash of cultures***	To explore the common experiences of parents whose children were diagnosed with moderate to severe TBI.	Pediatric	TBI	USA	Phenomenology (Descriptive)	Not reported	Family members, carers, signifcant others	42	Interviews (open, unstructured, in-depth, semi-structured, open ended)	Phenomenological analysis	Colaizzi (1978)	Not reported	Parent themes were: (a) grateful to still have my child; (b) grieving for the child I knew; (c) running on nerves; and (d) grappling to get what your child and family need. Parents reported cultural barriers because of others' misunderstandings.
Roscigno, Fleig and Knafl	Traumatology	2015	USA	RN/Nurse	School of Nursing	All HIC	Disability and Rehabilitation	***Parent management of the school reintegration needs of children and youth following moderate or severe traumatic brain injury***	Understand how both unique and common experience during children's school re-integration were explained by parents to influence the family.	Pediatric	TBI	USA	Phenomenology	Not reported	Family members, carers, signifcant others	42	Interviews (open, unstructured, in-depth, semi-structured, open ended)	Content analysis	Brofenbrenner (1989)	Not reported	Parents' school negotiation themes included: 1) legal versus moral basis for helping the child; 2) inappropriate state and local services that did not consider needs specific to TBI; and, 3) involvement in planning, implementing, and evaluating the child's education plan. Parents perceived that coordinated and collaboration leadership with school personnel lessened families' workload. Families who home-schooled had unique challenges.

Roscigno, Grant, Savage, et al.	Traumatology	2013	USA	Not reported	School of Nursing	All HIC	Brain Injury	***Parent perceptions of early prognostic encounters following children's severe traumatic brain injury: 'Locked up in this cage of absolute horror'***	to illustrate how the presentation of prognostic information might be interpreted differently by parents than any providers in early acute care might intend.	Pediatric	TBI	USA	Discourse analysis	Ethnography of speaking	Family members, carers, signifcant others	27	Interviews (open, unstructured, in-depth, semi-structured, open ended) - secondary analysis	Discourse analysis - SPEAKING framework	Hymes (1972)	Not reported	Parents perceived that: (a) parents were disadvantaged by provider delivery; (b) negative outcome values dominated some provider's talk; (c) truth-telling involves providers acknowledging all possibilities; (d) framing the child's prognosis with negative medical certainty when there is some uncertainty could damage parent-provider relationships; (e) parents needed to remain optimistic; and (f) children's outcomes could differ from providers' early acute care prognostications.
Roscigno, Savage, Grant, et al.	Traumatology	2013	USA	Not reported	College of Nursing,	All HIC	Social Science and Medicine	***How healthcare provider talk with parents of children following severe traumatic brain injury is perceived in early acute care***	to explore and discover what parents were trying to tell us explicitly and implicitly about the talk in these settings.	Pediatric	TBI	USA	Discourse analysis	Ethnography of speaking	Family members, carers, signifcant others	27	Interviews (open, unstructured, in-depth, semi-structured, open ended) - secondary analysis	Discourse analysis	Hymes (1972)	Not reported	We found the human constructed talk factors that parents reacted to were: a) access to the child, which is where information was; b) regular discussions with key personnel; c) updated information that is explained; d) differing expectations for talk in this context; and, e) perceived parental involvement in decisions. We found that the organization and nature of providers' talk with parents was perceived by parents to positively or negatively shape their early acute care identities in these locales, which influenced how they viewed these locales as places that either supported them and decreased their workload or discounted them and increased their workload for getting what they needed.
Roscigno, Swanson, Vavilala, et al.	Traumatology	2011	USA	RN/Nurse	College of Nursing,	All HIC	Brain injury	***Children's longing for everydayness: life following traumatic brain injury in the USA***	To explore themes of children's experiences following moderate to severe TBI.	Pediatric	TBI	USA	Phenomenology (Descriptive)	Interpretive paradigm	Patients	39	Interviews (open, unstructured, in-depth, semi-structured, open ended)	Phenomenological analysis	Collaizzi (1978)	Not reported	Six themes emerged: 1) it is like waking up in a bad dream; 2) I thought going home would get me back to my old life, but it did not; 3) everything is such hard work; 4) you feel like you will never be like the person you were before; 5) it is not all bad; and 6) some people get it, but many people do not.
Rotondi, Sinkule, Balzer, et al.	Traumatology	2007	USA	Not reported	Department of Critical Care Medicine	All HIC	Journal of Head Trauma Rehabilitation	***A qualitative needs assessment of persons who have experienced traumatic brain injury and their primary family caregivers***	To determine the expressed needs of persons with traumatic brain injury (TBI) and their primary family caregivers.	Adult	TBI	USA	Qualitative	Not reported	Patients AND Family members, carers, signifcant others	165	Interviews (open, unstructured, in-depth, semi-structured, open ended)	Content analysis	Miles & Huberman (1994)	Not reported	Respondents described their needs via phases that paralleled transitions in settings, treatments, and responsibilities (i.e., acute care, in-patient rehabilitation, return home, and living in the community). Prominent themes during in-patient phases included provider quality, emotional support, and understanding the injuries. Prominent themes during the latter 2 phases included guidance, life planning, community integration, and behavioral and emotional issues.
Saban, Hogan, Hogan, et al.	Traumatology	2015	USA	RN/Nurse	Center of Innovation for Complex Chronic Healthcare, Marcella Niehoff School of Nursing	All HIC	Rehabilitation nursing : the official journal of the Association of Rehabilitation Nurses	***He Looks Normal But ... Challenges of Family Caregivers of Veterans Diagnosed with a Traumatic Brain Injury***	To describe the experience of family caregivers providing care to veterans with traumatic brain injury (TBI)	Adult	TBI	USA	Qualitative (descriptive)	Not reported	Family members, carers, signifcant others	7	Interviews (open, in-depth, semi-structured, open ended)	Content analysis	Elo & Kyngas (2008); Hsieh & Shannon (2005)	Not reported	Findings resulted in the key concept phrased by participants as "He looks normal but." This phrase conceptualizes the participants' description of their experience caring for a brain injured veteran who could appear normal to others but the caregiver's description revealed substantial cognitive, social, and emotional deficits. Concepts include (a) Becoming aware of his disabilities, (b) Observing his troubling symptoms, (c) Dealing with his memory loss, (d) Being fearful of his anger, (e) Sensing his loneliness, (f) Acknowledging the effects on the children, and (g) Managing the best I can.
Sample, Greene and Johns	Traumatology	2012	USA	Not reported	Department of Occupational Therapy	All HIC	Brain Injury	***Life-Bombing-Injury-Life: A qualitative follow-up study of Oklahoma City bombing survivors with TBI***	To learn about and come to an understanding of the recovery process and outcomes experienced by the survivors of the 1995 Oklahoma City bombing, who sustained a traumatic brain injury (TBI) along with other injuries in the blast.	Mixed	TBI	USA	Phenomenology	Phenomenological	Patients AND Family members, carers, signifcant others	20	Mixed: Interviews, field notes and supplemental written and filmed materials	Content analysis	Not reported	Not reported	The researchers' data analysis yielded four themes (Trauma-Healing-Support; What TBI?; How I went back to work and life; Now I really need assistance!) that represented the content and meanings of the interviews and supplemental data.
Sander, Pappadis, Clark, et al.	Traumatology	2011	USA	Not reported	Department of Physical Medicine and Rehabilitation	All HIC	Journal of Head Trauma Rehabilitation	***Perceptions of community integration in an ethnically diverse sample***	To investigate the meaning of community integration in an ethnically diverse sample.	Adult	TBI	USA	Mixed methods	Not reported	Patients	167	Mixed: Interviews (open, unstructured, in-depth, semi-structured, open ended) AND questionnaires	Grounded theory analysis/constant comparison/open, axial and/or selective coding	Glaser & Strauss (1967)	Not reported	Resulting themes indicated that feeling part of the community was related to type and quality of community relationships, perceived safety and security, active involvement, feeling included and respected, and familiarity with the community. Themes regarding barriers included the following: environmental and social barriers; injury-related cognitive and physical changes; dissimilarities to others; relocation; and financial issues. Blacks and Hispanics placed more emphasis on domestic activities than did whites.
Smart and Giacino	Traumatology	2015	USA	Neuropsychologist	JFK-Johnson Rehabilitation Institute and the New Jersey Neuroscience Institute; Department of Psychology	All HIC	NeuroRehabilitation	***Exploring caregivers' knowledge of and receptivity toward novel diagnostic tests and treatments for persons with post-traumatic disorders of consciousness***	To gather information regarding the beliefs and opinions of caregivers regarding NTT for DOC.	Adult	TBI	USA	Qualitative	Not reported	Family members, carers, signifcant others	17	Focus groups	Grounded theory analysis/constant comparison/open, axial and/or selective coding	Gerber & Williams (1996); Lincoln and Guba (1985)	Not reported	While attitudes toward NTT were generally favorable, two main themes emerged that influenced willingness to pursue NTT: patient and caregiver-specific factors, and the acquisition/use of information to guide decision-making. While survey data suggested a lack of knowledge about NTT, qualitative data revealed that this was better explained by different standards for knowledge, i.e., anecdotal versus empirical information.
Tilden, Williams, Tucker, et al.	Traumatology	2009	USA	Not reported	Department of Gerontology/Geriatrics/Palliative Care	All HIC	Journal of Palliative Medicine	***Surgeons' attitudes and practices in the utilization of palliative and supportive care services for patients with a sudden advanced illness***	To explore surgeons' attitudes and decision-making practices regarding utilization of palliative and supportive care for patients with a sudden advanced illness from traumatic injury or physiologic insult at UAB Hospital, an American College of Surgeons certified level 1 trauma center.	N/A	TBI	USA	Not stated	Not reported	Surgeons (inc. Nsx)	9	Interviews (open, in-depth, semi-structured, open ended)	Grounded theory analysis/constant comparison/open, axial and/or selective coding	Not reported	Not reported	We organized the data using decision-making diagrams, identifying multiple pathways within the larger consultative framework. Although case-based responses exhibited variations in surgeons diagnostic or prognostic criteria, patient's location in the illness/injury trajectory, and surgeon's goals/desired outcomes; a general decision-making pathway emerged.
Todis, McCart and Glang	Traumatology	2018	USA	Not reported	Center on Brain Injury Research and Training	All HIC	NeuroRehabilitation	***Hospital to school transition following traumatic brain injury: A qualitative longitudinal study***	The objective of this study is to look closely at what transition services consist of, how they are delivered, and how they are received by educators.	Pediatric	TBI	USA	Qualitative	Not reported	Family members, carers, signifcant others AND non-clinical providers	77	Mixed: Interviews (open, unstructured, in-depth, semi-structured, open ended) AND observations	Type unclear	Not reported	Not reported	This paper presents two themes that emerged from interview and observational data collected over a 7-year period: 1) provision of hospital-school transition services is inconsistent and 2) teachers lack sufficient awareness of and training in TBI to implement hospital transition recommendations. Regardless of the type and quality of transition support, most educators in the students' schools remained unaware of the students' needs and how to meet them.

Tosch	Traumatology	1988	USA	RN/Nurse	Parkland Memorial Hospital	All HIC	The Journal of neuroscience nursing : journal of the American Association of Neuroscience Nurses	***Patients' recollections of their posttraumatic coma***	To explore recollections of posttraumatic coma as viewed retrospectively by the patient after regaining consciousness and prior to discharge from the hospital.	Adult	TBI	USA	Qualitative	Sigmund Freud's psychoanalytical theory	Patients		15	Interviews (open, unstructured, in-depth, semi-structured, open ended)	Other: Qualitative techniques	Not reported	Not reported	Based on results of this investigation, the following conclusions were drawn for patients in this setting: 1. Many patients were unable to recall the experience of unconsciousness. 2. Experiences of coma were recalled by patients as themes of being held prisoner, sensory experiences and death. 3. Physical touch, reassuring voice tone, and information relating to the situation were recalled by patients as helpful. 4. Body pinches were recalled by patients as not helpful. 5. Type of intracranial lesion sustained as a result of closed head injury and the GCS score did not ap- pear to affect recall response of coma for patients. 6. Length of the posttraumatic coma of patients appeared to affect recall of unconsciousness.
Voris and Steinkopf	Traumatology	2019	USA	Not reported	Department of Social Science and Education,	All HIC	Marriage and Family Review	***Suffering in the Shadows: Interviews with Wives of Combat Veterans Suffering from Post-Traumatic Stress Disorder and/or Traumatic Brain Injury***	To examine what it means to be a wife of a combat veteran who suffers from post-traumatic stress disorder (PTSD) and/or traumatic brain injury (TBI).	Adult	TBI (veterans; PTSD)	USA	Qualitative	Post-structural feminist	Family members, carers, signifcant others		7	Interviews (open, unstructured, in-depth, semi-structured, open ended)	Type unclear	Alvesson & Sköldberg (2009)	Not reported	Our findings indicate these women—who experience tremendous emotional, financial, and social challenges that arise from being the caregiver for their husbands—feel isolated from and abandoned by both the military community and the civilian community. Furthermore, the social and emotional disconnection of these women experience amplify the stresses they confront in daily life—stresses that are unique to their relationship to being with a combat veteran spouse who has PTSD and/or TBI. We argue future research should build upon this exploratory study to better understand how both the military community and the civilian community impact the subjectivity of these women and their efforts to reintegrate themselves and their families into civilian life.
Wan and Nasr	Traumatology	2021	USA	RN/Nurse	Stanford Children's Hospital,	All HIC	Journal of Clinical Nursing	***Return to learn: An ethnographic study of adolescent young adults returning to school post-concussion***	To better understand the perceptions of parent-adolescent dyads as male and female adolescents returned to learn after sustaining a concussion.	Adolescents	TBI (concussion)	USA	Ethnography	Bronfenbrenner's Ecological Systems Theory	Patients AND Family members, carers, signifcant others		20	Interviews (open, unstructured, in-depth, semi-structured, open ended)	Thematic analysis	De Santis & Ugarriza 2000	COREQ	Three categories were identified: adolescents feeling misunderstood by school staff about their injuries, adolescents feeling overwhelmed by their injuries, and academic accommodations lacking clarity and implementation. The overarching theme that emerged was clear: Parents and adolescents lacked consistent guidelines for the adolescents' return to learn after a concussion.
Weber Rawlins, Welch Bacon, Tomporowski, et al.	Traumatology	2021	USA	ATC (Certified athletic trainer)	Department of Interdisciplinary Health Sciences	All HIC	Journal of athletic training	***A Qualitative Analysis of Concussion-Reporting Behavior in Collegiate Student-Athletes With a History of Sport-Related Concussion***	To explore student-athletes' perspectives regarding factors that may influence the reporting of sport-related concussion.	Adult	TBI (concussion)	USA	Phenomenology (Hermeneutic)	Hermeneutic phenomenological	Patients		17	Interviews (open, unstructured, in-depth, semi-structured, open ended)	Other: Intuitive inquiry	Anderson (2011); Wertz et al (2011)	Not reported	We discovered 3 themes. Participants discussed concussion perceptions by describing their understanding of a concussion, their own injury experiences, and their perceptions of symptom severity and duration. Regarding reporting behavior, participants described an order of individuals with whom they would speak, symptoms present in order to report (e.g., feeling different from normal), immediate reactions, and influential factors for mitigating short- and long-term consequences. Lastly, participants discussed the value of support systems, such as how coaches can both positively and negatively influence reporting and athletic trainer involvement.
Wiley, Schultheis, Francis, et al.	Traumatology	2020	USA	MD/Physician	Yale University	All HIC	Academic Pediatrics	***Parents' Perceptions of Infant Crying: A Possible Path to Preventing Abusive Head Trauma***	to explore parental beliefs, emotions, and experiences regarding infant crying, and its relationship to abusive head trauma through interviews with new parents at the time of the birth of their newborns and at subsequent interviews 8 to 12 weeks after hospital discharge.	Pediatric	TBI	USA	Grounded theory	Not reported	Family members, carers, signifcant others		25	Interviews (open, unstructured, in-depth, semi-structured, open ended)	Grounded theory analysis/constant comparison/open and axial/selective coding	Strauss & Corbin (1990)	Not reported	Four themes emerged from the initial interviews: 1) previous experience is helpful to manage infant crying, 2) babies cry for a reason, 3) shaking an infant is incomprehensible to parents, and 4) the role of safety planning to manage frustration with crying. Analysis of the subsequent interviews revealed 2 additional themes: 1) use of supports for infant crying (e.g., technology and physician advice) and 2) effects of the audio clip on caregiving practices.
Brunner, Hemsley, Dann, et al.	Traumatology	2018	Australia	Not reported	Speech Pathology	All HIC	Brain Injury	***Hashtag #TBI: A content and network data analysis of tweets about Traumatic Brain Injury***	(a) determine how Twitter is used by people with traumatic brain injury (TBI) and TBI Organizations, (b) Analyze the Twitter networks and content of tweets tagged with TBI-related hashtags, and (c) identify any challenges people with TBI encounter in using Twitter.	Unclear	TBI	Worldwide	Mixed methods (Computational analysis; Content analysis)	Not reported	Other	Other: 893 Tweets		Social media content: Twitter hashtags	Content analysis (Computational analysis, Inductive coding of tweet content)	Hemsley et al (2014, 2017)	Not reported	Twitter was used to: (a) discuss health issues, (b) raise awareness of TBI, (c) talk about life after TBI, (d) talk about sport and concussion, and (e) communicate inspirational messages.
