# Supplementary material for: The range and reach of qualitative research in neurosurgery: A scoping review
Source: PLoS One. 2025 Aug 21;20(8):e0330770. doi: 10.1371/journal.pone.0330770 (PMC12370046; doi:10.1371/journal.pone.0330770)
Supplement: Attachment — Submitted filename: Response to reviewers.docx [file pone.0330770.s004.docx]

Please find below a summary of revisions made in responds to each of the points raised.

| Revision/query | Comment | Correction |
| --- | --- | --- |
| Editorial/journal requirements | | |
| 1. Please ensure that your manuscript meets PLOS ONE's style requirements, including those for file naming | I have reviewed the formatting conventions and updated the manuscript. I have removed additional details not requested; however, retained the funding statement on the title page. I hope this is acceptable. | A number of revisions have been made throughout the manuscript to align with the formatting requirements on the ‘title_authors_affiliations’ and ‘main body’ templates. |
| 2. Please state what role the funders took in the study | Thank you for confirming that you will change the online submission on our behalf. Please find the correct statement for inclusion. | The funders had no role in study design, data collection and analysis, decision to publish, or preparation of the manuscript |
| 3. Please amend your authorship list in your manuscript file to include author Charlotte Jane Jane Whiffin. 4. Please amend the manuscript submission data (via Edit Submission) to include author Charlotte J Whiffin. | My apologies the error has been corrected to reflect one author Charlotte J Whiffin | Charlotte J Whiffin |
| 5. We note that Figure 4 in your submission contain [map/satellite] images which may be copyrighted | Fig 4. Is a choropleth generated using GeoPanda. We have now referenced this python tool for geographic data next to the figure citation. | Fig.5  Line 233  (Geographic visualisation of citation counts per country was performed using GeoPandas [40]) |
| 6. Please remove all personal information, ensure that the data shared are in accordance with participant consent, and re-upload a fully anonymized data set. | Please may I seek further clarification on this point. The data set uploaded is a data extraction file of published evidence. Participant consent is not relevant to this scoping review. | N/A |
| 7. Please review your reference list to ensure that it is complete and correct. If you have cited papers that have been retracted, please include the rationale for doing so | The reference list has been checked and to the best of my knowledge is accurate. I am not aware of any papers that have been retracted. | References |
| Reviewer 1: |  |  |
| Reviewer #1: This is a comprehensive scoping review assessing qualitative research in neurosurgery. It addresses a gap long acknowledged in neurosurgical literature and aligns with recent calls for qualitative integration. The manuscript highlights disparities in authorship and data collection between HICs and LMICs and addresses concerns about "helicopter research" and representation. Almost 19% of the reviewed outputs influenced policy across fifteen countries - a compelling argument for qualitative research utility in neurosurgery. | Thank you for these kind comments. | N/A |
| 1. Quality Assessment:  Although scoping reviews typically avoid formal critical appraisal, a brief evaluation of methodological quality (eg Frequency of use of COREQ, audit tails, rigor indicators, reported paradigms, neurosurgeon involvement) would add weight. | Thank you for this suggestion. We have now summarised the data in the results section that are indicative of quality and referred directly to these in the discussion. | Line 369-380 |
| 2. Visual data representation  Analytical maps or conceptual/flow diagrams linking themes would enhance readability | Thank you for this helpful suggestion. As this review presents a descriptive rather than interpretive analysis, a conceptual or analytical framework was not developed. However, to enhance readability and visualise the thematic structure, we have included a sunburst diagram that illustrates the distribution of coding across themes and sub-themes within the evidence base. | Line 191 & 195  New Fig 2. Sunburst chart of themes and sub-themes |
| 3. Propose a framework for future research Based on the findings, suggest a conceptual model or framework for how qualitative research should be integrated into neurosurgery moving forward | Thank you for this thoughtful suggestion. While the primary aim of this review was to descriptively synthesise the current qualitative literature in neurosurgery, rather than develop a conceptual model, we recognise the value of guiding future research in this area. Therefore, we have added a brief section to the discussion highlighting opportunities for integrating qualitative approaches into key areas of neurosurgical research | Line 429-431 |
| 4. Highlight clinical impact examples Include a short boxed section or table showing a few high-impact studies (cited in policy, guideline-forming) and their qualitative contribution to practice change | Thank you for this helpful suggestion. We explored this recommendation in depth, including consultation with experts in bibliometric analysis. In doing so, we identified several limitations with the available SciVal data, which is based on Overton policy citation metrics.  First, our original manuscript cited the top five papers in terms of policy citations. However, on closer inspection, we found that the paper *‘I miss being me’* by Gilbert et al. (2017) was cited in only two unique policy documents, not five as initially reported. This discrepancy arose because the Overton database attributed multiple citations to the same policy document hosted on different webpages of EU organisations, thereby inflating the policy citation count.  We have therefore revised the manuscript to cite only the top four performing papers as fifth position is shared by multiple manuscripts, each cited in four policy documents, making it difficult to objectively prioritise a single additional study.  Second, we attempted to construct a summary table showing the study, citing policy, country, and year of citation, along with a qualitative review of each study’s contribution to policy or practice. However, several of the policy documents were either no longer publicly available or not written in English. This prevented a consistent and reliable analysis of the qualitative contribution of these studies to policy or guideline development.  As a result, we have not included a summary table but now address the limitations of bibliometric analysis more explicitly in the manuscript's limitations section. | Line 299  Line 450 - 453 |
| Overall, this is a strong manuscript that makes a critical and timely contribution to both neurosurgery and qualitative health research | Many thanks, we are pleased this manuscript was well-received. | N/A |
| Reviewer 2 |  |  |
| This is a well written paper that addresses a clear need in the neurosurgical literature. I commend the authors for taking on this work, in this under researched area. The international collaboration on this paper only adds to its value and should also be commended. | We are very grateful for these encouraging comments. | N/A |
